# Extended Treatment with Glial Cell Line-Derived Neurotrophic Factor in Parkinson’s Disease

**DOI:** 10.3233/JPD-191576

**Published:** 2019-05-23

**Authors:** Alan L. Whone, Mihaela Boca, Matthias Luz, Max Woolley, Lucy Mooney, Sonali Dharia, Jack Broadfoot, David Cronin, Christian Schroers, Neil U. Barua, Lara Longpre, C. Lynn Barclay, Chris Boiko, Greg A. Johnson, H. Christian Fibiger, Rob Harrison, Owen Lewis, Gemma Pritchard, Mike Howell, Charlie Irving, David Johnson, Suk Kinch, Christopher Marshall, Andrew D. Lawrence, Stephan Blinder, Vesna Sossi, A. Jon Stoessl, Paul Skinner, Erich Mohr, Steven S. Gill

**Affiliations:** a Translational Health Sciences, Bristol Medical School, University of Bristol, Bristol, UK; b Neurological and Musculoskeletal Sciences Division, North Bristol NHS Trust, Bristol, UK; c Med Genesis Therapeutix Inc., Victoria, BC, Canada; d Renishaw plc, New Mills, Wotton-under-Edge, Gloucestershire, UK; e The Wales Research and Diagnostic Positron Emission Tomography Imaging Centre (PETIC), Cardiff University, Cardiff, UK; f School of Psychology, Cardiff University, Cardiff, UK; g Department of Physics and Astronomy, The University of British Columbia, Vancouver, BC, Canada; h Djavad Mowafaghian Centre for Brain Health, Faculty of Medicine, The University of British Columbia, Vancouver, BC, Canada

**Keywords:** Glial cell line-derived neurotrophic factor, convection enhanced delivery, Parkinson’s disease, neurorestoration

## Abstract

**Background:**

Intraputamenal glial cell line-derived neurotrophic factor (GDNF), administered every 4 weeks to patients with moderately advanced Parkinson’s disease, did not show significant clinical improvements against placebo at 40 weeks, although it significantly increased [^18^F]DOPA uptake throughout the entire putamen.

**Objective:**

This open-label extension study explored the effects of continued (prior GDNF patients) or new (prior placebo patients) exposure to GDNF for another 40 weeks.

**Methods:**

Using the infusion protocol of the parent study, all patients received GDNF without disclosing prior treatment allocations (GDNF or placebo). The primary outcome was the percentage change from baseline to Week 80 in the OFF state Unified Parkinson’s Disease Rating Scale (UPDRS) motor score.

**Results:**

All 41 parent study participants were enrolled. The primary outcome decreased by 26.7±20.7% in patients on GDNF for 80 weeks (GDNF/GDNF; N = 21) and 27.6±23.6% in patients on placebo for 40 weeks followed by GDNF for 40 weeks (placebo/GDNF, N = 20; least squares mean difference: 0.4%, 95% CI: –13.9, 14.6, *p* = 0.96). Secondary endpoints did not show significant differences between the groups at Week 80 either. Prespecified comparisons between GDNF/GDNF at Week 80 and placebo/GDNF at Week 40 showed significant differences for mean OFF state UPDRS motor (–9.6±6.7 vs. –3.8±4.2 points, *p* = 0.0108) and activities of daily living score (–6.9±5.5 vs. –1.0±3.7 points, *p* = 0.0003). No treatment-emergent safety concerns were identified.

**Conclusions:**

The aggregate study results, from the parent and open-label extension suggest that future testing with GDNF will likely require an 80- rather than a 40-week randomized treatment period and/or a higher dose.

## INTRODUCTION

Glial cell line-derived neurotrophic factor (GDNF) is known for its neurorestorative and neuroprotective effects in nonhuman primate models of Parkinson’s disease [1]. However, despite promising results in early open-label clinical studies [2, 3], placebo-controlled trials testing GDNF as a disease-modifying treatment for this relentlessly progressing disease have not shown significant clinical benefit to date [4, 5]. In particular, as recently reported, treatment with fixed-dose GDNF intraputamenal infusions, administered every 4 weeks, did not show a significant improvement against placebo in OFF state Unified Parkinson’s Disease Rating Scale (UPDRS) motor score (part III) or other clinical endpoints at 40 weeks, although spatial delivery of GDNF sufficient to achieve a significant increase in [^18^F]DOPA uptake across the entire putamen was achieved [5].

The trial reported here is an open-label extension of the latter study and was performed under a separate protocol. This open label extension trial was initiated before the results from the double-blind parent investigation were known. It was developed to enable prior parent study placebo patients to receive GDNF, to gain longer term safety data, and to gather further exploratory information on GDNF clinical effects following a longer period of repeated tissue exposures. Recognizing the caveats of an open label clinical trial, it was hoped that this data may help to explore the effects of GDNF when administered every 4 weeks via a skull-mounted port, in a manner to achieve bilateral intraputamenal convection-enhanced delivery (CED), for a total of 80 weeks.

## MATERIALS AND METHODS

### Study design and structure

This single-center, open-label trial of intermittent bilateral intraputamenal infusions of GDNF administered via CED was a direct continuation of the preceding randomized double-blind parent study recently reported [5]. All parent study completers had the option to enroll in the extension investigation that was conducted at the same center in Bristol, UK.

Post-screening in the parent study, eligible patients underwent robot-assisted surgery for stereotactic implantation of the in-house CED system specified by the lead neurosurgeon (SSG), comprising four separate intraputamenal infusion catheters and a single skull-mounted transcutaneous port [5].

All patients were treated with GDNF intraputamenal infusions every 4 weeks using the dose regimen and intermittent infusion parameters employed in the parent study [5]. In an attempt to minimize the bias associated with the open design, treatment allocation during the parent study was only disclosed to patients after the database for the extension trial was locked. While being aware that they were receiving GDNF in the extension study, patients did not know, therefore, whether they had been on GDNF or placebo in the parent study. Furthermore, the only information on parent study outcome that was provided to participants or made publicly available before locking the extension study database was that the primary endpoint of the double-blind study had not been met. As in the parent study, throughout the extension trial, trained raters blinded to all other aspects of the patients’ condition and their prior treatment assignments performed motor scoring.

The study was registered through the EU Clinical Trials Register (EudraCT Number, 2013-001881-40). Local institutional approval was obtained including protocol approval, the study was executed in accord with the Helsinki Declaration of 1975 and all patients provided written informed consent. The Trial Steering Committee and an independent Data Monitoring Committee provided clinical oversight. The authors vouch for the accuracy and completeness of the data and for adherence to the study protocol (see Supplementary Material, Appendix A, for study protocol first and final versions as well as a summary of protocol amendments).

### Patients

From October 2013 through to April 2016, all 41 patients completing the parent study were screened for participation in the extension trial (see Supplementary Material, Appendix B, for CONSORT flow diagram). At entry in the parent study, patients were between 35 and 75 years old and presented with motor symptom duration ≥5 years, moderate disease severity (Hoehn and Yahr stage 2-3 and UPDRS motor score 25–45, both in a practically defined OFF state), motor fluctuations (average of at least 2.5 hours of OFF time per day on 3-day fluctuation diaries), and levodopa responsiveness defined as ≥40% improvement in UPDRS motor score following a levodopa challenge. Exclusion criteria in the extension study were early discontinuation of treatment or significant protocol deviation in the parent study, presence of clinically significant depression, cognitive decline (Montreal Cognitive Assessment [MoCA] < 24), or any new medical condition that might impair outcome measure assessments or safety.

### Study procedures and assessments

The schedule for study procedures and assessments in the parent study was continued in the extension. Where applicable, visits are designated both by the consecutive week number from baseline in the parent study and the extension study week number (denoted by “e”).

Patients returned within a week after completing the parent study to receive the first of 10 scheduled GDNF treatments at 4-week intervals (Weeks 40/e0 to 76/e36). At each treatment, 400*μ*L infusate (300*μ*L GDNF followed by 100*μ*L artificial CSF [aCSF]) were delivered per each of the 4 catheters. The concentration of GDNF in the infusate was 0.2*μ*g/*μ*L, and the total dose of GDNF given per 4 weeks was 240*μ*g (120*μ*g per putamen).

The protocol stated that Parkinson’s medication was to be kept stable during the study where possible but could be modified if required for symptom control.

Every 8 weeks, starting at Week 48/e8, patients completed 3-day diary recordings and underwent assessments of motor function in the practically defined OFF state and post an L-dopa challenge. All patients had been previously trained in the completion of PD diary recordings. Motor assessments were performed immediately prior to the GDNF administrations to reduce the risk of potential symptomatic effects from putamenal infusions *per se*. Other efficacy outcome measures were assessed at wider intervals (see study protocol, Supplementary Material, Appendix A). Samples for anti-GDNF antibodies and GDNF plasma concentrations were collected throughout the study. As in the parent investigation, catheter performance was verified at Week 80/e40 through an intraputamenal test infusion of a 2 mM solution of gadolinium in aCSF followed by MRI scan. [^18^F]DOPA positron emission tomography (PET) scans were not acquired in the extension study.

### Study outcomes

The primary endpoint of the study was the percentage change from baseline (Week 0) to Week 80/e40 in the practically defined OFF state UPDRS motor score, comparing the group that had received GDNF in the parent investigation followed by open-label GDNF (GDNF/GDNF) versus the group that received placebo in the parent investigation followed by open-label GDNF (placebo/GDNF).

Secondary endpoints included absolute change from baseline in OFF state UPDRS motor score, as well as absolute and percentage change from baseline in UPDRS motor score in the ON state and UPDRS activities of daily living (ADL; part II) and total (sum of motor and ADL) scores in the OFF and ON state, change in UPDRS part I and IV scores, and change from baseline in Parkinson’s disease diary ratings. A further prespecified secondary endpoint included comparing Week 80/e40 UPDRS scores in the GDNF/GDNF group against Week 40 scores in the placebo/GDNF group (i.e., at the end of the placebo treatment).

Supplementary endpoints included timed motor tests in both OFF and ON state, total daily levodopa and levodopa equivalent dose, the non-motor symptom scale for PD (NMSS), cognitive, mood and impulsivity measures, the University of Pennsylvania smell test (UPSIT), and Parkinson-related quality of life questionnaires (PDQ-39 and EQ-5D). Patients’ satisfaction and impact on quality of life in relation to the delivery device were not specifically explored.

A prospective responder analysis was performed to identify patients demonstrating a≥10 absolute points improvement in OFF state UPDRS motor score and/or a≥1-hour gain in total good-quality ON time per day. Good-quality ON time was defined as ON time with either no dyskinesia or non-troublesome dyskinesia as per the patient-reported diaries. *Post hoc*, it was decided to also include patients showing ≥5 points improvement in OFF state ADL score in the responder analysis.

UPDRS motor and ADL score assessments, timed taps and timed walks were completed by three trained raters who were blinded to all other aspects of the patients’ condition and remained blinded to the treatment allocation during the preceding double-blind parent trial. Wherever possible, the same rater that performed the baseline (Week 0) assessment also performed the Week 80/e40 assessment. All OFF assessments were performed at a similar time in the morning, following withholding of long-acting PD medications the day before and all other Parkinson’s disease medications from 6 pm the evening before.

During screening for the parent investigation, patients were trained on the completion of the Parkinson’s disease diary and had to demonstrate their ability to accurately determine their ON/OFF state as part of the inclusion criteria.

Imaging endpoints included change from baseline to Week 80/e40 in gadolinium-evidenced volume of infusate distribution, putamenal volume of interest coverage (VOI; the dorsal two thirds of the posterior putamen) and total putamenal coverage as assessed on T1-weighted MRIs.

Safety was assessed on the basis of adverse events (AEs), routine laboratory testing and anti-GDNF antibodies. All AEs were considered treatment-emergent. Dyskinesias, falls, adverse changes in mood, and impulsivity were tabulated as AEs of special interest. Furthermore, patients were monitored for cognitive function (MoCA and Mattis Dementia Rating Scale) and signs of impulsive or compulsive behavior (questionnaire for impulsive-compulsive disorders in Parkinson’s disease).

### Statistical analysis

Statistical analyses, as prespecified in the statistical analysis plan (SAP; see Supplementary Material, Appendix C for first and final versions of SAP as well as a summary of SAP amendments), were conducted with the use of Statistical Analysis System (SAS) software, version 9.4 (SAS Institute). Any hypothesis testing was performed with a 2-sided alternative at an alpha level of 0.05. No adjustments for multiplicity were made. Since the sample size was predefined by the parent study, no formal sample size calculations were made.

The parent study consisted of a Pilot Stage cohort (N = 6) and a Primary Stage cohort (N = 35) and included patients randomized after reaching post-surgical eligibility criteria. In the parent study analyses, it was found that the Pilot Stage and Primary Stage populations differed only slightly in baseline characteristics and outcome at Week 40 [5]. Hence, for this exploratory open-label extension study, it was decided prospectively to shift the focus of the efficacy analyses to the overall population to make best possible use of the data from all 41 patients. Thus, the data presented in tables and figures here are for the entire population. Results for the Primary Stage cohort alone, however, are presented in Supplementary Material, Appendix D.

The primary endpoint was compared between treatment groups using a mixed-effect model with repeated measures (MMRM) adjusted for the baseline value. Secondary and supplementary efficacy endpoints were analyzed using either the MMRM or an analysis of covariance (ANCOVA) model adjusted for the baseline value of the respective assessment. Treatment response rates were analyzed by means of Fisher’s exact test, and correlation testing was done with non-parametric Spearman rank correlation analyses. No corrections for multiple comparisons were made, and no hierarchical approach to the secondary endpoints was employed.

## RESULTS

### Patients

All 41 patients randomized and treated in the parent study were enrolled and completed the extension study. Demographic and baseline Parkinson’s disease characteristics are summarized in Table 1.

**Table 1:** Demographic and Parkinson’s Disease Characteristics at Screening

Characteristic	GDNF/GDNF (N = 21)	Placebo/GDNF (N = 20)
Age – years	55.9±8.8	54.3±7.6
Male sex - no. (%)	9 (42.9)	13 (65.0)
Race - no. (%)
White	21 (100)	19 (95.0)
Asian	0	1 (5.0)
OFF-state Hoehn and Yahr stage - no. (%)
Stage 2	11 (52.4)	5 (25.0)
Stage 2.5	4 (19.0)	9 (45.0)
Stage 3	6 (28.6)	6 (30.0)
Disease duration – years
Since first motor symptom	10.6±5.0	10.6±5.5
Since original diagnosis	8.6±4.4	7.9±3.5
UPDRS motor score
OFF-state	36.0±7.7	36.3±6.2
ON-state	15.7±5.8	16.6±4.6
Levodopa response - % ^a^	56.9±11.3	54.2±10.0
OFF-time per day – hours	6.3±2.0	6.0±2.0

^a^Percentage improvement in UPDRS motor score following a levodopa challenge. As shown in Table 1, the baseline groups appeared well matched. There were, however, two variables with noticeable differences between the groups: gender and H&Y score. Regarding gender, we had 9 (42.9%) GDNF males vs. 13 (65.0%) Placebo males in the study. We do not think that this biased the results, however, as there is nothing in the literature to indicate gender bias with GDNF treatment. With regard to H&Y score, we had 11 GDNF vs. 5 Placebo H&Y score 2 patients and 4 GDNF vs. 9 Placebo H&Y score 2.5 patients. The relevance of these chance differences is likely small and we think it reassuring that the aggregate of H&Y score 2 and 2.5 patients was similar in both groups (15 GDNF vs. 14 Placebo), and that H&Y score 3 was the same in both groups (6 vs. 6). Furthermore, we performed *post hoc* covariate analyses for a number of covariates including both gender and H&Y score at screening and the treatment effect was neither enhanced nor diminished when assessing these single covariates.

### Drug delivery

As in the parent study, compliance was very high: 401 (97.8%) of 410 scheduled intraputamenal GDNF infusions were administered as an outpatient procedure during the extension trial period. Altogether, 48 (12.0%) infusions were interrupted or terminated early, predominantly as a safety shutdown due to mechanical pressure increases in one or several infusion lines.

Mean gadolinium-evidenced coverage on MRI at study end (Week 80/e40) was consistently high across the left and right hemispheres in both treatment groups (ranging from 67.9±21.9% to 74.3±14.5% for putamenal VOI coverage, and from 48.3±21.2% to 57.2±21.5% for total putamen coverage). These values are similar to the coverage observed at the end of the parent study (Week 40) [5].

### Clinical outcomes

From baseline (Week 0) to the end of treatment (Week 80/e40), the OFF state UPDRS motor score improved by 26.7±20.7% (mean±standard deviation) in the GDNF/GDNF group and by 27.6±23.6% in the placebo/GDNF group, with no significant difference between the groups (least squares [LS] mean difference: 0.4%, 95% CI: –13.9, 14.6; *p* = 0.96; Table 2); therefore, the primary outcome of the extension trial was not met.

**Table 2:** Efficacy Outcomes

Outcome Category Variable	GDNF/GDNF (N = 21)			Placebo/GDNF (N = 20)			Least Squares Mean Difference vs. Placebo (95% CI); p
	Baseline	Week 80	Change	Baseline	Week 80	Change	
UPDRS scores
Motor (III) OFF	36.0±11.7	26.4±11.3	–9.6±6.7	32.2±8.3	23.2±9.0	–9.0±7.8	–0.0 (–4.4, 4.4); 0.99*
			–26.7±20.7%			27.6±23.6%	0.4% (–13.9, 14.6); 0.96*
Motor (III) ON	16.3±5.6	15.0±6.0	–1.4±4.8	16.3±7.2	14.8±6.0	–1.6±4.1	–0.2 (–2.9, 2.6); 0.91*
			–7.0±32.3%			5.1±22.7%	–3.7% (–21.7, 14.2); 0.67*
ADL (II) OFF	18.5±6.4	11.7±4.9	–6.9±5.5	16.9±5.8	12.3±6.6	–4.6±4.7	–1.7 (–4.6, 1.2); 0.25*
			–34.3±22.3%			28.2±26.2%	–4.4% (–20.2, 11.4); 0.58*
ADL (II) ON	5.5±4.1	2.9±3.0	–2.6±4.2	5.7±3.7	3.9±3.2	–1.8±3.5	–0.8 (–3.0, 1.3); 0.43*
			–33.9±62.6%			32.3±52.0%	–3.3% (–40.1, 33.5); 0.86*
Total (II+III) OFF	55.0±16.7	37.9±14.9	–17.1±8.6	49.1±11.0	35.5±13.0	–13.6±10.0	–2.6 (–8.3, 3.2); 0.37*
			–31.3±14.8%			–28.3±19.8%	–3.3% (–14.6, 8.0); 0.56*
Total (II+III) ON	21.8±8.4	17.4±7.6	–4.4±6.9	22.0±8.5	18.6±6.4	–3.3±6.1	–1.6 (–5.3, 2.2); 0.40*
			–17.5±31.9%			–11.3±23.1%	–7.9% (–25.7, 9.9); 0.37*
Timed tapping – no.
OFF-state	43.1±15.0	63.8±22.6	20.7±15.4	42.4±9.4	59.1±17.9	16.7±13.0	3.9 (–5.1, 12.9); 0.39*
ON-state	64.2±17.6	79.9±22.7	15.7±13.6	61.0±17.4	73.6±19.2	12.7±10.0	3.4 (–4.2, 11.0); 0.37*
Timed walking – sec
OFF-state	58.4±97.3	27.6±52.0	–27.0±106.5	17.6±10.8	11.7±2.9	–4.3±6.4	3.4 (–25.5, 32.3); 0.81*
ON-state	11.0±2.6	10.4±1.8	–0.6±1.8	10.4±1.9	9.8±1.6	–0.7±1.4	0.3 (–0.5, 1.0); 0.47*
Motor fluctuation diary ratings – hrs
Total OFF-time	6.1±1.7	4.5±1.8	–1.5±1.4	4.8±2.2	4.0±2.1	–0.8±2.8	–0.2 (–1.4, 1.1); 0.80*
Good-quality ON-time	10.2±2.0	11.8±2.2	1.6±1.5	12.5±2.6	13.1±3.1	0.5±3.0	0.6 (–1.0, 2.1); 0.46*
ON-time with troublesome dyskinesias	0.6±1.2	0.4±1.0	–0.2±0.8	0.5±1.0	0.4±0.7	–0.1±1.2	–0.1 (–0.6, 0.5); 0.80*
Total daily dose – mg
L-DOPA	639±306	675±310	36±186	561±284	721±391	160±230	–121 (–256, 14); 0.08^†^
L-DOPA equivalent	1,011±340	1,071±396	59±194	954±383	1,243±552	289±365	–233 (–419, –47); 0.02^†^

*MMRM with baseline variable as a covariate, treatment group and visit and treatment group*visit as fixed effects, and patient within treatment group as a random effect. ^†^ANCOVA model with baseline variable as a covariate and treatment group as a factor. Notes: (1) One GDNF/GDNF patient had a conus injury due to a car accident and was included in the UPDRS motor scores without items 22, 27, 28, 29, and 30. The same patient was excluded from the UPDRS ADL and total scores. (2) Timed tapping numbers are averages of left and right. (3) UPDRS parts I and IV, EQ-5D, body weight, Simplified Nutritional Appetite Questionnaire, Questionnaire for Impulsive-Compulsive Disorders in Parkinson’s disease, Montreal Cognitive Assessment, Mattis Dementia Rating Scale, Stroop test, Frontal Systems Behavioral Scale, Deary-Liewald reaction time, verbal fluency assessment, Beck Depression Inventory, and University of Pennsylvania Smell Identification Test remained essentially unchanged between baseline and Week 80 in both groups and did not reveal any significant treatment differences between GDNF/GDNF and placebo/GDNF.

Likewise, none of the secondary or supplementary outcomes spanning the entire 80-week period, except for change in L-DOPA equivalent dose, showed a significant difference, in either percentage or absolute change from baseline to Week 80/e40, between the GDNF/GDNF group and the placebo/GDNF group (Table 2). As an exception, the increase in the daily L-DOPA equivalent dose from baseline to Week 80/e40 was smaller in the GDNF/GDNF group (59±194 mg) than in the placebo/GDNF group (289±365 mg, LS mean difference: –233 mg, 95% CI: –419, –47; *p* = 0.02, Table 2). The mean total daily L-DOPA dose increased by 36±186 mg from baseline in the GDNF/GDNF group as compared to 160±230 mg in the placebo/GDNF group (*p* = 0.0769).

**Fig.1:**
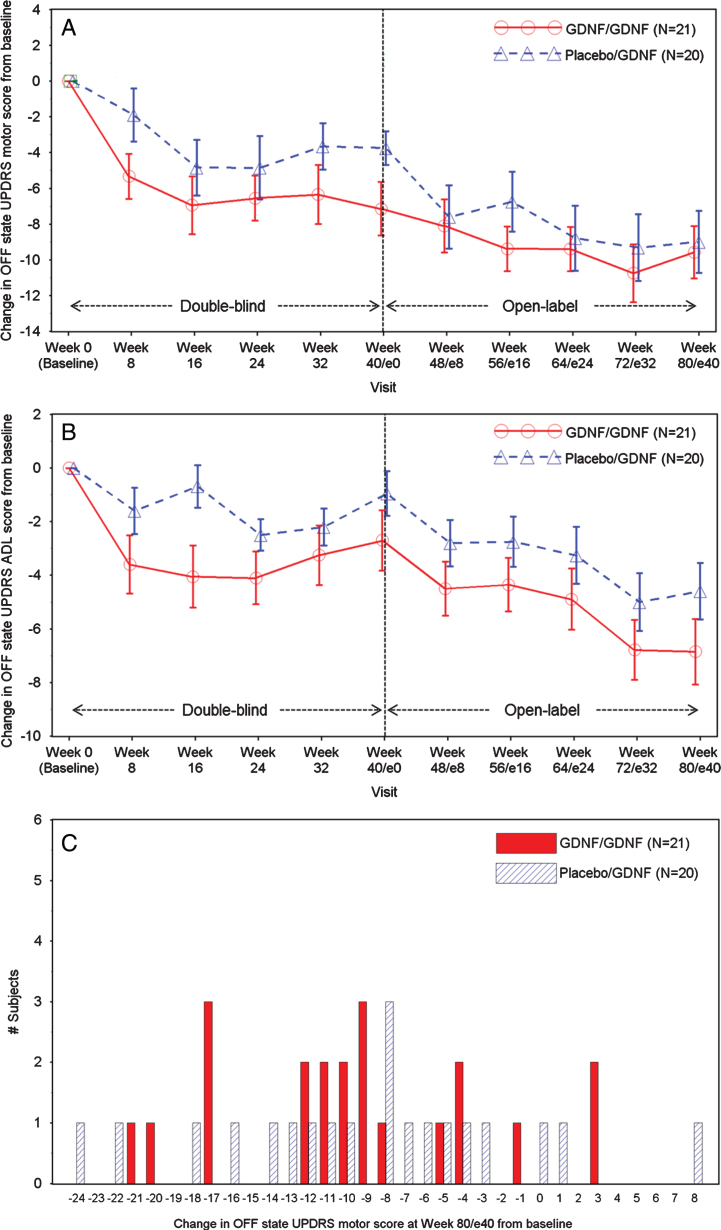
A. OFF State UPDRS Motor Score: Change over Time. Note: Data points represent means, and error bars represent standard errors. One GDNF/GDNF patient had a conus injury due to a car accident and was included in the motor score without items 22, 27, 28, 29, and 30. B. OFF State UPDRS ADL Score: Change over Time. Note: Data points represent means, and error bars represent standard errors. One GDNF/GDNF patient had a conus injury due to a car accident and was excluded from the analysis. C. OFF State UPDRS Motor Score: Frequency Distribution of Change at Week 80/e40. Note: One GDNF/GDNF patient had a conus injury due to a car accident and was included in the motor score without items 22, 27, 28, 29, and 30.

By Week 80/e40, the OFF state UPDRS motor score improved by 9.6±6.7 points in the GDNF/GDNF group and by 9.0±7.8 points in the placebo/GDNF group (Table 2, Fig. 1A). Compared to baseline, improved or stable OFF state UPDRS motor scores were seen in 37/41 patients (90%) across both treatment groups (Fig. 1C).

The OFF state UPDRS ADL score showed mean absolute improvements from baseline of 6.9±5.5 points in the GDNF/GDNF group and by 4.6±4.7 points in the placebo/GDNF group by Week 80/e40 (Table 2, Fig. 1B). Absolute changes in OFF state UPDRS motor and ADL scores in the GDNF/GDNF group at Week 80/e40 were significantly larger than the corresponding changes in the placebo group at Week 40 (motor: –9.6±6.7 vs. –3.8±4.2 points, *p* = 0.0108; ADL: –6.9±5.5 vs. –1.0±3.7 points, *p* = 0.0003).

**Fig.2:**
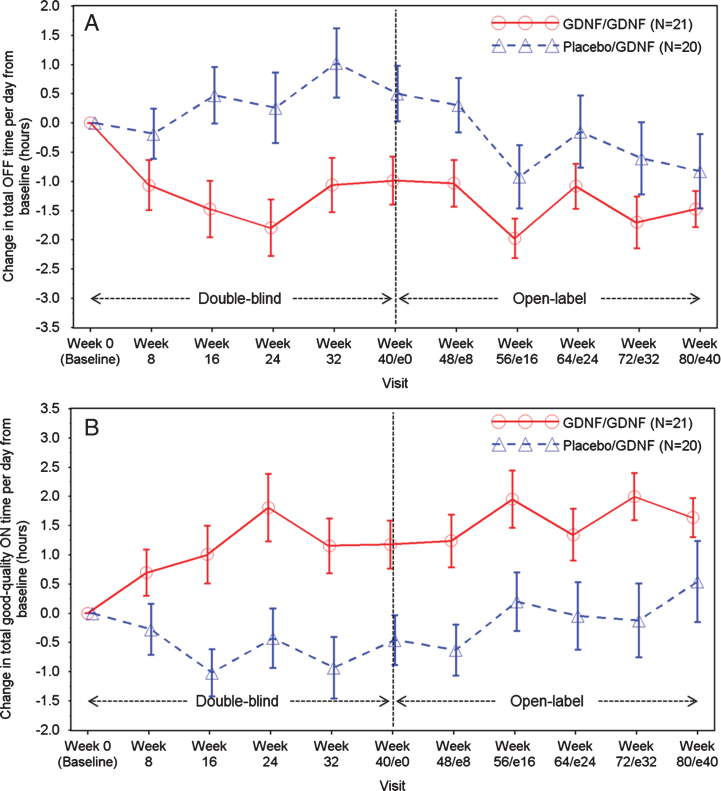
A. Total OFF Time per Day: Change over Time. Note: Data points represent means, and error bars represent standard errors. B. Total Good-Quality ON Time per Day: Change over Time. Note: Data points represent means, and error bars represent standard errors. Total good-quality ON time per day is defined as the sum of ON time per day without dyskinesias + ON time per day with non-troublesome dyskinesias.

Improvements in total OFF time and good-quality ON time per day were observed in both groups during the extension study (Table 2, Fig. 2A-B). Compared to baseline, mean total OFF time per day fell by 1.5±1.4 hours in the GDNF/GDNF group and by 0.8±2.8 hours in the placebo/GDNF group. Good-quality ON time increased by 1.6±1.5 hours in the GDNF/GDNF group and by 0.5±3.0 hours in the placebo/GDNF group.

In a responder analysis at Week 80/e40, 11 (52%) GDNF/GDNF patients and 9 (45%) placebo/GDNF patients showed a≥10 absolute points improvements in OFF state UPDRS motor score from baseline. The proportion of patients showing such a response after only 40 weeks of treatment with GDNF by the end of the extension study (placebo/GDNF group at Week 80/e40) was therefore similar to that seen in the GDNF-receiving patients at Week 40 in the parent study (GDNF/GDNF group at Week 40:9/21 [43%]).

Twenty (95%) GDNF/GDNF patients and fourteen (70%) placebo/GDNF patients reached or exceeded thresholds for clinically meaningful improvements by Week 80/e40 compared to baseline in OFF state UPDRS motor score (≥10 points), and/or OFF state UPDRS ADL score (≥5 points) and/or good-quality ON time per day (≥1 hour) [6, 7]. The numbers of patients who reached / exceeded thresholds in three or two of the above outcomes (triple and double responders at Week 80) were: 15 (71.4%) of 21 patients in the GDNF/GDNF group (4 triple responders, 11 double responders) vs. 8 (40%) of 20 patients in the placebo/GDNF group (5 triple responders, 3 double responders.

## SAFETY

AEs were reported for all 41 patients between Week 40 and Week 80/e40 (Table 3). No patient had an AE leading to discontinuation of study medication. As in the parent study, the most frequently reported AEs (10 or more patients overall) included dyskinesia, Lhermitte’s sign, paraesthesia, fall, ON/OFF phenomenon, and freezing. No differential pattern was discernible between the treatment groups, and overall frequencies were similar to the GDNF group by the end of the parent study [5]. The overall frequency of AEs of special interest was similar in both treatment groups (GDNF/GDNF: 76%, placebo/GDNF: 85%).

**Table 3:** Adverse Events Experienced by at Least 5 Patients Overall

Adverse Event – no. (%)	GDNF/GDNF (N = 21)	Placebo/GDNF (N = 20)	Total (N = 41)
Patients with at least one AE	21 (100)	20 (100)	41 (100)
Dyskinesia	8 (38)	9 (45)	17 (42)
Lhermitte’s sign	9 (43)	4 (20)	13 (32)
Nasopharyngitis	7 (33)	6 (30)	13 (32)
Paresthesia	6 (29)	7 (35)	13 (32)
Fall	5 (24)	7 (35)	12 (29)
ON and OFF phenomenon	4 (19)	7 (35)	11 (27)
Freezing phenomenon	7 (33)	3 (15)	10 (24)
Application site infection	5 (24)	4 (20)	9 (22)
Dystonia	5 (24)	4 (20)	9 (22)
Headache	4 (19)	5 (25)	9 (22)
Back pain	3 (14)	5 (25)	8 (20)
Muscle spasms	2 (10)	6 (30)	8 (20)
Contusion	4 (19)	3 (15)	7 (17)
Pain in extremity	5 (24)	2 (10)	7 (17)
Urinary tract infection	3 (14)	4 (20)	7 (17)
Application site erythema	2 (10)	4 (20)	6 (15)
Dizziness	3 (14)	3 (15)	6 (15)
Joint injury	4 (19)	2 (10)	6 (15)
Nausea	4 (19)	2 (10)	6 (15)
Constipation	2 (10)	3 (15)	5 (12)
Drug effect decreased	2 (10)	3 (15)	5 (12)

Serious AEs were reported for 8 (20%) patients overall (GDNF/GDNF: 7, placebo/GDNF: 1), and were all judged to be unrelated to study medication: device-related events (3), traumatic muscle rupture (1), menorrhagia requiring hysterectomy and postoperative infection (1), multifactorial confusion and fluctuating cognition (1), recurrence of pre-study depression and paranoia (1), and osteoarthritis (1).

Most frequent device-related AEs included application site erythema and infection. No intracranial infections occurred during the extension study trial.

Quantifiable GDNF plasma concentrations were found in 10 (5%) blood samples from 3 patients between Week 40 and Week 80/e40. However, no GDNF-binding serum antibodies were identified in the study at any time.

## DISCUSSION

We did not observe significant differences in the primary or secondary outcome measures spanning the entire 80-week period between the group receiving GDNF for 80 weeks and the group receiving placebo for the first 40 weeks followed by GDNF for the second 40 weeks (Table 2). It is probably not surprising, however, that the primary and secondary endpoints were not met given that there were no significant differences between the primary or secondary clinical outcomes in the double-blind parent investigation. In such a situation, it was unlikely that differences between groups would emerge while treating both groups with open-label GDNF for 40 weeks.

The extension study was initiated approximately two and a half years before the parent study was completed and read out. While patients knew that they were on GDNF in the extension trial, they were kept blinded to their treatment assignment in the parent study. However, the extension study was neither conceived nor powered as a delayed-start investigation of the type previously employed to assess putative neuroprotective agents in Parkinson’s disease [8]. Indeed, such a design may not be suitable for an agent that, like GDNF, is hypothetically capable of both neurone restoration and protection and thus, may lead to clinical improvement rather than disease stabilization alone.

An important question is whether the two studies, parent and extension, in aggregate, have fully tested the growth factor hypothesis with respect to the neurorestorative potential of GDNF. When discussing the results of the parent study [5], we raised the possibility that clinical effects may, at least in part, lag behind biological changes during disease reversal or need a longer period of repeated exposure to drug to develop. Such a supposition is based on the premise that whilst functional changes due to restoration of the dopamine phenotype of affected neurons may occur early [9], there may be delays between terminal sprouting, synapse formation and circuit reestablishment, and subsequent improvement in measurable clinical function [10].

The above would imply that there should be continued or added benefit during the second 40 weeks of treatment with GDNF. The limitation to testing this implication is that we no longer have the benefit of a control arm, due to the open label active treatment nature of the extension study, and hence any clinical benefit must be judged in that light. That said, we can note that although there was no statistical difference in OFF state UPDRS motor and ADL scores between the groups at Week 40 [5], the corresponding Week 80 results in the GDNF/GDNF group were significantly different from the Week 40 results in the placebo/GDNF group. This, however, could have been due to a second placebo effect as patients moved into the Extension Study, from which point they knew they were receiving GDNF whatever they had received in the first 40 Weeks. This pre-specified analysis was undertaken to compare the effects of treatment with GDNF over 80 weeks against 40 Weeks of placebo and was performed because up to 40 Weeks of placebo (the end of the double-blind study) is the longest period of treatment with placebo alone data we have. Whilst we recognise this does not serve as a control it is an additional comparison to consider whilst accepting its limitations. The curves for OFF state UPDRS motor score converge over the course of the extension study (Fig. 1A), and whilst the curves for OFF state UPDRS ADL score (Fig. 1B) and Parkinson’s disease diary-based outcomes (Fig. 2A, B) do not converge, they do not show significant differences between the groups at Week 80 either. In consequence, the above raised question of whether clinical effects lag behind biological changes during disease reversal or need a longer period of repeated exposure to drug to develop cannot be answered definitively on the basis of the extension study results. Conceivably, however, higher GDNF doses and/or a longer duration of repeated tissue exposure would have been required to show benefit in the parent investigation [5].

The mean improvements from baseline to Week 80 in OFF state UPDRS motor and ADL scores and Parkinson’s disease diary ratings were equivalent to moderate-to-large clinical effects [6, 7], although we recognise that such magnitude of change could represent a placebo response in an open label investigation where patients knew they were receiving active treatment. Given the requirement for a neurosurgical approach, some would suggest it might be appropriate to consider the magnitude of the symptomatic improvement achieved with deep brain stimulation (DBS) as a reference. In a recent open-label DBS study that included a not dissimilar patient cohort, the VANTAGE investigation, improvements from baseline in OFF state motor UPDRS and ADL scores and patient diaries were seen at 52 weeks that were approximately twofold larger than with GDNF at 80 weeks [11]. DBS, however, is a matured therapy and the symptomatic improvement achieved with DBS is not hypothesised to be via a neurorestorative mechanism and hence comparison with an experimental disease-modifying approach is potentially not valid.

Placebo effects are well recognized in Parkinson’s disease [12]. The size of these effects may reflect the magnitude of the intervention and the anticipated benefits, and could therefore be large in a study involving initial neurosurgery followed by intraputamenal infusions via a skull-mounted port every 4 weeks over a total of 80 weeks [13–15]. In addition, a second placebo response may have occurred as the patients switched from double-blind treatment to known open-label GDNF therapy in the extension study [16]. In this context, it is worth noting that the new occurrence of certain AEs, in particular Lhermitte’s phenomenon or paraesthesias, may have signalled to patients that their treatment changed in the extension trial. Likewise, investigator bias, once it was known that patients were receiving GDNF, may have played a part in the development of the clinical benefits over baseline observed after 18 months. It is also possible that the overall placebo response was enhanced by putamenal tissue disruption as a result of catheter implantation and repeated high-pressure CED infusions, since mechanical injury has been shown to induce dopaminergic axonal sprouting and increase dopaminergic activity in the nigrostriatal system, primarily via a GDNF- and BDNF-dependent mechanism mediated by activated macrophages and microglia [17–19]. In future studies, it may be useful to consider the addition of a standard-of-care arm and/or the replacement of the placebo arm (which may in fact be an experimental treatment arm) with a sham surgery arm to derive a better understanding of these mechanisms and the true effect size attributable to GDNF.

In the discussion of the parent study paper [5], we also contemplated that the inability to prove clinical benefit at 40 weeks may, at least in part, be related to the GDNF dose used in the trial. This question remains unanswered at the end of the extension study. Dosing is a complex issue in the context of intermittent CED, and while we did integrate the available preclinical and clinical information when defining the dose and delivery scheme, it is worth noting that although double the prior tested GDNF concentration was employed, (0.2*μ*g/*μ*L in this and the allied parent investigation versus 0.1*μ*g/*μ*L in previous clinical trials [2–4]), the total dose given every 4 weeks (240*μ*g; 120*μ*g/putamen) was less than one third of the monthly cumulative dose administered in previous studies which employed continuous dosing of GDNF via abdominal pumps [2–4]. This is potentially important considering that the neurorestorative effects of GDNF on its target neurons are known to be dose-dependent and tissue-exposure dependent [20, 21]. Using a higher dose with intermittent administration, however, would have required a higher infusate GDNF concentration (e.g., 0.6*μ*g/*μ*L) which could not be implemented before addressing concerns about unexpected cerebellar toxicity in rhesus monkeys that had been treated with a similar concentration, albeit in a continuous dosing study and at a total dose well beyond the clinical range [22]. In the meantime, the safety of the higher concentration, when used intermittently, has been confirmed [23], thus enabling its clinical use in the future.

We are aware of the history of AAV2-neurturin, which underwent clinical retesting based on “supportive” and *post-hoc* evidence from a Phase II study that did not reach its primary endpoint [24]. This further AAV2-neurturin trial, however, which included a higher dose and longer treatment duration, again generated negative data [25]. That said, there are several important differences between the GDNF and the AAV2-neurturin programmes, including the improvement in [^18^F]DOPA uptake with GDNF, that was not seen with AAV2-neurturin, and the spatial distribution achieved by the intermittent CED paradigm employed with GDNF.

The breadth of response to an infused trophic factor in a broad-spectrum disorder such as Parkinson’s disease is a priori likely to be wide and the factors determining the limits of any potential gain in an individual patient are yet to be established. While the covariate analyses in the parent study did not reveal any characteristics that predicted benefit, it is potentially noteworthy that 95% of patients receiving GDNF for 80 weeks passed the thresholds for meaningful clinical improvements in one or more of the core outcome measures [6, 7]. In a disorder which to date has no approved therapy for disease modification, this potential hint at broad-spectrum applicability is not unimportant. It is conceivable that certain neurorestorative or neuroprotective approaches may be more applicable to selected subgroups of Parkinson’s patients depending on the nature of the underlying molecular dysfunction. Therapies targeting protein aggregation, mitophagy or inflammation may require patient stratification where the target is a top-down driver of disease progression. Trophic factors, including GDNF, however, may not necessarily require personalized stratification to be beneficial given the potential for improvement via a final common pathway.

Intermittent intraputamenal administration of GDNF at the selected dose (240*μ*g every 4 weeks) over 80 weeks was well tolerated and safe and, contrary to the historic continuous dosing Phase II study [4], did not induce anti-GDNF antibody formation. Treatment compliance was high (97.8%) and there were neither drop-outs during the study nor any untoward problems with OFF state dyskinesias of the type reported in a previous foetal graft study [26].

In conclusion, neither the parent nor the extension study reached their primary endpoints. The integrated results of the two studies suggest that: Attending on an out-patient basis over 18 months, to receive intraputamenal infusions every 4 weeks via a skull-mounted port, is feasible; As evidenced by increased [^18^F]DOPA PET findings in the parent study, our novel method of administration enables a putamen-wide target tissue engagement; Intermittent intraputamenal administration of GDNF (240*μ*g every 4 weeks) for 80 weeks is well tolerated; and, Further testing of the growth factor hypothesis in a larger-scale study will likely require 80 weeks of randomized treatment and / or a higher dose to definitively determine whether GDNF has a future role as a neurorestorative treatment for Parkinson’s disease.

## CONFLICT OF INTEREST

Matthias Luz, Lara Longpre, Chris Boiko, Greg A. Johnson, H. Christian Fibiger and Erich Mohr are employed by and have shares and/or share options with MedGenesis Therapeutix Inc., owners of the license for GDNF. Lynn Barclay is a consultant to MedGenesis Therapeutix, Inc. Max Woolley, Rob Harrison, Owen Lewis, Gemma Pritchard, Mike Howell, Charlie Irving, David Johnson, Suk Kinch and Paul Skinner are employees of Renishaw plc, contract manufactures of the drug-delivery system. Steven Gill is the Medical Director of Renishaw plc and is the inventor of the drug delivery system from which he may have a future royalty share. He is on the scientific advisory board of MedGenesis Therapeutix Inc. for which he is reimbursed with share options. No other potential conflict of interest relevant to this article was reported.

## Supplementary Material

Supplementary Material Appendix A – Study protocol: 1) first version, 2) final version and 3) summary of protocol amendments

Supplementary Material Appendix B – CONSORT flow diagram

Supplementary Material Appendix C – Statistical analysis plan: 1) first version, 2) final version, 3) summary of SAP amendments, 4) issues identified in SAP, 5) memo to file to explain issues identified

Supplementary Material Appendix D – Efficacy Outcomes for Primary Stage cohort
