# Supplementary material for: Extended Treatment with Glial Cell Line-Derived Neurotrophic Factor in Parkinson’s Disease
Source: J Parkinsons Dis. 2019 May 23;9(2):301–13. doi: 10.3233/JPD-191576 (PMC6597995; doi:10.3233/JPD-191576)
Supplement: Supplementary Material — Appendix A – Study protocol: 1) first version, 2) final version and 3) summary of protocol amendments [file jpd-9-jpd191576-s001.pdf]

Study Title

An Extension Study to Assess the Safety and Efficacy of Intermittent Bilateral Intraputamenal Glial Cell Line-Derived Neurotrophic Factor (GDNF) Infusions Administered via Convection Enhanced Delivery (CED) in Subjects with Parkinson's Disease

Internal Reference No: 2797

Ethics Ref: 13/SW/0181

EudraCT Number: 2013-001881-40

Date and Version No: Version 1.0, 18 June 2013

Principal Investigator: Dr A Whone
Consultant Neurologist and Hon Senior Lecturer
Department of Neurology and the Burden Institute
Frenchay Hospital, North Bristol NHS Trust
Bristol BS16 1LE, United Kingdom

Study Neurosurgeon: Professor S Gill
Consultant Neurosurgeon
Department of Neurosurgery
Frenchay Hospital, North Bristol NHS Trust
Bristol BS16 1LE, United Kingdom

Sponsor: North Bristol NHS Trust (NBT)
Research & Innovation
Floor 3, Learning & Research Building
Bristol BS10 5NB, United Kingdom

Funding Sources: Parkinson's UK
215 Vauxhall Bridge Road
London SW1V 1EJ, United Kingdom

The Cure Parkinson's Trust
The Vestry
1, St Clement's Court
London EC4N 7HB, United Kingdom

Drug Manufacturer: MedGenesis Therapeutix, Inc.
730-730 View Street
Victoria, BC V8W 3Y7, Canada

Protocol authorised by:

Dr Alan Whone **18.06.2013**

TABLE OF CONTENTS

STUDY TITLE ... 1
1 SYNOPSIS ... 7
2 ABBREVIATIONS ... 10
3 BACKGROUND AND RATIONALE ... 12
4 OBJECTIVES ... 13
4.1 Primary Objective ... 13
4.2 Secondary Objectives ... 13
4.3 Other Objectives ... 13
5 TRIAL DESIGN ... 14
5.1 Summary of Trial Design ... 14
5.1.1 Overview ... 14
5.1.2 Post Study Access to Treatment ... 16
5.2 Study Endpoints ... 17
5.2.1 Primary Endpoint ... 17
5.2.2 Secondary Endpoints ... 17
5.2.3 Supplementary Efficacy Endpoints ... 18
5.2.4 Imaging Endpoints ... 18
5.2.5 Safety Endpoints ... 18
5.3 Trial Participants ... 19
5.3.1 Overall Description of Trial Participants ... 19
5.3.2 Inclusion Criteria ... 19
5.3.3 Exclusion Criteria ... 19
5.4 Expenses and Benefits ... 20
5.5 Study Procedures ... 20
5.5.1 Informed Consent ... 20
5.5.2 Screening and Eligibility Assessment ... 21
5.5.3 Preparation of Study Drug ... 21
5.6 Schedule of Treatments and Assessments ... 21
5.6.1 Treatments ... 22
5.6.2 Interim Assessments ... 23
5.6.3 Laboratory and Other Assessments ... 24
5.6.4 Week e40 or Early Discontinuation ... 25

5.7 Outcomes Measures 27
5.7.1 Efficacy Outcome Measures 27
5.7.2 Safety Outcome Measures 30
5.8 Substudies 33
5.9 Definition of End of Trial 33
5.10 Discontinuation/ Withdrawal of Subjects from Study Treatment 33
5.11 Source Data 34
6 TREATMENT OF TRIAL PARTICIPANTS 35
6.1 Description of Study Medication 35
6.2 Description of Convection-Enhanced Delivery System 35
6.2.1 Implantation of the Drug Delivery System 36
6.2.2 Drug Infusions 36
6.3 Treatment 37
6.4 Compliance with Study Treatment 37
6.5 Accountability of the Study Treatment 38
6.6 Concomitant Medication 38
7 SAFETY REPORTING 40
7.1 Definitions 40
7.2 Adverse Event (AE) 40
7.3 Adverse Reaction (AR) 40
7.4 Serious Adverse Event (SAE) 40
7.5 Serious Adverse Reaction (SAR) 41
7.6 Suspected Unexpected Serious Adverse Reaction (SUSAR) 41
7.7 Causality and Expectedness 41
7.8 Procedures for Recording Adverse Events 42
7.9 Reporting Procedures for Serious Adverse Events 43
7.10 SUSAR Reporting 43
7.11 Annual Safety Reports 43
8 STATISTICS 44
8.1 Description of Statistical Methods 44
8.2 Number of Participants 44
8.3 Level of Statistical Significance 44
8.4 Criteria for the Termination of the Trial 44

8.5 Procedure for Accounting for Missing, Unused, and Spurious Data 45
8.6 Procedures for Reporting any Deviation(s) from the Original Statistical Plan 45
8.7 Inclusion in Analysis 45
9 DIRECT ACCESS TO SOURCE DATA/DOCUMENTS 46
10 QUALITY CONTROL AND QUALITY ASSURANCE PROCEDURES 47
11 SERIOUS BREACHES 48
12 ETHICS 49
12.1 Declaration of Helsinki 49
12.2 ICH Guidelines for Good Clinical Practice 49
12.3 Approvals 49
12.4 Participant Confidentiality 49
13 DATA HANDLING AND RECORD KEEPING 50
14 FINANCE AND INSURANCE 51
14.1 Compensation for Harm 51
15 PUBLICATION POLICY 52
16 REFERENCES 53
17 APPENDIX A: STUDY SCHEDULE OF EVENTS 54
18 APPENDIX B: SAE REPORTING FLOW CHART ....
19 APPENDIX C: MONTREAL COGNITIVE ASSESSMENT (MOCA) ....
20 APPENDIX D: BECK DEPRESSION INVENTORY (BDI) ....
21 APPENDIX E: QUESTIONNAIRE FOR IMPULSIVE-COMPULSIVE DISORDERS IN PARKINSON'S DISEASE (QUIP) ....
22 APPENDIX F: UNIFIED PARKINSON'S DISEASE RATING SCALE (UPDRS) ....
23 APPENDIX G: PD FLUCTUATION DIARY ....
24 APPENDIX H: NON-MOTOR SYMPTOM ASSESSMENT SCALE FOR PD (NMSS) .
25 APPENDIX I: PARKINSON'S DISEASE QUESTIONNAIRE-39 (PDQ-39) ....
26 APPENDIX J: EUROQOL 5-DIMENSIONAL SCALE (EQ-5D) ....

List of Tables

Table 1 Schedule of Events 55

List of Figures

Figure 1 Study Schema 15

AMENDMENT HISTORY

Not applicable.

1 SYNOPSIS

Study Title	An Extension Study to Assess the Safety and Efficacy of Intermittent Bilateral Intraputamenal Glial Cell Line-Derived Neurotrophic Factor (GDNF) Infusions Administered via Convection Enhanced Delivery (CED) in Subjects with Parkinson's Disease
Internal Ref. No.	2797
Clinical Phase	Phase II
Trial Design	An open-label extension study for subjects who complete Study 2553.
Trial Participants	Subjects with bilateral idiopathic Parkinson's disease (PD), according to the United Kingdom (UK) Brain Bank Criteria, who complete a double-blind study of GDNF administered via CED (Study 2553)
Planned Sample Size	Up to 42 subjects
Treatment and Follow-up Duration	9 months
Planned Trial Period	The planned study period, including recruitment, is 27 months.
Primary Objective	To compare the effects of intermittent bilateral intraputamenal GDNF infusions on OFF-state motor function after 18 months of treatment with the effects after 9 months of treatment in subjects who completed in Study 2553.
Secondary and Other Objectives	• To compare the effects of intermittent bilateral intraputamenal GDNF infusions on ON-state motor function, motor complications, and ON- and OFF-state activities of daily living (ADL) after 18 months of treatment with the effects after 9 months of treatment in subjects who completed Study 2553.<br>• To assess the safety of intermittent bilateral intraputamenal GDNF infusions at 18 months in subjects who received GDNF or placebo for 9 months in Study 2553.<br>• To explore the effects of intermittent bilateral intraputamenal GDNF infusions on other motor and non-motor functions, quality of life assessments, and imaging endpoints at 18 months in subjects who completed Study 2553.<br>• To compare the results for various motor outcomes between the subjects who started GDNF early (i.e. were randomized to GDNF in Study 2553) and those who started GDNF late (i.e. were randomized to placebo in Study 2553).
Primary Efficacy Endpoint	Percentage change from baseline to the end of treatment in the practically defined OFF-state Unified Parkinson's Disease Rating Scale (UPDRS) motor score (part III).
Secondary and Other Efficacy Endpoints	Secondary efficacy endpoints will include the following:<br>• Percentage change from baseline to the end of treatment in UPDRS motor score (part III) in the ON-state (following a levodopa challenge).<br>• Percentage change from baseline to the end of treatment in UPDRS activities of daily living (ADL) (part II).<br>• Change from baseline to the end of treatment in PD diary ratings; i.e., total OFF-time per day, total good quality ON-time (ON without dyskinesias or ON with non-troublesome dyskinesias) and ON-time with troublesome

	dyskinesias.<br>• Change from baseline to the end of treatment in PD non-motor assessments of cognition, executive function, mood, other non-motor symptoms, smell, appetite, medication equivalents and quality of life<br>Analysis of the primary and secondary endpoints will be repeated for the overall ITT population including subjects randomised in the Pilot Stage.
Safety Endpoints	Safety endpoints include the following:<br>• Incidence of device-related adverse events (AEs) during the study period.<br>• Incidence of treatment-emergent AEs (related and unrelated) during the study period.<br>• Time with troublesome dyskinesias (from subject diaries).<br>• Clinical assessment of safety, including dyskinesias and falls, mood and impulsivity at each infusion visit.<br>• The Questionnaire for Impulsive-Compulsive Disorders in Parkinson's Disease (QUIP) every 8 weeks.<br>• Changes in the Montreal Cognitive Assessment (MoCA) and Mattis Dementia Rating Scale (MDRS) at the end of the study.<br>• Adverse changes in magnetic resonance imaging (MRI) findings.<br>• Anti-GDNF antibody samples taken at intervals during the study will be assessed at the end of the study.<br>• Routine laboratory blood tests (haematology, serum chemistry) and urinalysis performed at baseline and at intervals during the trial.<br>• Full-brain MRI.
Imaging Endpoints	Volume of distribution of infusate will be determined by T2-weighted and fluid-attenuated inversion recovery (FLAIR) 3T MRI. Scans are to be completed within 2 hours post-infusion and will be obtained after the last study treatment in all subjects. Additional scans may be performed at the discretion of the PI.
Statistical Analysis	Descriptive statistics will be presented for all endpoints by treatment group supporting the primary and secondary analyses. Detailed methodology will be provided in the statistical analysis plan (SAP). No inferential analyses are planned for this open-label extension study.<br>The efficacy analyses will be conducted after all Primary Study Stage subjects have reached the 18-month endpoint and will include only subjects who had been randomised to the Primary Study Stage of Study 2553. Supplementary analyses will include subjects treated in the Pilot Extension Stage.<br>Safety data will be presented descriptively, by treatment group, with standard Medical Dictionary for Regulatory Activities (MedDRA) coded AE and SAE frequency and incidence tables as well as shift tables for clinical laboratory parameters.<br>The Safety Population will comprise all subjects treated on study with subjects grouped according to treatment actually received. All safety analyses will be performed on the Safety Population.
Investigational Medicinal Products	GDNF in artificial cerebrospinal fluid (aCSF) at a concentration of 0.2 µg/µL (active treatment)
Form	Intracerebral infusion by CED.
Dose	600 µL (containing 240 µg of GDNF) per putamen every 4 weeks for 9 months.

Route	Intraputamenal infusion via a CED infusion system with 2 indwelling catheters per putamen.

2 ABBREVIATIONS

aCSF	Artificial cerebrospinal fluid
ADL	Activities of daily living
AE	Adverse event
ALT	Alanine transaminase
AR	Adverse reaction
BDI	Beck Depression Inventory
BP	Blood pressure
CED	Convection enhanced delivery
CSF	Cerebrospinal fluid
DMC	Data Monitoring Committee
ECG	Electrocardiogram
EDC	Electronic data capture (system)
eGFR	Estimated glomerular filtration rate
EQ-5D	EuroQOL 5-dimensional scale
FLAIR	Fluid-attenuated inversion recovery
GCP	Good Clinical Practice
GDNF	Glial cell line-derived neurotrophic factor
HR	Heart rate
ICH	International Conference of Harmonisation
MCH	Mean cellular haemoglobin
MCHC	Mean cellular haemoglobin concentration
MCV	Mean cellular volume
MDRS	Mattis Dementia Rating Scale
MedDRA	Medical Dictionary for Regulatory Activities
MHRA	Medicines and Healthcare Products Regulatory Agency
MoCA	Montreal Cognitive Assessment
MRI	Magnetic resonance imaging
NBT	North Bristol NHS Trust
NBT R&I	North Bristol NHS Trust Research and Innovation
NMSS	Non-Motor Symptom Assessment Scale for PD
PD	Parkinson's Disease
PDQ-39	Parkinson's Disease Questionnaire-39
PET	Positron emission tomography
PI	Principal Investigator
QTc	Corrected QT (interval)
QUIP	Questionnaire for Impulsive-Compulsive Disorders in Parkinson's Disease

RBC	Red blood cell (count)
REC	Research Ethics Committee
RR	Respiration rate
SAE	Serious adverse event
SAP	Statistical analysis plan
SAR	Serious adverse reaction
SMPC	Summary of Medicinal Product Characteristics
SUSAR	Suspected unexpected serious adverse reactions
TMF	Trial Master File
UK	United Kingdom
UPDRS	Unified Parkinson's Disease Rating Scale
UPSIT	University of Pennsylvania Smell Identification Test
WBC	White blood cell (count)
WHO	World Health Organization

3 BACKGROUND AND RATIONALE

Glial cell line-derived neurotrophic factor (GDNF) is a neurotrophic factor with potent effects on diverse nerve cell lines including dopaminergic, serotonergic, noradrenergic, and cholinergic neurones [1, 2]. Intraputamenal GDNF, administered via convection-enhanced delivery (CED) [3, 4], is being investigated as a treatment for Parkinson's disease (PD).

Study 2553 is a placebo-controlled, randomised, double-blind trial to assess the safety and efficacy of intermittent bilateral intraputamenal GDNF infusions administered via CED in subjects with PD. In that study, subjects receive GDNF or placebo q4 weekly for up to 9 months. Please refer to the Study 2553 protocol for further details regarding that study and to the GDNF Investigator's Brochure for background and safety information on GDNF.

This study is an open-label extension study for subjects who complete Study 2553. All enrolling subjects will be receiving active treatment for up to 9 months, regardless of their treatment assignment in Study 2553. Hence, the study will provide access to continued treatment for those subjects who received active treatment during Study 2553 and provide access to active treatment for those subjects who received placebo in Study 2553. However, individual treatment codes in Study 2553 will not be disclosed to subjects and study personnel until the end of the extension study.

The study is designed to assess the effects on efficacy and safety of extended (up to 18 months total) intermittent intraputamenal infusion of GDNF, using similar endpoints as Study 2553. In addition, the study is expected to provide initial insight into the question of whether the effect on motor outcome at 18 months is improved with a longer duration of treatment (9 vs. 18 months).

4 OBJECTIVES

4.1 Primary Objective

To compare the effects of intermittent bilateral intraputamenal GDNF infusions on OFF-state motor function after 18 months of study treatment with the effects after 9 months of treatment in subjects who completed Study 2553.

4.2 Secondary Objectives

- To compare the effects of intermittent bilateral intraputamenal GDNF infusions on ON-state motor function, motor complications, and ON- and OFF-state activities of daily living (ADL) after 18 months of treatment with the effects after 9 months of treatment in subjects who completed Study 2553.
- To assess the safety of intermittent bilateral intraputamenal GDNF infusions at 18 months in subjects who received GDNF or placebo for 9 months in Study 2553.

4.3 Other Objectives

- To explore the effects of intermittent bilateral intraputamenal GDNF infusions on other motor and non-motor functions, quality of life assessments and imaging endpoints at 18 months in subjects who completed Study 2553.
- To compare the results for various motor outcomes between the subjects who started GDNF early (i.e. were randomized to GDNF in Study 2553) and those who started GDNF late (i.e. were randomized to placebo in Study 2553).

5 TRIAL DESIGN

5.1 Summary of Trial Design

5.1.1 Overview

This is a phase II, single-centre, open-label trial, in subjects with idiopathic PD who have completed Study 2553 of intermittent bilateral posterior putamen GDNF infusions administered via CED. The study is planned to enrol a total of up to 42 subjects.

Following the final study visit at Week 40 in Study 2553, study completers will return within one week to receive their first infusion of open-label GDNF. Treatment will be continued at 4-week intervals for 9 months (40 weeks; 10 infusions total). Hence, at 18 months, subjects receiving GDNF in Study 2553 will have been treated with GDNF for a total of 18 months, while those receiving placebo in Study 2553 will have been treated with GDNF for a total of 9 months.

Figure 1presents the study schema.

The planned study period, including recruitment, is 27 months.

Although the primary statistical assessment of Study 2553 will have been performed before the extension study is complete, to reduce any potential for bias in this study, individual treatment codes from the parent study will not be disclosed until after the last subject has completed the final study visit, unless required for specific safety reasons.

As reflected in protocol amendments no. 3 and 4, the delivery technique and algorithm employed in the Primary Stage of Study 2553 was appreciably different from the Pilot Stage. Therefore, the primary analysis population of both studies only includes subjects randomised during the Primary Study Stage. However, secondary efficacy analyses will include subjects randomised in the Pilot Stage as well. Safety will be analysed for the Pilot and Primary Study Stages separately and for all subjects and both stages combined.

Figure 1 Study Schema

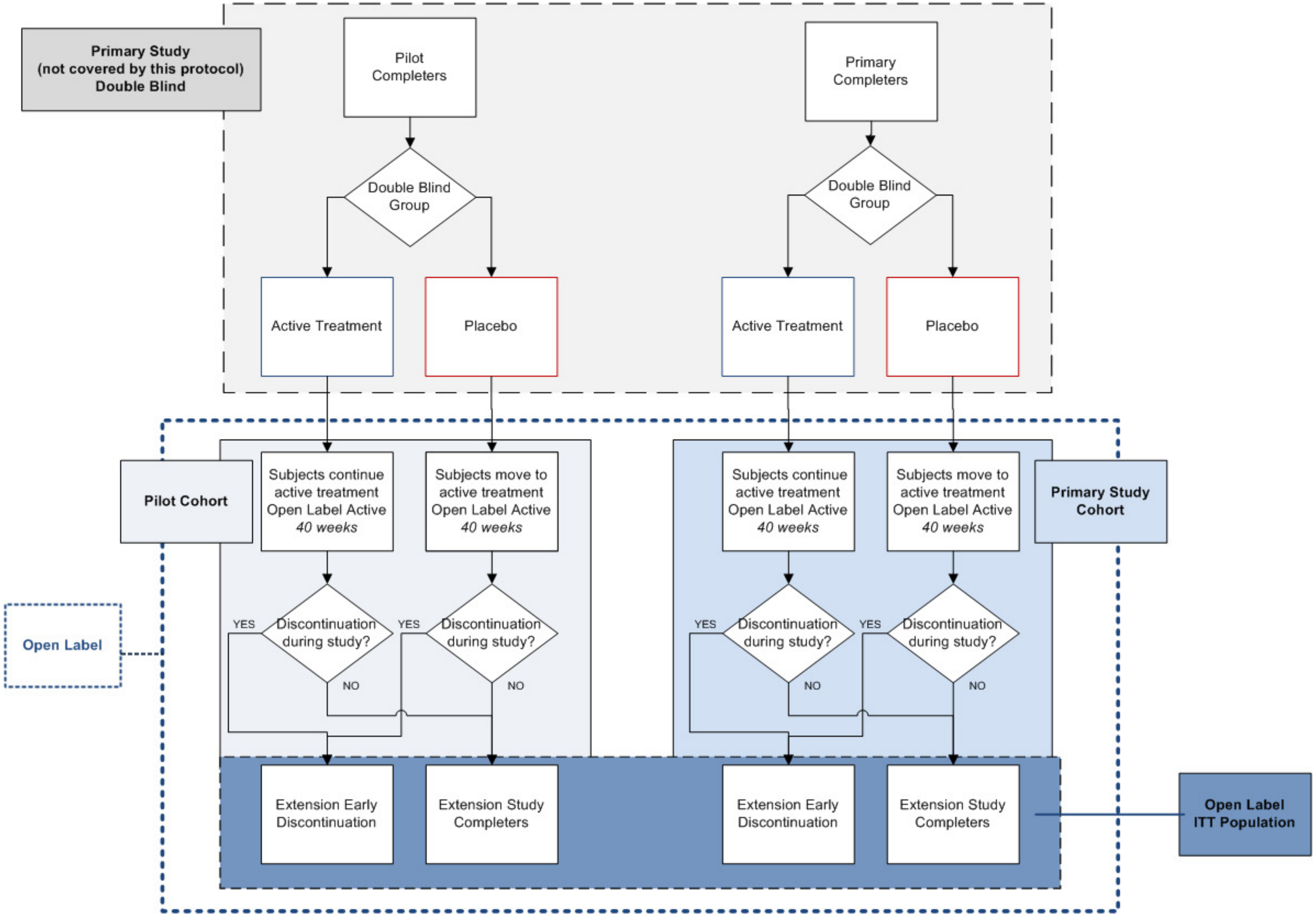

5.1.2 Post Study Access to Treatment

What happens at study end? The below information is taken directly from the Patient Information Sheet but is given here in order that the information provided to study subjects is also directly included in the protocol:

No one yet knows what will happen to the supply of GDNF or continuance of infusions once the extension study ends. Numerous factors will need to be in place for GDNF infusions to continue and none of these can be guaranteed. These include that the company that holds the license for GDNF will continue to produce the protein, that the regulatory bodies governing administration of drug therapies in the UK will allow us to continue administering, and that funding and capacity to continue either purchasing and/or administering GDNF is available.

The hope is that if the double-blind study shows positive benefits across the population of patients assessed, without side effects that prevent continuation, infusions may continue post the end of the extension study. This will enable us to continue to collect benefit and safety data. However, that this occurs can in no way be guaranteed. As discussed with participants prior to enrolling in the double-blind study GDNF infusions may very well stop post either the double-blind study or the extension study, even if participants have received great benefit. In terms of GDNF being developed as a routine therapy, the next step following the extension study (but only if the double-blind study is successful) will be a phase III trial, where presumably more than 100 patients will be enrolled and treated at multiple centres around the world. Only after such a multi-centre trial which may take several years, followed by a period of further regulatory approvals, is there the potential for GDNF therapy to be routinely available.

Hence receiving GDNF may well stop at the end of this extension study even if participants individually have received benefit from the infusions.

At the end of this trial, if it is found that there is no statistical level benefit to the participants as a whole from receiving GDNF it is unlikely that this therapy will continue to be available to subjects even if they as individuals feel or are better for it.

If this trial is statistically positive we still cannot guarantee that GDNF will be available, but if GDNF becomes available through normal commissioning processes,

the subjects' clinicians will review how appropriate subjects are to receive this treatment.

If GDNF is stopped or not available, the subjects' doctors will ensure that adequate consideration is given to the protection of subjects' interests and ongoing medical needs. This point will be discussed with subjects further prior to obtaining their consent to participate.

If during this trial GDNF becomes no longer available, it is anticipated that the port system behind the ear will be removed and closed. However, the remaining tubing inside the brain etc. may be left in place if this is deemed to be a safer or better option post discussion with the medical team.

5.2 Study Endpoints

The analysis of the primary and secondary endpoints will be restricted to the up to 36 subjects randomised during the Primary Stage in Study 2553. Subjects treated during the Pilot Stage in Study 2553 will, however, be included in the analysis of safety and supplementary efficacy analyses.

5.2.1 Primary Endpoint

The primary endpoint of the study is the percentage change from baseline to the end of treatment in the practically defined OFF-state UPDRS motor score (part III).

5.2.2 Secondary Endpoints

- Percentage change from baseline to the end of treatment in UPDRS motor score (part III) in the ON-state (following a levodopa challenge)
- Percentage change from baseline to the end of treatment in UPDRS ADL (part II) .
- Change from baseline to the end of treatment in PD diary ratings; i.e., total OFF-time per day, total good quality ON-time (ON without dyskinesias or ON with non-troublesome dyskinesias) and ON-time with troublesome dyskinesias.

5.2.3 Supplementary Efficacy Endpoints

Change from baseline to the end of treatment in PD non motor assessments of cognition, executive function, mood, other non-motor symptoms, smell, appetite, medication equivalents, and quality of life.

Analysis of the primary and secondary endpoints will be repeated for the overall ITT population including subjects randomised in the Pilot Stage.

5.2.4 Imaging Endpoints

Volume of distribution of infusate will be determined by T2-weighted and fluid-attenuated inversion recovery (FLAIR) 3T MRI. Scans are to be completed within 2 hours post-infusion and will be obtained after the last study treatment in all subjects. Additional scans may be performed at the discretion of the PI.

5.2.5 Safety Endpoints

- Incidence of device-related adverse events (AEs) during the study period.
- Incidence of treatment-emergent AEs (related and unrelated) during the study period.
- Time with troublesome dyskinesias (from subject diaries).
- Clinical assessment of safety, including dyskinesias and falls, mood and impulsivity at each infusion visit.
- The Questionnaire for Impulsive-Compulsive Disorders in Parkinson's Disease (QUIP) every 8 weeks.
- Changes in the Montreal Cognitive Assessment (MoCA) and Mattis Dementia Rating Scale (MDRS) at the end of the study.
- Adverse changes in magnetic resonance imaging (MRI) findings.
- Anti-GDNF antibody samples taken at intervals during the study will be assessed at the end of the study.
- Routine laboratory blood tests (haematology, serum chemistry) and urinalysis performed at intervals during the trial.
- Full-brain MRI.

5.3 Trial Participants

5.3.1 Overall Description of Trial Participants

Subjects with idiopathic PD with motor fluctuations who complete Study 2553.

5.3.2 Inclusion Criteria

In order to qualify for entry into the study, subjects MUST meet all of the following criteria:

1. Enrolled and completed treatment in the Pilot or Primary Study Stages of Study 2553.
2. Females of childbearing potential must have a negative pregnancy test at study entry and be willing to use an approved (by the PI or designee) form of contraception until the end of the study.
3. Males with female partners of childbearing potential must be willing to use condoms for contraception until the end of the study.
4. Provision of informed consent.

5.3.3 Exclusion Criteria

Subjects who meet any of the following criteria will NOT be eligible for inclusion in the study:

1. Discontinued treatment early in Study 2553.
2. Had any significant (in the opinion of the PI or designee) protocol deviation in Study 2553; this includes receipt of any disallowed anti-parkinsonian treatment or any investigational treatment.
3. Presence of clinically significant (in the opinion of the PI) depression.
4. MoCA score < 24 at the final assessment in Study 2553.
5. Any new medical condition which might impair outcome measure assessments or safety measures including ability to undergo MRI scanning.

5.4 Expenses and Benefits

Reasonable travel expenses for any visits additional to normal care will be reimbursed on production of receipts or a mileage allowance provided as appropriate.

5.5 Study Procedures

Subjects may enroll in this study immediately upon completion of Study 2553 provided they meet the eligibility criteria. Procedures and assessments performed at Week 40 of the parent study will be used as screening assessments for this study. The count of study weeks will be reset and numbers will be prefixed with an "e" (e.g., "Week e0") to differentiate them from the numbering in Study 2553.

The first infusion in the extension study will be performed at the Week e0 visit which is to occur within one week of the Week 40 assessment in Study 2553 so as to keep the interval between the last treatment in the parent study and the first treatment in the extension study to a maximum of 5 weeks. Beginning at Week e0, GDNF infusions will be administered every 4 weeks for 9 months; the total dose of GDNF administered at any given infusion remains unchanged from Study 2553 at 240 µg (600 µL of 0.20 µg/µL GDNF) per putamen.

Volumetric catheter performance will be determined by means of T2-weighted and FLAIR 3T MRI scans within 2 hours after the fifth and the last treatment prior to the visit e 40 . Key clinical outcome measures will be performed at 8-week intervals throughout the study. Additional clinical outcome measures will be assessed at visit e 40.

5.5.1 Informed Consent

The subject must personally sign and date the latest approved version of the informed consent form before any study specific procedures and assessments are performed (i.e., before Week e0 procedures for this study are performed). Week 40 procedures for Study 2553, which may be used as screening for this study, may be performed before consent for the extension study is obtained.

Written and verbal versions of the participant information and informed consent will be presented to the potential participants detailing no less than: the experimental nature of the study; the implications and constraints of the protocol; the known side effects and

any risks involved in taking part. It will be clearly stated that any participant is free to withdraw from the study at any time for any reason without prejudice to future care and with no obligation to give the reason for withdrawal.

The above information will be provided to the potential participants at the 36-Week visit or earlier in the parent study, so as to allow ample time to consider the information and provide the opportunity to question the PI or designee, their general practitioner, or other independent parties to decide whether they will participate in the study. Written informed consent will then be obtained by means of participant dated signature and dated signature of the person who presented and obtained the informed consent. The person who obtained the consent must be suitably qualified and experienced and have been authorised to do so by the PI. A copy of the signed informed consent will be given to the participant. The original signed form will be retained at the study site, and a copy will be filed in the medical notes.

5.5.2 Screening and Eligibility Assessment

Screening and eligibility assessments for this study will be the assessments performed as part of the Week 40 assessments for Study 2553; aside from informed consent, no additional screening procedures are required for this study.

5.5.3 Preparation of Study Drug

All subjects will receive active GDNF in this extension study.

Study drug will be prepared by a pharmacist at the study site. Ready to use preparations of GDNF will be provided to the PI or designee. Drug preparation guidelines are provided in a separate pharmacy manual.

5.6 Schedule of Treatments and Assessments

Upon receipt of study drug as described above, the PI or designee will initiate a 40-week (approximately 9 months) course of q4 weekly intraputamenal administration of GDNF via CED. The full course will comprise 11 scheduled visits (Week e0 and 10 q4 weekly visits through Week e40). Except for the final visit where no infusion will be given, at each visit GDNF will be administered, followed by clinical assessment of safety, including dyskinesias and falls, mood and impulsivity. AEs, concomitant medications and vital signs will be recorded at all visits. PD assessments and QUIP

will be performed every 8 weeks, and full assessments of safety and PD variables will be performed at the final assessment (Week e40).

5.6.1 Treatments

All subjects will receive open-label active treatment (GDNF) every 4 weeks, but subjects, and site personnel will remain blinded to the treatment codes of individual subjects during Study 2553 until the completion of the study unless unblinding is required for specific safety reasons.

Subjects will return to the clinic for treatments within one week after the completion of the Week 40 visit for Study 2553 (Week e0 of the extension study) and every 4 weeks (± 3 days) thereafter at Weeks e4, e8, e12, e16, e20, e24, e28, e32 and e36. Before each PD outcome measure assessment (Weeks e8, e16, e24 and e32) that is to be taken (detailed in Section 5.6.2), subjects will withhold all PD medications for assessment in the OFF state. For all other treatments, subjects will take their typical medications on the study treatment day and will report to the clinic irrespective of their ON/OFF state.

At every treatment, the following procedures and assessments will be performed before treatment:

- Vital signs: Seated systolic and diastolic blood pressure (BP), heart rate (HR), respiration rate (RR) and temperature will be assessed after the subject has been sitting quietly for at least 5 minutes. Standing systolic and diastolic BP and HR will also be assessed after the subject has been standing for 3 minutes.
- Any changes to concomitant medications from the previous visit will be recorded. All over-the-counter or prescription medications, vitamins, and/or herbal supplements will be recorded in the electronic data capture (EDC) system with their indications.
- Any AEs observed or reported since the previous visit will be recorded.

After completion of the pretreatment procedures and assessments, the PI or designee will administer the drug over a period of approximately 90 to 150 minutes using pre-programmed micro-infusion pumps.

Subjects will be carefully observed for safety during and after study drug administration. During administration, seated vital signs (systolic and diastolic BP, HR

and RR) will be collected every 15 (Week e0) to 30 (Weeks e4 to e36) minutes, and both seated and standing systolic and diastolic BP and HR will be assessed approximately 30 minutes after the end of the infusion. A brief neurological screen (Glasgow Coma Scale) will also be done before infusion, 30 minutes into the infusion, and after completion of the infusion.

Subjects will remain at the facility for safety observation for at least 2 to 4 hours after completing study drug administration at Week e0, and 1 to 2 hours after completing study drug administration at all subsequent time points.

The last study treatment will be administered at Week e36.

At the treatment before each Interim Assessment and at the last Treatment (i.e., at Weeks e4, e12, e20, e28 and e36), subjects will be issued PD fluctuation diaries (Section 5.7.1.4) to complete at home before the next assessment. The subject will be instructed to complete diaries for 3 consecutive predetermined days in the week prior to the next study treatment. The day immediately prior to the study treatment is NOT to be used for diary recording.

5.6.2 Interim Assessments

Subjects will undergo extended safety and efficacy assessments every 8 weeks at Weeks e8, e16, e24 and e32.

For these assessments, subjects will withhold all PD medications as follows and report to the clinic in the OFF state:

- No PD medications will be taken after 6:00 PM on the night before the assessments and
- No long-acting PD medications will be taken on the day before the assessments.

Subjects will refrain from eating any high-protein foods on the morning of the assessments.

Interim Assessment visits will also be Treatment visits, and procedures and assessments will include those listed in Section 5.6.1 for Treatments. However, the following assessments will also be performed pretreatment:

At the beginning of the assessment visit, all OFF period measures are to be completed first:

- UPDRS part II (ADL) and part III (motor) in OFF state (Section 5.7.1.1)
- Timed walking test (Section 5.7.1.2)
- Timed tapping test (Section 5.7.1.3)

A levodopa challenge will then be performed (refer to study-specific Standard Operating Procedures) and when the subject goes ON the following post-challenge tests will be performed:

- Full UPDRS (including parts I, II, III and IV; Section 5.7.1.1)
- Timed walking test (Section 5.7.1.2)
- Timed tapping test (Section 5.7.1.3)
- MDRS (Section 5.7.2.2)/MoCA (Section 5.7.2.1)
- Other brief measures of cognitive and executive function (Section 5.7.2.4)
- QUIP (Section 5.7.2.6)

Additional study procedures which can be performed irrespective of ON/OFF state include:

- Collection of subject PD fluctuation diaries (Section 5.7.1.4)
- Weight and height

5.6.3 Laboratory and Other Assessments

The following laboratory procedures will be performed at Weeks e4, e16 and e28:

- Clinical laboratory tests, including haematology (haematocrit, haemoglobin, mean cellular haemoglobin (MCH), mean cellular haemoglobin concentration (MCHC), mean cellular volume (MCV), platelet count, red blood cell (RBC) count, white blood cell (WBC) count and WBC differential [basophils, eosinophils, lymphocytes, monocytes and neutrophils]), serum chemistry (albumin, alkaline phosphatase, alanine transaminase (ALT), creatinine, estimated glomerular filtration rate (eGFR), glucose, potassium, sodium, total bilirubin and urea) and urinalysis (colour, appearance, pH, glucose, ketones and

nitrite; microscopy only required if indicated to follow up abnormal findings). A serum pregnancy test will be performed for all women of childbearing potential.

- Blood samples for anti-GDNF antibodies and plasma GDNF levels. The samples will be stored at -70°C until central analysis after all subjects have completed the study.

Subjects will undergo Non-Motor Symptom Assessment Scale for PD (NMSS) (Section 5.7.1.5) at Weeks e12 and e24.

T2-weighted and FLAIR 3T MRI are to be completed within 2 hours of completion of the infusions at Weeks e36. Additional scans may be performed at the discretion of the PI.

5.6.4 Week e40 or Early Discontinuation

Week e40 will mark the completion of the extension study. A full safety and efficacy assessment will be performed, and the same assessments should be performed, if possible, for any subjects who discontinue the study before Week e40.

For this assessment, subjects will withhold all PD medications as follows:

- No PD medications will be taken after 6:00 PM on the night before the assessments and
- No long-acting PD medications will be taken on the day before the assessments.

Subjects will refrain from eating any high-protein foods on the morning of the assessments.

At the beginning of the assessment, all OFF period measures are to be completed first:

- UPDRS part II (ADL) and part III (motor) in OFF state (Section 5.7.1.1)
- Timed walking test (Section 5.7.1.2)
- Timed tapping test (Section 5.7.1.3)

A levodopa challenge will then be performed (refer to study-specific Standard Operating Procedures) and when the subject goes ON the following post-challenge tests will be performed:

- Full UPDRS (including parts I, II, III and IV; Section 5.7.1.1)
- Timed walking (Section 5.7.1.2)
- Timed tapping test (Section 5.7.1.3)
- MDRS (Section 5.7.2.2)/MoCA (Section 5.7.2.1)
- Stroop (Section 5.7.2.3)
- Other brief measures of cognitive and executive function (Section 5.7.2.4)
- BDI (Section 5.7.2.4)
- PD NMSS (Section 5.7.1.5)
- Parkinson's Disease Questionnaire-39 (PDQ-39; Section 5.7.1.6)
- EuroQOL 5-dimensional scale (EQ-5D; Section 5.7.1.6)
- QUIP (Section 5.7.2.6)

The following procedures may be completed irrespective of the subject's ON/OFF status:

- University of Pennsylvania Smell Identification Test (UPSIT; Section 5.7.2.7)
- Collection of subject PD fluctuation diaries (Section 5.7.1.4)
- Weight and height
- Vital signs: Seated systolic and diastolic BP, HR, RR and temperature will be assessed after the subject has been sitting quietly for at least 5 minutes. Standing systolic and diastolic BP and HR will also be assessed after the subject has been standing for 3 minutes.
- Brief physical examination, targeted, at the investigators discretion, to identify any notable changes from baseline.
- Standard 12-lead electrocardiogram (ECG). At least the following ECG parameters will be recorded: HR, PR, QT, QRS and corrected QT (QTc) intervals. The report will be signed by the PI or designee and it will be recorded in the EDC whether it is normal, abnormal but not clinically significant, or abnormal AND clinically significant (together with details of the abnormality).

- Clinical laboratory tests, including haematology (haematocrit, haemoglobin, MCH, MCHC, MCV, platelet count, RBC, WBC count and WBC differential [basophils, eosinophils, lymphocytes, monocytes and neutrophils]), serum chemistry (albumin, alkaline phosphatase, ALT, creatinine, eGFR, glucose, potassium, sodium, total bilirubin and urea) and urinalysis (colour, appearance, pH, glucose, ketones and nitrite; microscopy only required if indicated to follow up abnormal findings). A serum pregnancy test will be performed for all women of childbearing potential.
- Blood samples for anti-GDNF antibodies and plasma GDNF levels. The samples will be stored at -70°C until central analysis after all subjects have completed the study.
- Any changes to concomitant medications from the previous visit will be recorded. All over-the-counter or prescription medications, vitamins, and/or herbal supplements will be recorded in the EDC with their indications.
- Any AEs observed or reported since the previous visit will be recorded.

5.7 Outcomes Measures

5.7.1 Efficacy Outcome Measures

5.7.1.1 Unified Parkinson's Disease Rating Scale

The UPDRS (Appendix F) was developed to as an outcome measure for rating PD in clinical trials. It has 4 parts which predominantly measure the motor signs and symptoms of PD: Mentation, Behaviour and Mood (part I), ADLs (part II), Motor Examination (part III), and Complications of Therapy (part IV). Except for the motor examination which is performed at the assessment, all sections of the UPDRS rate the subject based on his/her state in the week preceding the assessment. Higher scores represent worse functioning.

In this trial all UPDRS ratings will be performed by a trained rater who is blinded to all other aspects of the subject's condition. The motor UPDRS will be performed with the subject in a practically defined OFF state (at least 12 hours since last PD medication) and in the ON state after levodopa challenge.

The primary efficacy outcome for this study will be the percentage change in motor UPDRS in the practically defined OFF state between baseline (i.e., before beginning treatment in Study 2553) and Week e40. Other UPDRS endpoints include UPDRS part II in both the ON and OFF states, UPDRS part III in the ON state after levodopa challenge and total UPDRS scores. ON and OFF UPDRS ratings will be done at Weeks e8, e16, e24, e32 and e40.

5.7.1.2 Timed Walking test

During the timed walking test, the subject will walk as fast as possible 7 metres back and forth including turning. The time to perform this test is recorded. The subject will complete this test twice during the practically defined OFF period and twice in the ON state after levodopa challenge. The mean ON and mean OFF rating scores will then be calculated and entered into the eCRF. The ratings will be performed at Weeks e8, e16, e24, e32 and e40.

5.7.1.3 Timed Tapping Test

During the timed tapping test, the subject is instructed to alternate tapping the index finger between 2 points spaced 30 cm apart. Each hand is rated twice and the mean number of taps completed in 20 seconds on each side is to be recorded. This test is to be performed in the practically defined OFF state and in the ON state after levodopa challenge at Weeks e8, e16, e24, e32 and e40.

5.7.1.4 Subject Diaries

Motor fluctuations in this study will be quantified using subject completed diaries (Appendix G). For 3 days prior to the relevant study assessments (not including the day immediately prior to the assessment, as the subject will be asked to withhold PD medication on that day), subjects are to record their state for every half hour time period. Categories for rating include: ON with no dyskinesias, ON with non-troublesome dyskinesias, ON with troublesome dyskinesias, OFF or asleep. Caregivers may assist with the physical completion of the diary, however, the decision regarding the subject's state is to be made by the subject alone.

During the parent study, subjects will have been trained on the completion of the diary and will have demonstrated their ability to accurately determine their state by

comparing their own assessments to those of a qualified staff member over a 2-4 hour period.

Duration of good ON time (ON without dyskinesias and ON with non-troublesome dyskinesias) and OFF time will be secondary efficacy variables in this study. Duration of ON with troublesome dyskinesias will be a safety outcome for this study.

Diaries will be collected at Weeks e8, e16, e24, e32 and e40. Diaries are to be reviewed by the coordinator each time they are returned, and retraining should be offered if errors in completion are noted. Diaries are to be dispensed at the visit prior to their collection.

5.7.1.5 Non-Motor Symptom Scale

The NMSS (Appendix H) is an interview based scale developed to rate non-motor symptoms commonly occurring in PD. The 30 item scale rates symptoms which occurred in the preceding month in 9 domains – cardiovascular function including falls, sleep/fatigue, mood/cognition, perceptual problems/hallucinations, attention/memory, gastrointestinal, urinary, sexual function, and miscellaneous. Each item is rated from 0 (none) – 3 (severe) for severity and from 1 (rarely) to 4 (very frequent) and the score for each item is the product of the severity rating multiplied by the frequency.

The NMSS takes 20-30 minutes and is to be completed by the PI or designee when the subject is in the ON state. This scale is administered at Weeks e12, e24 and e40.

5.7.1.6 PDQ-39/EQ-5D

The PDQ-39 (Appendix I) and the EQ-5D (Appendix I) are both subject self-report measures of quality of life. The PDQ-39 is a PD-specific quality of life tool with 39 questions. The EQ-5D is a generic quality of life scale with 5 questions and a health "thermometer." These scales will be completed when the subject is in the ON state during their visit. The subject may receive assistance with the physical completion of the scales but the subject alone must determine the answer provided. The scales will take approximately 20 minutes for the subject to complete. The PDQ-39 and EQ-5D are completed at the end of the extension study (Week e40).

5.7.2 Safety Outcome Measures

5.7.2.1 Montreal Cognitive Assessment

The MoCA (Appendix C) is a cognitive screening tool which assesses both cortical and subcortical function. It has 8 components – visuospatial/executive, naming, memory, attention, language, abstraction, delayed recall and orientation, and the total score ranges from 0 – 30 with lower scores representing poorer cognitive function. It takes approximately 30 minutes to administer and will be performed when the subject is in the ON state at Week e16 and at the end of the extension study (Week e40).

Any trained site personnel may complete this scale. Subjects must score a minimum of 24 on the MoCA at the end of Study 2553 to be eligible for the study.

Learning effects are always of high importance in investigations such as this. However, it is planned to use parallel versions of the MoCA to minimize the impact of learning.

5.7.2.2 Mattis Dementia Rating Scale (MDRS)

The MDRS is a global scale of cognition that is sensitive to the frontal/subcortical deficits that are common in PD. It includes 5 subscales – attention, initiation/ perseveration, construction, conceptualization and memory. Scores range from 0 – 144 with higher scores representing better cognitive function. In PD, scores < 123 are associated with some degree of dementia. The MDRS can be analysed using total scores or using individual subscale scores.

The MDRS must be administered by a trained rater with the subject in the ON state and takes 30 – 45 minutes to complete. It will be performed at the end the extension study (Week e40).

5.7.2.3 Stroop

The Stroop is a cognitive test that measures the capacity to direct attention. In this test subjects are presented with words that are the names of colours but are printed in a different colour of ink than the name represents (e.g., "green" printed in red). In the first component of the test, the subject is required to read the colour names and disregard the colour of the text. The second component requires the naming of the colour of the word instead of reading the word.

The Stroop takes 5-10 minutes to complete and is administered with the subject in the ON state at the end of the extension study (Week e40).

5.7.2.4 Other Brief Measures of Cognitive and Executive Function

Cognitive testing has two aspects in this trial. The first is to demonstrate safety, i.e. an absence of cognitive decline and, more broadly, identify potential issues of lowering of mood, suicidality and impulse control. The second is to assess efficacy – particularly focusing upon cognitive functions served by the putamen and fronto-striatal circuits.

We wish to perform an informed measure of "premorbid function". The best measure is the National Adult Reading Test (NART). This is a very brief reading based estimate of premorbid cognitive function and quick to apply.

Given the high functioning status and young age of the individuals putting themselves forward for the preceding double blind study, measures such as MoCA and MDRS may not be sufficiently sensitive for potential minor deterioration. A sensitive measure of overall decline, however, can be calculated by the discrepancy between NART at baseline (as a premorbid measure) and a measure of "fluid intelligence" or general problem solving at 9 months employing the Cattell test. The Cattell test is quick and easy to administer. The advantage is that this measure has been validated longitudinally by Ian Deary and colleagues. The Cattell culture fair test (adult version, form 3) http://www.hogrefe.co.uk/culture-fair-intelligence-tests.html is quick (approximately 10 minutes) to complete. We will administer at follow up (9 months) only to look at change in overall cognitive function compared with NART at baseline.

Furthermore, as a safety measure, we have undertaken to document in the case notes deterioration in mood or impulsivity as assessed by direct questioning when the participant comes for each infusion visit (as one would do at a routine clinic review). We are not including a suicide risk scale assessment, but if someone reports significant lowering of mood we will enquire about suicidal thoughts etc. and document this in the case notes.

As an efficacy measure, we are employing the Stroop using a four card version that involves switching on the last card. In addition, however, we would like to add a more detailed assessment of verbal fluency than contained within the MoCA at baseline and 9

months. A potential difficulty with the Stroop alone is that participants can spot a mastering technique.

With regards to specific assays for putamen-related non motor functions, we are adding a choice reaction time task which is very sensitive, relies on putamenal dopamine and is short. We have Stroop at nine months post baseline and verbal fluency will be assessed at the same time points. Choice reaction time, however, as well as being assessed at baseline and nine months will be checked midway through the nine-month period, given the minimal impact of practice effects, performed at the time the MoCA is repeated. The Deary-Liewald reaction time RT tasks measure simple and choice (i.e. decision) reaction times. This has been validated in longitudinal studies, is suitable for older individuals, does not show much in the way of practice effects.

We are measuring height and weight, since BMI change might be an important outcome. We are also measuring appetite as a useful further outcome measure (4 item SNAQ). Posterior lateral putamen dopamine appears to be critical for eating.

Lastly, a self-report measure of executive function (FRSBE) is included at baseline and 9 months. This is very brief and easy to do. A self-report measure is helpful given our concerns around placebo effects.

5.7.2.5 Beck Depression Inventory

The BDI (Appendix D) is a depression scale that is commonly used both in clinical trials and in clinical practice. It has been recommended by the Movement Disorders Society as an outcome measure to rate the severity of depression in PD. The BDI is a 21-question subject self-report scale with each item being rated on a scale of 0 - 3. Higher scores represent greater degrees of depression.

The BDI will be completed by the subject in the ON state at the end of the extension study (Week e40).

5.7.2.6 Questionnaire for Impulsive- Compulsive Disorders in Parkinson's Disease

The QUIP (Appendix E) was developed to assess the occurrence of impulsive and compulsive disorders in PD. It is a subject self-administered scale which includes 13 questions covering symptoms related to the 4 commonest impulse control disorders in PD (gambling, sex, buying and eating) as well as other behaviours and problematic use

of medication. The QUIP is to be completed by the subject while in the ON state at Weeks e8, e16, e24, e32 and e40.

In addition, we will ask the spouse or partner to also complete a QUIP for the participant.

5.7.2.7 University of Pennsylvania Smell Identification Test British

The UPSIT can be self-administered and uses microencapsulated odorants, which are released by scratching standardised odor-impregnated test booklets. The study uses the British version of the UPSIT. The test can identify most malingerers and is sensitive to age, gender, smoking habits and a wide variety of olfactory disorders. The UPSIT can reliably identify and quantitate olfactory dysfunction in PD. The UPSIT is to be completed regardless of ON/OFF state, at the end of the extension study (Week e40).

5.8 Substudies

Independent substudies may be performed. Separate protocols will be prepared to describe the scope, objectives and procedures of these substudies.

5.9 Definition of End of Trial

The end of trial is the date of the last visit of the last subject.

5.10 Discontinuation/ Withdrawal of Subjects from Study Treatment

Each subject has the right to withdraw from the study at any time. In addition, the PI may discontinue a participant from the study at any time if considered necessary for any reason including:

- Pregnancy
- Ineligibility (either arising during the study or retrospectively, not having been identified at screening)
- Significant protocol deviation
- Significant non-compliance with treatment regimen or study requirements
- An AE which requires discontinuation of the study medication or results in inability to continue to comply with study procedures

- Disease progression which requires discontinuation of the study medication or results in inability to continue to comply with study procedures
- Consent withdrawn
- Lost to follow up

Subjects who discontinue the study early will be encouraged to observe the visit schedule for study assessments, at minimum the final visit (Section 5.6.4) unless they withdraw consent to do so.

The reason for withdrawal will be recorded in the EDC (withdrawal of consent or loss to follow-up will be recorded as such).

If the participant is withdrawn due to an AE, the PI or designee will arrange for follow-up visits or telephone calls until the AE has resolved or stabilised.

5.11 Source Data

Source documents are original documents, data, and records from which participants' EDC data are obtained. These include, but are not limited to, hospital records (from which medical history and previous and concurrent medication may be summarised into the EDC), clinical and office charts, laboratory and pharmacy records, diaries, microfiches, radiographs, and correspondence.

For subject-completed assessments (questionnaires, etc), the subject will complete paper copies of the assessments, which will be transcribed into the EDC by data management staff.

All documents will be stored safely in confidential conditions. On all study-specific documents, other than the signed consent, the participant will be referred to by the study participant number/code and initials, not by name.

6 TREATMENT OF TRIAL PARTICIPANTS

6.1 Description of Study Medication

The active drug is GDNF:

Chemical name:	Recombinant-methionyl human glial cell line-derived neurotrophic factor (r-metHuGDNF)
Physical description:	Clear, colourless liquid
Purity	Purity assumed to be 100% for dose calculation purposes
Concentration:	10 mg/mL in a buffer of 10 mM sodium citrate and 150 mM sodium chloride at pH 5.0
Container size:	2.0 mL
Fill size:	0.5 mL
Storage conditions:	-20 ± 5 °C

GDNF drug substance is prepared on behalf of MedGenesis Therapeutix, Inc., by Lonza Ltd., Visp, Switzerland; GDNF drug product at Aptuit Ltd., Glasgow, UK.

Artificial cerebrospinal fluid (CSF) will also be supplied. The artificial CSF (aCSF) is manufactured on behalf of MedGenesis Therapeutix, Inc. at Aptuit Ltd., Glasgow, UK and provided as:

Physical description:	Clear, colourless liquid
Container size:	20.0 mL
Fill size:	19.8 mL
Storage conditions:	15-30°C

Both GDNF and aCSF will be stored at the study site Pharmacy in secure, limited-access and temperature-controlled conditions.

6.2 Description of Convection-Enhanced Delivery System

The drug delivery system comprises 4 microcatheters, 4 catheter guide tubes and a skull mounted transcutaneous drug delivery port. The microcatheters are connected under the scalp to separate in-line bacterial and bubble filters and further to the drug delivery port.

6.2.1 Implantation of the Drug Delivery System

The drug delivery system will have been implanted during the parent study; please refer to Protocol 2553 for details.

6.2.2 Drug Infusions

The drug administration system compromises 4 programmable syringe pumps, four 5-mL Plastipak syringes (Becton Dickinson), 4 sealed extension lines with male Luer attachments (each containing a septum) at one end, 4 double-sided syringe connectors with female Luer attachments (each holding an internal needle), 4 lengths of sealed drug administration tubing with male Luer attachments (each containing a septum) at both ends and a 4-channelled drug administration set. The latter compromises 4 needles mounted in a hub, each connected to independent bacteria and bubble filters and thence to short lengths of tubing, each with a female Luer attachment holding an internal needle. In addition, 3 syringe connectors with female Luer attachments at both ends and holding an internal needle at one end are used by the pharmacist preparing the study drug to fill the sealed extension lines and sealed drug administration lines.

Prior to drug administration, the pharmacist will fill the 4 lengths of drug administration tubing with an appropriate dose of drug (using 2 syringe connectors, one at each end of the sealed tubing), and label each line appropriately without identifying the treatment allocation. The pharmacist will further fill 4 syringes with aCSF, connect them to the open ends of the extension lines and fill the extension lines (using a single syringe connector at the opposite end to evacuate air). All syringe connectors will be disconnected and discarded once all lines have been filled. The filling of devices will take place up to 48 hours before a planned drug infusion.

For the infusion, each syringe/sealed extension line assembly will be connected to a double-sided-syringe connector, then to a drug administration line, then to one of the 4 channels of the drug administration set which prior to this has been flushed with aCSF.

Connection of the drug administration set to the port begins with the health professional cleaning the port and the immediate surrounds with an aseptic technique. The cylindrical needle hub of the drug administration set will be positioned over the port and locked to the port using a socket screw key. The 4 needles will be lowered through the

port septum by use of a hand-tightened nut and thence individually guided into their separate channels that conduct the drug to identifiable catheters.

Once the connection has been secured, study medication is administered as detailed in Section 6.3. When the infusion is complete after approximately 90 to 150 minutes the administration set will be disconnected from the port by unscrewing both the nut and the socket screw.

6.3 Treatment

Study treatments will be prepared by a pharmacist at the study site. Ready to use preparations of GDNF will be provided to the PI or designee. Drug preparations guidelines are provided in a separate pharmacy manual.

At each treatment, infusion tubes will be connected with the transdermal port using standard aseptic technique. Every subject will receive study drug intraputamenally titrated up to an infusion rate of 3-5 μL/minute. The infusion rates will be up-titrated following a linear ramping scheme from 0 μL/min to 3-5 μL/min (0.18-0.30 mL/hour) over 30 to 40 minutes. The infusions will be delivered by external syringe pumps with pre-programmed infusion algorithms (Perfusor® Space, B. Braun, Melsungen, Germany).

Two catheters per putamen will be used, and 300 μL of study drug will be delivered per catheter at each treatment. This amounts to a dose of 120 μg GDNF per putamen per administration. An aCSF "flush" of up to 100 μL per catheter (at 3-5 μL/min) will clear the dead space after each infusion.

The infusions will be delivered by trained personnel. During the infusion, subjects will be semi-recumbent in a reclining chair. The total procedure time for the completion of the infusions will be approximately 90 to 150 minutes depending on potential reflux parameters observed on post infusion MRIs.

6.4 Compliance with Study Treatment

All treatments will be administered by PI or designee. Administration records will be kept on site, and administration information, including time and date of infusion, identification of the infusate, infusion rate and duration and the reasons for any

interruptions of the infusion or for any missed or omitted infusions, will be recorded in the EDC.

6.5 Accountability of the Study Treatment

It is forbidden to use investigational drug material for purposes other than as defined in this protocol.

All supplies of study medication will be accounted for in accordance with Good Clinical Practices (GCP). There will be an individual study drug accountability record for each subject and the pharmacist is to maintain accurate records of the disposition of all study medication supplies received and dispensed during the study. These records will include the amounts and dates clinical drug supplies were received, dispensed to the PI or designee for any given subject, and returned to the manufacturer. If errors or damages in the clinical drug supply shipments occur, the pharmacist will notify the manufacturer immediately so that corrective action can be taken as needed. Copies of the study medication accountability records will be provided by the pharmacist for inclusion in the Trial Master File (TMF) after database lock. The study monitor will periodically check the supplies of study medication held by the pharmacist to verify accountability of all medication used.

The PI or designee will administer the medication only to the identified subjects of this study, according to the procedures described in this study protocol. After the end of the study, all unused medication and all medication containers can be destroyed on-site as long as proper documentation is supplied. If destruction on-site is not possible then medication and all medication containers will be returned to the manufacturer for destruction and documentation will be returned to the manufacturer. The manufacturer will verify that a final report of drug accountability is prepared and maintained in the Investigator's Study Centre File.

6.6 Concomitant Medication

Subjects should have PD medication optimised and stabilised prior to study entry. Every effort should be made not to increase subjects' medication for PD during the study, but medication may be increased if required to maintain the subject's well being. PD medication dose may be decreased at the discretion of the PI to manage PD drug-

related side-effects. Non-PD medications may be altered at any time at the PI's discretion.

Throughout the study the PI may prescribe any concomitant medications or treatments deemed necessary to provide adequate supportive care.

Any medication other than the study medication, including prescription or over-the-counter medications, vitamins and herbal medications taken during the study will be recorded in the EDC with their indications.

7 SAFETY REPORTING

7.1 Definitions

7.2 Adverse Event (AE)

An AE or adverse experience is:

Any untoward medical occurrence in a subject or clinical investigation participant administered a medicinal product, which does not necessarily have a causal relationship with this treatment (the study medication).

An AE can therefore be any unfavourable and unintended sign (including an abnormal laboratory finding), symptom or disease temporally associated with the use of the study medication, whether or not considered related to the study medication.

7.3 Adverse Reaction (AR)

All untoward and unintended responses to a medicinal product related to any dose.

The phrase "responses to a medicinal product" means that a causal relationship between a study medication and an AE is at least a reasonable possibility, i.e., the relationship cannot be ruled out.

All cases judged by either the reporting medically qualified professional or the Sponsor as having a reasonable suspected causal relationship to the study medication qualify as ARs.

7.4 Serious Adverse Event (SAE)

An SAE is any untoward medical occurrence that at any dose:

- Results in death
- Is life-threatening; NOTE: The term "life-threatening" in the definition of "serious" refers to an event in which the participant was at risk of death at the time of the event; it does not refer to an event which hypothetically might have caused death if it were more severe.
- Requires inpatient hospitalisation or prolongation of existing hospitalisation
- Results in persistent or significant disability/incapacity

- Is a congenital anomaly/birth defect
- Other important medical events; NOTE: Other events that may not result in death, are not life-threatening, or do not require hospitalisation, may be considered an SAE when, based upon appropriate medical judgement, the event may jeopardise the subject and may require medical or surgical intervention to prevent one of the outcomes listed above.

To ensure no confusion or misunderstanding of the difference between the terms "serious" and "severe," which are not synonymous, the following note of clarification is provided:

The term "severe" is often used to describe the intensity (severity) of a specific event (as in mild, moderate, or severe myocardial infarction); the event itself, however, may be of relatively minor medical significance (such as severe headache). This is not the same as "serious," which is based on subject/event outcome or action criteria usually associated with events that pose a threat to a participant's life or functioning as defined in the bullet points above. Seriousness (not severity) serves as a guide for defining regulatory reporting obligations.

7.5 Serious Adverse Reaction (SAR)

An AE (expected or unexpected) that is both serious and, in the opinion of the reporting Investigator, believed with reasonable probability to be due to one of the study treatments, based on the information provided.

7.6 Suspected Unexpected Serious Adverse Reaction (SUSAR)

An SAR, the nature or severity of which is not consistent with the applicable product information (e.g., Investigator's Brochure for an unapproved investigational product or Summary of Medicinal Product Characteristics [SMPC] for an approved product).

7.7 Causality and Expectedness

The relationship of each AE to the trial medication must be determined by a medically qualified individual according to the following definitions:

Related: The AE follows a reasonable temporal sequence from trial medication administration. It cannot reasonably be attributed to any other cause.

Not Related: The AE is probably produced by the participant's clinical state or by other modes of therapy administered to the participant.

The expectedness of each **SAE** in the trial must be determined by a medically qualified individual according to the following definition:

Unexpected Adverse Drug Reaction: An adverse reaction, the nature or severity of which is not consistent with the applicable product information (i.e., Investigator's Brochure).

7.8 Procedures for Recording Adverse Events

All AEs occurring during the study until Week 82 (or 28 days after the last dose of study medication for subjects who discontinue study early), observed by the PI or designee or reported by the participant, whether or not attributed to study medication, will be recorded in the EDC.

The following information will be recorded: description, date of onset and end date, severity, assessment of relatedness to study medication, other suspect drug or device and action taken. Follow-up information should be provided as necessary.

AEs considered related to the study medication as judged by the PI or a medically qualified designee will be followed until resolution or the event is considered stable. All related AEs that result in a participant's withdrawal from the study or are present at the end of the study should be followed up until a satisfactory resolution occurs.

It will be left to the PI's or medically qualified designee's clinical judgment whether or not an AE is of sufficient severity to require the participant's removal from treatment. A participant may also voluntarily withdraw from treatment due to what he or she perceives as an intolerable AE. If either of these occurs, the participant must undergo an end of study assessment (unless he or she has withdrawn consent to do so) and be given appropriate care under medical supervision until symptoms cease or the condition becomes stable.

The severity of events will be assessed on the following scale:
1 = mild, 2 = moderate, 3 = severe.

The relationship of AEs to the study medication will be assessed by the PI or a medically qualified designee.

Any pregnancy occurring during the clinical study and the outcome of the pregnancy should be recorded and followed up for any congenital abnormality or birth defect.

7.9 Reporting Procedures for Serious Adverse Events

All SAEs must be reported to North Bristol NHS Trust Research and Innovation (NBT R&I) within 1 working day of discovery or notification of the event. NBT R&I will perform an initial check of the report, request any additional information and ensure it is reviewed. All SAEs must be reviewed at the next Trial Safety Group meeting. All SAE information must be recorded on an SAE form and faxed or emailed to NBT R&I. Additional information received for a case (follow-up or corrections to the original case) need to be detailed on a new SAE form and faxed or emailed to NBT R&I.

7.10 SUSAR Reporting

The PI will support NBT R&I to report all SUSARs to the MHRA, the local Research Ethics Committee (REC), and the manufacturer of the drug. Fatal or life-threatening SUSARs must be reported within 7 days and all other SUSARs within 15 days. The PI will inform all study staff concerned of relevant information about SUSARs that could adversely affect the safety of participants.

7.11 Annual Safety Reports

In addition to the expedited reporting above, the PI shall submit once a year throughout the clinical trial or on request a safety report to the MHRA, REC, and manufacturer of the drug.

8 STATISTICS

A full, detailed statistical analysis plan (SAP) will be prepared and completed before study data are locked.

8.1 Description of Statistical Methods

Descriptive statistics will be presented for all endpoints by treatment group supporting the primary and secondary analyses. Detailed methodology will be provided in the SAP. No inferential analyses are planned for this open-label extension study.

The efficacy analyses will be conducted after all Primary Study Stage subjects have reached the 18-month endpoint and will include only subjects who had been randomised to the Primary Study Stage of Study 2553. Supplementary analyses will include subjects treated in the Pilot Extension Stage.

Safety data will be presented descriptively, by treatment group, with standard Medical Dictionary for Regulatory Activities (MedDRA) coded AE and SAE frequency and incidence tables as well as shift tables for clinical laboratory parameters.

The Safety Population will comprise all subjects treated on study with subjects grouped according to treatment actually received. All safety analyses will be performed on the Safety Population.

8.2 Number of Participants

This is an open-label extension study. No power calculations were performed. The study will be open to all subjects who complete Study 2553 without significant toxicity or other exclusion.

8.3 Level of Statistical Significance

No inferential analyses are planned for this open-label extension study.

8.4 Criteria for the Termination of the Trial

The study will be monitored by an independent DMC established by the sponsor or designee and governed by a separate charter. The DMC may recommend termination of the trial at any point for safety reasons.

This study may be terminated by the Sponsor. The study may also be terminated prematurely at any time when agreed to by both the PI and the Sponsor as being in the best interests of subjects, and justified on either medical or ethical grounds. In terminating the study, the Sponsor and the PI will ensure that adequate consideration is given to the protection of the subjects' interests.

8.5 Procedure for Accounting for Missing, Unused, and Spurious Data

Methods for handling missing or incomplete data will be described in the SAP.

8.6 Procedures for Reporting any Deviation(s) from the Original Statistical Plan

Changes to the SAP planned before study data are locked will be documented in a revised SAP or SAP addendum or amendment. Any subsequent changes to the planned analyses will be clearly identified in the final Clinical Study Report.

8.7 Inclusion in Analysis

The primary efficacy analyses will be conducted after all Primary Study Stage subjects have reached the 18-month endpoint and will include only subjects who had been randomised to the Primary Study Stage of Study 2553. Supplementary analyses will include subjects treated in the Pilot Extension Stage.

The Safety Population will comprise all subjects treated on study with subjects grouped according to treatment actually received. All safety analyses will be performed on the Safety Population. They will be performed separately for the subjects who had been randomised to the Pilot and Primary Study Stages of Study 2553 and for all subjects and both stages combined.

Additional analysis populations may be defined in the SAP.

9 DIRECT ACCESS TO SOURCE DATA/DOCUMENTS

Direct access will be granted to authorised representatives from the Sponsor, host institution, manufacturer and the regulatory authorities to permit trial-related monitoring, audits and inspections.

10 QUALITY CONTROL AND QUALITY ASSURANCE PROCEDURES

The study will be conducted in accordance with the current approved protocol, International Conference on Harmonisation (ICH) GCP, relevant regulations and standard operating procedures.

Regular monitoring will be performed according to ICH GCP. The Sponsor or designee will complete a monitoring plan and provide a copy for the TMF. Data will be evaluated for compliance with the protocol and accuracy in relation to source documents. Following written standard operating procedures, the monitors will verify that the clinical trial is conducted and data are generated, documented and reported in compliance with the protocol, GCP and the applicable regulatory requirements.

The Sponsor or designee will establish an independent DMC. The establishment and operation of the DMC will adhere to relevant guidance, and the responsibilities and processes of the DMC will be specified in a DMC charter.

11 SERIOUS BREACHES

The Medicines for Human Use (Clinical Trials) Regulations contain a requirement for the notification of "serious breaches" to the MHRA within 7 days of the Sponsor becoming aware of the breach.

A serious breach is defined as "A breach of GCP or the trial protocol which is likely to affect to a significant degree –

(a) the safety or physical or mental integrity of the subjects of the trial; or

(b) the scientific value of the trial."

In the event that a serious breach is suspected NBT R&I should be contacted as soon as possible. Breaches of GCP will be managed in accordance with ISOP-C05 Noncompliance with ICH Good Clinical Practice (GCP) Guidelines (http://www.nbt.nhs.uk/education__research/researcher_resources/useful_documents__forms/standard_operating_procedures.aspx)

12 ETHICS

12.1 Declaration of Helsinki

The PI will ensure that this study is conducted in accordance with the principles of the Declaration of Helsinki (as amended by the World Medical Association in Seoul, Republic of Korea, October 2008).

12.2 ICH Guidelines for Good Clinical Practice

The PI will ensure that this study is conducted in full conformity with relevant regulations and with the ICH Guidelines for GCP (CPMP/ICH/135/95) July 1996.

12.3 Approvals

The protocol, informed consent form, participant information sheet and any proposed advertising material will be submitted to the appropriate REC, MHRA, and applicable host institution(s) for written approval.

The PI will submit and, where necessary, obtain approval from the above parties for all substantial amendments to the original approved documents.

12.4 Participant Confidentiality

The trial staff will ensure that the participants' anonymity is maintained. The participants will be identified only by initials and a participant identification number in the EDC and any written study materials. All documents will be stored securely and only accessible by trial staff and authorised personnel. The study will comply with the Data Protection Act which requires data to be anonymised as soon as it is practical to do so.

13 DATA HANDLING AND RECORD KEEPING

An EDC system will be used for the current study and a Data Management Plan will be prepared by the Sponsor or designee. The EDC data will be compared to source documentation by the study monitors. All monitored data will be processed by a data management group. Data edit checks will be performed using both EDC and SAS® programming as detailed in the Data Management Plan. Data queries will be issued to the clinical site in order to resolve any discrepancies found during the discrepancy management process, and data will be updated accordingly. The participants will be identified by a study specific participant number and/or code in any database. The name and any other identifying detail will NOT be included in any study data electronic file.

Previous and concomitant medications will be coded using the latest available World Health Organization (WHO) Drug Reference Dictionary. Coexistent diseases and AEs will be coded using MedDRA.

When the database has been declared to be complete and accurate, it will be locked. Any changes to the database after that time can only be made by written agreement and documented in the TMF.

14 FINANCE AND INSURANCE

14.1 Compensation for Harm

Good governance in respect of the monitoring of the trial will be provided through NBT, acting as the Sponsor of this trial. NBT undertakes to maintain an appropriate clinical study insurance policy. In the event that something does go wrong and participants are harmed during the research and this is due to someone's negligence, then participants may have grounds for a legal action for compensation against NBT. However, participants may have to pay their legal costs.

With NBT as the Sponsor, this project is covered by NHS Litigation Authority Indemnity Insurance.

This is a non-commercial trial and as such is mandated to have indemnity in respect of negligent harm only; there is no provision for indemnity in respect of liabilities arising from non-negligent harm. In other words, if participants come to harm through this trial due to proven negligence they will be able to attempt to claim under the negligent harm scheme. However, unforeseen adverse events (and we do not know all the potential long term complications of receiving GDNF therapy or having catheter systems in the brain) or adverse events that occur without there being negligence on the part of the investigating team are not covered by this scheme. This will be explained in detail to participants at the time they consent to participate in the double-blind study.

Deviations from the study protocol – especially the prescription of a dose other than that scheduled in the study protocol, other modes of administration, other indications, and longer treatment periods – are not permitted and shall not be covered by the statutory subject insurance scheme.

15 PUBLICATION POLICY

Authorship of any publication will be based upon substantial contribution to the design, analysis, interpretation of data, drafting and/or critically revising any manuscript(s) derived from the Study. The Sponsor, PI and/or Study Neurosurgeon shall have the right to publish the results of the Study. Intended publications employing data from the study will be discussed with all members of the Steering Committee in advance of writing up to allow all members to contribute.

The Drug Manufacturer must receive copies of any intended communication at least sixty (60) days in advance to review a manuscript and fifteen (15) days to review any poster presentation, abstract or other written or oral material which describes the results of the Study. In addition, if Drug Manufacturer requests in writing, the Sponsor, PI and/or Study Neurosurgeon shall withhold any publication or presentation an additional thirty (30) days to allow for further clarification.

16 REFERENCES

[1] Lin LF, Doherty DH, Lile JD, Bektesh S, Collins F. GDNF: a glial cell line-derived neurotrophic factor for midbrain dopaminergic neurons. Science 1993;260(5111):1130-2.

[2] Airaksinen MS, Saarma M. The GDNF family: signalling, biological functions and therapeutic value. Nat Rev Neurosci 2002;3(5):383-94.

[3] Bobo RH, Laske DW, Akbasak A, Morrison PF, Dedrick RL, Oldfield EH. Convection-enhanced delivery of macromolecules in the brain. Proc Natl Acad Sci U S A 1994;91(6):2076-80.

[4] Fiandaca MS, Forsayeth JR, Dickinson PJ, Bankiewicz KS. Image-guided convection-enhanced delivery platform in the treatment of neurological diseases. Neurotherapeutics 2008;5(1):123-7.

17 APPENDIX A: STUDY SCHEDULE OF EVENTS

Table 1 Schedule of Events

Procedure/Assessments	Week										
	e0 [a]	e4	e8 [b]	e12	e16 [b]	e20	e24 [b]	e28	e32 [b]	e36	e40 [b]
Informed consent [c]	(X)										
MRI [d]					X					X	
Vital signs [e]	X	X	X	X	X	X	X	X	X	X	X
Weight and height	(X)		X		X		X				X
Physical examination [f]											X
ECG											X
Laboratory assessment [g]		X			X			X			X
Anti-GDNF antibody levels and GDNF plasma concentration		X			X			X			X
UPDRS part II and part III in OFF state	(X)		X		X		X		X		X
Timed walking test in OFF state	(X)		X		X		X		X		X
Timed tapping test in OFF state	(X)		X		X		X		X		X
Levodopa challenge	(X)		X		X		X		X		X
UPDRS in on state	(X)		X		X		X		X		X
Timed walking test in on state	(X)		X		X		X		X		X
Timed tapping test in on state	(X)		X		X		X		X		X
PDQ-39											X
EQ-5D											X
MoCA & MDRS					X						X
Stroop test											X
UPSIT											X
NMSS				X			X				X
BDI											X
QUIP	(X)		X		X		X		X		X
Choice reaction time					X						X
SNAQ											X
Cattell culture fair test											X
Executive function self report											X
Verbal fluency											X
Direct questioning of impulsivity, mood, falls and freezing for recording in case notes	X	X	X	X	X	X	X	X	X	X	X
Collect PD fluctuation diaries	(X)		X		X		X		X		X
Dispense PD fluctuation diaries		X		X	X	X		X		X	
Infusion of study drug	X	X	X	X	X	X	X	X	X	X	
Glasgow Coma Scale [h]	X	X	X	X	X	X	X	X	X	X	
Adverse events [i]	X	X	X	X	X	X	X	X	X	X	X
Concomitant medications [j]	X	X	X	X	X	X	X	X	X	X	X

a Assessments that are put in parentheses will be done at the Week 40 visit in Study 2553 or earlier (informed consent). With the exception of informed consent, no procedures are required specifically for the extension study; however, procedures scheduled for Week 40 in Study 2553 must be performed in accordance with that protocol.

b At Weekse8, e16, e24 and e32, no PD medications will be taken after 6:00 PM on the night before the assessments and no long-acting PD medications will be taken on the day before the assessments. Subjects will refrain from eating any high-protein foods on the morning of the assessments.

c Informed consent must be obtained before any extension study-specific procedures or assessments are performed.

d T2-weighted and FLAIR 3T MRI are to be completed within 2 hours of study drug infusion.

e Seated systolic and diastolic BP, HR, RR and temperature will be assessed after the subject has been sitting quietly for at least 5 minutes. Standing systolic and diastolic BP and HR will also be assessed after the subject has been standing for 3 minutes. During administration, seated vital signs (systolic and diastolic BP, HR and RR) will be collected every 15 (Week e0) to 30 (Weeks e4 to e36) minutes, and both seated and standing systolic and diastolic BP and HR will be assessed approximately minutes after the end of the infusion.

f Brief physical examination, targeted, at the Investigator's discretion, to identify changes from baseline and from the previous assessment.

g Haematology (haematocrit, haemoglobin, MCH, MCHC, MCV, platelet count, RBC count, WBC count and WBC differential [basophils, eosinophils, lymphocytes, monocytes and neutrophils]), serum chemistry (albumin, alkaline phosphatase, ALT, creatinine, eGFR, glucose, potassium, sodium, total bilirubin and urea) and urinalysis (colour, appearance, pH, glucose, ketones and nitrite; microscopy as required to follow up abnormal observations).

h Performed before infusion, 30 minutes into the infusion, and after completion of the infusion.

i All AEs observed or reported from the time of the first imaging procedure until 28 days after the last dose of GDNF will be recorded in the Adverse Event EDC. AEs meeting the criteria for SAEs (Section 7.4) must be reported as directed in Section 7.9

j Concomitant medications: from study entry and until Week e40 or early discontinuation will be recorded. All over-the-counter or prescription medications, vitamins and/or herbal supplements will be recorded in the EDC with their indications.

Study Title

An Extension Study to Assess the Safety and Efficacy of Intermittent Bilateral Intraputamenal Glial Cell Line-Derived Neurotrophic Factor (GDNF) Infusions Administered via Convection Enhanced Delivery (CED) in Subjects with Parkinson's Disease

Internal Reference No: 2797

Ethics Ref: 13/SW/0181

EudraCT Number: 2013-001881-40

Version 1.4, 16 December 2015 (Incorporating Amendments 1-4)

Principal Investigator: Dr A Whone
Consultant Neurologist and Hon Senior Lecturer
Department of Neurology
Movement Disorder Service
Southmead Hospital
Bristol BS10 5NB, United Kingdom

Study Neurosurgeon: Professor S Gill
Consultant Neurosurgeon
Department of Neurosurgery
Southmead Hospital
Bristol BS10 5NB, United Kingdom

Sponsor: North Bristol NHS Trust (NBT)
Research & Innovation
Southmead Hospital
Bristol BS10 5NB, United Kingdom

Funding Sources: Parkinson's UK
215 Vauxhall Bridge Road
London SW1V 1EJ, United Kingdom

The Cure Parkinson's Trust
The Vestry
1, St Clement's Court
London EC4N 7HB, United Kingdom

Drug Manufacturer: MedGenesis Therapeutix, Inc.
730-730 View Street
Victoria, BC V8W 3Y7, Canada

Protocol authorised by:

Dr Alan Whone **16.12.2015**

TABLE OF CONTENTS

STUDY TITLE 1
1 SYNOPSIS 8
2 ABBREVIATIONS 11
3 BACKGROUND AND RATIONALE 13
4 OBJECTIVES 14
4.1 Primary Objective 14
4.2 Secondary Objectives 14
4.3 Other Objectives 14
5 TRIAL DESIGN 15
5.1 Summary of Trial Design 15
5.1.1 Overview 15
5.1.2 Post Study Access to Treatment 18
5.2 Study Endpoints 19
5.2.1 Primary Endpoint 19
5.2.2 Secondary Endpoints 19
5.2.3 Supplementary Efficacy Endpoints 20
5.2.4 Imaging Endpoints 20
5.2.5 Safety Endpoints 20
5.3 Trial Participants 21
5.3.1 Overall Description of Trial Participants 21
5.3.2 Inclusion Criteria 21
5.3.3 Exclusion Criteria 21
5.4 Expenses and Benefits 22
5.5 Study Procedures 22
5.5.1 Informed Consent 22
5.5.2 Screening and Eligibility Assessment 23
5.5.3 Preparation of Study Drug 23
5.6 Schedule of Treatments and Assessments 24
5.6.1 Treatments 24
5.6.2 Interim Assessments 26
5.6.3 Laboratory and Other Assessments 27
5.6.4 Week e40 or Early Discontinuation 27

5.7 Pilot Extension....30
5.7.1 Study Procedures....30
5.7.2 Treatments....31
5.7.3 Interim Assessments....31
5.7.4 Other Assessments....32
5.7.5 Week e2-80 or Early Discontinuation....32
5.8 Supplemental Extension....32
5.8.1 Study Procedures....33
5.8.2 Treatments....33
5.8.3 Interim Assessments....34
5.8.4 Study Completion or Early Discontinuation....34
5.9 Outcomes Measures....35
5.9.1 Efficacy Outcome Measures....35
5.9.2 Safety Outcome Measures....37
5.10 Substudies....41
5.11 Definition of End of Trial....41
5.12 Discontinuation/ Withdrawal of Subjects from Study Treatment....41
5.13 Source Data....42
6 TREATMENT OF TRIAL PARTICIPANTS....43
6.1 Description of Study Medication....43
6.2 Description of Convection-Enhanced Delivery System....44
6.2.1 Implantation of the Drug Delivery System....44
6.2.2 Drug Infusions....44
6.3 Treatment....45
6.4 Compliance with Study Treatment....46
6.5 Accountability of the Study Treatment....46
6.6 Test Infusions....47
6.7 Concomitant Medication....47
7 SAFETY REPORTING....48
7.1 Definitions....48
7.2 Adverse Event (AE)....48
7.3 Adverse Reaction (AR)....48
7.4 Serious Adverse Event (SAE)....48

7.5 Serious Adverse Reaction (SAR) 49
7.6 Suspected Unexpected Serious Adverse Reaction (SUSAR) 49
7.7 Causality and Expectedness 49
7.8 Procedures for Recording Adverse Events 50
7.9 Reporting Procedures for Serious Adverse Events 51
7.10 SUSAR Reporting 51
7.11 Annual Safety Reports 51
8 STATISTICS 52
8.1 Description of Statistical Methods 52
8.2 Number of Participants 52
8.3 Level of Statistical Significance 52
8.4 Criteria for the Termination of the Trial 52
8.5 Procedure for Accounting for Missing, Unused, and Spurious Data 53
8.6 Procedures for Reporting any Deviation(s) from the Original Statistical Plan 53
8.7 Inclusion in Analysis 53
9 DIRECT ACCESS TO SOURCE DATA/DOCUMENTS 54
10 QUALITY CONTROL AND QUALITY ASSURANCE PROCEDURES 55
11 SERIOUS BREACHES 56
12 ETHICS 57
12.1 Declaration of Helsinki 57
12.2 ICH Guidelines for Good Clinical Practice 57
12.3 Approvals 57
12.4 Participant Confidentiality 57
13 DATA HANDLING AND RECORD KEEPING 58
14 FINANCE AND INSURANCE 59
14.1 Compensation for Harm 59
15 PUBLICATION POLICY 60
16 REFERENCES 61
17 APPENDIX A: STUDY SCHEDULE OF EVENTS 62
18 APPENDIX B: SAE REPORTING FLOW CHART ....
19 APPENDIX C: MONTREAL COGNITIVE ASSESSMENT (MOCA) ....
20 APPENDIX D: BECK DEPRESSION INVENTORY (BDI) ....

21 APPENDIX E: QUESTIONNAIRE FOR IMPULSIVE-COMPULSIVE DISORDERS IN PARKINSON'S DISEASE (QUIP)..

22 APPENDIX F: UNIFIED PARKINSON'S DISEASE RATING SCALE (UPDRS)..........

23 APPENDIX G: PD FLUCTUATION DIARY..

24 APPENDIX H: NON-MOTOR SYMPTOM ASSESSMENT SCALE FOR PD (NMSS)..

25 APPENDIX I: PARKINSON'S DISEASE QUESTIONNAIRE-39 (PDQ-39) ....

26 APPENDIX J: EUROQOL 5-DIMENSIONAL SCALE (EQ-5D).....................................

List of Tables

Table 1 Schedule of Events......63
Table 2 Study Procedures of Additional Pilot Extension......65
Table 3 Study Procedures of Supplemental Extension......67

List of Figures

Figure 1 Study Schema......17

AMENDMENT HISTORY

Amendment 1 (02 September 2013)

Harmonisation of the initial reporting period for serious adverse events (SAEs) with current effective regulations.

Amendment 2 (30 June 2014)

Addition of further extension study visits, pilot cohort only (Pilot Extension), correction of minor protocol inconsistencies and removal of the Cattell Culture Fair Intelligence Test as an outcome measure.

Amendment 3 (19 September 2014)

Modification of the magnetic resonance imaging (MRI) schedule.

Amendment 4 (16 December 2015)

Addition of further extension study visits to facilitate continuation of the study until anticipated completion at the end of December 2016 and a number of clarifications and refinements that are considered mostly administrative in nature or have evolved during the preparation of protocol amendment 7 of Study 2553.

1 SYNOPSIS

Study Title	An Extension Study to Assess the Safety and Efficacy of Intermittent Bilateral Intraputamenal Glial Cell Line-Derived Neurotrophic Factor (GDNF) Infusions Administered via Convection Enhanced Delivery (CED) in Subjects with Parkinson's Disease
Internal Ref. No.	2797
Clinical Phase	Phase II
Trial Design	An open-label extension study for subjects who complete Study 2553.
Trial Participants	Subjects with bilateral idiopathic Parkinson's disease (PD), according to the United Kingdom (UK) Brain Bank Criteria, who complete a double-blind study of GDNF administered via CED (Study 2553)
Planned Sample Size	Up to 42 subjects
Treatment and Follow-up Duration	9 months<br>Pilot subjects only will be eligible for up to an additional 80 weeks of treatment (Pilot Extension). Pilot subjects and Primary Study Stage subjects will be eligible for the Supplemental Extension until the end of December 2016.
Planned Trial Period	The planned study period, including recruitment, is approximately 42 months.
Primary Objective	To compare the effects of intermittent bilateral intraputamenal GDNF infusions on OFF-state motor function after 18 months of treatment with the effects after 9 months of treatment in subjects who completed in Study 2553.
Secondary and Other Objectives	• To compare the effects of intermittent bilateral intraputamenal GDNF infusions on ON-state motor function, motor complications, and ON- and OFF-state activities of daily living (ADL) after 18 months of treatment with the effects after 9 months of treatment in subjects who completed Study 2553.<br>• To assess the safety of intermittent bilateral intraputamenal GDNF infusions at 18 months in subjects who received GDNF or placebo for 9 months in Study 2553.<br>• To explore the effects of intermittent bilateral intraputamenal GDNF infusions on other motor and non-motor functions, quality of life assessments, and imaging endpoints at 18 months in subjects who completed Study 2553.<br>• To compare the results for various motor outcomes between the subjects who started GDNF early (i.e. were randomized to GDNF in Study 2553) and those who started GDNF late (i.e. were randomized to placebo in Study 2553).<br>• Pilot and Supplemental Extensions: To generate long-term safety data and provide continued access to GDNF until the end of December 2016 when the results of Study 2553 are expected, which will inform interested parties with potential future studies.
Primary Efficacy Endpoint	Percentage change from baseline to the end of treatment in the practically defined OFF-state Unified Parkinson's Disease Rating Scale (UPDRS) motor score (part III).
Secondary and Other Efficacy Endpoints	Secondary efficacy endpoints will include the following:<br>• Percentage change from baseline to the end of treatment in UPDRS motor score (part III) in the ON-state (following a levodopa challenge).

	• Percentage change from baseline to the end of treatment in UPDRS activities of daily living (ADL) (part II).<br>• Change from baseline to the end of treatment in PD diary ratings; i.e., total OFF-time per day, total good quality ON-time (ON without dyskinesias or ON with non-troublesome dyskinesias) and ON-time with troublesome dyskinesias.<br>• Change from baseline to the end of treatment in PD non-motor assessments of cognition, executive function, mood, other non-motor symptoms, smell, appetite, medication equivalents and quality of life<br>Analysis of the primary and secondary endpoints will be repeated for the overall ITT population including subjects randomised in the Pilot Stage.
Safety Endpoints	Safety endpoints include the following:<br>• Incidence of device-related adverse events (AEs) during the study period.<br>• Incidence of treatment-emergent AEs (related and unrelated) during the study period.<br>• Time with troublesome dyskinesias (from subject diaries).<br>• Clinical assessment of safety, including dyskinesias and falls, mood and impulsivity at each infusion visit.<br>• The Questionnaire for Impulsive-Compulsive Disorders in Parkinson's Disease (QUIP) every 8 weeks.<br>• Changes in the Montreal Cognitive Assessment (MoCA) and Mattis Dementia Rating Scale (MDRS) at the end of the study.<br>• Adverse changes in magnetic resonance imaging (MRI) findings.<br>• Anti-GDNF antibody samples taken at intervals during the study will be assessed at the end of the study.<br>• Routine laboratory blood tests (haematology, serum chemistry) and urinalysis performed at baseline and at intervals during the trial.<br>• Full-brain MRI.
Imaging Endpoints	Volume of distribution of infusate will be determined both by contrast-enhanced T1-weighted and by T2-weighted and fluid-attenuated inversion recovery (FLAIR) 3T MRI. Scans are to be completed within 2 hours of delivering a gadolinium contrast-containing test infusion of diluent at Week e40 in the extension study (Primary Study Stage subjects only) and at Weeks e2-0 and e2-80 in the Pilot Extension. GDNF will not be delivered at any of these visits, and no interim MRI scans will be done during the study unless clinically indicated.
Statistical Analysis	Descriptive statistics will be presented for all endpoints by treatment group supporting the primary and secondary analyses. Detailed methodology will be provided in the statistical analysis plan (SAP). No inferential analyses are planned for this open-label extension study.<br>The efficacy analyses will be conducted after all Primary Study Stage subjects have reached the 18-month endpoint and will include only subjects who had been randomised to the Primary Study Stage of Study 2553. Supplementary analyses will include pilot subjects.<br>Safety data will be presented descriptively, by treatment group, with standard Medical Dictionary for Regulatory Activities (MedDRA) coded AE and SAE frequency and incidence tables as well as shift tables for clinical laboratory parameters.<br>The Safety Population will comprise all subjects treated on study with subjects grouped according to treatment actually received. All safety analyses will be performed on the Safety Population.

Investigational Medicinal Products	GDNF in artificial cerebrospinal fluid (aCSF) at a concentration of 0.2 μg/μL (active treatment)
Form	Intracerebral infusion by CED.
Dose	600 μL (containing 120 μg of GDNF) per putamen every 4 weeks for 9 months for all subjects.<br>Pilot Extension and Supplemental Extension subjects will continue to receive infusions according to the same protocol until the end of December 2016.
Route	Intraputamenal infusion via a CED infusion system with 2 indwelling catheters per putamen.

2 ABBREVIATIONS

aCSF	Artificial cerebrospinal fluid
ADL	Activities of daily living
AE	Adverse event
ALT	Alanine transaminase
AR	Adverse reaction
BDI	Beck Depression Inventory
BP	Blood pressure
CED	Convection enhanced delivery
CRF	Case report form
CSF	Cerebrospinal fluid
DMC	Data Monitoring Committee
ECG	Electrocardiogram
eGFR	Estimated glomerular filtration rate
EQ-5D	EuroQOL 5-dimensional scale
FLAIR	Fluid-attenuated inversion recovery
GCP	Good Clinical Practice
GDNF	Glial cell line-derived neurotrophic factor
HR	Heart rate
ICH	International Conference of Harmonisation
MCH	Mean cellular haemoglobin
MCHC	Mean cellular haemoglobin concentration
MCV	Mean cellular volume
MDRS	Mattis Dementia Rating Scale
MedDRA	Medical Dictionary for Regulatory Activities
MHRA	Medicines and Healthcare Products Regulatory Agency
MoCA	Montreal Cognitive Assessment
MRI	Magnetic resonance imaging
NBT	North Bristol NHS Trust
NBT R&I	North Bristol NHS Trust Research and Innovation
NMSS	Non-Motor Symptom Assessment Scale for PD
PD	Parkinson's Disease
PDQ-39	Parkinson's Disease Questionnaire-39
PI	Principal Investigator
QTc	Corrected QT (interval)
QUIP	Questionnaire for Impulsive-Compulsive Disorders in Parkinson's Disease
RBC	Red blood cell (count)

REC	Research Ethics Committee
RR	Respiration rate
SAE	Serious adverse event
SAP	Statistical analysis plan
SAR	Serious adverse reaction
SMPC	Summary of Medicinal Product Characteristics
SUSAR	Suspected unexpected serious adverse reactions
TMF	Trial Master File
UK	United Kingdom
UPDRS	Unified Parkinson's Disease Rating Scale
UPSIT	University of Pennsylvania Smell Identification Test
WBC	White blood cell (count)
WHO	World Health Organization

3 BACKGROUND AND RATIONALE

Glial cell line-derived neurotrophic factor (GDNF) is a neurotrophic factor with potent effects on diverse nerve cell lines including dopaminergic, serotonergic, noradrenergic, and cholinergic neurones [1, 2]. Intraputamenal GDNF, administered via convection-enhanced delivery (CED) [3, 4], is being investigated as a treatment for Parkinson's disease (PD).

Study 2553 is a placebo-controlled, randomised, double-blind trial to assess the safety and efficacy of intermittent bilateral intraputamenal GDNF infusions administered via CED in subjects with PD. In that study, subjects receive GDNF or placebo q4 weekly for up to 9 months. Please refer to the Study 2553 protocol for further details regarding that study and to the GDNF Investigator's Brochure for background and safety information on GDNF.

This study is an open-label extension study for subjects who complete Study 2553. All enrolling subjects will be receiving active treatment for up to 9 months, regardless of their treatment assignment in Study 2553, and pilot subjects who complete these 9 months will be eligible for up to a further 80 weeks of treatment (the Pilot Extension). Pilot subjects completing Week e2-80 and Primary Study Stage subjects completing Week e40 will be eligible to enrol in a further extension phase to this study (the "Supplemental Extension") and receive ongoing 4-weekly GDNF infusions until the end of December 2016. Hence, the study will provide access to continued treatment for those subjects who received active treatment during Study 2553 and provide access to active treatment for those subjects who received placebo in Study 2553. However, individual treatment codes in Study 2553 will not be disclosed to subjects until database lock for Study 2797. Also, every effort will be made to avoid unblinding of the blinded UPDRS raters until database lock for Study 2797.

The study is designed to assess the effects on efficacy and safety of extended (up to 18 months total) intermittent intraputamenal infusion of GDNF, using similar endpoints as Study 2553. In addition, the study is expected to provide initial insight into the question of whether the effect on motor outcome at 18 months is improved with a longer duration of treatment (9 vs. 18 months). Additional long-term safety data will be obtained from the Pilot and Supplemental Extension parts of this study.

4 OBJECTIVES

4.1 Primary Objective

To compare the effects of intermittent bilateral intraputamenal GDNF infusions on OFF-state motor function after 18 months of study treatment with the effects after 9 months of treatment in subjects who completed Study 2553.

4.2 Secondary Objectives

- To compare the effects of intermittent bilateral intraputamenal GDNF infusions on ON-state motor function, motor complications, and ON- and OFF-state activities of daily living (ADL) after 18 months of treatment with the effects after 9 months of treatment in subjects who completed Study 2553.
- To assess the safety of intermittent bilateral intraputamenal GDNF infusions at 18 months in subjects who received GDNF or placebo for 9 months in Study 2553.

4.3 Other Objectives

- To explore the effects of intermittent bilateral intraputamenal GDNF infusions on other motor and non-motor functions, quality of life assessments and imaging endpoints at 18 months in subjects who completed Study 2553.
- To compare the results for various motor outcomes between the subjects who started GDNF early (i.e. were randomized to GDNF in Study 2553) and those who started GDNF late (i.e. were randomized to placebo in Study 2553).
- Pilot and Supplemental Extensions: To generate long-term safety data and provide continued access to GDNF until the end of December 2016 when the results of Study 2553 are expected, which will inform interested parties with potential future studies.

5 TRIAL DESIGN

5.1 Summary of Trial Design

5.1.1 Overview

This is a phase II, single-centre, open-label trial, in subjects with idiopathic PD who have completed Study 2553 of intermittent bilateral posterior putamen GDNF infusions administered via CED. The study is planned to enrol a total of up to 42 subjects.

Following the final study visit at Week 40 in Study 2553, study completers will return within one week to receive their first infusion of open-label GDNF. Treatment will be continued at 4-week intervals for 9 months (40 weeks; 10 infusions total). Hence, at 18 months, subjects receiving GDNF in Study 2553 will have been treated with GDNF for a total of 18 months, while those receiving placebo in Study 2553 will have been treated with GDNF for a total of 9 months.

Pilot subjects who complete this 9-month extension study and provide informed consent will be eligible for up to an additional 80 weeks of treatment with GDNF.

Pilot subjects completing Week e2-80 and Primary Study Stage subjects completing Week e40 who provide informed consent will be eligible to enrol in a further extension phase to this study (the "Supplemental Extension") and receive ongoing 4-weekly GDNF infusions until the end of December 2016.

Figure 1 presents the study schema.

The planned study period, including recruitment, is approximately 42 months. Although the primary statistical assessment of Study 2553 will have been performed before the extension study is complete, to reduce any potential for bias in this study, individual treatment codes from the parent study will not be disclosed to subjects until database lock for Study 2797, unless required for specific safety reasons. In addition, every effort will be made to avoid unblinding of the blinded UPDRS raters until database lock for Study 2797.

As reflected in protocol amendments no. 3 and 4, the delivery technique and algorithm employed in the Primary Stage of Study 2553 was appreciably different from the Pilot Stage. Therefore, the primary analysis population of both studies only includes subjects randomised during the Primary Study Stage. However, secondary efficacy analyses

will include subjects randomised in the Pilot Stage as well. Safety will be analysed for the Pilot and Primary Study Stages separately and for all subjects and both stages combined.

Figure 1 Study Schema

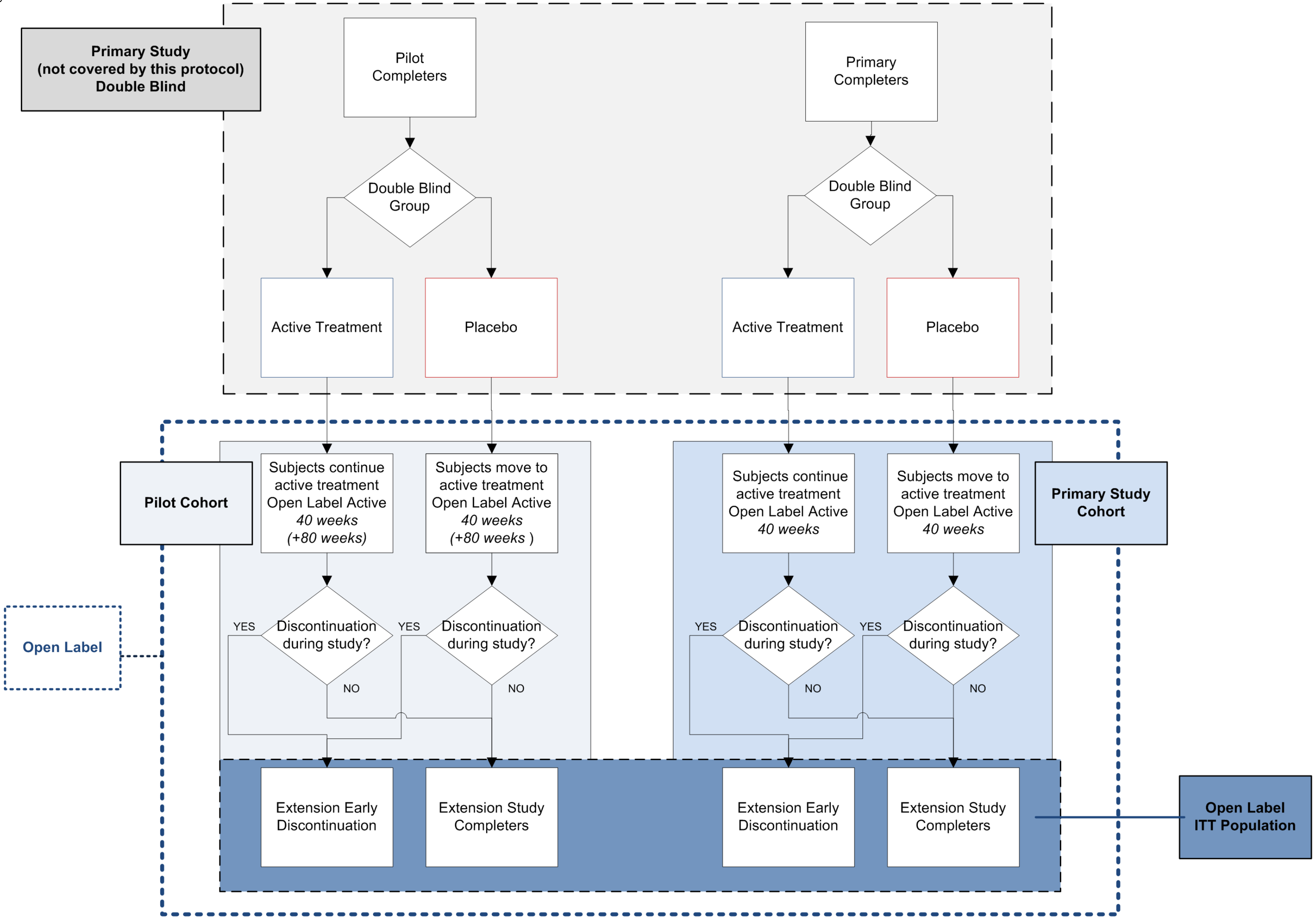

Note: The schema does not include the Supplemental Extension part of the study.

5.1.2 Post Study Access to Treatment

What happens at study end? For subjects who reach the end of the current protocol, the following information applies which is taken directly from the Patient Information Sheet but is given here in order that the information provided to study subjects is also directly included in the protocol:

No one yet knows what will happen to the supply of GDNF or continuance of infusions once the extension study ends. Numerous factors will need to be in place for GDNF infusions to continue and none of these can be guaranteed. These include that the company that holds the license for GDNF will continue to produce the protein, that the regulatory bodies governing administration of drug therapies in the UK will allow us to continue administering, and that funding and capacity to continue either purchasing and/or administering GDNF is available.

The hope is that if the double-blind study shows positive benefits across the population of patients assessed, without side effects that prevent continuation, infusions may continue post the end of the extension study. This will enable us to continue to collect benefit and safety data. However, that this occurs can in no way be guaranteed. As discussed with participants prior to enrolling in the double-blind study GDNF infusions may very well stop post either the double-blind study or the extension study, even if participants have received great benefit. In terms of GDNF being developed as a routine therapy, the next step following the extension study (but only if the double-blind study is successful) will be a phase III trial, where presumably more than 100 patients will be enrolled and treated at multiple centres around the world. Only after such a multi-centre trial which may take several years, followed by a period of further regulatory approvals, is there the potential for GDNF therapy to be routinely available.

Hence receiving GDNF may well stop at the end of this extension study even if participants individually have received benefit from the infusions.

At the end of this trial, if it is found that there is no statistical level benefit to the participants as a whole from receiving GDNF it is unlikely that this therapy will continue to be available to subjects even if they as individuals feel or are better for it.

If this trial is statistically positive we still cannot guarantee that GDNF will be available, but if GDNF becomes available through normal commissioning processes,

the subjects' clinicians will review how appropriate subjects are to receive this treatment.

If GDNF is stopped or not available, the subjects' doctors will ensure that adequate consideration is given to the protection of subjects' interests and ongoing medical needs. This point will be discussed with subjects further prior to obtaining their consent to participate.

If during this trial GDNF becomes no longer available, it is anticipated that the port system behind the ear will be removed and closed. However, the remaining tubing inside the brain etc. may be left in place if this is deemed to be a safer or better option post discussion with the medical team.

5.2 Study Endpoints

The analysis of the primary and secondary endpoints will be restricted to the up to 36 subjects randomised during the Primary Stage in Study 2553. Subjects treated during the Pilot Stage in Study 2553 will, however, be included in the analysis of safety and supplementary efficacy analyses.

5.2.1 Primary Endpoint

The primary endpoint of the study is the percentage change from baseline to the end of treatment in the practically defined OFF-state UPDRS motor score (part III).

5.2.2 Secondary Endpoints

- Percentage change from baseline to the end of treatment in UPDRS motor score (part III) in the ON-state (following a levodopa challenge)
- Percentage change from baseline to the end of treatment in UPDRS ADL (part II) .
- Change from baseline to the end of treatment in PD diary ratings; i.e., total OFF-time per day, total good quality ON-time (ON without dyskinesias or ON with non-troublesome dyskinesias) and ON-time with troublesome dyskinesias.

5.2.3 Supplementary Efficacy Endpoints

Change from baseline to the end of treatment in PD non motor assessments of cognition, executive function, mood, other non-motor symptoms, smell, appetite, medication equivalents, and quality of life.

Analysis of the primary and secondary endpoints will be repeated for the overall ITT population including subjects randomised in the Pilot Stage.

5.2.4 Imaging Endpoints

Volume of distribution of infusate will be determined both by contrast-enhanced T1-weighted and by T2-weighted and fluid-attenuated inversion recovery (FLAIR) 3T MRI. Scans are to be completed within 2 hours of delivering a gadolinium contrast-containing test infusion of diluent at Week e40 in the extension study (Primary Study Stage subjects only) and at Weeks e2-0 and e2-80 in the Pilot Extension. GDNF will not be delivered at any of these visits, and no interim MRI scans will be done during the study unless clinically indicated.

5.2.5 Safety Endpoints

- Incidence of device-related adverse events (AEs) during the study period.
- Incidence of treatment-emergent AEs (related and unrelated) during the study period.
- Time with troublesome dyskinesias (from subject diaries).
- Clinical assessment of safety, including dyskinesias and falls, mood and impulsivity at each infusion visit.
- The Questionnaire for Impulsive-Compulsive Disorders in Parkinson's Disease (QUIP) every 8 weeks.
- Changes in the Montreal Cognitive Assessment (MoCA) and Mattis Dementia Rating Scale (MDRS) at the end of the study.
- Adverse changes in magnetic resonance imaging (MRI) findings.
- Anti-GDNF antibody samples taken at intervals during the study will be assessed at the end of the study.

- Routine laboratory blood tests (haematology, serum chemistry) and urinalysis performed at intervals during the trial.
- Full-brain MRI.

5.3 Trial Participants

5.3.1 Overall Description of Trial Participants

Subjects with idiopathic PD with motor fluctuations who complete Study 2553.

5.3.2 Inclusion Criteria

In order to qualify for entry into the study, subjects MUST meet all of the following criteria:

1. Enrolled and completed treatment in the Pilot or Primary Study Stages of Study 2553.
2. Females of childbearing potential must have a negative pregnancy test at study entry and be willing to use an approved (by the PI or designee) form of contraception until the end of the study.
3. Males with female partners of childbearing potential must be willing to use condoms for contraception until the end of the study.
4. Provision of informed consent.

5.3.3 Exclusion Criteria

Subjects who meet any of the following criteria will NOT be eligible for inclusion in the study:

1. Discontinued treatment early in Study 2553.
2. Had any significant (in the opinion of the PI or designee) protocol deviation in Study 2553; this includes receipt of any disallowed anti-parkinsonian treatment or any investigational treatment.
3. Presence of clinically significant (in the opinion of the PI) depression.
4. MoCA score < 24 at the final assessment in Study 2553.

5. Any new medical condition which might impair outcome measure assessments or safety measures including ability to undergo MRI scanning.

5.4 Expenses and Benefits

Reasonable travel expenses for any visits additional to normal care will be reimbursed on production of receipts or a mileage allowance provided as appropriate.

5.5 Study Procedures

Subjects may enrol in this study immediately upon completion of Study 2553 provided they meet the eligibility criteria. Procedures and assessments performed at Week 40 of the parent study will be used as screening assessments for this study. The count of study weeks will be reset and numbers will be prefixed with an "e" (e.g., "Week e0") to differentiate them from the numbering in Study 2553.

The first infusion in the extension study will be performed at the Week e0 visit which is to occur within one week of the Week 40 assessment in Study 2553 so as to keep the interval between the last treatment in the parent study and the first treatment in the extension study to a maximum of 5 weeks. Beginning at Week e0, GDNF infusions will be administered every 4 weeks for 9 months; the total dose of GDNF administered at any given infusion remains unchanged from Study 2553 at 120 µg (600 µL of 0.20 µg/µL GDNF) per putamen.

Volumetric catheter performance will be determined by means of both gadolinium contrast-enhanced T1-weighted and T2-weighted and FLAIR 3T MRI scans at Week e40 in the extension study (Primary Study Stage subjects only) and at Weeks e2-0 and e2-80 in the Pilot Extension. Key clinical outcome measures will be performed at 8-week intervals throughout the study. Additional clinical outcome measures will be assessed at visit e40.

5.5.1 Informed Consent

The subject must personally sign and date the latest approved version of the informed consent form before any study specific procedures and assessments are performed (i.e., before Week e0 procedures for this study are performed). Week 40 procedures for Study 2553, which may be used as screening for this study, may be performed before consent for the extension study is obtained.

Written and verbal versions of the participant information and informed consent will be presented to the potential participants detailing no less than: the experimental nature of the study; the implications and constraints of the protocol; the known side effects and any risks involved in taking part. It will be clearly stated that any participant is free to withdraw from the study at any time for any reason without prejudice to future care and with no obligation to give the reason for withdrawal.

The above information will be provided to the potential participants at the 36-Week visit or earlier in the parent study, so as to allow ample time to consider the information and provide the opportunity to question the PI or designee, their general practitioner, or other independent parties to decide whether they will participate in the study. Written informed consent will then be obtained by means of participant dated signature and dated signature of the person who presented and obtained the informed consent. The person who obtained the consent must be suitably qualified and experienced and have been authorised to do so by the PI. A copy of the signed informed consent will be given to the participant. The original signed form will be retained at the study site, and a copy will be filed in the medical notes.

Subjects who enrol in the Pilot and Supplemental Extensions will be required to sign and date an additional informed consent form prior to initiation of any Pilot or Supplemental Extension procedures.

5.5.2 Screening and Eligibility Assessment

Screening and eligibility assessments for this study will be the assessments performed as part of the Week 40 assessments for Study 2553; aside from informed consent, no additional screening procedures are required for this study.

5.5.3 Preparation of Study Drug

All subjects will receive active GDNF in this extension study.

Study drug will be prepared by a pharmacist at the study site. Ready to use preparations of GDNF will be provided to the PI or designee. Drug preparation guidelines are provided in a separate pharmacy manual.

5.6 Schedule of Treatments and Assessments

Upon receipt of study drug as described above, the PI or designee will initiate a 40-week (approximately 9 months) course of q4 weekly intraputamenal administration of GDNF via CED. The full course will comprise 11 scheduled visits (Week e0 and 10 q4 weekly visits through Week e40). Except for the final visit where no GDNF infusion will be given, at each visit GDNF will be administered, followed by clinical assessment of safety, including dyskinesias and falls, mood and impulsivity. If necessary, visits may take place over more than 1 day, for example when study drug infusion is planned for early morning.

AEs, concomitant medications and vital signs will be recorded at all visits. PD assessments and QUIP will be performed every 8 weeks, and full assessments of safety and PD variables will be performed at the final assessment (Week e40).

At Week e40, Primary Study Stage subjects will receive a gadolinium contrast-containing test infusion of diluent, followed both by T1-weighted and by T2-weighted and FLAIR MRI scans.

5.6.1 Treatments

All subjects will receive open-label active treatment (GDNF) every 4 weeks, but subjects will remain blinded to the treatment codes of individual subjects during Study 2553 until database lock for Study 2797 unless unblinding is required for specific safety reasons.

Subjects will return to the clinic for treatments within one week after the completion of all study measures from the Week 40 visit for Study 2553 for their Week e0 visit of the extension study and then every 4 weeks (± 3 days) thereafter at Weeks e4, e8, e12, e16, e20, e24, e28, e32 and e36. If necessary, a treatment may be given a maximum of + 7 days from the scheduled date; if the treatment cannot be given within + 7 days, it will be considered missed and the treatments will resume with the following scheduled date. Before each PD outcome measure assessment (Weeks e8, e16, e24 and e32) that is to be taken (detailed in Section 5.6.2), subjects will withhold all PD medications for assessment in the OFF state. For all other treatments, subjects will take their usual medications on the study treatment day and will report to the clinic irrespective of their ON/OFF state.

At every treatment, the following procedures and assessments will be performed before treatment:

- Vital signs: Seated systolic and diastolic blood pressure (BP), heart rate (HR), respiration rate (RR) and temperature will be assessed after the subject has been sitting quietly for at least 5 minutes. Standing systolic and diastolic BP and HR will also be assessed after the subject has been standing for 3 minutes.
- Any changes to concomitant medications from the previous visit will be recorded. All over-the-counter or prescription medications, vitamins, and/or herbal supplements will be recorded in the case report form (CRF) with their indications.
- Any AEs observed or reported since the previous visit will be recorded.

After completion of the pretreatment procedures and assessments, the PI or designee will administer the drug over a period of approximately 90 to 150 minutes using pre-programmed micro-infusion pumps.

Subjects will be carefully observed for safety during and after study drug administration. During administration, seated vital signs (systolic and diastolic BP, HR and RR) will be collected every 15 (Week e0) to 30 (Weeks e4 to e36) minutes, and both seated and standing systolic and diastolic BP and HR will be assessed within 30 minutes after the end of the infusion. A brief neurological screen (Glasgow Coma Scale) will also be done before infusion, 30 minutes into the infusion, and after completion of the infusion.

Subjects will remain at the facility for safety observation for at least 2 to 4 hours after completing study drug administration at Week e0, and 1 to 2 hours after completing study drug administration at all subsequent time points.

The last study treatment will be administered at Week e36.

At the treatment before each Interim Assessment and at the last Treatment (i.e., at Weeks e4, e12, e20, e28 and e36), subjects will be issued PD fluctuation diaries (Section 5.9.1.4) to complete at home before the next assessment. The subject will be instructed to complete diaries for 3 consecutive predetermined days in the week prior to the next study treatment. The day immediately prior to the study treatment is NOT to be used for diary recording.

5.6.2 Interim Assessments

Subjects will undergo extended safety and efficacy assessments every 8 weeks at Weeks e8, e16, e24 and e32.

For these assessments, subjects will withhold all PD medications as follows and report to the clinic in the OFF state:

- No PD medications will be taken after 6:00 PM on the night before the assessments and
- No long-acting PD medications will be taken on the day before the assessments.

Subjects will refrain from eating any high-protein foods on the morning of the assessments.

Interim Assessment visits will also be Treatment visits, and procedures and assessments will include those listed in Section 5.6.1 for Treatments. However, the following assessments will also be performed pretreatment:

At the beginning of the assessment visit, all OFF period measures are to be completed first:

- UPDRS part II (ADL) and part III (motor) in OFF state (Section 5.9.1.1)
- Timed walking test (Section 5.9.1.2)
- Timed tapping test (Section 5.9.1.3)

A levodopa challenge will then be performed (refer to site-specific general guidelines) and when the subject goes ON the following post-challenge tests will be performed:

- Full UPDRS (including parts I, II, III and IV; Section 5.9.1.1)
- Timed walking test (Section 5.9.1.2)
- Timed tapping test (Section 5.9.1.3)
- MDRS (Section 5.9.2.2)/MoCA (Section 5.9.2.1) (Week e16 only)
- Deary-Liewald reaction time (Section 5.9.2.4) (Week e16 only)
- QUIP (Section 5.9.2.6)

Additional study procedures which can be performed irrespective of ON/OFF state include:

- Collection of subject PD fluctuation diaries (Section 5.9.1.4)
- Weight and height

5.6.3 Laboratory and Other Assessments

The following laboratory procedures will be performed at Weeks e4, e16 and e28:

- Clinical laboratory tests, including haematology (haematocrit, haemoglobin, mean cellular haemoglobin (MCH), mean cellular haemoglobin concentration (MCHC), mean cellular volume (MCV), platelet count, red blood cell (RBC) count, white blood cell (WBC) count and WBC differential [basophils, eosinophils, lymphocytes, monocytes and neutrophils]), serum chemistry (albumin, alkaline phosphatase, alanine transaminase (ALT), creatinine, estimated glomerular filtration rate (eGFR), glucose, potassium, sodium, total bilirubin and urea) and urinalysis (colour, appearance, pH, glucose, ketones and nitrite; microscopy only required if indicated to follow up abnormal findings). A pregnancy test will be performed for all women of childbearing potential.
- Blood samples for anti-GDNF antibodies and plasma GDNF levels. The samples will be stored at -70°C until central analysis after all subjects have completed the study.

Subjects will undergo Non-Motor Symptom Assessment Scale for PD (NMSS) (Section 5.9.1.5) at Weeks e12 and e24.

5.6.4 Week e40 or Early Discontinuation

Week e40 will mark the completion of the extension study. A full safety and efficacy assessment will be performed, and the same assessments should be performed, if possible, for any subjects who discontinue the study before Week e40. The Week e40 visit must occur ±3 days of the scheduled date and may be completed over more than 1 day due to the number of assessments.

For this assessment, subjects will withhold all PD medications as follows:

- No PD medications will be taken after 6:00 PM on the night before the assessments and
- No long-acting PD medications will be taken on the day before the assessments.

Subjects will refrain from eating any high-protein foods on the morning of the assessments.

At the beginning of the assessment, all OFF period measures are to be completed first:

- UPDRS part II (ADL) and part III (motor) in OFF state (Section 5.9.1.1)
- Timed walking test (Section 5.9.1.2)
- Timed tapping test (Section 5.9.1.3)

A levodopa challenge will then be performed (refer to site-specific general guidelines) and when the subject goes ON the following post-challenge tests will be performed:

- Full UPDRS (including parts I, II, III and IV; Section 5.9.1.1)
- Timed walking (Section 5.9.1.2)
- Timed tapping test (Section 5.9.1.3)
- MDRS (Section 5.9.2.2)/MoCA (Section 5.9.2.1)
- Stroop (Section 5.9.2.3)
- Other brief measures of cognitive and executive function (Section 5.9.2.4)
- BDI (Section 5.9.2.4)
- PD NMSS (Section 5.9.1.5)
- Parkinson's Disease Questionnaire-39 (PDQ-39; Section 5.9.1.6)
- EuroQOL 5-dimensional scale (EQ-5D; Section 5.9.1.6)
- QUIP (Section 5.9.2.6)

The following procedures may be completed irrespective of the subject's ON/OFF status:

- University of Pennsylvania Smell Identification Test (UPSIT; Section 5.9.2.7)
- Collection of subject PD fluctuation diaries (Section 5.9.1.4)

- Weight and height
- Vital signs: Seated systolic and diastolic BP, HR, RR and temperature will be assessed after the subject has been sitting quietly for at least 5 minutes. Standing systolic and diastolic BP and HR will also be assessed after the subject has been standing for 3 minutes.
- Brief physical examination, targeted, at the investigators discretion, to identify any notable changes from baseline.
- Standard 12-lead electrocardiogram (ECG). At least the following ECG parameters will be recorded: HR, PR, QT, QRS and corrected QT (QTc) intervals. The report will be signed by the PI or designee and it will be recorded in the CRF whether it is normal, abnormal but not clinically significant, or abnormal AND clinically significant (together with details of the abnormality).
- Clinical laboratory tests, including haematology (haematocrit, haemoglobin, MCH, MCHC, MCV, platelet count, RBC, WBC count and WBC differential [basophils, eosinophils, lymphocytes, monocytes and neutrophils]), serum chemistry (albumin, alkaline phosphatase, ALT, creatinine, eGFR, glucose, potassium, sodium, total bilirubin and urea) and urinalysis (colour, appearance, pH, glucose, ketones and nitrite; microscopy only required if indicated to follow up abnormal findings). A pregnancy test will be performed for all women of childbearing potential.
- Blood samples for anti-GDNF antibodies and plasma GDNF levels. The samples will be stored at -70°C until central analysis after all subjects have completed the study.
- Any changes to concomitant medications from the previous visit will be recorded. All over-the-counter or prescription medications, vitamins, and/or herbal supplements will be recorded in the CRF with their indications.
- Any AEs observed or reported since the previous visit will be recorded.

At any time within a 2-week window around Week e40, but preferably 1 week before Week e40 if possible, Primary Study Stage subjects will receive a gadolinium contrast-containing test infusion of diluent (using the infusion algorithm for study medication

detailed in Section 6.3), followed both by T1-weighted and by T2-weighted and FLAIR MRI scans. Subjects will be carefully observed for safety during and after administration of the test infusion. During administration, seated vital signs (systolic and diastolic BP, HR and RR) will be collected every 30 minutes, and both seated and standing systolic and diastolic BP and HR will be assessed within 30 minutes after the end of the infusion. A brief neurological screen (Glasgow Coma Scale) will also be done before infusion, 30 minutes into the infusion, and after completion of the infusion.

5.7 Pilot Extension

Pilot subjects completing visit e40 who in the opinion of the Principal Investigator (PI) have no contraindication to continued GDNF infusions and sign informed consent are eligible to enrol in an extension phase to this study and receive ongoing 4 weekly GDNF infusions for up to 80 additional weeks.

5.7.1 Study Procedures

Pilot subjects may enrol in this study immediately upon completion of visit e40. Procedures and assessments performed at Week e40 will be used as baseline assessments for this study. The count of study weeks will be reset and numbers will be prefixed with an "e2" (e.g., "Week e2-0") to differentiate them from the numbering in the extension study.

Week e2-0 is to occur within one week of the Week e40 assessment so as to keep the interval between the last treatment in the extension study and the first treatment in the Pilot Extension to a minimum as no GDNF infusion will be administered at Week e2-0. Instead, a gadolinium contrast-containing test infusion of diluent will be given (using the infusion algorithm for study medication detailed in Section 6.3), followed both by T1-weighted and by T2-weighted and FLAIR MRI scans.

The first GDNF infusion in the Pilot Extension will be performed at Week e2-4 and GDNF infusions will be administered every 4 weeks for a maximum of 19 infusions; the total dose of GDNF administered at any given infusion will remain unchanged from the extension study at 120 μg per putamen.

Volumetric catheter performance will again be determined by means of both contrast-enhanced T1-weighted and T2-weighted and FLAIR 3T MRI scans within 2 hours of

delivering a gadolinium contrast-containing test infusion of diluent at Week e2-80. Key clinical outcome measures will be performed at Weeks e2-16, e2-32, e2-56 and e2-80. Additional clinical outcome measures will be assessed at Week e2-80.

5.7.2 Treatments

Pilot subjects will receive open-label active treatment (GDNF) every 4 weeks, but subjects will remain blinded to the treatment codes of individual subjects during Study 2553 until database lock for Study 2797 unless unblinding is required for specific safety reasons.

Subjects will return to the clinic for infusions at Week e2-0 within one week after the completion of all study measures from the Week e40 visit and then every 4 weeks (± 3 days) thereafter. At all visits from Week e2-4 through e2-76, open-label treatment with GDNF will be administered. At Weeks e2-0 and e2-80, a gadolinium contrast-containing test infusion of diluent will be given instead of GDNF.

Before each PD outcome measure assessment (Weeks e2-16, e2-32, and e2-56), subjects will withhold all PD medications for assessment in the OFF state (as detailed in Section 5.6.2). For all other treatments, subjects will take their usual medications on the study treatment day and will report to the clinic irrespective of their ON/OFF state.

At every treatment visit, the procedures and assessments will be performed as outlined above in Section 5.6.1 for the extension study with the exception of the timing of diary distribution. This will occur at Weeks e2-12, e2-28, e2-52 and e2-76.

5.7.3 Interim Assessments

Subjects will undergo extended safety and efficacy assessments at Weeks e2-16, e2-32 and e2-56.

For these assessments, subjects will withhold all PD medications and report to the clinic in the OFF state as detailed in Section 5.6.2, and all assessments and procedures from Section 5.6.2 will be performed with the exception of the MDRS, MoCA and Deary-Liewald reaction time. The QUIP will be administered at Weeks e2-16, e2-32, e2-48 and e2-64. Weight and height will be measured at Weeks e2-24 and e2-56.

5.7.4 Other Assessments

Subjects will undergo Non-Motor Symptom Assessment Scale for PD (NMSS) (Section 5.9.1.5) at Week e2-40.

Blood samples for anti-GDNF antibodies and plasma GDNF levels will be collected at Weeks e2-24, e2-48 and e2-72. The samples will be stored at -70°C until central analysis after all subjects have completed the extension study.

5.7.5 Week e2-80 or Early Discontinuation

Week e2-80 will mark the completion of the Pilot Extension. A full set of safety and efficacy assessments will be performed, and the same assessments should be performed, if possible, for any subjects who discontinue the study before Week e2-80. The Week e2-80 visit must occur ±3 days of the scheduled date and may be completed over more than 1 day due to the number of assessments.

For this assessment, subjects will withhold all PD medications and follow the procedures of the Week e40 visit as described in Section 5.6.4, except that the quality of life and most cognitive measures will not be performed.

At any time within a 2-week window around Week e2-80 but preferably 1 week before Week e2-80 if possible, subjects will receive a gadolinium contrast-containing test infusion of diluent, followed both by T1-weighted and by T2-weighted and FLAIR MRI scans.

5.8 Supplemental Extension

Pilot Stage subjects completing Week e2-80 and Primary Study Stage subjects completing Week e40 who, in the opinion of the Principal Investigator (PI), have no contraindication to continued GDNF infusions and sign informed consent are eligible to enrol in a further extension phase to this study (the "Supplemental Extension") and receive ongoing 4 weekly GDNF infusions until the end of December 2016. Only subjects who can have at least one visit by the end of December 2016 will be enrolled in the Supplemental Extension.

5.8.1 Study Procedures

Subjects may enrol in the Supplemental Extension immediately upon completion of Week e2-80 (Pilot Stage subjects) or Week e40 (Primary Study Stage subjects). Procedures and assessments performed at Week e2-80 (Pilot Stage subjects) or Week e40 (Primary Study Stage subjects) will be used as baseline assessments for this extension. The count of study weeks will be reset and numbers will be prefixed with an "e3" (e.g., "Week e3-0") to differentiate them from the numbering in the previous extensions.

Week e3-0 is to occur approximately 1-2 weeks after the Week e2-80 or Week e40 visit so as to keep the interval between the last treatment in the previous extension and the first treatment in the Supplemental Extension to a minimum while retaining a minimum interval of 2 weeks from the end-of-study test infusion in the previous extension.

The first GDNF infusion in the Supplemental Extension will be performed at Week e3-0 and GDNF infusions will be administered every 4 weeks until the end of December 2016 for a maximum of 13 infusions; the total dose of GDNF administered at any given infusion will remain unchanged from the previous extension at 120 µg per putamen.

Key clinical outcome measures will be performed at Weeks e3-16 and e3-32 (if applicable). A small panel of safety assessments will be performed at the last study visit.

5.8.2 Treatments

Subjects will receive open-label active treatment (GDNF) every 4 weeks, but subjects will remain blinded to the treatment codes of individual subjects during Study 2553 until database lock for Study 2797 unless unblinding is required for specific safety reasons.

Subjects will return to the clinic for infusions at Week e3-0 approximately 1-2 weeks after the completion of all study measures from the Week e2-80 or Week e40 visit (i.e. 2-3 weeks after the administration of the end-of-study test infusion prior to these visits) and then every 4 weeks (± 3 days) thereafter.

Before each PD outcome measure assessment (Weeks e3-16 and e3-32), subjects will withhold all PD medications for assessment in the OFF state (as detailed in Section 5.6.2). For all other treatments, subjects will take their usual medications on the study treatment day and will report to the clinic irrespective of their ON/OFF state.

At every treatment visit, the procedures and assessments will be performed as outlined above in Section 5.6.1 for the extension study with the exception of the timing of diary distribution. This will occur at Weeks e3-12 and e3-28.

5.8.3 Interim Assessments

Subjects will undergo extended safety and efficacy assessments at Weeks e3-16 and e3-32.

For these assessments, subjects will withhold all PD medications and report to the clinic in the OFF state as detailed in Section 5.6.2, and all assessments and procedures from Section 5.6.2 will be performed with the exception of the MDRS, MoCA, Deary-Liewald reaction time, and weight and height.

5.8.4 Study Completion or Early Discontinuation

Subjects will continue in the Supplemental Extension until the last study visit in December 2016 (which will occur at Week e3-48 or earlier).

At this time, the subject will undergo all procedures and assessments scheduled for the respective visit reached by the subject as per the visit schedule. In addition, regardless of the assessments scheduled for the respective visit, MoCA, MDRS, QUIP and BDI assessments will be performed and samples will be obtained for laboratory assessment and determination of anti-GDNF antibody levels and GDNF plasma concentrations.

The same approach to final assessments should be taken, if possible, for any subjects who discontinue the Supplemental Extension early.

5.9 Outcomes Measures

5.9.1 Efficacy Outcome Measures

5.9.1.1 Unified Parkinson's Disease Rating Scale

The UPDRS (Appendix F) was developed as an outcome measure for rating PD in clinical trials. It has 4 parts which predominantly measure the motor signs and symptoms of PD: Mentation, Behaviour and Mood (part I), ADLs (part II), Motor Examination (part III), and Complications of Therapy (part IV). Except for the motor examination which is performed at the assessment, all sections of the UPDRS rate the subject based on his/her state in the week preceding the assessment. Higher scores represent worse functioning.

In this trial all UPDRS ratings will be performed by a trained rater who is blinded to all other aspects of the subject's condition. The motor UPDRS will be performed with the subject in a practically defined OFF state (at least 12 hours since last PD medication) and in the ON state after levodopa challenge. Every effort will be made to avoid unblinding of the raters until database lock for Study 2797.

The primary efficacy outcome for this study will be the percentage change in motor UPDRS in the practically defined OFF state between baseline (i.e., before beginning treatment in Study 2553) and Week e40. Other UPDRS endpoints include UPDRS part II in both the ON and OFF states, UPDRS part III in the ON state after levodopa challenge and total UPDRS scores. ON and OFF UPDRS ratings will be done at Weeks e8, e16, e24, e32 and e40.

ON and OFF UPDRS ratings will be done in the Pilot Extension at Weeks e2-16, e2-32, e2-56 and e2-80 and in the Supplemental Extension at Weeks e3-16 and e3-32.

5.9.1.2 Timed Walking Test

During the timed walking test, the subject will walk as fast as possible 7 metres back and forth including turning. The time to perform this test is recorded. The subject will complete this test twice during the practically defined OFF period and twice in the ON state after levodopa challenge. The ratings will be performed at Weeks e8, e16, e24, e32 and e40.

Ratings will be done in the Pilot Extension at Weeks e2-16, e2-32, e2-56 and e2-80 and in the Supplemental Extension at Weeks e3-16 and e3-32.

5.9.1.3 Timed Tapping Test

During the timed tapping test, the subject is instructed to alternate tapping the index finger between 2 points spaced 30 cm apart. Each hand is rated twice and the number of taps completed in 20 seconds on each side is to be recorded. This test is to be performed in the practically defined OFF state and in the ON state after levodopa challenge at Weeks e8, e16, e24, e32 and e40.

Ratings will be done in the Pilot Extension at Weeks e2-16, e2-32, e2-56 and e2-80 and in the Supplemental Extension at Weeks e3-16 and e3-32.

5.9.1.4 Subject Diaries

Motor fluctuations in this study will be quantified using subject completed diaries (Appendix G). For 3 days prior to the relevant study assessments (not including the day immediately prior to the assessment, as the subject will be asked to withhold PD medication on that day), subjects are to record their state for every half hour time period. Categories for rating include: ON with no dyskinesias, ON with non-troublesome dyskinesias, ON with troublesome dyskinesias, OFF or asleep. Caregivers may assist with the physical completion of the diary, however, the decision regarding the subject's state is to be made by the subject alone.

During the parent study, subjects will have been trained on the completion of the diary and will have demonstrated their ability to accurately determine their state by comparing their own assessments to those of a qualified staff member over a 2-4 hour period.

Duration of good ON time (ON without dyskinesias and ON with non-troublesome dyskinesias) and OFF time will be secondary efficacy variables in this study. Duration of ON with troublesome dyskinesias will be a safety outcome for this study.

Diaries will be collected at Weeks e8, e16, e24, e32 and e40. Diaries are to be reviewed by the coordinator each time they are returned, and retraining should be offered if errors in completion are noted. Diaries are to be dispensed at the visit prior to their collection.

Diaries will be collected at Weeks e2-16, e2-32, e2-56 and e2-80 in the Pilot Extension and at Weeks e3-16 and e3-32 in the Supplemental Extension.

5.9.1.5 Non-Motor Symptom Scale

The NMSS (Appendix H) is an interview based scale developed to rate non-motor symptoms commonly occurring in PD. The 30 item scale rates symptoms which occurred in the preceding month in 9 domains – cardiovascular function including falls, sleep/fatigue, mood/cognition, perceptual problems/hallucinations, attention/memory, gastrointestinal, urinary, sexual function, and miscellaneous. Each item is rated from 0 (none) – 3 (severe) for severity and from 1 (rarely) to 4 (very frequent) and the score for each item is the product of the severity rating multiplied by the frequency.

The NMSS takes 20-30 minutes and is to be completed by the PI or designee when the subject is in the ON state. This scale is administered at Weeks e12, e24 and e40.

The NMSS will be administered at Weeks e2-40 and e2-80 in the Pilot Extension, but will not be administered in the Supplemental Extension.

5.9.1.6 PDQ-39/EQ-5D

The PDQ-39 (Appendix I) and the EQ-5D (Appendix I) are both subject self-report measures of quality of life. The PDQ-39 is a PD-specific quality of life tool with 39 questions. The EQ-5D is a generic quality of life scale with 5 questions and a health "thermometer." These scales will be completed when the subject is in the ON state during their visit. The subject may receive assistance with the physical completion of the scales but the subject alone must determine the answer provided. The scales will take approximately 20 minutes for the subject to complete. The PDQ-39 and EQ-5D are completed at the end of the extension study (Week e40).

These measures will not be performed in the Pilot Extension or the Supplemental Extension.

5.9.2 Safety Outcome Measures

5.9.2.1 Montreal Cognitive Assessment

The MoCA (Appendix C) is a cognitive screening tool which assesses both cortical and subcortical function. It has 8 components – visuospatial/executive, naming, memory,

attention, language, abstraction, delayed recall and orientation, and the total score ranges from 0 – 30 with lower scores representing poorer cognitive function. It takes approximately 30 minutes to administer and will be performed when the subject is in the ON state at Week e16 and at the end of the extension study (Week e40).

This test will be performed at Week e2-80 in the Pilot Extension and at the last visit in the Supplemental Extension.

Any trained site personnel may complete this scale. Subjects must score a minimum of 24 on the MoCA at the end of Study 2553 to be eligible for the study.

Learning effects are always of high importance in investigations such as this. However, it is planned to use parallel versions of the MoCA to minimize the impact of learning.

5.9.2.2 Mattis Dementia Rating Scale (MDRS)

The MDRS is a global scale of cognition that is sensitive to the frontal/subcortical deficits that are common in PD. It includes 5 subscales – attention, initiation/ perseveration, construction, conceptualization and memory. Scores range from 0 – 144 with higher scores representing better cognitive function. In PD, scores < 123 are associated with some degree of dementia. The MDRS can be analysed using total scores or using individual subscale scores.

The MDRS must be administered by a trained rater with the subject in the ON state and takes 30 – 45 minutes to complete. It will be performed at the end the extension study (Week e40).

This test will be performed at Week e2-80 in the Pilot Extension and at the last visit in the Supplemental Extension.

5.9.2.3 Stroop

The Stroop is a cognitive test that measures the capacity to direct attention. In this test subjects are presented with words that are the names of colours but are printed in a different colour of ink than the name represents (e.g., "green" printed in red). In the first component of the test, the subject is required to read the colour names and disregard the colour of the text. The second component requires the naming of the colour of the word instead of reading the word.

The Stroop takes 5-10 minutes to complete and is administered with the subject in the ON state at the end of the extension study (Week e40).

This test will not be performed in the Pilot Extension or the Supplemental Extension.

5.9.2.4 Other Brief Measures of Cognitive and Executive Function

Cognitive testing has two aspects in this trial. The first is to demonstrate safety, i.e. an absence of cognitive decline and, more broadly, identify potential issues of lowering of mood, suicidality and impulse control. The second is to assess efficacy – particularly focusing upon cognitive functions served by the putamen and fronto-striatal circuits.

As a safety measure, we have undertaken to document in the case notes deterioration in mood or impulsivity as assessed by direct questioning when the participant comes for each infusion visit (as one would do at a routine clinic review). We are not including a suicide risk scale assessment, but if someone reports significant lowering of mood we will enquire about suicidal thoughts etc. and document this in the case notes.

As an efficacy measure, we are employing the Stroop using a four card version that involves switching on the last card. In addition, however, we would like to add a more detailed assessment of verbal fluency than contained within the MoCA at baseline and 9 months. A potential difficulty with the Stroop alone is that participants can spot a mastering technique.

With regards to specific assays for putamen-related non motor functions, we are adding the Deary-Liewald reaction time test which is very sensitive, relies on putamenal dopamine and is short. We have Stroop at nine months post baseline and verbal fluency will be assessed at the same time points. The Deary-Liewald reaction time, however, as well as being assessed at baseline and nine months will be checked midway through the nine-month period, given the minimal impact of practice effects, performed at the time the MoCA is repeated. The Deary-Liewald reaction time tasks measure simple and choice (i.e. decision) reaction times. This has been validated in longitudinal studies, is suitable for older individuals, does not show much in the way of practice effects.

We are measuring height and weight, since BMI change might be an important outcome. We are also measuring appetite as a useful further outcome measure (4 item SNAQ). Posterior lateral putamen dopamine appears to be critical for eating.

Lastly, a self-report measure of executive function (FRSBE) is included at baseline and 9 months. This is very brief and easy to do. A self-report measure is helpful given our concerns around placebo effects.

These tests will not be performed in the Pilot Extension or the Supplemental Extension.

5.9.2.5 Beck Depression Inventory

The BDI (Appendix D) is a depression scale that is commonly used both in clinical trials and in clinical practice. It has been recommended by the Movement Disorders Society as an outcome measure to rate the severity of depression in PD. The BDI is a 21-question subject self-report scale with each item being rated on a scale of 0 - 3. Higher scores represent greater degrees of depression.

The BDI will be completed by the subject in the ON state at the end of the extension study (Week e40), at Week e2-80 in the Pilot Extension, and at the last visit in the Supplemental Extension.

5.9.2.6 Questionnaire for Impulsive- Compulsive Disorders in Parkinson's Disease

The QUIP (Appendix E) was developed to assess the occurrence of impulsive and compulsive disorders in PD. It is a subject self-administered scale which includes 13 questions covering symptoms related to the 4 commonest impulse control disorders in PD (gambling, sex, buying and eating) as well as other behaviours and problematic use of medication. The QUIP is to be completed by the subject while in the ON state at Weeks e8, e16, e24, e32 and e40, at Weeks e2-16, e2-32, e2-48, e2-64 and e2-80 in the Pilot Extension, and at Weeks e3-16, e3-32, and the last visit in the Supplemental Extension.

In addition, we will ask the spouse or partner to also complete a QUIP for the participant, although not in the Pilot Extension or the Supplemental Extension.

5.9.2.7 University of Pennsylvania Smell Identification Test British

The UPSIT can be self-administered and uses microencapsulated odorants, which are released by scratching standardised odor-impregnated test booklets. The study uses the British version of the UPSIT. The test can identify most malingerers and is sensitive to age, gender, smoking habits and a wide variety of olfactory disorders. The UPSIT can reliably identify and quantitate olfactory dysfunction in PD. The UPSIT is to be

completed regardless of ON/OFF state at the end of the extension study (Week e40) and at Week e2-80 of the Pilot Extension, but will not be completed in the Supplemental Extension.

5.10 Substudies

Independent substudies may be performed. Separate protocols will be prepared to describe the scope, objectives and procedures of these substudies.

5.11 Definition of End of Trial

The end of trial is the date of the last visit of the last subject.

5.12 Discontinuation/ Withdrawal of Subjects from Study Treatment

Each subject has the right to withdraw from the study at any time. In addition, the PI may discontinue a participant from the study at any time if considered necessary for any reason including:

- Pregnancy
- Ineligibility (either arising during the study or retrospectively, not having been identified at screening)
- Significant protocol deviation
- Significant non-compliance with treatment regimen or study requirements
- An AE which requires discontinuation of the study medication or results in inability to continue to comply with study procedures
- Disease progression which requires discontinuation of the study medication or results in inability to continue to comply with study procedures
- Consent withdrawn
- Lost to follow up

Subjects who discontinue the study early will be encouraged to observe the visit schedule for study assessments, at minimum the final visit (Section 5.6.4, Section 5.7.5, Section 5.8.4) unless they withdraw consent to do so.

The reason for withdrawal will be recorded in the CRF (withdrawal of consent or loss to follow-up will be recorded as such).

If the participant is withdrawn due to an AE, the PI or designee will arrange for follow-up visits or telephone calls until the AE has resolved or stabilised.

5.13 Source Data

Source documents are original documents, data, and records from which participants' CRF data are obtained. These include, but are not limited to, hospital records (from which medical history and previous and concurrent medication may be summarised into the CRF), clinical and office charts, laboratory and pharmacy records, diaries, microfiches, radiographs, and correspondence.

For the FrsBe, the subject will complete the assessment and the results will be transcribed into the CRF. For the other subject-completed questionnaires (PDQ-39, EQ-5D, QUIP, and SNAQ) and the diaries, the subject will complete the assessment directly into the CRF. Also, entries for the Deary-Liewald reaction time will be made directly on the CRF.

All documents will be stored safely in confidential conditions. On all study-specific documents, other than the signed consent, the participant will be referred to by the study participant number/code and initials, not by name.

6 TREATMENT OF TRIAL PARTICIPANTS

6.1 Description of Study Medication

The active drug is GDNF:

Chemical name:	Recombinant-methionyl human glial cell line-derived neurotrophic factor (r-metHuGDNF)
Physical description:	Clear, colourless liquid
Purity	Purity assumed to be 100% for dose calculation purposes
Concentration:	10 mg/mL in a buffer of 10 mM sodium citrate and 150 mM sodium chloride at pH 5.0
Container size:	2.0 mL
Fill size:	0.5 mL
Storage conditions:	-20 ± 5 °C

GDNF drug substance is prepared on behalf of MedGenesis Therapeutix, Inc., by Lonza Ltd., Visp, Switzerland; GDNF drug product at Aptuit Ltd., Glasgow, UK.

Artificial cerebrospinal fluid (CSF) will also be supplied. The artificial CSF (aCSF) is manufactured on behalf of MedGenesis Therapeutix, Inc. at Aptuit Ltd., Glasgow, UK and provided as:

Physical description:	Clear, colourless liquid
Container size:	20.0 mL
Fill size:	19.6 mL
Storage conditions:	15-30°C

Both GDNF and aCSF will be stored at the study site Pharmacy in secure, limited-access and temperature-controlled conditions.

Commercial gadopentetate dimeglumine (Magnevist®) will be used at a 2 mM concentration in aCSF for infusion at Week e40 in the extension study (Primary Study Stage subjects only) and at Weeks e2-0 and e2-80 in the Pilot Extension.

6.2 Description of Convection-Enhanced Delivery System

The drug delivery system comprises 4 microcatheters, 4 catheter guide tubes and a skull mounted transcutaneous drug delivery port. The microcatheters are connected under the scalp to separate in-line bacterial and bubble filters and further to the drug delivery port.

6.2.1 Implantation of the Drug Delivery System

The drug delivery system will have been implanted during the parent study; please refer to Protocol 2553 for details.

6.2.2 Drug Infusions

The drug administration system compromises 4 programmable syringe pumps, four 5-mL Plastipak syringes (Becton Dickinson), 4 sealed extension lines with male Luer attachments (each containing a septum) at one end, 4 double-sided syringe connectors with female Luer attachments (each holding an internal needle), 4 lengths of sealed drug administration tubing with male Luer attachments (each containing a septum) at both ends and a 4-channelled drug administration set. The latter compromises 4 needles mounted in a hub, each connected to independent bacteria and bubble filters and thence to short lengths of tubing, each with a female Luer attachment holding an internal needle. In addition, 3 syringe connectors with female Luer attachments at both ends and holding an internal needle at one end are used by the pharmacist preparing the study drug to fill the sealed extension lines and sealed drug administration lines.

Prior to drug administration, the pharmacist will fill the 4 lengths of drug administration tubing with an appropriate dose of drug (using 2 syringe connectors, one at each end of the sealed tubing), and label each line appropriately without identifying the treatment allocation. The pharmacist will further fill 4 syringes with aCSF, connect them to the open ends of the extension lines and fill the extension lines (using a single syringe connector at the opposite end to evacuate air). All syringe connectors will be disconnected and discarded once all lines have been filled. The filling of devices will take place up to 48 hours before a planned drug infusion.

For the infusion, each syringe/sealed extension line assembly will be connected to a double-sided-syringe connector, then to a drug administration line, then to one of the 4 channels of the drug administration set which prior to this has been flushed with aCSF.

Connection of the drug administration set to the port begins with the health professional cleaning the port and the immediate surrounds with an aseptic technique. The cylindrical needle hub of the drug administration set will be positioned over the port and locked to the port using a socket screw key. The 4 needles will be lowered through the port septum by use of a hand-tightened nut and thence individually guided into their separate channels that conduct the drug to identifiable catheters.

Once the connection has been secured, study medication is administered as detailed in Section 6.3. When the infusion is complete after approximately 90 to 150 minutes the administration set will be disconnected from the port by unscrewing both the nut and the socket screw.

6.3 Treatment

Study treatments will be prepared by a pharmacist at the study site. Ready to use preparations of GDNF will be provided to the PI or designee. Drug preparations guidelines are provided in a separate pharmacy manual.

At each treatment, infusion tubes will be connected with the transdermal port using standard aseptic technique. Every subject will receive study drug intraputamenally titrated up to an infusion rate of 3-5 μL/minute. The infusion rates will be up-titrated following a linear ramping scheme from 0 μL/min to 3-5 μL/min (0.18-0.30 mL/hour) over 30 to 40 minutes. The infusions will be delivered by external syringe pumps with pre-programmed infusion algorithms (Perfusor® Space, B. Braun, Melsungen, Germany).

Two catheters per putamen will be used, and 300 μL of study drug will be delivered per catheter at each treatment. This amounts to a dose of 120 μg GDNF per putamen per administration. An aCSF "flush" of up to 100 μL per catheter (at 3-5 μL/min) will clear the dead space after each infusion.

The infusions will be delivered by trained personnel. During the infusion, subjects will be semi-recumbent in a reclining chair. The total procedure time for the completion of the infusions will be approximately 90 to 150 minutes depending on potential reflux parameters observed on post infusion MRIs.

6.4 Compliance with Study Treatment

All treatments will be administered by PI or designee. Administration records will be kept on site, and administration information, including time and date of infusion, identification of the infusate, infusion rate and duration and the reasons for any interruptions of the infusion or for any missed or omitted infusions, will be recorded in the CRF.

6.5 Accountability of the Study Treatment

It is forbidden to use investigational drug material for purposes other than as defined in this protocol.

All supplies of study medication will be accounted for in accordance with Good Clinical Practices (GCP). There will be an individual study drug accountability record for each subject and the pharmacist is to maintain accurate records of the disposition of all study medication supplies received and dispensed during the study. These records will include the amounts and dates clinical drug supplies were received, dispensed to the PI or designee for any given subject, and returned to the manufacturer. If errors or damages in the clinical drug supply shipments occur, the pharmacist will notify the manufacturer immediately so that corrective action can be taken as needed. Copies of the study medication accountability records will be provided by the pharmacist for inclusion in the Trial Master File (TMF) after database lock. The study monitor will periodically check the supplies of study medication held by the pharmacist to verify accountability of all medication used.

The PI or designee will administer the medication only to the identified subjects of this study, according to the procedures described in this study protocol. After the end of the study, all unused medication and all medication containers can be destroyed on-site as long as proper documentation is supplied. If destruction on-site is not possible then medication and all medication containers will be returned to the manufacturer for destruction and documentation will be returned to the manufacturer. The manufacturer will verify that a final report of drug accountability is prepared and maintained in the Investigator's Study Centre File.

6.6 Test Infusions

A test infusion of gadolinium contrast-containing diluent will be administered at Week e40 in the extension study (Primary Study Stage subjects only) and at Weeks e2-0 and e2-80 in the Pilot Extension. GDNF will not be delivered at any of these visits. The infusions will be freshly prepared on site prior to use following the steps described for study medication above. Specifically, 4 lengths of drug administration tubing (each holding 300 μL) will be filled 2 mM gadopentetate dimeglumine in aCSF (1:250 dilution of Magnevist®). The drug administration lines will then be connected as specified in Section 6.2.2, and the infusate will be delivered according to the infusion protocol provided in Section 6.3.

6.7 Concomitant Medication

Subjects should have PD medication optimised and stabilised prior to study entry. Every effort should be made not to increase subjects' medication for PD during the study, but medication may be increased if required to maintain the subject's well being. PD medication dose may be decreased at the discretion of the PI to manage PD drug-related side-effects. Non-PD medications may be altered at any time at the PI's discretion.

Throughout the study the PI may prescribe any concomitant medications or treatments deemed necessary to provide adequate supportive care.

Any medication other than the study medication, including prescription or over-the-counter medications, vitamins and herbal medications taken during the study will be recorded in the CRF with their indications.

7 SAFETY REPORTING

7.1 Definitions

7.2 Adverse Event (AE)

An AE or adverse experience is:

Any untoward medical occurrence in a subject or clinical investigation participant administered a medicinal product, which does not necessarily have a causal relationship with this treatment (the study medication).

An AE can therefore be any unfavourable and unintended sign (including an abnormal laboratory finding), symptom or disease temporally associated with the use of the study medication, whether or not considered related to the study medication.

7.3 Adverse Reaction (AR)

All untoward and unintended responses to a medicinal product related to any dose.

The phrase "responses to a medicinal product" means that a causal relationship between a study medication and an AE is at least a reasonable possibility, i.e., the relationship cannot be ruled out.

All cases judged by either the reporting medically qualified professional or the Sponsor as having a reasonable suspected causal relationship to the study medication qualify as ARs.

7.4 Serious Adverse Event (SAE)

An SAE is any untoward medical occurrence that at any dose:

- Results in death
- Is life-threatening; NOTE: The term "life-threatening" in the definition of "serious" refers to an event in which the participant was at risk of death at the time of the event; it does not refer to an event which hypothetically might have caused death if it were more severe.
- Requires inpatient hospitalisation or prolongation of existing hospitalisation
- Results in persistent or significant disability/incapacity

- Is a congenital anomaly/birth defect
- Other important medical events; NOTE: Other events that may not result in death, are not life-threatening, or do not require hospitalisation, may be considered an SAE when, based upon appropriate medical judgement, the event may jeopardise the subject and may require medical or surgical intervention to prevent one of the outcomes listed above.

To ensure no confusion or misunderstanding of the difference between the terms "serious" and "severe," which are not synonymous, the following note of clarification is provided:

The term "severe" is often used to describe the intensity (severity) of a specific event (as in mild, moderate, or severe myocardial infarction); the event itself, however, may be of relatively minor medical significance (such as severe headache). This is not the same as "serious," which is based on subject/event outcome or action criteria usually associated with events that pose a threat to a participant's life or functioning as defined in the bullet points above. Seriousness (not severity) serves as a guide for defining regulatory reporting obligations.

7.5 Serious Adverse Reaction (SAR)

An AE (expected or unexpected) that is both serious and, in the opinion of the reporting Investigator, believed with reasonable probability to be due to one of the study treatments, based on the information provided.

7.6 Suspected Unexpected Serious Adverse Reaction (SUSAR)

An SAR, the nature or severity of which is not consistent with the applicable product information (e.g., Investigator's Brochure for an unapproved investigational product or Summary of Medicinal Product Characteristics [SMPC] for an approved product).

7.7 Causality and Expectedness

The relationship of each AE to the trial medication must be determined by a medically qualified individual according to the following definitions:

Related: The AE follows a reasonable temporal sequence from trial medication administration. It cannot reasonably be attributed to any other cause.

Not Related: The AE is probably produced by the participant's clinical state or by other modes of therapy administered to the participant.

The expectedness of each **SAE** in the trial must be determined by a medically qualified individual according to the following definition:

Unexpected Adverse Drug Reaction: An adverse reaction, the nature or severity of which is not consistent with the applicable product information (i.e., Investigator's Brochure).

7.8 Procedures for Recording Adverse Events

All AEs occurring during the study until 28 days after the last dose of GDNF, observed by the PI or designee or reported by the participant, whether or not attributed to study medication, will be recorded on the AE pages of the CRF.

The following information will be recorded: description, date and time of onset, date and time of end, occurrence during infusion, severity, assessment of relatedness to study medication or device, seriousness, action taken, and outcome. Follow-up information should be provided as necessary.

AEs considered related to the study medication or to the device as judged by the PI or a medically qualified designee will be followed until resolution or the event is considered stable. All related AEs that result in a participant's withdrawal from the study or are present at the end of the study should be followed up until a satisfactory resolution occurs.

It will be left to the PI's or medically qualified designee's clinical judgment whether or not an AE is of sufficient severity to require the participant's removal from treatment. A participant may also voluntarily withdraw from treatment due to what he or she perceives as an intolerable AE. If either of these occurs, the participant must undergo an end of study assessment (unless he or she has withdrawn consent to do so) and be given appropriate care under medical supervision until symptoms cease or the condition becomes stable.

The severity of events will be assessed on the following scale:
1 = mild, 2 = moderate, 3 = severe.

The relationship of AEs to the study medication will be assessed by the PI or a medically qualified designee.

Any pregnancy occurring during the clinical study and the outcome of the pregnancy should be recorded and followed up for any congenital abnormality or birth defect.

7.9 Reporting Procedures for Serious Adverse Events

All SAEs must be reported to North Bristol NHS Trust Research and Innovation (NBT R&I) within 24 hours of discovery or notification of the event. NBT R&I will perform an initial check of the report, request any additional information and ensure it is reviewed. All SAEs must be reviewed at the next Trial Safety Group meeting. All SAE information must be recorded on an SAE form and faxed or emailed to NBT R&I. Additional information received for a case (follow-up or corrections to the original case) need to be detailed on a new SAE form and faxed or emailed to NBT R&I.

7.10 SUSAR Reporting

The PI will support NBT R&I to report all SUSARs to the MHRA, the local Research Ethics Committee (REC), and the manufacturer of the drug. Fatal or life-threatening SUSARs must be reported within 7 days and all other SUSARs within 15 days. The PI will inform all study staff concerned of relevant information about SUSARs that could adversely affect the safety of participants.

7.11 Annual Safety Reports

In addition to the expedited reporting above, the PI shall submit once a year throughout the clinical trial or on request a safety report to the MHRA, REC, and manufacturer of the drug.

8 STATISTICS

A full, detailed statistical analysis plan (SAP) will be prepared and completed before study data are locked.

8.1 Description of Statistical Methods

Descriptive statistics will be presented for all endpoints by treatment group supporting the primary and secondary analyses. Detailed methodology will be provided in the SAP. No inferential analyses are planned for this open-label extension study.

The efficacy analyses will be conducted after all Primary Study Stage subjects have reached the 18-month endpoint and will include only subjects who had been randomised to the Primary Study Stage of Study 2553. Supplementary analyses will include subjects treated in the Pilot Stage.

Safety data will be presented descriptively, by treatment group, with standard Medical Dictionary for Regulatory Activities (MedDRA) coded AE and SAE frequency and incidence tables as well as shift tables for clinical laboratory parameters.

The Safety Population will comprise all subjects treated on study with subjects grouped according to treatment actually received. All safety analyses will be performed on the Safety Population.

8.2 Number of Participants

This is an open-label extension study. No power calculations were performed. The study will be open to all subjects who complete Study 2553 without significant toxicity or other exclusion.

8.3 Level of Statistical Significance

No inferential analyses are planned for this open-label extension study.

8.4 Criteria for the Termination of the Trial

The study will be monitored by an independent DMC established by the sponsor or designee and governed by a separate charter. The DMC may recommend termination of the trial at any point for safety reasons.

This study may be terminated by the Sponsor. The study may also be terminated prematurely at any time when agreed to by both the PI and the Sponsor as being in the best interests of subjects, and justified on either medical or ethical grounds. In terminating the study, the Sponsor and the PI will ensure that adequate consideration is given to the protection of the subjects' interests.

8.5 Procedure for Accounting for Missing, Unused, and Spurious Data

Methods for handling missing or incomplete data will be described in the SAP.

8.6 Procedures for Reporting any Deviation(s) from the Original Statistical Plan

Changes to the SAP planned before study data are locked will be documented in a revised SAP or SAP addendum or amendment. Any subsequent changes to the planned analyses will be clearly identified in the final Clinical Study Report.

8.7 Inclusion in Analysis

The primary efficacy analyses will be conducted after all Primary Study Stage subjects have reached the 18-month endpoint and will include only subjects who had been randomised to the Primary Study Stage of Study 2553. Supplementary analyses will include subjects treated in the Pilot Stage.

The Safety Population will comprise all subjects treated on study with subjects grouped according to treatment actually received. All safety analyses will be performed on the Safety Population. They will be performed separately for the subjects who had been randomised to the Pilot and Primary Study Stages of Study 2553 and for all subjects and both stages combined.

Additional analysis populations may be defined in the SAP.

9 DIRECT ACCESS TO SOURCE DATA/DOCUMENTS

Direct access will be granted to authorised representatives from the Sponsor, host institution, manufacturer and the regulatory authorities to permit trial-related monitoring, audits and inspections.

10 QUALITY CONTROL AND QUALITY ASSURANCE PROCEDURES

The study will be conducted in accordance with the current approved protocol, International Conference on Harmonisation (ICH) GCP, relevant regulations and standard operating procedures.

Regular monitoring will be performed according to ICH GCP. The Sponsor or designee will complete a monitoring plan and provide a copy for the TMF. Data will be evaluated for compliance with the protocol and accuracy in relation to source documents. Following written standard operating procedures, the monitors will verify that the clinical trial is conducted and data are generated, documented and reported in compliance with the protocol, GCP and the applicable regulatory requirements.

The Sponsor or designee will establish an independent DMC. The establishment and operation of the DMC will adhere to relevant guidance, and the responsibilities and processes of the DMC will be specified in a DMC charter.

11 SERIOUS BREACHES

The Medicines for Human Use (Clinical Trials) Regulations contain a requirement for the notification of "serious breaches" to the MHRA within 7 days of the Sponsor becoming aware of the breach.

A serious breach is defined as "A breach of GCP or the trial protocol which is likely to affect to a significant degree –

(a) the safety or physical or mental integrity of the subjects of the trial; or

(b) the scientific value of the trial."

In the event that a serious breach is suspected NBT R&I should be contacted as soon as possible. Breaches of GCP will be managed in accordance with ISOP-C05 Noncompliance with ICH Good Clinical Practice (GCP) Guidelines (http://www.nbt.nhs.uk/education__research/researcher_resources/useful_documents__forms/standard_operating_procedures.aspx)

12 ETHICS

12.1 Declaration of Helsinki

The PI will ensure that this study is conducted in accordance with the principles of the Declaration of Helsinki (as amended by the World Medical Association in Seoul, Republic of Korea, October 2008).

12.2 ICH Guidelines for Good Clinical Practice

The PI will ensure that this study is conducted in full conformity with relevant regulations and with the ICH Guidelines for GCP (CPMP/ICH/135/95) July 1996.

12.3 Approvals

The protocol, informed consent form, participant information sheet and any proposed advertising material will be submitted to the appropriate REC, MHRA, and applicable host institution(s) for written approval.

The PI will submit and, where necessary, obtain approval from the above parties for all substantial amendments to the original approved documents.

12.4 Participant Confidentiality

The trial staff will ensure that the participants' anonymity is maintained. The participants will be identified only by initials and a participant identification number in the CRF and any written study materials. All documents will be stored securely and only accessible by trial staff and authorised personnel. The study will comply with the Data Protection Act which requires data to be anonymised as soon as it is practical to do so.

13 DATA HANDLING AND RECORD KEEPING

Paper CRFs will be used for the current study and a Data Management Plan will be prepared by the Sponsor or designee. The CRF data will be compared to source documentation by the study monitors. All monitored data will be processed by a data management group. Data edit checks will be performed using both CRFs and SAS® programming as detailed in the Data Management Plan. Data queries will be issued to the clinical site in order to resolve any discrepancies found during the discrepancy management process, and data will be updated accordingly. The participants will be identified by a study specific participant number and/or code in any database. The name and any other identifying detail will NOT be included in any study data electronic file.

Previous and concomitant medications will be coded using the latest available World Health Organization (WHO) Drug Reference Dictionary. Coexistent diseases and AEs will be coded using MedDRA.

When the database has been declared to be complete and accurate, it will be locked. Any changes to the database after that time can only be made by written agreement and documented in the TMF.

14 FINANCE AND INSURANCE

14.1 Compensation for Harm

Good governance in respect of the monitoring of the trial will be provided through NBT, acting as the Sponsor of this trial. NBT undertakes to maintain an appropriate clinical study insurance policy. In the event that something does go wrong and participants are harmed during the research and this is due to someone's negligence, then participants may have grounds for a legal action for compensation against NBT. However, participants may have to pay their legal costs.

With NBT as the Sponsor, this project is covered by NHS Litigation Authority Indemnity Insurance.

This is a non-commercial trial and as such is mandated to have indemnity in respect of negligent harm only; there is no provision for indemnity in respect of liabilities arising from non-negligent harm. In other words, if participants come to harm through this trial due to proven negligence they will be able to attempt to claim under the negligent harm scheme. However, unforeseen adverse events (and we do not know all the potential long term complications of receiving GDNF therapy or having catheter systems in the brain) or adverse events that occur without there being negligence on the part of the investigating team are not covered by this scheme. This will be explained in detail to participants at the time they consent to participate in the double-blind study.

Deviations from the study protocol – especially the prescription of a dose other than that scheduled in the study protocol, other modes of administration, other indications, and longer treatment periods – are not permitted and shall not be covered by the statutory subject insurance scheme.

15 PUBLICATION POLICY

Authorship of any publication will be based upon substantial contribution to the design, analysis, interpretation of data, drafting and/or critically revising any manuscript(s) derived from the Study. The Sponsor, PI and/or Study Neurosurgeon shall have the right to publish the results of the Study. Intended publications employing data from the study will be discussed with all members of the Steering Committee in advance of writing up to allow all members to contribute.

The Drug Manufacturer must receive copies of any intended communication at least sixty (60) days in advance to review a manuscript and fifteen (15) days to review any poster presentation, abstract or other written or oral material which describes the results of the Study. In addition, if Drug Manufacturer requests in writing, the Sponsor, PI and/or Study Neurosurgeon shall withhold any publication or presentation an additional thirty (30) days to allow for further clarification.

16 REFERENCES

[1] Lin LF, Doherty DH, Lile JD, Bektesh S, Collins F. GDNF: a glial cell line-derived neurotrophic factor for midbrain dopaminergic neurons. Science 1993;260(5111):1130-2.

[2] Airaksinen MS, Saarma M. The GDNF family: signalling, biological functions and therapeutic value. Nat Rev Neurosci 2002;3(5):383-94.

[3] Bobo RH, Laske DW, Akbasak A, Morrison PF, Dedrick RL, Oldfield EH. Convection-enhanced delivery of macromolecules in the brain. Proc Natl Acad Sci U S A 1994;91(6):2076-80.

[4] Fiandaca MS, Forsayeth JR, Dickinson PJ, Bankiewicz KS. Image-guided convection-enhanced delivery platform in the treatment of neurological diseases. Neurotherapeutics 2008;5(1):123-7.

17 APPENDIX A: STUDY SCHEDULE OF EVENTS

Table 1 Schedule of Events

Procedure/Assessments	Week										
	e0 [a]	e4	e8 [b]	e12	e16 [b]	e20	e24 [b]	e28	e32 [b]	e36	e40 [b]
Informed consent [c]	(X)										
MRI [d]											X
Vital signs [e]	X	X	X	X	X	X	X	X	X	X	X
Weight and height	(X)		X		X		X		X		X
Physical examination [f]											X
ECG											X
Laboratory assessment [g]		X			X			X			X
Anti-GDNF antibody levels and GDNF plasma concentration		X			X			X			X
UPDRS part II and part III in OFF state	(X)		X		X		X		X		X
Timed walking test in OFF state	(X)		X		X		X		X		X
Timed tapping test in OFF state	(X)		X		X		X		X		X
Levodopa challenge	(X)		X		X		X		X		X
UPDRS in on state	(X)		X		X		X		X		X
Timed walking test in on state	(X)		X		X		X		X		X
Timed tapping test in on state	(X)		X		X		X		X		X
PDQ-39											X
EQ-5D											X
MoCA & MDRS					X						X
Stroop test											X
UPSIT											X
NMSS				X			X				X
BDI											X
QUIP	(X)		X		X		X		X		X
Deary-Liewald reaction time					X						X
SNAQ											X
Executive function self report											X
Verbal fluency											X
Direct questioning of impulsivity, mood, falls and freezing for recording in case notes	X	X	X	X	X	X	X	X	X	X	X
Collect PD fluctuation diaries	(X)		X		X		X		X		X
Dispense PD fluctuation diaries		X		X	X	X		X		X	
Infusion of study drug	X	X	X	X	X	X	X	X	X	X	
Glasgow Coma Scale [h]	X	X	X	X	X	X	X	X	X	X	X
Adverse events [i]	X	X	X	X	X	X	X	X	X	X	X
Concomitant medications [j]	X	X	X	X	X	X	X	X	X	X	X

a Assessments that are put in parentheses will be done at the Week 40 visit in Study 2553 or earlier (informed consent). With the exception of informed consent, no procedures are required specifically for the extension study; however, procedures scheduled for Week 40 in Study 2553 must be performed in accordance with that protocol.

b At Weeks e8, e16, e24 and e32, no PD medications will be taken after 6:00 PM on the night before the assessments and no long-acting PD medications will be taken on the day before the assessments. Subjects will refrain from eating any high-protein foods on the morning of the assessments.

c Informed consent must be obtained before any extension study-specific procedures or assessments are performed.

d T1-weighted as well as T2-weighted and FLAIR 3T MRI are to be completed in Primary Study Stage subjects within 2 hours of a gadolinium contrast-containing test infusion at Week e40 (±1 week). If possible, the test infusion and MRI should be done 1 week before the visit.

e Seated systolic and diastolic BP, HR, RR and temperature will be assessed after the subject has been sitting quietly for at least 5 minutes. Standing systolic and diastolic BP and HR will also be assessed after the subject has been standing for 3 minutes. During administration, seated vital signs (systolic and diastolic BP, HR and RR) will be collected every 15 (Week e0) to 30 (Weeks e4 to e36) minutes, and both seated and standing systolic and diastolic BP and HR will be assessed within 30 minutes after the end of the infusion.

f Brief physical examination, targeted, at the Investigator's discretion, to identify changes from baseline and from the previous assessment.

g Haematology (haematocrit, haemoglobin, MCH, MCHC, MCV, platelet count, RBC count, WBC count and WBC differential [basophils, eosinophils, lymphocytes, monocytes and neutrophils]), serum chemistry (albumin, alkaline phosphatase, ALT, creatinine, eGFR, glucose, potassium, sodium, total bilirubin and urea) and urinalysis (colour, appearance, pH, glucose, ketones and nitrite; microscopy as required to follow up abnormal observations). A pregnancy test will be performed for all women of childbearing potential (women not of childbearing potential are those who are surgically sterile or are > 45 years of age and without menses for ≥ 2 years).

h Performed before infusion, 30 minutes into the infusion, and after completion of the infusion.

i All AEs occurring during the study until 28 days after the last dose of GDNF will be recorded on the AE pages of the CRF. AEs meeting the criteria for SAEs (Section 7.4) must be reported as directed in Section 7.9.

j Concomitant medications: from study entry and until Week e40 or early discontinuation will be recorded. All over-the-counter or prescription medications, vitamins and/or herbal supplements will be recorded in the CRF with their indications.

Table 2 Study Procedures of Additional Pilot Extension

											Week in Extension 2										
**Procedure/Assessments**	**e2-0 [a]**	**e2-4**	**e2-8**	**e2-12**	**e2-16 [b]**	**e2-20**	**e2-24**	**e2-28**	**e2-32 [b]**	**e2-36**	**e2-40**	**e2-44**	**e2-48**	**e2-52**	**e2-56 [b]**	**e2-60**	**e2-64**	**e2-68**	**e2-72**	**e2-76**	**e2-80 [b]**
Informed consent [c]	(X)																				
MRI [d]	X																				X
Vital signs [e]	X	X	X	X	X	X	X	X	X	X	X	X	X	X	X	X	X	X	X	X	X
Weight and height							X								X						X
Physical examination [f]																					X
ECG																					X
Laboratory assessment [g]																					X
Anti-GDNF antibody levels and GDNF plasma concentration							X						X						X		
UPDRS part II and part III in OFF state	(X)				X				X						X						X
Timed walking test in OFF state	(X)				X				X						X						X
Timed tapping test in OFF state	(X)				X				X						X						X
Levodopa challenge	(X)				X				X						X						X
UPDRS in on state	(X)				X				X						X						X
Timed walking test in on state	(X)				X				X						X						X
Timed tapping test in on state	(X)				X				X						X						X
MoCA & MDRS	(X)																				X
UPSIT	(X)																				X
NMSS	(X)										X										X
BDI	(X)																				X
QUIP	(X)				X				X				X				X				X
Direct questioning of impulsivity, mood, falls and freezing for recording in case notes	X	X	X	X	X	X	X	X	X	X	X	X	X	X	X	X	X	X	X	X	X
Collect PD fluctuation diaries	(X)				X				X						X						X
Dispense PD fluctuation diaries				X				X						X						X	
Infusion of study drug		X	X	X	X	X	X	X	X	X	X	X	X	X	X	X	X	X	X	X	
Glasgow Coma Scale [h]	X	X	X	X	X	X	X	X	X	X	X	X	X	X	X	X	X	X	X	X	X
Adverse events [i]	X	X	X	X	X	X	X	X	X	X	X	X	X	X	X	X	X	X	X	X	X
Concomitant medications [j]	X	X	X	X	X	X	X	X	X	X	X	X	X	X	X	X	X	X	X	X	X

a Visit e2-0 will take place at the same time (or within 1 week) of visit e40. Assessments in parentheses will be done at Week e40 or earlier (informed consent). The safety assessments and outcome measures taken at Week e40 will serve as the baseline for this part of the study and do not need to be repeated at Visit e2-0.

b At Weeks e2-16, e2-32, e2-56, and e2-80, no PD medications will be taken after 6:00 PM on the night before the assessments and no long-acting PD medications will be taken on the day before the assessments. Subjects will refrain from eating any high-protein foods on the morning of the assessments.

c Informed consent must be obtained before any extension study-specific procedures or assessments are performed.

d T1-weighted as well as T2-weighted and FLAIR 3T MRI are to be completed within 2 hours of a gadolinium contrast-containing test study drug infusion at Weeks e2-0 and e2-80 (±1 week). At the Week e2-80 visit, the test infusion and MRI should be done 1 week before the visit, if possible.

e Seated systolic and diastolic BP, HR, RR and temperature will be assessed after the subject has been sitting quietly for at least 5 minutes. Standing systolic and diastolic BP and HR will also be assessed after the subject has been standing for 3 minutes. During administration, seated vital signs (systolic and diastolic BP, HR and RR) will be collected every 30 minutes, and both seated and standing systolic and diastolic BP and HR will be assessed within 30 minutes after the end of the infusion.

f Brief physical examination, targeted, at the Investigator's discretion, to identify changes from baseline and from the previous assessment.

g Haematology (haematocrit, haemoglobin, MCH, MCHC, MCV, platelet count, RBC count, WBC count and WBC differential [basophils, eosinophils, lymphocytes, monocytes and neutrophils]), serum chemistry (albumin, alkaline phosphatase, ALT, creatinine, eGFR, glucose, potassium, sodium, total bilirubin and urea) and urinalysis (colour, appearance, pH, glucose, ketones and nitrite; microscopy as required to follow up abnormal observations). A pregnancy test will be performed for all women of childbearing potential (women not of childbearing potential are those who are surgically sterile or are > 45 years of age and without menses for ≥ 2 years).

h Performed before infusion, 30 minutes into the infusion, and after completion of the infusion.

i All AEs occurring during the study until 28 days after the last dose of GDNF will be recorded on the AE pages of the CRF. AEs meeting the criteria for SAEs (Section 7.4) must be reported as directed in Section 7.9.

j Concomitant medications from study entry and until Week e2-80 or early discontinuation will be recorded. All over-the-counter or prescription medications, vitamins and/or herbal supplements will be recorded in the CRF with their indications.

Table 3 Study Procedures of Supplemental Extension

Procedure/Assessments	Week in Supplemental Extension													
	e3-0 [a]	e3-4	e3-8	e3-12	e3-16 [b]	e3-20	e3-24	e3-28	e3-32 [b]	e3-36	e3-40	e3-44	e3-48 [c]	Last Study Visit Additional procedures/assessments [c]
Informed consent [d]	(X)													
Vital signs [e]	X	X	X	X	X	X	X	X	X	X	X	X	X	
Laboratory assessment [f]														X
Anti-GDNF antibody levels and GDNF plasma concentration														X
UPDRS part II and part III in OFF state	(X)				X				X					
Timed walking test in OFF state	(X)				X				X					
Timed tapping test in OFF state	(X)				X				X					
Levodopa challenge	(X)				X				X					
UPDRS in on state	(X)				X				X					
Timed walking test in on state	(X)				X				X					
Timed tapping test in on state	(X)				X				X					
MoCA & MDRS														X
BDI	(X)													X
QUIP	(X)				X				X					X
Direct questioning of impulsivity, mood, falls and freezing for recording in case notes	X	X	X	X	X	X	X	X	X	X	X	X	X	
Collect PD fluctuation diaries	(X)				X				X					
Dispense PD fluctuation diaries				X				X						
Infusion of study drug	X	X	X	X	X	X	X	X	X	X	X	X	X	
Glasgow Coma Scale [g]	X	X	X	X	X	X	X	X	X	X	X	X	X	
Adverse events [h]	X	X	X	X	X	X	X	X	X	X	X	X	X	
Concomitant medications [i]	X	X	X	X	X	X	X	X	X	X	X	X	X	

a Visit e3-0 will take place approximately 1-2 weeks after Week e40 (Primary Study Stage subjects) or Week e2-80 (Pilot Stage subjects). Assessments in parentheses will be done at Week e40 (Primary Study Stage subjects) or Week e2-80 (Pilot Stage subjects) or earlier (informed consent). The safety assessments and outcome measures taken at Week e40 or Week e2-80 (whichever is applicable) will serve as the baseline for this part of the study and do not need to be repeated at Week e3-0.

b At Weeks e3-16 and e3-32, no PD medications will be taken after 6:00 PM on the night before the assessments and no long-acting PD medications will be taken on the day before the assessments. Subjects will refrain from eating any high-protein foods on the morning of the assessments.

c At the last study visit in December 2016 (which will occur at Week e3-48 or earlier), the subject will undergo all procedures and assessments scheduled for the respective visit reached by the subject as per the visit schedule. In addition, regardless of the assessments scheduled for the respective visit, MoCA, MDRS, QUIP and BDI assessments will be performed and samples will be obtained for laboratory assessment and determination of anti-GDNF antibody levels and GDNF plasma concentrations. The same approach to final assessments should be taken, if possible, for any subjects who discontinue the Supplemental Extension early.

d Informed consent must be obtained before any extension study-specific procedures or assessments are performed.

e Seated systolic and diastolic BP, HR, RR and temperature will be assessed after the subject has been sitting quietly for at least 5 minutes. Standing systolic and diastolic BP and HR will also be assessed after the subject has been standing for 3 minutes. During administration, seated vital signs (systolic and diastolic BP, HR and RR) will be collected every 30 minutes, and both seated and standing systolic and diastolic BP and HR will be assessed within 30 minutes after the end of the infusion.

f Haematology (haematocrit, haemoglobin, MCH, MCHC, MCV, platelet count, RBC count, WBC count and WBC differential [basophils, eosinophils, lymphocytes, monocytes and neutrophils]), serum chemistry (albumin, alkaline phosphatase, ALT, creatinine, eGFR, glucose, potassium, sodium, total bilirubin and urea) and urinalysis (colour, appearance, pH, glucose, ketones and nitrite; microscopy as required to follow up

abnormal observations). A pregnancy test will be performed for all women of childbearing potential (women not of childbearing potential are those who are surgically sterile or are > 45 years of age and without menses for ≥ 2 years).

g Performed before infusion, 30 minutes into the infusion, and after completion of the infusion.

h All AEs occurring during the study until 28 days after the last dose of GDNF will be recorded on the AE pages of the CRF. AEs meeting the criteria for SAEs (Section 7.4) must be reported as directed in Section 7.9.

i Concomitant medications from study entry and until last study visit will be recorded. All over-the-counter or prescription medications, vitamins and/or herbal supplements will be recorded in the CRF with their indications.

Summary of Protocol Amendments

An Extension Study to Assess the Safety and Efficacy of Intermittent Bilateral Intraputamenal Glial Cell Line-Derived Neurotrophic Factor (GDNF) Infusions Administered via Convection-Enhanced Delivery (CED) in Subjects with Parkinson's Disease

Internal Reference No: 2797

Ethics Ref: 13/SW/0181

EudraCT Number: 2013-001881-40

The original protocol (version 1.0) was dated 18 Jun 2013. There were 4 protocol amendments (3 major, 1 minor). A summary of the most important changes introduced by each amendment is given below.

1.1.1.1 Amendment 1, dated 02 Sep 2013

This amendment revised protocol section 7.9 (Reporting Procedures for Serious Adverse Events) to harmonize the initial reporting period for serious AEs with current effective regulations stipulating that all serious AEs had to be reported to North Bristol NHS Trust Research and Innovation within 24 hours (instead of 1 working day) of discovery or notification of the event.

1.1.1.2 Amendment 2, dated 30 Jun 2014

This amendment added further extension study visits for Pilot Stage subjects (Pilot Extension), corrected minor protocol inconsistencies, provided the new addresses for the Sponsor, principal investigator, and study neurosurgeon, and removed the Cattell Culture Fair Intelligence Test as an outcome measure.

The Pilot Stage subjects from study 2553 began treatment substantially before those from the main study cohort (Primary Stage). The Pilot Extension comprised an additional 80 weeks of treatment and was designed to generate long-term safety data and ensure ongoing access to GDNF for the Pilot Stage subjects until results of study 2553 become available. At the beginning of the Pilot Extension (Week e2-0), Pilot Stage subjects were to receive an infusion of aCSF containing gadolinium contrast to allow for volumetric catheter performance by means of T1-weighted MRI scans in addition to T2-weighted and FLAIR MRI scans.

1.1.1.3 Amendment 3, dated 19 Sep 2014

This amendment modified the MRI schedule to reflect changes introduced by amendments in study 2553. A gadolinium contrast-containing test infusion of aCSF, followed both by contrast-enhanced T1-weighted MRI and by T2-weighted and FLAIR MRI scans, was added at Week 80/e40 in the extension study (Primary Stage subjects only) and at Weeks e2-0 and e2-80 in the Pilot Extension. GDNF was not to be delivered at any of these visits. A 2-week window around the Week 80/e40 and Week e2-80 visits was allowed for the test infusions, in order to minimize the burden on subjects at these busy visits while keeping a 3- to 5-week window from the preceding infusion of GDNF at Week 76/e36 and Week e2-76, respectively. Interim MRI scans were no longer to be done during the study unless clinically indicated.

1.1.1.4 Amendment 4, dated 16 Dec 2015

This amendment added further extension study visits to facilitate continuation of the study until anticipated completion at the end of December 2016. The amendment also provided a number of clarifications and refinements that were considered mostly administrative in nature or evolved during the preparation of protocol amendment 7 of study 2553. The most important changes were:

- **Supplemental Extension**: Further extension study visits were added to generate long-term safety data and to facilitate continuation of the study until the end of December 2016 when the results of study 2553 were expected. The protocol was modified accordingly.
- **Disclosure of treatment codes in parent study**. Where applicable, the protocol text was modified to clarify that individual treatment codes from the parent study 2553 would not be disclosed to subjects until database lock for study 2797, unless required for specific safety reasons. In addition, additional text was inserted to indicate that every effort would be made to avoid unblinding of the blinded UPDRS raters until database lock for study 2797.
- **Timing of test infusion relative to visits:** For the test infusions to be administered at Week 80/e40 and Week e2-80, the protocol was modified to clarify that the test infusion followed by T1-weighted, T2-weighted, and FLAIR MRI scans should take place preferably 1 week before the study visit, if possible.
- **Protocol section 5.6.1 (Treatments):** In order to avoid infusion intervals of <3 weeks, the protocol text was modified to clarify that, if necessary, a treatment could be given a maximum of + 7 days from the scheduled date; if the treatment could not be given within + 7 days, it was to be considered missed and the treatments resumed with the following scheduled date.
- **MoCA and MDRS:** The protocol was modified to indicate that the MoCA and MDRS were also to be performed at Week e2-80 at the end of the Pilot Extension.
- **AE documentation:** The protocol was revised to eliminate inconsistencies with respect to the required approach to documentation of AEs. All AEs occurring during the study until 28 days after the last dose of GDNF were to be recorded on the AE pages of the CRF. In addition, the protocol was revised to correctly reflect the information documented in the CRF for each AE.
