# Supplementary figures and images for: Extended Treatment with Glial Cell Line-Derived Neurotrophic Factor in Parkinson’s Disease
Source: J Parkinsons Dis. 2019 May 23;9(2):301–13. doi: 10.3233/JPD-191576 (PMC6597995; doi:10.3233/JPD-191576)

## Appendix B – CONSORT Flow Diagram

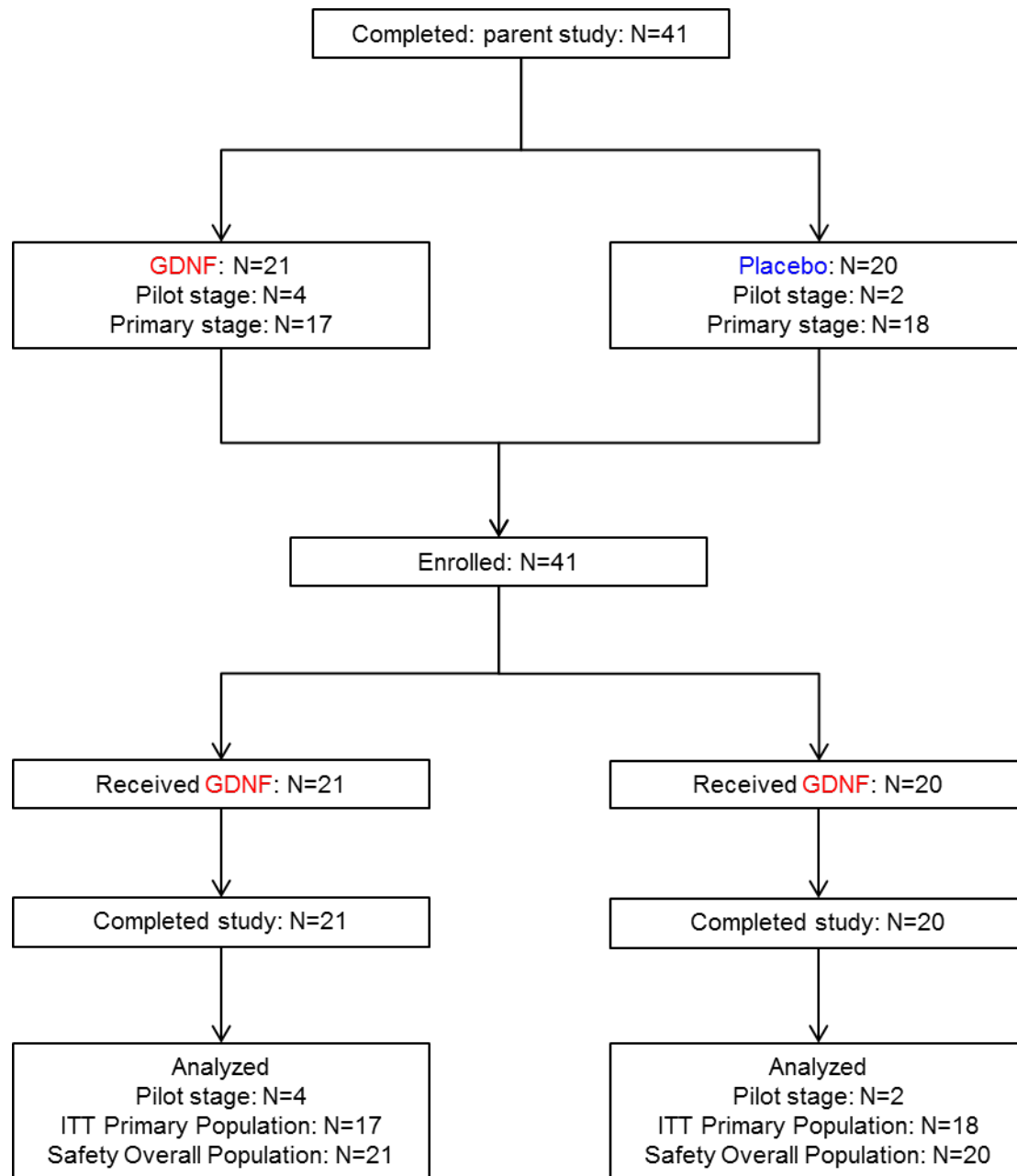

Supplement: Supplementary Material — Appendix B – CONSORT flow diagram [file jpd-9-jpd191576-s002.pdf]
