# Supplementary material for: Extended Treatment with Glial Cell Line-Derived Neurotrophic Factor in Parkinson’s Disease
Source: J Parkinsons Dis. 2019 May 23;9(2):301–13. doi: 10.3233/JPD-191576 (PMC6597995; doi:10.3233/JPD-191576)
Supplement: Supplementary Material — Appendix C – Statistical analysis plan: 1) first version, 2) final version, 3) summary of SAP amendments, 4) issues identified in SAP, 5) memo to file to explain issues identified [file jpd-9-jpd191576-s003.pdf]

Statistical Analysis Plan

**Sponsor:**	North Bristol NHS Trust (NBT)
**Client:**	MedGenesis Therapeutix
**Protocol No./EudraCT No.:**	GDNF 2797 / 2013-001881-40
**Version No./Date**	1.4 (Incorporating Amendments 1-4) / 16-Dec-2015
**Title**	An Extension Study to Assess the Safety and Efficacy of Intermittent Bilateral Intraputamenal Glial Cell Line-Derived Neurotrophic Factor (GDNF) Infusions Administered via Convection Enhanced Delivery (CED) in Subjects with Parkinson's Disease
**PRA Project Id:**	MDGGNDNFD-GDNFDM
**SAP Version No./Date:**	Version 1.0 / 10-Feb-2017

Approvals

Principal Investigator (PI)

PI Affiliation: Department of Neurology and the Burden Institute
Movement Disorder Service,
Brain Centre,
Southmead Hospital, North Bristol NHS Trust
Bristol BS10 5NB, United Kingdom

PI Name, Title: Alan Whone, FRCP, PhD, Consultant Senior Lecturer and Hon Consultant Neurologist

Signature, Date: 15/Feb/2017

Sponsor

Sponsor Name: North Bristol NHS Trust (NBT)

Representative, Title: Rebecca Smith, PhD, Deputy Director of Research

Signature, Date: 21/FEB/2017

Client

Client Name: MedGenesis Therapeutix

Representative, Title: Lara Longpre, Chief Operating Officer

Signature, Date: 18FEB2017

Representative, Title: Matthias Luz, MD, Chief Medical Officer

Signature, Date: 10-Feb-2017

PRA

Project Manager, Title: Diana Soto, Project Manager

Signature, Date: 21-FEB-2017

Biostatistician, Title: Emma Lewis, Principal Biostatistician

Signature, Date: 21 FEB2017

Table of Contents

Approvals 2
Table of Contents 3
1.0 Introduction 5
1.1 Changes from Protocol 5
2.0 Study Objectives 7
2.1 Primary Objective 7
2.2 Secondary Objectives 7
2.3 Other Objectives 7
3.0 Study Design 7
3.1 Sample Size Considerations 16
3.2 Randomization 16
4.0 Study Endpoints 16
4.1 Efficacy Endpoints 16
4.1.1 Primary Efficacy Endpoint 16
4.1.2 Secondary Efficacy Endpoints 16
4.1.3 Supplementary Efficacy Endpoints 17
4.2 Imaging Endpoints 17
4.3 Safety Endpoints 18
5.0 Definitions 19
6.0 Analysis Populations 28
6.1 Intent-to-Treat Populations 28
6.1.1 ITT Primary Population 28
6.1.2 ITT Overall Population 28
6.2 Safety Population 28
6.2.1 Safety Overall Population 28
7.0 Interim Analyses 28
8.0 Data Review 28
8.1 Data Handling and Transfer 28
8.2 Data Screening 29
9.0 Statistical Methods 29
9.1 Missing Data Methods 29
9.1.1 Missing or Partial Dates 29
9.1.2 Missing Efficacy Data 30
9.1.3 Missing Imaging Data 30
9.1.4 Missing Data for Questionnaires 30
9.1.5 Missing Safety Data 30
9.1.6 Special Arrangements for Subject 45 30
9.2 Subject Disposition 30
9.3 Protocol Deviations 31
9.4 Demographic and Baseline Characteristics 31
9.4.1 Demographic Characteristics 31
9.4.2 Parkinson’s Disease History at Screening 31
9.5 Concomitant Medications 32
9.6 Surgery and Test Infusions 32
9.6.1 Catheter Trajectories and Positioning Accuracy 32

9.6.2 Contrast-Enhanced Test Infusions with T1-Weighted MRI Prior to Start of Study Medication 32
9.7 Efficacy Analyses 32
9.7.1 Analyses of Primary Efficacy Endpoint 33
9.7.2 Analyses of Secondary Efficacy Endpoints 34
9.7.3 Analyses of Supplementary Efficacy Endpoints 37
9.8 Imaging Analyses 40
9.8.1 MRI Analyses 40
9.8.2 Correlation Analyses 41
9.9 Safety Analyses 43
9.9.1 Study Medication Exposure 43
9.9.2 Adverse Events 43
9.9.3 Port Symptoms 44
9.9.4 Laboratory Data 44
9.9.5 Anti-GDNF Serum Antibodies 45
9.9.6 Plasma GDNF Concentrations 45
9.9.7 Physical Examination 45
9.9.8 Vital Signs 45
9.9.9 Weight 46
9.9.10 Electrocardiogram 46
9.9.11 Glasgow Coma Scale 47
9.9.12 Questionnaire for Impulsive-Compulsive Disorders 47
9.9.13 Montreal Cognitive Assessment 47
9.9.14 Mattis Dementia Rating Scale 48
9.9.15 Stroop Test 48
9.9.16 Frontal Systems Behavioural Scale 48
9.9.17 Deary-Liewald Reaction Time 49
9.9.18 Verbal Fluency Assessment 49
9.9.19 Beck Depression Inventory 49
9.9.20 University of Pennsylvania Smell Identification Test 49
10.0 Validation 50
Appendix 1 Glossary of Abbreviations 51
Appendix 2 Protocol Deviation Guidance 53
Appendix 3 List of MedDRA Preferred Terms for Adverse Events of Special Interest 55
Appendix 4 List of Conversion Factors for the Calculation of Levodopa and Levodopa Equivalent Doses 56
Appendix 5 List of Post-Text Tables, Figures and Listings 57
Appendix 6 Shells for Post-Text Tables, Figures and Listings 64
Document History 185

1.0 Introduction

This statistical analysis plan (SAP) describes the statistical methods to be used during the reporting and analyses of data collected under North Bristol NHS Trust (NBT) Protocol 2797.

This SAP should be read in conjunction with the study protocol and case report form (CRF). This version of the plan has been developed using the protocol version 1.4 (incorporating Amendments 1-4) dated 16DEC2015 and the corresponding CRF. Any further changes to the protocol or CRF may necessitate updates to the SAP.

The SAP was developed in two stages. The purpose was to “finalize” an SAP so that PRA could start programming earlier in the process. Versions of the SAP up to initial approval were known as SAP1. Changes following approval of SAP1 were tracked in the SAP Change Log and a final version of the SAP, known as SAP2, was issued for approval prior to database lock.

1.1 Changes from Protocol

No inferential analyses are described in the protocol. All inferential analyses described in the SAP will be interpreted in an exploratory manner only.

During the preparation of the SAP for the parent Study 2553, it was recognized that the wording of certain study endpoints was less clear than anticipated. In addition, a number of endpoints were added in order to provide for a more comprehensive analysis of the study data. The endpoints in Study 2797 have been refined in a similar manner to achieve consistency between the protocols and to improve the preciseness of the definitions. In addition, treatment response has been added as a secondary endpoint pursuant to the post-hoc analysis of Study 2553. Due to the open, uncontrolled design of the extension study, these changes have been implemented in the SAP without amending the protocol itself.

No change has been made to the primary efficacy endpoint.

The secondary efficacy endpoints have been reworded and expanded (including some endpoints originally classified as supplementary) as follows:

- Change from baseline to Week 80/e40 in OFF state UPDRS motor score (part III).
- Change and percentage change from baseline to Week 80/e40 in:
    - UPDRS motor score (part III) in the ON state (following a levodopa challenge).
    - UPDRS activities of daily living (ADL) score (part II) in the OFF state and in the ON state.
    - UPDRS total score (sum of motor + ADL scores) in the OFF state and in the ON state.
- Change from baseline to Week 40/e0 for the GDNF/GDNF group compared to change from baseline to Week 80/e40 for the placebo/GDNF group in:
    - OFF state UPDRS motor score (part III).
    - OFF state UPDRS ADL score (part II).
    - OFF state UPDRS total score (sum of motor + ADL scores).
- Change from baseline to Week 80/e40 for the GDNF/GDNF group compared to change from baseline to Week 40/e0 for the placebo/GDNF group in:
    - OFF state UPDRS motor score (part III).
    - OFF state UPDRS ADL score (part II).
    - OFF state UPDRS total score (sum of motor + ADL scores).

- Change from baseline to Week 80/e40 in PD diary ratings:
    - Total OFF time per day.
    - Total good-quality ON time per day (ON without dyskinesias or ON with non-troublesome dyskinesias).
    - ON time per day with troublesome dyskinesias.
- Treatment response based on the following criteria:
    - Decrease from baseline to Week 80/e40 by ≥10 points in OFF state UPDRS motor score (part III).
    - Increase from baseline to Week 80/e40 by ≥1 hour in total good-quality ON time per day (ON without dyskinesias or ON with non-troublesome dyskinesias).
    - Both of the above criteria, i.e. decrease from baseline to Week 80/e40 by ≥10 points in OFF state UPDRS motor score (part III) and increase from baseline to Week 80/e40 by ≥1 hour in total good-quality ON time per day (ON without dyskinesias or ON with non-troublesome dyskinesias).

The efficacy endpoint definitions provided in the SAP are independent of the corresponding analysis populations. The analysis populations for the individual efficacy endpoints are specified in Section 9.7.

The following imaging endpoints have been added:

- Change from baseline to Week 80/e40 in volume of distribution of infusate as determined by contrast-enhanced T1-weighted MRI.
- Change from baseline to Week 80/e40 in volume of interest (VOI) coverage and total putamenal coverage as determined by contrast-enhanced T1-weighted MRI.
- Correlation between primary study endpoint and VOI coverage and total putamenal coverage at baseline as determined by contrast-enhanced T1-weighted MRI.
- Correlation between primary study endpoint and change from baseline to Week 40/e0 in ^{18}F-DOPA uptake as determined by PET scan.
- Correlation between baseline OFF state UPDRS motor score (part III) and baseline ^{18}F-DOPA uptake as determined by PET scan.
- Correlation between baseline OFF state UPDRS ADL score (part II) and baseline ^{18}F-DOPA uptake as determined by PET scan.
- Correlation between Week 40/e0 OFF state UPDRS motor score (part III) and Week 40/e0 ^{18}F-DOPA uptake as determined by PET scan.
- Correlation between Week 40/e0 OFF state UPDRS ADL score (part II) and Week 40/e0 ^{18}F-DOPA uptake as determined by PET scan.
- Correlation between Week 80/e40 OFF state UPDRS motor score (part III) and Week 40/e0 ^{18}F-DOPA uptake as determined by PET scan.
- Correlation between Week 80/e40 OFF state UPDRS ADL score (part II) and Week 40/e0 ^{18}F-DOPA uptake as determined by PET scan.

The wording of the safety endpoints has been revised to provide more clarity and accuracy of the individual parameters to be analyzed. Time with troublesome dyskinesias (from subject diaries) has been removed from the list of safety endpoints since it is already listed as a secondary efficacy endpoint. Full brain MRI has been removed from the list as it is redundant with adverse changes in MRI findings. The new wording of the safety endpoints is shown in Section 4.3.

2.0 Study Objectives

2.1 Primary Objective

To compare the effects of intermittent bilateral intraputamenal GDNF infusions on OFF state motor function after 18 months of treatment with the effects after 9 months of treatment in subjects who completed Study 2553.

2.2 Secondary Objectives

- To compare the effects of intermittent bilateral intraputamenal GDNF infusions on ON state motor function, motor complications, and ON and OFF state activities of daily living (ADL) after 18 months of treatment with the effects after 9 months of treatment in subjects who completed Study 2553.
- To assess the safety of intermittent bilateral intraputamenal GDNF infusions at 18 months in subjects who received GDNF or placebo for 9 months in Study 2553.

2.3 Other Objectives

- To explore the effects of intermittent bilateral intraputamenal GDNF infusions on other motor and non-motor functions, quality of life (QOL) assessments, and imaging endpoints at 18 months in subjects who completed Study 2553.
- To compare the results for various motor outcomes between the subjects who started GDNF early (i.e. were randomized to GDNF in Study 2553) and those who started GDNF late (i.e. were randomized to placebo in Study 2553).
- Pilot and Supplemental Extensions: To generate long-term safety data and provide continued access to GDNF until the end of December 2016 when the results of Study 2553 are expected, which will inform interested parties with potential future studies.

3.0 Study Design

This is a phase II, single-center, open-label trial of intermittent bilateral intraputamenal GDNF infusions administered via convection-enhanced delivery (CED) in subjects with idiopathic PD who have completed Study 2553.

Following the final study visit at Week 40 in Study 2553, study completers return within one week to receive their first infusion of open-label GDNF. GDNF is administered using the same treatment protocol as in Study 2553. Treatment is given at 4-weekly intervals for 9 months (40 weeks; 10 infusions total). Hence, at 18 months, subjects receiving GDNF in Study 2553 have been treated with GDNF for a total of 18 months, while those receiving placebo in Study 2553 have been treated with GDNF for a total of 9 months.

Key clinical outcomes are measured at 8-week intervals throughout this initial 9-month extension (the “Initial Extension”). The Schedule of Events for this extension is shown in Table 1.

Pilot Stage subjects who complete the Initial Extension and provide informed consent are eligible for up to an additional 80 weeks of treatment with GDNF (the “Pilot Extension”). The Schedule of Events for this extension is shown in Table 2.

Pilot Stage subjects completing the Pilot Extension and Primary Stage subjects completing the Initial Extension who provide informed consent are eligible to enroll in a further extension (the “Supplemental Extension”) and continue to receive 4-weekly GDNF infusions until the end of December 2016. The Schedule of Events for this extension is shown in Table 3.

Figure 1 presents the study schema.

Although the statistical assessment of Study 2553 was performed before completion of the extension study, to reduce any potential for bias in this study, individual treatment codes from the parent study will not be disclosed to subjects until database lock for Study 2797, unless required for specific safety reasons. In addition, every effort will be made to avoid unblinding of the blinded UPDRS raters before database lock for Study 2797.

The primary analysis of the extension study is the intention-to-treat (ITT) analysis of the percentage change from baseline (in Study 2553) to Week 80/e40 in the practically defined OFF state UPDRS motor score (part III) in Primary Stage subjects.

Figure 1. Study Schema

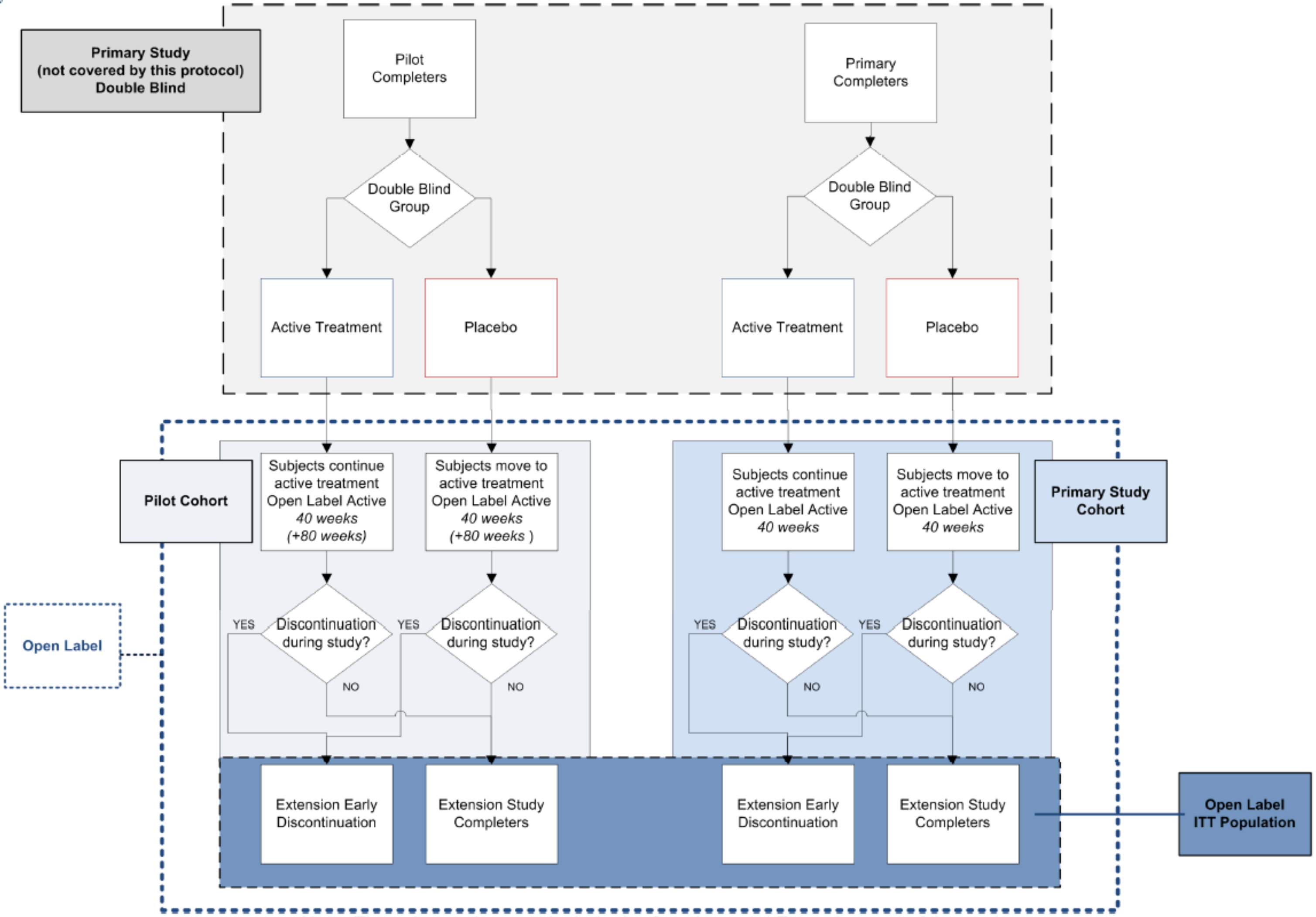

Note: The schema does not include the Supplemental Extension part of the study.

Table 1. Schedule of Events - Initial Extension

Procedure/Assessments	Week										
	40/e0[a]	44/e4	48/e8[b]	52/e12	56/e16[b]	60/e20	64/e24[b]	68/e28	72/e32[b]	76/e36	80/e40[b]
Informed consent[c]	(X)										
MRI[d]											X
Vital signs[e]	X	X	X	X	X	X	X	X	X	X	X
Weight and height	(X)		X		X		X		X		X
Physical examination[f]											X
Port review	X	X	X	X	X	X	X	X	X	X	X
ECG											X
Laboratory assessment[g]		X			X			X			X
Anti-GDNF serum antibody levels and GDNF plasma concentrations		X			X			X			X
UPDRS part II and part III in OFF state	(X)		X		X		X		X		X
Timed walking test in OFF state	(X)		X		X		X		X		X
Timed tapping test in OFF state	(X)		X		X		X		X		X
Levodopa challenge	(X)		X		X		X		X		X
UPDRS in ON state	(X)		X		X		X		X		X
Timed walking test in ON state	(X)		X		X		X		X		X
Timed tapping test in ON state	(X)		X		X		X		X		X
PDQ-39											X
EQ-5D											X
MoCA and MDRS					X						X
Stroop test											X
UPSIT											X
NMSS				X			X				X
BDI											X
QUIP	(X)		X		X		X		X		X
Deary-Liewald reaction time					X						X
SNAQ											X
FrSBe											X
Verbal fluency											X
Direct questioning of impulsivity, mood, falls and freezing for recording in case notes	X	X	X	X	X	X	X	X	X	X	X
Collect PD fluctuation diaries	(X)		X		X		X		X		X
Dispense PD fluctuation diaries		X		X		X		X		X	

**Procedure/Assessments**	**Week**										
	**40/e0[a]**	**44/e4**	**48/e8[b]**	**52/e12**	**56/e16[b]**	**60/e20**	**64/e24[b]**	**68/e28**	**72/e32[b]**	**76/e36**	**80/e40[b]**
Infusion of study drug	X	X	X	X	X	X	X	X	X	X	
Glasgow Coma Scale[h]	X	X	X	X	X	X	X	X	X	X	X
Adverse events[i]	X	X	X	X	X	X	X	X	X	X	X
Concomitant medications[j]	X	X	X	X	X	X	X	X	X	X	X

a. Assessments that are put in parentheses are done at the Week 40 visit in Study 2553 or earlier (informed consent). With the exception of informed consent, no procedures are required specifically for the extension study; however, procedures scheduled for Week 40 in Study 2553 must be performed in accordance with the protocol.
b. At Weeks 48/e8, 56/e16, 64/e24 and 72/e32, no PD medications are taken after 6:00 PM on the night before the assessments and no long-acting PD medications are taken on the day before the assessments. Subjects refrain from eating any high-protein foods on the morning of the assessments.
c. Informed consent must be obtained before any extension study-specific procedures or assessments are performed.
d. T1-weighted as well as T2-weighted and FLAIR 3T MRI are to be completed in Primary Stage subjects within 2 hours of a gadolinium contrast-containing test infusion at Week 80/e40 (±1 week). If possible, the test infusion and MRI should be done 1 week before the visit.
e. For times of assessment of vital signs, see protocol.
f. Brief physical examination, targeted, at the Investigator's discretion, to identify changes from baseline and from the previous assessment.
g. Hematology, serum chemistry, urinalysis, pregnancy test. For individual tests, see protocol.
h. Performed before infusion, 30 minutes into the infusion, and after completion of the infusion.
i. All AEs occurring during the study until 28 days after the last dose of GDNF are recorded on the AE pages of the CRF.
j. Concomitant medications: from study entry and until Week 80/e40 or early discontinuation are recorded. All over-the-counter or prescription medications, vitamins and/or herbal supplements are recorded in the CRF with their indications.

Table 2. Schedule of Events - Pilot Extension

**Procedure/Assessments**	**Week in Pilot Extension**																				
	**e2-0[a]**	**e2-4**	**e2-8**	**e2-12**	**e2-16[b]**	**e2-20**	**e2-24**	**e2-28**	**e2-32[b]**	**e2-36**	**e2-40**	**e2-44**	**e2-48**	**e2-52**	**e2-56[b]**	**e2-60**	**e2-64**	**e2-68**	**e2-72**	**e2-76**	**e2-80[b]**
Informed consent[c]	(X)																				
MRI[d]	X																				X
Vital signs[e]	X	X	X	X	X	X	X	X	X	X	X	X	X	X	X	X	X	X	X	X	X
Weight and height							X								X						X
Physical examination[f]																					X
Port review	X	X	X	X	X	X	X	X	X	X	X	X	X	X	X	X	X	X	X	X	X
ECG																					X
Laboratory assessment[g]																					X
Anti-GDNF serum antibody levels and GDNF plasma concentrations							X						X						X		
UPDRS part II and part III in OFF state	(X)				X				X						X						X
Timed walking test in OFF state	(X)				X				X						X						X
Timed tapping test in OFF state	(X)				X				X						X						X
Levodopa challenge	(X)				X				X						X						X
UPDRS in ON state	(X)				X				X						X						X
Timed walking test in ON state	(X)				X				X						X						X
Timed tapping test in ON state	(X)				X				X						X						X
MoCA and MDRS	(X)																				X
UPSIT	(X)																				X
NMSS	(X)										X										X
BDI	(X)																				X
QUIP	(X)				X				X				X				X				X
Direct questioning of impulsivity, mood, falls and freezing for recording in case notes	X	X	X	X	X	X	X	X	X	X	X	X	X	X	X	X	X	X	X	X	X
Collect PD fluctuation diaries	(X)				X				X						X						X
Dispense PD fluctuation diaries				X				X						X						X	
Infusion of study drug		X	X	X	X	X	X	X	X	X	X	X	X	X	X	X	X	X	X	X	
Glasgow Coma Scale[h]	X	X	X	X	X	X	X	X	X	X	X	X	X	X	X	X	X	X	X	X	X
Adverse events[i]	X	X	X	X	X	X	X	X	X	X	X	X	X	X	X	X	X	X	X	X	X
Concomitant medications[j]	X	X	X	X	X	X	X	X	X	X	X	X	X	X	X	X	X	X	X	X	X

a. Visit e2-0 takes place at the same time as (or within 1 week of) the Week 80/e40 visit. Assessments in parentheses are done at Week 80/e40 or earlier (informed consent). The safety assessments and outcome measures taken at Week 80/e40 serve as the baseline for this part of the study and do not need to be repeated at Visit e2-0.
b. At Weeks e2-16, e2-32, e2-56 and e2-80, no PD medications are taken after 6:00 PM on the night before the assessments and no long-acting PD medications are taken on the day before the assessments. Subjects refrain from eating any high-protein foods on the morning of the assessments.
c. Informed consent must be obtained before any extension study-specific procedures or assessments are performed.
d. T1-weighted as well as T2-weighted and FLAIR 3T MRI are to be completed in Primary Stage subjects within 2 hours of a gadolinium contrast-containing test infusion at Weeks e2-0 and e2-80 (±1 week). At the Week e2-80 visit, the test infusion and MRI should be done 1 week before the visit, if possible.
e. For times of assessment of vital signs, see protocol.
f. Brief physical examination, targeted, at the Investigator’s discretion, to identify changes from baseline and from the previous assessment.
g. Hematology, serum chemistry, urinalysis, pregnancy test. For individual tests, see protocol.
h. Performed before infusion, 30 minutes into the infusion, and after completion of the infusion.
i. All AEs occurring during the study until 28 days after the last dose of GDNF are recorded on the AE pages of the CRF.
j. Concomitant medications from study entry and until Week e2-80 or early discontinuation are recorded. All over-the-counter or prescription medications, vitamins and/or herbal supplements are recorded in the CRF with their indications.

Table 3. Schedule of Events - Supplemental Extension

Procedure/Assessments	Week in Supplemental Extension													
	e3-0[a]	e3-4	e3-8	e3-12	e3-16[b]	e3-20	e3-24	e3-28	e3-32[b]	e3-36	e3-40	e3-44	e3-48[c]	Last Study Visit Additional procedures/assessments[c]
Informed consent[d]	(X)													
Vital signs[e]	X	X	X	X	X	X	X	X	X	X	X	X	X	
Port review	X	X	X	X	X	X	X	X	X	X	X	X	X	
Laboratory assessment[f]														X
Anti-GDNF serum antibody levels and GDNF plasma concentrations														X
UPDRS part II and part III in OFF state	(X)				X				X					
Timed walking test in OFF state	(X)				X				X					
Timed tapping test in OFF state	(X)				X				X					
Levodopa challenge	(X)				X				X					
UPDRS in ON state	(X)				X				X					
Timed walking test in ON state	(X)				X				X					
Timed tapping test in ON state	(X)				X				X					
MoCA and MDRS														X
BDI	(X)													X
QUIP	(X)				X				X					X
Direct questioning of impulsivity, mood, falls and freezing for recording in case notes	X	X	X	X	X	X	X	X	X	X	X	X	X	
Collect PD fluctuation diaries	(X)				X				X					
Dispense PD fluctuation diaries				X				X						
Infusion of study drug	X	X	X	X	X	X	X	X	X	X	X	X	X	
Glasgow Coma Scale[g]	X	X	X	X	X	X	X	X	X	X	X	X	X	
Adverse event[h]	X	X	X	X	X	X	X	X	X	X	X	X	X	
Concomitant medications[i]	X	X	X	X	X	X	X	X	X	X	X	X	X	

a. Visit e3-0 takes place approximately 1-2 weeks after Week 80/e40 (Primary Stage subjects) or Week e2-80 (Pilot Stage subjects). Assessments in parentheses are done at Week 80/e40 (Primary Stage subjects) or Week e2-80 (Pilot Stage subjects) or earlier (informed consent). The safety assessments and outcome measures taken at Week 80/e40 or Week e2-80 (whichever is applicable) serve as the baseline for this part of the study and do not need to be repeated at Visit e3-0.

b. At Weeks e3-16 and e3-32, no PD medications are taken after 6:00 PM on the night before the assessments and no long-acting PD medications are taken on the day before the assessments. Subjects refrain from eating any high-protein foods on the morning of the assessments.
c. At the last study visit in December 2016 (which occurs at Week e3-48 or earlier), the subject undergoes all procedures and assessments scheduled for the respective visit reached by the subject as per the visit schedule. In addition, regardless of the assessments scheduled for the respective visit, MoCA, MDRS, QUIP and BDI assessments are performed and samples are obtained for laboratory assessment and determination of anti-GDNF serum antibody levels and GDNF plasma concentrations. The same approach to final assessments should be taken, if possible, for any subjects who discontinue the Supplemental Extension early.
d. Informed consent must be obtained before any extension study-specific procedures or assessments are performed.
e. For times of assessment of vital signs, see protocol.
f. Hematology, serum chemistry, urinalysis, pregnancy test. For individual tests, see protocol.
g. Performed before infusion, 30 minutes into the infusion, and after completion of the infusion.
h. All AEs occurring during the study until 28 days after the last dose of GDNF are recorded on the AE pages of the CRF.
i. Concomitant medications: from study entry and until last study visit are recorded. All over-the-counter or prescription medications, vitamins and/or herbal supplements are recorded in the CRF with their indications.

Abbreviations used in the tables

AE: Adverse event; BDI: Beck Depression Inventory; CRF: Case report form; ECG: Electrocardiogram; EQ-5D: EuroQOL 5-Dimensional Scale; FLAIR: Fluid-attenuated inversion recovery; FrSBe: Frontal Systems Behavioural Scale; GDNF: Glial cell line-derived neurotrophic factor; MDRS: Mattis Dementia Rating Scale; MoCA: Montreal Cognitive Assessment; MRI: Magnetic resonance imaging; NMSS: Non-Motor Symptom Scale; PD: Parkinson's disease; PDQ-39: Parkinson's Disease Questionnaire-39; QUIP: Questionnaire for Impulsive-Compulsive Disorders in Parkinson's Disease; SNAQ: Simplified Nutritional Appetite Questionnaire; UPDRS: Unified Parkinson's Disease Rating Scale; UPSIT: University of Pennsylvania Smell Identification Test.

3.1 Sample Size Considerations

This is an open-label extension study. No power calculations were performed. The study was open to all 41 subjects who completed Study 2553 and met all eligibility criteria specified in the protocol.

3.2 Randomization

This study is not randomized. All subjects enrolled receive active treatment: 600 µL of 0.20 µg/µL GDNF in artificial cerebrospinal fluid (aCSF) per putamen every 4 weeks, regardless of their treatment assignment in Study 2553.

4.0 Study Endpoints

4.1 Efficacy Endpoints

Efficacy endpoints are analyzed only for the period up to the end of the Initial Extension. Efficacy data collected during the Pilot Extension and Supplemental Extension are included in the subject listings.

Depending on the individual endpoint, efficacy analyses are performed for both the ITT Primary Population and the ITT Overall Population, or for the ITT Overall Population alone (see Section 9.7 for details). The primary analysis is the analysis of the primary efficacy endpoint in the ITT Primary Population.

4.1.1 Primary Efficacy Endpoint

The primary efficacy endpoint of the study is:

- Percentage change from baseline to Week 80/e40 in the practically defined OFF state UPDRS motor score (part III).

4.1.2 Secondary Efficacy Endpoints

The secondary efficacy endpoints are:

- Change from baseline to Week 80/e40 in OFF state UPDRS motor score (part III).
- Change and percentage change from baseline to Week 80/e40 in:
    - UPDRS motor score (part III) in the ON state (following a levodopa challenge).
    - UPDRS activities of daily living (ADL) score (part II) in the OFF state and in the ON state.
    - UPDRS total score (sum of motor + ADL scores) in the OFF state and in the ON state.
- Change from baseline to Week 40/e0 for the GDNF/GDNF group compared to change from baseline to Week 80/e40 for the placebo/GDNF group in:
    - OFF state UPDRS motor score (part III).
    - OFF state UPDRS ADL score (part II).
    - OFF state UPDRS total score (sum of motor + ADL scores).
- Change from baseline to Week 80/e40 for the GDNF/GDNF group compared to change from baseline to Week 40/e0 for the placebo/GDNF group in:
    - OFF state UPDRS motor score (part III).
    - OFF state UPDRS ADL score (part II).
    - OFF state UPDRS total score (sum of motor + ADL scores).

- Change from baseline to Week 80/e40 in PD diary ratings:
    - Total OFF time per day.
    - Total good-quality ON time per day (ON without dyskinesias or ON with non-troublesome dyskinesias).
    - ON time per day with troublesome dyskinesias.
- Treatment response based on the following criteria:
    - Decrease from baseline to Week 80/e40 by ≥10 points in OFF state UPDRS motor score (part III).
    - Increase from baseline to Week 80/e40 by ≥1 hour in total good-quality ON time per day (ON without dyskinesias or ON with non-troublesome dyskinesias).
    - Both of the above criteria, i.e. decrease from baseline to Week 80/e40 by ≥10 points in OFF state UPDRS motor score (part III) and increase from baseline to Week 80/e40 by ≥1 hour in total good-quality ON time per day (ON without dyskinesias or ON with non-troublesome dyskinesias).

4.1.3 Supplementary Efficacy Endpoints

The following are supplementary efficacy endpoints:

- Change from baseline to Week 80/e40 in supplementary motor, non-motor, medication, and quality of life endpoints, including the following
    - Timed walking test (OFF and ON state).
    - Timed tapping test (OFF and ON state).
    - Non-Motor Symptom Assessment Scale for PD (NMSS).
    - Parkinson’s Disease Questionnaire-39 (PDQ-39).
    - EuroQOL 5-Dimensional Scale (EQ-5D).
    - Simplified Nutritional Appetite Questionnaire (SNAQ).
    - Total daily dose of levodopa and total daily levodopa equivalent dose.

4.2 Imaging Endpoints

The analysis of imaging endpoints is performed for the ITT Primary Population and/or ITT Overall Population as specified below.

The following are analyzed as imaging endpoints:

- Change from baseline to Week 80/e40 in volume of distribution of infusate as determined by contrast-enhanced T1-weighted MRI. This analysis will be done for the ITT Primary Population.
- Change from baseline to Week 80/e40 in volume of interest (VOI) coverage and total putamenal coverage as determined by contrast-enhanced T1-weighted MRI. This analysis will be done for the ITT Primary Population.
- Correlation between primary study endpoint and VOI coverage and total putamenal coverage at baseline as determined by contrast-enhanced T1-weighted MRI. This analysis will be done for the ITT Primary Population.

- Correlation between primary study endpoint and change from baseline to Week 40/e0 in ^{18}F-DOPA uptake as determined by PET scan. This analysis will be done for both the ITT Primary Population and the ITT Overall Population.
- Correlation between baseline OFF state UPDRS motor score (part III) and baseline ^{18}F-DOPA uptake as determined by PET scan. This analysis will be done for the ITT Overall Population only.
- Correlation between baseline OFF state UPDRS ADL score (part II) and baseline ^{18}F-DOPA uptake as determined by PET scan. This analysis will be done for the ITT Overall Population only.
- Correlation between Week 40/e0 OFF state UPDRS motor score (part III) and Week 40/e0 ^{18}F-DOPA uptake as determined by PET scan. This analysis will be done for the ITT Overall Population only.
- Correlation between Week 40/e0 OFF state UPDRS ADL score (part II) and Week 40/e0 ^{18}F-DOPA uptake as determined by PET scan. This analysis will be done for the ITT Overall Population only.
- Correlation between Week 80/e40 OFF state UPDRS motor score (part III) and Week 40/e0 ^{18}F-DOPA uptake as determined by PET scan. This analysis will be done for the ITT Overall Population only.
- Correlation between Week 80/e40 OFF state UPDRS ADL score (part II) and Week 40/e0 ^{18}F-DOPA uptake as determined by PET scan. This analysis will be done for the ITT Overall Population only.

4.3 Safety Endpoints

The analysis of safety endpoints includes all subjects enrolled who received at least one dose of open-label study medication (Safety Overall Population; see Section 6.2.1). Safety endpoints are reported for the entire study period including the Initial Extension, Pilot Extension and/or Supplemental Extension as applicable (see Schedules of Events in Section 3.0 for assessment time points). Due to the temporal proximity of the study start to the end of Study 2553, all adverse events (AEs) are considered treatment-emergent AEs (TEAEs).

The following are analyzed as safety endpoints:

- Frequency of TEAEs (all TEAEs and TEAEs related to study drug) during the study period.
- Frequency of device-related TEAEs during the study period.
- Frequency of dyskinesias, falls, adverse changes in mood, and impulsivity reported as TEAEs during the study period (AEs of special interest, AESIs).
- Adverse changes in MRI findings as captured by AE reporting.
- Results of routine laboratory blood tests (hematology, serum chemistry) and urinalysis performed during the study period
- Frequency of subjects with anti-GDNF serum antibodies during the study period.
- Change from baseline in the Questionnaire for Impulsive-Compulsive Disorders in Parkinson’s Disease (QUIP) as assessed during the study period.
- Change from baseline in the Montreal Cognitive Assessment (MoCA) as assessed during the study period.
- Change from baseline in the Mattis Dementia Rating Scale (MDRS) as assessed during the study period.

The following other safety data are also analyzed or listed:

- Exposure to study medication.
- Plasma GDNF concentrations.
- Physical examination.
- Port symptoms.
- Vital signs.
- Weight and height.
- Electrocardiogram (ECG).
- Glasgow Coma Scale.
- Stroop test.
- Frontal Systems Behavioural Scale (FrSBe).
- Deary-Liewald reaction time (RT).
- Verbal fluency assessment.
- Beck Depression Inventory (BDI).
- University of Pennsylvania Smell Identification Test (UPSIT).

5.0 Definitions

Adverse changes in MRI findings

Adverse changes in MRI findings as captured by AE reporting are defined as a Medical Dictionary for Regulatory Activities (MedDRA) preferred term of “Nuclear magnetic resonance imaging brain abnormal” (MedDRA higher level term “Central nervous system imaging procedures”).

Adverse events of special interest

TEAEs including dyskinesias, falls, adverse changes in mood, and impulsivity are considered AESIs in this study. AESIs are defined as follows.

Dyskinesias

Dyskinesia is defined as any of the following MedDRA preferred terms:

- Dyskinesia
- Chorea
- Ballism
- Athetosis
- Dystonia

Falls

A fall is defined as a MedDRA preferred term of “Fall.”

Adverse changes in mood / impulsivity

Prior to database lock, all AE data were reviewed by a qualified physician to identify any relevant MedDRA preferred terms for the categories “Adverse changes in mood” and “Impulsivity”. The MedDRA preferred terms found are listed in Appendix 3.

Age

The following SAS® code will be used to calculate subject age (years) at baseline in Study 2553:

Age = floor ((intck(‘month’, birth date, IC date) - (day(IC date) < day(birth date))) / 12),

where intck is a SAS® function counting integer days, birth date is the database variable for date of birth, and informed consent (IC) date is the database variable for initial informed consent date in Study 2553.

Baseline, change from baseline, percentage change from baseline

Baseline values for comparisons with postbaseline values

For efficacy and imaging analyses, for comparisons of postbaseline values to baseline values, the baseline value is defined as the baseline value from Study 2553.

For laboratory data, Glasgow Coma Scale and QUIP analyses, the baseline value is defined as the value collected at Week 40/e0. If data was collected at both Week 40 from Study 2553 and Week e0, then the latter value will be used as the baseline value.

For all other safety analyses, the baseline value is defined as the baseline value from Study 2553.

Pre-infusion baseline values for comparison with values during or after infusion

For comparisons of values during or after infusion with pre-infusion values, the baseline is the pre-infusion value. This type of comparison applies to vital sign assessments.

Change from baseline

Change from baseline is defined as (postbaseline value – value at baseline).

Percentage change from baseline

Percentage change from baseline is defined as [(postbaseline value – value at baseline) / value at baseline] * 100%.

Body mass index

Body mass index (BMI) is calculated as kg/m^2 where kg is weight in kilograms and m^2 is height in meters, squared.

Catheter positioning accuracy

There are 4 catheters per subject (2 catheters per putamen). Catheter positioning accuracy is assessed at repeat surgeries by measurement of the actual target versus the planned target in mm for the tip of each catheter. This parameter is not derived, but is located in the Post-Operative CT Scan CRF as “Distance between planned target and actual target (mm)” for catheters #1-4 for each subject.

Completion of study

A subject who completes the study is identified as such on the End of Study CRF in the database. This relates to the completion of the Initial Extension only.

Concomitant medication

Concomitant medications (Parkinson’s disease medications and other medications) are defined as any medications ongoing at the start of open-label study medication dosing or with a start date on or after the first open-label study medication dose date. In the case of missing or partial dates, any medications that

could have been ongoing at the start of open-label study medication dosing or could have started on or after the first open-label study medication dose date are assumed to be concomitant.

Duration of infusion of open-label study medication

Duration of infusion of open-label study medication in minutes is calculated as (infusion end time – infusion start time + 1). Interruptions are not subtracted.

Duration since first Parkinson's disease symptom, duration since Parkinson's disease diagnosis

Duration since PD symptom onset and duration since PD diagnosis in years in Study 2553 is calculated as (screening Visit 1 date – initial PD symptom/diagnosis date + 1)/365.25. If the day only of PD symptom/diagnosis date is missing, then the 1st day of the month is imputed; if the month only is missing or month and day are missing, then January or January 1st is imputed, respectively. PD symptom/diagnosis dates with a missing year are not included in the calculations.

Early termination of study

A subject who terminates the study prior to the completion of the Initial Extension is identified as such on the End of Study CRF in the database; a primary reason for early termination is provided. No early termination data are presented for the Pilot Extension or Supplemental Extension.

Enrolled subject

An enrolled subject is one with a record in the database who meets all of the inclusion/exclusion criteria for Study 2797.

Levodopa challenge dose

The levodopa challenge dose is the dose of levodopa in mg that the subject receives when undergoing a levodopa challenge.

Levodopa dose, total daily levodopa dose

The levodopa dose at baseline and Week 80/e40 is the total daily levodopa dose in mg that the subject is on at the time of the visit.

The actual daily doses of the individual levodopa preparations taken are documented on the Levodopa and Levodopa Equivalent Medications CRF. Since the bioavailability of levodopa preparations differs, specific conversion factors must be used in order to characterize the subject's effective levodopa dose (see Appendix 4). Immediate release preparations taken without concomitant catechol-O-methyl transferase (COMT) inhibitors do not require conversion (conversion factor 1.0). The daily doses of immediate release preparations taken with COMT inhibitors and of controlled release preparations are multiplied by the corresponding conversion factors. The total daily levodopa dose is then calculated by adding together the converted daily doses of all individual levodopa-containing preparations. COMT inhibitor doses are not included in the calculation.

Levodopa equivalent dose, total daily levodopa equivalent dose

Subjects with PD generally take numerous medications to control their symptoms. In order to have a measure of their total medication intake, a levodopa equivalent dose is calculated. Each PD medication, as documented on the Levodopa and Levodopa Equivalent Medications CRF, is multiplied by a specific conversion factor indicating the drug's relative potency with respect to immediate release levodopa unaccompanied by COMT inhibitors (see Appendix 4 for a full list of conversion factors). The total daily levodopa equivalent dose is calculated by adding together the daily levodopa equivalent doses of all individual PD medications. COMT inhibitor doses are not included in the calculation.

Measures of infusion performance

Measures of infusion performance are determined by hemisphere on the basis of contrast-enhanced T1-weighted MRI.

Volume of distribution

Volume of distribution per hemisphere is documented as “Volume of distribution (mL), left” and “Volume of distribution (mL), right” in the Post-Infusion MRI CRF.

Total volume of putamen

Total volume of putamen is documented as “Total volume of putamen (mL), left” and “Total volume of putamen (mL), right” in the Post Randomization MRI Review CRF (source: Baseline and Planning MRI CRF) from Study 2553.

Putamenal volume of distribution

Putamenal volume of distribution is documented as “Volume of distribution (mL), left putamen” and “Volume of distribution (mL), right putamen”. The baseline value is located in the Post Randomization MRI Review CRF from Study 2553. The Week 80/e40 value is located in the Post-Infusion MRI CRF, Extension Week 40.

Total putamenal coverage

Total putamenal coverage is defined as (putamenal volume of distribution / total volume of putamen * 100%). This parameter is derived for each putamen.

Volume of interest

Volume of interest is documented as “Volume of interest (mL), left” and “Volume of interest (mL), right” in the Post Randomization MRI Review CRF (source: Baseline and Planning MRI CRF) from Study 2553.

Volume of interest coverage (absolute)

Absolute VOI coverage is documented as “Volume of interest covered by infusate (mL), left” and “Volume of interest covered by infusate (mL), right”. The baseline value is taken from the Post Randomization MRI Review CRF (source: Post-Infusion MRI CRF, Healing Phase) from Study 2553. The Week 80/e40 value is located in the Post-Infusion MRI CRF, Extension Week 40.

Volume of interest coverage (relative)

Relative VOI coverage is defined as (volume of interest covered by infusate / volume of interest * 100%). This parameter is documented as “Volume of interest covered by infusate (%), left” and “Volume of interest covered by infusate (%), right”. The baseline value is taken from the Post Randomization MRI Review CRF (source: Post-Infusion MRI CRF, Healing Phase) from Study 2553. The Week 80/e40 value is located in the Post-Infusion MRI CRF, Extension Week 40.

Protocol deviations

Protocol deviations are recorded on the protocol deviation form. They are categorized for summarization, applying controlled terminology including inclusion criteria, exclusion criteria, study medication (including overdose), non-study medication, study schedule/visit window, outcome assessment, and other). They are also classified as major or minor, based on whether they potentially impact the outcome of the study. See Appendix 2 for further details. Prior to database lock, the database entries for protocol deviations will be reviewed by an adjudication team (including, at a minimum, the PI, the study statistician and the Chief Medical Officer of MedGenesis Therapeutix) for consistency of the categorizations and classifications. Final determination of the classifications (major or minor) will be made by the Study Sponsor in view of the recommendations made by the adjudication team.

Study day, last visit on study

If the assessment date is prior to the first open-label study medication dose date then the study day is calculated as (assessment date – first open-label study medication dose date); if the assessment date is on or after the first open-label study medication dose date then the study day is calculated as

(assessment date – first open-label study medication dose date + 1). Per Clinical Data Interchange Standards Consortium (CDISC) Standard Data Tabulation Model (SDTM) conventions, study Day 1 corresponds to the day of the first open-label study medication dose (ie, Week e0 visit).

The last visit on study is the last study visit attended including the Initial Extension, Pilot Extension and Supplemental Extension.

Total exposure to open-label study medication

Total exposure to open-label study medication in mg is calculated as (number of infusions * 0.240 mg). This calculation assumes that the entire dose was infused at each administration. This will be derived for the Initial Extension, Pilot Extension, Supplemental Extension and overall.

Total good-quality ON time per day

Total good-quality ON time per day is defined as the sum of ON time per day without dyskinesias + ON time per day with non-troublesome dyskinesias from the PD diary, where each half-hour interval checked contributes 30 minutes to the sum.

Treatment-emergent adverse event

Due to the temporal proximity of the start of Study 2797 to the end of Study 2553, all AEs reported during the study period are considered TEAEs, regardless of whether their onset was before, on, or after the first open-label study medication dose date. TEAEs that are present or ongoing at the beginning of Study 2797 (date of consent or date of last visit in Study 2553, if consent was obtained earlier) are considered pre-existing, if the event term, severity, and date and time of onset in Study 2797 are identical to the corresponding information given for the TEAE in Study 2553. If any of these conditions is not met, the TEAE is considered new or worsening.

Treatment group

In the analysis, findings are organized by “treatment group”. The names of the treatment groups reflect the randomized treatment (GDNF or placebo) received in Study 2553 followed by the non-randomized GDNF treatment received in Study 2797. Subjects included in the GDNF/GDNF treatment group are those that received randomized double-blind GDNF in Study 2553, followed by open-label GDNF in Study 2797. Subjects included in the placebo/GDNF treatment group are those that received randomized double-blind placebo in Study 2553, followed by open-label GDNF in Study 2797.

Treatment response

Treatment response is defined based on the change in OFF state UPDRS motor score (part III), total good quality ON time per day, and a composite of both. Specifically, the following response definitions apply:

- OFF state UPDRS motor score (part III): Decrease from baseline to Week 80/e40 by ≥10 points.
- Total good-quality ON time per day (ON without dyskinesias or ON with non-troublesome dyskinesias): Increase from baseline to Week 80/e40 by ≥1 hour.
- Composite: Decrease from baseline to Week 80/e40 by ≥10 points in OFF state UPDRS motor score (part III) and increase from baseline to Week 80/e40 by ≥1 hour in total good quality ON time per day (ON without dyskinesias or ON with non troublesome dyskinesias).

UPDRS score, OFF state total and ON state total

OFF state UPDRS total score is the sum of the OFF state motor score (part III) and the OFF state ADL score (part II).

ON state UPDRS total score is the sum of the ON state motor score (part III) and the ON state ADL score (part II).

Visits and visit windows

Scheduled visits in Study 2553 were as follows:

- Screening Visit 1
- Screening Visit 2
- Surgery and healing phase
- Week 0 / baseline
- [Week 2 prior to Study 2553 Amendment 3]
- Week 4
- [Week 6 prior to Study 2553 Amendment 3]
- Week 8
- [Week 10 prior to Study 2553 Amendment 3]
- Week 12
- [Week 14 prior to Study 2553 Amendment 3]
- Week 16
- [Week 18 prior to Study 2553 Amendment 3]
- Week 20
- [Week 22 prior to Study 2553 Amendment 3]
- Week 24
- [Week 26 prior to Study 2553 Amendment 3]
- Week 28
- [Week 30 prior to Study 2553 Amendment 3]
- Week 32
- [Week 34 prior to Study 2553 Amendment 3]
- Week 36
- [Week 38 prior to Study 2553 Amendment 3]
- Week 40 (Month 9 endpoint)/early termination

Scheduled visits in the Initial Extension of Study 2797 are as follows:

- Week e0 (same as Study 2553 Week 40 visit for many assessments)
- Week 44/e4
- Week 48/e8
- Week 52/e12
- Week 56/e16
- Week 60/e20

- Week 64/e24
- Week 68/e28
- Week 72/e32
- Week 76/e36
- Week 80/e40/early termination

The designation of the visits in the Initial Extension has been modified from the protocol by adding the consecutive week number from Study 2553 baseline to facilitate the interpretaion of the analyses.

In the Initial Extension, the Week 80/e40/early termination visit may occur at any time on or after study Day 1 for early terminators of the study. For subjects who terminate from the study early, early termination assessments are assigned to an earlier scheduled visit using the study day of the early termination visit date. If only the day of the early termination visit date is missing, then the 1st day of the month is imputed. If the early termination visit date has missing month and/or year after the data query process, the early termination assessments are not assigned to an earlier visit.

The early termination visit may occur during a visit period in which a scheduled visit has already occurred. In this case, the visit that is closer to the nominal study day is selected for tabulations and plots by visit.

Scheduled visits for the Pilot Extension of Study 2797 are as follows:

- Week e2-0 (at same time or within 1 week of Week e40)
- Week e2-4
- Week e2-8
- Week e2-12
- Week e2-16
- Week e2-20
- Week e2-24
- Week e2-28
- Week e2-32
- Week e2-36
- Week e2-40
- Week e2-44
- Week e2-48
- Week e2-52
- Week e2-56
- Week e2-60
- Week e2-64
- Week e2-68
- Week e2-72
- Week e2-76

- Week e2-80

Scheduled visits for the supplemental extension are as follows:

- Week e3-0 (1-2 weeks after Week e40 [Primary Stage subjects] or Week e2-80 [Pilot Stage subjects])
- Week e3-4
- Week e3-8
- Week e3-12
- Week e3-16
- Week e3-20
- Week e3-24
- Week e3-28
- Week e3-32
- Week e3-36
- Week e3-40
- Week e3-44
- Week e3-48

The visit windows in Table 4 are applied for analyses of the Initial Extension of Study 2797 in conjunction with data from Study 2553 (see definitions of completion of study, early termination of study, and study day in this section). The visit schedules are distinguished using either database visit labels for Pilot Stage subjects or by study day for Primary Stage subjects for Study 2553 visits. The visit schedules are distinguished using study day for all subjects for Study 2797 visits.

Table 4. Visit Windows

Visit	Nominal Study Day	Study Day Range Prior to Study 2553 Amendment 3	Study Day Range After Study 2553 Amendment 3	Study Day Range for Study 2797
Week 0	0	-6-7	-13-14	
Week 2	14	8-21		
Week 4	28	22-35	15-42	
Week 6	42	36-49		
Week 8	56	50-63	43-70	
Week 10	70	64-77		
Week 12	84	78-91	71-98	
Week 14	98	92-105		
Week 16	112	106-119	99-126	
Week 18	126	120-133		
Week 20	140	134-147	127-154	
Week 22	154	148-161		
Week 24	168	162-175	155-182	
Week 26	182	176-189		
Week 28	196	190-203	183-210	
Week 30	210	204-217		
Week 32	224	218-231	211-238	
Week 34	238	232-245		
Week 36	252	246-259	239-259	
Week 38	266	260-273		
Week 40	280	274-287	260-304	
Week e0	1			-13-14
Week 44/e4	29			15-42
Week 48/e8	57			43-70
Week 52/e12	85			71-98
Week 56/e16	113			99-126
Week 60/e20	141			127-154
Week 64/e24	169			155-182
Week 68/e28	197			183-210
Week 72/e32	225			211-238
Week 76/e36	252			239-259
Week 80/e40	281			260-304

The Week 40 and Week 80/e40 visit windows are wider than for other visits since the number and duration of assessments required special arrangements in some cases in order to minimize subject burden. As a consequence, the adjacent windows (Week 36 and Week 76/e36) are smaller.

Other than the Week 40/e0/early termination and Week 80/e40/early termination visits, postbaseline unscheduled visit values are not windowed and are excluded from tabulations by visit. All unscheduled visit values are included in data listings.

6.0 Analysis Populations

Enrolled subjects are defined in Section 5.0.

6.1 Intent-to-Treat Populations

6.1.1 ITT Primary Population

The ITT Primary Population is defined as all enrolled Primary Stage subjects. This population is used for analyses of the primary efficacy endpoint, some secondary efficacy endpoints, and all imaging endpoints. It is also used for tabulation of subject disposition and summaries of demographic and baseline characteristics from Study 2553. Subjects are counted according to their randomized treatment group in Study 2553.

6.1.2 ITT Overall Population

The ITT Overall Population is defined as all enrolled Pilot Stage subjects plus all enrolled Primary Stage subjects. This population is used for analyses of all efficacy endpoints and some correlation imaging endpoints, for tabulation of subject disposition, and for summaries of demographic and baseline characteristics from Study 2553. Subjects are counted according to their randomized treatment group in Study 2553.

6.2 Safety Population

6.2.1 Safety Overall Population

The Safety Overall Population is defined as all enrolled Pilot Stage subjects who received at least one dose of open-label study medication in Study 2797 plus all enrolled Primary Stage subjects who received at least one dose of open-label study medication in Study 2797. This population is used for all safety analyses. Subjects are counted according to the treatment actually received in Study 2553.

7.0 Interim Analyses

No interim analysis is planned for the study.

8.0 Data Review

8.1 Data Handling and Transfer

Data management for this study is performed by PRA. PRA performs data processing according to approved procedures including database specifications, CRF tracking, and dictionary coding and data validation. A quality control of site responses to data queries is also performed.

Data are entered by the investigational site into CRFs, which are entered by PRA into a clinical database built with Oracle Clinical version 4.5.3 and exported as SAS® version 9.4 or higher datasets (SAS Institute, Inc., Cary, NC). Converted datasets are created using SAS® and following CDISC SDTM conventions (v3.1.3 implementation guide v1.3). Derived analysis datasets are generated using SAS® and following standard CDISC Analysis Dataset Model conventions (implementation guide v1.0). Data analyses including summary tables, figures, and listings (TFLs) are produced using SAS®.

No central laboratory is used for this study. Local laboratory results are collected in the CRF in standard units along with clinical significance. Local laboratory reference ranges are collected outside of the CRF and sent to PRA directly.

AEs are coded using MedDRA version 19.0 to assign a system organ class (SOC) and preferred term (PT) to each AE. Concomitant medications are coded to preferred names using the World Health

Organization Drug Dictionary Enhanced (WHODRUG DDE, 2016Mar01). Anatomical Therapeutic Chemical (ATC) classification coding is included.

PRA's data handling and transfer procedures are documented separately in the study specific data management plan.

8.2 Data Screening

Beyond the data screening built into the PRA Data Management Plan, the PRA programming of analysis datasets and TFLs provides additional data screening. Presumed data issues are output into SAS® logs identified by the word “Problem” and extracted from the logs by a SAS® macro and sent to Data Management.

Review of post-freeze TFLs run on the frozen database allows for further data screening prior to database lock. The post-freeze TFLs are discussed with the sponsor and client in a data review meeting to identify any final data issues and seek corrections prior to database lock. Database lock must be approved by the approvers of the SAP.

9.0 Statistical Methods

All analyses use SAS® version 9.4 or higher. Summary tables are organized by treatment group reflecting the randomized treatment (GDNF or placebo) received in Study 2553 followed by the GDNF treatment received in Study 2797 (for definition see Section 5.0). Summary tables and listings for efficacy and imaging analyses will include baseline and Week 40 data from Study 2553. MMRM analyses will include all scheduled postbaseline data from Study 2553 in the model but data from interim visits in Study 2553 will not be summarized in tables or listed. Line graphs will display all data from baseline to Week 80/e40, including all data from Study 2553. Summary tables and listings for safety analyses that use Week 0 from Study 2553 as the baseline value will include both baseline and Week 40 data from Study 2553. With the exception of demographic data and PD history at screening in Study 2553, no other data from the parent study will be included in summary tables or listings. Important CRF data are included in data listings, sorted by treatment group, subject, and by visit within subject.

Data from the Pilot Extension and Supplemental Extension will be listed only, except for exposure data, concomitant medications, new or worsening TEAEs, QUIP, MoCA, MDRS, anti-GDNF serum antibody data, and GDNF plasma concentration data, which will be included in summary tables.

Unless otherwise noted, categorical data are presented using counts and percentages, with the number of subjects in the analysis population by treatment group as the denominator for percentages. Percentages are rounded to one decimal place. Continuous data, unless otherwise noted, are summarized using the number of observations (n), mean, standard deviation (SD), median, minimum, and maximum. Minima and maxima are rounded to the precision of the original value, and means, medians, and 95% confidence intervals (CIs) are rounded to 1 decimal place greater than the precision of the original value. SD is rounded to 2 decimal places greater than the precision of the original value, up to a maximum of 3 decimal places.

Any hypothesis testing is performed with a 2-sided alternative at the level of $\alpha = 0.05$. P-values are presented with 4 decimal places. No adjustments for multiplicity are made. All inferential analyses are for exploratory purposes only.

9.1 Missing Data Methods

9.1.1 Missing or Partial Dates

Missing or partial dates for AEs, concomitant medications, dosing records, Week 40/e0/early termination and Week 80/e40/early termination visits are imputed as described in Section 5.0 (see definitions for TEAE, concomitant medications, and visit windows).

9.1.2 Missing Efficacy Data

For efficacy endpoints, missing data are not imputed.

Details on handling of missing and duplicate PD motor fluctuation diary ratings are described in Section 9.7.2.5.

The handling of missing data for supplementary efficacy endpoints is described in the appropriate parts of Section 9.7.3.

9.1.3 Missing Imaging Data

For imaging endpoints, missing data are not imputed.

9.1.4 Missing Data for Questionnaires

There are 2 levels of missing data possible for questionnaires: either the entire instrument was not assessed at a scheduled time point, or one or more individual items on the instrument were left blank. In the former case of an entirely missed assessment, no imputation is performed.

In the latter case of one or more individual items missed, imputation is performed according to the scoring instructions of the instrument. If the scoring instructions do not address individual missing items, then the imputation method depends on the frequency of postbaseline scheduled assessments. For instruments that have multiple postbaseline scheduled time points, individual missing items are imputed using last observation carried forward (LOCF) (ie, the score for the missing item is taken from the last non-missing postbaseline time point including data from Study 2553 where applicable). For instruments that have only one postbaseline scheduled time point and at least 5 individual items in the subscale or scale being scored, individual missing responses are imputed using the average of non-missing scores. An exception to this rule occurs if more than half of the individual items are missing, in which case no imputation is performed and the subscale or scale score is left missing. Finally, for instruments that have only one postbaseline scheduled time point and fewer than 5 individual items (eg, SNAQ), the total score is considered missing if one or more individual response is missing.

Handling of individual missing items for each scale is discussed in the appropriate parts of Section 9.7 and Section 9.9.

9.1.5 Missing Safety Data

No imputation is performed for missing safety data other than questionnaire data.

9.1.6 Special Arrangements for Subject 45

Special arrangements have been made for subject 45 who had a conus injury during Study 2553 that was unrelated to study treatment or device. As a result of this, items 27, 28, 29 and 30 of the UPDRS score could not be completed beyond Week 8. Item 22, although recorded, is considered to be confounded. Therefore, for this subject, these 5 items are excluded from all calculations of the UPDRS motor score (part III) used in the efficacy analyses, and a truncated score including all other items of part III is used instead. UPDRS parts I, II and IV were collected as far as possible, but the data are not included in the related efficacy analyses because they are considered to be confounded due to the injury. Timed walk could not be done after Week 8 and is therefore excluded from the efficacy analyses. PDQ 39 (items 14 to 39), EQ-5D and NMSS were recorded but are considered to be confounded due to the injury and are therefore excluded from the efficacy analyses.

9.2 Subject Disposition

A tabulation of subject disposition for the Initial Extension is provided for the following categories (see Section 6.0 for population definitions):

- Numbers of Pilot Stage subjects who were enrolled in the extension study and were treated.

- Numbers of Primary Stage subjects who were enrolled in the extension study (ITT Primary Population) and were treated.
- Numbers of Pilot Stage + Primary Stage subjects who were enrolled in the extension study (ITT Overall Population) and were treated (Safety Overall Population).

Subject disposition is also tabulated in a similar manner for the Pilot Extension and Supplemental Extension as appropriate.

The number and percentage of subjects who completed the Week 80/e40 visit is summarized for the ITT Primary Population and ITT Overall Population by treatment group and overall, together with the number and percentage of subjects who withdrew from the study prematurely during the Initial Extension and a breakdown of the corresponding primary reasons for early termination. See Section 5.0 for definitions of completion of study and early termination.

Disposition data for all extension parts of Study 2797 are listed by subject, as are population inclusion data (showing which subjects are included in which analysis population). Data for informed consent and inclusion/exclusion criteria are not listed, but are included in SDTM datasets.

9.3 Protocol Deviations

Protocol deviations are presented by treatment group and overall for the ITT Overall Population by deviation category (major and minor deviations) and deviation name (only major deviations), displaying the number and percentage of subjects in each group to which each deviation category and deviation name apply. Protocol deviations are discussed in Section 5.0.

Major and minor protocol deviation data are listed by study stage, treatment group, and subject.

9.4 Demographic and Baseline Characteristics

9.4.1 Demographic Characteristics

The following demographic characteristics from Study 2553 are tabulated by treatment group and overall for the ITT Primary Population and the ITT Overall Population.

- Age (years)
- Age group (< 65, ≥ 65 years)
- Sex (female, male)
- Race (white, black, Asian, Native Hawaiian or other Pacific Islander, American Indian or Alaska Native, other)
- Ethnicity (Hispanic or Latino, not Hispanic or Latino)
- Baseline weight (kg)
- Baseline height (m)
- Baseline BMI (kg/m^2; see definition in Section 5.0)
- National Adult Reading Test (NART) error score (points)

Demographic data from Study 2553 are listed by study stage, treatment group, and subject.

9.4.2 Parkinson’s Disease History at Screening

The following PD history items from Study 2553 are tabulated by treatment group and overall for the ITT Primary Population and the ITT Overall Population.

- Duration since first PD symptom (years)
- Duration since PD diagnosis (years)
- Hoehn and Yahr stage in OFF state (0, 1, 1.5, 2, 2.5, 3)
- OFF state UPDRS motor score (part III; points)
- ON state UPDRS motor score (part III; points)
- Total daily levodopa dose (mg)
- Total daily levodopa equivalent dose (mg)
- PD medications as recorded on the Levodopa and Levodopa Equivalent Medications CRF (eg, levodopa preparations, dopamine agonists, COMT inhibitors, MAO-B inhibitors, other)
- Responsiveness to levodopa (ie, percentage change in screening UPDRS motor score [part III] following a levodopa challenge)
- OFF time per day (hours)

PD history data from Study 2553 are listed by study stage, treatment group, and subject.

9.5 Concomitant Medications

Medications received concomitantly with open-label study medication in any extension part of Study 2797 (Initial Extension, Pilot Extension, Supplemental Extension), categorized by ATC class and preferred name according to WHODRUG DDE, are summarized by treatment group for the ITT Primary Population. Separate summaries are presented for concomitant PD medications and other concomitant medications. The number and percentage of subjects using any concomitant medication is displayed together with the number and percentage of subjects using at least one medication within each ATC class and preferred name. See definition of concomitant medications in Section 5.0.

Concomitant medication data from all extension parts of Study 2797 (Initial Extension, Pilot Extension, Supplemental Extension) are listed by study stage, treatment group and subject. Levodopa challenge dosing data are not listed, but are included in SDTM datasets.

9.6 Surgery and Test Infusions

9.6.1 Catheter Trajectories and Positioning Accuracy

Catheter trajectory data and catheter positioning accuracy data from repositioning surgery are listed by study stage, treatment group, and subject. All other surgery data, including data from post-operative CT scans, are not listed, but are included in SDTM datasets.

9.6.2 Contrast-Enhanced Test Infusions with T1-Weighted MRI Prior to Start of Study Medication

Data for all test infusions, including repeat or unscheduled test infusions, are listed by study stage, treatment group, and subject.

9.7 Efficacy Analyses

Unless otherwise specified, efficacy analyses described in this SAP assess change from the baseline value of Study 2553 to Week 80 (Week e40 in the Initial Extension of Study 2797).

The primary efficacy analysis uses the ITT Primary Population. A sensitivity analysis of the primary endpoint is performed using the ITT Overall Population. Analyses of secondary endpoints are performed

using the ITT Primary Population and/or ITT Overall Population in a manner similar to the analyses of the primary endpoint. Analyses of supplementary endpoints are performed for the ITT Overall Population.

All efficacy endpoints are tested at the α = 0.05 level, 2-sided without multiplicity adjustment.

End of study/early termination visit data from the Initial Extension of Study 2797 are windowed to the appropriate scheduled visit and are not included in Week 80/e40 scheduled visit data (see Section 5.0 definition of visit windows).

UPDRS motor (part III) and ADL (part II) scores are generally assessed by raters who are blinded to the subject's treatment assignment in Study 2553.

For efficacy endpoints based on the UPDRS, missing data are not imputed.

All UPDRS data from all extension parts of Study 2797 (Initial Extension, Pilot Extension, Supplemental Extension) are listed by study stage, treatment group, and subject for all UPDRS parameters, including OFF and ON state motor score (part III), OFF and ON state ADL score (part II), OFF and ON state UPDRS total score (sum of motor + ADL scores), mentation, behavior, and mood score (part I), and complications of therapy score (part IV). Baseline and Week 40 values from Study 2553 and changes from baseline in each score are also listed.

9.7.1 Analyses of Primary Efficacy Endpoint

9.7.1.1 Primary Analysis: MMRM of Primary Efficacy Endpoint

The percentage change from baseline to Week 80/e40 in the practically defined OFF state UPDRS motor score (part III) is compared between treatment groups for the ITT Primary Population using a mixed-effect model with repeated measures (MMRM). Baseline UPDRS score is a covariate, treatment group and visit and treatment group*visit are fixed effects, and subject within treatment group is a random effect. The covariance matrix is unstructured. The following SAS® code fragment approximates the analysis:

proc mixed data=<input>;

class <usubjid> <trt01p> <visit>;

model <pchg> = <base> <trt01p> <visit> <trt01p>*<visit>/ ddfm=KR [A];

repeated / type=un subject=<usubjid>(<trt01p>);

lsmeans <trt01p> / pdiff;

estimate 'Trt diff at Wk80' <trt01p> 1 -1 <trt01p>*<visit> 0 0 0 0 0 0 0 0 0 1 0 0 0 0 0 0 0 0 0 -1 / cl;

run;

[A] The Kenward and Roger method for calculating the denominator degrees of freedom for tests of fixed effects will be used.

Summary statistics are tabulated by treatment group expressed as least squares means with 95% CIs, difference between least squares means (GDNF/GDNF – placebo/GDNF) with 95% CIs, and corresponding p-value. Subjects who are missing their Week 80/e40 UPDRS motor score (part III) value are counted in the model at all postbaseline time points for which data are present. Summary statistics are displayed by visit for baseline, Week 40/e0, and Week 80/e40, but inferential statistics are presented only for Week 80/Week e40. Scheduled visits included in the model are Week 0 (baseline; see Section 5.0 definition of baseline) and Weeks 8, 16, 24, 32, 40/e0, 48/e8, 56/e16, 64/e24, 72/e32 and 80/e40.

Percentage change from baseline in OFF state UPDRS motor score (part III) over time up to Week 80/e40 is plotted on a line plot displaying mean values and standard error (SE) bars.

An exploratory model was developed to describe the disease progression in PD as a function of time using Parkinson's Progression Markers Initiative (PPMI) data. Baseline severity was a significant covariate in the model showing a decrease in the rate of disease progression as baseline severity increases. The population estimate of disease progression was similar to previously reported values. Using a baseline UPDRS motor score (part III) of 35, disease progression is predicted to occur at a modest annual rate of 1.56 points (4.5%) per year, translating to an increase of the baseline score by 1.17 points (3.3%) at 9 months and 2.34 points (6.7%) at 18 months. This modelled control will also be plotted on the percentage change from baseline figure for a visual comparision versus both treatment groups.

Individual subject data for OFF state UPDRS motor score (part III) over time up to Week 80/e40 are also plotted on a line plot by treatment group (displayed in separate graphs).

The GDNF/GDNF treatment group is judged superior compared with placebo/GDNF if there is sufficient statistical evidence to reject the following null hypothesis in the direction favorable to GDNF/GDNF:

> *H_0: No significant difference in the percentage change from baseline to Week 80/e40 in the OFF state UPDRS motor score (part III) between GDNF/GDNF and placebo/GDNF*
>
> and accept the alternative hypothesis:
>
> *H_a: A significantly greater percentage decrease (lower UPDRS is better) in the change from baseline to Week 80/e40 in the OFF state UPDRS motor score (part III) for GDNF/GDNF relative to placebo/GDNF*

It is also possible that a significantly greater percentage decrease for placebo/GDNF as compared with GDNF/GDNF is found, in which case placebo/GDNF is judged superior to GDNF/GDNF (ie, the test is 2-sided).

9.7.1.2 Sensitivity Analysis of Primary Efficacy Endpoint

The primary efficacy analysis is repeated for the ITT Overall Population.

9.7.2 Analyses of Secondary Efficacy Endpoints

The analysis populations for the analyses of secondary efficacy endpoints are specified in the following sections.

9.7.2.1 Change From Baseline in OFF State UPDRS Motor Score (Part III)

The change from baseline to Week 80/e40 in OFF state UPDRS motor score (part III) is compared between treatment groups for both the ITT Primary Population and the ITT Overall Population and reported in a manner identical to that for the primary efficacy endpoint using an MMRM. Summary statistics are tabulated by treatment group expressed as least squares means with 95% CIs, difference between least squares means (GDNF/GDNF – placebo/GDNF) with 95% CIs, and corresponding p-value.

Change from baseline in OFF state UPDRS motor score (part III) over time up to Week 80/e40 is plotted on a line plot displaying mean values and SE bars.

9.7.2.2 Change and Percentage Change from Baseline to Week 80/e40 in Other UPDRS Scores

Change and percentage change from baseline to Week 80/e40 are also analyzed for the following other UPDRS scores:

- UPDRS motor score (part III) in the ON state (following a levodopa challenge) (ITT Overall Population).
- UPDRS activities of daily living (ADL) score (part II) in the OFF state (ITT Primary Population and ITT Overall Population) and in the ON state (ITT Overall Population).

- UPDRS total score (sum of motor + ADL scores) in the OFF state (ITT Primary Population and ITT Overall Population) and in the ON state (ITT Overall Population).

The change and percentage change from baseline to Week 80/e40 in each UPDRS score are compared between treatment groups for the respective analysis populations and reported in a manner identical to that for the primary efficacy endpoint using an MMRM. Summary statistics are tabulated by treatment group expressed as least squares means with 95% CIs, difference between least squares means (GDNF/GDNF – placebo/GDNF) with 95% CIs, and corresponding p-value.

Change from baseline in each score over time up to Week 80/e40 is plotted on line plots displaying mean values and SE bars.

9.7.2.3 Change in UPDRS Scores from Baseline to Week 40/e0 for the GDNF/GDNF Group Compared to Change from Baseline to Week 80/e40 for the Placebo/GNDF Group

Change from baseline to Week 40/e0 for the GDNF/GDNFgroup is compared to change from baseline to Week 80/e40 for the placebo/GDNF group for the following UPDRS scores:

- OFF state UPDRS motor score (part III).
- OFF state UPDRS ADL score (part II).
- OFF state UPDRS total score (sum of motor + ADL scores).

The change in score from baseline to Week 40/e0 for the GDNF/GDNF group is compared to the change in the score from baseline to Week 80/e40 for the placebo/GDNF group for the ITT Overall Population and reported in a manner identical to that for the primary efficacy endpoint using an MMRM. Summary statistics are tabulated by treatment group expressed as least squares means with 95% CIs, difference between least squares means (GDNF/GDNF – placebo/GDNF) with 95% CIs, and corresponding p-value.

9.7.2.4 Change in UPDRS Scores from Baseline to Week 80/e40 for the GDNF/GDNF Group Compared to Change from Baseline to Week 40/e0 for the Placebo/GNDF Group

Change from baseline to Week 80/e40 for the GDNF/GDNFgroup is compared to change from baseline to Week 40/e0 for the placebo/GDNF group for the following UPDRS scores:

- OFF state UPDRS motor score (part III).
- OFF state UPDRS ADL score (part II).
- OFF state UPDRS total score (sum of motor + ADL scores).

The change in score from baseline to Week 80/e40 for the GDNF/GDNF group is compared to the change in the score from baseline to Week 40/e0 for the placebo/GDNF group for the ITT Overall Population and reported in a manner identical to that for the primary efficacy endpoint using an MMRM. Summary statistics are tabulated by treatment group expressed as least squares means with 95% CIs, difference between least squares means (GDNF/GDNF – placebo/GDNF) with 95% CIs, and corresponding p-value.

9.7.2.5 Change From Baseline in PD Diary Ratings

PD motor fluctuation diary rating data are collected on scheduled visit days at baseline and every 8 weeks in Study 2553 and in the Initial Extension of Study 2797, and at longer intervals in the Pilot Extension and Supplemental Extension of Study 2797. For diary purposes, a day is defined as a 24-hour period broken into half-hour intervals starting with the 06:00 am interval and ending with the 05:30 am interval the following day. Missing PD motor fluctuation diary ratings are not imputed.

A total of 3 diaries per scheduled visit day are completed by subjects. Each diary collects the state that represents the predominant status during each half-hour interval of the 24-hour period (asleep, OFF, ON without dyskinesias, ON with non-troublesome dyskinesias, ON with troublesome dyskinesias). Errors in

diary data and multiple responses in the same half-hour interval are defined as missing data. Data for half-hour intervals with errors are not used for analysis, but the remaining data recorded on the diary are valid only if a maximum of 4 errors are present. If 5 or more errors are present in a given diary, then the entire diary is considered invalid and not used for analysis.

Among the valid diaries (up to 3) per subject and scheduled visit, the mean times of any given state over all valid diaries are used for analysis. From these data, total OFF time per day, total good-quality ON time per day, and ON time per day with troublesome dyskinesias are estimated for the subject and scheduled visit, where each half-hour interval checked contributes 30 minutes to the sum (see definitions in Section 5.0).

The change from baseline to Week 80/e40 in PD motor fluctuation diary ratings is compared between treatment groups for both the ITT Primary Population and the ITT Overall Population and reported in a manner identical to that for the primary efficacy endpoint using an MMRM. Parameters include:

- Total OFF time per day
- Total good-quality ON time per day (sum of ON time per day without dyskinesias + ON time per day with non-troublesome dyskinesias)
    - ON time per day without dyskinesias
    - ON time per day with non-troublesome dyskinesias
- ON time per day with troublesome dyskinesias

Summary statistics are displayed by visit for baseline, Week 40/e0, and Week 80/e40, but inferential statistics are presented only for Week 80/Week e40. Scheduled visits included in the model are Week 0 (baseline; see Section 5.0 definition of baseline) and Weeks 8, 16, 24, 32, 40/e0, 48/e8, 56/e16, 64/e24, 72/e32 and 80/e40.

Change from baseline in total OFF time per day and change from baseline in total good-quality ON time per day up to Week 80/e40 are plotted on line plots displaying mean values and SE bars.

PD diary ratings from all extension parts of Study 2797 (Initial Extension, Pilot Extension, Supplemental Extension) are listed by study stage, treatment group, and subject. Diary training, diary dispensation, and diary collection and review are not listed, but are included in SDTM datasets.

9.7.2.6 Treatment Response

Treatment response at Week 80/e40 is compared between treatment groups for the ITT Overall Population based on the following criteria:

- Decrease from baseline to Week 80/e40 by ≥10 points in OFF state UPDRS motor score (part III).
- Increase from baseline to Week 80/e40 by ≥1 hour in total good-quality ON time per day (ON without dyskinesias or ON with non-troublesome dyskinesias).
- Both of the above criteria, i.e. decrease from baseline to Week 80/e40 by ≥10 points in OFF state UPDRS motor score (part III) and increase from baseline to Week 80/e40 by ≥1 hour in total good-quality ON time per day (ON without dyskinesias or ON with non-troublesome dyskinesias).

Treatment response at Week 40/e0 is defined using the same criteria.

Summary statistics are displayed by visit for Week 40/e0 and Week 80/e40, but inferential statistics are presented only for Week 80/e40.

UPDRS responder (≥10 points in OFF state UPDRS motor score (part III)) data are also summarized using a shift table by treatment group. Using the number of responders/non-responders at each timepoint, this table compares Week 80/e40 to Week 40/e0.

Absolute change from baseline to Week 40/e0 and Week 80/e40 in OFF state UPDRS motor score (part III) is plotted as frequency distribution plots displaying the number of subjects for any given change by treatment group.

Treatment response in the Initial Extension is listed by study stage, treatment group, and subject.

9.7.3 Analyses of Supplementary Efficacy Endpoints

All analyses of supplementary endpoints are done for the ITT Overall Population.

9.7.3.1 MMRM of Change From Baseline in Timed Walking Test

During the timed walking test, the subject walks as fast as possible 7 meters back and forth including turning. The time to perform this test is recorded for 2 trials in the OFF state and 2 trials in the ON state after levodopa challenge.

The change from baseline to Week 80/e40 in timed walking test is compared between treatment groups for the ITT Primary Population and reported in a manner identical to that for the primary efficacy endpoint using an MMRM. Results are summarized by treatment group expressed as least squares means with 95% CIs, difference between least squares means (GDNF/GDNF – placebo/GDNF) with 95% CIs, and corresponding p-value. Timed walking test parameters are OFF state timed walking test and ON state timed walking test in seconds.

Summary statistics are displayed by visit for baseline, Week 40/e0, and Week 80/e40, but inferential statistics are presented only for Week 80/e40. Scheduled visits included in the model are Week 0 (baseline; see Section 5.0 definition of baseline) and Weeks 8, 16, 24, 32, 40/e0, 48/e8, 56/e16, 64/e24, 72/e32 and 80/e40. The results of the two separate trials per state at each visit are averaged and the mean used for analysis of each state. If only the first trial is completed for a particular state, then the second trial result will be imputed with the worst non-missing second trial result from all subjects for that particular state during the study. If both trials are missing, then the endpoint is not reported for that visit.

Timed walking test data from all extension parts of Study 2797 (Initial Extension, Pilot Extension, Supplemental Extension) are listed by study stage, treatment group, and subject. Changes from baseline in the results are listed.

9.7.3.2 MMRM of Change From Baseline in Timed Tapping Test

During the timed tapping test, the subject alternates tapping the index finger for 20 seconds between 2 points spaced 30 cm apart. The test is performed twice for each hand in the OFF state and twice for each hand in the ON state after levodopa challenge.

The change from baseline to Week 80/e40 in timed tapping test is compared between treatment groups for the ITT Primary Population and reported in a manner identical to that for the primary efficacy endpoint using an MMRM. Results are summarized by treatment group expressed as least squares means with 95% CIs, difference between least squares means (GDNF/GDNF – placebo/GDNF) with 95% CIs, and corresponding p-value. Timed tapping test parameters are OFF state timed tapping test and ON state timed tapping test in number of taps completed in 20 seconds.

Summary statistics are displayed by visit for baseline, Week 40/e0, and Week 80/e40, but inferential statistics are presented only for Week 80/e40. Scheduled visits included in the model are Week 0 (baseline; see Section 5.0 definition of baseline) and Weeks 8, 16, 24, 32, 40/e0, 48/e8, 56/e16, 64/e24, 72/e32 and 80/e40. The results of the four separate trials per state at each visit are averaged and the mean used for analysis for each state. If any trial is not completed for a particular state, then the trial result will be imputed with the worst non-missing applicable trial result (for example, first left hand trial)

from all subjects for that particular state during the study. If all four trials are missing, then the endpoint is not reported for that visit.

Timed tapping test data from all extension parts of Study 2797 (Initial Extension, Pilot Extension, Supplemental Extension) are listed by study stage, treatment group, and subject. Changes from baseline in the results are listed.

9.7.3.3 MMRM of Change from Baseline in NMSS Score

The NMSS is an interview-based scale used to rate non-motor symptoms commonly occurring in PD (developed by the International Parkinson's Disease Non-Motor Group). It is administered with the subject in the ON state. The 30-item scale rates symptoms that occurred in the preceding month in 9 domains (cardiovascular including falls; sleep/fatigue; mood/cognition; perceptual problems/hallucinations; attention/memory; gastrointestinal tract; urinary; sexual function; miscellaneous). Each item is rated from 0 (none) to 3 (severe) for severity and from 1 (rarely) to 4 (very frequent) for frequency. The score for each item is the product of the severity rating multiplied by the frequency. The maximum score for an individual item is 12. The higher the score, the worse the subject's condition. Individual item scores in each domain are summed to give the domain score, and the domains are summed to give the total score. The maximum NMSS total score is 360. Missing individual item scores are imputed using LOCF.

The change from baseline to Week 80/e40 in NMSS data is compared between treatment groups for the ITT Primary Population and reported in a manner identical to that for the primary efficacy endpoint using an MMRM. Results are summarized by treatment group expressed as least squares means with 95% CIs, difference between least squares means (GDNF/GDNF – placebo/GDNF) with 95% CIs, and corresponding p-value. NMSS parameters are the 9 NMSS domains and the NMSS total score.

Summary statistics are displayed by visit for baseline, Week 40/e0, and Week 80/e40, but inferential statistics are presented only for Week 80/e40. Scheduled visits included in the model are Screening Visit 2 from Study 2553 (baseline; see Section 5.0 definition of baseline) and Weeks 12, 24, 40/e0, 52/e12, 64/e24 and 80/e40.

NMSS data from the Initial Extension and Pilot Extension of Study 2797 are listed by study stage, treatment group, and subject.

9.7.3.4 ANCOVA of Change From Baseline in PDQ-39 Score

The PDQ-39 is a self-administered 39-item PD-specific scale that rates symptoms that occurred in the preceding month in the 8 dimensions listed below. Each item is rated from 0 (never) to 4 (always) for frequency. Each dimension is calculated as a scale from 0 to 100 based on the following formulas:

- Mobility = [(Sum of Scores of questions 1 – 10)/ (4*10)] *100
- Activities of Daily Living = [(Sum of Scores of questions 11 – 16)/(4*6)]*100
- Emotional Well Being = [(Sum of Scores of questions 17 – 22)/(4*6)]*100
- Stigma = [(Sum of Scores of questions 23 – 26)/(4*4)]*100
- Social Support = [(Sum of Scores of questions 27 – 29)/(4*3)]*100
    - o If respondents indicate that they do not have a spouse/partner on question 28, then Social Support = [(Sum of Scores of questions 27 & 29)/(4*2)]*100
- Cognitions = [(Sum of Scores of questions 30 – 33)/(4*4)]*100
- Communication = [(Sum of Scores of questions 34 – 36)/(4*3)]*100
- Bodily Discomfort = [(Sum of Scores of questions 37 – 39)/(4*3)]*100

The total score, or single index, is the average of all 8 dimension scores. The higher the score, the worse the subject's condition. If the response to an individual question is missing, then no score is calculated for that dimension and therefore the single index score cannot be calculated.

Analysis of PDQ-39 data on the ITT Primary Population utilizes an ANCOVA model adjusted for baseline PDQ-39 score. Input data are restricted to observed data at baseline and Week 80/e40 only. PDQ-39 parameters are the 8 PDQ-39 dimensions and the single index (total) PDQ-39 score.

Summary statistics are displayed for Screening Visit 2 (baseline; see Section 5.0 definition of baseline), Week 40/e0, and Week 80/e40. Estimated mean difference (GDNF/GDNF – placebo/GDNF), 95% CIs, and p-values are reported.

PDQ-39 data are listed by study stage, treatment group, and subject.

9.7.3.5 ANCOVA of Change From Baseline in EQ-5D Score

The EQ-5D is a subject self-report measure of quality of life consisting of a questionnaire and a visual analog scale. The questionnaire comprises 5 questions on mobility, self-care, usual activities, pain/discomfort, and anxiety/depression, with three possible answers for each item (1=no problem, 2=moderate problem, 3=severe problem). The visual analog scale serves as an indicator of general health status; the scale ranges from 0 to 100, where 0 indicates worst health and 100 indicates best health. Missing values for individual questions are coded as 9; missing values for the visual analog scale are coded as 999. Missing values are not included in observed data analyses.

EQ-5D questionnaire data are reported using frequency counts and percentages for Screening Visit 2 (baseline; see Section 5.0 definition of baseline), Week 40/e0, and Week 80/e40. Input data are restricted to observed data at baseline, Week 40/e0, and Week 80/e40 only. Parameters are the frequency counts and percentages of subjects with the different answers for each of the 5 questions.

Analysis of EQ-5D visual analog scale data on the ITT Primary Population utilizes an ANCOVA model adjusted for baseline EQ-5D visual analog scale score. Input data are restricted to observed data at baseline and Week 80/e40 only. The parameter is the visual analog scale score. Summary statistics are displayed for Screening Visit 2 from Study 2553 (baseline; see Section 5.0 definition of baseline), Week 40/e0, and Week 80/e40. Estimated mean difference (GDNF/GDNF – placebo/GDNF), 95% CIs, and p-values are reported.

EQ-5D data are listed by study stage, treatment group, and subject.

9.7.3.6 ANCOVA of Change From Baseline in SNAQ Score

The SNAQ is a self-administered 4-question instrument with total scores ranging from 4 to 20 (4=poor appetite, 20=good appetite). The SNAQ has one total score. If an individual question is not answered, then the total score is considered missing.

Analysis of SNAQ data on the ITT Primary Population utilizes an ANCOVA model adjusted for baseline SNAQ score. Input data are restricted to observed data at baseline and Week 80/e40 only.

Summary statistics are displayed for Screening Visit 2 from Study 2553 (baseline; see Section 5.0 definition of baseline), Week 40/e0, and Week 80/e40. Estimated mean difference (GDNF/GDNF – placebo/GDNF), 95% CIs, and p-values are reported.

SNAQ data are listed by study stage, treatment group, and subject.

9.7.3.7 ANCOVA of Change From Baseline in Total Daily Levodopa Dose and Total Daily Levodopa Equivalent Dose

Analysis of change from baseline to Week 80/e40 data on the ITT Primary Population utilizes an ANCOVA model adjusted for baseline total daily levodopa dose and total daily levodopa equivalent dose,

respectively (see definitions in Section 5.0). Input data are restricted to observed data at baseline and Week 80/e40 only (no imputation of missing data).

Summary statistics are displayed for Week 0 (baseline; see Section 5.0 definition of baseline), Week 40/e0, and Week 80/e40. Estimated mean difference (GDNF/GDNF – placebo/GDNF), 95% CIs, and p-values are reported.

Levodopa dose data and levodopa equivalent dose data from the Initial Extension are listed by study stage, treatment group, and subject. Separate listings are provided for actual total daily dose of each PD medication and for the total daily dose of levodopa and the total daily dose of levodopa equivalent.

9.8 Imaging Analyses

The analysis populations for imaging analyses are specified in the following sections.

9.8.1 MRI Analyses

All MRI analyses are restricted to the ITT Primary Population since in Pilot Stage subjects, only T2-weighted MRI scans were taken at baseline.

Post-infusion MRI data needed for the imaging analyses described in this section are listed for Primary Stage subjects; although not analyzed or tabulated, corresponding post-infusion MRI data are also listed for Pilot Stage subjects. All other MRI data, including real-time MRI data, are not listed, but are included in SDTM datasets.

9.8.1.1 ANCOVA of Change From Baseline in Volume of Distribution of Infusate as Determined by Contrast-Enhanced T1-Weighted MRI

Analysis of change from baseline to Week 80/e40 in volume of distribution of infusate between treatment groups utilizes an ANCOVA model adjusted for baseline volume of distribution. Input data are restricted to observed data at the end of the healing phase in Study 2553, Week 40/e0, and at Week 80/e40 only. The parameter is the volume of distribution (in mL), separately for left and right hemispheres, as determined by contrast-enhanced T1-weighted MRI.

Summary statistics are displayed for the last test infusion at the end of the healing phase in Study 2553 (baseline; see Section 5.0 definition of baseline), Week 40/e0, and Week 80/e40. Estimated mean difference (GDNF/GDNF – placebo/GDNF), 95% CIs, and p-values are reported.

Volume of distribution data from the Initial Extension and Pilot Extension of Study 2797 are listed by study stage, treatment group, and subject.

9.8.1.2 ANCOVA of Change from Baseline in Volume of Interest Coverage and Total Putamenal Coverage as Determined by Contrast-Enhanced T1-Weighted MRI

Analysis of change from baseline to Week 80/e40 in VOI coverage and total putamenal coverage utilizes an ANCOVA model adjusted for baseline coverage. Input data are restricted to observed data at the end of the healing phase in Study 2553, Week 40/e0, and at Week 80/e40 only. Parameters include VOI coverage as a percentage of total VOI and total putamenal coverage as a percentage of total putamenal volume, separately for left and right putamen and for both putamina combined.

Summary statistics are displayed for the last test infusion at the end of the healing phase in Study 2553 (baseline; see Section 5.0 definition of baseline), Week 40/e0, and Week 80/e40. Estimated mean difference (GDNF/GDNF – placebo/GDNF), 95% CIs, and p-values are reported for each analysis.

VOI coverage (absolute and relative) and total putamenal coverage data from the Initial Extension and Pilot Extension of Study 2797, including underlying data (VOI, putamenal volume of distribution, and total volume of putamen), are listed by study stage, treatment group, and subject.

9.8.2 Correlation Analyses

9.8.2.1 Correlation Between Primary Study Endpoint and Volume of Interest Coverage and Total Putamenal Coverage at Baseline as Determined by Contrast-Enhanced T1-Weighted MRI

These analyses are restricted to the ITT Primary Population since in Pilot Stage subjects, only T2-weighted MRI scans were taken at baseline.

These analyses use non-parametric Spearman rank correlation to explore the relationship between percentage change from baseline to Week 80/e40 in OFF state UPDRS motor score (part III) and both VOI coverage and total putamenal coverage after the last test infusion at the end of the healing phase in Study 2553 by treatment group. Correlations are calculated separately for each treatment group. Parameters include percentage change from baseline to Week 80/e40 in OFF state UPDRS motor score (part III) and both VOI coverage as a percentage of total VOI and total putamenal coverage as a percentage of total putamenal volume, for both putamina combined.

The estimated correlation coefficient, 95% CI, and p-value are tabulated for each analysis and treatment group, and scatterplots are provided.

9.8.2.2 Correlation Between Primary Study Endpoint and Change From Baseline to Week 40/e0 in ^{18}F-DOPA Uptake as Determined by PET Scan

This analysis uses non-parametric Spearman rank correlation to explore the relationship between percentage change from baseline to Week 80/e40 in OFF state UPDRS motor score (part III) and change from baseline to Week 40/e0 in PET imaging of ^{18}F-DOPA uptake by treatment group for both the ITT Primary Population and the ITT Overall Population. Correlations are calculated separately for each treatment group. Parameters include percentage change from baseline to Week 80/e40 in OFF state UPDRS motor score (part III) and change from baseline to Week 40/e0 in ^{18}F-DOPA uptake rate constant determined by PET for each of 5 regions of interest (dorsal caudate nucleus, dorsal anterior putamen, dorsal central/posterior putamen, ventral striatum, and substantia nigra), using the average from both hemispheres for each region.

The estimated correlation coefficient, 95% CI, and p-value are tabulated by treatment group, and scatterplots are provided.

9.8.2.3 Correlation Between Baseline OFF State UPDRS Motor Score (Part III) and Baseline ^{18}F-DOPA Uptake as Determined by PET Scan

This analysis uses non-parametric Spearman rank correlation to explore the relationship between baseline (Week 0) OFF state UPDRS motor score (part III) and baseline (Week 0) PET imaging of ^{18}F-DOPA uptake by treatment group for the ITT Overall Population. Parameters include baseline OFF state UPDRS motor score (part III) and baseline ^{18}F-DOPA uptake rate constant determined by PET for dorsal central/posterior putamen and for dorsal anterior putamen, using the averages from both hemispheres. Correlations are calculated separately for each treatment group and putamen region combination.

The estimated correlation coefficients and p-values are provided on scatterplots, one for each of the 4 correlations analyzed.

9.8.2.4 Correlation Between Baseline OFF State UPDRS ADL Score (Part II) and Baseline ^{18}F-DOPA Uptake as Determined by PET Scan

This analysis uses non-parametric Spearman rank correlation to explore the relationship between baseline (Week 0) OFF state UPDRS ADL score (part II) and baseline (Week 0) PET imaging of ^{18}F-DOPA uptake by treatment group for ITT Overall Population. Parameters include baseline OFF state

UPDRS ADL score (part II) and baseline ^{18}F-DOPA uptake rate constant determined by PET for dorsal central/posterior putamen and for dorsal anterior putamen, using the averages from both hemispheres. Correlations are calculated separately for each treatment group and putamen region combination.

The estimated correlation coefficients and p-values are provided on scatterplots, one for each of the 4 correlations analyzed.

9.8.2.5 Correlation Between Week 40/e0 OFF State UPDRS Motor Score (Part III) and Week 40/e0 ^{18}F-DOPA Uptake as Determined by PET Scan

This analysis uses non-parametric Spearman rank correlation to explore the relationship between Week 40/e0 OFF state UPDRS motor score (part III) and Week 40/e0 PET imaging of ^{18}F-DOPA uptake by treatment group for the ITT Overall Population. Parameters include Week 40/e0 OFF state UPDRS motor score (part III) and Week 40/e0 ^{18}F-DOPA uptake rate constant determined by PET for dorsal central/posterior putamen and for dorsal anterior putamen, using the averages from both hemispheres. Correlations are calculated separately for each treatment group and putamen region combination.

The estimated correlation coefficients and p-values are provided on scatterplots, one for each of the 4 correlations analyzed.

9.8.2.6 Correlation Between Week 40/e0 OFF State UPDRS ADL Score (Part II) and Week 40/e0 ^{18}F-DOPA Uptake as Determined by PET Scan

This analysis uses non-parametric Spearman rank correlation to explore the relationship between Week 40/e0 OFF state UPDRS ADL score (part II) and Week 40/e0 PET imaging of ^{18}F-DOPA uptake by treatment group for the ITT Overall Population. Parameters include Week 40/e0 OFF state UPDRS ADL score (part II) and Week 40/e0 ^{18}F-DOPA uptake rate constant determined by PET for dorsal central/posterior putamen and for dorsal anterior putamen, using the averages from both hemispheres. Correlations are calculated separately for each treatment group and putamen region combination.

The estimated correlation coefficients and p-values are provided on scatterplots, one for each of the 4 correlations analyzed.

9.8.2.7 Correlation Between Week 80/e40 OFF State UPDRS Motor Score (Part III) and Week 40/e0 ^{18}F-DOPA Uptake as Determined by PET Scan

This analysis uses non-parametric Spearman rank correlation to explore the relationship between Week 80/e40 OFF state UPDRS motor score (part III) and Week 40/e0 PET imaging of ^{18}F-DOPA uptake by treatment group for the ITT Overall Population. Parameters include Week 80/e40 OFF state UPDRS motor score (part III) and Week 40/e0 ^{18}F-DOPA uptake rate constant determined by PET for dorsal central/posterior putamen and for dorsal anterior putamen, using the averages from both hemispheres. Correlations are calculated separately for each treatment group and putamen region combination.

The estimated correlation coefficients and p-values are provided on scatterplots, one for each of the 4 correlations analyzed.

9.8.2.8 Correlation Between Week 80/e40 OFF State UPDRS ADL Score (Part II) and Week 40/e0 ^{18}F-DOPA Uptake as Determined by PET Scan

This analysis uses non-parametric Spearman rank correlation to explore the relationship between Week 80/e40 OFF state UPDRS ADL score (part II) and Week 40/e0 PET imaging of ^{18}F-DOPA uptake by treatment group for the ITT Overall Population. Parameters include Week 80/e40 OFF state UPDRS ADL score (part II) and Week 40/e0 ^{18}F-DOPA uptake rate constant determined by PET for dorsal central/posterior putamen and for dorsal anterior putamen, using the averages from both hemispheres. Correlations are calculated separately for each treatment group and putamen region combination.

The estimated correlation coefficients and p-values are provided on scatterplots, one for each of the 4 correlations analyzed.

9.9 Safety Analyses

Unless otherwise specified, safety analyses described in this SAP assess the safety findings obtained in the extension Study 2797 for the Safety Overall Population. Data collected at Week 40 in Study 2553 is used as the baseline, where a baseline value was not obtained at Week e0 of the extension study.

No imputation is performed for missing safety data other than questionnaire data, as described below.

9.9.1 Study Medication Exposure

Study medication exposure data include number of infusions received and total GDNF exposure in mg, assuming the entire dose was infused at each administration (see definition of total exposure in Section 5.0). These data are presented by treatment group, separately for each extension part of Study 2797 (Initial Extension, Pilot Extension, Supplemental Extension) and overall in Study 2797.

Infusion details per study medication visit include duration of infusion (minutes) and any infusion interruptions/early terminations (yes/no; see definition of duration of infusion in Section 5.0). These data are summarized by treatment group by visit, including the Initial Extension, Pilot Extension and Supplemental Extension infusion visits.

Study medication exposure data from all parts of Study 2797 (Initial Extension, Pilot Extension, Supplemental Extension), along with infusion details, are listed by study stage, treatment group, and subject.

9.9.2 Adverse Events

AEs are collected throughout the study until 28 days after the last dose of study medication. Due to the temporal proximity of the start of Study 2797 to the end of Study 2553, all AEs reported during Study 2797 are considered TEAEs, regardless of whether their onset was before, on, or after the first open-label study medication dose date. TEAEs that are present or ongoing at the beginning of Study 2797 (date of consent or date of last visit in Study 2553, if consent was obtained earlier) are considered pre-existing, if the event term, severity, and date and time of onset in Study 2797 are identical to the corresponding information given for the TEAE in Study 2553. If any of these conditions is not met, the TEAE is considered new or worsening. Only new or worsening TEAEs are tabulated. Pre-existing TEAEs are listed. Unless otherwise specified, in the text below and in the table shells for summary tables, the term “TEAE” denotes “new or worsening TEAE”.

9.9.2.1 All Adverse Events

A summary of all TEAEs reported from all extension parts of Study 2797 (Initial Extension, Pilot Extension, Supplemental Extension) is presented by treatment group and overall. The summary includes the following categories:

- Overall summary of TEAEs
- TEAEs by MedDRA SOC and PT
- TEAEs experienced by at least 3 subjects in any treatment group by PT (number of subjects and number of events)
- TEAEs by MedDRA SOC, PT, and maximum severity
- Serious TEAEs by MedDRA SOC and PT
- Study medication-related TEAEs by MedDRA SOC and PT

- Serious study medication-related TEAEs by MedDRA SOC and PT
- Device-related TEAEs by MedDRA SOC and PT
- Serious device-related TEAEs by MedDRA SOC and PT

Except where specified, counting is by subject, not event, and subjects are only counted once within each SOC or PT. Sorting is alphabetically by SOC and then alphabetically for PT.

All TEAEs are listed by study stage, treatment group, and subject; pre-existing TEAEs are flagged.

9.9.2.2 Adverse Events of Special Interest

Four categories of treatment-emergent AESIs will be analyzed: dyskinesias; falls; adverse changes in mood; and impulsivity (see definitions in Section 5.0). AESIs are presented by category and PT by treatment group and overall.

AESIs are listed by category, study stage, treatment group, and subject.

9.9.3 Port Symptoms

Evaluation of port symptoms includes the following categories:

- No skin reaction
- Redness with slight swelling
- Redness, moistness and moderate swelling with tissue granulation
- Overt infection

Port symptoms are listed by study stage, treatment group, and subject.

9.9.4 Laboratory Data

Clinical laboratory evaluations (hematology, serum chemistry, and urinalysis) are performed at Weeks 44/e4, 56/e16, 68/e8 and 80/e40, e2-80, and the last study visit in the Supplemental Extension. Laboratory parameters tested are listed in Table 5.

Table 5. Laboratory Parameters

Hematology	Serum Chemistry	Urinalysis	Other
RBC count	Alkaline phosphatase	Color	Pregnancy test
Hematocrit	ALT	Appearance	
Hemoglobin	Total bilirubin	pH	
MCH	Creatinine	Glucose	
MCHC	Urea	Ketones	
MCV	eGFR	Nitrite	
Platelet count	Albumin	Microscopy	
WBC count	Glucose		
WBC differential (basophils, eosinophils, lymphocytes, monocytes and neutrophils)	Potassium		
	Sodium		

Postbaseline hematology and serum chemistry results rated clinically significant by the investigator are summarized with the direction of significance indicated (high or low). Results for eGFR are listed only. For ALT and total bilirubin, any results recorded in the format <X or >X are included in tables as a value of X. These results are listed in the original format. Urinalysis parameters are listed only.

Hematology, serum chemistry, and urinalysis data from all extension parts of Study 2797 (Initial Extension, Pilot Extension, Supplemental Extension) are listed by study stage, treatment group, and subject with values outside of the normal range and clinically significant values flagged. Pregnancy test data are included in SDTM datasets only.

9.9.5 Anti-GDNF Serum Antibodies

The data for anti-GDNF serum antibodies (this section) and plasma GDNF concentrations (Section 9.9.6 below) will not be available until after database lock. In case of a substantial delay, an addendum to the CSR may be written at a later time containing these analyses.

Anti-GDNF binding and neutralizing serum antibody data are reported for screening from Study 2553 (baseline), Weeks 40/e0, 44/e4, 56/e16, 68/e28 and 80/e40; e2-24, e2-48 and e2-72; and the last study visit in the Supplemental Extension. Anti-GDNF serum antibody data are summarized by treatment group with number and percentage of subjects in each category (positive, negative, or not done) by visit. Summary data are also provided for subjects who are positive at any postbaseline visit in Study 2553 or Study 2797 and those who are positive at more than one postbaseline visit in Study 2553 or Study 2797. If a subject has a repeat sample, then the worse result is used in the analysis. A positive result is considered worse than a negative result.

Anti-GDNF binding and neutralizing serum antibody data from all extension parts of Study 2797 (Initial Extension, Pilot Extension, Supplemental Extension) are listed by study stage, treatment group, and subject.

9.9.6 Plasma GDNF Concentrations

Plasma GDNF concentrations are reported for screening from Study 2553 (baseline), Weeks 40/e0, 44/e4, 56/e16, 68/e28 and 80/e40; e2-24, e2-48 and e2-72; and the last study visit in the Supplemental Extension. Plasma GDNF concentrations are summarized by treatment group by comparing postbaseline to baseline values using summary statistics for changes from baseline. Values below the limit of quantitation are not included in summary statistics. If a subject has a repeat sample, then the higher result is used in the analysis.

Plasma GDNF concentration data from all extension parts of Study 2797 (Initial Extension, Pilot Extension, Supplemental Extension) are listed by extension part, treatment group, and subject.

9.9.7 Physical Examination

A brief physical examination is conducted at Week 80/e40 and Week e2-80. Physical examination data are listed by study stage, treatment group, subject, and body system.

9.9.8 Vital Signs

Vital sign evaluations including pulse (sitting and standing), systolic and diastolic blood pressure (sitting and standing), respiration rate, and temperature are reported for all test infusion and study medication infusion visits. During infusion visits, repeated assessments are done pre-dose (baseline), at various time points during infusion, and after the end of infusion.

Frequency tabulations for vital signs display the number and percentage of subjects with clinically relevant abnormalities during or after infusion. Findings are displayed for all test infusion visits (interim

visits after catheter repositioning and Week 80/e40 visits) and study medication infusion visits in the Initial Extension. The criteria for clinically relevant postbaseline abnormalities are outlined in Table 6.

Vital sign data from infusions with a clinically revelant postbaseline abnormal result from all extension parts of Study 2797 (Initial Extension, Pilot Extension, Supplemental Extension) are listed by study stage, treatment group, and subject.

Table 6. Clinically Relevant Postbaseline Abnormalities for Vital Signs Parameters

Parameter	Criterion
Temperature	> 38°C and an increase from pre-dose of at least 1°C
Respiration rate	< 12 or > 20 breaths/min
Pulse	≥ 120 bpm or an increase from pre-dose of > 20 bpm
Pulse	< 50 bpm
Systolic BP	≥ 180 mm Hg or an increase from pre-dose of ≥ 30 mmHg
Systolic BP	< 90 mmHg or a decrease from pre-dose of ≥ 30 mmHg
Diastolic BP	≥ 105 mmHg or an increase from pre-dose of ≥ 20 mmHg
Diastolic BP	< 50 mmHg or a decrease from pre-dose of ≥ 20 mmHg

Pre-dose relates to the pre-infusion value at the respective visit.

9.9.9 Weight

Body weight data from all extension parts of Study 2797 (Initial Extension, Pilot Extension, Supplemental Extension) are listed by study stage, treatment group, and subject.

9.9.10 Electrocardiogram

ECG data are reported at the screening visit from Study 2553 (baseline), Week 40/e0, and Week 80/e40. Quantitative parameters are heart rate (beats/min), PR interval (ms), QRS interval (ms), QT interval (ms), and QTc interval (ms). An overall impression is recorded in the CRF as normal or abnormal and, if abnormal, clinically significant yes (specify abnormality) or no in the investigator's judgment. In addition, objective criteria for clinically relevant QTc abnormalities are defined in Table 7.

Table 7. Clinically Relevant Abnormalities for QTc*

Parameter	Criterion
QTc	> 450 ms
QTc	> 500 ms
QTc change from baseline	> 30 ms
QTc change from baseline	> 60 ms

* Based on ICH E14 guideline.

Quantitative ECG data are tabulated by treatment group, comparing Week 40/e0 and Week 80/e40 to baseline values using summary statistics for changes from baseline.

A categorical table summarizes the number and percentage of subjects with normal and abnormal ECG results at Week 80/e40 (overall and those judged clinically significant by the investigator) and the number and percentage of subjects with Week 80/e40 QTc abnormalities assessed as clinically relevant according to the criteria in Table 7 (overall and per category).

ECG data, both quantitative parameters and overall impression, from the Initial Extension and Pilot Extension of Study 2797 are listed by study stage, treatment group, and subject with flags for clinically significant overall ECG impression and clinically relevant QTc abnormalities.

9.9.11 Glasgow Coma Scale

Glasgow Coma Scale is reported for all test infusion and study medication infusion visits. The assessments are performed before infusion, 30 minutes into the infusion, and after completion of infusion. Glasgow Coma Scale items include visual response, verbal ability, and motor skills. Each item has 4-6 possible responses. The possible responses for each item are listed in Table 8. The best possible total score is 15.

Table 8. Glasgow Coma Scale Scoring

Visual Response	Verbal Ability	Motor Skills
1. No eye opening	1. No verbal response	1. No motor response
2. Eye opening to pain	2. Incomprehensible sounds	2. Extension to pain
3. Eye opening to verbal command	3. Inappropriate words	3. Flexion to pain
4. Eyes open spontaneously	4. Confused	4. Withdrawal from pain
	5. Orientated	5. Localizing pain
		6. Obeys commands

Frequency tabulations display the number and percentage of subjects with a total Glasgow Coma Scale score of 15 or less than 15 at any time during or after infusion. Findings are displayed for all test infusion visits (interim visits after catheter repositioning and Week 80/e40 visits) and study medication infusion visits in the Initial Extension.

Glasgow Coma Scale results < 15 or missing in all extension parts of Study 2797 (Initial Extension, Pilot Extension, Supplemental Extension) are listed by study stage, treatment group, and subject.

9.9.12 Questionnaire for Impulsive-Compulsive Disorders

The QUIP-Current-Full, version 1.0, is a self-administered or informant-completed scale that includes 13 questions covering symptoms related to the 4 commonest impulse control disorders in PD (gambling, sex, buying, and eating) as well as other behaviors and problematic use of medication. The number of "Yes" responses is compared to the number required for a positive result per the scoring sheet. Missing individual responses are imputed using LOCF. Each impulse control disorder, other behavior, and problematic used of medication is rated as a positive result (ie, present) or a negative result (ie, not present).

QUIP data are summarized for both subject and informant parameters in a table comparing postbaseline to baseline values using shifts from baseline by treatment group and responder. QUIP results are summarized for Week 40/e0 (baseline), Weeks 48/e8, 56/e16, 64/e24, 72/e32 and 80/e40; e2-16, e2-32, e2-48, e2-64, and e2-80; e3-16, e3-32, and the last study visit in the Supplemental Extension.

QUIP items answered with "yes" in all extension parts of Study 2797 (Initial Extension, Pilot Extension, Supplemental Extension) are listed by study stage, treatment group, subject, and responder (informant/subject).

9.9.13 Montreal Cognitive Assessment

The MoCA version 7.1 is a rater-administered cognitive screening tool with 8 components: visuospatial/executive, naming, memory, attention, language, abstraction, delayed recall, and orientation. The total score ranges from 0 to 30, with lower scores representing poorer cognitive function. A total

score of 26 or above is considered normal. The MoCA will be analyzed using total score only. Missing individual scores are imputed using LOCF if necessary.

MoCA data are summarized in a table comparing postbaseline to baseline values using summary statistics for changes from baseline by treatment group. MoCA results are summarized for screening, pre-test infusion (baseline), Weeks 40/e0, 56/e16, and 80/e40; e2-80; and the last study visit in the Supplemental Extension.

MoCA total score data from all extension parts of Study 2797 (Initial Extension, Pilot Extension, Supplemental Extension) are listed by study stage, treatment group, and subject.

9.9.14 Mattis Dementia Rating Scale

The MDRS version 2 is a rater-administered global scale of cognition including 5 subscales: attention, initiation/perseveration, construction, conceptualization, and memory. Scores range from 0 to 144, with higher scores representing better cognitive function. In PD, scores <123 are associated with some degree of dementia. The age- and education-corrected Mayo Older Adults Normative Studies (MOANS) scaled score (AEMSS) total score ranges from 0 to 20, with higher scores representing better cognitive function. The MDRS will be analyzed using AEMSS total score only as entered in the CRF.

MDRS data are summarized in a table comparing postbaseline to baseline values using summary statistics for changes from baseline by treatment group. MDRS results are summarized for screening (baseline), Weeks 40/e0, 56/e16, and 80/e40; e2-80; and the last study visit in the Supplemental Extension.

MDRS AEMSS total score data from all extension parts of Study 2797 (Initial Extension, Pilot Extension, Supplemental Extension) are listed by study stage, treatment group, and subject.

9.9.15 Stroop Test

The Stroop test is a global scale of reaction time including 4 conditions: color naming, word reading, inhibition, and inhibition/switching. Total time to complete the test in each condition can range from 0 to 999 seconds, with lower time representing better reaction time. The Stroop test will be analyzed separately in each condition.

Stroop test data are summarized in a table comparing postbaseline to baseline values using summary statistics for changes from baseline by treatment group. Stroop test results are summarized for screening (baseline), Week 40/e0, and Week 80/e40.

Stroop test data are listed by study stage, treatment group, and subject.

9.9.16 Frontal Systems Behavioural Scale

The FrSBe is a scale that assesses behavior related to frontal systems damage including 3 subscales: apathy, disinhibition, and executive dysfunction. Higher subscale scores indicate greater pathology. Total score is not analyzed for this study. Individual missing items are imputed using the average of non-missing scores in each subscale.

FrSBe data are summarized in a table comparing postbaseline (Week 40/e0 “after” and Week 80/e40 “after”) to baseline values (“after” values for Screening Visit 2 from Study 2553) for each subscale using summary statistics for changes from baseline by treatment group. FrSBe results are summarized for screening (before and after [baseline]), Week 40/e0, and Week 80/e40.

FrSBe test data are listed by study stage, treatment group, and subject.

9.9.17 Deary-Liewald Reaction Time

The Deary-Liewald RT is a computerized measure of simple and four-choice RT. The parameter is the mean reaction time, variance and SD for correct responses for four-choice RT. A shorter reaction time is better.

Deary-Liewald four-choice RT data are summarized in a table comparing postbaseline to baseline values using summary statistics for changes from baseline by treatment group. Deary-Liewald RT results are summarized for Screening Visit 2 (baseline), Week 40/e0, Week 56/e16, and Week 80/e40.

Four-choice RT test data are listed by study stage, treatment group, and subject. Simple RT data are not listed, but are included in SDTM datasets.

9.9.18 Verbal Fluency Assessment

The verbal fluency assessment is a test of verbal functioning in 2 categories: phonemic and semantic. Scores (number of correct words in one minute) range from 0 to 200, with a higher score representing better verbal functioning. Verbal fluency will be analyzed by number of correct responses in each category.

Verbal fluency data are summarized in a table comparing postbaseline to baseline values using summary statistics for changes from baseline by treatment group. Verbal fluency results are summarized for screening (baseline), Week 40/e0, and Week 80/e40. Parameters are phonemic fluency score and semantic fluency score.

Verbal fluency assessment test data are listed by study stage, treatment group.

9.9.19 Beck Depression Inventory

The Beck Depression Inventory (BDI) is a self-administered test which consists of 21 questions that measure the severity of depression. Scores range from 0 to 63, with higher scores representing worse depression. Individual missing items are imputed using the average of non-missing scores in each subscale. The total score is the parameter analyzed.

BDI data are summarized in a table comparing postbaseline to baseline values using summary statistics for changes from baseline by treatment group. BDI results are summarized for screening (baseline), Week 40/e0, Week 80/e40; and the last study visit in both the Pilot Extension and the Supplemental Extension.

BDI total score data from all extension parts of Study 2797 (Initial Extension, Pilot Extension, Supplemental Extension) are listed by study stage, treatment group, and subject.

9.9.20 University of Pennsylvania Smell Identification Test

The UPSIT is a self-administered test which can be used to identify and quantitate olfactory dysfunction in PD. The number of correct responses out of 40 total items constitutes a subject’s score with interpretations as shown in Table 9. Lower scores represent greater olfactory dysfunction. Individual missing responses are imputed as zeros (ie, incorrect responses).

Table 9. UPSIT Score Interpretation

Test Score (Males)	Test Score (Females)	Olfactory Diagnosis
0 – 5	0 – 5	Probable malingering
6-18	6-18	Total anosmia
19-25	19-25	Severe microsmia
26-29	26-30	Moderate microsmia
30-33	31-34	Mild microsmia
34-40	35-40	Normosmia

UPSIT data are summarized in a table comparing postbaseline to baseline values using summary statistics for changes from baseline by treatment group. UPSIT results are summarized for screening (baseline), Week 40/e0, and Week 80/e40.

UPSIT test data from the Initial Extension and Pilot Extension of Study 2797 are listed by study stage, treatment group, and subject.

10.0 Validation

PRA's goal is to ensure that each TFL delivery is submitted to the highest level of quality. Our quality control procedures will be documented separately in the study specific quality control plan.

Appendix 1 Glossary of Abbreviations

**aCSF**	Artificial Cerebrospinal Fluid
**ADL**	Activities of Daily Living
**AE**	Adverse Event
**AESI**	Adverse Event of Special Interest
**ALT**	Alanine Aminotransferase
**ANCOVA**	Analysis of Covariance
**ATC**	Anatomical Therapeutic Chemical
**BDI**	Beck Depression Inventory
**BP**	Blood Pressure
**CDISC**	Clinical Data Interchange Standards Consortium
**CED**	Convection-Enhanced Delivery
**CI**	Confidence Interval
**COMT**	Catechol-O-methyl transferase
**CRF**	Case Report Form
**ECG**	Electrocardiogram
**eGFR**	Estimated Glomerular Filtration Rate
**EQ-5D**	EuroQOL 5-dimensional Scale
**FLAIR**	Fluid-attenuated Inversion Recovery
**FrSBe**	Frontal Systems Behavioural Scale
**GDNF**	Glial Cell Line-derived Neurotrophic Factor
**ITT**	Intent-To-Treat
**LOCF**	Last Observation Carried Forward
**MCH**	Mean Corpuscular Hemoglobin
**MCHC**	Mean Corpuscular Hemoglobin Concentration
**MCV**	Mean Corpuscular Volume
**MDRS**	Mattis Dementia Rating Scale
**MedDRA**	Medical Dictionary for Regulatory Activities
**MHRA**	Medicines and Healthcare Products Regulatory Agency
**MMRM**	Mixed-effect Model with Repeated Measures
**MoCA**	Montreal Cognitive Assessment
**MRI**	Magnetic Resonance Imaging
**NBT**	North Bristol National Health System Trust

**NMSS**	Non-Motor Symptom Assessment Scale
**PD**	Parkinson's Disease
**PDQ-39**	Parkinson's Disease Questionnaire-39
**PRA**	Pharmaceutical Research Associates
**PT**	Preferred Term
**QUIP**	Questionnaire for Impulsive-Compulsive Disorders in Parkinson's Disease
**RBC**	Red Blood Cells
**RT**	Reaction Time
**SAE**	Serious Adverse Event
**SAP**	Statistical Analysis Plan
**SD**	Standard Deviation
**SDTM**	Standard Data Tabulation Model
**SE**	Standard Error
**SNAQ**	Simplified Nutritional Appetite Questionnaire
**SOC**	System Organ Class
**TEAE**	Treatment-Emergent Adverse Event
**TFL**	Tables, Figures, and Listings
**UPDRS**	Unified Parkinson's Disease Rating Scale
**UPSIT**	University of Pennsylvania Smell Identification Test
**VOI**	Volume of Interest
**WBC**	White Blood Cells
**WHODRUG DDE**	World Health Organization Drug Dictionary Enhanced

Appendix 2 Protocol Deviation Guidance

<<To be updated prior to finalizing SAP v2>>

Definitions

A protocol deviation is any change, divergence, or departure from the study design or procedures defined in the protocol.

Major protocol deviations are a subset of protocol deviations that might significantly affect a subject's rights, safety, or well-being or that might significantly affect the completeness, accuracy, and/or reliability of core study data. For example, major protocol deviations might include enrolling subjects in violation of key eligibility criteria designed to ensure a specific subject population or failing to collect data necessary to interpret the primary endpoint, as this may compromise the scientific value of the trial.

Minor deviations are deviations that do not meet the above criteria, i.e. do not impact subject safety or the scientific integrity of the study.

Examples of major and minor protocol violations are provided in the table below.

Major Deviations	Minor Deviations
Key eligibility criteria violated	Visit outside of window
Subject missed more than one study treatment	Protocol required assessment not completed (except for OFF state UPDRS motor score at baseline and/or Week 80/e40)
Subject significantly overdosed	Failure to report SAE in required timeframe
Baseline and/or Week 80/e40 OFF state UPDRS motor score not completed properly	Entire visit not conducted (other than Baseline and Week 80/e40)
Failure to obtain informed consent from subject	Lab sample preparation or storage not adequate (anti-GDNF antibodies and plasma concentration of GDNF)

As per the study protocol, the primary analysis will be an intention-to-treat (ITT) analysis including all subjects enrolled in the Primary Stage (ITT Primary Population).

Process

From study start until January 2015, protocol deviations were documented in file notes by the study staff, and provided to PRA for entry into their Clinical Trials Management System (see Memo to File from Robert Appel dated 03-Jun-2014, RE: Project Plan: Planned Process Deviations process). Starting in January 2015, Case Report Forms (CRFs) were created to capture protocol deviations for entry into the clinical database. Protocol deviations previously captured in file notes will be transcribed to the Protocol Deviation CRF.

Prior to database lock, all protocol deviations will be reviewed to assess the deviation classification (major/minor) and category (categories and guidance are provided in the table below). For this review, the adjudication team will use the list of protocol deviations generated from the clinical database as an input, and the list of subjects in the ITT population from the Biostatistician or Analysis Programmer. Final determination of the deviation classification (major or minor) will be made by the Study Sponsor in view of the recommendations made by the adjudication team.

**Category**	**Guidance**
Inclusion criteria	Any deviation relating to inclusion criteria not being met
Exclusion criteria	Any deviation relating to exclusion criteria being met
Study medication (including overdose)	Any deviation relating to the administration of study medication. This includes GDNF/aCSF only and does not include test infusions or medications given for levodopa challenge.
Non-study medication	Any deviation relating to medication other than study medication. This includes concomitant medications and PD medications.
Study schedule/visit windows	Any deviation relating to an assessment which was completed, but outside the visit window or incorrectly completed. An assessment refers to efficacy, safety or imaging.
Outcome assessment	Any deviation relating to an assessment which was entirely missed. An assessment refers to efficacy, safety or imaging.
Other, specify	Deviations which clearly do not fit in any of the other categories.

Appendix 3 List of MedDRA Preferred Terms for Adverse Events of Special Interest

<<To be updated prior to finalizing SAP v2>>

Preferred Terms Related to Adverse Changes in Mood:

SOC Psychiatric disorders

- Agitation
- Anxiety
- Depressed mood
- Suicidal ideation
- Tearfulness

Preferred Terms Related to Impulsivity:

SOC Psychiatric disorders

- Compulsive shopping
- Hypersexuality
- Impulsive behaviour
- Libido increased
- Obsessive-compulsive disorder

Appendix 4 List of Conversion Factors for the Calculation of Levodopa and Levodopa Equivalent Doses

PD Medication	Conversion Factor
**Levodopa Preparations**	
Immediate release preparations without COMT inhibition	1.0
Immediate release preparations with entacapone	1.33
Immediate release preparations with tolcapone	1.5
Controlled release preparations	0.75
Levodopa/carbidopa (Duodopa)	1.11
**MAO-B Inhibitors**	
Selegiline oral	10
Selegiline sublingual	80
Rasagiline	100
**Dopamine Agonists**	
Ropinirole immediate release	20
Ropinirole long acting	20
Pramipexole immediate release (base)	140
Pramipexole immediate release (salt)	100
Pramipexole long acting (base)	140
Pramipexole long acting (salt)	100
Cabergoline	70
Rotigotine	30
Piribedil	1
Apomorphine	10
Bromocriptine	10
Pergolide	100
Lisuride	100
Dihydroergocryptine (DHEC)	5
**Other**	
Amantadine	1

Note: COMT inhibitor doses are not included in the calculation of levodopa or levodopa equivalent doses. They contribute to the levodopa or levodopa equivalent dose by modifying the dose of concurrently administered levodopa.

Appendix 5 List of Post-Text Tables, Figures and Listings

16.1 DEMOGRAPHIC DATA TABLES 65
Table 16.1.1.1 Subject Populations - All Enrolled Subjects 65
Table 16.1.1.2.1 Subject Disposition - ITT Primary Population 66
Table 16.1.1.2.2 Subject Disposition - ITT Overall Population 67
Table 16.1.2 Protocol Deviations - ITT Overall Population 68
Table 16.1.3.1 Demographic Characteristics from Study 2553 - ITT Primary Population 69
Table 16.1.3.2 Demographic Characteristics from Study 2553 - ITT Overall Population 71
Table 16.1.4.1 Parkinson's Disease History at Screening from Study 2553 - ITT Primary Population 72
Table 16.1.4.2 Parkinson's Disease History at Screening from Study 2553 - ITT Overall Population 74
Table 16.1.5.1 Concomitant Parkinson's Disease Medications by WHO Drug Dictionary ATC Class and Preferred Name - ITT Primary Population 75
Table 16.1.5.2 Other Concomitant Medications by WHO Drug Dictionary ATC Class and Preferred Name - ITT Primary Population 76
16.2 EFFICACY DATA TABLES 77
16.2.1 UPDRS PRIMARY EFFICACY TABLES 77
Table 16.2.1.1 OFF State UPDRS Motor Score (Part III): Change and Percentage Change from Baseline to Week 80/e40 – MMRM, ITT Primary Population 78
Table 16.2.1.2 OFF State UPDRS Motor Score (Part III): Change and Percentage Change from Baseline to Week 80/e40 – MMRM, ITT Overall Population 79
Table 16.2.2.1 ON State UPDRS Motor Score (Part III): Change and Percentage Change from Baseline to Week 80/e40 – MMRM, ITT Overall Population 80
Table 16.2.2.2.1 OFF State UPDRS ADL Score (Part II): Change and Percentage Change from Baseline to Week 80/e40 – MMRM, ITT Primary Population 80
Table 16.2.2.2.2 OFF State UPDRS ADL Score (Part II): Change and Percentage Change from Baseline to Week 80/e40 – MMRM, ITT Overall Population 80
Table 16.2.2.3 ON State UPDRS ADL Score (Part II): Change and Percentage Change from Baseline to Week 80/e40 – MMRM, ITT Overall Population 80
Table 16.2.2.4.1 OFF State UPDRS Total Score: Change and Percentage Change from Baseline to Week 80/e40 – MMRM, ITT Primary Population 81
Table 16.2.2.4.2 OFF State UPDRS Total Score: Change and Percentage Change from Baseline to Week 80/e40 – MMRM, ITT Overall Population 81
Table 16.2.2.5 ON State UPDRS Total Score: Change and Percentage Change from Baseline to Week 80/e40 – MMRM, ITT Overall Population 81
Table 16.2.2.6.1 OFF State UPDRS Motor Score (Part III): Change from Baseline to Week 40/e0 for GDNF/GDNF Group Compared to Change from Baseline to Week 80/e40 for Placebo/GDNF Group – MMRM, ITT Overall Population 82
Table 16.2.2.6.2 OFF State UPDRS ADL Score (Part II): Change from Baseline to Week 40/e0 for GDNF/GDNF Group Compared to Change from Baseline to Week 80/e40 for Placebo/GDNF Group – MMRM, ITT Overall Population 83
Table 16.2.2.6.3 OFF State UPDRS Total Score: Change from Baseline to Week 40/e0 for GDNF/GDNF Group Compared to Change from Baseline to Week 80/e40 for Placebo/GDNF Group – MMRM, ITT Overall Population 83

Table 16.2.2.7.1 OFF State UPDRS Motor Score (Part III): Change from Baseline to Week 80/e40 for GDNF/GDNF Group Compared to Change from Baseline to Week 40/e0 for Placebo/GDNF Group – MMRM, ITT Overall Population 83
Table 16.2.2.7.2 OFF State UPDRS ADL Score (Part II): Change from Baseline to Week 80/e40 for GDNF/GDNF Group Compared to Change from Baseline to Week 40/e0 for Placebo/GDNF Group – MMRM, ITT Overall Population 83
Table 16.2.2.7.3 OFF State UPDRS Total Score: Change from Baseline to Week 80/e40 for GDNF/GDNF Group Compared to Change from Baseline to Week 40/e0 for Placebo/GDNF Group – MMRM, ITT Overall Population 83
16.2.3 PD DIARY SECONDARY EFFICACY TABLES 84
Table 16.2.3.1 Motor Fluctuation Diary Ratings by Visit: Change from Baseline – MMRM, ITT Primary Population 84
Table 16.2.3.2 Motor Fluctuation Diary Ratings by Visit: Change from Baseline – MMRM, ITT Overall Population 86
Table 16.2.3.3.1 Treatment Response at Week 40/e0 and Week 80/e40 - ITT Overall Population 87
Table 16.2.3.3.2 UPDRS Responder at Week 80/e40 Compared to Week 40/e0 - ITT Overall Population 88
16.2.4 SUPPLEMENTARY EFFICACY TABLES 89
Table 16.2.4.1 OFF and ON State Timed Walking Test by Visit: Change from Baseline – MMRM, ITT Overall Population 89
Table 16.2.4.2 OFF and ON State Timed Tapping Test by Visit: Change from Baseline – MMRM, ITT Overall Population 91
Table 16.2.4.3 NMSS Score by Visit: Change from Baseline – MMRM, ITT Overall Population 92
Table 16.2.4.4 PDQ-39 Score: Change from Baseline to Week 80/e40 –ANCOVA, ITT Overall Population 93
Table 16.2.4.5.1 EQ-5D Questionnaire: Baseline, Week 40/e0, and Week 80/e40 – Observed Data, ITT Overall Population 95
Table 16.2.4.5.2 EQ-5D Visual Analog Scale: Change from Baseline to Week 80/e40 – ANCOVA, ITT Overall Population 96
Table 16.2.4.6 SNAQ Score: Change from Baseline to Week 80/e40 –ANCOVA, ITT Overall Population 96
Table 16.2.4.7 Total Daily Levodopa Dose (mg): Change From Baseline to Week 80/e40 – ANCOVA, ITT Overall Population 97
Table 16.2.4.8 Total Daily Levodopa Equivalent Dose (mg): Change From Baseline to Week 80/e40 –ANCOVA, ITT Overall Population 98
16.3 IMAGING TABLES 99
Table 16.3.1 Volume of Distribution of Infusate as Determined by Contrast-Enhanced T1-Weighted MRI: Change from Baseline to Week 80/e40 –ANCOVA, ITT Primary Population 99
Table 16.3.2.1 Volume of Interest Coverage as Determined by Contrast-Enhanced T1-Weighted MRI: Change from Baseline to Week 80/e40 –ANCOVA, ITT Primary Population 99
Table 16.3.2.2 Total Putamenal Coverage as Determined by Contrast-Enhanced T1-Weighted MRI: Change from Baseline to Week 80/e40 –ANCOVA, ITT Primary Population 99
Table 16.3.3.1 Correlation Analyses of Percentage Change from Baseline to Week 80/e40 in OFF State UPDRS Motor Score (Part III) to Volume of Interest Coverage and Total Putamenal Coverage at Baseline as Determined by Contrast-Enhanced T1-Weighted MRI – ITT Primary Population 100

Table 16.3.3.2 Correlation Analysis of Percentage Change from Baseline to Week 80/e40 in OFF State UPDRS Motor Score (Part III) to Change from Baseline to Week 40/e0 in ^{18}F-DOPA Uptake as Determined by PET Scan – ITT Primary Population 101
Table 16.3.3.3 Correlation Analysis of Percentage Change from Baseline to Week 80/e40 in OFF State UPDRS Motor Score (Part III) to Change from Baseline to Week 40/e0 in ^{18}F-DOPA Uptake as Determined by PET Scan – ITT Overall Population 101
16.4 Safety Data Tables 102
16.4.1 Exposure Data Tables 102
Table 16.4.1.1 Exposure to Study Medication - Safety Overall Population 102
Table 16.4.1.2 Study Medication Infusion Details by Visit - Safety Overall Population 104
16.4.2 Adverse Event Tables 105
Table 16.4.2.1 Overall Summary of Adverse Events - Safety Overall Population 105
Table 16.4.2.2 Treatment-Emergent Adverse Events by System Organ Class and Preferred Term - Safety Overall Population 106
Table 16.4.2.3 Treatment-Emergent Adverse Events Experienced by at Least 3 Subjects Overallby Preferred Term - Safety Overall Population 107
Table 16.4.2.4 Treatment-Emergent Adverse Events by System Organ Class, Preferred Term, and Maximum Severity - Safety Overall Population 108
Table 16.4.2.5 Serious Treatment-Emergent Adverse Events by System Organ Class and Preferred Term - Safety Overall Population 110
Table 16.4.2.6 Study Medication-Related Treatment-Emergent Adverse Events by System Organ Class and Preferred Term - Safety Overall Population 111
Table 16.4.2.7 Serious Study Medication-Related Treatment-Emergent Adverse Events by System Organ Class and Preferred Term - Safety Overall Population 111
Table 16.4.2.8 Device-Related Treatment-Emergent Adverse Events by System Organ Class and Preferred Term - Safety Overall Population 111
Table 16.4.2.9 Serious Device-Related Treatment-Emergent Adverse Events by System Organ Class and Preferred Term - Safety Overall Population 111
Table 16.4.2.10 Treatment-Emergent Adverse Events of Special Interest by Preferred Term - Safety Overall Population 112
16.4.3 Laboratory Tables 113
Table 16.4.3.1 Clinically Significant Postbaseline Hematology Results - Safety Overall Population 113
Table 16.4.3.2 Clinically Significant Postbaseline Serum Chemistry Results - Safety Overall Population 114
16.4.4 Anti-GDNF Serum Antibody Tables 115
Table 16.4.4.1 Anti-GDNF Binding Serum Antibodies by Visit – Safety Overall Population 115
Table 16.4.4.2 Anti-GDNF Neutralizing Serum Antibodies by Visit - Safety Overall Population 116
16.4.5 Plasma GDNF Concentration Tables 117
Table 16.4.5 Plasma GDNF Concentrations by Visit - Safety Overall Population 117
16.4.6 Vital Sign Table 118
Table 16.4.6 Clinically Relevant Postbaseline Abnormalities in Vital Sign Results in the Initial Extension - Safety Overall Population 118
16.4.7 Electrocardiogram Tables 119
Table 16.4.7.1 Electrocardiogram Results by Visit – Safety Overall Population 119
Table 16.4.7.2 Summary of Electrocardiogram Results at Week 80/e40 - Safety Overall Population 120

16.4.8 GLASGOW COMA SCALE TABLE 121
Table 16.4.8 Glasgow Coma Scale Score = 15 or < 15 During or After Infusion by Visit and Time Point in the Initial Extension– Safety Overall Population 121
16.4.9 OTHER SAFETY TABLES 122
Table 16.4.9.1 QUIP by Visit - Safety Overall Population 122
Table 16.4.9.2 MoCA by Visit - Safety Overall Population 124
Table 16.4.9.3 MDRS by Visit - Safety Overall Population 124
Table 16.4.9.4 Stroop Test by Visit, Safety Overall Population 124
Table 16.4.9.5 FrSBe by Visit - Safety Overall Population 124
Table 16.4.9.6 Deary-Liewald Four-Choice Reaction Time by Visit - Safety Overall Population 124
Table 16.4.9.7 Verbal Fluency Assessment by Visit - Safety Overall Population 124
Table 16.4.9.8 BDI by Visit - Safety Overall Population 125
Table 16.4.9.9 UPSIT by Visit - Safety Overall Population 125
16.5 FIGURES 126
Figure 16.5.1.1.1 OFF State UPDRS Motor Score (Part III): Percentage Change Over Time - ITT Primary Population 126
Figure 16.5.1.1.2 OFF State UPDRS Motor Score (Part III): Percentage Change Over Time - ITT Overall Population 128
Figure 16.5.1.2.1 OFF State UPDRS Motor Score (Part III): Change Over Time - ITT Primary Population 128
Figure 16.5.1.2.2 OFF State UPDRS Motor Score (Part III): Change Over Time - ITT Overall Population 128
Figure 16.5.1.3 ON State UPDRS Motor Score (Part III): Change Over Time - ITT Overall Population 128
Figure 16.5.1.4.1 OFF State UPDRS ADL Score (Part II): Change Over Time - ITT Primary Population 128
Figure 16.5.1.4.2 OFF State UPDRS ADL Score (Part II): Change Over Time - ITT Overall Population 128
Figure 16.5.1.5 ON State UPDRS ADL Score (Part II): Change Over Time - ITT Overall Population 128
Figure 16.5.1.6.1 OFF State UPDRS Total Score: Change Over Time - ITT Primary Population 128
Figure 16.5.1.6.2 OFF State UPDRS Total Score: Change Over Time - ITT Overall Population 129
Figure 16.5.1.7 ON State UPDRS Total Score: Change Over Time - ITT Overall Population 129
Figure 16.5.2.1 Motor Fluctuation Diary Total OFF Time Per Day (Hours): Change Over Time - ITT Primary Population 130
Figure 16.5.2.2 Motor Fluctuation Diary Total Good-Quality ON Time Per Day (Hours): Change Over Time - ITT Primary Population 130
Figure 16.5.3 OFF State UPDRS Motor Score (Part III): Subject Scores Over Time - ITT Primary Population 130
Figure 16.5.4.1 OFF State UPDRS Motor Score (Part III): Frequency Distribution of Change at Week 40/e0 - ITT Overall Population 131
Figure 16.5.4.2 OFF State UPDRS Motor Score (Part III): Frequency Distribution of Change at Week 80/e40 - ITT Overall Population 131
Figure 16.5.5.1 Correlation Analysis of Percentage Change from Baseline to Week 80/e40 in OFF State UPDRS Motor Score (Part III) to Volume of Interest Coverage at Baseline as Determined by Contrast-Enhanced T1-Weighted MRI - ITT Primary Population 132

Figure 16.5.5.2 Correlation Analysis of Percentage Change from Baseline to Week 80/e40 in OFF State UPDRS Motor Score (Part III) to Total Putamenal Coverage at Baseline as Determined by Contrast-Enhanced T1-Weighted MRI - ITT Primary Population 133
Figure 16.5.5.3 Correlation Analysis of Percentage Change from Baseline to Week 80/e40 in OFF State UPDRS Motor Score (Part III) to Change From Baseline to Week 40/e0 in ^{18}F-DOPA Uptake as Determined by PET Scan - ITT Primary Population 134
Figure 16.5.5.4 Correlation Analysis of Baseline (Week 0) OFF State UPDRS Motor Score (Part III) to Baseline (Week 0) ^{18}F-DOPA Uptake as Determined by PET Scan for GDNF/GDNF Subjects - ITT Overall Population 134
Figure 16.5.5.5 Correlation Analysis of Baseline (Week 0) OFF State UPDRS Motor Score (Part III) to Baseline (Week 0) ^{18}F-DOPA Uptake as Determined by PET Scan for Placebo/GDNF Subjects - ITT Overall Population 134
Figure 16.5.5.6 Correlation Analysis of Baseline (Week 0) OFF State UPDRS ADLScore (Part II) to Baseline (Week 0) ^{18}F-DOPA Uptake as Determined by PET Scan for GDNF/GDNF Subjects - ITT Overall Population 134
Figure 16.5.5.7 Correlation Analysis of Baseline (Week 0) OFF State UPDRS ADL Score (Part II) to Baseline (Week 0) ^{18}F-DOPA Uptake as Determined by PET Scan for Placebo/GDNF Subjects - ITT Overall Population 134
Figure 16.5.5.8 Correlation Analysis of Week 40/e0 OFF State UPDRS Motor Score (Part III) to Week 40/e0 ^{18}F-DOPA Uptake as Determined by PET Scan for GDNF/GDNF Subjects - ITT Overall Population 134
Figure 16.5.5.9 Correlation Analysis of Week 40/e0 OFF State UPDRS Motor Score (Part III) to Week 40/e0 ^{18}F-DOPA Uptake as Determined by PET Scan for Placebo/GDNF Subjects - ITT Overall Population 135
Figure 16.5.5.10 Correlation Analysis of Week 40/e0 OFF State UPDRS ADLScore (Part II) to Week 40/e0 ^{18}F-DOPA Uptake as Determined by PET Scan for GDNF/GDNF Subjects - ITT Overall Population 135
Figure 16.5.5.11 Correlation Analysis of Week 40/e0 OFF State UPDRS ADL Score (Part II) to Week 40/e0 ^{18}F-DOPA Uptake as Determined by PET Scan for Placebo/GDNF Subjects - ITT Overall Population 135
Figure 16.5.5.12 Correlation Analysis of Week 80/e40 OFF State UPDRS Motor Score (Part III) to Week 40/e0 ^{18}F-DOPA Uptake as Determined by PET Scan for GDNF/GDNF Subjects - ITT Overall Population 135
Figure 16.5.5.13 Correlation Analysis of Week 80/e40 OFF State UPDRS Motor Score (Part III) to Week 40/e0 ^{18}F-DOPA Uptake as Determined by PET Scan for Placebo/GDNF Subjects - ITT Overall Population 135
Figure 16.5.5.14 Correlation Analysis of Week 80/e40 OFF State UPDRS ADLScore (Part II) to Week 40/e0 ^{18}F-DOPA Uptake as Determined by PET Scan for GDNF/GDNF Subjects - ITT Overall Population 135
Figure 16.5.5.15 Correlation Analysis of Week 80/e40 OFF State UPDRS ADL Score (Part II) to Week 40/e0 ^{18}F-DOPA Uptake as Determined by PET Scan for Placebo/GDNF Subjects - ITT Overall Population 136
17 DATA LISTINGS 137
17.2.1 BASELINE LISTINGS 137
Listing 17.2.1.1 Study Completion Status - ITT Overall Population 137
Listing 17.2.1.2.1 Major Protocol Deviations - ITT Overall Population 138
Listing 17.2.1.2.2 Minor Protocol Deviations - ITT Overall Population 139

Listing 17.2.1.3 Subject Populations - ITT Overall Population 140
Listing 17.2.1.4.1 Demographic Characteristics in Study 2553 - ITT Overall Population 141
Listing 17.2.1.4.2 Parkinson's Disease History at Screening in Study 2553 - ITT Overall Population 142
Listing 17.2.1.5 Concomitant Medications – ITT Overall Population 143
Listing 17.2.1.6 Catheter Trajectory - ITT Overall Population 144
Listing 17.2.1.7 Catheter Positioning Accuracy by Surgery - ITT Overall Population 145
Listing 17.2.1.8 Test Infusion Data by Visit - ITT Overall Population 146
Listing 17.2.1.9 Test Infusion Catheter Interruptions/Early Terminations by Visit - ITT Overall Population 147
17.2.2 EFFICACY LISTINGS 148
Listing 17.2.2.1 OFF and ON state UPDRS Scores by Visit - ITT Overall Population 148
Listing 17.2.2.2 PD Motor Fluctuation Diary Ratings by Visit - ITT Overall Population 150
Listing 17.2.2.3 Treatment Response at Week 40/e0 and Week 80/e40 - ITT Overall Population 151
Listing 17.2.2.4.1 OFF and ON State Timed Walking Test by Visit - ITT Overall Population 153
Listing 17.2.2.4.2 OFF and ON State Timed Tapping Test by Visit - ITT Overall Population 154
Listing 17.2.2.5 NMSS Scores by Visit - ITT Overall Population 155
Listing 17.2.2.6 PDQ-39 Scores by Visit - ITT Overall Population 155
Listing 17.2.2.7 EQ-5D Scores by Visit - ITT Overall Population 155
Listing 17.2.2.8 SNAQ Scores by Visit - ITT Overall Population 155
Listing 17.2.2.9.1 Levodopa and Levodopa Equivalent Medication Actual Total Daily Doses - ITT Overall Population 156
Listing 17.2.2.9.2 Effective Levodopa and Levodopa Equivalent Medication Total Daily Doses - ITT Overall Population 158
17.2.3 IMAGING LISTINGS 159
Listing 17.2.3.1 Volume of Distribution, Volume of Interest Coverage, and Total Putamenal Coverage as Determined by Contrast-Enhanced T1-Weighted MRI by Visit - ITT Overall Population 159
17.2.4 SAFETY LISTINGS 160
Listing 17.2.4.1.1 Exposure to Study Medication - Safety Overall Population 160
Listing 17.2.4.1.2 Study Medication Infusion Data by Visit - Safety Overall Population 161
Listing 17.2.4.1.3 Study Medication Infusion Catheter Interruptions/Early Terminations by Visit - Safety Overall Population 162
Listing 17.2.4.2 Adverse Events - Safety Overall Population 163
Listing 17.2.4.3.1 Adverse Events of Special Interest: Dyskinesias- Safety Overall Population 164
Listing 17.2.4.3.2 Adverse Events of Special Interest: Falls- Safety Overall Population 164
Listing 17.2.4.3.3 Adverse Events of Special Interest: Adverse Changes in Mood- Safety Overall Population 164
Listing 17.2.4.3.4 Adverse Events of Special Interest: Impulsivity- Safety Overall Population 164
Listing 17.2.4.4 Port Symptoms by Visit - Safety Overall Population 165
Listing 17.2.4.5 Hematology Results by Visit - Safety Overall Population 166
Listing 17.2.4.6 Serum Chemistry Results by Visit - Safety Overall Population 167
Listing 17.2.4.7 Urinalysis Results by Visit - Safety Overall Population 167
Listing 17.2.4.8 Anti-GDNF Serum Antibodies - Safety Overall Population 168
Listing 17.2.4.9 Plasma GDNF Concentration Results by Visit - Safety Overall Population 169
Listing 17.2.4.10 Physical Examination - Safety Overall Population 170

Listing 17.2.4.11 Vital Signs from Infusions with a Clinically Revelant Postbaseline Abnormal Result by Visit and Time Point - Safety Overall Population 172
Listing 17.2.4.12 Weight by Visit - Safety Overall Population 173
Listing 17.2.4.13 Electrocardiogram Results by Visit – Continuous Parameters, Safety Overall Population 173
Listing 17.2.4.14 Electrocardiogram Results by Visit – Overall Impression, Safety Overall Population 174
Listing 17.2.4.15 Glasgow Coma Scale Results < 15 by Visit - Safety Overall Population 175
Listing 17.2.4.16 QUIP Items Answered with Yes by Visit - Safety Overall Population 176
Listing 17.2.4.17 MoCA Total Score by Visit - Safety Overall Population 177
Listing 17.2.4.18 MDRS AEMSS Total Score by Visit - Safety Overall Population 178
Listing 17.2.4.19 Stroop Test by Visit - Safety Overall Population 179
Listing 17.2.4.20 FrSBe by Visit - Safety Overall Population 180
Listing 17.2.4.21 Deary-Liewald Four-Choice Reaction Time by Visit - Safety Overall Population 181
Listing 17.2.4.22 Verbal Fluency Assessment by Visit - Safety Overall Population 182
Listing 17.2.4.23 BDI by Visit - Safety Overall Population 183
Listing 17.2.4.24 UPSIT by Visit - Safety Overall Population 184

Appendix 6 Shells for Post-Text Tables, Figures and Listings

16.1 Demographic Data Tables

Table 16.1.1.1 Subject Populations - All Enrolled Subjects

Population	GDNF/GDNF	Placebo/GDNF	Total
INITIAL EXTENSION			
Pilot Stage	xx	xx	xx
Subjects enrolled [n]	xx	xx	xx
Subjects treated [n]			
Primary Stage			
Subjects enrolled (ITT Primary Population) [n]	xx	xx	xx
Subjects treated [n]	xx	xx	xx
Overall (Pilot + Primary Stage)			
Subjects enrolled (ITT Overall Population) [n]	xx	xx	xx
Subjects treated (Safety Overall Population) [n]	xx	xx	xx
PILOT EXTENSION (Pilot Stage subjects only)			
Subjects enrolled [n]	xx	xx	xx
Subjects treated [n]	xx	xx	xx
SUPPLEMENTAL EXTENSION			
Pilot Stage	xx	xx	xx
Subjects enrolled [n]	xx	xx	xx
Subjects treated [n]			
Primary Stage			
Subjects enrolled [n]	xx	xx	xx
Subjects treated [n]	xx	xx	xx
Overall (Pilot + Primary Stage)			
Subjects enrolled [n]	xx	xx	xx
Subjects treated [n]	xx	xx	xx

Source: Listing xx.x.xx, Dataset: [NAME], Program: xxxxxx.sas, Output: T_16_1_1_1_XXXXX.rtf, Generated on: DDMONYYYY HH:MM
Page 1 of 1

Table 16.1.1.2.1 Subject Disposition - ITT Primary Population

Variable	GDNF/GDNF (N = XXX) n (%)	Placebo/GDNF (N = XXX) n (%)	Total (N = XXX) n (%)
Completed Initial Extension	xx (xx.x)	xx (xx.x)	xx (xx.x)
Discontinued study during Initial Extension (primary reason for early termination)	xx (xx.x)	xx (xx.x)	xx (xx.x)
Death	xx (xx.x)	xx (xx.x)	xx (xx.x)
Adverse event(s)	xx (xx.x)	xx (xx.x)	xx (xx.x)
Withdrawal by subject	xx (xx.x)	xx (xx.x)	xx (xx.x)
Protocol violation(s)	xx (xx.x)	xx (xx.x)	xx (xx.x)
Lost to follow-up	xx (xx.x)	xx (xx.x)	xx (xx.x)
Physician decision	xx (xx.x)	xx (xx.x)	xx (xx.x)
Other	xx (xx.x)	xx (xx.x)	xx (xx.x)
Missing	xx (xx.x)	xx (xx.x)	xx (xx.x)

Note: Reasons for discontinuation are based on the End of Study CRF page.
Source: Listing xx.x.xx, Dataset: [NAME], Program: xxxxxx.sas, Output: T_16_1_1_2_1_XXXXX.rtf, Generated on: DDMONYYYY HH:MM
Page 1 of 1

Programming Note: Sort in order as on CRF. Include all discontinuation reasons on CRF (even if no subjects discontinued for that reason).

Table 16.1.1.2.2 Subject Disposition - ITT Overall Population

Programming Note: Repeat table for different population.

Table 16.1.2 Protocol Deviations - ITT Overall Population

**Category**<br>**Deviation (brief description)**	**GDNF/GDNF**<br>**(N = XXX)**<br>**n (%)**	**Placebo/GDNF**<br>**(N = XXX)**<br>**n (%)**	**Total**<br>**(N = XXX)**<br>**n (%)**
Subjects with at least one major protocol deviation	xx (xx.x)	xx (xx.x)	xx (xx.x)
[Category 1]	xx (xx.x)	xx (xx.x)	xx (xx.x)
[Deviation 1]	xx (xx.x)	xx (xx.x)	xx (xx.x)
[Deviation 2]	xx (xx.x)	xx (xx.x)	xx (xx.x)
[Category 2]	xx (xx.x)	xx (xx.x)	xx (xx.x)
[Deviation 1]	xx (xx.x)	xx (xx.x)	xx (xx.x)
[Deviation 2]	xx (xx.x)	xx (xx.x)	xx (xx.x)
…			
Subjects with at least one minor protocol deviation	xx (xx.x)	xx (xx.x)	xx (xx.x)
[Category 1]	xx (xx.x)	xx (xx.x)	xx (xx.x)
[Category 2]	xx (xx.x)	xx (xx.x)	xx (xx.x)
…			

Note: For each category and deviation, subjects are included only once, even if they experienced multiple events in that category or deviation.
Source: Listing xx.x.xx, Dataset: [NAME], Program: xxxxxx.sas, Output: T_16_1_2_XXXXX.rtf, Generated on: DDMONYYYY HH:MM
Page 1 of 1

Programming Note: Within category, sort should be by decreasing frequency of deviations in Total group.

Table 16.1.3.1 Demographic Characteristics from Study 2553 - ITT Primary Population

Variable	GDNF/GDNF (N = XXX)	Placebo/GDNF (N = XXX)	Total (N = XXX)
Age (years)			
N	xx	xx	xx
Mean (SD)	xx.x (xx.xx)	xx.x (xx.xx)	xx.x (xx.xx)
Median	xx.x	xx.x	xx.x
Min, Max	xx, xx	xx, xx	xx, xx
Age group [n (%)]			
< 65 years	xx (xx.x)	xx (xx.x)	xx (xx.x)
≥ 65 years	xx (xx.x)	xx (xx.x)	xx (xx.x)
Sex [n (%)]			
Female	xx (xx.x)	xx (xx.x)	xx (xx.x)
Male	xx (xx.x)	xx (xx.x)	xx (xx.x)
Race [n (%)]			
White	xx (xx.x)	xx (xx.x)	xx (xx.x)
Black	xx (xx.x)	xx (xx.x)	xx (xx.x)
Asian	xx (xx.x)	xx (xx.x)	xx (xx.x)
Native Hawaiian or other Pacific Islander	xx (xx.x)	xx (xx.x)	xx (xx.x)
American Indian or Alaska Native	xx (xx.x)	xx (xx.x)	xx (xx.x)
Other	xx (xx.x)	xx (xx.x)	xx (xx.x)
Missing	xx (xx.x)	xx (xx.x)	xx (xx.x)
Ethnicity [n (%)]			
Hispanic or Latino	xx (xx.x)	xx (xx.x)	xx (xx.x)
Not Hispanic or Latino	xx (xx.x)	xx (xx.x)	xx (xx.x)

[a]NART error score is the number of words pronounced incorrectly out of 50 total words.
Source: Listing xx.x.xx, Dataset: [NAME], Program: xxxxxx.sas, Output: T_16_1_3_1_XXXXX.rtf, Generated on: DDMONYYYY HH:MM
Page 1 of 2

Table 16.1.3.1 Demographic Characteristics from Study 2553 - ITT Primary Population (cont.)

Variable	GDNF/GDNF (N = XXX)	Placebo/GDNF (N = XXX)	Total (N = XXX)
Weight at baseline (kg)			
N	xx	xx	xx
Mean (SD)	xx.xx (xx.xxx)	xx.xx (xx.xxx)	xx.xx (xx.xxx)
Median	xx.x	xx.x	xx.x
Min, Max	xx.x, xx.x	xx.x, xx.x	xx.x, xx.x
Height at baseline (m)			
N	xx	xx	xx
Mean (SD)	xx.x (xx.xx)	xx.x (xx.xx)	xx.x (xx.xx)
Median	xx	xx	xx
Min, Max	xx, xx	xx, xx	xx, xx
BMI at baseline (kg/m^2)			
N	xx	xx	xx
Mean (SD)	xx.x (xx.xx)	xx.x (xx.xx)	xx.x (xx.xx)
Median	xx	xx	xx
Min, Max	xx, xx	xx, xx	xx, xx
NART error score (points)[a]			
n	xx	xx	xx
Mean (SD)	xx.x (xx.xx)	xx.x (xx.xx)	xx.x (xx.xx)
Median	xx	xx	xx
Min, Max	xx, xx	xx, xx	xx, xx

[a] NART error score is the number of words pronounced incorrectly out of 50 total words.
Source: Listing xx.x.xx, Dataset: [NAME], Program: xxxxxx.sas, Output: T_16_1_3_1_XXXXX.rtf, Generated on: DDMONYYYY HH:MM
Page 2 of 2

Programming Note: Add “Missing” if necessary to categorical variables.

Table 16.1.3.2 Demographic Characteristics from Study 2553 - ITT Overall Population

Programming Note: Repeat table for different population.

Table 16.1.4.1 Parkinson's Disease History at Screening from Study 2553 - ITT Primary Population

Variable	GDNF/GDNF (N = XXX)	Placebo/GDNF (N = XXX)	Total (N = XXX)
Duration since first PD symptom (years)			
N	xx	xx	xx
Mean (SD)	xx.xx (xx.xxx)	xx.xx (xx.xxx)	xx.xx (xx.xxx)
Median	xx.x	xx.x	xx.x
Min, Max	xx.x, xx.x	xx.x, xx.x	xx.x, xx.x
Duration since PD diagnosis (years)			
N	xx	xx	xx
Mean (SD)	xx.xx (xx.xxx)	xx.xx (xx.xxx)	xx.xx (xx.xxx)
Median	xx.x	xx.x	xx.x
Min, Max	xx.x, xx.x	xx.x, xx.x	xx.x, xx.x
Hoehn and Yahr stage in OFF state [n (%)]			
Stage 0: No signs of disease	xx (xx.x)	xx (xx.x)	xx (xx.x)
Stage 1: Unilateral symptoms only	xx (xx.x)	xx (xx.x)	xx (xx.x)
Stage 1.5: Unilateral and axial involvement	xx (xx.x)	xx (xx.x)	xx (xx.x)
Stage 2: Bilateral symptoms; no impairment of balance	xx (xx.x)	xx (xx.x)	xx (xx.x)
Stage 2.5: Mild bilateral disease with recovery on pull test	xx (xx.x)	xx (xx.x)	xx (xx.x)
Stage 3: Balance impairment; mild to moderate disease; physically independent	xx (xx.x)	xx (xx.x)	xx (xx.x)
Missing	xx (xx.x)	xx (xx.x)	xx (xx.x)
OFF state UPDRS motor score (part III)			
N	xx	xx	xx
Mean (SD)	xx.xx (xx.xxx)	xx.xx (xx.xxx)	xx.xx (xx.xxx)
Median	xx.x	xx.x	xx.x
Min, Max	xx.x, xx.x	xx.x, xx.x	xx.x, xx.x
ON state UPDRS motor score (part III)			
N	xx	xx	xx
Mean (SD)	xx.xx (xx.xxx)	xx.xx (xx.xxx)	xx.xx (xx.xxx)
Median	xx.x	xx.x	xx.x
Min, Max	xx.x, xx.x	xx.x, xx.x	xx.x, xx.x

[a] Percentage change in UPDRS motor score (part III) following a levodopa challenge.
Source: Listing xx.x.xx, Dataset: [NAME], Program: xxxxxx.sas, Output: T_16_1_4_1_XXXXX.rtf, Generated on: DDMONYYYY HH:MM
Page 1 of 2

Table 16.1.4.1 Parkinson's Disease History at Screening from Study 2553 - ITT Primary Population (cont.)

Variable	GDNF/GDNF (N = XXX)	Placebo/GDNF (N = XXX)	Total (N = XXX)
Total daily levodopa dose (mg)			
N	xx	xx	xx
Mean (SD)	xx.xx (xx.xxx)	xx.xx (xx.xxx)	xx.xx (xx.xxx)
Median	xx.x	xx.x	xx.x
Min, Max	xx.x, xx.x	xx.x, xx.x	xx.x, xx.x
Total daily levodopa equivalent dose (mg)			
N	xx	xx	xx
Mean (SD)	xx.xx (xx.xxx)	xx.xx (xx.xxx)	xx.xx (xx.xxx)
…	…	…	…
PD medications [n (%)]			
Levodopa preparations	xx (xx.x)	xx (xx.x)	xx (xx.x)
Dopamine agonists	xx (xx.x)	xx (xx.x)	xx (xx.x)
COMT inhibitors	xx (xx.x)	xx (xx.x)	xx (xx.x)
MAO-B inhibitors	xx (xx.x)	xx (xx.x)	xx (xx.x)
Other	xx (xx.x)	xx (xx.x)	xx (xx.x)
Responsiveness to levodopa[a] (%)			
N	xx	xx	xx
Mean (SD)	xx.xx (xx.xxx)	xx.xx (xx.xxx)	xx.xx (xx.xxx)
…	…	…	…
OFF time per day (hours)			
N	xx	xx	xx
Mean (SD)	xx.xx (xx.xxx)	xx.xx (xx.xxx)	xx.xx (xx.xxx)
…	…	…	…

[a] Percentage change in UPDRS motor score (part III) following a levodopa challenge.
Source: Listing xx.x.xx, Dataset: [NAME], Program: xxxxxx.sas, Output: T_16_1_4_1_XXXXX.rtf, Generated on: DDMONYYYY HH:MM
Page 2 of 2

Programming Note: Add "Missing" if necessary to categorical variables. See Section 5.0 definition of screening levodopa and levodopa equivalent dose.

Table 16.1.4.2 Parkinson's Disease History at Screening from Study 2553 - ITT Overall Population

Programming Note: Repeat table for different population.

Table 16.1.5.1 Concomitant Parkinson's Disease Medications by WHO Drug Dictionary ATC Class and Preferred Name - ITT Primary Population

**ATC Class**<br>**Preferred Name**	**GDNF/GDNF**<br>**(N = XXX)**<br>**n (%)**	**Placebo/GDNF**<br>**(N = XXX)**<br>**n (%)**	**Total**<br>**(N = XXX)**<br>**n (%)**
Subjects with at least one concomitant PD medication	xx (xx.x)	xx (xx.x)	xx (xx.x)
[ATC Class 1]	xx (xx.x)	xx (xx.x)	xx (xx.x)
[Preferred Name 1]	xx (xx.x)	xx (xx.x)	xx (xx.x)
[Preferred Name 2]	xx (xx.x)	xx (xx.x)	xx (xx.x)
[ATC Class 2]	xx (xx.x)	xx (xx.x)	xx (xx.x)
[Preferred Name 1]	xx (xx.x)	xx (xx.x)	xx (xx.x)
[Preferred Name 2]	xx (xx.x)	xx (xx.x)	xx (xx.x)
…			

Note: Concomitant medications are defined as any medications ongoing at the start of open-label study medication dosing or with a start date on or after the first open-label study medication dose date. Medications are coded using WHO Drug Dictionary Enhanced version 2016Mar01. For each ATC class and preferred name, subjects are included only once.
Source: Listing xx.x.xx, Dataset: [NAME], Program: xxxxxx.sas, Output: T_16_1_3_XXXXX.rtf, Generated on: DDMONYYYY HH:MM
Page x of y

Programming Note: Sort alphabetically for both ATC class and preferred name.

Table 16.1.5.2 Other Concomitant Medications by WHO Drug Dictionary ATC Class and Preferred Name - ITT Primary Population

ATC Class<br>Preferred Name	GDNF/GDNF<br>(N = XXX)<br>n (%)	Placebo/GDNF<br>(N = XXX)<br>n (%)	Total<br>(N = XXX)<br>n (%)
Subjects with at least one other concomitant medication	xx (xx.x)	xx (xx.x)	xx (xx.x)
[ATC Class 1]	xx (xx.x)	xx (xx.x)	xx (xx.x)
[Preferred Name 1]	xx (xx.x)	xx (xx.x)	xx (xx.x)
[Preferred Name 2]	xx (xx.x)	xx (xx.x)	xx (xx.x)
[ATC Class 2]	xx (xx.x)	xx (xx.x)	xx (xx.x)
[Preferred Name 1]	xx (xx.x)	xx (xx.x)	xx (xx.x)
[Preferred Name 2]	xx (xx.x)	xx (xx.x)	xx (xx.x)
…			

Note: Concomitant medications are defined as any medications ongoing at the start of open-label study medication dosing or with a start date on or after the first open-label study medication dose date. Medications are coded using WHO Drug Dictionary Enhanced version 2016Mar01. For each ATC class and preferred name, subjects are included only once.
Source: Listing xx.x.xx, Dataset: [NAME], Program: xxxxxx.sas, Output: T_16_1_3_XXXXX.rtf, Generated on: DDMONYYYY HH:MM
Page x of y

Programming Note: Sort alphabetically for both ATC class and preferred name.

16.2 Efficacy Data Tables

16.2.1 UPDRS Primary Efficacy Tables

Table 16.2.1.1 OFF State UPDRS Motor Score (Part III): Change and Percentage Change from Baseline to Week 80/e40 – MMRM, ITT Primary Population

	GDNF/GDNF (N = XXX)			Placebo/GDNF (N = XXX)		
**Visit<br>Statistic**	**Value**	**Change From Baseline**	**Percentage Change From Baseline**	**Value**	**Change From Baseline**	**Percentage Change From Baseline**
Week 0 (Baseline)						
N	xx			xx		
Mean (SD)	xx.xx (xx.xxx)			xx.xx (xx.xxx)		
Median	xx.x			xx.x		
Min, Max	xx.x, xx.x			xx.x, xx.x		
Week 40/e0						
N	xx	xx	xx	xx	xx	xx
Mean (SD)	xx.xx (xx.xxx)	xx.xx (xx.xxx)	xx.xx (xx.xxx)	xx.xx (xx.xxx)	xx.xx (xx.xxx)	xx.xx (xx.xxx)
Median	xx.x	xx.x	xx.x	xx.x	xx.x	xx.x
Min, Max	xx.x, xx.x	xx.x, xx.x	xx.x, xx.x	xx.x, xx.x	xx.x, xx.x	xx.x, xx.x
Week 80/e40						
N	xx	xx	xx	xx	xx	xx
Mean (SD)	xx.xx (xx.xxx)	xx.xx (xx.xxx)	xx.xx (xx.xxx)	xx.xx (xx.xxx)	xx.xx (xx.xxx)	xx.xx (xx.xxx)
Median	xx.x	xx.x	xx.x	xx.x	xx.x	xx.x
Min, Max	xx.x, xx.x	xx.x, xx.x	xx.x, xx.x	xx.x, xx.x	xx.x, xx.x	xx.x, xx.x
Least squares mean[a] (95% CI)		xx.x (xx.x, xx.x)	xx.x (xx.x, xx.x)		xx.x (xx.x, xx.x)	xx.x (xx.x, xx.x)
Least squares mean difference versus placebo[a] (95% CI)		xx.x (xx.x, xx.x)	xx.x (xx.x, xx.x)			
p-value[a]		0.xxxx	0.xxxx			

[a] Estimates are from a mixed-effect model with repeated measures (MMRM) with baseline UPDRS score as a covariate, treatment group and visit and treatment group*visit as fixed effects, and subject within treatment group as a random effect.
Note: Lower scores represent better functioning. End of study visit data are included in the appropriate visit week. For subject 45, items 22, 27, 28, 29 and 30 are excluded from the score.
Source: Listing xx.x.xx, Dataset: [NAME], Program: xxxxxx.sas, Output: xxxx.rtf, Generated on: DDMONYYYY HH:MM
Page x of y

Table 16.2.1.2 OFF State UPDRS Motor Score (Part III): Change and Percentage Change from Baseline to Week 80/e40 – MMRM, ITT Overall Population

Programming Note: Repeat Table 16.2.1.1 for different population.

16.2.2 UPDRS Secondary Efficacy Tables

Table 16.2.2.1 ON State UPDRS Motor Score (Part III): Change and Percentage Change from Baseline to Week 80/e40 – MMRM, ITT Overall Population

Programming Note: Repeat of Table 16.2.1.1, but for ON state parameters and ITT Overall Population.

Table 16.2.2.2.1 OFF State UPDRS ADL Score (Part II): Change and Percentage Change from Baseline to Week 80/e40 – MMRM, ITT Primary Population

Programming Note: Repeat of Table 16.2.1.1, but for OFF state ADL (Part II) score. Change footnote regarding subject 45 to: "Data for subject 45 are excluded from analysis."

Table 16.2.2.2.2 OFF State UPDRS ADL Score (Part II): Change and Percentage Change from Baseline to Week 80/e40 – MMRM, ITT Overall Population

Programming Note: Repeat of Table 16.2.2.2.1, but with different population.

Table 16.2.2.3 ON State UPDRS ADL Score (Part II): Change and Percentage Change from Baseline to Week 80/e40 – MMRM, ITT Overall Population

Programming Note: Repeat of Table 16.2.2.2.1, but for ON state ADL (Part II) score and with different population.

Table 16.2.2.4.1 OFF State UPDRS Total Score: Change and Percentage Change from Baseline to Week 80/e40 – MMRM, ITT Primary Population

Programming Note: Repeat of Table 16.2.1.1, but for OFF state total score. Change footnote regarding subject 45 to: "Data for subject 45 are excluded from analysis."

Table 16.2.2.4.2 OFF State UPDRS Total Score: Change and Percentage Change from Baseline to Week 80/e40 – MMRM, ITT Overall Population

Programming Note: Repeat of Table 16.2.2.4.1, but with different population,

Table 16.2.2.5 ON State UPDRS Total Score: Change and Percentage Change from Baseline to Week 80/e40 – MMRM, ITT Overall Population

Programming Note: Repeat of Table 16.2.2.4.1, but for ON state total score and different population."

Table 16.2.2.6.1 OFF State UPDRS Motor Score (Part III): Change from Baseline to Week 40/e0 for GDNF/GDNF Group Compared to Change from Baseline to Week 80/e40 for Placebo/GDNF Group – MMRM, ITT Overall Population

	GDNF/GDNF (N = XXX)		Placebo/GDNF (N = XXX)	
Visit<br>Statistic	Value	Change From Baseline	Value	Change From Baseline
Week 0 (Baseline)				
N	xx		xx	
Mean (SD)	xx.xx (xx.xxx)		xx.xx (xx.xxx)	
Median	xx.x		xx.x	
Min, Max	xx.x, xx.x		xx.x, xx.x	
Week 40/e0				
N	xx	xx		
Mean (SD)	xx.xx (xx.xxx)	xx.xx (xx.xxx)		
Median	xx.x	xx.x		
Min, Max	xx.x, xx.x	xx.x, xx.x		
Week 80/e40				
N			xx	xx
Mean (SD)			xx.xx (xx.xxx)	xx.xx (xx.xxx)
Median			xx.x	xx.x
Min, Max			xx.x, xx.x	xx.x, xx.x
Least squares mean[a] (95% CI)		xx.x (xx.x, xx.x)		xx.x (xx.x, xx.x)
Least squares mean difference versus placebo[a] (95% CI)		xx.x (xx.x, xx.x)		
p-value[a]		0.xxxx		

[a] Estimates are from a mixed-effect model with repeated measures (MMRM) with baseline UPDRS score as a covariate, treatment group and visit and treatment group*visit as fixed effects, and subject within treatment group as a random effect.
Note: Lower scores represent better functioning. End of study visit data are included in the appropriate visit week. For subject 45, items 22, 27, 28, 29 and 30 are excluded from the score.
Source: Listing xx.x.xx, Dataset: [NAME], Program: xxxxxx.sas, Output: xxxx.rtf, Generated on: DDMONYYYY HH:MM
Page x of y

Table 16.2.2.6.2 OFF State UPDRS ADL Score (Part II): Change from Baseline to Week 40/e0 for GDNF/GDNF Group Compared to Change from Baseline to Week 80/e40 for Placebo/GDNF Group – MMRM, ITT Overall Population

Programming Note: Repeat of Table 16.2.2.6.1. Change footnote regarding subject 45 to: "Data for subject 45 are excluded from analysis."

Table 16.2.2.6.3 OFF State UPDRS Total Score: Change from Baseline to Week 40/e0 for GDNF/GDNF Group Compared to Change from Baseline to Week 80/e40 for Placebo/GDNF Group – MMRM, ITT Overall Population

Programming Note: Repeat of Table 16.2.2.6.1. Change footnote regarding subject 45 to: "Data for subject 45 are excluded from analysis."

Table 16.2.2.7.1 OFF State UPDRS Motor Score (Part III): Change from Baseline to Week 80/e40 for GDNF/GDNF Group Compared to Change from Baseline to Week 40/e0 for Placebo/GDNF Group – MMRM, ITT Overall Population

Programming Note: Repeat Table 16.2.2.6.1 for different endpoint and population. Apart from baseline, present only Week 80/e40 for GDNF/GDNF and Week 40/e0 for Placebo/GDNF.

Table 16.2.2.7.2 OFF State UPDRS ADL Score (Part II): Change from Baseline to Week 80/e40 for GDNF/GDNF Group Compared to Change from Baseline to Week 40/e0 for Placebo/GDNF Group – MMRM, ITT Overall Population

Programming Note: Repeat of Table 16.2.2.6.1. Apart from baseline, present only Week 80/e40 for GDNF/GDNF and Week 40/e0 for Placebo/GDNF. Change footnote regarding subject 45 to: "Data for subject 45 are excluded from analysis."

Table 16.2.2.7.3 OFF State UPDRS Total Score: Change from Baseline to Week 80/e40 for GDNF/GDNF Group Compared to Change from Baseline to Week 40/e0 for Placebo/GDNF Group – MMRM, ITT Overall Population

Programming Note: Repeat of Table 16.2.2.6.1. Apart from baseline, present only Week 80/e40 for GDNF/GDNF and Week 40/e0 for Placebo/GDNF. Change footnote regarding subject 45 to: "Data for subject 45 are excluded from analysis."

16.2.3 PD DIARY SECONDARY EFFICACY TABLES

Table 16.2.3.1 Motor Fluctuation Diary Ratings by Visit: Change from Baseline – MMRM, ITT Primary Population

Parameter<br>Visit<br>Statistic	GDNF/GDNF (N = XXX)		Placebo/GDNF (N = XXX)	
	Value	Change From Baseline	Value	Change From Baseline
OFF time per day (hours)				
Week 0 (Baseline)				
n	xx		xx	
Mean (SD)	xx.xx (xx.xxx)		xx.xx (xx.xxx)	
Median	xx.x		xx.x	
Min, Max	xx.x, xx.x		xx.x, xx.x	
Week 40/e0				
n	xx	xx	xx	xx
Mean (SD)	xx.xx (xx.xxx)	xx.xx (xx.xxx)	xx.xx (xx.xxx)	xx.xx (xx.xxx)
Median	xx.x	xx.x	xx.x	xx.x
Min, Max	xx.x, xx.x	xx.x, xx.x	xx.x, xx.x	xx.x, xx.x
Week 80/e40				
n	xx	xx	xx	xx
Mean (SD)	xx.xx (xx.xxx)	xx.xx (xx.xxx)	xx.xx (xx.xxx)	xx.xx (xx.xxx)
Median	xx.x	xx.x	xx.x	xx.x
Min, Max	xx.x, xx.x	xx.x, xx.x	xx.x, xx.x	xx.x, xx.x
Least squares mean[a] (95% CI)		xx.x (xx.x, xx.x)		xx.x (xx.x, xx.x)
Least squares mean difference versus placebo[a] (95% CI)		xx.x (xx.x, xx.x)		
p-value[a]		0.xxxx		

[a] Estimates are from a mixed-effect model with repeated measures (MMRM) with baseline variable as a covariate, treatment group and visit and treatment group*visit as fixed effects, and subject within treatment group as a random effect.
Note: End of study visit data are included in the appropriate visit week.
Note: Total good-quality ON time per day is defined as the sum of ON time per day without dyskinesias + ON time per day with non-troublesome dyskinesias.

Source: Listing xx.x.xx, Dataset: [NAME], Program: xxxxxx.sas, Output: xxxx.rtf, Generated on: DDMONYYYY HH:MM
Page x of y

Programming Note: PD diary parameters are OFF time per day, total good-quality ON time per day (sum of ON time per day without dyskinesias + ON time per day with non-troublesome dyskinesias), ON time per day without dyskinesias, ON time per day with non-troublesome dyskinesias, and ON time per day with troublesome dyskinesias. Visits are baseline, Week 40/e0, and Week 80/e40. Statistics are for Week 80/e40 only.

Table 16.2.3.2 Motor Fluctuation Diary Ratings by Visit: Change from Baseline – MMRM, ITT Overall Population

Programming Note: Repeat above table for different population. Statistics are for Week 80/e40 only.

Table 16.2.3.3.1 Treatment Response at Week 40/e0 and Week 80/e40 - ITT Overall Population

Treatment Response Criteria	Visit	GDNF/GDNF (N = XXX) n (%)	Placebo/GDNF (N = XXX) n (%)	Total (N = XXX) n (%)
Decrease from baseline by >=10 points in OFF state UPDRS motor score (part III)	Week 40/e0	xx (xx.x)	xx (xx.x)	xx (xx.x)
	Week 80/e40	xx (xx.x)	xx (xx.x)	xx (xx.x)
Increase from baseline by >=1 hour in total good-quality ON time per day (ON without dyskinesias or ON with non-trouble-some dyskinesias)	Week 40/e0	xx (xx.x)	xx (xx.x)	xx (xx.x)
	Week 80/e40	xx (xx.x)	xx (xx.x)	xx (xx.x)
Both of the above criteria	Week 40/e0	xx (xx.x)	xx (xx.x)	xx (xx.x)
	Week 80/e40	xx (xx.x)	xx (xx.x)	xx (xx.x)

Source: Listing xx.x.xx, Dataset: [NAME], Program: xxxxxx.sas, Output: T_16_X_XXXXX.rtf, Generated on: DDMONYYYY HH:MM
Page x of y

Table 16.2.3.3.2 UPDRS Responder at Week 80/e40 Compared to Week 40/e0 - ITT Overall Population

	GDNF/GDNF (N = XXX)			Placebo/GDNF (N = XXX)		
	UPDRS Responder at Week 80/e40			UPDRS Responder at Week 80/e40		
**UPDRS Responder at Week 40/e0**	**Yes n (%)**	**No n (%)**	**Missing n (%)**	**Yes n (%)**	**No n (%)**	**Missing n (%)**
Yes	xxx (xx.x)	xxx (xx.x)	xxx (xx.x)	xxx (xx.x)	xxx (xx.x)	xxx (xx.x)
No	xxx (xx.x)	xxx (xx.x)	xxx (xx.x)	xxx (xx.x)	xxx (xx.x)	xxx (xx.x)
Missing	xxx (xx.x)	xxx (xx.x)	xxx (xx.x)	xxx (xx.x)	xxx (xx.x)	xxx (xx.x)

Note: UPDRS responder is defined as a decrease from baseline by >=10 points in OFF state UPDRS motor score (part III).
Source: Listing xx.x.xx, Dataset: [NAME], Program: xxxxxx.sas, Output: xxxx.rtf, Generated on: DDMONYYYY HH:MM
Page x of y

16.2.4 Supplementary Efficacy Tables

Table 16.2.4.1 OFF and ON State Timed Walking Test by Visit: Change from Baseline – MMRM, ITT Overall Population

Parameter<br>Visit<br>Statistic	GDNF/GDNF (N = XXX)<br>Value	GDNF/GDNF (N = XXX)<br>Change From Baseline	Placebo/GDNF (N = XXX)<br>Value	Placebo/GDNF (N = XXX)<br>Change From Baseline
OFF state timed walking test (seconds)				
Week 0 (Baseline)				
n	xx		xx	
Mean (SD)	xx.xx (xx.xxx)		xx.xx (xx.xxx)	
Median	xx.x		xx.x	
Min, Max	xx.x, xx.x		xx.x, xx.x	
Week 40/e0				
n	xx	xx	xx	xx
Mean (SD)	xx.xx (xx.xxx)	xx.xx (xx.xxx)	xx.xx (xx.xxx)	xx.xx (xx.xxx)
Median	xx.x	xx.x	xx.x	xx.x
Min, Max	xx.x, xx.x	xx.x, xx.x	xx.x, xx.x	xx.x, xx.x
Week 80/e40				
n	xx	xx	xx	xx
Mean (SD)	xx.xx (xx.xxx)	xx.xx (xx.xxx)	xx.xx (xx.xxx)	xx.xx (xx.xxx)
Median	xx.x	xx.x	xx.x	xx.x
Min, Max	xx.x, xx.x	xx.x, xx.x	xx.x, xx.x	xx.x, xx.x
Least squares mean[a] (95% CI)		xx.x (xx.x, xx.x)		xx.x (xx.x, xx.x)
Least squares mean difference versus placebo[a] (95% CI)		xx.x (xx.x, xx.x)		
p-value[a]		0.xxxx		

[a] Estimates are from a mixed-effect model with repeated measures (MMRM) with baseline time as a covariate, treatment group and visit and treatment group*visit as fixed effects, and subject within treatment group as a random effect.
Note: Shorter times represent better function. Two trials per state per visit are averaged for analysis. If only the first trial is completed for a particular state, then the second trial result will be imputed with the worst non-missing second trial result from all subjects for that particular state during the study. If both trials are missing, then the endpoint is not reported for that visit. End of study visit data are included in the appropriate visit week. Data for subject 45 are excluded from analysis.
Source: Listing xx.x.xx, Dataset: [NAME], Program: xxxxxx.sas, Output: xxxx.rtf, Generated on: DDMONYYYY HH:MM
Page x of y

Programming Note: Timed walking test parameters are OFF state timed walking test and ON state timed walking test (seconds). Visits are baseline, Week 40/e0, and Week 80/e40. Statistics are for Week 80/e40 only.

Table 16.2.4.2 OFF and ON State Timed Tapping Test by Visit: Change from Baseline – MMRM, ITT Overall Population

Programming Note: Repeat Table 16.2.4.1, but for time tapping test by visit. Timed tapping test parameters are OFF state timed tapping test, and ON state timed tapping test . Visits are baseline, Week 40/e0, and Week 80/e40*. Statistics are for Week 80/e40 only. Replace footnote text with "…(MMRM) with baseline number of taps as a covariate…". Replace note with: "More taps represent better function. Four trials per state for each visit are averaged for analysis. If any trial is not completed for a particular state, then the trial result will be imputed with the worst non-missing applicable trial result (for example, first left hand trial) from all subjects for that particular state during the study. If all four trials are missing, then the endpoint is not reported for that visit. End of study visit data are included in the appropriate visit week."*

Table 16.2.4.3 NMSS Score by Visit: Change from Baseline – MMRM, ITT Overall Population

Parameter<br>Visit<br>Statistic	GDNF/GDNF (N = XXX) Value	GDNF/GDNF (N = XXX) Change From Baseline	Placebo/GDNF (N = XXX) Value	Placebo/GDNF (N = XXX) Change From Baseline
NMSS Total Score				
Screening (Baseline)				
n	xx		xx	
Mean (SD)	xx.xx (xx.xxx)		xx.xx (xx.xxx)	
Median	xx.x		xx.x	
Min, Max	xx.x, xx.x		xx.x, xx.x	
Week 40/e0				
…				
Week 80/e40				
n	xx	xx	xx	xx
Mean (SD)	xx.xx (xx.xxx)	xx.xx (xx.xxx)	xx.xx (xx.xxx)	xx.xx (xx.xxx)
Median	xx.x	xx.x	xx.x	xx.x
Min, Max	xx.x, xx.x	xx.x, xx.x	xx.x, xx.x	xx.x, xx.x
Least squares mean[a] (95% CI)		xx.x (xx.x, xx.x)		xx.x (xx.x, xx.x)
Least squares mean difference versus placebo[a] (95% CI)		xx.x (xx.x, xx.x)		
p-value[a]		0.xxxx		

[a] Estimates are from a mixed-effect model with repeated measures (MMRM) with baseline score as a covariate, treatment group and visit and treatment group*visit as fixed effects, and subject within treatment group as a random effect.
Note: The NMSS is a 30-item interview-based scale that rates non-motor symptoms that occurred in the preceding month in 9 domains. Each item is rated from 0 (none) to 3 (severe) for severity and from 1 (rarely) to 4 (very frequent) for frequency. The maximum score for an individual item is 12. The higher the score, the worse the subject's condition. The maximum NMSS total score is 360. Missing individual item scores are imputed using LOCF. End of study visit data are included in the appropriate visit week. Data for subject 45 are excluded from analysis.
Source: Listing xx.x.xx, Dataset: [NAME], Program: xxxxxx.sas, Output: xxxx.rtf, Generated on: DDMONYYYY HH:MM
Page x of y

Programming Note: NMSS parameters are Cardiovascular including falls domain, Sleep/fatigue domain, Mood/cognition domain, Perceptual problems/hallucinations domain; Attention/memory domain, Gastrointestinal tract domain, Urinary domain, Sexual function domain, Miscellaneous domain, and NMSS total score. Visits are baseline, Week 40/e0, and Week 80/e40. Statistics are for Week 80/e40 only.

Table 16.2.4.4 PDQ-39 Score: Change from Baseline to Week 80/e40 –ANCOVA, ITT Overall Population

Parameter<br>Visit<br>Statistic	GDNF/GDNF (N = XXX) Value	GDNF/GDNF (N = XXX) Change From Baseline	Placebo/GDNF (N = XXX) Value	Placebo/GDNF (N = XXX) Change From Baseline
Single Index (Total) PDQ-39 Score				
Screening (Baseline)				
n	xx		xx	
Mean (SD)	xx.xx (xx.xxx)		xx.xx (xx.xxx)	
Median	xx.x		xx.x	
Min, Max	xx.x, xx.x		xx.x, xx.x	
Week 40/e0				
n	xx	xx	xx	xx
Mean (SD)	xx.xx (xx.xxx)	xx.xx (xx.xxx)	xx.xx (xx.xxx)	xx.xx (xx.xxx)
Median	xx.x	xx.x	xx.x	xx.x
Min, Max	xx.x, xx.x	xx.x, xx.x	xx.x, xx.x	xx.x, xx.x
Week 80/e40				
n	xx	xx	xx	xx
Mean (SD)	xx.xx (xx.xxx)	xx.xx (xx.xxx)	xx.xx (xx.xxx)	xx.xx (xx.xxx)
Median	xx.x	xx.x	xx.x	xx.x
Min, Max	xx.x, xx.x	xx.x, xx.x	xx.x, xx.x	xx.x, xx.x
Mean difference versus placebo[a]				
(95% CI)		xx.x (xx.x, xx.x)		
p-value[a]		0.xxxx		

[a] Estimates are from an analysis of covariance (ANCOVA) model with baseline score as a covariate and treatment group as a factor.
Note: The PDQ-39 is a self-administered 39-item PD-specific scale that rates symptoms that occurred in the preceding month in 8 dimensions. Each item is rated from 0 (never) to 4 (always) for frequency. The score for each dimension is the average of responses in the dimension, weighted by the number of questions and multiplied by 100. PDQ-39 dimension and total scores range from 0 to 100 (0 = no problem, 100 = problem as worse as possible). The higher the score, the worse the subject's condition. Only subjects with a Week 40 value are included in the observed data analysis. If an individual question response is missing, then that dimension score and the single index (total) score are also missing. Data for subject 45 are excluded from analysis.
Source: Listing xx.x.xx, Dataset: [NAME], Program: xxxxxx.sas, Output: xxxx.rtf, Generated on: DDMONYYYY HH:MM
Page x of y

Programming Note: PDQ-39 parameters are Mobility dimension, ADL dimension, Emotional well-being dimension, Stigma dimension, Social support dimension, Cognitions dimension, Communication dimension, Bodily discomfort dimension, and Single index (total) PDQ-39 score. Visits are baseline, Week 40/e0, and Week 80/e40.

Table 16.2.4.5.1 EQ-5D Questionnaire: Baseline, Week 40/e0, and Week 80/e40 – Observed Data, ITT Overall Population

**EQ-5D Dimension**<br>**Visit**<br>**Response Level**	**GDNF/GDNF**<br>**(N = XXX)**<br>**n (%)**	**Placebo/GDNF**<br>**(N = XXX)**<br>**n (%)**
Mobility		
Screening (Baseline)		
No problem	xx (xx.x)	xx (xx.x)
Moderate problem	xx (xx.x)	xx (xx.x)
Severe problem	xx (xx.x)	xx (xx.x)
Week 40/e0		
No problem	xx (xx.x)	xx (xx.x)
Moderate problem	xx (xx.x)	xx (xx.x)
Severe problem	xx (xx.x)	xx (xx.x)
Week 80/e40		
No problem	xx (xx.x)	xx (xx.x)
Moderate problem	xx (xx.x)	xx (xx.x)
Severe problem	xx (xx.x)	xx (xx.x)
Self-care		
Screening (Baseline)		
No problem	xx (xx.x)	xx (xx.x)
Moderate problem	xx (xx.x)	xx (xx.x)
Severe problem	xx (xx.x)	xx (xx.x)
…		

Note: The EQ-5D is a self-administered 5-item scale with three possible answers for each item (1=no problem, 2=moderate problem, 3=severe problem), and a visual analog scale ranging from 0 to 100, where 0 indicates worst health and 100 indicates best health. Only subjects with a Week 40 value are included in the observed data analysis. Data for subject 45 are excluded from analysis.
Source: Listing xx.x.xx, Dataset: [NAME], Program: xxxxxx.sas, Output: T_16_2_4_5_1_XXXXX.rtf, Generated on: DDMONYYYY HH:MM
Page 1 of 1

Programming Note: Sort in order as on CRF. Continue for Self-care, Usual Activities, Pain / Discomfort, and Anxiety / Depression. Visits are baseline, Week 40/e0, and Week 80/e40. EQ-5D parameters are the frequency counts and percentages of subjects with the different answers to the 5 questions (categorical) and the visual analog scale score (continuous). Visual analog scale data is in the next table.

Table 16.2.4.5.2 EQ-5D Visual Analog Scale: Change from Baseline to Week 80/e40 – ANCOVA, ITT Overall Population

Programming Note: Repeat ANCOVA table for EQ-5D. Report observed data for baseline, Week 40/e0, Week 80/e40, and change from baseline summary statistics for visual analog scale (with mean difference, 95% CI, and p-value). Visits are baseline, Week 40/e0 and Week 80/e40. Add note: "The EQ-5D is a self-administered 5-item scale with three possible answers for each item (1=no problem, 2=moderate problem, 3=severe problem), and a visual analog scale ranging from 0 to 100, where 0 indicates worst health and 100 indicates best health. Only subjects with a Week 80/e40 value are included in the analysis. Data for subject 45 are excluded from analysis" EQ-5D parameters are the frequency counts and percentages of subjects with the different answers to the 5 questions (categorical) and the visual analog scale score (continuous).

Table 16.2.4.6 SNAQ Score: Change from Baseline to Week 80/e40 –ANCOVA, ITT Overall Population

Programming Note: Repeat ANCOVA table for SNAQ. SNAQ has one total SNAQ score. Visits are baseline, Week 40/e0 and Week 80/e40. Add note: "The SNAQ is a self-administered 4-question instrument with total scores ranging from 4 to 20 (4=poor appetite, 20=good appetite). Only subjects with a Week 80/e40 value are included in the analysis. If an individual question is not answered, then the total score is considered missing."

Table 16.2.4.7 Total Daily Levodopa Dose (mg): Change From Baseline to Week 80/e40 –ANCOVA, ITT Overall Population

	GDNF/GDNF (N = XXX)		Placebo/GDNF (N = XXX)	
**Visit Statistic**	**Value**	**Change From Baseline**	**Value**	**Change From Baseline**
Week 0 (Baseline)				
n	xx		xx	
Mean (SD)	xx.xx (xx.xxx)		xx.xx (xx.xxx)	
Median	xx.x		xx.x	
Min, Max	xx.x, xx.x		xx.x, xx.x	
Week 40/e0				
n	xx	xx	xx	xx
Mean (SD)	xx.xx (xx.xxx)	xx.xx (xx.xxx)	xx.xx (xx.xxx)	xx.xx (xx.xxx)
Median	xx.x	xx.x	xx.x	xx.x
Min, Max	xx.x, xx.x	xx.x, xx.x	xx.x, xx.x	xx.x, xx.x
Week 80/e40				
n	xx	xx	xx	xx
Mean (SD)	xx.xx (xx.xxx)	xx.xx (xx.xxx)	xx.xx (xx.xxx)	xx.xx (xx.xxx)
Median	xx.x	xx.x	xx.x	xx.x
Min, Max	xx.x, xx.x	xx.x, xx.x	xx.x, xx.x	xx.x, xx.x
Mean difference versus placebo[a] (95% CI)		xx.x (xx.x, xx.x)		
p-value[a]		0.xxxx		

[a] Estimates are from an analysis of covariance (ANCOVA) model with baseline levodopa dose as a covariate and treatment group as a factor.
Note: Only subjects with a Week 80/e40 levodopa value are included in the analysis.
Source: Listing xx.x.xx, Dataset: [NAME], Program: xxxxxx.sas, Output: xxxx.rtf, Generated on: DDMONYYYY HH:MM
Page x of y

Programming Note: See Section 5.0 for definition of levodopa dose.

Table 16.2.4.8 Total Daily Levodopa Equivalent Dose (mg): Change From Baseline to Week 80/e40 –ANCOVA, ITT Overall Population

	GDNF/GDNF (N = XXX)		Placebo/GDNF (N = XXX)	
**Visit**<br>**Statistic**	**Value**	**Change From Baseline**	**Value**	**Change From Baseline**
Week 0 (Baseline)				
n	xx		xx	
Mean (SD)	xx.xx (xx.xxx)		xx.xx (xx.xxx)	
Median	xx.x		xx.x	
Min, Max	xx.x, xx.x		xx.x, xx.x	
Week 40/e0				
n	xx	xx	xx	xx
Mean (SD)	xx.xx (xx.xxx)	xx.xx (xx.xxx)	xx.xx (xx.xxx)	xx.xx (xx.xxx)
Median	xx.x	xx.x	xx.x	xx.x
Min, Max	xx.x, xx.x	xx.x, xx.x	xx.x, xx.x	xx.x, xx.x
Week 80/e40				
n	xx	xx	xx	Xx
Mean (SD)	xx.xx (xx.xxx)	xx.xx (xx.xxx)	xx.xx (xx.xxx)	xx.xx (xx.xxx)
Median	xx.x	xx.x	xx.x	xx.x
Min, Max	xx.x, xx.x	xx.x, xx.x	xx.x, xx.x	xx.x, xx.x
Mean difference versus placebo[a]				
(95% CI)		xx.x (xx.x, xx.x)		
p-value[a]		0.xxxx		

[a] Estimates are from an analysis of covariance (ANCOVA) model with baseline levodopa equivalent dose as a covariate and treatment group as a factor.
Note: Only subjects with a Week 80/e40 levodopa equivalent value are included in the analysis.
Source: Listing xx.x.xx, Dataset: [NAME], Program: xxxxxx.sas, Output: xxxx.rtf, Generated on: DDMONYYYY HH:MM
Page x of y

Programming Note: See Section 5.0 for definition of levodopa equivalent dose.

16.3 IMAGING TABLES

Table 16.3.1 Volume of Distribution of Infusate as Determined by Contrast-Enhanced T1-Weighted MRI: Change from Baseline to Week 80/e40 –ANCOVA, ITT Primary Population

Programming Note: Repeat ANCOVA Table 16.2.4.4. Visits are Healing phase (Baseline) (last test infusion at the end of the healing phase), Week 40/e0, and Week 80/e40. The post-infusion time point at each visit is used. Parameter is volume of distribution (in mL), separately for left and right hemispheres, as determined by contrast-enhanced T1-weighted MRI. Table will have 2 pages, one for left and right hemisphere; please subtitle each page clearly.

Table 16.3.2.1 Volume of Interest Coverage as Determined by Contrast-Enhanced T1-Weighted MRI: Change from Baseline to Week 80/e40 – ANCOVA, ITT Primary Population

Programming Note: Repeat observed data ANCOVA Table 16.2.4.4. Visits are Healing phase (Baseline) (last test infusion at the end of the healing phase), Week 40/e0, and Week 80/e40. The post-infusion time point at each visit is used. Parameter is VOI coverage as a percentage of total VOI, separately for left putamen and right putamen and for both putamina combined. Table will have 3 pages, one for each of left and right putamen and one for both putamina combined; please subtitle each page clearly.

Table 16.3.2.2 Total Putamenal Coverage as Determined by Contrast-Enhanced T1-Weighted MRI: Change from Baseline to Week 80/e40 – ANCOVA, ITT Primary Population

Programming Note: Repeat observed data ANCOVA Table 16.2.4.4. Visits are Randomization (Baseline), Week 40/e0, and Week 80/e40. The post-infusion time point at each visit is used. Parameter is total putamenal coverage as a percentage of total putamenal volume, separately for left putamen and right putamen and for both putamina combined. Table will have 3 pages, one for each of left and right putamen and one for both putamina combined; please subtitle each page clearly.

Table 16.3.3.1 Correlation Analyses of Percentage Change from Baseline to Week 80/e40 in OFF State UPDRS Motor Score (Part III) to Volume of Interest Coverage and Total Putamenal Coverage at Baseline as Determined by Contrast-Enhanced T1-Weighted MRI – ITT Primary Population

**Parameters**	**GDNF/GDNF (N = XXX)**<br>**n**<br>**Spearman Rank Correlation (95% CI)**<br>**p-value**	**Placebo/GDNF (N = XXX)**<br>**n**<br>**Spearman Rank Correlation (95% CI)**<br>**p-value**
Percentage change from baseline to Week 80/e40 in OFF state UPDRS motor score (part III)<br>versus<br>VOI coverage at baseline, both putamina combined	xx<br>0.xxx (0.xxx, 0.xxx)<br>0.xxxx	xx<br>0.xxx (0.xxx, 0.xxx)<br>0.xxxx
Percentage change from baseline to Week 80/e40 in OFF state UPDRS motor score (part III)<br>versus<br>Total putamenal coverage at baseline, both putamina combined	xx<br>0.xxx (0.xxx, 0.xxx)<br>0.xxxx	xx<br>0.xxx (0.xxx, 0.xxx)<br>0.xxxx

Note: For subject 45, items 22, 27, 28, 29 and 30 are excluded from the UPDRS score.
Source: Listing xx.x.xx, Dataset: [NAME], Program: xxxxxx.sas, Output: xxxx.rtf, Generated on: DDMONYYYY HH:MM
Page x of y

Table 16.3.3.2 Correlation Analysis of Percentage Change from Baseline to Week 80/e40 in OFF State UPDRS Motor Score (Part III) to Change from Baseline to Week 40/e0 in ^{18}F-DOPA Uptake as Determined by PET Scan – ITT Primary Population

Programming Note: Repeat above Table 16.3.3.1 for parameters "Percentage change from baseline to Week 80/e40 in OFF state UPDRS Motor score (Part III)" and "Change from Baseline to Week 40/e0 in ^{18}F-DOPA Uptake."

Table 16.3.3.3 Correlation Analysis of Percentage Change from Baseline to Week 80/e40 in OFF State UPDRS Motor Score (Part III) to Change from Baseline to Week 40/e0 in ^{18}F-DOPA Uptake as Determined by PET Scan – ITT Overall Population

Programming Note: Repeat Table 16.3.3.2 for different population.

16.4 Safety Data Tables

16.4.1 Exposure Data Tables

Table 16.4.1.1 Exposure to Study Medication - Safety Overall Population

Extension Part<br>Variable	GDNF/GDNF<br>(N = XXX)	Placebo/GDNF<br>(N = XXX)
Initial Extension		
Number of infusions of study medication		
n	xx	Xx
Mean (SD)	xx.x (xx.xx)	xx.x (xx.xx)
Median	xx.x	xx.x
Min, Max	xx, xx	xx, xx
Total GDNF exposure[a] (mg)		
n	xx	Xx
Mean (SD)	xx.x (xx.xx)	xx.x (xx.xx)
Median	xx.x	xx.x
Min, Max	xx, xx	xx, xx
Pilot Extension		
Number of infusions of study medication		
n	xx	Xx
Mean (SD)	xx.x (xx.xx)	xx.x (xx.xx)
Median	xx.x	xx.x
Min, Max	xx, xx	xx, xx
Total GDNF exposure[a] (mg)		
n	xx	Xx
Mean (SD)	xx.x (xx.xx)	xx.x (xx.xx)
Median	xx.x	xx.x
Min, Max	xx, xx	xx, xx

[a] Total exposure in mg assumes the entire infusion was completed at each administration.
Source: Listing xx.x.xx, Dataset: [NAME], Program: xxxxxx.sas, Output: T_16_4_1_1_XXXXX.rtf, Generated on: DDMONYYYY HH:MM
Page x of y

Table 16.4.1.1 Exposure to Study Medication - Safety Overall Population (cont.)

Extension Part<br>Variable	GDNF/GDNF<br>(N = XXX)	Placebo/GDNF<br>(N = XXX)
Supplemental Extension		
Number of infusions of study medication		
n	xx	Xx
Mean (SD)	xx.x (xx.xx)	xx.x (xx.xx)
Median	xx.x	xx.x
Min, Max	xx, xx	xx, xx
Total GDNF exposure[a] (mg)		
n	xx	Xx
Mean (SD)	xx.x (xx.xx)	xx.x (xx.xx)
Median	xx.x	xx.x
Min, Max	xx, xx	xx, xx
Overall		
Number of infusions of study medication		
n	xx	Xx
Mean (SD)	xx.x (xx.xx)	xx.x (xx.xx)
Median	xx.x	xx.x
Min, Max	xx, xx	xx, xx
Total GDNF exposure[a] (mg)		
n	xx	Xx
Mean (SD)	xx.x (xx.xx)	xx.x (xx.xx)
Median	xx.x	xx.x
Min, Max	xx, xx	xx, xx

[a] Total exposure in mg assumes the entire infusion was completed at each administration.
Source: Listing xx.x.xx, Dataset: [NAME], Program: xxxxxx.sas, Output: T_16_4_1_1_XXXXX.rtf, Generated on: DDMONYYYY HH:MM
Page x of y

Table 16.4.1.2 Study Medication Infusion Details by Visit - Safety Overall Population

Visit<br>Variable	GDNF/GDNF<br>(N = XXX)	Placebo/GDNF<br>(N = XXX)
Week e0		
Duration of infusion[a] (minutes)		
n	xx	Xx
Mean (SD)	xx.x (xx.xx)	xx.x (xx.xx)
Median	xx.x	xx.x
Min, Max	xx, xx	xx, xx
Any infusion interruption/early termination [n (%)]		
No	xx (xx.x)	xx (xx.x)
Yes	xx (xx.x)	xx (xx.x)
Missing	xx (xx.x)	xx (xx.x)
Week 44/ e4		
…		

[a] Duration of infusion of study medication in minutes is calculated as (infusion end time – infusion start time + 1). Interruptions are not subtracted.
Source: Listing xx.x.xx, Dataset: [NAME], Program: xxxxxx.sas, Output: T_16_4_1_2_XXXXX.rtf, Generated on: DDMONYYYY HH:MM
Page x of y

Programming Note: Continue for all infusion visits, including the Pilot Extension and Supplemental Extension.

16.4.2 Adverse Event Tables

Table 16.4.2.1 Overall Summary of Adverse Events - Safety Overall Population

Adverse Event Category	GDNF/GDNF (N = XXX) n (%)	Placebo/GDNF (N = XXX) n (%)	Total (N = XXX) n (%)
Any TEAE	xxx (xx.x)	xxx (xx.x)	xxx (xx.x)
Any severe TEAE	xxx (xx.x)	xxx (xx.x)	xxx (xx.x)
Any serious TEAE	xxx (xx.x)	xxx (xx.x)	xxx (xx.x)
Any TEAE leading to permanent discontinuation of study medication	xxx (xx.x)	xxx (xx.x)	xxx (xx.x)
Any study medication-related TEAE	xxx (xx.x)	xxx (xx.x)	xxx (xx.x)
Any serious study medication-related TEAE	xxx (xx.x)	xxx (xx.x)	xxx (xx.x)
Any device-related TEAE	xxx (xx.x)	xxx (xx.x)	xxx (xx.x)
Any serious device-related TEAE	xxx (xx.x)	xxx (xx.x)	xxx (xx.x)

Note: For each category, subjects are included only once, even if they experienced multiple events in that category. All AEs reported during the study period are considered TEAEs. TEAEs that are present or ongoing at the beginning of Study 2797 (date of consent or date of last visit in Study 2553, if consent was obtained earlier) are considered pre-existing, if the event term, severity, and date and time of onset in Study 2797 are identical to the corresponding information given for the TEAE in Study 2553. If any of these conditions is not met, the TEAE is considered new or worsening. Only new or worsening TEAEs are summarized.
Source: Listing xx.x.xx, Dataset: [NAME], Program: xxxxxx.sas, Output: xxxx.rtf, Generated on: DDMONYYYY HH:MM
Page 1 of 1

Table 16.4.2.2 Treatment-Emergent Adverse Events by System Organ Class and Preferred Term - Safety Overall Population

**System Organ Class**<br>**Preferred Term**	**GDNF/GDNF**<br>**(N = XXX)**<br>**n (%)**	**Placebo/GDNF**<br>**(N = XXX)**<br>**n (%)**	**Total**<br>**(N = XXX)**<br>**n (%)**
Subjects with at least one TEAE	xx (xx.x)	xx (xx.x)	xx (xx.x)
[System Organ Class 1]	xx (xx.x)	xx (xx.x)	xx (xx.x)
[Preferred Term 1]	xx (xx.x)	xx (xx.x)	xx (xx.x)
[Preferred Term 2]	xx (xx.x)	xx (xx.x)	xx (xx.x)
[System Organ Class 2]	xx (xx.x)	xx (xx.x)	xx (xx.x)
[Preferred Term 1]	xx (xx.x)	xx (xx.x)	xx (xx.x)
[Preferred Term 2]	xx (xx.x)	xx (xx.x)	xx (xx.x)
…			

Note: Adverse events are coded using MedDRA version 19.0. All AEs reported during the study period are considered TEAEs. TEAEs that are present or ongoing at the beginning of Study 2797 (date of consent or date of last visit in Study 2553, if consent was obtained earlier) are considered pre-existing, if the event term, severity, and date and time of onset in Study 2797 are identical to the corresponding information given for the TEAE in Study 2553. If any of these conditions is not met, the TEAE is considered new or worsening. Only new or worsening TEAEs are summarized. For each system organ class and preferred term, subjects are included only once, even if they experienced multiple events in that system organ class or preferred term.
Source: Listing xx.x.xx, Dataset: [NAME], Program: xxxxxx.sas, Output: T_16_4_2_2_XXXXX.rtf, Generated on: DDMONYYYY HH:MM
Page x of y

Programming Note: Sort alphabetically by SOC and then alphabetically for PT.

Table 16.4.2.3 Treatment-Emergent Adverse Events Experienced by at Least 3 Subjects Overallby Preferred Term - Safety Overall Population

	GDNF/GDNF (N = XXX)		Placebo/GDNF (N = XXX)		Total (N=XXX)	
**Preferred Term**	**n Subjects (%)**	**n Events**	**n Subjects (%)**	**n Events**	**n Subjects (%)**	**n Events**
[Preferred Term 1]	xx (xx.x)	xxx	xx (xx.x)	Xxx	xx (xx.x)	Xxx
[Preferred Term 2]	xx (xx.x)	xxx	xx (xx.x)	Xxx	xx (xx.x)	Xxx
[Preferred Term 3]	xx (xx.x)	xxx	xx (xx.x)	Xxx	xx (xx.x)	Xxx
[Preferred Term 4]	xx (xx.x)	xxx	xx (xx.x)	Xxx	xx (xx.x)	Xxx
[Preferred Term 5]	xx (xx.x)	xxx	xx (xx.x)	Xxx	xx (xx.x)	Xxx
[Preferred Term 6]	xx (xx.x)	xxx	xx (xx.x)	Xxx	xx (xx.x)	Xxx
[Preferred Term 7]	xx (xx.x)	xxx	xx (xx.x)	Xxx	xx (xx.x)	Xxx
[Preferred Term 8]	xx (xx.x)	xxx	xx (xx.x)	Xxx	xx (xx.x)	Xxx
…						

Note: Adverse events are coded using MedDRA version 19.0. All AEs reported during the study period are considered TEAEs. TEAEs that are present or ongoing at the beginning of Study 2797 (date of consent or date of last visit in Study 2553, if consent was obtained earlier) are considered pre-existing, if the event term, severity, and date and time of onset in Study 2797 are identical to the corresponding information given for the TEAE in Study 2553. If any of these conditions is not met, the TEAE is considered new or worsening. Only new or worsening TEAEs are summarized. For each preferred term, subjects are included only once, even if they experienced multiple events in that preferred term.
Source: Listing xx.x.xx, Dataset: [NAME], Program: xxxxxx.sas, Output: T_16_4_2_3_XXXXX.rtf, Generated on: DDMONYYYY HH:MM
Page 1 of 1

Programming Note: Sort by descending frequency of number of subjects in the total column.

Table 16.4.2.4 Treatment-Emergent Adverse Events by System Organ Class, Preferred Term, and Maximum Severity - Safety Overall Population

	GDNF/GDNF (N = XXX)				Placebo/GDNF (N = XXX)			
**System Organ Class<br>Preferred Term**	**Mild<br>n (%)**	**Moderate<br>n (%)**	**Severe<br>n (%)**	**Total<br>n (%)**	**Mild<br>n (%)**	**Moderate<br>n (%)**	**Severe<br>n (%)**	**Total<br>n (%)**
Subjects with at least one TEAE	xxx (xx.x)	xxx (xx.x)	xxx (xx.x)	xxx (xx.x)	xxx (xx.x)	xxx (xx.x)	xxx (xx.x)	xxx (xx.x)
[System Organ Class 1]	xxx (xx.x)	xxx (xx.x)	xxx (xx.x)	xxx (xx.x)	xxx (xx.x)	xxx (xx.x)	xxx (xx.x)	xxx (xx.x)
[Preferred Term 1]]	xxx (xx.x)	xxx (xx.x)	xxx (xx.x)	xxx (xx.x)	xxx (xx.x)	xxx (xx.x)	xxx (xx.x)	xxx (xx.x)
[Preferred Term 2]	xxx (xx.x)	xxx (xx.x)	xxx (xx.x)	xxx (xx.x)	xxx (xx.x)	xxx (xx.x)	xxx (xx.x)	xxx (xx.x)
[Preferred Term 3]	xxx (xx.x)	xxx (xx.x)	xxx (xx.x)	xxx (xx.x)	xxx (xx.x)	xxx (xx.x)	xxx (xx.x)	xxx (xx.x)
[Preferred Term 4]	xxx (xx.x)	xxx (xx.x)	xxx (xx.x)	xxx (xx.x)	xxx (xx.x)	xxx (xx.x)	xxx (xx.x)	xxx (xx.x)
…								
[System Organ Class 2]	xxx (xx.x)	xxx (xx.x)	xxx (xx.x)	xxx (xx.x)	xxx (xx.x)	xxx (xx.x)	xxx (xx.x)	xxx (xx.x)
[Preferred Term 1]	xxx (xx.x)	xxx (xx.x)	xxx (xx.x)	xxx (xx.x)	xxx (xx.x)	xxx (xx.x)	xxx (xx.x)	xxx (xx.x)
[Preferred Term 2]	xxx (xx.x)	xxx (xx.x)	xxx (xx.x)	xxx (xx.x)	xxx (xx.x)	xxx (xx.x)	xxx (xx.x)	xxx (xx.x)
…								
[System Organ Class 3]	xxx (xx.x)	xxx (xx.x)	xxx (xx.x)	xxx (xx.x)	xxx (xx.x)	xxx (xx.x)	xxx (xx.x)	xxx (xx.x)
…								

Note: Adverse events are coded using MedDRA version 19.0. All AEs reported during the study period are considered TEAEs. TEAEs that are present or ongoing at the beginning of Study 2797 (date of consent or date of last visit in Study 2553, if consent was obtained earlier) are considered pre-existing, if the event term, severity, and date and time of onset in Study 2797 are identical to the corresponding information given for the TEAE in Study 2553. If any of these conditions is not met, the TEAE is considered new or worsening. Only new or worsening TEAEs are summarized. For each system organ class and preferred term, subjects are included only once, in the maximum severity, for that TEAE. TEAEs without severity information are counted in the respective Total column for severity only.

Source: Listing xx.x.xx, Dataset: [NAME], Program: xxxxxx.sas, Output: xxxx.rtf, Generated on: DDMONYYYY HH:MM

Page x of y

Table 16.4.2.4 Treatment-Emergent Adverse Events by System Organ Class, Preferred Term, and Maximum Severity - Safety Overall Population (cont.)

	Total (N = XXX)			
**System Organ Class**<br>**Preferred Term**	**Mild**<br>**n (%)**	**Moderate**<br>**n (%)**	**Severe**<br>**n (%)**	**Total**<br>**n (%)**
Subjects with at least one TEAE	xxx (xx.x)	xxx (xx.x)	xxx (xx.x)	xxx (xx.x)
[System Organ Class 1]	xxx (xx.x)	xxx (xx.x)	xxx (xx.x)	xxx (xx.x)
[Preferred Term 1]]	xxx (xx.x)	xxx (xx.x)	xxx (xx.x)	xxx (xx.x)
[Preferred Term 2]	xxx (xx.x)	xxx (xx.x)	xxx (xx.x)	xxx (xx.x)
[Preferred Term 3]	xxx (xx.x)	xxx (xx.x)	xxx (xx.x)	xxx (xx.x)
[Preferred Term 4]	xxx (xx.x)	xxx (xx.x)	xxx (xx.x)	xxx (xx.x)
…				
[System Organ Class 2]	xxx (xx.x)	xxx (xx.x)	xxx (xx.x)	xxx (xx.x)
[Preferred Term 1]	xxx (xx.x)	xxx (xx.x)	xxx (xx.x)	xxx (xx.x)
[Preferred Term 2]	xxx (xx.x)	xxx (xx.x)	xxx (xx.x)	xxx (xx.x)
…				
[System Organ Class 3]	xxx (xx.x)	xxx (xx.x)	xxx (xx.x)	xxx (xx.x)
…				

Note: Adverse events are coded using MedDRA version 19.0. All AEs reported during the study period are considered TEAEs. TEAEs that are present or ongoing at the beginning of Study 2797 (date of consent or date of last visit in Study 2553, if consent was obtained earlier) are considered pre-existing, if the event term, severity, and date and time of onset in Study 2797 are identical to the corresponding information given for the TEAE in Study 2553. If any of these conditions is not met, the TEAE is considered new or worsening. Only new or worsening TEAEs are summarized. For each system organ class and preferred term, subjects are included only once, in the maximum severity, for that TEAE. TEAEs without severity information are counted in the respective Total column for severity only.
Source: Listing xx.x.xx, Dataset: [NAME], Program: xxxxxx.sas, Output: xxxx.rtf, Generated on: DDMONYYYY HH:MM
Page x of y

Programming Note: Sort alphabetically by SOC and then alphabetically for PT.

Table 16.4.2.5 Serious Treatment-Emergent Adverse Events by System Organ Class and Preferred Term - Safety Overall Population

Repeat SOC & PT Table 16.4.2.2. First row label is "Subjects with at least one serious TEAE."

Table 16.4.2.6 Study Medication-Related Treatment-Emergent Adverse Events by System Organ Class and Preferred Term - Safety Overall Population

Programming Note: Repeat SOC & PT Table 16.4.2.2. First row label is "Subjects with at least one study medication-related TEAE."

Table 16.4.2.7 Serious Study Medication-Related Treatment-Emergent Adverse Events by System Organ Class and Preferred Term - Safety Overall Population

Programming Note: Repeat Table SOC & PT 16.4.2.2. First row label is "Subjects with at least one serious study medication-related TEAE."

Table 16.4.2.8 Device-Related Treatment-Emergent Adverse Events by System Organ Class and Preferred Term - Safety Overall Population

Programming Note: Repeat Table SOC & PT 16.4.2.2. First row label is "Subjects with at least one device-related TEAE."

Table 16.4.2.9 Serious Device-Related Treatment-Emergent Adverse Events by System Organ Class and Preferred Term - Safety Overall Population

Programming Note: Repeat Table SOC & PT 16.4.2.2. First row label is "Subjects with at least one serious device-related TEAE."

Table 16.4.2.10 Treatment-Emergent Adverse Events of Special Interest by Preferred Term - Safety Overall Population

AESI Category<br>Preferred Term	GDNF/GDNF<br>(N = XXX)<br>n (%)	Placebo/GDNF<br>(N = XXX)<br>n (%)	Total<br>(N = XXX)<br>n ((%)
Subjects with at least one treatment-emergent AESI	xx (xx.x)	xx (xx.x)	xx (xx.x)
Dyskinesias	xx (xx.x)	xx (xx.x)	xx (xx.x)
[Preferred Term 1]	xx (xx.x)	xx (xx.x)	xx (xx.x)
[Preferred Term 2]	xx (xx.x)	xx (xx.x)	xx (xx.x)
Falls	xx (xx.x)	xx (xx.x)	xx (xx.x)
[Preferred Term 1]	xx (xx.x)	xx (xx.x)	xx (xx.x)
[Preferred Term 2]	xx (xx.x)	xx (xx.x)	xx (xx.x)
Adverse changes in mood	xx (xx.x)	xx (xx.x)	xx (xx.x)
[Preferred Term 1]	xx (xx.x)	xx (xx.x)	xx (xx.x)
[Preferred Term 2]	xx (xx.x)	xx (xx.x)	xx (xx.x)
Impulsivity	xx (xx.x)	xx (xx.x)	xx (xx.x)
[Preferred Term 1]	xx (xx.x)	xx (xx.x)	xx (xx.x)
[Preferred Term 2]	xx (xx.x)	xx (xx.x)	xx (xx.x)
…			

Note: Adverse events are coded using MedDRA version 19.0. All AEs reported during the study period are considered TEAEs. TEAEs that are present or ongoing at the beginning of Study 2797 (date of consent or date of last visit in Study 2553, if consent was obtained earlier) are considered pre-existing, if the event term, severity, and date and time of onset in Study 2797 are identical to the corresponding information given for the TEAE in Study 2553. If any of these conditions is not met, the TEAE is considered new or worsening. Only new or worsening TEAEs are summarized. For each category and preferred term, subjects are included only once, even if they experienced multiple events in that category or preferred term.
Source: Listing xx.x.xx, Dataset: [NAME], Program: xxxxxx.sas, Output: T_16_4_2_10_XXXXX.rtf, Generated on: DDMONYYYY HH:MM
Page 1 of 1

16.4.3 Laboratory Tables

Table 16.4.3.1 Clinically Significant Postbaseline Hematology Results - Safety Overall Population

Parameter	GDNF/GDNF (N = XXX) n (%)	Placebo/GDNF (N = XXX) n (%)
[Lab parameter 1 – high]	xxx (xx.x)	xxx (xx.x)
[Lab parameter 2]	xxx (xx.x)	xxx (xx.x)
[Lab parameter 3]	xxx (xx.x)	xxx (xx.x)
[Lab parameter 4]	xxx (xx.x)	xxx (xx.x)
[Lab parameter 5]	xxx (xx.x)	xxx (xx.x)
[Lab parameter 6]	xxx (xx.x)	xxx (xx.x)
[Lab parameter 7]	xxx (xx.x)	xxx (xx.x)
[Lab parameter 8]	xxx (xx.x)	xxx (xx.x)
…		

Note: Results were rated by the investigator as clinically significant on the CRF based on medical judgment, not using any pre-specified numerical criteria. For each parameter, subjects are included only once, even if they experienced more than one clinically significant result.
Source: Listing xx.x.xx, Dataset: [NAME], Program: xxxxxx.sas, Output: xxxx.rtf, Generated on: DDMONYYYY HH:MM
Page x of y

Programming Note: See text for parameters. Sort by order of parameters shown in SAP Table 5.

Table 16.4.3.2 Clinically Significant Postbaseline Serum Chemistry Results - Safety Overall Population

Programming Note: Repeat clinically significant hematology Table 16.4.3.1 for serum chemistry parameters.

16.4.4 ANTI-GDNF SERUM ANTIBODY TABLES

Table 16.4.4.1 Anti-GDNF Binding Serum Antibodies by Visit – Safety Overall Population

Visit<br>Variable	GDNF/GDNF<br>(N = XXX)<br>n (%)	Placebo/GDNF<br>(N = XXX)<br>n (%)
All visits[a]		
All Negative	xxx (xx.x)	xxx (xx.x)
1 Positive	xxx (xx.x)	xxx (xx.x)
> 1 Positive	xxx (xx.x)	xxx (xx.x)
Screening (Baseline)		
Positive	xxx (xx.x)	xxx (xx.x)
Negative	xxx (xx.x)	xxx (xx.x)
Not done	xxx (xx.x)	xxx (xx.x)
Week 40/e0		
Positive	xxx (xx.x)	xxx (xx.x)
Negative	xxx (xx.x)	xxx (xx.x)
Not done	xxx (xx.x)	xxx (xx.x)
Week 44/e4		
Positive	xxx (xx.x)	xxx (xx.x)
Negative	xxx (xx.x)	xxx (xx.x)
Not done	xxx (xx.x)	xxx (xx.x)
…		

[a] "All visits" includes all postbaseline visits from Study 2553 and Study 2797.
Source: Listing xx.x.xx, Dataset: [NAME], Program: xxxxxx.sas, Output: xxxx.rtf, Generated on: DDMONYYYY HH:MM
Page x of y

Programming Note: Visits are screening and Weeks 40/e0, 44/e4, 56/e16, 68/e28, 80/e40, e2-24, e2-48, e2-72, and Last Study Visit in Supplemental Extension.

Table 16.4.4.2 Anti-GDNF Neutralizing Serum Antibodies by Visit - Safety Overall Population

Programming Note: Repeat table for neutralizing antibodies.

16.4.5 Plasma GDNF Concentration Tables

Table 16.4.5 Plasma GDNF Concentrations by Visit - Safety Overall Population

Plasma GDNF Concentration (unit)<br>Visit<br>Statistic	GDNF/GDNF<br>(N = XXX)<br>Value	 <br>Change From Baseline	Placebo/GDNF<br>(N = XXX)<br>Value	 <br>Change From Baseline
Screening (Baseline)				
n	xx		xx	
Mean (SD)	xx.xx (xx.xxx)		xx.xx (xx.xxx)	
Median	xx.x		xx.x	
Min, Max	xx.x, xx.x		xx.x, xx.x	
Week 40/e0				
n	xx	xx	xx	xx
Mean (SD)	xx.xx (xx.xxx)	xx.xx (xx.xxx)	xx.xx (xx.xxx)	xx.xx (xx.xxx)
Median	xx.x	xx.x	xx.x	xx.x
Min, Max	xx.x, xx.x	xx.x, xx.x	xx.x, xx.x	xx.x, xx.x
Week 44/e4				
n	xx	xx	xx	Xx
Mean (SD)	xx.xx (xx.xxx)	xx.xx (xx.xxx)	xx.xx (xx.xxx)	xx.xx (xx.xxx)
Median	xx.x	xx.x	xx.x	xx.x
Min, Max	xx.x, xx.x	xx.x, xx.x	xx.x, xx.x	xx.x, xx.x
Week 56/e16				
n	xx	xx	xx	Xx
Mean (SD)	xx.xx (xx.xxx)	xx.xx (xx.xxx)	xx.xx (xx.xxx)	xx.xx (xx.xxx)
Median	xx.x	xx.x	xx.x	xx.x
Min, Max	xx.x, xx.x	xx.x, xx.x	xx.x, xx.x	xx.x, xx.x
Week 68/e28				
…				

Source: Listing xx.x.xx, Dataset: [NAME], Program: xxxxxx.sas, Output: xxxx.rtf, Generated on: DDMONYYYY HH:MM
Page x of y

Programming Note: Visits are screening and Weeks 40/e0, 44/e4, 56/e16, 68/e28, 80/e40, e2-24, e2-48, e2-72 and Last Study Visit in Supplemental Extension.

16.4.6 VITAL SIGN TABLE

Table 16.4.6 Clinically Relevant Postbaseline Abnormalities in Vital Sign Results in the Initial Extension - Safety Overall Population

Parameter and Criterion<br>..Visit<br>Time Point	GDNF/GDNF<br>(N = XXX)<br>n (%)	Placebo/GDNF<br>(N = XXX)<br>n (%)
[VS parameter and criterion 1] at any visit in Initial Extension	xxx (xx.x)	xxx (xx.x)
[Visit 1]	xxx (xx.x)	xxx (xx.x)
[Time point 1]	xxx (xx.x)	xxx (xx.x)
[Time point 2]	xxx (xx.x)	xxx (xx.x)
…		

Note: bpm = beats per minute. For each parameter and criterion, visit, and time point, subjects are included only once, even if they experienced more than one clinically relevant abnormality. For the comparisons of values during or after infusion with pre-infusion values, the baseline is the pre-infusion value.
Source: Listing xx.x.xx, Dataset: [NAME], Program: xxxxxx.sas, Output: xxxx.rtf, Generated on: DDMONYYYY HH:MM
Page x of y

Programming Note: Only include those VS parameters and criteria that have at least one clinically relevant abnormality present in the data. This table should include test infusion visits (interim visits after catheter repositioning and Week 80/e40 visits) as well as study medication infusion visits in the Initial Extension.

16.4.7 Electrocardiogram Tables

Table 16.4.7.1 Electrocardiogram Results by Visit – Safety Overall Population

Parameter (unit)<br>Visit<br>Statistic	GDNF/GDNF<br>(N = XXX)		Placebo/GDNF<br>(N = XXX)	
	**Value**	**Change From Baseline**	**Value**	**Change From Baseline**
Heart rate (beats/minute)				
Screening (Baseline)				
n	xx		xx	
Mean (SD)	xx.xx (xx.xxx)		xx.xx (xx.xxx)	
Median	xx.x		xx.x	
Min, Max	xx.x, xx.x		xx.x, xx.x	
Week 40/e0				
n	xx	xx	xx	xx
Mean (SD)	xx.xx (xx.xxx)	xx.xx (xx.xxx)	xx.xx (xx.xxx)	xx.xx (xx.xxx)
Median	xx.x	xx.x	xx.x	xx.x
Min, Max	xx.x, xx.x	xx.x, xx.x	xx.x, xx.x	xx.x, xx.x
Week 80/e40				
n	xx	xx	xx	Xx
Mean (SD)	xx.xx (xx.xxx)	xx.xx (xx.xxx)	xx.xx (xx.xxx)	xx.xx (xx.xxx)
Median	xx.x	xx.x	xx.x	xx.x
Min, Max	xx.x, xx.x	xx.x, xx.x	xx.x, xx.x	xx.x, xx.x

Note: Hodges QT correction formula was used.
Source: Listing xx.x.xx, Dataset: [NAME], Program: xxxxxx.sas, Output: xxxx.rtf, Generated on: DDMONYYYY HH:MM
Page x of y

Programming Note: Visits are screening, Week 40/e0 and Week 80/e40. Parameters are heart rate (beats/min), PR interval (ms), QRS interval (ms), QT interval (ms), and QTc interval (ms).

Table 16.4.7.2 Summary of Electrocardiogram Results at Week 80/e40 - Safety Overall Population

ECG Evaluation	GDNF/GDNF (N = XXX) n (%)	Placebo/GDNF (N = XXX) n (%)
Overall impression		
Normal Week 80/e40 result	xxx (xx.x)	xxx (xx.x)
Any abnormal Week 80/e40 result	xxx (xx.x)	xxx (xx.x)
Any clinically significant abnormal Week 80/e40 result	xxx (xx.x)	xxx (xx.x)
Any clinically relevant abnormal Week 80/e40 QTc interval result based on criteria below	xxx (xx.x)	xxx (xx.x)
[Criterion 1]	xxx (xx.x)	xxx (xx.x)
[Criterion 2]	xxx (xx.x)	xxx (xx.x)
…		

Note: Overall ECG impression was rated by the investigator as abnormal and clinically significant on the CRF based on medical judgment. Hodges QT correction formula was used.

Source: Listing xx.x.xx, Dataset: [NAME], Program: xxxxxx.sas, Output: xxxx.rtf, Generated on: DDMONYYYY HH:MM
Page x of y

Programming Note: Present all QTc parameters even if no subjects had an abnormal result.

16.4.8 GLASGOW COMA SCALE TABLE

Table 16.4.8 Glasgow Coma Scale Score = 15 or < 15 During or After Infusion by Visit and Time Point in the Initial Extension– Safety Overall Population

Visit and Time Point<br>Glasgow Coma Scale Score	GDNF/GDNF<br>(N = XXX)<br>n (%)	Placebo/GDNF<br>(N = XXX)<br>n (%)
Week 40/e0 at any time during or after infusion		
All scores = 15	xx (xx.x)	xx (xx.x)
Any score < 15	xx (xx.x)	xx (xx.x)
No score < 15 but at least one score is missing	xx (xx.x)	xx (xx.x)
Week 44/e4 at any time during or after infusion		
....		
Week 80/e40 at any time during or after infusion		
…		

Note: Glasgow Coma Scale items include visual response, verbal ability, and motor skills. Each item has 4-6 possible responses. The best possible total score is 15.
Source: Listing xx.x.xx, Dataset: [NAME], Program: xxxxxx.sas, Output: xxxx.rtf, Generated on: DDMONYYYY HH:MM
Page x of y

Programming Note: Present for all test infusion visits and study medication infusion visits in the Initial Extension. Add information in Table 8 as an endnote.

16.4.9 Other Safety Tables

Table 16.4.9.1 QUIP by Visit - Safety Overall Population

	GDNF/GDNF (N = XXX)			Placebo/GDNF (N = XXX)		
	Postbaseline Value			Postbaseline Value		
**Assessor<br>Parameter<br>Visit<br>Baseline Value**	**Positive<br>n (%)**	**Negative<br>n (%)**	**Missing<br>n (%)**	**Positive<br>n (%)**	**Negative<br>n (%)**	**Missing<br>n (%)**
Subject assessment						
Issue with too much gambling						
Week 48/e8						
Positive	xxx (xx.x)	xxx (xx.x)	xxx (xx.x)	xxx (xx.x)	xxx (xx.x)	xxx (xx.x)
Negative	xxx (xx.x)	xxx (xx.x)	xxx (xx.x)	xxx (xx.x)	xxx (xx.x)	xxx (xx.x)
Missing	xxx (xx.x)	xxx (xx.x)	xxx (xx.x)	xxx (xx.x)	xxx (xx.x)	xxx (xx.x)
Week 56/e16						
Positive	xxx (xx.x)	xxx (xx.x)	xxx (xx.x)	xxx (xx.x)	xxx (xx.x)	xxx (xx.x)
Negative	xxx (xx.x)	xxx (xx.x)	xxx (xx.x)	xxx (xx.x)	xxx (xx.x)	xxx (xx.x)
Missing	xxx (xx.x)	xxx (xx.x)	xxx (xx.x)	xxx (xx.x)	xxx (xx.x)	xxx (xx.x)
…						

Note: The QUIP is a self-administered or informant-completed scale that includes 13 questions covering symptoms related to the 4 commonest impulse control disorders in PD, other behaviors, and problematic use of medication. The number of "Yes" responses is compared to the number required for a positive result. Missing individual responses are imputed using LOCF. Each impulse control disorder, other behavior, and problematic use of medication is rated as a positive result (ie, present) or a negative result (ie, not present). Percentages are based on the total number of observations for that visit.
Source: Listing xx.x.xx, Dataset: [NAME], Program: xxxxxx.sas, Output: xxxx.rtf, Generated on: DDMONYYYY HH:MM
Page x of y

Programming Note: Visits are Week 40/e0 (baseline), Weeks 48/e8, 56/e16, 64/e24, 72/e32, and 80/e40; e2 16, e2-32, e2-48, e2-64, and e2-80; e3-16, e3-32, and the last study visit in the Supplemental Extension. Assessor includes subject assessment and informant assessment. Parameters (30) include: Issue with too much gambling, issue with too much sex, issue with too much buying, issue with too much eating,
Think too much about gambling, think too much about sex, think too much about buying, think too much about eating,
Excessive or distressing urge for gambling, excessive or distressing urge for sex, excessive or distressing urge for buying, excessive or distressing urge for eating,
Difficulty controlling gambling, difficulty controlling sex, difficulty controlling buying, difficulty controlling eating,
Engage in activities specifically to continue gambling, engage in activities specifically to continue sex, engage in activities specifically to continue buying, engage in activities specifically to continue eating, Spend too much time on specific tasks hobbies or other organized activities, spend too much time repeating certain simple

motor activities, spend too much time walking or driving with no intended goal or specific purpose, difficulty controlling the amount of time spent on these activities, activities interfere with daily functioning or cause relationship or work difficulties,
Consistently take too much PD medications, increased PD medications without medical advice for psychological reasons, increased PD medications without medical advice because only feel fully "on" when dyskinetic, difficulty controlling use of PD medications, hoard or hide PD medications to increase overall dosage.

Table 16.4.9.2 MoCA by Visit - Safety Overall Population

Programming Note: Repeat Table 16.4.5 (Electrocardiogram results by visit specified) for MoCA total score. Visits are screening, pre-test infusion (baseline), Weeks 40/e0, 56/e16, and 80/e40; e2-80; and the last study visit in the Supplemental Extension. Add footnote: "Note: The MoCA is a rater-administered cognitive screening tool with 8 components. The total score ranges from 0 to 30, with lower scores representing poorer cognitive function. A total score of 26 or above is considered normal. Missing individual scores are imputed using LOCF if necessary."

Table 16.4.9.3 MDRS by Visit - Safety Overall Population

Programming Note: Repeat Table 16.4.5 (Electocardiogram results by visit specified) for MDRS total score. Visits are screening (baseline), Weeks 40/e0, 56/e16 and 80/e40; e2-80; and the last study visit in the Supplemental Extension. Add footnote: "Note: The MDRS is a rater-administered global scale of cognition including 5 subscales. The total score ranges from 0 to 144, with higher scores representing better cognitive function. A total score lower than 123 is associated with some degree of dementia in PD. The AEMSS total score ranges from 0 to 20, with higher scores representing better cognitive function. Individual missing items are imputed using the average of non-missing scores in each subscale if necessary."

Table 16.4.9.4 Stroop Test by Visit, Safety Overall Population

Programming Note: Repeat Table 16.4.5 (Electocardiogram results by visit specified) for Stroop test parameters. The parameters are the 4 conditions: color naming, word reading, inhibition, and inhibition/switching. Visits are screening, Week 40/e0, and Week 80/e40. Add footnote: "Note: The Stroop test is a global scale of reaction time including 4 conditions. Total time to complete the test in each condition can range from 0 to 999 seconds, with lower time representing better reaction time."

Table 16.4.9.5 FrSBe by Visit - Safety Overall Population

Programming Note: Repeat Table 16.4.5 (Electocardiogram results by visit specified) for FrSBe parameters. The parameters are the 3 subscales: apathy, disinhibition, and executive dysfunction. Total scores are not calculated. Visits are screening "before", screening "after" (baseline), Week 40/e0 "after", and Week 80/e40 "after." Add footnote: "Note: The FrSBe is a scale that assesses behavior related to frontal systems damage including 3 subscales. Higher subscale scores indicate greater pathology. Individual missing items are imputed using the average of non-missing scores in each subscale."

Table 16.4.9.6 Deary-Liewald Four-Choice Reaction Time by Visit - Safety Overall Population

Programming Note: Repeat Table 16.4.5 (Electocardiogram results by visit specified) for RT parameters. Visits are screening (baseline), Week 40/e0, and Weeks 56/e16 and 80/e40. Add footnote: "Note: The Deary-Liewald RT is a computerized measure of simple and four-choice reaction time. The parameter is the mean reaction time, variance and SD for correct responses for four-choice reaction time. A shorter reaction time is better."

Table 16.4.9.7 Verbal Fluency Assessment by Visit - Safety Overall Population

Programming Note: Repeat Table 16.4.5 (Electocardiogram results by visit specified) for *for verbal fluency parameters. Parameters are phonemic and semantic verbal fluency. Visits are screening (baseline), Week 40/e0, and Week 80/e40. Add footnote: "Note: The verbal fluency assessment measures verbal functioning in 2 categories. Scores represent number of correct words in one minute and range from 0 to 200. Higher scores represent better verbal functioning."*

Table 16.4.9.8 BDI by Visit - Safety Overall Population

Programming Note: Repeat Table 16.4.5 (Electocardiogram results by visit specified) for *for BDI parameters. The parameter is the total score. Visits are screening (baseline), Week 40/e0, and Week 80/e40; and the last study visit in both the Pilot Extension and the Supplemental Extension. Add footnote: "Note: The BDI is a self-administered test which consists of 21 questions that measure the severity of depression. Scores range from 0 to 63, with higher scores representing worse depression. Individual missing items are imputed using the average of non-missing scores in each subscale."*

Table 16.4.9.9 UPSIT by Visit - Safety Overall Population

Programming Note: Repeat Table 16.4.5 (Electocardiogram results by visit specified) for UPSIT parameters. The parameter is the number of correct responses out of 40 total items. *Visits are screening (baseline), Week 40/e0, and Week 80/e40. Add footnote: "Note: The UPSIT is a self-administered test which can be used to identify and quantitate olfactory dysfunction in PD. The number of correct responses out of 40 total items constitutes a subject's score. Lower scores represent greater olfactory dysfunction. Individual missing responses are imputed as zeros (ie, incorrect responses)." Add information in SAP Table 9 as an endnote.*

16.5 FIGURES

Figure 16.5.1.1.1 OFF State UPDRS Motor Score (Part III): Percentage Change Over Time - ITT Primary Population

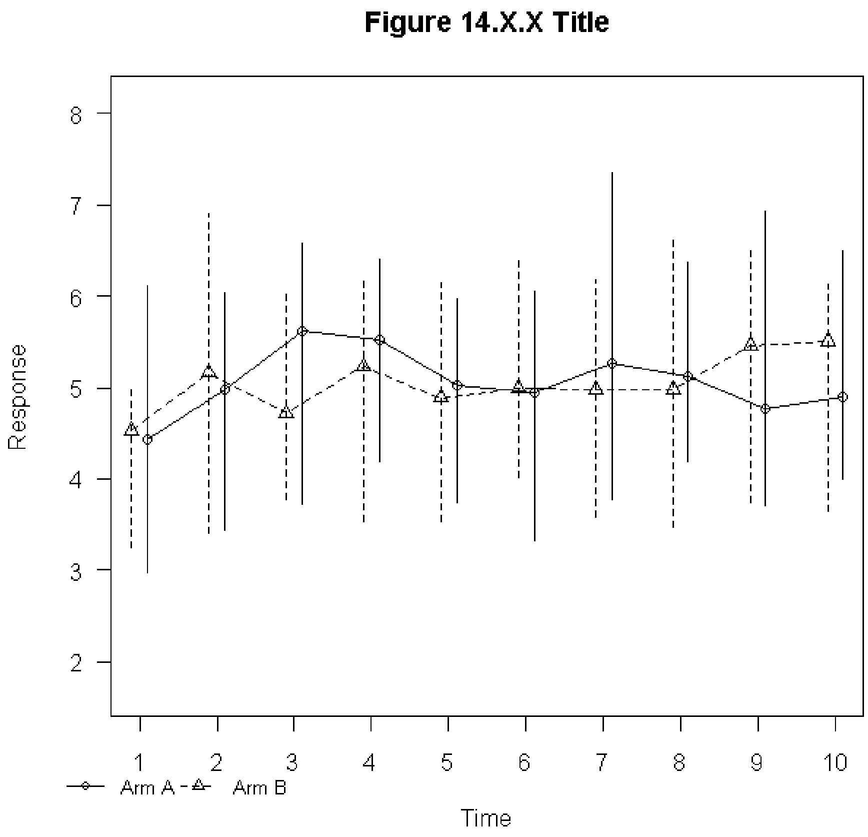

Note: Data points represent means, and error bars represent standard errors. For subject 45, items 22, 27, 28, 29 and 30 are excluded from the score.

Programming Note: Replace with appropriate titles, labels (eg, for y-axis "Change in OFF state UPDRS Motor Score from baseline [unit]"), and legend. Visits are Weeks 0, 8, 16, 24, 32, 40/e0, 48/e8, 56/e16, 64/e24, 72/e32 and 80/e40. Add a line for the modelled control using the values 3.3% at Week 40 and 6.7% at Week 80.

Figure 16.5.1.1.2 OFF State UPDRS Motor Score (Part III): Percentage Change Over Time - ITT Overall Population

Programming Note: Repeat figure above for OFF state UPDRS motor score (part III) percentage change over time in the ITT Overall Population. Visits are Weeks 0, 8, 16, 24, 32, 40/e0, 48/e8, 56/e16, 64/e24, 72/e32 and 80/e40.

Figure 16.5.1.2.1 OFF State UPDRS Motor Score (Part III): Change Over Time - ITT Primary Population

Programming Note: Repeat figure above for OFF state UPDRS motor score (part III) absolute change over time in the ITT Primary Population. Visits are Weeks 0, 8, 16, 24, 32, 40/e0, 48/e8, 56/e16, 64/e24, 72/e32 and 80/e40.

Figure 16.5.1.2.2 OFF State UPDRS Motor Score (Part III): Change Over Time - ITT Overall Population

Programming Note: Repeat figure above for OFF state UPDRS motor score (part III) absolute change over time in the ITT Overall Population. Visits are Weeks 0, 8, 16, 24, 32, 40/e0, 48/e8, 56/e16, 64/e24, 72/e32 and 80/e40.

Figure 16.5.1.3 ON State UPDRS Motor Score (Part III): Change Over Time - ITT Overall Population

Programming Note: Repeat figure above for ON state UPDRS motor score (part III) absolute change in the ITT Overall Population. Visits are Weeks 0, 8, 16, 24, 32, 40/e0, 48/e8, 56/e16, 64/e24, 72/e32 and 80/e40.

Figure 16.5.1.4.1 OFF State UPDRS ADL Score (Part II): Change Over Time - ITT Primary Population

Programming Note: Repeat figure above for OFF state UPDRS ADL score (part II) absolute change in the ITT Primary Population. Visits are Weeks 0, 8, 16, 24, 32, 40/e0, 48/e8, 56/e16, 64/e24, 72/e32 and 80/e40. Change footnote regarding subject 45 to: "Data for Subject 45 are excluded from analysis."

Figure 16.5.1.4.2 OFF State UPDRS ADL Score (Part II): Change Over Time - ITT Overall Population

Programming Note: Repeat figure above for OFF state UPDRS ADL score (part II) absolute change in the ITT Overall Population. Visits are Weeks 0, 8, 16, 24, 32, 40/e0, 48/e8, 56/e16, 64/e24, 72/e32 and 80/e40. Change footnote regarding subject 45 to: "Data for Subject 45 are excluded from analysis."

Figure 16.5.1.5 ON State UPDRS ADL Score (Part II): Change Over Time - ITT Overall Population

Programming Note: Repeat figure above for ON state UPDRS ADL score (part II) absolute change in the ITT Overall Population. Visits are Weeks 0, 8, 16, 24, 32, 40/e0, 48/e8, 56/e16, 64/e24, 72/e32 and 80/e40. Change footnote regarding subject 45 to: "Data for Subject 45 are excluded from analysis."

Figure 16.5.1.6.1 OFF State UPDRS Total Score: Change Over Time - ITT Primary Population

Programming Note: Repeat figure above for OFF state UPDRS total score (parts II+III) absolute change in the ITT Primary Population. Visits are Weeks 0, 8, 16, 24, 32, 40/e0, 48/e8, 56/e16, 64/e24, 72/e32 and 80/e40. Change footnote regarding subject 45 to: "Data for Subject 45 are excluded from analysis."

Figure 16.5.1.6.2 OFF State UPDRS Total Score: Change Over Time - ITT Overall Population

Programming Note: Repeat figure above for OFF state UPDRS total score (parts II+III) absolute change in the ITT Overall Population. Visits are Weeks 0, 8, 16, 24, 32, 40/e0, 48/e8, 56/e16, 64/e24, 72/e32 and 80/e40. Change footnote regarding subject 45 to: "Data for Subject 45 are excluded from analysis."

Figure 16.5.1.7 ON State UPDRS Total Score: Change Over Time - ITT Overall Population

Programming Note: Repeat figure above for ON state UPDRS total score (parts II+III) absolute change in the ITT Overall Population. Visits are Weeks 0, 8, 16, 24, 32, 40/e0, 48/e8, 56/e16, 64/e24, 72/e32 and 80/e40. Change footnote regarding subject 45 to: "Data for Subject 45 are excluded from analysis."

Figure 16.5.2.1 Motor Fluctuation Diary Total OFF Time Per Day (Hours): Change Over Time - ITT Primary Population

Programming Note: Repeat figure above for OFF time per day (hours). Visits are Weeks 0, 8, 16, 24, 32, 40/e0, 48/e8, 56/e16, 64/e24, 72/e32 and 80/e40. Delete footnote regarding subject 45.

Figure 16.5.2.2 Motor Fluctuation Diary Total Good-Quality ON Time Per Day (Hours): Change Over Time - ITT Primary Population

Programming Note: Repeat figure above for good-quality ON time per day (hours). Visits are Weeks 0, 8, 16, 24, 32, 40/e0, 48/e8, 56/e16, 64/e24, 72/e32 and 80/e40. Delete footnote regarding subject 45. Add footnote "Note: Total good-quality ON time per day is defined as the sum of ON time per day without dyskinesias + ON time per day with non-troublesome dyskinesias."

Figure 16.5.3 OFF State UPDRS Motor Score (Part III): Subject Scores Over Time - ITT Primary Population

Figure	16.5.3
Title 1	OFF State UPDRS Motor Score (Part III): Subject Scores Over Time
Title 2	ITT Primary Population
Type of graph	Lineplot
y-axis	Individual subject OFF State UPDRS Motor Score
y-axis (label)	OFF State UPDRS Motor Score
x-axis	Visit
x-axis (label)	Visit
Legend (if applicable)	Not applicable
Footnote 1	Note: For subject 45, items 22, 27, 28, 29 and 30 are excluded from the score.
Footnote 2	
Additional information	GDNF/GDNF and placebo/GDNF groups should be on separate pages, keeping the y-axis scale the same for both

Figure 16.5.4.1 OFF State UPDRS Motor Score (Part III): Frequency Distribution of Change at Week 40/e0 - ITT Overall Population

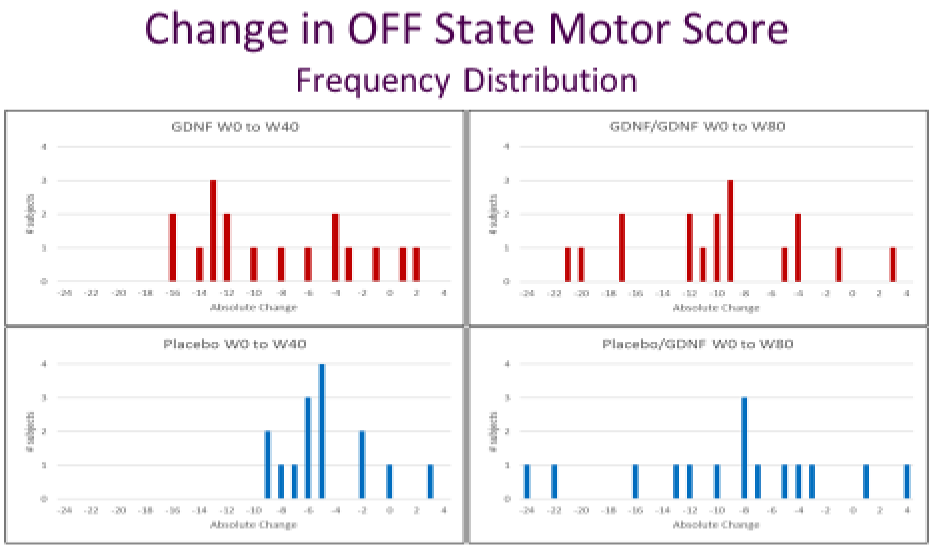

Note: For subject 45, items 22, 27, 28, 29 and 30 are excluded from the score.

Programming Note: Replace with appropriate titles, labels (eg, for x-axis “Change in OFF state UPDRS Motor Score at Week 40/e0 from baseline [unit]”), and legend. Only create one panel for change at Week 40 but include both treatment groups on the same graph with an appropriate legend.

Figure 16.5.4.2 OFF State UPDRS Motor Score (Part III): Frequency Distribution of Change at Week 80/e40 - ITT Overall Population
Programming Note: Repeat figure above for Week 80/e40.

Figure 16.5.5.1 Correlation Analysis of Percentage Change from Baseline to Week 80/e40 in OFF State UPDRS Motor Score (Part III) to Volume of Interest Coverage at Baseline as Determined by Contrast-Enhanced T1-Weighted MRI - ITT Primary Population

Figure	16.5.5.1
Title 1	Correlation Analysis of Percentage Change from Baseline to Week 80/e40 in OFF State UPDRS Motor Score (Part III) to Volume of Interest Coverage at Baseline as Determined by Contrast-Enhanced T1-Weighted MRI
Title 2	ITT Primary Population
Type of graph	Scatterplot
y-axis	Change From Baseline to Week 80/e40 in OFF State UPDRS Motor Score (Part III) (%)
y-axis (label)	Change From Baseline to Week 80/e40 in OFF State UPDRS Motor Score (Part III) (%)
x-axis	Volume of Interest Coverage at Baseline, Average of Both Putamina (%)
x-axis (label)	Volume of Interest Coverage at Baseline, Average of Both Putamina (%)
Legend (if applicable)	Treatment group symbol (please include N’s)
Footnote 1	Note: Analysis uses non-parametric Spearman rank correlation. For subject 45, items 22, 27, 28, 29 and 30 are excluded from the score.
Footnote 2	
Additional information	Include estimated correlation coefficient value and p-value.

Figure 16.5.5.2 Correlation Analysis of Percentage Change from Baseline to Week 80/e40 in OFF State UPDRS Motor Score (Part III) to Total Putamenal Coverage at Baseline as Determined by Contrast-Enhanced T1-Weighted MRI - ITT Primary Population

Figure	16.5.5.2
Title 1	Correlation Analysis of Percentage Change from Baseline to Week 80/e40 in OFF State UPDRS Motor Score (Part III) to Total Putamenal Coverage at Baseline as Determined by Contrast-Enhanced T1-Weighted MRI
Title 2	ITT Primary Population
Type of graph	Scatterplot
y-axis	Change From Baseline to Week 80/e40 in OFF State UPDRS Motor Score (Part III) (%)
y-axis (label)	Change From Baseline to Week 80/e40 in OFF State UPDRS Motor Score (Part III) (%)
x-axis	Total Putamenal Coverage at Baseline, Average of Both Putamina (%)
x-axis (label)	Total Putamenal Coverage at Baseline, Average of Both Putamina (%)
Legend (if applicable)	Treatment group symbol (please include N’s)
Footnote 1	Note: Analysis uses non-parametric Spearman rank correlation. For subject 45, items 22, 27, 28, 29 and 30 are excluded from the score.
Footnote 2	
Additional information	Include estimated correlation coefficient value and p-value.

Figure 16.5.5.3 Correlation Analysis of Percentage Change from Baseline to Week 80/e40 in OFF State UPDRS Motor Score (Part III) to Change From Baseline to Week 40/e0 in ^{18}F-DOPA Uptake as Determined by PET Scan - ITT Primary Population

Programming Note: Repeat above figures. Parameters are "Percentage change from baseline to Week 80/e40 in OFF state UPDRS motor score (part III)" and "Change from baseline to Week 40/e0 in ^{18}F-DOPA uptake rate constant, average of both hemispheres". Perform separate analyses for each of the 5 regions (dorsal caudate nucleus, dorsal anterior putamen, dorsal central/posterior putamen, ventral striatum, and substantia nigra). ***Figure will have 5 pages, one for each of 5 regions; please subtitle each page clearly.***

Figure 16.5.5.4 Correlation Analysis of Baseline (Week 0) OFF State UPDRS Motor Score (Part III) to Baseline (Week 0) ^{18}F-DOPA Uptake as Determined by PET Scan for GDNF/GDNF Subjects - ITT Overall Population

Programming Note: Repeat above figure. Parameters are "Baseline (Week 0) OFF state UPDRS motor score (part III)" and "Baseline (Week 0) ^{18}F-DOPA uptake rate constant for dorsal central/posterior putamen and for dorsal anterior putamen, averages of both hemispheres". ***Figure will have 2 pages, one for each region; please subtitle each page clearly.***

Figure 16.5.5.5 Correlation Analysis of Baseline (Week 0) OFF State UPDRS Motor Score (Part III) to Baseline (Week 0) ^{18}F-DOPA Uptake as Determined by PET Scan for Placebo/GDNF Subjects - ITT Overall Population

Programming Note: Repeat above figure. Parameters are "Baseline (Week 0) OFF state UPDRS motor score (part III)" and "Baseline (Week 0) ^{18}F-DOPA uptake rate constant for dorsal central/posterior putamen and for dorsal anterior putamen, averages of both hemispheres". ***Figure will have 2 pages, one for each region; please subtitle each page clearly.***

Figure 16.5.5.6 Correlation Analysis of Baseline (Week 0) OFF State UPDRS ADLScore (Part II) to Baseline (Week 0) ^{18}F-DOPA Uptake as Determined by PET Scan for GDNF/GDNF Subjects - ITT Overall Population

Programming Note: Repeat above figure. Parameters are "Baseline (Week 0) OFF state UPDRS ADL score (part II)" and "Baseline (Week 0) ^{18}F-DOPA uptake rate constant for dorsal central/posterior putamen and for dorsal anterior putamen, averages of both hemispheres". ***Figure will have 2 pages, one for each region; please subtitle each page clearly.***

Figure 16.5.5.7 Correlation Analysis of Baseline (Week 0) OFF State UPDRS ADL Score (Part II) to Baseline (Week 0) ^{18}F-DOPA Uptake as Determined by PET Scan for Placebo/GDNF Subjects - ITT Overall Population

Programming Note: Repeat above figure. Parameters are "Baseline (Week 0) OFF state UPDRS ADL score (part II)" and "Baseline (Week 0) ^{18}F-DOPA uptake rate constant for dorsal central/posterior putamen and for dorsal anterior putamen, averages of both hemispheres". ***Figure will have 2 pages, one for each region; please subtitle each page clearly.***

Figure 16.5.5.8 Correlation Analysis of Week 40/e0 OFF State UPDRS Motor Score (Part III) to Week 40/e0 ^{18}F-DOPA Uptake as Determined by PET Scan for GDNF/GDNF Subjects - ITT Overall Population

Programming Note: Repeat above figure. Parameters are "Week 40/e0 OFF state UPDRS motor score (part III)" and "Week 40/e0 ^{18}F-DOPA uptake rate constant for dorsal central/posterior putamen and for dorsal anterior putamen, averages of both hemispheres". ***Figure will have 2 pages, one for each region; please subtitle each page clearly.***

Figure 16.5.5.9 Correlation Analysis of Week 40/e0 OFF State UPDRS Motor Score (Part III) to Week 40/e0 ^{18}F-DOPA Uptake as Determined by PET Scan for Placebo/GDNF Subjects - ITT Overall Population

Programming Note: Repeat above figure. Parameters are "Week 40/e0 OFF state UPDRS motor score (part III)" and "Week 40/e0 ^{18}F-DOPA uptake rate constant for dorsal central/posterior putamen and for dorsal anterior putamen, averages of both hemispheres". ***Figure will have 2 pages, one for each region; please subtitle each page clearly.***

Figure 16.5.5.10 Correlation Analysis of Week 40/e0 OFF State UPDRS ADLScore (Part II) to Week 40/e0 ^{18}F-DOPA Uptake as Determined by PET Scan for GDNF/GDNF Subjects - ITT Overall Population

Programming Note: Repeat above figure. Parameters are "Week 40/e0 OFF state UPDRS ADL score (part II)" and "Week 40/e0 ^{18}F-DOPA uptake rate constant for dorsal central/posterior putamen and for dorsal anterior putamen, averages of both hemispheres". ***Figure will have 2 pages, one for each region; please subtitle each page clearly.***

Figure 16.5.5.11 Correlation Analysis of Week 40/e0 OFF State UPDRS ADL Score (Part II) to Week 40/e0 ^{18}F-DOPA Uptake as Determined by PET Scan for Placebo/GDNF Subjects - ITT Overall Population

Programming Note: Repeat above figure. Parameters are "Week 40/e0 OFF state UPDRS ADL score (part II)" and "Week 40/e0 ^{18}F-DOPA uptake rate constant for dorsal central/posterior putamen and for dorsal anterior putamen, averages of both hemispheres". ***Figure will have 2 pages, one for each region; please subtitle each page clearly.***

Figure 16.5.5.12 Correlation Analysis of Week 80/e40 OFF State UPDRS Motor Score (Part III) to Week 40/e0 ^{18}F-DOPA Uptake as Determined by PET Scan for GDNF/GDNF Subjects - ITT Overall Population

Programming Note: Repeat above figure. Parameters are "Week 80/e40 OFF state UPDRS motor score (part III)" and "Week 40/e0 ^{18}F-DOPA uptake rate constant for dorsal central/posterior putamen and for dorsal anterior putamen, averages of both hemispheres". ***Figure will have 2 pages, one for each region; please subtitle each page clearly.***

Figure 16.5.5.13 Correlation Analysis of Week 80/e40 OFF State UPDRS Motor Score (Part III) to Week 40/e0 ^{18}F-DOPA Uptake as Determined by PET Scan for Placebo/GDNF Subjects - ITT Overall Population

Programming Note: Repeat above figure. Parameters are "Week 80/e40 OFF state UPDRS motor score (part III)" and "Week 40/e0 ^{18}F-DOPA uptake rate constant for dorsal central/posterior putamen and for dorsal anterior putamen, averages of both hemispheres". ***Figure will have 2 pages, one for each region; please subtitle each page clearly.***

Figure 16.5.5.14 Correlation Analysis of Week 80/e40 OFF State UPDRS ADLScore (Part II) to Week 40/e0 ^{18}F-DOPA Uptake as Determined by PET Scan for GDNF/GDNF Subjects - ITT Overall Population

Programming Note: Repeat above figure. Parameters are "Week 80/e40 OFF state UPDRS ADL score (part II)" and "Week 40/e0 ^{18}F-DOPA uptake rate constant for dorsal central/posterior putamen and for dorsal anterior putamen, averages of both hemispheres". ***Figure will have 2 pages, one for each region; please subtitle each page clearly.***

Figure 16.5.5.15 Correlation Analysis of Week 80/e40 OFF State UPDRS ADL Score (Part II) to Week 40/e0 ^{18}F-DOPA Uptake as Determined by PET Scan for Placebo/GDNF Subjects - ITT Overall Population

Programming Note: Repeat above figure. Parameters are "Week 80/e40 OFF state UPDRS ADL score (part II)" and "Week 40/e0 ^{18}F-DOPA uptake rate constant for dorsal central/posterior putamen and for dorsal anterior putamen, averages of both hemispheres". ***Figure will have 2 pages, one for each region; please subtitle each page clearly.***

Programming note for all correlation figures: *Use the same scale for the axes in all plots for a given clinical outcome.*

17 DATA LISTINGS

17.2.1 BASELINE LISTINGS

Listing 17.2.1.1 Study Completion Status - ITT Overall Population

ITT Pilot Stage (N=XXX)

GDNF/GDNF (N=XXX)

Subject ID Age/Race/Ethnicity/ Sex	Completed Initial Extension?	Last visit	Date of Last Dose of Study Medication	Date of Discontinuation	Primary Reason for Discontinuation
xxxxxxxxxx/xx/x/x/x	Yes/No	Week XX, DDMMMYYYY	DDMMMYYYY	DDMMMYYYY	Xxxxxxxxxxxxxxxxxxxxxxxxxxxxxxxxxxxxxxxxxxxx

Note: W = white; B = black; P = native Hawaiian or other Pacific islander; A = Asian; I = American Indian or Alaska native; H = Hispanic or Latino; N = not Hispanic or Latino; M = male; F = female.
Source: Dataset: [NAME], Program: xxxxxx.sas, Output: xxxx.rtf, Generated on: DDMONYYYY HH:MM
Page x of y

Programming Note: Repeat listing for ITT Pilot Stage (N=XXX) Placebo/GDNF (N=XXX), then ITT Primary Stage (N=XXX) GDNF/GDNF (N=XXX) and Placebo/GDNF (N=XXX). Sort by stage, treatment group, and subject ID. Include all ITT Overall subjects. If primary reason for discontinuation is Other, then concatenate specify text.

Listing 17.2.1.2.1 Major Protocol Deviations - ITT Overall Population

ITT Pilot Stage (N=XXX)			
GDNF/GDNF (N=XXX)			
**Subject ID Age/Race/Ethnicity/ Sex**	**Date of Deviation**	**Category**	**Deviation (brief description)**
xxxxxxxxxx/xx/x/x/x	DDMMMYYYY	Xxxxxxxxxxxxxxxxxxxx	Xxxxxxxxxxxxxxxxxxxxxxxxxxxxxxxxxxxxxxxxxxxxxxxxxxxxxxxxxxxxxxxx

Note: W = white; B = black; P = native Hawaiian or other Pacific islander; A = Asian; I = American Indian or Alaska native; H = Hispanic or Latino; N = not Hispanic or Latino; M = male; F = female.
Source: Dataset: [NAME], Program: xxxxxx.sas, Output: xxxx.rtf, Generated on: DDMONYYYY HH:MM
Page x of y

Programming Note: Repeat listing for ITT Pilot Stage (N=XXX) Placebo/GDNF (N=XXX), then ITT Primary Stage (N=XXX) GDNF/GDNF (N=XXX) and Placebo/GDNF (N=XXX). Sort by stage, treatment group, and subject ID. Include all ITT Overall subjects with major protocol deviations.

Listing 17.2.1.2.2 Minor Protocol Deviations - ITT Overall Population

Programming Note: Repeat listing above for minor protocol deviations.

Listing 17.2.1.3 Subject Populations - ITT Overall Population

ITT Pilot Stage (N=XXX)			
GDNF/GDNF (N=XXX)			
**Subject ID Age/Race/Ethnicity/Sex**	**Treatment Received?**	**Safety Population**	**ITT Population**
xxxxxxxxxx/xx/x/x/x	Yes	Yes	Yes

Note: W = white; B = black; P = native Hawaiian or other Pacific islander; A = Asian; I = American Indian or Alaska native; H = Hispanic or Latino; N = not Hispanic or Latino; M = male; F = female.
<Subjects who are not treated are excluded from the safety population; subjects with no postbaseline assessments are excluded from the ITT population.>
Source: Dataset: [NAME], Program: xxxxxx.sas, Output: xxxx.rtf, Generated on: DDMONYYYY HH:MM
Page x of y

Programming Note: Repeat listing for ITT Pilot Stage (N=XXX) Placebo/GDNF (N=XXX), then ITT Primary Stage (N=XXX) GDNF/GDNF (N=XXX) and Placebo/GDNF (N=XXX). Sort by stage, treatment group, and subject ID. Include all ITT Overall subjects.

Listing 17.2.1.4.1 Demographic Characteristics in Study 2553 - ITT Overall Population

ITT Pilot Stage (N=XXX)					
GDNF/GDNF (N=XXX)					
**Subject ID Age/Race/Ethnicity/ Sex**	**Date of Birth**	**Race (specify if Other)**	**Baseline Weight (kg) Height (m)**	**Baseline BMI (kg/m^2)**	**NART Error Score (points)**
xxxxxxxxxx/xx/x/x/x	DDMMMYYYY	Xxxxxxxxxxx	xxx.x<br>xxx.x	xx.x	xx

Note: W = white; B = black; P = native Hawaiian or other Pacific islander; A = Asian; I = American Indian or Alaska native; H = Hispanic or Latino; N = not Hispanic or Latino; M = male; F = female.
Source: Dataset: [NAME], Program: xxxxxx.sas, Output: xxxx.rtf, Generated on: DDMONYYYY HH:MM
Page x of y

Programming Note: Repeat listing for ITT Pilot Stage (N=XXX) Placebo/GDNF (N=XXX), then ITT Primary Stage (N=XXX) GDNF/GDNF (N=XXX) and Placebo/GDNF (N=XXX). Sort by stage, treatment group, and subject ID. Include all ITT Overall subjects.

Listing 17.2.1.4.2 Parkinson's Disease History at Screening in Study 2553 - ITT Overall Population

ITT Pilot Stage (N=XXX)

GDNF/GDNF (N=XXX)							
**Subject ID Age/Race/Ethnicity/ Sex**	**Date of First PD Symptom**	**Date of PD Diagnosis**	**Hoehn and Yahr Stage in OFF State**	**UPDRS Motor Score (Part III) OFF State**	**UPDRS Motor Score (Part III) ON State**	**OFF Time per Day (hours)**	**Response to Levodopa[a] (%)**
xxxxxxxxxx/xx/x/x/x	DDMMMYYYY	DDMMMYYYY	x	xx	xx	xx	xx.x

[a] Percentage improvement in screening UPDRS motor score (part III) following a levodopa challenge.

Note: W = white; B = black; P = native Hawaiian or other Pacific islander; A = Asian; I = American Indian or Alaska native; H = Hispanic or Latino; N = not Hispanic or Latino; M = male; F = female.

Source: Dataset: [NAME], Program: xxxxxx.sas, Output: xxxx.rtf, Generated on: DDMONYYYY HH:MM

Page x of y

Programming Note: Repeat listing for ITT Pilot Stage (N=XXX) Placebo/GDNF (N=XXX), then ITT Primary Stage (N=XXX) GDNF/GDNF (N=XXX) and Placebo/GDNF (N=XXX). Sort by stage, treatment group, and subject ID. Include all ITT Overall subjects.

Listing 17.2.1.5 Concomitant Medications – ITT Overall Population

ITT Pilot Stage (N=XXX)						
GDNF/GDNF (N=XXX)						
**Subject ID Age/Race/Ethnicity/ Sex**	**ATC Class Coded Medication Name Verbatim Medication Name**	**Start Date (Study Day)/ Stop Date (Study Day) or Ongoing**	**Dose per Frequency**	**Unit**	**Frequency**	**Indication**
xxxxxxxxxx/xx/x/x/x	xxxxxxxxxxxxxxxxxxx<br>xxxxxxxxxxxxxxxxxxx<br>xxxxxxxxxxxxxx	DDMONYYYY (xxx)/<br>DDMONYYYY (xxx)	xxx	xxx	xxx	xxxxxxxxxxxx

Note: Medications are coded using WHODRUG DDE version MONYYYY. W = white; B = black; P = native Hawaiian or other Pacific islander; A = Asian; I = American Indian or Alaska native; H = Hispanic or Latino; N = not Hispanic or Latino; M = male; F = female.
Source: Dataset: [NAME], Program: xxxxxx.sas, Output: xxxx.rtf, Generated on: DDMONYYYY HH:MM
Page x of y

Programming Note: Repeat listing for ITT Pilot Stage (N=XXX) Placebo/GDNF (N=XXX), then ITT Primary Stage (N=XXX) GDNF/GDNF (N=XXX) and Placebo/GDNF (N=XXX). Sort by stage, treatment group, subject ID and start date. Include all ITT Overall subjects with data.

Listing 17.2.1.6 Catheter Trajectory - ITT Overall Population

ITT Pilot Stage (N=XXX)			
GDNF/GDNF (N=XXX)			
**Subject ID Age/Race/Ethnicity/ Sex**	**Surgery**	**Date of Surgery (Study Day)**	**Catheter Placement**
xxxxxxxxxx/xx/x/x/x	Respositioning Surgery x	DDMONYYYY (xxx)	Vertical/ Horizontal: Anterior-Posterior/ Horizontal: Posterior-Anterior

Note: W = white; B = black; P = native Hawaiian or other Pacific islander; A = Asian; I = American Indian or Alaska native; H = Hispanic or Latino; N = not Hispanic or Latino; M = male; F = female.
Source: Dataset: [NAME], Program: xxxxxx.sas, Output: xxxx.rtf, Generated on: DDMONYYYY HH:MM
Page x of y

Programming Note: Repeat listing for ITT Pilot Stage (N=XXX) Placebo/GDNF (N=XXX), then ITT Primary Stage (N=XXX) GDNF/GDNF (N=XXX) and Placebo/GDNF (N=XXX). Sort by stage, treatment group, subject ID and date of surgery. Include all ITT Overall subjects with data.

Listing 17.2.1.7 Catheter Positioning Accuracy by Surgery - ITT Overall Population

ITT Pilot Stage (N=XXX)

GDNF/GDNF (N=XXX)

Subject Age/Race/ Ethnicity/ Sex	Surgery	Date and Time	Distance Between Planned Target and Actual Target (mm) Catheter 1	Catheter 2	Catheter 3	Catheter 4	Mean Across All Catheters	Catheter Positioning Satisfactory	Hemorrhage
xxxxxxxxxx/xx /x/x/x	Repositioning Surgery #1	DDMONYYYY HHMM	xx.x	xx.x	xx.x	xx.x	xx.x	Satisfactory<br>Required Repositioning<br>Other: xxxxxxx	No Hemorrhage Detected<br>Minor Hemorrhage without Clinical Signs<br>Minor Hemorrhage with Clinical Signs<br>Major Hemorrhage
		DDMONYYYY HHMM	xx.x	xx.x	xx.x	xx.x	xx.x		

Note: W = white; B = black heritage; P = native Hawaiian or other Pacific islander; A = Asian; I = American Indian or Alaska native; H = Hispanic or Latino; N = not Hispanic or Latino; M = male; F = female.
Source: Dataset: [NAME], Program: xxxxxx.sas, Output: xxxx.rtf, Generated on: DDMONYYYY HH:MM
Page x of y

Programming Note: Repeat listing for ITT Pilot Stage (N=XXX) Placebo/GDNF (N=XXX), then ITT Primary Stage (N=XXX) GDNF/GDNF (N=XXX) and Placebo/GDNF (N=XXX). Sort by stage, treatment group, and subject ID then surgery. Include all ITT Overall subjects with data. Surgeries are Initial Surgery, Repositioning surgery #1, and so on. Abbreviate as necessary.

Listing 17.2.1.8 Test Infusion Data by Visit - ITT Overall Population

ITT Pilot Stage (N=XXX)

GDNF/GDNF (N=XXX)

Subject ID<br>Age/Race/Ethnicity/<br>Sex	Visit	Date	Time First Start<br>Time Last Stop	Catheter 1	Catheter 2	Catheter 3	Catheter 4
xxxxxxxxxx/xx/x/x/x	Week 40/e0	DDMONYYYY	HH:MM<br>HH:MM	Standard	Standard	Standard	Not Used
		DDMONYYYY	HH:MM<br>HH:MM	Non-standard	Standard	Standard	Not Used

Note: W = white; B = black; P = native Hawaiian or other Pacific islander; A = Asian; I = American Indian or Alaska native; H = Hispanic or Latino; N = not Hispanic or Latino; M = male; F = female.
Note: Standard regime is a linear ramp up infusion rate of 3-5 µL/min with 400 µL total volume.
Source: Dataset: [NAME], Program: xxxxxx.sas, Output: xxxx.rtf, Generated on: DDMONYYYY HH:MM
Page x of y

Programming Note: Repeat listing for ITT Pilot Stage (N=XXX) Placebo/GDNF (N=XXX), then ITT Primary Stage (N=XXX) GDNF/GDNF (N=XXX) and Placebo/GDNF (N=XXX). Sort by stage, treatment group, subject ID, visit, and date of infusion. Include all ITT Overall subjects with data. Include all test infusions , including repeat or unscheduled test infusions, from all extension parts of Study 2797. For all listings by visit, use actual visit labels and not “screening” or “baseline.”

Listing 17.2.1.9 Test Infusion Catheter Interruptions/Early Terminations by Visit - ITT Overall Population

ITT Pilot Stage (N=XXX)

GDNF/GDNF (N=XXX)

Subject ID Age/Race/Ethnicity/ Sex	Visit	Date	Time First Start Time Last Stop	Catheter No. Interrupted/ Terminated Early	Time Infusion Stopped	Time Infusion Restarted	Reason for Stop
xxxxxxxxxx/xx/x/x/x	Week 40/e0	DDMONYYYY	HH:MM HH:MM	1	HH:MM	HH:MM	xxxxxx
				2	HH:MM	HH:MM	xxxxxx
				3	HH:MM	HH:MM	xxxxxx
				4	HH:MM	HH:MM	xxxxxx
		DDMONYYYY	HH:MM HH:MM	1	HH:MM	HH:MM	xxxxxx
				2	HH:MM	HH:MM	xxxxxx
				3	HH:MM	HH:MM	xxxxxx
				4	HH:MM	HH:MM	xxxxxx

Note: W = white; B = black; P = native Hawaiian or other Pacific islander; A = Asian; I = American Indian or Alaska native; H = Hispanic or Latino; N = not Hispanic or Latino; M = male; F = female.
Source: Dataset: [NAME], Program: xxxxxx.sas, Output: xxxx.rtf, Generated on: DDMONYYYY HH:MM
Page x of y

Programming Note: Repeat listing for ITT Pilot Stage (N=XXX) Placebo/GDNF (N=XXX), then ITT Primary Stage (N=XXX) GDNF/GDNF (N=XXX) and Placebo/GDNF (N=XXX). Sort by stage, treatment group, subject ID, visit, and date of infusion. Include all ITT Overall subjects with data. Include all test infusions , including repeat or unscheduled test infusions, from all extension parts of Study 2797. For all listings by visit, use actual visit labels and not "screening" or "baseline."

17.2.2 EFFICACY LISTINGS

Listing 17.2.2.1 OFF and ON state UPDRS Scores by Visit - ITT Overall Population

ITT Pilot Stage (N=XXX)

GDNF/GDNF (N=XXX)

Subject ID Age/Race/ Ethnicity/ Sex	Parameter (points)	Visit	Date and Time	Result	Change From Baseline
xxxxxxxxxx/xx /x/x/x	OFF state UPDRS motor score (part III)	Week 0	DDMONYYYY HHMM	xxx	
		Week 40/e0	DDMONYYYY HHMM	xxx	xxx.x
		Week 48/e8	DDMONYYYY HHMM	xxx	xxx.x
		…	…	…	…
	ON state UPDRS motor score (part III)	Week 0	DDMONYYYY HHMM	xxx	
		Week 40/e0	DDMONYYYY HHMM	xxx	xxx.x
		Week 48/e8	DDMONYYYY HHMM	xxx	xxx.x
		…	…	…	…
	OFF state UPDRS ADL score (part II)	Week 0	DDMONYYYY HHMM	xxx	
		Week 40/e0	DDMONYYYY HHMM	xxx	xxx.x
		Week 48/e8	DDMONYYYY HHMM	xxx	xxx.x
		…	…	…	…
	ON state UPDRS ADL score (part II)	Week 0	DDMONYYYY HHMM	xxx	
		Week 40/e0	DDMONYYYY HHMM	xxx	xxx.x
		Week 48/e8	DDMONYYYY HHMM	xxx	xxx.x
	etc.	…	…	…	…

Note: Lower scores represent better functioning. OFF and ON state UPDRS total score are sums of motor score (part III) and ADL score (part II). For subject 45, items 22, 27, 28, 29 and 30 are excluded from the motor score (part III). Subject 45 had observed OFF state UPDRS motor score (part III) values of XX, XX, … at visits X, X, … respectively. Subject 45 had observed ON state UPDRS motor score (part III) values of XX, XX, … at visits X, X, … respectively. The observed OFF state and ON state UPDRS motor scores at Screening of subject 45 are used in the calculation of summary statistics in Tables 16.1.4.1 and 16.1.4.2. (Parkinson's Disease History at Screening from Study 2553). W = white; B = black; P = native Hawaiian or other Pacific islander; A = Asian; I = American Indian or Alaska native; H = Hispanic or Latino; N = not Hispanic or Latino; M = male; F = female.
Source: Dataset: [NAME], Program: xxxxxx.sas, Output: xxxx.rtf, Generated on: DDMONYYYY HH:MM
Page x of y

Programming Note: Repeat listing for ITT Pilot Stage (N=XXX) Placebo/GDNF (N=XXX), then ITT Primary Stage (N=XXX) GDNF/GDNF (N=XXX) and Placebo/GDNF (N=XXX). Sort by stage, treatment group, and subject ID then parameter, OFF/ON state, and visit. Include all ITT Overall subjects with data.

Parameters are OFF and ON state UPDRS Motor score (part III), OFF and ON state UPDRS Activities of daily living score (part II), OFF and ON state UPDRS total score (sum of motor + ADL scores), Mentation, behavior, and mood score (part I), and Complications of therapy score (part IV). Visits are Screening Visit 2, Weeks 0, 40/e0, 48/e8, 56/e16, 64/e24, 72/e32 and 80/e40 and all Pilot and Supplemental Extension visits. Baseline is Week 0 for all parameters. Abbreviate as necessary. Do not include imputed values. For all listings by visit, use actual visit labels and not “screening” or “baseline.”

Listing 17.2.2.2 PD Motor Fluctuation Diary Ratings by Visit - ITT Overall Population

ITT Pilot Stage (N=XXX)

GDNF/GDNF (N=XXX)

Subject ID Age/Race/ Ethnicity/ Sex	Parameter (hours)	Visit	Date	Result	Change From Baseline
xxxxxxxxxx/xx /x/x/x	OFF time per day	Week 0	DDMONYYYY	xx	
		Week 40/e0	DDMONYYYY	xx	xx.x
		Week 48/e8	DDMONYYYY	xx	xx.x
		…	…	…	…
	Total good-quality ON time per day	Week 0	DDMONYYYY	xx	
		Week 40/e0	DDMONYYYY	xx	xx.x
		Week 48/e8	DDMONYYYY	xx	xx.x
		…	…	…	…
	ON time per day without dyskinesias	Week 0	DDMONYYYY	xx	
		Week 40/e0	DDMONYYYY	xx	xx.x
		Week 48/e8	DDMONYYYY	xx	xx.x
		…	…	…	…
	ON time per day with non-troublesome dyskinesias	Week 0	DDMONYYYY	xx	
		Week 40/e0	DDMONYYYY	xx	xx.x
		Week 48/e8	DDMONYYYY	xx	xx.x
		…	…	…	…
	ON time per day with troublesome dyskinesias	Week 0	DDMONYYYY	xx	
		Week 40/e0	DDMONYYYY	xx	xx.x
		Week 48/e8	DDMONYYYY	xx	xx.x
	etc.	…	…	…	…

Note: W = white; B = black; P = native Hawaiian or other Pacific islander; A = Asian; I = American Indian or Alaska native; H = Hispanic or Latino; N = not Hispanic or Latino; M = male; F = female.
Note: Total good-quality ON time per day is defined as the sum of ON time per day without dyskinesias + ON time per day with non-troublesome dyskinesias.
Source: Dataset: [NAME], Program: xxxxxx.sas, Output: xxxx.rtf, Generated on: DDMONYYYY HH:MM
Page x of y

Programming Note: Repeat listing for ITT Pilot Stage (N=XXX) Placebo/GDNF (N=XXX), then ITT Primary Stage (N=XXX) GDNF/GDNF (N=XXX) and Placebo/GDNF (N=XXX). Sort by stage, treatment group, and subject ID then parameter, and visit. Include all ITT Overall subjects with data. PD diary parameters are OFF time per day, total good-quality ON time per day (sum of ON time per day without dyskinesias + ON time per day with non-troublesome dyskinesias), ON time per day without dyskinesias, ON time per day with non-troublesome dyskinesias, and ON time per day with troublesome dyskinesias. Visits are Screening Visit 2, Weeks 0, 40/e0, 48/e8, 56/e16, 64/e24, 72/e32 and 80/e40 and all Pilot and Supplemental Extension visits. Baseline is Week 0. Abbreviate as necessary. For all listings by visit, use actual visit labels and not "screening" or "baseline."

Listing 17.2.2.3 Treatment Response at Week 40/e0 and Week 80/e40 - ITT Overall Population

ITT Pilot Stage (N=XXX)

GDNF/GDNF (N=XXX)

Subject ID/Age/Race/ Ethnicity/Sex	Criteria	Treatment Responder
xxxxxxxxxx/xx/x/x/x	Decrease from baseline to Week 40/e0 by ≥10 points in OFF state UPDRS motor score (part III)	Yes/No
	Increase from baseline to Week 40/e0 by ≥1 hour in total good quality ON time per day (ON without dyskinesias or ON with non troublesome dyskinesias)	Yes/No
	Decrease from baseline to Week 40/e0 by ≥10 points in OFF state UPDRS motor score (part III) and increase from baseline to Week 40/e0 by ≥1 hour in total good quality ON time per day (ON without dyskinesias or ON with non troublesome dyskinesias)	Yes/No
	Decrease from baseline to Week 80/e40 by ≥10 points in OFF state UPDRS motor score (part III)	Yes/No
	Increase from baseline to Week 80/e40 by ≥1 hour in total good quality ON time per day (ON without dyskinesias or ON with non troublesome dyskinesias)	Yes/No
	Decrease from baseline to Week 80/e40 by ≥10 points in OFF state UPDRS motor score (part III) and increase from baseline to Week 80/e40 by ≥1 hour in total good quality ON time per day (ON without dyskinesias or ON with non troublesome dyskinesias)	Yes/No
xxxxxxxxxx/xx/x/x/x	Decrease from baseline to Week 40/e0 by ≥10 points in OFF state UPDRS motor score (part III)	Yes/No
	Increase from baseline to Week 40/e0 by ≥1 hour in total good quality ON time per day (ON without dyskinesias or ON with non troublesome dyskinesias)	Yes/No
	Decrease from baseline to Week 40/e0 by ≥10 points in OFF state UPDRS motor score (part III) and increase from baseline to Week 40/e0 by ≥1 hour in total good quality ON time per day (ON without dyskinesias or ON with non troublesome dyskinesias)	
	Decrease from baseline to Week 80/e40 by ≥10 points in OFF state UPDRS motor score (part III)	
	Increase from baseline to Week 80/e40 by ≥1 hour in total good quality ON time per day (ON without dyskinesias or ON with non troublesome dyskinesias)	
	Decrease from baseline to Week 80/e40 by ≥10 points in OFF state UPDRS motor score (part III) and increase from baseline to Week 80/e40 by ≥1 hour in total good quality ON time per day (ON without dyskinesias or ON with non troublesome dyskinesias)	

Note: W = white; B = black; P = native Hawaiian or other Pacific islander; A = Asian; I = American Indian or Alaska native; H = Hispanic or Latino; N = not Hispanic or Latino; M = male; F = female.
Source: Dataset: [NAME], Program: xxxxxx.sas, Output: xxxx.rtf, Generated on: DDMONYYYY HH:MM
Page x of y

Programming Note: Repeat listing for ITT Pilot Stage (N=XXX) Placebo/GDNF (N=XXX), then ITT Primary Stage (N=XXX) GDNF/GDNF (N=XXX) and Placebo/GDNF (N=XXX). Sort by stage, treatment group, and subject ID then visit. Include all ITT Overall subjects with data. For all listings by visit, use actual visit labels and not "screening" or "baseline."

Listing 17.2.2.4.1 OFF and ON State Timed Walking Test by Visit - ITT Overall Population

ITT Pilot Stage (N=XXX)

GDNF/GDNF (N=XXX)

Subject Age/Race/ Ethnicity/ Sex	Parameter (seconds)	Visit	Date and Time	Result (mean of replicates)	Change From Baseline
xxxxxxxxxx/xx /x/x/x	OFF state timed walking test	Screening Visit 2	DDMONYYYY HHMM	xx	
		Week 0	DDMONYYYY HHMM	xx	
		Week 40/e0	DDMONYYYY HHMM	xx	xx.x
		…	…	…	…
	ON state timed walking test	Screening Visit 2	DDMONYYYY HHMM	xx	
		Week 0	DDMONYYYY HHMM	xx	
		Week 40/e0	DDMONYYYY HHMM	xx	xx.x
		…	…	…	…

Note: Shorter times represent better function. Two trials per state per visit are averaged for analysis. If only the first trial is completed for a particular state, then the second trial result will be imputed with the worst non-missing second trial result from all subjects for that particular state during the study. If both trials are missing, then the endpoint is not reported for that visit. W = white; B = black; P = native Hawaiian or other Pacific islander; A = Asian; I = American Indian or Alaska native; H = Hispanic or Latino; N = not Hispanic or Latino; M = male; F = female.

Source: Dataset: [NAME], Program: xxxxxx.sas, Output: xxxx.rtf, Generated on: DDMONYYYY HH:MM

Page x of y

Programming Note: Same format as Listing 17.2.2.1. Repeat listing for ITT Pilot Stage (N=XXX) Placebo/GDNF (N=XXX), then ITT Primary Stage (N=XXX) GDNF/GDNF (N=XXX) and Placebo/GDNF (N=XXX). Sort by stage, treatment group, and subject ID then parameter, OFF/ON state, and visit. Include all ITT Overall subjects with data. Parameters are OFF state timed walking test and ON state timed walking test. Visits are Screening Visit 2, Weeks 0, 40/e0, 48/e8, 56/e16, 64/e24, 72/e32 and 80/e40 and all Pilot and Supplemental Extension visits. Baseline is Week 0. Abbreviate as necessary. For all listings by visit, use actual visit labels and not "screening" or "baseline."

Listing 17.2.2.4.2 OFF and ON State Timed Tapping Test by Visit - ITT Overall Population

ITT Pilot Stage (N=XXX)

GDNF/GDNF (N=XXX)

Subject Age/Race/ Ethnicity/ Sex	Parameter (taps)	Visit	Date and Time	Result (mean of replicates)	Change From Baseline
xxxxxxxxxx/xx /x/x/x	OFF state timed tapping test (left hand)	Screening Visit 2	DDMONYYYY HHMM	xx	
		Week 0	DDMONYYYY HHMM	xx	
		Week 40/e0	DDMONYYYY HHMM	xx	xx.x
		…	…	…	…
	OFF state timed tapping test (right hand)	Screening Visit 2	DDMONYYYY HHMM	xx	
		Week 0	DDMONYYYY HHMM	xx	
		Week 40/e0	DDMONYYYY HHMM	xx	xx.x
		…	…	…	…
	OFF state timed tapping test (both hands)	Screening Visit 2	DDMONYYYY HHMM	xx	
		Week 0	DDMONYYYY HHMM	xx	
		Week 40/e0	DDMONYYYY HHMM	xx	xx.x
		…	…	…	…

Note: More taps represent better function. The four separate trials per state for each visit are averaged for analysis. If any trial is not completed for a particular state, then the trial result will be imputed with the worst non-missing applicable trial result (for example, first left hand trial) from all subjects for that particular state during the study. If all four trials are missing, then the endpoint is not reported for that visit. W = white; B = black; P = native Hawaiian or other Pacific islander; A = Asian; I = American Indian or Alaska native; H = Hispanic or Latino; N = not Hispanic or Latino; M = male; F = female.
Source: Dataset: [NAME], Program: xxxxxx.sas, Output: xxxx.rtf, Generated on: DDMONYYYY HH:MM
Page x of y

Programming Note: Same format as Listing 17.2.2.1. Repeat listing for ITT Pilot Stage (N=XXX) Placebo/GDNF (N=XXX), then ITT Primary Stage (N=XXX) GDNF/GDNF (N=XXX) and Placebo/GDNF (N=XXX). Sort by stage, treatment group, and subject ID then parameter, OFF/ON state, and visit. Include all ITT Overall subjects with data. Parameters are OFF state timed tapping test (left hand), OFF state timed tapping test (right hand), OFF state timed tapping test (both hands), ON state timed tapping test (left hand), ON state timed tapping test (right hand), and ON state timed tapping test (both hands). Visits are Screening Visit 2, Weeks 0, 40/e0, 48/e8, 56/e16, 64/e24, 72/e32 and 80/e40 and all Pilot and Supplemental Extension visits. Baseline is Week 0. Abbreviate as necessary. For all listings by visit, use actual visit labels and not "screening" or "baseline."

Listing 17.2.2.5 NMSS Scores by Visit - ITT Overall Population

Programming Note: Repeat listing as above (17.2.2.x). Repeat listing for ITT Pilot Stage (N=XXX) Placebo/GDNF *(N=XXX), then ITT Primary Stage (N=XXX) GDNF*/GDNF *(N=XXX) and Placebo*/GDNF *(N=XXX). Sort by stage, treatment group, and subject ID then parameter, and visit. Include all ITT Overall subjects with data. NMSS parameters are Cardiovascular including falls domain, Sleep/fatigue domain, Mood/cognition domain, Perceptual problems/hallucinations domain; Attention/memory domain, Gastrointestinal tract domain, Urinary domain, Sexual function domain, Miscellaneous domain, and NMSS total score. Visits are Screening Visit 2, Weeks* 40/e0, 52/e12, 64/e24 and 80/e40 and all Pilot Extension visits. *Abbreviate as necessary. Add footnote "Note: The NMSS is a 30-item interview-based scale that rates non-motor symptoms that occurred in the preceding month in 9 domains. Each item is rated from 0 (none) to 3 (severe) for severity and from 1 (rarely) to 4 (very frequent) for frequency. The maximum score for an individual item is 12. The higher the score, the worse the subject's condition. The maximum NMSS total score is 360." Do not list imputed values. For all listings by visit, use actual visit labels and not "screening" or "baseline."*

Listing 17.2.2.6 PDQ-39 Scores by Visit - ITT Overall Population

Programming Note: Repeat listing as above (17.2.2.x)*. Repeat listing for ITT Pilot Stage (N=XXX) Placebo/GDNF (N=XXX), then ITT Primary Stage (N=XXX) GDNF/GDNF (N=XXX) and Placebo/GDNF (N=XXX). Sort by stage, treatment group, and subject ID then parameter, and visit. Include all ITT Overall subjects with data. PDQ-39 parameters are Mobility dimension, ADL dimension, Emotional well-being dimension, Stigma dimension, Social support dimension, Cognitions dimension, Communication dimension, Bodily discomfort dimension, and the Single index (total) PDQ-39 score. Visits are Screening Visit 2, Week 40/e0, and Week 80/e40. Abbreviate as necessary. Add note: The PDQ-39 is a self-administered 39-item PD-specific scale that rates symptoms that occurred in the preceding month in 8 dimensions. Each item is rated from 0 (never) to 4 (always) for frequency. The score for each dimension is the average of responses in the dimension, weighted by the number of questions and multiplied by 100. PDQ-39 dimension and total scores range from 0 to 100 (0 = no problem, 100 = problem as worse as possible). The higher the score, the worse the subject's condition. For all listings by visit, use actual visit labels and not "screening" or "baseline."*

Listing 17.2.2.7 EQ-5D Scores by Visit - ITT Overall Population

Programming Note: Repeat listing as above (17.2.2.x)*. Repeat listing for ITT Pilot Stage (N=XXX) Placebo/GDNF (N=XXX), then ITT Primary Stage (N=XXX) GDNF/GDNF (N=XXX) and Placebo/GDNF (N=XXX). Sort by stage, treatment group, and subject ID then parameter, and visit. Include all ITT Overall subjects with data. Parameters are the individual item scores and the visual analog scale score. Visits are Screening Visit 2, Week 40/e0, and Week 80/e40. Abbreviate as necessary. Add note: The EQ-5D is a self-administered 5-item scale with three possible answers for each item (1=no problem, 2=moderate problem, 3=severe problem), and a visual analog scale ranging from 0 to 100, where 0 indicates worst health and 100 indicates best health. Change from baseline only applies to the visual analog scale. For all listings by visit, use actual visit labels and not "screening" or "baseline."*

Listing 17.2.2.8 SNAQ Scores by Visit - ITT Overall Population

Programming Note: Repeat listing as above (17.2.2.x)*. Repeat listing for ITT Pilot Stage (N=XXX) Placebo/GDNF (N=XXX), then ITT Primary Stage (N=XXX) GDNF/GDNF (N=XXX) and Placebo/GDNF (N=XXX). Sort by stage, treatment group, and subject ID then parameter, and visit. Include all ITT Overall subjects with data. Parameters are total SNAQ score. Visits are Screening Visit 2, Week 40/e0, and Week 80/e40. Abbreviate as necessary. Add note: The SNAQ is a self-administered 4-question instrument with total scores ranging from 4 to 20 (4=poor appetite, 20=good appetite). For all listings by visit, use actual visit labels and not "screening" or "baseline."*

Listing 17.2.2.9.1 Levodopa and Levodopa Equivalent Medication Actual Total Daily Doses - ITT Overall Population

ITT Pilot Stage (N=XXX)

GDNF/GDNF (N=XXX)

**Subject ID Age/Race/Ethnicity/ Sex**	**Category**	**Medication**	**Actual Total Daily Dose (mg) Baseline (Week 0)**	**Week 40/e0**	**Week 80/e40**
xxxxxxxxxx/xx /x/x/x	Levodopa preparations	Immediate release preparations without COMT inhibition		xx	xx
		Immediate release preparations with entacapone			
		Immediate release preparations with tolcapone			
		Controlled release preparations			
		Levodopa/carbidopa (Duodopa)			
	Dopamine agonists	Ropinirole immediate release			
		Ropinirole long acting			
		Pramipexole immediate release (base)			
		Pramipexole immediate release (salt)			
		Pramipexole long acting (base)			
		Pramipexole long acting (salt)			
		Cabergoline			
		Rotigotine			
		Piribedil			
		Apomorphine			
		Bromocriptine			
		Pergolide			
		Lisuride			
		Dihydroergocryptine (DHEC)			
	COMT inhibitors	Entacapone			
		Tolcapone			
	MAO-B inhibitors	Selegiline oral			
		Selegiline sublingual			
		Rasagiline			
	Other	Amantadine			
		Other: xxxxxxxx			

Note: W = white; B = black; P = native Hawaiian or other Pacific islander; A = Asian; I = American Indian or Alaska native; H = Hispanic or Latino; N = not Hispanic or Latino; M = male; F = female.
Source: Dataset: [NAME], Program: xxxxxx.sas, Output: xxxx.rtf, Generated on: DDMONYYYY HH:MM
Page x of y

Programming Note: Repeat listing for ITT Pilot Stage (N=XXX) Placebo/GDNF (N=XXX), then ITT Primary Stage (N=XXX) GDNF/GDNF (N=XXX) and

Placebo/GDNF (N=XXX). Sort by stage, treatment group, subject ID and category. Include all ITT Overall subjects with data. ***Actual dose means as documented on the CRF (ie, unconverted). Include only those categories and medications with data.***

Listing 17.2.2.9.2 Effective Levodopa and Levodopa Equivalent Medication Total Daily Doses - ITT Overall Population

ITT Pilot Stage (N=XXX)

GDNF/GDNF (N=XXX)

		Total Daily Dose (mg)		
**Subject ID Age/Race/Ethnicity/ Sex**	**Medication**	**Baseline (Week 0)**	**Week 40/e0**	**Week 80/e40**
xxxxxxxxxx/xx /x/x/x	Effective Levodopa		xx	xx
	Levodopa Equivalent		xx	xx
xxxxxxxxxx/xx /x/x/x	Effective Levodopa		xx	xx
	Levodopa Equivalent		xx	xx

Note: W = white; B = black; P = native Hawaiian or other Pacific islander; A = Asian; I = American Indian or Alaska native; H = Hispanic or Latino; N = not Hispanic or Latino; M = male; F = female.

Note: Specific conversion factors are used in order to characterize the subject's effective levodopa dose. Immediate release preparations taken without concomitant catechol-O-methyl transferase (COMT) inhibitors do not require conversion. The daily doses of immediate release preparations taken with COMT inhibitors and of controlled release preparations are multiplied by the corresponding conversion factors. The total daily levodopa dose is then calculated by adding together the converted daily doses of all individual levodopa-containing preparations. Levodopa equivalent dose is calculated by multiplying each PD medication dose by a specific conversion factor indicating the drug's relative potency with respect to immediate release levodopa unaccompanied by COMT inhibitors. The total daily levodopa equivalent dose is calculated by adding together the daily levodopa equivalent doses of all individual PD medications. COMT inhibitor doses are not included in either of these calculations.

Source: Dataset: [NAME], Program: xxxxxx.sas, Output: xxxx.rtf, Generated on: DDMONYYYY HH:MM
Page x of y

Programming Note: Repeat listing for ITT Pilot Stage (N=XXX) Placebo/GDNF (N=XXX), then ITT Primary Stage (N=XXX) GDNF/GDNF (N=XXX) and Placebo/GDNF (N=XXX). Sort by stage, treatment group, subject ID and category. Include all ITT Overall subjects with data. ***Total daily doses are the converted doses.***

17.2.3 Imaging Listings

Listing 17.2.3.1 Volume of Distribution, Volume of Interest Coverage, and Total Putamenal Coverage as Determined by Contrast-Enhanced T1-Weighted MRI by Visit - ITT Overall Population

Programming Note: Repeat listing as above (17.2.2.x)*. Sort by stage, treatment group and subject ID then parameter and visit. Include all ITT Overall subjects with data. Parameters are volume of distribution (mL, left and right), VOI (mL, left and right), VOI coverage (mL, left and right; %, left and right), putamenal volume of distribution (mL, left and right), total volume of putamen (mL, left and right), total putamenal coverage (%, left and right). Visits are last test infusion at the end of the healing phase, Week 40/e0, and Week 80/e40. Abbreviate as necessary. For all listings by visit, use actual visit labels and not "screening" or "baseline."*

17.2.4 Safety Listings

Listing 17.2.4.1.1 Exposure to Study Medication - Safety Overall Population

Safety Pilot Stage (N=XXX)		
GDNF/GDNF (N=XXX)		
**Subject ID Age/Race/Ethnicity/ Sex**	**No. of Infusions of Study Medication**	**Total Exposure (mg)**
xxxxxxxxxx/xx/x/x/x	xx	xxxx.x

Note: W = white; B = black; P = native Hawaiian or other Pacific islander; A = Asian; I = American Indian or Alaska native; H = Hispanic or Latino; N = not Hispanic or Latino; M = male; F = female.
Source: Dataset: [NAME], Program: xxxxxx.sas, Output: xxxx.rtf, Generated on: DDMONYYYY HH:MM
Page x of y

Programming Note: Repeat listing for Safety Pilot Stage (N=XXX) Placebo/GDNF (N=XXX), then Safety Primary Stage (N=XXX) GDNF/GDNF (N=XXX) and Placebo/GDNF (N=XXX). Sort by stage, treatment group, and subject ID. Include all Safety Overall subjects. Include only infusions from Study 2797.

Listing 17.2.4.1.2 Study Medication Infusion Data by Visit - Safety Overall Population

Safety Pilot Stage (N=XXX) GDNF/GDNF (N=XXX)							
**Subject ID Age/Race/Ethnicity/ Sex**	**Visit**	**Date**	**Time First Start Time Last Stop**	**Catheter 1**	**Catheter 2**	**Catheter 3**	**Catheter 4**
xxxxxxxxxx/xx/x/x/x	Week x	DDMONYYYY	HH:MM HH:MM	Standard	Standard	Standard	Not Used
	Week x	DDMONYYYY	HH:MM HH:MM	Non-standard	Standard	Standard	Not Used

Note: W = white; B = black; P = native Hawaiian or other Pacific islander; A = Asian; I = American Indian or Alaska native; H = Hispanic or Latino; N = not Hispanic or Latino; M = male; F = female.
Note: Standard regime is a linear ramp up infusion rate of 3-5 µL/min with 400 µL total volume.

Source: Dataset: [NAME], Program: xxxxxx.sas, Output: xxxx.rtf, Generated on: DDMONYYYY HH:MM
Page x of y

Programming Note: Repeat listing for Safety Pilot Stage (N=XXX) Placebo/GDNF (N=XXX), then Safety Primary Stage (N=XXX) GDNF/GDNF (N=XXX) and Placebo/GDNF (N=XXX). Sort by stage, treatment group, and subject ID then visit for all visits in Study 2797. Include all Safety Overall subjects. For all listings by visit, use actual visit labels and not "screening" or "baseline."

Listing 17.2.4.1.3 Study Medication Infusion Catheter Interruptions/Early Terminations by Visit - Safety Overall Population

Safety Pilot Stage (N=XXX)

GDNF/GDNF (N=XXX)

Subject ID Age/Race/Ethnicity/ Sex	Visit	Date	Time First Start Time Last Stop	Catheter No. Interrupted/ Terminated Early	Time Infusion Stopped	Time Infusion Restarted	Reason for Stop
xxxxxxxxxx/xx/x/x/x	Week x	DDMONYYYY	HH:MM	1	HH:MM	HH:MM	
			HH:MM	2	HH:MM	HH:MM	
				3	HH:MM	HH:MM	
				4	HH:MM	HH:MM	xxxxxx
	Week x	DDMONYYYY	HH:MM	1	HH:MM	HH:MM	
			HH:MM	2	HH:MM	HH:MM	
				3	HH:MM	HH:MM	
				4	HH:MM	HH:MM	xxxxxx

Note: W = white; B = black; P = native Hawaiian or other Pacific islander; A = Asian; I = American Indian or Alaska native; H = Hispanic or Latino; N = not Hispanic or Latino; M = male; F = female.
Source: Dataset: [NAME], Program: xxxxxx.sas, Output: xxxx.rtf, Generated on: DDMONYYYY HH:MM
Page x of y

Programming Note: Repeat listing for Safety Pilot Stage (N=XXX) Placebo/GDNF (N=XXX), then Safety Primary Stage (N=XXX) GDNF/GDNF (N=XXX) and Placebo/GDNF (N=XXX). Sort by stage, treatment group, and subject ID then visit for all visits in Study 2797. Include all Safety Overall subjects. For all listings by visit, use actual visit labels and not “screening” or “baseline.”

Listing 17.2.4.2 Adverse Events - Safety Overall Population

Safety Pilot Stage (N=XXX)									
GDNF/GDNF (N=XXX)									
Subject ID Age/Race/Ethnicity Sex	MedDRA Preferred Term VERBATIM TERM	Start Date and Time (Day)/ Stop Date and Time (Day) or Ongoing	Pre-existing?	SAE	Severity	Related to Study Drug?	Related to Device?	Out-come[a]	Ac-tion[b]
xxxxxxxxxx/xx/x/x/x	xxxxxxxxxxx xxxxxxxxxxx	DDMONYYYY HHMM (xxx)/ DDMONYYYY HHMM (xxx)	No	Yes	Mild	No	Yes	xx	99: xxxxxx

Note: Adverse events are coded using MedDRA version 19.0. W = white; B = black; P = native Hawaiian or other Pacific islander; A = Asian; I = American Indian or Alaska native; H = Hispanic or Latino; N = not Hispanic or Latino; M = male; F = female.

a Outcome: 1 = Recovered/resolved; 2 = Not recovered/not resolved; 3 = Recovered/resolved with sequelae; 4 = Fatal.

b Action: 1 = Current infusion interrupted and restarted; 2 = Current infusion terminated; 3 = Infusion protocol modified; 4 = Infusion schedule suspended and resumed; 5 = Treatment discontinued permanently; 6 = Surgical revision/replacement of extracerebral device parts; 7 = Surgical repositioning/replacement of intracerebral device parts; 99 = Other.

Source: Dataset: [NAME], Program: xxxxxx.sas, Output: xxxx.rtf, Generated on: DDMONYYYY HH:MM

Page x of y

Programming Note: Repeat listing for Safety Pilot Stage (N=XXX) Placebo/GDNF (N=XXX), then Safety Primary Stage (N=XXX) GDNF/GDNF (N=XXX) and Placebo/GDNF (N=XXX). Sort by stage, treatment group, subject ID, start date and time. Include all Safety Overall subjects with data.

Listing 17.2.4.3.1 Adverse Events of Special Interest: Dyskinesias- Safety Overall Population

Programming Note: Repeat AE listing above. Repeat listing for Safety Pilot Stage (N=XXX) Placebo/GDNF (N=XXX), then Safety Primary Stage (N=XXX) GDNF/GDNF (N=XXX) and Placebo/GDNF (N=XXX). Sort by stage, treatment group, subject ID, start date and time. Include all Safety Overall subjects with data.

Listing 17.2.4.3.2 Adverse Events of Special Interest: Falls- Safety Overall Population

Repeat Listing 17.2.4.3.1

Listing 17.2.4.3.3 Adverse Events of Special Interest: Adverse Changes in Mood- Safety Overall Population

Repeat Listing 17.2.4.3.1

Listing 17.2.4.3.4 Adverse Events of Special Interest: Impulsivity- Safety Overall Population

Repeat Listing 17.2.4.3.1

Listing 17.2.4.4 Port Symptoms by Visit - Safety Overall Population

Safety Pilot Stage (N=XXX)

GDNF/GDNF (N=XXX)

Subject ID Age/Race/Ethnicity Sex	Visit	Date Performed	Result
xxxxxxxxxx/xx/x/x/x	Week 40/e0	DDMONYYYY	No skin reaction
	…	….	Redness with slight swelling
			Redness, moistness and moderate swelling with tissue granulation
			Overt infection

Note: W = white; B = black; P = native Hawaiian or other Pacific islander; A = Asian; I = American Indian or Alaska native; H = Hispanic or Latino; N = not Hispanic or Latino; M = male; F = female.
Source: Dataset: [NAME], Program: xxxxxx.sas, Output: xxxx.rtf, Generated on: DDMONYYYY HH:MM
Page x of y

Programming Note: Repeat listing for Safety Pilot Stage (N=XXX) Placebo/GDNF (N=XXX), then Safety Primary Stage (N=XXX) GDNF/GDNF (N=XXX) and Placebo/GDNF (N=XXX). Sort by stage, treatment group, and subject ID then visit for all visits in Study 2797. Include all Safety Overall subjects with data.

Listing 17.2.4.5 Hematology Results by Visit - Safety Overall Population

Safety Pilot Stage (N=XXX)

GDNF/GDNF (N=XXX)

Subject ID Age/Race/Ethnicity/ Sex	Parameter (Unit)	Visit	Sample Date	Result	Change from Baseline	Normal Range	Flag	Clin Sig?
xxxxxxxxxx/xx/x/x/x	xxxxxxxxxxxxx	Week 40/e0	DDMONYYYY	xxx.xx		xxx.xx – xxx.xx	H	No
		Week X	DDMONYYYY	xxx.xx	xx.xx	xxx.xx – xxx.xx	H	No
		…	…	…	…	…	…	…

Note: W = white; B = black; P = native Hawaiian or other Pacific islander; A = Asian; I = American Indian or Alaska native; H = Hispanic or Latino; N = not Hispanic or Latino; M = male; F = female; H = high; L = low.
Source: Dataset: [NAME], Program: xxxxxx.sas, Output: xxxx.rtf, Generated on: DDMONYYYY HH:MM
Page x of y

Programming Note: Repeat listing for Safety Pilot Stage (N=XXX) Placebo/GDNF *(N=XXX), then Safety Primary Stage (N=XXX) GDNF*/GDNF *(N=XXX) and Placebo*/GDNF *(N=XXX). Sort by stage, treatment group, and subject ID then parameter (order as shown in SAP Table 5), and visit for Week 40/e0 and all further visits in Study 2797. Include all Safety Overall subjects with data. For all listings by visit, use actual visit labels and not "screening" or "baseline."*

Listing 17.2.4.6 Serum Chemistry Results by Visit - Safety Overall Population

Programming Note: Repeat listing for serum chemistry parameters. Repeat listing for Safety Pilot Stage (N=XXX) Placebo/GDNF *(N=XXX), then Safety Primary Stage (N=XXX) GDNF*/GDNF *(N=XXX) and Placebo*/GDNF *(N=XXX). Sort by stage, treatment group, and subject ID then parameter (order as shown in SAP Table 5), and visit for Week 40/e0 and all further visits in Study 2797. Include all Safety Overall subjects with data. For all listings by visit, use actual visit labels and not "screening" or "baseline."*

Listing 17.2.4.7 Urinalysis Results by Visit - Safety Overall Population

Programming Note: Repeat listing for urinalysis parameters. Repeat listing for Safety Pilot Stage (N=XXX) Placebo/GDNF *(N=XXX), then Safety Primary Stage (N=XXX) GDNF*/GDNF *(N=XXX) and Placebo*/GDNF *(N=XXX). Sort by stage, treatment group, and subject ID then parameter (order as shown in SAP Table 5), and visit for Week 40/e0 and all further visits in Study 2797. Include all Safety Overall subjects with data. For all listings by visit, use actual visit labels and not "screening" or "baseline."*

Listing 17.2.4.8 Anti-GDNF Serum Antibodies - Safety Overall Population

Safety Pilot Stage (N=XXX)				
GDNF/GDNF (N=XXX)				
**Subject ID Age/Race/Ethnicity/ Sex**	**Parameter**	**Visit**	**Sample Date**	**Result**
xxxxxxxxxx/xx/x/x/x	Anti-GDNF Binding Serum Antibody	Screening Visit 1<br>…	DDMONYYYY	Negative
	Anti-GDNF Neutralizing Serum Antibody	…		Positive

Note: W = white; B = black; P = native Hawaiian or other Pacific islander; A = Asian; I = American Indian or Alaska native; H = Hispanic or Latino; N = not Hispanic or Latino; M = male; F = female.
Source: Dataset: [NAME], Program: xxxxxx.sas, Output: xxxx.rtf, Generated on: DDMONYYYY HH:MM
Page x of y

Programming Note: Repeat listing for Safety Pilot Stage (N=XXX) Placebo/GDNF (N=XXX), then Safety Primary Stage (N=XXX) GDNF/GDNF (N=XXX) and Placebo/GDNF (N=XXX). Sort by stage, treatment group, and subject ID then parameter and visit for screening from Study 2553and all postbaseline visits from Study 2553 and Study 2797. Include all Safety Overall subjects with data. Abbreviate as necessary. For all listings by visit, use actual visit labels and not “screening” or “baseline.”

Listing 17.2.4.9 Plasma GDNF Concentration Results by Visit - Safety Overall Population

Safety Pilot Stage (N=XXX)

GDNF/GDNF (N=XXX)

Subject ID Age/Race/Ethnicity/ Sex	Visit	Sample Date	Plasma GDNF Concentration (Unit)	Change from Baseline
xxxxxxxxxx/xx/x/x/x	Screening Visit 1	DDMONYYYY	xxx.xx	
	Week 40/e0	DDMONYYYY	xxx.xx	xx.xx
	....			

Note: W = white; B = black; P = native Hawaiian or other Pacific islander; A = Asian; I = American Indian or Alaska native; H = Hispanic or Latino; N = not Hispanic or Latino; M = male; F = female.
Source: Dataset: [NAME], Program: xxxxxx.sas, Output: xxxx.rtf, Generated on: DDMONYYYY HH:MM
Page x of y

Programming Note: Repeat listing for Safety Pilot Stage (N=XXX) Placebo/GDNF (N=XXX), then Safety Primary Stage (N=XXX) GDNF/GDNF (N=XXX) and Placebo/GDNF (N=XXX). Sort by stage, treatment group, and subject ID then parameter, and visit for screening from Study 2553, Week 40/e0, and all further visits in Study 2797. Include all Safety Overall subjects with data. For all listings by visit, use actual visit labels and not “screening” or “baseline.”

Listing 17.2.4.10 Physical Examination - Safety Overall Population

Safety Pilot Stage (N=XXX)

GDNF/GDNF (N=XXX)

Subject ID Age/Race/Ethnicity/ Sex	Body System	Visit	Date	Normal/ Abnormal/ Not Done	Abnormality
xxxxxxxxxx/xx/x/x/x	Skin	Week 80/e40	DDMONYYYY	xxxxxxxx	xxxxxxxxxxxxxxxxxxxx
		Week e2-80	DDMONYYYY	xxxxxxxx	xxxxxxxxxxxxxxxxxxxx
	Head, Ears, Eyes, Nose, and Throat	Week 80/e40	DDMONYYYY	xxxxxxxx	xxxxxxxxxxxxxxxxxxxx
		Week e2-80	DDMONYYYY	xxxxxxxx	xxxxxxxxxxxxxxxxxxxx
	Respiratory	Week 80/e40	DDMONYYYY	xxxxxxxx	xxxxxxxxxxxxxxxxxxxx
		Week e2-80	DDMONYYYY	xxxxxxxx	xxxxxxxxxxxxxxxxxxxx
	Cardiovascular	Week 80/e40	DDMONYYYY	xxxxxxxx	xxxxxxxxxxxxxxxxxxxx
		Week e2-80	DDMONYYYY	xxxxxxxx	xxxxxxxxxxxxxxxxxxxx
	Abdomen (incl. liver and kidneys)	Week 80/e40	DDMONYYYY	xxxxxxxx	xxxxxxxxxxxxxxxxxxxx
		Week e2-80	DDMONYYYY	xxxxxxxx	xxxxxxxxxxxxxxxxxxxx
	Musculoskeletal	Week 80/e40	DDMONYYYY	xxxxxxxx	xxxxxxxxxxxxxxxxxxxx
		Week e2-80	DDMONYYYY	xxxxxxxx	xxxxxxxxxxxxxxxxxxxx
	Neurological	Week 80/e40	DDMONYYYY	xxxxxxxx	xxxxxxxxxxxxxxxxxxxx
		Week e2-80	DDMONYYYY	xxxxxxxx	xxxxxxxxxxxxxxxxxxxx
	Gastrointestinal	Week 80/e40	DDMONYYYY	xxxxxxxx	xxxxxxxxxxxxxxxxxxxx
		Week e2-80	DDMONYYYY	xxxxxxxx	xxxxxxxxxxxxxxxxxxxx
	Genitourinary	Week 80/e40	DDMONYYYY	xxxxxxxx	xxxxxxxxxxxxxxxxxxxx
		Week e2-80	DDMONYYYY	xxxxxxxx	xxxxxxxxxxxxxxxxxxxx
	Endocrine	Week 80/e40	DDMONYYYY	xxxxxxxx	xxxxxxxxxxxxxxxxxxxx
		Week e2-80	DDMONYYYY	xxxxxxxx	xxxxxxxxxxxxxxxxxxxx
	Lymph nodes	Week 80/e40	DDMONYYYY	xxxxxxxx	xxxxxxxxxxxxxxxxxxxx
		Week e2-80	DDMONYYYY	xxxxxxxx	xxxxxxxxxxxxxxxxxxxx
	Other	Week 80/e40	DDMONYYYY	xxxxxxxx	xxxxxxxxxxxxxxxxxxxx
		Week e2-80	DDMONYYYY	xxxxxxxx	xxxxxxxxxxxxxxxxxxxx

Note: W = white; B = black; P = native Hawaiian or other Pacific islander; A = Asian; I = American Indian or Alaska native; H = Hispanic or Latino; N = not Hispanic or Latino; M = male; F = female.
Source: Dataset: [NAME], Program: xxxxxx.sas, Output: xxxx.rtf, Generated on: DDMONYYYY HH:MM
Page x of y

Programming Note: Repeat listing for Safety Pilot Stage (N=XXX) Placebo/GDNF *(N=XXX), then Safety Primary Stage (N=XXX) GDNF*/GDNF *(N=XXX) and Placebo*/GDNF *(N=XXX). Sort by stage, treatment group, and subject ID then body system and visit. Include all Safety Overall subjects with data. Visits are Week 80/e40 and e2-80. Abbreviate as necessary. For all listings by visit, use actual visit labels and not "screening" or "baseline."*

Listing 17.2.4.11 Vital Signs from Infusions with a Clinically Revelant Postbaseline Abnormal Result by Visit and Time Point - Safety Overall Population

			Safety Pilot Stage (N=XXX)				
			GDNF/GDNF (N=XXX)				
**Subject ID Age/Race/Ethnicity/ Sex**	**Parameter (Unit)**	**Visit**	**Date**	**Time Point**	**Result**	**Change from Baseline[a]**	**Clinically Relevant? If Y, then criteria**
xxxxxxxxxx/xx/x/x/x	xxxxxxxxxxxxx	Week e0	DDMONYYYY		xxx.xx		
		…					
		Week x	DDMONYYYY	Pre-infusion	xxx.xx		
				15 min after infusion start	xxx.xx	xx.xx	N
				…	…	…	Y/ < 50 bpm

[a] For test infusion and study medication infusion visits, change from baseline refers to change from pre-infusion value.

Note: W = white; B = black; P = native Hawaiian or other Pacific islander; A = Asian; I = American Indian or Alaska native; H = Hispanic or Latino; N = not Hispanic or Latino; M = male; F = female.

Note: All values for a given parameter with a clinically relevant abnormal result at a particular visit are listed.

Source: Dataset: [NAME], Program: xxxxxx.sas, Output: xxxx.rtf, Generated on: DDMONYYYY HH:MM

Page x of y

*Programming Note: Repeat listing for Safety Pilot Stage (N=XXX) Placebo/*GDNF *(N=XXX), then Safety Primary Stage (N=XXX) GDNF/*GDNF *(N=XXX) and Placebo/*GDNF *(N=XXX). Sort by stage, treatment group, and subject ID then parameter, visit, and time point. Include all Safety Overall subjects with data. Parameters are pulse (sitting and standing; beats/min), respiration (breaths/min), systolic and diastolic blood pressure (BP; sitting and standing; mmHg), and temperature (℃). Data are presented for all test infusion visits (interim visits after catheter repositioning and Week 80/e40 visits) and study medication infusion visits in the Initial Extension, including pre-infusion value, 15 min after infusion start, 30 min after infusion start, 45 min after infusion start, 60 min after infusion start, 75 min after infusion start, 90 min after infusion start, 105 min after infusion start, 120 min after infusion start, and post-dose, where applicable. If clinically relevant, then concatenate the criteria listed in SAP Table 6. For all listings by visit, use actual visit labels and not "screening" or "baseline."*

Listing 17.2.4.12 Weight by Visit - Safety Overall Population

*Programming Note: Repeat UPDRS Listing 17.2.2.1 omitting the parameter column. Repeat listing for Safety Pilot Stage (N=XXX) Placebo/*GDNF *(N=XXX), then Safety Primary Stage (N=XXX) GDNF/*GDNF *(N=XXX) and Placebo/*GDNF *(N=XXX). Sort by stage, treatment group, and subject ID then parameter and visit for Week 40/e0 and all further visits in Study 2797. Include all Safety Overall subjects with data. Abbreviate as necessary. For all listings by visit, use actual visit labels and not "screening" or "baseline."*

Listing 17.2.4.13 Electrocardiogram Results by Visit – Continuous Parameters, Safety Overall Population

*Programming Note: Repeat VS Listing 17.2.4.12 (without column for time points). Repeat listing for Safety Pilot Stage (N=XXX) Placebo/*GDNF *(N=XXX), then Safety Primary Stage (N=XXX) GDNF/*GDNF *(N=XXX) and Placebo/*GDNF *(N=XXX). Sort by stage, treatment group, and subject ID then parameter and visit for screening, Week 40/e0, and all further visits in Study 2797. Include all Safety Overall subjects with data. Parameters are heart rate (beats/min), PR interval (ms), QRS interval (ms), QT interval (ms), and QTc interval (ms). Abbreviate as necessary. If clinically relevant, then concatenate the criteria listed in SAP Table 7. For all listings by visit, use actual visit labels and not "screening" or "baseline." Add the following footnote:*
Note: Hodges QT correction formula was used.

Listing 17.2.4.14 Electrocardiogram Results by Visit – Overall Impression, Safety Overall Population

Safety Pilot Stage (N=XXX)

GDNF/GDNF (N=XXX)

Subject ID Age/Race/Ethnicity/Sex	Visit	Date	Overall Impression (Normal or Abnormal?)	Clinically Significant?	Comment
xxxxxxxxxx/xx/x/x/x	Screening Visit 1	DDMONYYYY	Abnormal	Yes	xxxxxxxxxxxxxxxxxxxxxxxx
	Week 40/e0	DDMONYYYY	Normal		
	...				

Note: W = white; B = black; P = native Hawaiian or other Pacific islander; A = Asian; I = American Indian or Alaska native; H = Hispanic or Latino; N = not Hispanic or Latino; M = male; F = female.
Source: Dataset: [NAME], Program: xxxxxx.sas, Output: xxxx.rtf, Generated on: DDMONYYYY HH:MM
Page x of y

*Programming Note: Repeat listing for Safety Pilot Stage (N=XXX) Placebo/*GDNF *(N=XXX), then Safety Primary Stage (N=XXX) GDNF/*GDNF *(N=XXX) and Placebo/*GDNF *(N=XXX). Sort by stage, treatment group, and subject ID then visit for screening, Week 40/e0, and all further visits in Study 2797. Include all Safety Overall subjects with data. Abbreviate as necessary. For all listings by visit, use actual visit labels and not "screening" or "baseline."*

Listing 17.2.4.15 Glasgow Coma Scale Results < 15 by Visit - Safety Overall Population

Safety Pilot Stage (N=XXX)

GDNF/GDNF (N=XXX)

Subject ID Age/Race/ Ethnicity/Sex	Parameter	Visit	Date and Time	Time Point	Result
xxxxxxxxxx/xx/x/x/x	Visual Response	Week e0	DDMONYYYY HHMM	Pre-infusion	x
			DDMONYYYY HHMM	30 min after infusion start	x
			DDMONYYYY HHMM	Post-infusion	x
	Verbal Ability		DDMONYYYY HHMM	Pre-infusion	x
			DDMONYYYY HHMM	30 min after infusion start	x
		…	…	…	…

Note: Glasgow Coma Scale items include visual response, verbal ability, and motor skills. Each item has 4-6 possible responses. The best possible total score is 15. W = white; B = black; P = native Hawaiian or other Pacific islander; A = Asian; I = American Indian or Alaska native; H = Hispanic or Latino; N = not Hispanic or Latino; M = male; F = female.
Source: Dataset: [NAME], Program: xxxxxx.sas, Output: xxxx.rtf, Generated on: DDMONYYYY HH:MM
Page x of y

Programming Note: Repeat listing for Safety Pilot Stage (N=XXX) Placebo/GDNF *(N=XXX), then Safety Primary Stage (N=XXX) GDNF*/GDNF *(N=XXX) and Placebo*/GDNF *(N=XXX). Sort by stage, treatment group, and subject ID, then parameter, visit, date, and time point for all visits in Study 2797. Include all Safety Overall subjects with a result <15 for a particular visit or missing in all extension parts of Study 2797 (Initial Extension, Pilot Extension, Supplemental Extension). Abbreviate as necessary. Include the scoring information in SAP Table 8 in an endnote.*

Listing 17.2.4.16 QUIP Items Answered with Yes by Visit - Safety Overall Population

Safety Pilot Stage (N=XXX)

GDNF/GDNF (N=XXX)

Subject ID Age/Race/ Ethnicity/Sex	Reported By	Question	Behavior	Visit	Date
xxxxxxxxxx/xx/x/x/x	Subject	Do you or others think you have an issue with too much gambling, sex, buying, or eating behaviors?	Gambling	Week 40/e0	DDMONYYYY
		…	…	Week 48/e8	DDMONYYYY
			Sex	…	DDMONYYYY
			Buying	…	DDMONYYYY
			Eating	…	DDMONYYYY
			…	…	
	Informant	Do you or others think you have an issue with too much gambling, sex, buying, or eating behaviors?	Gambling	Week 40/e0	DDMONYYYY
		…	…	…	…

Note: The QUIP is a self-administered or informant-completed scale that includes 13 questions covering symptoms related to the 4 commonest impulse control disorders in PD, other behaviors, and problematic use of medication. W = white; B = black; P = native Hawaiian or other Pacific islander; A = Asian; I = American Indian or Alaska native; H = Hispanic or Latino; N = not Hispanic or Latino; M = male; F = female.
Source: Dataset: [NAME], Program: xxxxxx.sas, Output: xxxx.rtf, Generated on: DDMONYYYY HH:MM
Page x of y

Programming Note: Repeat listing for Safety Pilot Stage (N=XXX) Placebo/GDNF *(N=XXX), then Safety Primary Stage (N=XXX) GDNF*/GDNF *(N=XXX) and Placebo*/GDNF *(N=XXX). Sort by stage, treatment group, and subject ID, then reported by, question, behavior, visit, and date for Week 40/e0 and all further visits in Study 2797. Include all Safety Overall subjects with data. Do not list imputed values. Note that behavior column is not applicable for "Other Behaviors" (subquestions can go in "Behavior" column) and "Medication Use" (leave "Behavior" column blank) sections. For all listings by visit, use actual visit labels and not "screening" or "baseline."*

Listing 17.2.4.17 MoCA Total Score by Visit - Safety Overall Population

Safety Pilot Stage (N=XXX)

GDNF/GDNF (N=XXX)

**Subject ID Age/Race/ Ethnicity/Sex**	**Visit**	**Date**	**Result**
xxxxxxxxxx/xx/x/x/x	Screening Visit 1	DDMONYYYY	x
	Pre-test infusion	DDMONYYYY	x
	Week 40/e0	DDMONYYYY	x
		DDMONYYYY	x
		DDMONYYYY	x
	…	…	…

Note: The MoCA is a rater-administered cognitive screening tool with 8 components. The total score ranges from 0 to 30, with lower scores representing poorer cognitive function. A total score of 26 or above is considered normal. W = white; B = black; P = native Hawaiian or other Pacific islander; A = Asian; I = American Indian or Alaska native; H = Hispanic or Latino; N = not Hispanic or Latino; M = male; F = female.

Source: Dataset: [NAME], Program: xxxxxx.sas, Output: xxxx.rtf, Generated on: DDMONYYYY HH:MM

Page x of y

Programming Note: Repeat listing for Safety Pilot Stage (N=XXX) Placebo/GDNF *(N=XXX), then Safety Primary Stage (N=XXX) GDNF*/GDNF *(N=XXX) and Placebo*/GDNF *(N=XXX). Sort by stage, treatment group, and subject ID, then visit and date for screening, pre-infusion, Week 40/e0, and all further visits in Study 2797. Include all Safety Overall subjects with data. Do not list imputed values. For all listings by visit, use actual visit labels and not “screening” or “baseline.”*

Listing 17.2.4.18 MDRS AEMSS Total Score by Visit - Safety Overall Population

Safety Pilot Stage (N=XXX)

GDNF/GDNF (N=XXX)

Subject ID<br>Age/Race/<br>Ethnicity/Sex	Visit	Date	AEMSS Total Score
xxxxxxxxxx/xx/x/x/x	Screening Visit 1	DDMONYYYY	x
	Week 40/e0	DDMONYYYY	x
		DDMONYYYY	x
		DDMONYYYY	x
		DDMONYYYY	x
	…	…	x
			xxx

Note: The MDRS is a rater-administered global scale of cognition including 5 subscales. The total score ranges from 0 to 144, with higher scores representing better cognitive function. A total score lower than 123 is associated with some degree of dementia in PD. W = white; B = black; P = native Hawaiian or other Pacific islander; A = Asian; I = American Indian or Alaska native; H = Hispanic or Latino; N = not Hispanic or Latino; M = male; F = female.
Source: Dataset: [NAME], Program: xxxxxx.sas, Output: xxxx.rtf, Generated on: DDMONYYYY HH:MM
Page x of y

Programming Note: Repeat listing for Safety Pilot Stage (N=XXX) Placebo/GDNF *(N=XXX), then Safety Primary Stage (N=XXX) GDNF*/GDNF *(N=XXX) and Placebo*/GDNF *(N=XXX). Sort by stage, treatment group, and subject ID, then visit and date for screening, Week 40/e0, and all further visits in Study 2797. Include all Safety Overall subjects with data. Do not list imputed values. For all listings by visit, use actual visit labels and not "screening" or "baseline."*

Listing 17.2.4.19 Stroop Test by Visit - Safety Overall Population

Safety Pilot Stage (N=XXX)

GDNF/GDNF (N=XXX)

Subject ID Age/Race/ Ethnicity/Sex	Condition	Visit	Date and Time	Total Uncorrected Errors	Total Self-Corrected Errors	Total Time to Complete (secs)
xxxxxxxxxx/xx/x/x/x	Color Naming	Screening Visit 2	DDMONYYYY HHMM	xx	xx	xx
		Week 40/e0	DDMONYYYY HHMM	xx	xx	xx
		Week 80/e40				
	Word Reading		DDMONYYYY HHMM			
	Inhibition		DDMONYYYY HHMM			
	Inhibition/Switching	…	…			

Note: The Stroop test is a global scale of reaction time including 4 conditions. Total time to complete the test in each condition can range from 0 to 999 seconds, with lower time representing better reaction time. W = white; B = black; P = native Hawaiian or other Pacific islander; A = Asian; I = American Indian or Alaska native; H = Hispanic or Latino; N = not Hispanic or Latino; M = male; F = female.

Source: Dataset: [NAME], Program: xxxxxx.sas, Output: xxxx.rtf, Generated on: DDMONYYYY HH:MM

Page x of y

Programming Note: Repeat listing for Safety Pilot Stage (N=XXX) Placebo/GDNF *(N=XXX), then Safety Primary Stage (N=XXX) GDNF*/GDNF *(N=XXX) and Placebo*/GDNF *(N=XXX). Sort by stage, treatment group, and subject ID, then condition (CRF order), visit, and date for screening, Week 40/e0, and Week 80/e40. Include all Safety Overall subjects with data. For all listings by visit, use actual visit labels and not "screening" or "baseline."*

Listing 17.2.4.20 FrSBe by Visit - Safety Overall Population

Safety Pilot Stage (N=XXX)

GDNF/GDNF (N=XXX)

Subject ID Age/Race/ Ethnicity/Sex	Scale	Visit	Date	Time Point	Result
xxxxxxxxxx/xx/x/x/x	Apathy Score	Screening Visit 2	DDMONYYYY	Before	x
		Screening Visit 2	DDMONYYYY	After	x
		Week 40/e0	DDMONYYYY	After	x
		Week 80/e40	DDMONYYYY	After	
	Disinhibition	…	…	Before	…
	Disinhibition			After	
	Executive Dysfunction			Before	
	Executive Dysfunction			After	

Note: The FrSBe is a scale that assesses behavior related to frontal systems damage including 3 subscales. Higher subscale scores indicate greater pathology. W = white; B = black; P = native Hawaiian or other Pacific islander; A = Asian; I = American Indian or Alaska native; H = Hispanic or Latino; N = not Hispanic or Latino; M = male; F = female.

Source: Dataset: [NAME], Program: xxxxxx.sas, Output: xxxx.rtf, Generated on: DDMONYYYY HH:MM

Page x of y

Programming Note: Repeat listing for Safety Pilot Stage (N=XXX) Placebo/GDNF *(N=XXX), then Safety Primary Stage (N=XXX) GDNF*/GDNF *(N=XXX) and Placebo*/GDNF *(N=XXX). Sort by stage, treatment group, and subject ID, then scale (CRF order), visit, and date for screening, Week 40/e0, and Week 80/e40. Include all Safety Overall subjects with data. Baseline value is "after" at Screening Visit 2; Week 80/e40 data has "after" only. Do not list imputed values. For all listings by visit, use actual visit labels and not "screening" or "baseline."*

Listing 17.2.4.21 Deary-Liewald Four-Choice Reaction Time by Visit - Safety Overall Population

Safety Pilot Stage (N=XXX)

GDNF/GDNF (N=XXX)

**Subject ID Age/Race/ Ethnicity/Sex**	**For correct responses for the four-choice RT: Parameter**	**Visit**	**Date**	**Result (ms)**
xxxxxxxxxx/xx/x/x/x	Mean reaction time	Screening Visit 2	DDMONYYYY	x
	Variance	Week 40/e0	DDMONYYYY	x
	SD	Week 56/e16	DDMONYYYY	x
		Week 80/e40	DDMONYYYY	x

Note: The Deary-Liewald RT is a computerized measure of simple and four-choice reaction time. The parameter reported is the mean reaction time, variance, and SD for correct responses for four-choice reaction time. A shorter reaction time is better. W = white; B = black; P = native Hawaiian or other Pacific islander; A = Asian; I = American Indian or Alaska native; H = Hispanic or Latino; N = not Hispanic or Latino; M = male; F = female.

Source: Dataset: [NAME], Program: xxxxxx.sas, Output: xxxx.rtf, Generated on: DDMONYYYY HH:MM

Page x of y

Programming Note: Repeat listing for Safety Pilot Stage (N=XXX) Placebo/GDNF *(N=XXX), then Safety Primary Stage (N=XXX) GDNF*/GDNF *(N=XXX) and Placebo*/GDNF *(N=XXX). Sort by stage, treatment group, and subject ID, then parameter, visit, and date for screening, Week 40/e0, Week 56/e16, and Week 80/e40. Include all Safety Overall subjects with data. For all listings by visit, use actual visit labels and not “screening” or “baseline.”*

Listing 17.2.4.22 Verbal Fluency Assessment by Visit - Safety Overall Population

Safety Pilot Stage (N=XXX)

GDNF/GDNF (N=XXX)

Subject ID Age/Race/ Ethnicity/Sex	Test	Visit	Date	Number of Correct Responses in 1 Minute
xxxxxxxxxx/xx/x/x/x	Phonemic Verbal Fluency	Screening Visit 2	DDMONYYYY	xx
		Week 40/e0	DDMONYYYY	xx
		Week 80/e40		
	Semantic Verbal Fluency	Screening Visit 2	DDMONYYYY	xx
		Week 40/e0	DDMONYYYY	xx
		Week 80/e40		

Note: The verbal fluency assessment measures verbal functioning in 2 categories. Scores represent number of correct words in one minute and range from 0 to 200. Higher scores represent better verbal functioning. W = white; B = black; P = native Hawaiian or other Pacific islander; A = Asian; I = American Indian or Alaska native; H = Hispanic or Latino; N = not Hispanic or Latino; M = male; F = female.

Source: Dataset: [NAME], Program: xxxxxx.sas, Output: xxxx.rtf, Generated on: DDMONYYYY HH:MM

Page x of y

Programming Note: Repeat listing for Safety Pilot Stage (N=XXX) Placebo/GDNF *(N=XXX), then Safety Primary Stage (N=XXX) GDNF*/GDNF *(N=XXX) and Placebo*/GDNF *(N=XXX). Sort by stage, treatment group, and subject ID, then test, visit, and date for screening, Week 40/e0, and Week 80/e40. Include all Safety Overall subjects with data. For all listings by visit, use actual visit labels and not "screening" or "baseline."*

Listing 17.2.4.23 BDI by Visit - Safety Overall Population

Safety Pilot Stage (N=XXX)

GDNF/GDNF (N=XXX)

**Subject ID Age/Race/ Ethnicity/Sex**	**Visit**	**Date**	**Total Score**
xxxxxxxxxx/xx/x/x/x	Screening Visit 2	DDMONYYYY	xx
	Week 40/e0	DDMONYYYY	
	Week 80/e40	DDMONYYYY	
	Last study visit, Pilot Extension	DDMONYYYY	
	Last study visit, Supplemental Extension	DDMONYYYY	

Note: The BDI is a self-administered test which consists of 21 questions that measure the severity of depression. Scores range from 0 to 63, with higher scores representing worse depression. W = white; B = black; P = native Hawaiian or other Pacific islander; A = Asian; I = American Indian or Alaska native; H = Hispanic or Latino; N = not Hispanic or Latino; M = male; F = female.

Source: Dataset: [NAME], Program: xxxxxx.sas, Output: xxxx.rtf, Generated on: DDMONYYYY HH:MM

Page x of y

Programming Note: Repeat listing for Safety Pilot Stage (N=XXX) Placebo/GDNF *(N=XXX), then Safety Primary Stage (N=XXX) GDNF*/GDNF *(N=XXX) and Placebo*/GDNF *(N=XXX). Sort by stage, treatment group, and subject ID, then visit and date for screening, Week 40/e0, Week 80/e40 and the last study visit in both the Pilot Extension and the Supplemental Extension. Include all Safety Overall subjects with data. For all listings by visit, use actual visit labels and not "screening" or "baseline."*

Listing 17.2.4.24 UPSIT by Visit - Safety Overall Population

Safety Pilot Stage (N=XXX)

GDNF/GDNF (N=XXX)

Subject ID Age/Race/ Ethnicity/Sex	Visit	Date	Total Number of Correct Responses	Percentile Value	Descriptive Term
xxxxxxxxxx/xx/x/x/x					Normosmia/ Mild microsmia/ Moderate microsmia/ Severe microsmia/ Anosmia/ Probable malingering
	Screening Visit 2	DDMONYYYY	xx	xx	
	Week 40/e0	DDMONYYYY	xx	xx	
	Week 80/e40	DDMONYYYY	xx	xx	
	..				

Note: The UPSIT is a self-administered test which can be used to identify and quantitate olfactory dysfunction in PD. The number of correct responses out of 40 total items constitutes a subject's score. Lower scores represent greater olfactory dysfunction. W = white; B = black; P = native Hawaiian or other Pacific islander; A = Asian; I = American Indian or Alaska native; H = Hispanic or Latino; N = not Hispanic or Latino; M = male; F = female.
Source: Dataset: [NAME], Program: xxxxxx.sas, Output: xxxx.rtf, Generated on: DDMONYYYY HH:MM
Page x of y
Programming Note: Repeat listing for Safety Pilot Stage (N=XXX) Placebo/GDNF *(N=XXX), then Safety Primary Stage (N=XXX) GDNF*/GDNF *(N=XXX) and Placebo*/GDNF *(N=XXX). Sort by stage, treatment group, and subject ID, then visit, and date for screening, Week 40/e0, and Week 80/e40. Include all Safety Overall subjects with data. For all listings by visit, use actual visit labels and not "screening" or "baseline."*

Document History

Version Date	Modified/Reviewed By	Brief Summary of changes to current version
29-NOV-2016	Emma Lewis/ Kerri Barber/ Liam Collard/ Lara Longpre/ Matthias Luz	Draft version 0.1. Created from PRS 005 T 17 E.
16-DEC-2016	Emma Lewis/ Lara Longpre/ Matthias Luz	Draft version 0.2.
10-JAN-2017	Emma Lewis/ Lara Longpre/ Matthias Luz	Draft version 0.3.
10-FEB-2017	Emma Lewis/ Lara Longpre/ Matthias Luz	Final version 1.0.

Statistical Analysis Plan

**Sponsor:**	North Bristol NHS Trust (NBT)
**Client:**	MedGenesis Therapeutix
**Protocol No./EudraCT No.:**	GDNF 2797 / 2013-001881-40
**Version No./Date**	1.4 (Incorporating Amendments 1-4) / 16-Dec-2015
**Title**	An Extension Study to Assess the Safety and Efficacy of Intermittent Bilateral Intraputamenal Glial Cell Line-Derived Neurotrophic Factor (GDNF) Infusions Administered via Convection Enhanced Delivery (CED) in Subjects with Parkinson's Disease
**PRA Project Id:**	MDGGNDNFD-GDNFDM
**SAP Version No./Date:**	Version 2.0 / 10-Apr-2017

Approvals

**Principal Investigator (PI)**	
**PI Affiliation:**	Department of Neurology and the Burden Institute<br>Movement Disorder Service,<br>Brain Centre,<br>Southmead Hospital, North Bristol NHS Trust<br>Bristol BS10 5NB, United Kingdom
**PI Name, Title:**	Alan Whone, FRCP, PhD, Consultant Senior Lecturer and Hon Consultant Neurologist
**Signature, Date:**	
**Sponsor**	
**Sponsor Name:**	North Bristol NHS Trust (NBT)
**Representative, Title:**	Rebecca Smith, PhD, Deputy Director of Research
**Signature, Date:**	
**Client**	
**Client Name:**	MedGenesis Therapeutix
**Representative, Title:**	Lara Longpre, Chief Operating Officer
**Signature, Date:**	
**Representative, Title:**	Matthias Luz, MD, Chief Medical Officer
**Signature, Date:**	
**PRA**	
**Project Manager, Title:**	Diana Soto, Project Manager
**Signature, Date:**	
**Biostatistician, Title:**	Emma Lewis, Principal Biostatistician
**Signature, Date:**	

Table of Contents

Approvals 2
Table of Contents 3
1.0 Introduction 5
1.1 Changes from Protocol 5
2.0 Study Objectives 6
2.1 Primary Objective 6
2.2 Secondary Objectives 7
2.3 Other Objectives 7
3.0 Study Design 7
3.1 Sample Size Considerations 15
3.2 Randomization 15
4.0 Study Endpoints 15
4.1 Efficacy Endpoints 15
4.1.1 Primary Efficacy Endpoint 15
4.1.2 Secondary Efficacy Endpoints 15
4.1.3 Supplementary Efficacy Endpoints 16
4.2 Imaging Endpoints 16
4.3 Safety Endpoints 17
5.0 Definitions 18
6.0 Analysis Populations 27
6.1 Intent-to-Treat Populations 27
6.1.1 ITT Primary Population 27
6.1.2 ITT Overall Population 27
6.2 Safety Population 27
6.2.1 Safety Overall Population 27
7.0 Interim Analyses 27
8.0 Data Review 27
8.1 Data Handling and Transfer 27
8.2 Data Screening 28
9.0 Statistical Methods 28
9.1 Missing Data Methods 28
9.1.1 Missing or Partial Dates 28
9.1.2 Missing Efficacy Data 29
9.1.3 Missing Imaging Data 29
9.1.4 Missing Data for Questionnaires 29
9.1.5 Missing Safety Data 29
9.1.6 Special Arrangements for Subject 45 29
9.2 Subject Disposition 29
9.3 Protocol Deviations 30
9.4 Demographic and Baseline Characteristics 30
9.4.1 Demographic Characteristics 30
9.4.2 Parkinson's Disease History at Screening 31
9.5 Concomitant Medications 31
9.6 Surgery and Test Infusions 31
9.6.1 Catheter Trajectories and Positioning Accuracy 31
9.6.2 Contrast-Enhanced Test Infusions with T1-Weighted MRI Prior to Start of Study Medication 31
9.7 Efficacy Analyses 32
9.7.1 Analyses of Primary Efficacy Endpoint 32
9.7.2 Analyses of Secondary Efficacy Endpoints 33

9.7.3 Analyses of Supplementary Efficacy Endpoints 36
9.8 Imaging Analyses 39
9.8.1 MRI Analyses 39
9.8.2 Correlation Analyses 40
9.9 Safety Analyses 42
9.9.1 Study Medication Exposure 42
9.9.2 Adverse Events 42
9.9.3 Port Symptoms 43
9.9.4 Laboratory Data 43
9.9.5 Anti-GDNF Serum Antibodies 44
9.9.6 Plasma GDNF Concentrations 44
9.9.7 Physical Examination 45
9.9.8 Vital Signs 45
9.9.9 Weight 45
9.9.10 Electrocardiogram 45
9.9.11 Glasgow Coma Scale 46
9.9.12 Questionnaire for Impulsive-Compulsive Disorders 47
9.9.13 Montreal Cognitive Assessment 47
9.9.14 Mattis Dementia Rating Scale 47
9.9.15 Stroop Test 47
9.9.16 Frontal Systems Behavioural Scale 48
9.9.17 Deary-Liewald Reaction Time 48
9.9.18 Verbal Fluency Assessment 48
9.9.19 Beck Depression Inventory 48
9.9.20 University of Pennsylvania Smell Identification Test 49
10.0 Validation 49
Appendix 1 Glossary of Abbreviations 50
Appendix 2 Protocol Deviation Guidance 52
Appendix 3 List of MedDRA Preferred Terms for Adverse Events of Special Interest 54
Appendix 4 List of Conversion Factors for the Calculation of Levodopa and Levodopa Equivalent Doses 55
Appendix 5 List of Post-Text Tables, Figures and Listings 56
Appendix 6 Shells for Post-Text Tables, Figures and Listings 63
Document History 187

1.0 Introduction

This statistical analysis plan (SAP) describes the statistical methods to be used during the reporting and analyses of data collected under North Bristol NHS Trust (NBT) Protocol 2797.

This SAP should be read in conjunction with the study protocol and case report form (CRF). This version of the plan has been developed using the protocol version 1.4 (incorporating Amendments 1-4) dated 16DEC2015 and the corresponding CRF. Any further changes to the protocol or CRF may necessitate updates to the SAP.

The SAP was developed in two stages. The purpose was to “finalize” an SAP so that PRA could start programming earlier in the process. Versions of the SAP up to initial approval were known as SAP1. Changes following approval of SAP1 were tracked in the SAP Change Log and a final version of the SAP, known as SAP2, was issued for approval prior to database lock.

1.1 Changes from Protocol

No inferential analyses are described in the protocol. All inferential analyses described in the SAP will be interpreted in an exploratory manner only.

During the preparation of the SAP for the parent Study 2553, it was recognized that the wording of certain study endpoints was less clear than anticipated. In addition, a number of endpoints were added in order to provide for a more comprehensive analysis of the study data. The endpoints in Study 2797 have been refined in a similar manner to achieve consistency between the protocols and to improve the preciseness of the definitions. In addition, treatment response has been added as a secondary endpoint pursuant to the post-hoc analysis of Study 2553. Due to the open, uncontrolled design of the extension study, these changes have been implemented in the SAP without amending the protocol itself.

No change has been made to the primary efficacy endpoint.

The secondary efficacy endpoints have been reworded and expanded (including some endpoints originally classified as supplementary) as follows:

- Change from baseline to Week 80/e40 in OFF state UPDRS motor score (part III).
- Change and percentage change from baseline to Week 80/e40 in:
  - UPDRS motor score (part III) in the ON state (following a levodopa challenge).
  - UPDRS activities of daily living (ADL) score (part II) in the OFF state and in the ON state.
  - UPDRS total score (sum of motor + ADL scores) in the OFF state and in the ON state.
- Change from baseline to Week 40/e0 for the GDNF/GDNF group compared to change from baseline to Week 80/e40 for the placebo/GDNF group in:
  - OFF state UPDRS motor score (part III).
  - OFF state UPDRS ADL score (part II).
  - OFF state UPDRS total score (sum of motor + ADL scores).
- Change from baseline to Week 80/e40 for the GDNF/GDNF group compared to change from baseline to Week 40/e0 for the placebo/GDNF group in:
  - OFF state UPDRS motor score (part III).
  - OFF state UPDRS ADL score (part II).
  - OFF state UPDRS total score (sum of motor + ADL scores).
- Change from baseline to Week 80/e40 in PD diary ratings:
  - Total OFF time per day.
  - Total good-quality ON time per day (ON without dyskinesias or ON with non-troublesome dyskinesias).

- ON time per day with troublesome dyskinesias.

- Treatment response based on the following criteria:
  - Decrease from baseline to Week 80/e40 by ≥10 points in OFF state UPDRS motor score (part III).
  - Increase from baseline to Week 80/e40 by ≥1 hour in total good-quality ON time per day (ON without dyskinesias or ON with non-troublesome dyskinesias).
  - Both of the above criteria, i.e. decrease from baseline to Week 80/e40 by ≥10 points in OFF state UPDRS motor score (part III) and increase from baseline to Week 80/e40 by ≥1 hour in total good-quality ON time per day (ON without dyskinesias or ON with non-troublesome dyskinesias).

The efficacy endpoint definitions provided in the SAP are independent of the corresponding analysis populations. The analysis populations for the individual efficacy endpoints are specified in Section 9.7.

The following imaging endpoints have been added:

- Change from baseline to Week 80/e40 in volume of distribution of infusate as determined by contrast-enhanced T1-weighted MRI.
- Change from baseline to Week 80/e40 in volume of interest (VOI) coverage and total putamenal coverage as determined by contrast-enhanced T1-weighted MRI.
- Correlation between primary study endpoint and VOI coverage and total putamenal coverage at baseline as determined by contrast-enhanced T1-weighted MRI.
- Correlation between primary study endpoint and change from baseline to Week 40/e0 in ^{18}F-DOPA uptake as determined by PET scan.
- Correlation between baseline OFF state UPDRS motor score (part III) and baseline ^{18}F-DOPA uptake as determined by PET scan.
- Correlation between baseline OFF state UPDRS ADL score (part II) and baseline ^{18}F-DOPA uptake as determined by PET scan.
- Correlation between Week 40/e0 OFF state UPDRS motor score (part III) and Week 40/e0 ^{18}F-DOPA uptake as determined by PET scan.
- Correlation between Week 40/e0 OFF state UPDRS ADL score (part II) and Week 40/e0 ^{18}F-DOPA uptake as determined by PET scan.
- Correlation between Week 80/e40 OFF state UPDRS motor score (part III) and Week 40/e0 ^{18}F-DOPA uptake as determined by PET scan.
- Correlation between Week 80/e40 OFF state UPDRS ADL score (part II) and Week 40/e0 ^{18}F-DOPA uptake as determined by PET scan.

The wording of the safety endpoints has been revised to provide more clarity and accuracy of the individual parameters to be analyzed. Time with troublesome dyskinesias (from subject diaries) has been removed from the list of safety endpoints since it is already listed as a secondary efficacy endpoint. Full brain MRI has been removed from the list as it is redundant with adverse changes in MRI findings. The new wording of the safety endpoints is shown in Section 4.3.

2.0 Study Objectives

2.1 Primary Objective

To compare the effects of intermittent bilateral intraputamenal GDNF infusions on OFF state motor function after 18 months of treatment with the effects after 9 months of treatment in subjects who completed Study 2553.

2.2 Secondary Objectives

- To compare the effects of intermittent bilateral intraputamenal GDNF infusions on ON state motor function, motor complications, and ON and OFF state activities of daily living (ADL) after 18 months of treatment with the effects after 9 months of treatment in subjects who completed Study 2553.
- To assess the safety of intermittent bilateral intraputamenal GDNF infusions at 18 months in subjects who received GDNF or placebo for 9 months in Study 2553.

2.3 Other Objectives

- To explore the effects of intermittent bilateral intraputamenal GDNF infusions on other motor and non-motor functions, quality of life (QOL) assessments, and imaging endpoints at 18 months in subjects who completed Study 2553.
- To compare the results for various motor outcomes between the subjects who started GDNF early (i.e. were randomized to GDNF in Study 2553) and those who started GDNF late (i.e. were randomized to placebo in Study 2553).
- Pilot and Supplemental Extensions: To generate long-term safety data and provide continued access to GDNF until the end of December 2016 when the results of Study 2553 are expected, which will inform interested parties with potential future studies.

3.0 Study Design

This is a phase II, single-center, open-label trial of intermittent bilateral intraputamenal GDNF infusions administered via convection-enhanced delivery (CED) in subjects with idiopathic PD who have completed Study 2553.

Following the final study visit at Week 40 in Study 2553, study completers return within one week to receive their first infusion of open-label GDNF. GDNF is administered using the same treatment protocol as in Study 2553. Treatment is given at 4-weekly intervals for 9 months (40 weeks; 10 infusions total). Hence, at 18 months, subjects receiving GDNF in Study 2553 have been treated with GDNF for a total of 18 months, while those receiving placebo in Study 2553 have been treated with GDNF for a total of 9 months.

Key clinical outcomes are measured at 8-week intervals throughout this initial 9-month extension (the “Initial Extension”). The Schedule of Events for this extension is shown in Table 1.

Pilot Stage subjects who complete the Initial Extension and provide informed consent are eligible for up to an additional 80 weeks of treatment with GDNF (the “Pilot Extension”). The Schedule of Events for this extension is shown in Table 2.

Pilot Stage subjects completing the Pilot Extension and Primary Stage subjects completing the Initial Extension who provide informed consent are eligible to enroll in a further extension (the “Supplemental Extension”) and continue to receive 4-weekly GDNF infusions until the end of December 2016. The Schedule of Events for this extension is shown in Table 3.

Figure 1 presents the study schema.

Although the statistical assessment of Study 2553 was performed before completion of the extension study, to reduce any potential for bias in this study, individual treatment codes from the parent study will not be disclosed to subjects until database lock for Study 2797, unless required for specific safety reasons. In addition, every effort will be made to avoid unblinding of the blinded UPDRS raters before database lock for Study 2797.

The primary analysis of the extension study is the intention-to-treat (ITT) analysis of the percentage change from baseline (in Study 2553) to Week 80/e40 in the practically defined OFF state UPDRS motor score (part III) in Primary Stage subjects.

Figure 1. Study Schema

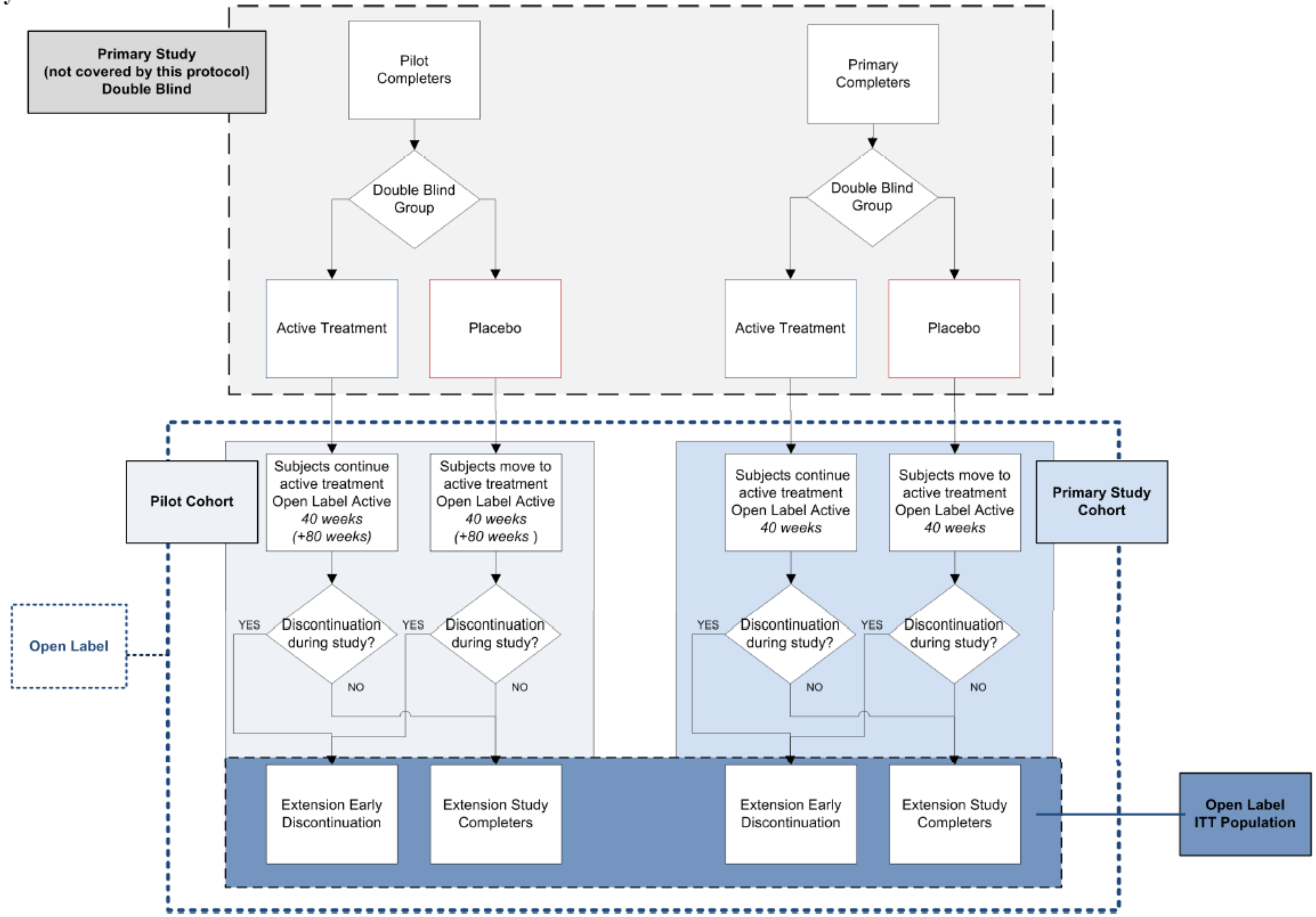

Note: The schema does not include the Supplemental Extension part of the study.

Table 1. Schedule of Events - Initial Extension

Procedure/Assessments	Week										
	40/e0[a]	44/e4	48/e8[b]	52/e12	56/e16[b]	60/e20	64/e24[b]	68/e28	72/e32[b]	76/e36	80/e40[b]
Informed consent[c]	(X)										
MRI[d]											X
Vital signs[e]	X	X	X	X	X	X	X	X	X	X	X
Weight and height	(X)		X		X		X		X		X
Physical examination[f]											X
Port review	X	X	X	X	X	X	X	X	X	X	X
ECG											X
Laboratory assessment[g]		X			X			X			X
Anti-GDNF serum antibody levels and GDNF plasma concentrations		X			X			X			X
UPDRS part II and part III in OFF state	(X)		X		X		X		X		X
Timed walking test in OFF state	(X)		X		X		X		X		X
Timed tapping test in OFF state	(X)		X		X		X		X		X
Levodopa challenge	(X)		X		X		X		X		X
UPDRS in ON state	(X)		X		X		X		X		X
Timed walking test in ON state	(X)		X		X		X		X		X
Timed tapping test in ON state	(X)		X		X		X		X		X
PDQ-39											X
EQ-5D											X
MoCA and MDRS					X						X
Stroop test											X
UPSIT											X
NMSS				X			X				X
BDI											X
QUIP	(X)		X		X		X		X		X
Deary-Liewald reaction time					X						X
SNAQ											X
FrSBe											X
Verbal fluency											X
Direct questioning of impulsivity, mood, falls and freezing for recording in case notes	X	X	X	X	X	X	X	X	X	X	X
Collect PD fluctuation diaries	(X)		X		X		X		X		X

Procedure/Assessments	Week										
	40/e0[a]	44/e4	48/e8[b]	52/e12	56/e16[b]	60/e20	64/e24[b]	68/e28	72/e32[b]	76/e36	80/e40[b]
Dispense PD fluctuation diaries		X		X		X		X		X	
Infusion of study drug	X	X	X	X	X	X	X	X	X	X	
Glasgow Coma Scale[h]	X	X	X	X	X	X	X	X	X	X	X
Adverse events[i]	X	X	X	X	X	X	X	X	X	X	X
Concomitant medications[j]	X	X	X	X	X	X	X	X	X	X	X

a. Assessments that are put in parentheses are done at the Week 40 visit in Study 2553 or earlier (informed consent). With the exception of informed consent, no procedures are required specifically for the extension study; however, procedures scheduled for Week 40 in Study 2553 must be performed in accordance with the protocol.
b. At Weeks 48/e8, 56/e16, 64/e24 and 72/e32, no PD medications are taken after 6:00 PM on the night before the assessments and no long-acting PD medications are taken on the day before the assessments. Subjects refrain from eating any high-protein foods on the morning of the assessments.
c. Informed consent must be obtained before any extension study-specific procedures or assessments are performed.
d. T1-weighted as well as T2-weighted and FLAIR 3T MRI are to be completed in Primary Stage subjects within 2 hours of a gadolinium contrast-containing test infusion at Week 80/e40 (±1 week). If possible, the test infusion and MRI should be done 1 week before the visit.
e. For times of assessment of vital signs, see protocol.
f. Brief physical examination, targeted, at the Investigator’s discretion, to identify changes from baseline and from the previous assessment.
g. Hematology, serum chemistry, urinalysis, pregnancy test. For individual tests, see protocol.
h. Performed before infusion, 30 minutes into the infusion, and after completion of the infusion.
i. All AEs occurring during the study until 28 days after the last dose of GDNF are recorded on the AE pages of the CRF.
j. Concomitant medications: from study entry and until Week 80/e40 or early discontinuation are recorded. All over-the-counter or prescription medications, vitamins and/or herbal supplements are recorded in the CRF with their indications.

Table 2. Schedule of Events - Pilot Extension

Procedure/Assessments	Week in Pilot Extension																				
	e2-0[a]	e2-4	e2-8	e2-12	e2-16[b]	e2-20	e2-24	e2-28	e2-32[b]	e2-36	e2-40	e2-44	e2-48	e2-52	e2-56[b]	e2-60	e2-64	e2-68	e2-72	e2-76	e2-80[b]
Informed consent[c]	(X)																				
MRI[d]	X																				X
Vital signs[e]	X	X	X	X	X	X	X	X	X	X	X	X	X	X	X	X	X	X	X	X	X
Weight and height							X								X						X
Physical examination[f]																					X
Port review	X	X	X	X	X	X	X	X	X	X	X	X	X	X	X	X	X	X	X	X	X
ECG																					X
Laboratory assessment[g]																					X
Anti-GDNF serum antibody levels and GDNF plasma concentrations							X						X						X		
UPDRS part II and part III in OFF state	(X)				X				X						X						X
Timed walking test in OFF state	(X)				X				X						X						X
Timed tapping test in OFF state	(X)				X				X						X						X
Levodopa challenge	(X)				X				X						X						X
UPDRS in ON state	(X)				X				X						X						X
Timed walking test in ON state	(X)				X				X						X						X
Timed tapping test in ON state	(X)				X				X						X						X
MoCA and MDRS	(X)																				X
UPSIT	(X)																				X
NMSS	(X)										X										X
BDI	(X)																				X
QUIP	(X)				X				X				X				X				X

Procedure/Assessments	Week in Pilot Extension																				
	e2-0[a]	e2-4	e2-8	e2-12	e2-16[b]	e2-20	e2-24	e2-28	e2-32[b]	e2-36	e2-40	e2-44	e2-48	e2-52	e2-56[b]	e2-60	e2-64	e2-68	e2-72	e2-76	e2-80[b]
Direct questioning of impulsivity, mood, falls and freezing for recording in case notes	X	X	X	X	X	X	X	X	X	X	X	X	X	X	X	X	X	X	X	X	X
Collect PD fluctuation diaries	(X)				X				X						X						X
Dispense PD fluctuation diaries				X				X						X						X	
Infusion of study drug		X	X	X	X	X	X	X	X	X	X	X	X	X	X	X	X	X	X	X	
Glasgow Coma Scale[h]	X	X	X	X	X	X	X	X	X	X	X	X	X	X	X	X	X	X	X	X	X
Adverse events[i]	X	X	X	X	X	X	X	X	X	X	X	X	X	X	X	X	X	X	X	X	X
Concomitant medications[j]	X	X	X	X	X	X	X	X	X	X	X	X	X	X	X	X	X	X	X	X	X

a. Visit e2-0 takes place at the same time as (or within 1 week of) the Week 80/e40 visit. Assessments in parentheses are done at Week 80/e40 or earlier (informed consent). The safety assessments and outcome measures taken at Week 80/e40 serve as the baseline for this part of the study and do not need to be repeated at Visit e2-0.

b. At Weeks e2-16, e2-32, e2-56 and e2-80, no PD medications are taken after 6:00 PM on the night before the assessments and no long-acting PD medications are taken on the day before the assessments. Subjects refrain from eating any high-protein foods on the morning of the assessments.

c. Informed consent must be obtained before any extension study-specific procedures or assessments are performed.

d. T1-weighted as well as T2-weighted and FLAIR 3T MRI are to be completed in Primary Stage subjects within 2 hours of a gadolinium contrast-containing test infusion at Weeks e2-0 and e2-80 (±1 week). At the Week e2-80 visit, the test infusion and MRI should be done 1 week before the visit, if possible.

e. For times of assessment of vital signs, see protocol.

f. Brief physical examination, targeted, at the Investigator's discretion, to identify changes from baseline and from the previous assessment.

g. Hematology, serum chemistry, urinalysis, pregnancy test. For individual tests, see protocol.

h. Performed before infusion, 30 minutes into the infusion, and after completion of the infusion.

i. All AEs occurring during the study until 28 days after the last dose of GDNF are recorded on the AE pages of the CRF.

j. Concomitant medications from study entry and until Week e2-80 or early discontinuation are recorded. All over-the-counter or prescription medications, vitamins and/or herbal supplements are recorded in the CRF with their indications.

Table 3. Schedule of Events - Supplemental Extension

Procedure/Assessments	Week in Supplemental Extension													
	e3-0[a]	e3-4	e3-8	e3-12	e3-16[b]	e3-20	e3-24	e3-28	e3-32[b]	e3-36	e3-40	e3-44	e3-48[c]	Last Study Visit Additional procedures/assessments[c]
Informed consent[d]	(X)													
Vital signs[e]	X	X	X	X	X	X	X	X	X	X	X	X	X	
Port review	X	X	X	X	X	X	X	X	X	X	X	X	X	
Laboratory assessment[f]														X
Anti-GDNF serum antibody levels and GDNF plasma concentrations														X
UPDRS part II and part III in OFF state	(X)				X				X					
Timed walking test in OFF state	(X)				X				X					
Timed tapping test in OFF state	(X)				X				X					
Levodopa challenge	(X)				X				X					
UPDRS in ON state	(X)				X				X					
Timed walking test in ON state	(X)				X				X					
Timed tapping test in ON state	(X)				X				X					
MoCA and MDRS														X
BDI	(X)													X
QUIP	(X)				X				X					X
Direct questioning of impulsivity, mood, falls and freezing for recording in case notes	X	X	X	X	X	X	X	X	X	X	X	X	X	
Collect PD fluctuation diaries	(X)				X				X					
Dispense PD fluctuation diaries				X				X						
Infusion of study drug	X	X	X	X	X	X	X	X	X	X	X	X	X	
Glasgow Coma Scale[g]	X	X	X	X	X	X	X	X	X	X	X	X	X	
Adverse event[h]	X	X	X	X	X	X	X	X	X	X	X	X	X	
Concomitant medications[i]	X	X	X	X	X	X	X	X	X	X	X	X	X	

a. Visit e3-0 takes place approximately 1-2 weeks after Week 80/e40 (Primary Stage subjects) or Week e2-80 (Pilot Stage subjects). Assessments in parentheses are done at Week 80/e40 (Primary Stage subjects) or Week e2-80 (Pilot Stage subjects) or earlier

(informed consent). The safety assessments and outcome measures taken at Week 80/e40 or Week e2-80 (whichever is applicable) serve as the baseline for this part of the study and do not need to be repeated at Visit e3-0.

b. At Weeks e3-16 and e3-32, no PD medications are taken after 6:00 PM on the night before the assessments and no long-acting PD medications are taken on the day before the assessments. Subjects refrain from eating any high-protein foods on the morning of the assessments.
c. At the last study visit in December 2016 (which occurs at Week e3-48 or earlier), the subject undergoes all procedures and assessments scheduled for the respective visit reached by the subject as per the visit schedule. In addition, regardless of the assessments scheduled for the respective visit, MoCA, MDRS, QUIP and BDI assessments are performed and samples are obtained for laboratory assessment and determination of anti-GDNF serum antibody levels and GDNF plasma concentrations. The same approach to final assessments should be taken, if possible, for any subjects who discontinue the Supplemental Extension early.
d. Informed consent must be obtained before any extension study-specific procedures or assessments are performed.
e. For times of assessment of vital signs, see protocol.
f. Hematology, serum chemistry, urinalysis, pregnancy test. For individual tests, see protocol.
g. Performed before infusion, 30 minutes into the infusion, and after completion of the infusion.
h. All AEs occurring during the study until 28 days after the last dose of GDNF are recorded on the AE pages of the CRF.
i. Concomitant medications: from study entry and until last study visit are recorded. All over-the-counter or prescription medications, vitamins and/or herbal supplements are recorded in the CRF with their indications.

<u>Abbreviations used in the tables</u>

AE: Adverse event; BDI: Beck Depression Inventory; CRF: Case report form; ECG: Electrocardiogram; EQ-5D: EuroQOL 5-Dimensional Scale; FLAIR: Fluid-attenuated inversion recovery; FrSBe: Frontal Systems Behavioural Scale; GDNF: Glial cell line-derived neurotrophic factor; MDRS: Mattis Dementia Rating Scale; MoCA: Montreal Cognitive Assessment; MRI: Magnetic resonance imaging; NMSS: Non-Motor Symptom Scale; PD: Parkinson's disease; PDQ-39: Parkinson's Disease Questionnaire-39; QUIP: Questionnaire for Impulsive-Compulsive Disorders in Parkinson's Disease; SNAQ: Simplified Nutritional Appetite Questionnaire; UPDRS: Unified Parkinson's Disease Rating Scale; UPSIT: University of Pennsylvania Smell Identification Test.

3.1 Sample Size Considerations

This is an open-label extension study. No power calculations were performed. The study was open to all 41 subjects who completed Study 2553 and met all eligibility criteria specified in the protocol.

3.2 Randomization

This study is not randomized. All subjects enrolled receive active treatment: 600 µL of 0.20 µg/µL GDNF in artificial cerebrospinal fluid (aCSF) per putamen every 4 weeks, regardless of their treatment assignment in Study 2553.

4.0 Study Endpoints

4.1 Efficacy Endpoints

Efficacy endpoints are analyzed only for the period up to the end of the Initial Extension. Efficacy data collected during the Pilot Extension and Supplemental Extension are included in the subject listings.

Depending on the individual endpoint, efficacy analyses are performed for both the ITT Primary Population and the ITT Overall Population, or for the ITT Overall Population alone (see Section 9.7 for details). The primary analysis is the analysis of the primary efficacy endpoint in the ITT Primary Population.

4.1.1 Primary Efficacy Endpoint

The primary efficacy endpoint of the study is:

- Percentage change from baseline to Week 80/e40 in the practically defined OFF state UPDRS motor score (part III).

4.1.2 Secondary Efficacy Endpoints

The secondary efficacy endpoints are:

- Change from baseline to Week 80/e40 in OFF state UPDRS motor score (part III).
- Change and percentage change from baseline to Week 80/e40 in:
    - UPDRS motor score (part III) in the ON state (following a levodopa challenge).
    - UPDRS activities of daily living (ADL) score (part II) in the OFF state and in the ON state.
    - UPDRS total score (sum of motor + ADL scores) in the OFF state and in the ON state.
- Change from baseline to Week 40/e0 for the GDNF/GDNF group compared to change from baseline to Week 80/e40 for the placebo/GDNF group in:
    - OFF state UPDRS motor score (part III).
    - OFF state UPDRS ADL score (part II).
    - OFF state UPDRS total score (sum of motor + ADL scores).
- Change from baseline to Week 80/e40 for the GDNF/GDNF group compared to change from baseline to Week 40/e0 for the placebo/GDNF group in:
    - OFF state UPDRS motor score (part III).
    - OFF state UPDRS ADL score (part II).
    - OFF state UPDRS total score (sum of motor + ADL scores).
- Change from baseline to Week 80/e40 in PD diary ratings:

 - Total OFF time per day.
 - Total good-quality ON time per day (ON without dyskinesias or ON with non-troublesome dyskinesias).
 - ON time per day with troublesome dyskinesias.
- Treatment response based on the following criteria:
 - Decrease from baseline to Week 80/e40 by ≥10 points in OFF state UPDRS motor score (part III).
 - Increase from baseline to Week 80/e40 by ≥1 hour in total good-quality ON time per day (ON without dyskinesias or ON with non-troublesome dyskinesias).
 - Both of the above criteria, i.e. decrease from baseline to Week 80/e40 by ≥10 points in OFF state UPDRS motor score (part III) and increase from baseline to Week 80/e40 by ≥1 hour in total good-quality ON time per day (ON without dyskinesias or ON with non-troublesome dyskinesias).

4.1.3 Supplementary Efficacy Endpoints

The following are supplementary efficacy endpoints:

- Change from baseline to Week 80/e40 in supplementary motor, non-motor, medication, and quality of life endpoints, including the following
 - Timed walking test (OFF and ON state).
 - Timed tapping test (OFF and ON state).
 - Non-Motor Symptom Assessment Scale for PD (NMSS).
 - Parkinson's Disease Questionnaire-39 (PDQ-39).
 - EuroQOL 5-Dimensional Scale (EQ-5D).
 - Simplified Nutritional Appetite Questionnaire (SNAQ).
 - Total daily dose of levodopa and total daily levodopa equivalent dose.

4.2 Imaging Endpoints

The analysis of imaging endpoints is restricted to the ITT Primary Population, unless otherwise specified.

The following are analyzed as imaging endpoints:

- Change from baseline to Week 80/e40 in volume of distribution of infusate as determined by contrast-enhanced T1-weighted MRI.
- Change from baseline to Week 80/e40 in volume of interest (VOI) coverage and total putamenal coverage as determined by contrast-enhanced T1-weighted MRI.
- Correlation between primary study endpoint and VOI coverage and total putamenal coverage at baseline as determined by contrast-enhanced T1-weighted MRI.
- Correlation between primary study endpoint and change from baseline to Week 40/e0 in ^{18}F-DOPA uptake as determined by PET scan. This analysis will be done for the ITT Overall Population in addition to the ITT Primary Population.
- Correlation between baseline OFF state UPDRS motor score (part III) and baseline ^{18}F-DOPA uptake as determined by PET scan. This analysis will be done for the ITT Overall Population only.

- Correlation between baseline OFF state UPDRS ADL score (part II) and baseline ^{18}F-DOPA uptake as determined by PET scan. This analysis will be done for the ITT Overall Population only.
- Correlation between Week 40/e0 OFF state UPDRS motor score (part III) and Week 40/e0 ^{18}F-DOPA uptake as determined by PET scan. This analysis will be done for the ITT Overall Population only.
- Correlation between Week 40/e0 OFF state UPDRS ADL score (part II) and Week 40/e0 ^{18}F-DOPA uptake as determined by PET scan. This analysis will be done for the ITT Overall Population only.
- Correlation between Week 80/e40 OFF state UPDRS motor score (part III) and Week 40/e0 ^{18}F-DOPA uptake as determined by PET scan. This analysis will be done for the ITT Overall Population only.
- Correlation between Week 80/e40 OFF state UPDRS ADL score (part II) and Week 40/e0 ^{18}F-DOPA uptake as determined by PET scan. This analysis will be done for the ITT Overall Population only.

4.3 Safety Endpoints

The analysis of safety endpoints includes all subjects enrolled who received at least one dose of open-label study medication (Safety Overall Population; see Section 6.2.1). Safety endpoints are reported for the entire study period including the Initial Extension, Pilot Extension and/or Supplemental Extension as applicable (see Schedules of Events in Section 3.0 for assessment time points). Due to the temporal proximity of the study start to the end of Study 2553, all adverse events (AEs) are considered treatment-emergent AEs (TEAEs).

The following are analyzed as safety endpoints:

- Frequency of TEAEs (all TEAEs and TEAEs related to study drug) during the study period.
- Frequency of device-related TEAEs during the study period.
- Frequency of dyskinesias, falls, adverse changes in mood, and impulsivity reported as TEAEs during the study period (AEs of special interest, AESIs).
- Adverse changes in MRI findings as captured by AE reporting.
- Results of routine laboratory blood tests (hematology, serum chemistry) and urinalysis performed during the study period
- Frequency of subjects with anti-GDNF serum antibodies during the study period.
- Change from baseline in the Questionnaire for Impulsive-Compulsive Disorders in Parkinson’s Disease (QUIP) as assessed during the study period.
- Change from baseline in the Montreal Cognitive Assessment (MoCA) as assessed during the study period.
- Change from baseline in the Mattis Dementia Rating Scale (MDRS) as assessed during the study period.

The following other safety data are also analyzed or listed:

- Exposure to study medication.
- Plasma GDNF concentrations.
- Physical examination.
- Port symptoms.

- Vital signs.
- Weight and height.
- Electrocardiogram (ECG).
- Glasgow Coma Scale.
- Stroop test.
- Frontal Systems Behavioural Scale (FrSBe).
- Deary-Liewald reaction time (RT).
- Verbal fluency assessment.
- Beck Depression Inventory (BDI).
- University of Pennsylvania Smell Identification Test (UPSIT).

5.0 Definitions

Adverse changes in MRI findings

Adverse changes in MRI findings as captured by AE reporting are defined as a Medical Dictionary for Regulatory Activities (MedDRA) preferred term of “Nuclear magnetic resonance imaging brain abnormal” (MedDRA higher level term “Central nervous system imaging procedures”).

Adverse events of special interest

TEAEs including dyskinesias, falls, adverse changes in mood, and impulsivity are considered AESIs in this study. AESIs are defined as follows.

Dyskinesias

Dyskinesia is defined as any of the following MedDRA preferred terms:

- Dyskinesia
- Chorea
- Ballism
- Athetosis
- Dystonia

Falls

A fall is defined as a MedDRA preferred term of “Fall.”

Adverse changes in mood / impulsivity

Prior to database lock, all AE data were reviewed by a qualified physician to identify any relevant MedDRA preferred terms for the categories “Adverse changes in mood” and “Impulsivity”. The MedDRA preferred terms found are listed in Appendix 3.

Age

The following SAS® code will be used to calculate subject age (years) at baseline in Study 2553:

Age = floor ((intck(‘month’, birth date, IC date) - (day(IC date) < day(birth date))) / 12),

where intck is a SAS® function counting integer days, birth date is the database variable for date of birth, and informed consent (IC) date is the database variable for initial informed consent date in Study 2553.

Baseline, change from baseline, percentage change from baseline

Baseline values for comparisons with postbaseline values

For efficacy and imaging analyses, for comparisons of postbaseline values to baseline values, the baseline value is defined as the baseline value from Study 2553.

For laboratory data, Glasgow Coma Scale and QUIP analyses, the baseline value is defined as the value collected at Week 40/e0. If data was collected at both Week 40 from Study 2553 and Week e0, then the latter value will be used as the baseline value.

For all other safety analyses, the baseline value is defined as the baseline value from Study 2553.

Pre-infusion baseline values for comparison with values during or after infusion

For comparisons of values during or after infusion with pre-infusion values, the baseline is the pre-infusion value. This type of comparison applies to vital sign assessments.

Change from baseline

Change from baseline is defined as (postbaseline value – value at baseline).

Percentage change from baseline

Percentage change from baseline is defined as [(postbaseline value – value at baseline) / value at baseline] * 100%.

Body mass index

Body mass index (BMI) is calculated as kg/m^2 where kg is weight in kilograms and m^2 is height in meters, squared.

Catheter positioning accuracy

There are 4 catheters per subject (2 catheters per putamen). Catheter positioning accuracy is assessed at repeat surgeries by measurement of the actual target versus the planned target in mm for the tip of each catheter. This parameter is not derived, but is located in the Post-Operative CT Scan CRF as “Distance between planned target and actual target (mm)” for catheters #1-4 for each subject.

Completion of study

A subject who completes the study is identified as such on the End of Study CRF in the database. This relates to the completion of the Initial Extension only.

Concomitant medication

Concomitant medications (Parkinson’s disease medications and other medications) are defined as any medications ongoing at the start of open-label study medication dosing or with a start date on or after the first open-label study medication dose date. In the case of missing or partial dates, any medications that could have been ongoing at the start of open-label study medication dosing or could have started on or after the first open-label study medication dose date are assumed to be concomitant.

Duration of infusion of open-label study medication

Duration of infusion of open-label study medication in minutes is calculated as (infusion end time – infusion start time + 1). Interruptions are not subtracted.

Duration since first Parkinson's disease symptom, duration since Parkinson's disease diagnosis

Duration since PD symptom onset and duration since PD diagnosis in years in Study 2553 is calculated as (screening Visit 1 date – initial PD symptom/diagnosis date + 1)/365.25. If the day only of PD symptom/diagnosis date is missing, then the 1st day of the month is imputed; if the month only is missing or month and day are missing, then January or January 1st is imputed, respectively. PD symptom/diagnosis dates with a missing year are not included in the calculations.

Early termination of study

A subject who terminates the study prior to the completion of the Initial Extension is identified as such on the End of Study CRF in the database; a primary reason for early termination is provided. No early termination data are presented for the Pilot Extension or Supplemental Extension.

Enrolled subject

An enrolled subject is one with a record in the database who meets all of the inclusion/exclusion criteria for Study 2797.

Levodopa challenge dose

The levodopa challenge dose is the dose of levodopa in mg that the subject receives when undergoing a levodopa challenge.

Levodopa dose, total daily levodopa dose

The levodopa dose at baseline and Week 80/e40 is the total daily levodopa dose in mg that the subject is on at the time of the visit.

The actual daily doses of the individual levodopa preparations taken are documented on the Levodopa and Levodopa Equivalent Medications CRF. Since the bioavailability of levodopa preparations differs, specific conversion factors must be used in order to characterize the subject's effective levodopa dose (see Appendix 4). Immediate release preparations taken without concomitant catechol-O-methyl transferase (COMT) inhibitors do not require conversion (conversion factor 1.0). The daily doses of immediate release preparations taken with COMT inhibitors and of controlled release preparations are multiplied by the corresponding conversion factors. The total daily levodopa dose is then calculated by adding together the converted daily doses of all individual levodopa-containing preparations. COMT inhibitor doses are not included in the calculation.

Levodopa equivalent dose, total daily levodopa equivalent dose

Subjects with PD generally take numerous medications to control their symptoms. In order to have a measure of their total medication intake, a levodopa equivalent dose is calculated. Each PD medication, as documented on the Levodopa and Levodopa Equivalent Medications CRF, is multiplied by a specific conversion factor indicating the drug's relative potency with respect to immediate release levodopa unaccompanied by COMT inhibitors (see Appendix 4 for a full list of conversion factors). The total daily levodopa equivalent dose is calculated by adding together the daily levodopa equivalent doses of all individual PD medications. COMT inhibitor doses are not included in the calculation.

Measures of infusion performance

Measures of infusion performance are determined by hemisphere on the basis of contrast-enhanced T1-weighted MRI.

Volume of distribution

Volume of distribution per hemisphere is documented as "Volume of distribution (mL), left" and "Volume of distribution (mL), right" in the Post-Infusion MRI CRF.

Total volume of putamen

Total volume of putamen is documented as “Total volume of putamen (mL), left” and “Total volume of putamen (mL), right” in the Post Randomization MRI Review CRF (source: Baseline and Planning MRI CRF) from Study 2553.

Putamenal volume of distribution

Putamenal volume of distribution is documented as “Volume of distribution (mL), left putamen” and “Volume of distribution (mL), right putamen”. The baseline value is located in the Post Randomization MRI Review CRF from Study 2553. The Week 80/e40 value is located in the Post-Infusion MRI CRF, Extension Week 40.

Total putamenal coverage

Total putamenal coverage is defined as (putamenal volume of distribution / total volume of putamen * 100%). This parameter is derived for each putamen.

Volume of interest

Volume of interest is documented as “Volume of interest (mL), left” and “Volume of interest (mL), right” in the Post Randomization MRI Review CRF (source: Baseline and Planning MRI CRF) from Study 2553.

Volume of interest coverage (absolute)

Absolute VOI coverage is documented as “Volume of interest covered by infusate (mL), left” and “Volume of interest covered by infusate (mL), right”. The baseline value is taken from the Post Randomization MRI Review CRF (source: Post-Infusion MRI CRF, Healing Phase) from Study 2553. The Week 80/e40 value is located in the Post-Infusion MRI CRF, Extension Week 40.

Volume of interest coverage (relative)

Relative VOI coverage is defined as (volume of interest covered by infusate / volume of interest * 100%). This parameter is documented as “Volume of interest covered by infusate (%), left” and “Volume of interest covered by infusate (%), right”. The baseline value is taken from the Post Randomization MRI Review CRF (source: Post-Infusion MRI CRF, Healing Phase) from Study 2553. The Week 80/e40 value is located in the Post-Infusion MRI CRF, Extension Week 40.

Protocol deviations

Protocol deviations are recorded on the protocol deviation form. They are categorized for summarization, applying controlled terminology including inclusion criteria, exclusion criteria, study medication (including overdose), non-study medication, study schedule/visit window, outcome assessment, and other). They are also classified as major or minor, based on whether they potentially impact the outcome of the study. See Appendix 2 for further details. Prior to database lock, the database entries for protocol deviations will be reviewed by an adjudication team (including, at a minimum, the PI, the study statistician and the Chief Medical Officer of MedGenesis Therapeutix) for consistency of the categorizations and classifications. Final determination of the classifications (major or minor) will be made by the Study Sponsor in view of the recommendations made by the adjudication team.

Study day, last visit on study

If the assessment date is prior to the first open-label study medication dose date then the study day is calculated as (assessment date – first open-label study medication dose date); if the assessment date is on or after the first open-label study medication dose date then the study day is calculated as (assessment date – first open-label study medication dose date + 1). Per Clinical Data Interchange Standards Consortium (CDISC) Standard Data Tabulation Model (SDTM) conventions, study Day 1 corresponds to the day of the first open-label study medication dose (ie, Week e0 visit).

The last visit on study is the last study visit attended including the Initial Extension, Pilot Extension and Supplemental Extension.

Total exposure to open-label study medication

Total exposure to open-label study medication in mg is calculated as (number of infusions * 0.240 mg). This calculation assumes that the entire dose was infused at each administration. This will be derived for the Initial Extension, Pilot Extension, Supplemental Extension and overall.

Total good-quality ON time per day

Total good-quality ON time per day is defined as the sum of ON time per day without dyskinesias + ON time per day with non-troublesome dyskinesias from the PD diary, where each half-hour interval checked contributes 30 minutes to the sum.

Treatment-emergent adverse event

Due to the temporal proximity of the start of Study 2797 to the end of Study 2553, all AEs reported during the study period are considered TEAEs, regardless of whether their onset was before, on, or after the first open-label study medication dose date. TEAEs that are present or ongoing at the beginning of Study 2797 (date of consent or date of last visit in Study 2553, if consent was obtained earlier) are considered pre-existing, if the event term, severity, and date and time of onset in Study 2797 are identical to the corresponding information given for the TEAE in Study 2553. If any of these conditions is not met, the TEAE is considered new or worsening.

Treatment group

In the analysis, findings are organized by “treatment group”. The names of the treatment groups reflect the randomized treatment (GDNF or placebo) received in Study 2553 followed by the non-randomized GDNF treatment received in Study 2797. Subjects included in the GDNF/GDNF treatment group are those that received randomized double-blind GDNF in Study 2553, followed by open-label GDNF in Study 2797. Subjects included in the placebo/GDNF treatment group are those that received randomized double-blind placebo in Study 2553, followed by open-label GDNF in Study 2797.

Treatment response

Treatment response is defined based on the change in OFF state UPDRS motor score (part III), total good quality ON time per day, and a composite of both. Specifically, the following response definitions apply:

- OFF state UPDRS motor score (part III): Decrease from baseline to Week 80/e40 by ≥10 points.
- Total good-quality ON time per day (ON without dyskinesias or ON with non-troublesome dyskinesias): Increase from baseline to Week 80/e40 by ≥1 hour.
- Composite: Decrease from baseline to Week 80/e40 by ≥10 points in OFF state UPDRS motor score (part III) and increase from baseline to Week 80/e40 by ≥1 hour in total good quality ON time per day (ON without dyskinesias or ON with non troublesome dyskinesias).

UPDRS score, OFF state total and ON state total

OFF state UPDRS total score is the sum of the OFF state motor score (part III) and the OFF state ADL score (part II).

ON state UPDRS total score is the sum of the ON state motor score (part III) and the ON state ADL score (part II).

Visits and visit windows

Scheduled visits in Study 2553 were as follows:

- Screening Visit 1

- Screening Visit 2
- Surgery and healing phase
- Week 0 / baseline
- [Week 2 prior to Study 2553 Amendment 3]
- Week 4
- [Week 6 prior to Study 2553 Amendment 3]
- Week 8
- [Week 10 prior to Study 2553 Amendment 3]
- Week 12
- [Week 14 prior to Study 2553 Amendment 3]
- Week 16
- [Week 18 prior to Study 2553 Amendment 3]
- Week 20
- [Week 22 prior to Study 2553 Amendment 3]
- Week 24
- [Week 26 prior to Study 2553 Amendment 3]
- Week 28
- [Week 30 prior to Study 2553 Amendment 3]
- Week 32
- [Week 34 prior to Study 2553 Amendment 3]
- Week 36
- [Week 38 prior to Study 2553 Amendment 3]
- Week 40 (Month 9 endpoint)/early termination

Scheduled visits in the Initial Extension of Study 2797 are as follows:

- Week e0 (same as Study 2553 Week 40 visit for many assessments)
- Week 44/e4
- Week 48/e8
- Week 52/e12
- Week 56/e16
- Week 60/e20
- Week 64/e24
- Week 68/e28
- Week 72/e32

- Week 76/e36
- Week 80/e40/early termination

The designation of the visits in the Initial Extension has been modified from the protocol by adding the consecutive week number from Study 2553 baseline to facilitate the interpretaion of the analyses.

In the Initial Extension, the Week 80/e40/early termination visit may occur at any time on or after study Day 1 for early terminators of the study. For subjects who terminate from the study early, early termination assessments are assigned to an earlier scheduled visit using the study day of the early termination visit date. If only the day of the early termination visit date is missing, then the 1st day of the month is imputed. If the early termination visit date has missing month and/or year after the data query process, the early termination assessments are not assigned to an earlier visit.

The early termination visit may occur during a visit period in which a scheduled visit has already occurred. In this case, the visit that is closer to the nominal study day is selected for tabulations and plots by visit.

Scheduled visits for the Pilot Extension of Study 2797 are as follows:

- Week e2-0 (at same time or within 1 week of Week e40)
- Week e2-4
- Week e2-8
- Week e2-12
- Week e2-16
- Week e2-20
- Week e2-24
- Week e2-28
- Week e2-32
- Week e2-36
- Week e2-40
- Week e2-44
- Week e2-48
- Week e2-52
- Week e2-56
- Week e2-60
- Week e2-64
- Week e2-68
- Week e2-72
- Week e2-76
- Week e2-80

Scheduled visits for the supplemental extension are as follows:

- Week e3-0 (1-2 weeks after Week e40 [Primary Stage subjects] or Week e2-80 [Pilot Stage subjects])
- Week e3-4
- Week e3-8
- Week e3-12
- Week e3-16
- Week e3-20
- Week e3-24
- Week e3-28
- Week e3-32
- Week e3-36
- Week e3-40
- Week e3-44
- Week e3-48

The visit windows in Table 4 are applied for analyses of the Initial Extension of Study 2797 in conjunction with data from Study 2553 (see definitions of completion of study, early termination of study, and study day in this section). The visit schedules are distinguished using either database visit labels for Pilot Stage subjects or study day for Primary Stage subjects for Study 2553 visits. The visit schedules are distinguished using study day for all subjects for Study 2797 visits.

Table 4. Visit Windows

Visit	Nominal Study Day	Study Day Range Prior to Study 2553 Amendment 3	Study Day Range After Study 2553 Amendment 3	Study Day Range for Study 2797
Week 0	0	-6-7	-13-14	
Week 2	14	8-21		
Week 4	28	22-35	15-42	
Week 6	42	36-49		
Week 8	56	50-63	43-70	
Week 10	70	64-77		
Week 12	84	78-91	71-98	
Week 14	98	92-105		
Week 16	112	106-119	99-126	
Week 18	126	120-133		
Week 20	140	134-147	127-154	
Week 22	154	148-161		
Week 24	168	162-175	155-182	
Week 26	182	176-189		
Week 28	196	190-203	183-210	
Week 30	210	204-217		
Week 32	224	218-231	211-238	
Week 34	238	232-245		
Week 36	252	246-259	239-259	
Week 38	266	260-273		
Week 40	280	274-287	260-304	
Week e0	1			-13-14
Week 44/e4	29			15-42
Week 48/e8	57			43-70
Week 52/e12	85			71-98
Week 56/e16	113			99-126
Week 60/e20	141			127-154
Week 64/e24	169			155-182
Week 68/e28	197			183-210
Week 72/e32	225			211-238
Week 76/e36	252			239-259
Week 80/e40	281			260-309

The Week 40 and Week 80/e40 visit windows are wider than for other visits since the number and duration of assessments required special arrangements in some cases in order to minimize subject burden. As a consequence, the adjacent windows (Week 36 and Week 76/e36) are smaller.

Other than the Week 40/e0/early termination and Week 80/e40/early termination visits, postbaseline unscheduled visit values are not windowed and are excluded from tabulations by visit. All unscheduled visit values are included in data listings.

6.0 Analysis Populations

Enrolled subjects are defined in Section 5.0.

6.1 Intent-to-Treat Populations

6.1.1 ITT Primary Population

The ITT Primary Population is defined as all enrolled Primary Stage subjects. This population is used for analyses of the primary efficacy endpoint, some secondary efficacy endpoints, and all imaging endpoints. It is also used for tabulation of subject disposition and summaries of demographic and baseline characteristics from Study 2553. Subjects are counted according to their randomized treatment group in Study 2553.

6.1.2 ITT Overall Population

The ITT Overall Population is defined as all enrolled Pilot Stage subjects plus all enrolled Primary Stage subjects. This population is used for analyses of all efficacy endpoints and some correlation imaging endpoints, for tabulation of subject disposition, and for summaries of demographic and baseline characteristics from Study 2553. Subjects are counted according to their randomized treatment group in Study 2553.

6.2 Safety Population

6.2.1 Safety Overall Population

The Safety Overall Population is defined as all enrolled Pilot Stage subjects who received at least one dose of open-label study medication in Study 2797 plus all enrolled Primary Stage subjects who received at least one dose of open-label study medication in Study 2797. This population is used for all safety analyses. Subjects are counted according to the treatment actually received in Study 2553.

7.0 Interim Analyses

No interim analysis is planned for the study.

8.0 Data Review

8.1 Data Handling and Transfer

Data management for this study is performed by PRA. PRA performs data processing according to approved procedures including database specifications, CRF tracking, and dictionary coding and data validation. A quality control of site responses to data queries is also performed.

Data are entered by the investigational site into CRFs, which are entered by PRA into a clinical database built with Oracle Clinical version 4.5.3 and exported as SAS® version 9.4 or higher datasets (SAS Institute, Inc., Cary, NC). Converted datasets are created using SAS® and following CDISC SDTM conventions (v3.1.3 implementation guide v1.3). Derived analysis datasets are generated using SAS® and following standard CDISC Analysis Dataset Model conventions (implementation guide v1.0). Data analyses including summary tables, figures, and listings (TFLs) are produced using SAS®.

No central laboratory is used for this study. Local laboratory results are collected in the CRF in standard units along with clinical significance. Local laboratory reference ranges are collected outside of the CRF and sent to PRA directly.

AEs are coded using MedDRA version 19.0 to assign a system organ class (SOC) and preferred term (PT) to each AE. Concomitant medications are coded to preferred names using the World Health Organization

Drug Dictionary Enhanced (WHODRUG DDE, 2016Mar01). Anatomical Therapeutic Chemical (ATC) classification coding is included.

PRA's data handling and transfer procedures are documented separately in the study specific data management plan.

8.2 Data Screening

Beyond the data screening built into the PRA Data Management Plan, the PRA programming of analysis datasets and TFLs provides additional data screening. Presumed data issues are output into SAS® logs identified by the word “Problem” and extracted from the logs by a SAS® macro and sent to Data Management.

Review of post-freeze TFLs run on the frozen database allows for further data screening prior to database lock. The post-freeze TFLs are discussed with the sponsor and client in a data review meeting to identify any final data issues and seek corrections prior to database lock. Database lock must be approved by the approvers of the SAP.

9.0 Statistical Methods

All analyses use SAS® version 9.4 or higher. Summary tables are organized by treatment group reflecting the randomized treatment (GDNF or placebo) received in Study 2553 followed by the GDNF treatment received in Study 2797 (for definition see Section 5.0). Summary tables and listings for efficacy and imaging analyses will include baseline and Week 40 data from Study 2553. MMRM analyses will include all scheduled postbaseline data from Study 2553 in the model but data from interim visits in Study 2553 will not be summarized in tables or listed. Line graphs will display all data from baseline to Week 80/e40, including all data from Study 2553. Summary tables and listings for safety analyses that use Week 0 from Study 2553 as the baseline value will include both baseline and Week 40 data from Study 2553. With the exception of demographic data and PD history at screening in Study 2553, no other data from the parent study will be included in summary tables or listings. Important CRF data are included in data listings, sorted by treatment group, subject, and by visit within subject.

Data from the Pilot Extension and Supplemental Extension will be listed only, except for exposure data, concomitant medications, new or worsening TEAEs, QUIP, MoCA, MDRS, anti-GDNF serum antibody data, and GDNF plasma concentration data, which will be included in summary tables.

Unless otherwise noted, categorical data are presented using counts and percentages, with the number of subjects in the analysis population by treatment group as the denominator for percentages. Percentages are rounded to one decimal place. Continuous data, unless otherwise noted, are summarized using the number of observations (n), mean, standard deviation (SD), median, minimum, and maximum. Minima and maxima are rounded to the precision of the original value, and means, medians, and 95% confidence intervals (CIs) are rounded to 1 decimal place greater than the precision of the original value. SD is rounded to 2 decimal places greater than the precision of the original value, up to a maximum of 3 decimal places.

Any hypothesis testing is performed with a 2-sided alternative at the level of $\alpha = 0.05$. P-values are presented with 4 decimal places. No adjustments for multiplicity are made. All inferential analyses are for exploratory purposes only.

9.1 Missing Data Methods

9.1.1 Missing or Partial Dates

Missing or partial dates for AEs, concomitant medications, dosing records, Week 40/e0/early termination and Week 80/e40/early termination visits are imputed as described in Section 5.0 (see definitions for TEAE, concomitant medications, and visit windows).

9.1.2 Missing Efficacy Data

For primary and secondary efficacy endpoints, missing data are not imputed.

Details on handling of missing and duplicate PD motor fluctuation diary ratings are described in Section 9.7.2.5.

The handling of missing data for supplementary efficacy endpoints is described in the appropriate parts of Section 9.7.3.

9.1.3 Missing Imaging Data

For imaging endpoints, missing postbaseline PET scan data are imputed using last observation carried forward (LOCF) (ie, the missing value is replaced with the last non-missing postbaseline time point including data from Study 2553 where applicable). No other missing imaging data are imputed.

9.1.4 Missing Data for Questionnaires

There are 2 levels of missing data possible for questionnaires: either the entire instrument was not assessed at a scheduled time point, or one or more individual items on the instrument were left blank. In the former case of an entirely missed assessment, no imputation is performed.

In the latter case of one or more individual items missed, imputation is performed according to the scoring instructions of the instrument. If the scoring instructions do not address individual missing items, then the imputation method depends on the frequency of postbaseline scheduled assessments. For instruments that have multiple postbaseline scheduled time points, individual missing items are imputed using LOCF (ie, the score for the missing item is taken from the last non-missing postbaseline time point including data from Study 2553 where applicable). For instruments that have only one postbaseline scheduled time point and at least 5 individual items in the subscale or scale being scored, individual missing responses are imputed using the average of non-missing scores. An exception to this rule occurs if more than half of the individual items are missing, in which case no imputation is performed and the subscale or scale score is left missing. Finally, for instruments that have only one postbaseline scheduled time point and fewer than 5 individual items (eg, SNAQ), the total score is considered missing if one or more individual response is missing.

Handling of individual missing items for each scale is discussed in the appropriate parts of Section 9.7 and Section 9.9.

9.1.5 Missing Safety Data

No imputation is performed for missing safety data other than questionnaire data.

9.1.6 Special Arrangements for Subject 45

Special arrangements have been made for subject 45 who had a conus injury during Study 2553 that was unrelated to study treatment or device. As a result of this, items 27, 28, 29 and 30 of the UPDRS score could not be completed beyond Week 8. Item 22, although recorded, is considered to be confounded. Therefore, for this subject, these 5 items are excluded from all calculations of the UPDRS motor score (part III) used in the efficacy analyses, and a truncated score including all other items of part III is used instead. UPDRS parts I, II and IV were collected as far as possible, but the data are not included in the related efficacy analyses because they are considered to be confounded due to the injury. Timed walk could not be done after Week 8 and is therefore excluded from the efficacy analyses. PDQ 39 (items 14 to 39), EQ-5D and NMSS were recorded but are considered to be confounded due to the injury and are therefore excluded from the efficacy analyses.

9.2 Subject Disposition

A tabulation of subject disposition for the Initial Extension is provided for the following categories (see Section 6.0 for population definitions):

- Numbers of Pilot Stage subjects who were enrolled in the extension study and were treated.
- Numbers of Primary Stage subjects who were enrolled in the extension study (ITT Primary Population) and were treated.
- Numbers of Pilot Stage + Primary Stage subjects who were enrolled in the extension study (ITT Overall Population) and were treated (Safety Overall Population).

Subject disposition is also tabulated in a similar manner for the Pilot Extension and Supplemental Extension as appropriate.

The number and percentage of subjects who completed the Week 80/e40 visit is summarized for the ITT Primary Population and ITT Overall Population by treatment group and overall, together with the number and percentage of subjects who withdrew from the study prematurely during the Initial Extension and a breakdown of the corresponding primary reasons for early termination. See Section 5.0 for definitions of completion of study and early termination.

Disposition data for all extension parts of Study 2797 are listed by subject, as are population inclusion data (showing which subjects are included in which analysis population). Data for informed consent and inclusion/exclusion criteria are not listed, but are included in SDTM datasets.

9.3 Protocol Deviations

Protocol deviations are presented by treatment group and overall for the ITT Overall Population by deviation category (major and minor deviations) and deviation name (only major deviations), displaying the number and percentage of subjects in each group to which each deviation category and deviation name apply. Protocol deviations are discussed in Section 5.0.

Major and minor protocol deviation data are listed by study stage, treatment group, and subject.

9.4 Demographic and Baseline Characteristics

9.4.1 Demographic Characteristics

The following demographic characteristics from Study 2553 are tabulated by treatment group and overall for the ITT Primary Population and the ITT Overall Population.

- Age (years)
- Age group (< 65, ≥ 65 years)
- Sex (female, male)
- Race (white, black, Asian, Native Hawaiian or other Pacific Islander, American Indian or Alaska Native, other)
- Ethnicity (Hispanic or Latino, not Hispanic or Latino)
- Baseline weight (kg)
- Baseline height (m)
- Baseline BMI (kg/m^2; see definition in Section 5.0)
- National Adult Reading Test (NART) error score (points)

Demographic data from Study 2553 are listed by study stage, treatment group, and subject.

9.4.2 Parkinson's Disease History at Screening

The following PD history items from Study 2553 are tabulated by treatment group and overall for the ITT Primary Population and the ITT Overall Population.

- Duration since first PD symptom (years)
- Duration since PD diagnosis (years)
- Hoehn and Yahr stage in OFF state (0, 1, 1.5, 2, 2.5, 3)
- OFF state UPDRS motor score (part III; points)
- ON state UPDRS motor score (part III; points)
- Total daily levodopa dose (mg)
- Total daily levodopa equivalent dose (mg)
- PD medications as recorded on the Levodopa and Levodopa Equivalent Medications CRF (eg, levodopa preparations, dopamine agonists, COMT inhibitors, MAO-B inhibitors, other)
- Responsiveness to levodopa (ie, percentage change in screening UPDRS motor score [part III] following a levodopa challenge)
- OFF time per day (hours)

PD history data from Study 2553 are listed by study stage, treatment group, and subject.

9.5 Concomitant Medications

Medications received concomitantly with open-label study medication in any extension part of Study 2797 (Initial Extension, Pilot Extension, Supplemental Extension), categorized by ATC class and preferred name according to WHODRUG DDE, are summarized by treatment group for the ITT Primary Population. Separate summaries are presented for concomitant PD medications and other concomitant medications. The number and percentage of subjects using any concomitant medication is displayed together with the number and percentage of subjects using at least one medication within each ATC class and preferred name. See definition of concomitant medications in Section 5.0.

Concomitant medication data from all extension parts of Study 2797 (Initial Extension, Pilot Extension, Supplemental Extension) are listed by study stage, treatment group and subject. Levodopa challenge dosing data are not listed, but are included in SDTM datasets.

9.6 Surgery and Test Infusions

9.6.1 Catheter Trajectories and Positioning Accuracy

Catheter trajectory data and catheter positioning accuracy data from repositioning surgery are listed by study stage, treatment group, and subject. All other surgery data, including data from post-operative CT scans, are not listed, but are included in SDTM datasets.

9.6.2 Contrast-Enhanced Test Infusions with T1-Weighted MRI Prior to Start of Study Medication

Data for all test infusions, including repeat or unscheduled test infusions, are listed by study stage, treatment group, and subject.

9.7 Efficacy Analyses

Unless otherwise specified, efficacy analyses described in this SAP assess change from the baseline value of Study 2553 to Week 80 (Week e40 in the Initial Extension of Study 2797).

The primary efficacy analysis uses the ITT Primary Population. A sensitivity analysis of the primary endpoint is performed using the ITT Overall Population. Analyses of secondary endpoints are performed using the ITT Primary Population and/or ITT Overall Population in a manner similar to the analyses of the primary endpoint. Analyses of supplementary endpoints are performed for the ITT Overall Population.

All efficacy endpoints are tested at the α = 0.05 level, 2-sided without multiplicity adjustment.

End of study/early termination visit data from the Initial Extension of Study 2797 are windowed to the appropriate scheduled visit and are not included in Week 80/e40 scheduled visit data (see Section 5.0 definition of visit windows).

UPDRS motor (part III) and ADL (part II) scores are generally assessed by raters who are blinded to the subject's treatment assignment in Study 2553.

For efficacy endpoints based on the UPDRS, missing data are not imputed.

All UPDRS data from all extension parts of Study 2797 (Initial Extension, Pilot Extension, Supplemental Extension) are listed by study stage, treatment group, and subject for all UPDRS parameters, including OFF and ON state motor score (part III), OFF and ON state ADL score (part II), OFF and ON state UPDRS total score (sum of motor + ADL scores), mentation, behavior, and mood score (part I), and complications of therapy score (part IV). Baseline and Week 40 values from Study 2553 and changes from baseline in each score are also listed.

9.7.1 Analyses of Primary Efficacy Endpoint

9.7.1.1 Primary Analysis: MMRM of Primary Efficacy Endpoint

The percentage change from baseline to Week 80/e40 in the practically defined OFF state UPDRS motor score (part III) is compared between treatment groups for the ITT Primary Population using a mixed-effect model with repeated measures (MMRM). Baseline UPDRS score is a covariate, treatment group and visit and treatment group*visit are fixed effects, and subject within treatment group is a random effect. The covariance matrix is unstructured. The following SAS® code fragment approximates the analysis:

```
proc mixed data=<input>;
    class <usubjid> <trt01p> <visit>;
    model <pchg> = <base> <trt01p> <visit> <trt01p>*<visit>/ ddfm=KR A;
    repeated / type=un subject=<usubjid>(<trt01p>);
    lsmeans <trt01p> / pdiff;
    estimate 'Trt diff at Wk80' <trt01p> 1 -1 <trt01p>*<visit> 0 0 0 0 0 0 0 0 0 1 0 0 0 0 0 0 0 0
    0 -1 / cl;
run;
```

[A] The Kenward and Roger method for calculating the denominator degrees of freedom for tests of fixed effects will be used.

Summary statistics are tabulated by treatment group expressed as least squares means with 95% CIs, difference between least squares means (GDNF/GDNF – placebo/GDNF) with 95% CIs, and corresponding p-value. Subjects who are missing their Week 80/e40 UPDRS motor score (part III) value are counted in the model at all postbaseline time points for which data are present. Summary statistics are displayed by visit for baseline, Week 40/e0, and Week 80/e40, but inferential statistics are presented only for Week

80/Week e40. Scheduled visits included in the model are Week 0 (baseline; see Section 5.0 definition of baseline) and Weeks 8, 16, 24, 32, 40/e0, 48/e8, 56/e16, 64/e24, 72/e32 and 80/e40.

Percentage change from baseline in OFF state UPDRS motor score (part III) over time up to Week 80/e40 is plotted on a line plot displaying mean values and standard error (SE) bars.

An exploratory model was developed to describe the disease progression in PD as a function of time using Parkinson's Progression Markers Initiative (PPMI) data (Pfizer Report PMAR-EQDD-B847a-DP3-570). Baseline severity was a significant covariate in the model showing a decrease in the rate of disease progression as baseline severity increases. The population estimate of disease progression was similar to previously reported values. Using a baseline OFF state UPDRS motor score (part III) of 35, disease progression is predicted to occur at a modest annual rate of 1.56 points (4.5%) per year, translating to an increase of the baseline score by 1.17 points (3.3%) at 9 months and 2.34 points (6.7%) at 18 months. This modelled control will be plotted on the change and percentage change from baseline figures of OFF state UPDRS motor score (part III) for a visual comparison versus both treatment groups.

Individual subject data for OFF state UPDRS motor score (part III) over time up to Week 80/e40 are also plotted on a line plot by treatment group (displayed in separate graphs).

The GDNF/GDNF treatment group is judged superior compared with placebo/GDNF if there is sufficient statistical evidence to reject the following null hypothesis in the direction favorable to GDNF/GDNF:

> *H_0: No significant difference in the percentage change from baseline to Week 80/e40 in the OFF state UPDRS motor score (part III) between GDNF/GDNF and placebo/GDNF*
>
> and accept the alternative hypothesis:
>
> *H_a: A significantly greater percentage decrease (lower UPDRS is better) in the change from baseline to Week 80/e40 in the OFF state UPDRS motor score (part III) for GDNF/GDNF relative to placebo/GDNF*

It is also possible that a significantly greater percentage decrease for placebo/GDNF as compared with GDNF/GDNF is found, in which case placebo/GDNF is judged superior to GDNF/GDNF (ie, the test is 2-sided).

9.7.1.2 Sensitivity Analysis of Primary Efficacy Endpoint

The primary efficacy analysis is repeated for the ITT Overall Population.

9.7.2 Analyses of Secondary Efficacy Endpoints

The analysis populations for the analyses of secondary efficacy endpoints are specified in the following sections.

9.7.2.1 Change From Baseline in OFF State UPDRS Motor Score (Part III)

The change from baseline to Week 80/e40 in OFF state UPDRS motor score (part III) is compared between treatment groups for both the ITT Primary Population and the ITT Overall Population and reported in a manner identical to that for the primary efficacy endpoint using an MMRM. Summary statistics are tabulated by treatment group expressed as least squares means with 95% CIs, difference between least squares means (GDNF/GDNF – placebo/GDNF) with 95% CIs, and corresponding p-value.

Change from baseline in OFF state UPDRS motor score (part III) over time up to Week 80/e40 is plotted on a line plot displaying mean values and SE bars.

9.7.2.2 Change and Percentage Change from Baseline to Week 80/e40 in Other UPDRS Scores

Change and percentage change from baseline to Week 80/e40 are also analyzed for the following other UPDRS scores:

- UPDRS motor score (part III) in the ON state (following a levodopa challenge) (ITT Overall Population).
- UPDRS activities of daily living (ADL) score (part II) in the OFF state (ITT Primary Population and ITT Overall Population) and in the ON state (ITT Overall Population).
- UPDRS total score (sum of motor + ADL scores) in the OFF state (ITT Primary Population and ITT Overall Population) and in the ON state (ITT Overall Population).

The change and percentage change from baseline to Week 80/e40 in each UPDRS score are compared between treatment groups for the respective analysis populations and reported in a manner identical to that for the primary efficacy endpoint using an MMRM. Summary statistics are tabulated by treatment group expressed as least squares means with 95% CIs, difference between least squares means (GDNF/GDNF – placebo/GDNF) with 95% CIs, and corresponding p-value.

Change from baseline in each score over time up to Week 80/e40 is plotted on line plots displaying mean values and SE bars.

9.7.2.3 Change in UPDRS Scores from Baseline to Week 40/e0 for the GDNF/GDNF Group Compared to Change from Baseline to Week 80/e40 for the Placebo/GNDF Group

Change from baseline to Week 40/e0 for the GDNF/GDNF group is compared to change from baseline to Week 80/e40 for the placebo/GDNF group for the following UPDRS scores:

- OFF state UPDRS motor score (part III).
- OFF state UPDRS ADL score (part II).
- OFF state UPDRS total score (sum of motor + ADL scores).

The change in score from baseline to Week 40/e0 for the GDNF/GDNF group is compared to the change in the score from baseline to Week 80/e40 for the placebo/GDNF group for the ITT Overall Population and reported in a manner identical to that for the primary efficacy endpoint using an MMRM. Summary statistics are tabulated by treatment group expressed as least squares means with 95% CIs, difference between least squares means (GDNF/GDNF – placebo/GDNF) with 95% CIs, and corresponding p-value.

9.7.2.4 Change in UPDRS Scores from Baseline to Week 80/e40 for the GDNF/GDNF Group Compared to Change from Baseline to Week 40/e0 for the Placebo/GNDF Group

Change from baseline to Week 80/e40 for the GDNF/GDNF group is compared to change from baseline to Week 40/e0 for the placebo/GDNF group for the following UPDRS scores:

- OFF state UPDRS motor score (part III).
- OFF state UPDRS ADL score (part II).
- OFF state UPDRS total score (sum of motor + ADL scores).

The change in score from baseline to Week 80/e40 for the GDNF/GDNF group is compared to the change in the score from baseline to Week 40/e0 for the placebo/GDNF group for the ITT Overall Population and reported in a manner identical to that for the primary efficacy endpoint using an MMRM. Summary statistics are tabulated by treatment group expressed as least squares means with 95% CIs, difference between least squares means (GDNF/GDNF – placebo/GDNF) with 95% CIs, and corresponding p-value.

9.7.2.5 Change From Baseline in PD Diary Ratings

PD motor fluctuation diary rating data are collected on scheduled visit days at baseline and every 8 weeks in Study 2553 and in the Initial Extension of Study 2797, and at longer intervals in the Pilot Extension and Supplemental Extension of Study 2797. For diary purposes, a day is defined as a 24-hour period broken into half-hour intervals starting with the 06:00 am interval and ending with the 05:30 am interval the following day. Missing PD motor fluctuation diary ratings are not imputed.

A total of 3 diaries per scheduled visit day are completed by subjects. Each diary collects the state that represents the predominant status during each half-hour interval of the 24-hour period (asleep, OFF, ON without dyskinesias, ON with non-troublesome dyskinesias, ON with troublesome dyskinesias). Errors in diary data and multiple responses in the same half-hour interval are defined as missing data. Data for half-hour intervals with errors are not used for analysis, but the remaining data recorded on the diary are valid only if a maximum of 4 errors are present. If 5 or more errors are present in a given diary, then the entire diary is considered invalid and not used for analysis.

Among the valid diaries (up to 3) per subject and scheduled visit, the mean times of any given state over all valid diaries are used for analysis. From these data, total OFF time per day, total good-quality ON time per day, and ON time per day with troublesome dyskinesias are estimated for the subject and scheduled visit, where each half-hour interval checked contributes 30 minutes to the sum (see definitions in Section 5.0).

The change from baseline to Week 80/e40 in PD motor fluctuation diary ratings is compared between treatment groups for both the ITT Primary Population and the ITT Overall Population and reported in a manner identical to that for the primary efficacy endpoint using an MMRM. Parameters include:

- Total OFF time per day
- Total good-quality ON time per day (sum of ON time per day without dyskinesias + ON time per day with non-troublesome dyskinesias)
    - ON time per day without dyskinesias
    - ON time per day with non-troublesome dyskinesias
- ON time per day with troublesome dyskinesias

Summary statistics are displayed by visit for baseline, Week 40/e0, and Week 80/e40, but inferential statistics are presented only for Week 80/Week e40. Scheduled visits included in the model are Week 0 (baseline; see Section 5.0 definition of baseline) and Weeks 8, 16, 24, 32, 40/e0, 48/e8, 56/e16, 64/e24, 72/e32 and 80/e40.

Change from baseline in total OFF time per day and change from baseline in total good-quality ON time per day up to Week 80/e40 are plotted on line plots displaying mean values and SE bars.

PD diary ratings from all extension parts of Study 2797 (Initial Extension, Pilot Extension, Supplemental Extension) are listed by study stage, treatment group, and subject. Diary training, diary dispensation, and diary collection and review are not listed, but are included in SDTM datasets.

9.7.2.6 Treatment Response

Treatment response at Week 80/e40 is compared between treatment groups using Fisher's exact test for the ITT Overall Population based on the following criteria:

- Decrease from baseline to Week 80/e40 by ≥10 points in OFF state UPDRS motor score (part III).
- Increase from baseline to Week 80/e40 by ≥1 hour in total good-quality ON time per day (ON without dyskinesias or ON with non-troublesome dyskinesias).

- Both of the above criteria, i.e. decrease from baseline to Week 80/e40 by ≥10 points in OFF state UPDRS motor score (part III) and increase from baseline to Week 80/e40 by ≥1 hour in total good-quality ON time per day (ON without dyskinesias or ON with non-troublesome dyskinesias).

Treatment response at Week 40/e0 is defined using the same criteria.

Summary statistics and inferential statistics are displayed by visit for Week 40/e0 and Week 80/e40.

UPDRS responder (≥10 points in OFF state UPDRS motor score (part III)) data are also summarized using a shift table by treatment group. Using the number of responders/non-responders at each timepoint, this table compares Week 80/e40 to Week 40/e0.

Absolute change from baseline to Week 40/e0 and Week 80/e40 in OFF state UPDRS motor score (part III) is plotted as frequency distribution plots displaying the number of subjects for any given change by treatment group.

Treatment response in the Initial Extension is listed by study stage, treatment group, and subject.

9.7.3 Analyses of Supplementary Efficacy Endpoints

All analyses of supplementary endpoints are done for the ITT Overall Population.

9.7.3.1 MMRM of Change From Baseline in Timed Walking Test

During the timed walking test, the subject walks as fast as possible 7 meters back and forth including turning. The time to perform this test is recorded for 2 trials in the OFF state and 2 trials in the ON state after levodopa challenge.

The change from baseline to Week 80/e40 in timed walking test is compared between treatment groups for the ITT Overall Population and reported in a manner identical to that for the primary efficacy endpoint using an MMRM. Results are summarized by treatment group expressed as least squares means with 95% CIs, difference between least squares means (GDNF/GDNF – placebo/GDNF) with 95% CIs, and corresponding p-value. Timed walking test parameters are OFF state timed walking test and ON state timed walking test in seconds.

Summary statistics are displayed by visit for baseline, Week 40/e0, and Week 80/e40, but inferential statistics are presented only for Week 80/e40. Scheduled visits included in the model are Week 0 (baseline; see Section 5.0 definition of baseline) and Weeks 8, 16, 24, 32, 40/e0, 48/e8, 56/e16, 64/e24, 72/e32 and 80/e40. The results of the two separate trials per state at each visit are averaged and the mean used for analysis of each state. If only the first trial is completed for a particular state, then the second trial result will be imputed with the worst non-missing second trial result from all subjects for that particular state during the study. If both trials are missing, then the endpoint is not reported for that visit.

Timed walking test data from all extension parts of Study 2797 (Initial Extension, Pilot Extension, Supplemental Extension) are listed by study stage, treatment group, and subject. Changes from baseline in the results are listed.

9.7.3.2 MMRM of Change From Baseline in Timed Tapping Test

During the timed tapping test, the subject alternates tapping the index finger for 20 seconds between 2 points spaced 30 cm apart. The test is performed twice for each hand in the OFF state and twice for each hand in the ON state after levodopa challenge.

The change from baseline to Week 80/e40 in timed tapping test is compared between treatment groups for the ITT Overall Population and reported in a manner identical to that for the primary efficacy endpoint using an MMRM. Results are summarized by treatment group expressed as least squares means with 95% CIs, difference between least squares means (GDNF/GDNF – placebo/GDNF) with 95% CIs, and corresponding

p-value. Timed tapping test parameters are OFF state timed tapping test and ON state timed tapping test in number of taps completed in 20 seconds.

Summary statistics are displayed by visit for baseline, Week 40/e0, and Week 80/e40, but inferential statistics are presented only for Week 80/e40. Scheduled visits included in the model are Week 0 (baseline; see Section 5.0 definition of baseline) and Weeks 8, 16, 24, 32, 40/e0, 48/e8, 56/e16, 64/e24, 72/e32 and 80/e40. The results of the four separate trials per state at each visit are averaged and the mean used for analysis for each state. If any trial is not completed for a particular state, then the trial result will be imputed with the worst non-missing applicable trial result (for example, first left hand trial) from all subjects for that particular state during the study. If all four trials are missing, then the endpoint is not reported for that visit.

Timed tapping test data from all extension parts of Study 2797 (Initial Extension, Pilot Extension, Supplemental Extension) are listed by study stage, treatment group, and subject. Changes from baseline in the results are listed.

9.7.3.3 MMRM of Change from Baseline in NMSS Score

The NMSS is an interview-based scale used to rate non-motor symptoms commonly occurring in PD (developed by the International Parkinson's Disease Non-Motor Group). It is administered with the subject in the ON state. The 30-item scale rates symptoms that occurred in the preceding month in 9 domains (cardiovascular including falls; sleep/fatigue; mood/cognition; perceptual problems/hallucinations; attention/memory; gastrointestinal tract; urinary; sexual function; miscellaneous). Each item is rated from 0 (none) to 3 (severe) for severity and from 1 (rarely) to 4 (very frequent) for frequency. The score for each item is the product of the severity rating multiplied by the frequency. The maximum score for an individual item is 12. The higher the score, the worse the subject's condition. Individual item scores in each domain are summed to give the domain score, and the domains are summed to give the total score. The maximum NMSS total score is 360. Missing individual item scores are imputed using LOCF.

The change from baseline to Week 80/e40 in NMSS data is compared between treatment groups for the ITT Overall Population and reported in a manner identical to that for the primary efficacy endpoint using an MMRM. Results are summarized by treatment group expressed as least squares means with 95% CIs, difference between least squares means (GDNF/GDNF – placebo/GDNF) with 95% CIs, and corresponding p-value. NMSS parameters are the 9 NMSS domains and the NMSS total score.

Summary statistics are displayed by visit for baseline, Week 40/e0, and Week 80/e40, but inferential statistics are presented only for Week 80/e40. Scheduled visits included in the model are Screening Visit 2 from Study 2553 (baseline; see Section 5.0 definition of baseline) and Weeks 12, 24, 40/e0, 52/e12, 64/e24 and 80/e40.

NMSS data from the Initial Extension and Pilot Extension of Study 2797 are listed by study stage, treatment group, and subject.

9.7.3.4 ANCOVA of Change From Baseline in PDQ-39 Score

The PDQ-39 is a self-administered 39-item PD-specific scale that rates symptoms that occurred in the preceding month in the 8 dimensions listed below. Each item is rated from 0 (never) to 4 (always) for frequency. Each dimension is calculated as a scale from 0 to 100 based on the following formulas:

- Mobility = [(Sum of Scores of questions 1 – 10)/ (4*10)] *100
- Activities of Daily Living = [(Sum of Scores of questions 11 – 16)/(4*6)]*100
- Emotional Well Being = [(Sum of Scores of questions 17 – 22)/(4*6)]*100
- Stigma = [(Sum of Scores of questions 23 – 26)/(4*4)]*100
- Social Support = [(Sum of Scores of questions 27 – 29)/(4*3)]*100

 - If respondents indicate that they do not have a spouse/partner on question 28, then Social Support = [(Sum of Scores of questions 27 & 29)/(4*2)]*100

- Cognitions = [(Sum of Scores of questions 30 – 33)/(4*4)]*100
- Communication = [(Sum of Scores of questions 34 – 36)/(4*3)]*100
- Bodily Discomfort = [(Sum of Scores of questions 37 – 39)/(4*3)]*100

The total score, or single index, is the average of all 8 dimension scores. The higher the score, the worse the subject's condition. If the response to an individual question is missing, then no score is calculated for that dimension and therefore the single index score cannot be calculated.

Analysis of PDQ-39 data on the ITT Overall Population utilizes an ANCOVA model adjusted for baseline PDQ-39 score. Input data are restricted to observed data at baseline and Week 80/e40 only. PDQ-39 parameters are the 8 PDQ-39 dimensions and the single index (total) PDQ-39 score.

Summary statistics are displayed for Screening Visit 2 (baseline; see Section 5.0 definition of baseline), Week 40/e0, and Week 80/e40. Estimated mean difference (GDNF/GDNF – placebo/GDNF), 95% CIs, and p-values are reported.

PDQ-39 data are listed by study stage, treatment group, and subject.

9.7.3.5 ANCOVA of Change From Baseline in EQ-5D Score

The EQ-5D is a subject self-report measure of quality of life consisting of a questionnaire and a visual analog scale. The questionnaire comprises 5 questions on mobility, self-care, usual activities, pain/discomfort, and anxiety/depression, with three possible answers for each item (1=no problem, 2=moderate problem, 3=severe problem). The visual analog scale serves as an indicator of general health status; the scale ranges from 0 to 100, where 0 indicates worst health and 100 indicates best health. Missing values for individual questions are coded as 9; missing values for the visual analog scale are coded as 999. Missing values are not included in observed data analyses.

EQ-5D questionnaire data are reported using frequency counts and percentages for Screening Visit 2 (baseline; see Section 5.0 definition of baseline), Week 40/e0, and Week 80/e40. Input data are restricted to observed data at baseline, Week 40/e0, and Week 80/e40 only. Parameters are the frequency counts and percentages of subjects with the different answers for each of the 5 questions.

Analysis of EQ-5D visual analog scale data on the ITT Overall Population utilizes an ANCOVA model adjusted for baseline EQ-5D visual analog scale score. Input data are restricted to observed data at baseline and Week 80/e40 only. The parameter is the visual analog scale score. Summary statistics are displayed for Screening Visit 2 from Study 2553 (baseline; see Section 5.0 definition of baseline), Week 40/e0, and Week 80/e40. Estimated mean difference (GDNF/GDNF – placebo/GDNF), 95% CIs, and p-values are reported.

EQ-5D data are listed by study stage, treatment group, and subject.

9.7.3.6 ANCOVA of Change From Baseline in SNAQ Score

The SNAQ is a self-administered 4-question instrument with total scores ranging from 4 to 20 (4=poor appetite, 20=good appetite). The SNAQ has one total score. If an individual question is not answered, then the total score is considered missing.

Analysis of SNAQ data on the ITT Overall Population utilizes an ANCOVA model adjusted for baseline SNAQ score. Input data are restricted to observed data at baseline and Week 80/e40 only.

Summary statistics are displayed for Screening Visit 2 from Study 2553 (baseline; see Section 5.0 definition of baseline), Week 40/e0, and Week 80/e40. Estimated mean difference (GDNF/GDNF – placebo/GDNF), 95% CIs, and p-values are reported.

SNAQ data are listed by study stage, treatment group, and subject.

9.7.3.7 ANCOVA of Change From Baseline in Total Daily Levodopa Dose and Total Daily Levodopa Equivalent Dose

Analysis of change from baseline to Week 80/e40 data on the ITT Overall Population utilizes an ANCOVA model adjusted for baseline total daily levodopa dose and total daily levodopa equivalent dose, respectively (see definitions in Section 5.0). Input data are restricted to observed data at baseline and Week 80/e40 only (no imputation of missing data).

Summary statistics are displayed for Week 0 (baseline; see Section 5.0 definition of baseline), Week 40/e0, and Week 80/e40. Estimated mean difference (GDNF/GDNF – placebo/GDNF), 95% CIs, and p-values are reported.

Levodopa dose data and levodopa equivalent dose data from the Initial Extension are listed by study stage, treatment group, and subject. Separate listings are provided for actual total daily dose of each PD medication and for the total daily dose of levodopa and the total daily dose of levodopa equivalent.

9.8 Imaging Analyses

The analysis populations for imaging analyses are specified in the following sections.

9.8.1 MRI Analyses

All MRI analyses are restricted to the ITT Primary Population since in Pilot Stage subjects, only T2-weighted MRI scans were taken at baseline.

Post-infusion MRI data needed for the imaging analyses described in this section are listed for Primary Stage subjects; although not analyzed or tabulated, corresponding post-infusion MRI data are also listed for Pilot Stage subjects. All other MRI data, including real-time MRI data, are not listed, but are included in SDTM datasets.

9.8.1.1 ANCOVA of Change From Baseline in Volume of Distribution of Infusate as Determined by Contrast-Enhanced T1-Weighted MRI

Analysis of change from baseline to Week 80/e40 in volume of distribution of infusate between treatment groups utilizes an ANCOVA model adjusted for baseline volume of distribution. Input data are restricted to observed data at the end of the healing phase in Study 2553, Week 40/e0, and at Week 80/e40 only. The parameter is the volume of distribution (in mL), separately for left and right hemispheres, as determined by contrast-enhanced T1-weighted MRI.

Summary statistics are displayed for the last test infusion at the end of the healing phase in Study 2553 (baseline; see Section 5.0 definition of baseline), Week 40/e0, and Week 80/e40. Estimated mean difference (GDNF/GDNF – placebo/GDNF), 95% CIs, and p-values are reported.

Volume of distribution data from the Initial Extension and Pilot Extension of Study 2797 are listed by study stage, treatment group, and subject.

9.8.1.2 ANCOVA of Change from Baseline in Volume of Interest Coverage and Total Putamenal Coverage as Determined by Contrast-Enhanced T1-Weighted MRI

Analysis of change from baseline to Week 80/e40 in VOI coverage and total putamenal coverage utilizes an ANCOVA model adjusted for baseline coverage. Input data are restricted to observed data at the end of the healing phase in Study 2553, Week 40/e0, and at Week 80/e40 only. Parameters include VOI coverage as a percentage of total VOI and total putamenal coverage as a percentage of total putamenal volume, separately for left and right putamen and for both putamina combined.

Summary statistics are displayed for the last test infusion at the end of the healing phase in Study 2553 (baseline; see Section 5.0 definition of baseline), Week 40/e0, and Week 80/e40. Estimated mean difference (GDNF/GDNF – placebo/GDNF), 95% CIs, and p-values are reported for each analysis.

VOI coverage (absolute and relative) and total putamenal coverage data from the Initial Extension and Pilot Extension of Study 2797, including underlying data (VOI, putamenal volume of distribution, and total volume of putamen), are listed by study stage, treatment group, and subject.

9.8.2 Correlation Analyses

9.8.2.1 Correlation Between Primary Study Endpoint and Volume of Interest Coverage and Total Putamenal Coverage at Baseline as Determined by Contrast-Enhanced T1-Weighted MRI

These analyses are restricted to the ITT Primary Population since in Pilot Stage subjects, only T2-weighted MRI scans were taken at baseline.

These analyses use non-parametric Spearman rank correlation to explore the relationship between percentage change from baseline to Week 80/e40 in OFF state UPDRS motor score (part III) and both VOI coverage and total putamenal coverage after the last test infusion at the end of the healing phase in Study 2553 by treatment group. Correlations are calculated separately for each treatment group. Parameters include percentage change from baseline to Week 80/e40 in OFF state UPDRS motor score (part III) and both VOI coverage as a percentage of total VOI and total putamenal coverage as a percentage of total putamenal volume, for both putamina combined.

The estimated correlation coefficient, 95% CI, and p-value are tabulated for each analysis and treatment group, and scatterplots are provided.

9.8.2.2 Correlation Between Primary Study Endpoint and Change From Baseline to Week 40/e0 in ^{18}F-DOPA Uptake as Determined by PET Scan

This analysis uses non-parametric Spearman rank correlation to explore the relationship between percentage change from baseline to Week 80/e40 in OFF state UPDRS motor score (part III) and change from baseline to Week 40/e0 in PET imaging of ^{18}F-DOPA uptake by treatment group for both the ITT Primary Population and the ITT Overall Population. Correlations are calculated separately for each treatment group. Parameters include percentage change from baseline to Week 80/e40 in OFF state UPDRS motor score (part III) and change from baseline to Week 40/e0 in ^{18}F-DOPA uptake rate constant determined by PET for each of 5 regions of interest (dorsal caudate nucleus, dorsal anterior putamen, dorsal central/posterior putamen, ventral striatum, and substantia nigra), using the average from both hemispheres for each region.

The estimated correlation coefficient, 95% CI, and p-value are tabulated by treatment group, and scatterplots are provided.

9.8.2.3 Correlation Between Baseline OFF State UPDRS Motor Score (Part III) and Baseline ^{18}F-DOPA Uptake as Determined by PET Scan

This analysis uses non-parametric Spearman rank correlation to explore the relationship between baseline (Week 0) OFF state UPDRS motor score (part III) and baseline (Week 0) PET imaging of ^{18}F-DOPA uptake for the ITT Overall Population. This will be repeated just for the GDNF/GDNF treatment group. Parameters include baseline OFF state UPDRS motor score (part III) and baseline ^{18}F-DOPA uptake rate constant determined by PET for dorsal central/posterior putamen, using the average from both hemispheres.

The estimated correlation coefficient and p-value are provided on a scatterplot, one for all subjects and one for GDNF/GDNF subjects only.

9.8.2.4 Correlation Between Baseline OFF State UPDRS ADL Score (Part II) and Baseline ^{18}F-DOPA Uptake as Determined by PET Scan

This analysis uses non-parametric Spearman rank correlation to explore the relationship between baseline (Week 0) OFF state UPDRS ADL score (part II) and baseline (Week 0) PET imaging of ^{18}F-DOPA uptake for the ITT Overall Population. Correlations are calculated separately for each treatment group. This will be repeated just for the GDNF/GDNF treatment group. Parameters include baseline OFF state UPDRS ADL score (part II) and baseline ^{18}F-DOPA uptake rate constant determined by PET for dorsal central/posterior putamen, using the average from both hemispheres.

The estimated correlation coefficient and p-value are provided on a scatterplot, one for all subjects and one for GDNF/GDNF subjects only.

9.8.2.5 Correlation Between Week 40/e0 OFF State UPDRS Motor Score (Part III) and Week 40/e0 ^{18}F-DOPA Uptake as Determined by PET Scan

This analysis uses non-parametric Spearman rank correlation to explore the relationship between Week 40/e0 OFF state UPDRS motor score (part III) and Week 40/e0 PET imaging of ^{18}F-DOPA uptake for the ITT Overall Population. Correlations are calculated separately for each treatment group. This will be repeated just for the GDNF/GDNF treatment group. Parameters include Week 40/e0 OFF state UPDRS motor score (part III) and Week 40/e0 ^{18}F-DOPA uptake rate constant determined by PET for dorsal central/posterior putamen, using the average from both hemispheres.

The estimated correlation coefficient and p-value are provided on a scatterplot, one for all subjects and one for GDNF/GDNF subjects only.

9.8.2.6 Correlation Between Week 40/e0 OFF State UPDRS ADL Score (Part II) and Week 40/e0 ^{18}F-DOPA Uptake as Determined by PET Scan

This analysis uses non-parametric Spearman rank correlation to explore the relationship between Week 40/e0 OFF state UPDRS ADL score (part II) and Week 40/e0 PET imaging of ^{18}F-DOPA uptake for the ITT Overall Population. Correlations are calculated separately for each treatment group. This will be repeated just for the GDNF/GDNF treatment group. Parameters include Week 40/e0 OFF state UPDRS ADL score (part II) and Week 40/e0 ^{18}F-DOPA uptake rate constant determined by PET for dorsal central/posterior putamen, using the average from both hemispheres.

The estimated correlation coefficient and p-value are provided on a scatterplot, one for all subjects and one for GDNF/GDNF subjects only.

9.8.2.7 Correlation Between Week 80/e40 OFF State UPDRS Motor Score (Part III) and Week 80/e40 ^{18}F-DOPA Uptake as Determined by PET Scan

This analysis uses non-parametric Spearman rank correlation to explore the relationship between Week 80/e40 OFF state UPDRS motor score (part III) and Week 80/e40 PET imaging of ^{18}F-DOPA uptake for the ITT Overall Population. Correlations are calculated separately for each treatment group. This will be repeated just for the GDNF/GDNF treatment group. Parameters include Week 80/e40 OFF state UPDRS motor score (part III) and Week 80/e40 ^{18}F-DOPA uptake rate constant determined by PET for dorsal central/posterior putamen, using the average from both hemispheres.

The estimated correlation coefficient and p-value are provided on a scatterplot, one for all subjects and one for GDNF/GDNF subjects only.

9.8.2.8 Correlation Between Week 80/e40 OFF State UPDRS ADL Score (Part II) and Week 80/e40 ^{18}F-DOPA Uptake as Determined by PET Scan

This analysis uses non-parametric Spearman rank correlation to explore the relationship between Week 80/e40 OFF state UPDRS ADL score (part II) and Week 80/e40 PET imaging of ^{18}F-DOPA uptake for the

ITT Overall Population. Correlations are calculated separately for each treatment group. This will be repeated just for the GDNF/GDNF treatment group. Parameters include Week 80/e40 OFF state UPDRS ADL score (part II) and Week 80/e40 ^{18}F-DOPA uptake rate constant determined by PET for dorsal central/posterior putamen, using the average from both hemispheres.

The estimated correlation coefficient and p-value are provided on a scatterplot, one for all subjects and one for GDNF/GDNF subjects only.

9.9 Safety Analyses

Unless otherwise specified, safety analyses described in this SAP assess the safety findings obtained in the extension Study 2797 for the Safety Overall Population. Data collected at Week 40 in Study 2553 is used as the baseline, where a baseline value was not obtained at Week e0 of the extension study.

No imputation is performed for missing safety data other than questionnaire data, as described below.

9.9.1 Study Medication Exposure

Study medication exposure data include number of infusions received and total GDNF exposure in mg, assuming the entire dose was infused at each administration (see definition of total exposure in Section 5.0). These data are presented by treatment group, separately for each extension part of Study 2797 (Initial Extension, Pilot Extension, Supplemental Extension) and overall in Study 2797.

Infusion details per study medication visit include duration of infusion (minutes) and any infusion interruptions/early terminations (yes/no; see definition of duration of infusion in Section 5.0). These data are summarized by treatment group by visit, including the Initial Extension, Pilot Extension and Supplemental Extension infusion visits.

Study medication exposure data from all parts of Study 2797 (Initial Extension, Pilot Extension, Supplemental Extension), along with infusion details, are listed by study stage, treatment group, and subject.

9.9.2 Adverse Events

AEs are collected throughout the study until 28 days after the last dose of study medication. Due to the temporal proximity of the start of Study 2797 to the end of Study 2553, all AEs reported during Study 2797 are considered TEAEs, regardless of whether their onset was before, on, or after the first open-label study medication dose date. TEAEs that are present or ongoing at the beginning of Study 2797 (date of consent or date of last visit in Study 2553, if consent was obtained earlier) are considered pre-existing, if the event term, severity, and date and time of onset in Study 2797 are identical to the corresponding information given for the TEAE in Study 2553. If any of these conditions is not met, the TEAE is considered new or worsening. Only new or worsening TEAEs are tabulated. Pre-existing TEAEs are listed. Unless otherwise specified, in the text below and in the table shells for summary tables, the term “TEAE” denotes “new or worsening TEAE”.

9.9.2.1 All Adverse Events

A summary of all TEAEs reported from all extension parts of Study 2797 (Initial Extension, Pilot Extension, Supplemental Extension) is presented by treatment group and overall. The summary includes the following categories:

- Overall summary of TEAEs
- TEAEs by MedDRA SOC and PT
- TEAEs experienced by at least 3 subjects in any treatment group by PT (number of subjects and number of events)
- TEAEs by MedDRA SOC, PT, and maximum severity

- Serious TEAEs by MedDRA SOC and PT
- Study medication-related TEAEs by MedDRA SOC and PT
- Serious study medication-related TEAEs by MedDRA SOC and PT
- Device-related TEAEs by MedDRA SOC and PT
- Serious device-related TEAEs by MedDRA SOC and PT

A summary of all TEAEs reported from the Initial Extension (where the AE start date is before or on the date of the Week 80/e40 visit) is presented by treatment group and overall. The summary includes the following categories:

- Overall summary of TEAEs
- TEAEs experienced by at least 3 subjects overall by PT
- Serious TEAEs by MedDRA SOC and PT

A summary of all TEAEs reported from the Pilot and Supplemental Extensions (where the AE start date is after the date of the Week 80/e40 visit) is presented by treatment group and overall. The summary includes the following categories:

- Overall summary of TEAEs
- TEAEs experienced by at least 3 subjects overall by PT
- Serious TEAEs by MedDRA SOC and PT

Except where specified, counting is by subject, not event, and subjects are only counted once within each SOC or PT. Sorting is alphabetically by SOC and then alphabetically for PT.

All TEAEs are listed by study stage, treatment group, and subject; pre-existing TEAEs are flagged.

9.9.2.2 Adverse Events of Special Interest

Four categories of treatment-emergent AESIs will be analyzed: dyskinesias; falls; adverse changes in mood; and impulsivity (see definitions in Section 5.0). AESIs are presented by category and PT by treatment group and overall.

AESIs are listed by category, study stage, treatment group, and subject.

9.9.3 Port Symptoms

Evaluation of port symptoms includes the following categories:

- No skin reaction
- Redness with slight swelling
- Redness, moistness and moderate swelling with tissue granulation
- Overt infection

Port symptoms are listed by study stage, treatment group, and subject.

9.9.4 Laboratory Data

Clinical laboratory evaluations (hematology, serum chemistry, and urinalysis) are performed at Weeks 44/e4, 56/e16, 68/e8 and 80/e40, e2-80, and the last study visit in the Supplemental Extension. Laboratory parameters tested are listed in Table 5.

Table 5. Laboratory Parameters

Hematology	Serum Chemistry	Urinalysis	Other
RBC count<br>Hematocrit<br>Hemoglobin<br>MCH<br>MCHC<br>MCV<br>Platelet count<br>WBC count<br>WBC differential (basophils, eosinophils, lymphocytes, monocytes and neutrophils)	Alkaline phosphatase<br>ALT<br>Total bilirubin<br>Creatinine<br>Urea<br>eGFR<br>Albumin<br>Glucose<br>Potassium<br>Sodium	Color<br>Appearance<br>pH<br>Glucose<br>Ketones<br>Nitrite<br>Microscopy	Pregnancy test

Postbaseline hematology and serum chemistry results rated clinically significant by the investigator are summarized with the direction of significance indicated (high or low). Results for eGFR are listed only. For ALT and total bilirubin, any results recorded in the format <X or >X are included in tables as a value of X. These results are listed in the original format. Urinalysis parameters are listed only.

Hematology, serum chemistry, and urinalysis data from all extension parts of Study 2797 (Initial Extension, Pilot Extension, Supplemental Extension) are listed by study stage, treatment group, and subject with values outside of the normal range and clinically significant values flagged. Pregnancy test data are included in SDTM datasets only.

9.9.5 Anti-GDNF Serum Antibodies

The data for anti-GDNF serum antibodies (this section) and plasma GDNF concentrations (Section 9.9.6 below) will not be available until after database lock. In case of a substantial delay, an addendum to the CSR may be written at a later time containing these analyses.

Anti-GDNF binding and neutralizing serum antibody data are reported for screening from Study 2553 (baseline), Weeks 40/e0, 44/e4, 56/e16, 68/e28 and 80/e40; e2-24, e2-48 and e2-72; and the last study visit in the Supplemental Extension. Anti-GDNF serum antibody data are summarized by treatment group with number and percentage of subjects in each category (positive, negative, or not done) by visit. Summary data are also provided for subjects who are positive at any postbaseline visit in Study 2553 or Study 2797 and those who are positive at more than one postbaseline visit in Study 2553 or Study 2797. If a subject has a repeat sample, then the worse result is used in the analysis. A positive result is considered worse than a negative result.

Anti-GDNF binding and neutralizing serum antibody data from all extension parts of Study 2797 (Initial Extension, Pilot Extension, Supplemental Extension) are listed by study stage, treatment group, and subject.

9.9.6 Plasma GDNF Concentrations

Plasma GDNF concentrations are reported for screening from Study 2553 (baseline), Weeks 40/e0, 44/e4, 56/e16, 68/e28 and 80/e40; e2-24, e2-48 and e2-72; and the last study visit in the Supplemental Extension.

Plasma GDNF concentrations are summarized by treatment group by comparing postbaseline to baseline values using summary statistics for changes from baseline. Values below the limit of quantitation are not included in summary statistics. If a subject has a repeat sample, then the higher result is used in the analysis.

Plasma GDNF concentration data from all extension parts of Study 2797 (Initial Extension, Pilot Extension, Supplemental Extension) are listed by extension part, treatment group, and subject.

9.9.7 Physical Examination

A brief physical examination is conducted at Week 80/e40 and Week e2-80. Physical examination data are listed by study stage, treatment group, subject, and body system.

9.9.8 Vital Signs

Vital sign evaluations including pulse (sitting and standing), systolic and diastolic blood pressure (sitting and standing), respiration rate, and temperature are reported for all test infusion and study medication infusion visits. During infusion visits, repeated assessments are done pre-dose (baseline), at various time points during infusion, and after the end of infusion.

Frequency tabulations for vital signs display the number and percentage of subjects with clinically relevant abnormalities during or after infusion. Findings are displayed for all test infusion visits (interim visits after catheter repositioning and Week 80/e40 visits) and study medication infusion visits in the Initial Extension. The criteria for clinically relevant postbaseline abnormalities are outlined in Table 6.

Vital sign data from infusions with a clinically revelant postbaseline abnormal result from all extension parts of Study 2797 (Initial Extension, Pilot Extension, Supplemental Extension) are listed by study stage, treatment group, and subject.

Table 6. Clinically Relevant Postbaseline Abnormalities for Vital Signs Parameters

Parameter	Criterion
Temperature	> 38°C and an increase from pre-dose of at least 1°C
Respiration rate	< 12 or > 20 breaths/min
Pulse	≥ 120 bpm or an increase from pre-dose of > 20 bpm
Pulse	< 50 bpm
Systolic BP	≥ 180 mm Hg or an increase from pre-dose of ≥ 30 mmHg
Systolic BP	< 90 mmHg or a decrease from pre-dose of ≥ 30 mmHg
Diastolic BP	≥ 105 mmHg or an increase from pre-dose of ≥ 20 mmHg
Diastolic BP	< 50 mmHg or a decrease from pre-dose of ≥ 20 mmHg

Pre-dose relates to the pre-infusion value at the respective visit.

9.9.9 Weight

Body weight data from all extension parts of Study 2797 (Initial Extension, Pilot Extension, Supplemental Extension) are listed by study stage, treatment group, and subject.

9.9.10 Electrocardiogram

ECG data are reported at the screening visit from Study 2553 (baseline), Week 40/e0, and Week 80/e40. Quantitative parameters are heart rate (beats/min), PR interval (ms), QRS interval (ms), QT interval (ms), and QTc interval (ms). An overall impression is recorded in the CRF as normal or abnormal and, if abnormal,

clinically significant yes (specify abnormality) or no in the investigator's judgment. In addition, objective criteria for clinically relevant QTc abnormalities are defined in Table 7.

Table 7. Clinically Relevant Abnormalities for QTc*

Parameter	Criterion
QTc	> 450 ms
QTc	> 500 ms
QTc change from baseline	> 30 ms
QTc change from baseline	> 60 ms

* Based on ICH E14 guideline.

Quantitative ECG data are tabulated by treatment group, comparing Week 40/e0 and Week 80/e40 to baseline values using summary statistics for changes from baseline.

A categorical table summarizes the number and percentage of subjects with normal and abnormal ECG results at Week 80/e40 (overall and those judged clinically significant by the investigator) and the number and percentage of subjects with Week 80/e40 QTc abnormalities assessed as clinically relevant according to the criteria in Table 7 (overall and per category).

ECG data, both quantitative parameters and overall impression, from the Initial Extension and Pilot Extension of Study 2797 are listed by study stage, treatment group, and subject with flags for clinically significant overall ECG impression and clinically relevant QTc abnormalities.

9.9.11 Glasgow Coma Scale

Glasgow Coma Scale is reported for all test infusion and study medication infusion visits. The assessments are performed before infusion, 30 minutes into the infusion, and after completion of infusion. Glasgow Coma Scale items include visual response, verbal ability, and motor skills. Each item has 4-6 possible responses. The possible responses for each item are listed in Table 8. The best possible total score is 15.

Table 8. Glasgow Coma Scale Scoring

Visual Response	Verbal Ability	Motor Skills
1. No eye opening	1. No verbal response	1. No motor response
2. Eye opening to pain	2. Incomprehensible sounds	2. Extension to pain
3. Eye opening to verbal command	3. Inappropriate words	3. Flexion to pain
4. Eyes open spontaneously	4. Confused	4. Withdrawal from pain
	5. Orientated	5. Localizing pain
		6. Obeys commands

Frequency tabulations display the number and percentage of subjects with a total Glasgow Coma Scale score of 15 or less than 15 at any time during or after infusion. Findings are displayed for all test infusion visits (interim visits after catheter repositioning and Week 80/e40 visits) and study medication infusion visits in the Initial Extension.

Glasgow Coma Scale results < 15 or missing in all extension parts of Study 2797 (Initial Extension, Pilot Extension, Supplemental Extension) are listed by study stage, treatment group, and subject.

9.9.12 Questionnaire for Impulsive-Compulsive Disorders

The QUIP-Current-Full, version 1.0, is a self-administered or informant-completed scale that includes 13 questions covering symptoms related to the 4 commonest impulse control disorders in PD (gambling, sex, buying, and eating) as well as other behaviors and problematic use of medication. The number of "Yes" responses is compared to the number required for a positive result per the scoring sheet. Missing individual responses are imputed using LOCF. Each impulse control disorder, other behavior, and problematic used of medication is rated as a positive result (ie, present) or a negative result (ie, not present).

QUIP data are summarized for both subject and informant parameters in a table comparing postbaseline to baseline values using shifts from baseline by treatment group and responder. QUIP results are summarized for Week 40/e0 (baseline), Weeks 48/e8, 56/e16, 64/e24, 72/e32 and 80/e40; e2-16, e2-32, e2-48, e2-64, and e2-80; e3-16, e3-32, and the last study visit in the Supplemental Extension.

QUIP items answered with "yes" in all extension parts of Study 2797 (Initial Extension, Pilot Extension, Supplemental Extension) are listed by study stage, treatment group, subject, and responder (informant/subject).

9.9.13 Montreal Cognitive Assessment

The MoCA version 7.1 is a rater-administered cognitive screening tool with 8 components: visuospatial/executive, naming, memory, attention, language, abstraction, delayed recall, and orientation. The total score ranges from 0 to 30, with lower scores representing poorer cognitive function. A total score of 26 or above is considered normal. The MoCA will be analyzed using total score only. Missing individual scores are imputed using LOCF if necessary.

MoCA data are summarized in a table comparing postbaseline to baseline values using summary statistics for changes from baseline by treatment group. MoCA results are summarized for screening, pre-test infusion (baseline), Weeks 40/e0, 56/e16, and 80/e40; e2-80; and the last study visit in the Supplemental Extension.

MoCA total score data from all extension parts of Study 2797 (Initial Extension, Pilot Extension, Supplemental Extension) are listed by study stage, treatment group, and subject.

9.9.14 Mattis Dementia Rating Scale

The MDRS version 2 is a rater-administered global scale of cognition including 5 subscales: attention, initiation/perseveration, construction, conceptualization, and memory. Scores range from 0 to 144, with higher scores representing better cognitive function. In PD, scores <123 are associated with some degree of dementia. The age- and education-corrected Mayo Older Adults Normative Studies (MOANS) scaled score (AEMSS) total score ranges from 0 to 20, with higher scores representing better cognitive function. The MDRS will be analyzed using AEMSS total score only as entered in the CRF.

MDRS data are summarized in a table comparing postbaseline to baseline values using summary statistics for changes from baseline by treatment group. MDRS results are summarized for screening (baseline), Weeks 40/e0, 56/e16, and 80/e40; e2-80; and the last study visit in the Supplemental Extension.

MDRS AEMSS total score data from all extension parts of Study 2797 (Initial Extension, Pilot Extension, Supplemental Extension) are listed by study stage, treatment group, and subject.

9.9.15 Stroop Test

The Stroop test is a global scale of reaction time including 4 conditions: color naming, word reading, inhibition, and inhibition/switching. Total time to complete the test in each condition can range from 0 to 999 seconds, with lower time representing better reaction time. The Stroop test will be analyzed separately in each condition.

Stroop test data are summarized in a table comparing postbaseline to baseline values using summary statistics for changes from baseline by treatment group. Stroop test results are summarized for screening (baseline), Week 40/e0, and Week 80/e40.

Stroop test data are listed by study stage, treatment group, and subject.

9.9.16 Frontal Systems Behavioural Scale

The FrSBe is a scale that assesses behavior related to frontal systems damage including 3 subscales: apathy, disinhibition, and executive dysfunction. Higher subscale scores indicate greater pathology. Total score is not analyzed for this study. Individual missing items are imputed using the average of non-missing scores in each subscale.

FrSBe data are summarized in a table comparing postbaseline (Week 40/e0 “after” and Week 80/e40 “after”) to baseline values (“after” values for Screening Visit 2 from Study 2553) for each subscale using summary statistics for changes from baseline by treatment group. FrSBe results are summarized for screening (before and after [baseline]), Week 40/e0, and Week 80/e40.

FrSBe test data are listed by study stage, treatment group, and subject.

9.9.17 Deary-Liewald Reaction Time

The Deary-Liewald RT is a computerized measure of simple and four-choice RT. The parameter is the mean reaction time, variance and SD for correct responses for four-choice RT. A shorter reaction time is better.

Deary-Liewald four-choice RT data are summarized in a table comparing postbaseline to baseline values using summary statistics for changes from baseline by treatment group. Deary-Liewald RT results are summarized for Screening Visit 2 (baseline), Week 40/e0, Week 56/e16, and Week 80/e40.

Four-choice RT test data are listed by study stage, treatment group, and subject. Simple RT data are not listed, but are included in SDTM datasets.

9.9.18 Verbal Fluency Assessment

The verbal fluency assessment is a test of verbal functioning in 2 categories: phonemic and semantic. Scores (number of correct words in one minute) range from 0 to 200, with a higher score representing better verbal functioning. Verbal fluency will be analyzed by number of correct responses in each category.

Verbal fluency data are summarized in a table comparing postbaseline to baseline values using summary statistics for changes from baseline by treatment group. Verbal fluency results are summarized for screening (baseline), Week 40/e0, and Week 80/e40. Parameters are phonemic fluency score and semantic fluency score.

Verbal fluency assessment test data are listed by study stage, treatment group.

9.9.19 Beck Depression Inventory

The Beck Depression Inventory (BDI) is a self-administered test which consists of 21 questions that measure the severity of depression. Scores range from 0 to 63, with higher scores representing worse depression. Individual missing items are imputed using the average of non-missing scores in each subscale. The total score is the parameter analyzed.

BDI data are summarized in a table comparing postbaseline to baseline values using summary statistics for changes from baseline by treatment group. BDI results are summarized for screening (baseline), Week 40/e0, Week 80/e40; and the last study visit in both the Pilot Extension and the Supplemental Extension.

BDI total score data from all extension parts of Study 2797 (Initial Extension, Pilot Extension, Supplemental Extension) are listed by study stage, treatment group, and subject.

9.9.20 University of Pennsylvania Smell Identification Test

The UPSIT is a self-administered test which can be used to identify and quantitate olfactory dysfunction in PD. The number of correct responses out of 40 total items constitutes a subject's score with interpretations as shown in Table 9. Lower scores represent greater olfactory dysfunction. Individual missing responses are imputed as zeros (ie, incorrect responses).

Table 9. UPSIT Score Interpretation

Test Score (Males)	Test Score (Females)	Olfactory Diagnosis
0 – 5	0 – 5	Probable malingering
6-18	6-18	Total anosmia
19-25	19-25	Severe microsmia
26-29	26-30	Moderate microsmia
30-33	31-34	Mild microsmia
34-40	35-40	Normosmia

UPSIT data are summarized in a table comparing postbaseline to baseline values using summary statistics for changes from baseline by treatment group. UPSIT results are summarized for screening (baseline), Week 40/e0, and Week 80/e40.

UPSIT test data from the Initial Extension and Pilot Extension of Study 2797 are listed by study stage, treatment group, and subject.

10.0 Validation

PRA's goal is to ensure that each TFL delivery is submitted to the highest level of quality. Our quality control procedures will be documented separately in the study specific quality control plan.

Appendix 1 Glossary of Abbreviations

**aCSF**	Artificial Cerebrospinal Fluid
**ADL**	Activities of Daily Living
**AE**	Adverse Event
**AESI**	Adverse Event of Special Interest
**ALT**	Alanine Aminotransferase
**ANCOVA**	Analysis of Covariance
**ATC**	Anatomical Therapeutic Chemical
**BDI**	Beck Depression Inventory
**BP**	Blood Pressure
**CDISC**	Clinical Data Interchange Standards Consortium
**CED**	Convection-Enhanced Delivery
**CI**	Confidence Interval
**COMT**	Catechol-O-methyl transferase
**CRF**	Case Report Form
**ECG**	Electrocardiogram
**eGFR**	Estimated Glomerular Filtration Rate
**EQ-5D**	EuroQOL 5-dimensional Scale
**FLAIR**	Fluid-attenuated Inversion Recovery
**FrSBe**	Frontal Systems Behavioural Scale
**GDNF**	Glial Cell Line-derived Neurotrophic Factor
**ITT**	Intent-To-Treat
**LOCF**	Last Observation Carried Forward
**MCH**	Mean Corpuscular Hemoglobin
**MCHC**	Mean Corpuscular Hemoglobin Concentration
**MCV**	Mean Corpuscular Volume
**MDRS**	Mattis Dementia Rating Scale
**MedDRA**	Medical Dictionary for Regulatory Activities
**MHRA**	Medicines and Healthcare Products Regulatory Agency
**MMRM**	Mixed-effect Model with Repeated Measures
**MoCA**	Montreal Cognitive Assessment
**MRI**	Magnetic Resonance Imaging
**NBT**	North Bristol National Health System Trust

**NMSS**	Non-Motor Symptom Assessment Scale
**PD**	Parkinson's Disease
**PDQ-39**	Parkinson's Disease Questionnaire-39
**PRA**	Pharmaceutical Research Associates
**PT**	Preferred Term
**QUIP**	Questionnaire for Impulsive-Compulsive Disorders in Parkinson's Disease
**RBC**	Red Blood Cells
**RT**	Reaction Time
**SAE**	Serious Adverse Event
**SAP**	Statistical Analysis Plan
**SD**	Standard Deviation
**SDTM**	Standard Data Tabulation Model
**SE**	Standard Error
**SNAQ**	Simplified Nutritional Appetite Questionnaire
**SOC**	System Organ Class
**TEAE**	Treatment-Emergent Adverse Event
**TFL**	Tables, Figures, and Listings
**UPDRS**	Unified Parkinson's Disease Rating Scale
**UPSIT**	University of Pennsylvania Smell Identification Test
**VOI**	Volume of Interest
**WBC**	White Blood Cells
**WHODRUG DDE**	World Health Organization Drug Dictionary Enhanced

Appendix 2 Protocol Deviation Guidance

Definitions

A protocol deviation is any change, divergence, or departure from the study design or procedures defined in the protocol.

Major protocol deviations are a subset of protocol deviations that might significantly affect a subject's rights, safety, or well-being or that might significantly affect the completeness, accuracy, and/or reliability of core study data. For example, major protocol deviations might include enrolling subjects in violation of key eligibility criteria designed to ensure a specific subject population or failing to collect data necessary to interpret the primary endpoint, as this may compromise the scientific value of the trial.

Minor deviations are deviations that do not meet the above criteria, i.e. do not impact subject safety or the scientific integrity of the study.

Examples of major and minor protocol violations are provided in the table below.

Major Deviations	Minor Deviations
Key eligibility criteria violated	Visit outside of window
If a subject missed more than one study drug infusion during the Initial Extension, the second missed infusion is classified as major	Protocol required assessment not completed (except for OFF state UPDRS motor score at baseline and/or Week 80/e40)
Subject significantly overdosed	Failure to report SAE in required timeframe
Baseline and/or Week 80/e40 OFF state UPDRS motor score not completed properly	Entire visit not conducted (other than Baseline and Week 80/e40)
Failure to obtain informed consent from subject	Lab sample preparation or storage not adequate (anti-GDNF antibodies and plasma concentration of GDNF)
	Any missed study drug infusion other than a second missed study drug infusion during the Initial Extension

As per the study protocol, the primary analysis will be an intention-to-treat (ITT) analysis including all subjects enrolled in the Primary Stage (ITT Primary Population).

Process

From study start until January 2015, protocol deviations were documented in file notes by the study staff, and provided to PRA for entry into their Clinical Trials Management System (see Memo to File from Robert Appel dated 03-Jun-2014, RE: Project Plan: Planned Process Deviations process). Starting in January 2015, Case Report Forms (CRFs) were created to capture protocol deviations for entry into the clinical database. Protocol deviations previously captured in file notes will be transcribed to the Protocol Deviation CRF.

Prior to database lock, all protocol deviations will be reviewed to assess the deviation classification (major/minor) and category (categories and guidance are provided in the table below). For this review, the adjudication team will use the list of protocol deviations generated from the clinical database as an input, and the list of subjects in the ITT population from the Biostatistician or Analysis Programmer. Final determination of the deviation classification (major or minor) will be made by the Study Sponsor in view of the recommendations made by the adjudication team.

Category	Guidance
Inclusion criteria	Any deviation relating to inclusion criteria not being met
Exclusion criteria	Any deviation relating to exclusion criteria being met
Study medication (including overdose)	Any deviation relating to the administration of study medication. This includes GDNF/aCSF only and does not include test infusions or medications given for levodopa challenge.
Non-study medication	Any deviation relating to medication other than study medication. This includes concomitant medications and PD medications.
Study schedule/visit windows	Any deviation relating to an assessment which was completed, but outside the visit window or incorrectly completed. An assessment refers to efficacy, safety or imaging.
Outcome assessment	Any deviation relating to an assessment which was entirely missed. An assessment refers to efficacy, safety or imaging.
Other, specify	Deviations which clearly do not fit in any of the other categories.

Appendix 3 List of MedDRA Preferred Terms for Adverse Events of Special Interest

Preferred Terms Related to Adverse Changes in Mood:

SOC Psychiatric disorders

- Agitation
- Anxiety
- Crying
- Depressed mood
- Depression
- Feeling of despair
- Hypomania
- Suicidal ideation
- Tearfulness

Preferred Terms Related to Impulsivity:

SOC Psychiatric disorders

- Compulsive shopping
- Dopamine dysregulation syndrome
- Hypersexuality
- Impulsive behaviour
- Libido increased
- Obsessive-compulsive disorder

Appendix 4 List of Conversion Factors for the Calculation of Levodopa and Levodopa Equivalent Doses

PD Medication	Conversion Factor
**Levodopa Preparations**	
Immediate release preparations without COMT inhibition	1.0
Immediate release preparations with entacapone	1.33
Immediate release preparations with tolcapone	1.5
Controlled release preparations	0.75
Levodopa/carbidopa (Duodopa)	1.11
**MAO-B Inhibitors**	
Selegiline oral	10
Selegiline sublingual	80
Rasagiline	100
**Dopamine Agonists**	
Ropinirole immediate release	20
Ropinirole long acting	20
Pramipexole immediate release (base)	140
Pramipexole immediate release (salt)	100
Pramipexole long acting (base)	140
Pramipexole long acting (salt)	100
Cabergoline	70
Rotigotine	30
Piribedil	1
Apomorphine	10
Bromocriptine	10
Pergolide	100
Lisuride	100
Dihydroergocryptine (DHEC)	5
**Other**	
Amantadine	1

Note: COMT inhibitor doses are not included in the calculation of levodopa or levodopa equivalent doses. They contribute to the levodopa or levodopa equivalent dose by modifying the dose of concurrently administered levodopa.

Appendix 5 List of Post-Text Tables, Figures and Listings

16.1 DEMOGRAPHIC DATA TABLES ... 64
Table 16.1.1.1 Subject Populations - All Enrolled Subjects ... 64
Table 16.1.1.2.1 Subject Disposition - ITT Primary Population ... 65
Table 16.1.1.2.2 Subject Disposition - ITT Overall Population ... 66
Table 16.1.2 Protocol Deviations - ITT Overall Population ... 67
Table 16.1.3.1 Demographic Characteristics from Study 2553 - ITT Primary Population ... 68
Table 16.1.3.2 Demographic Characteristics from Study 2553 - ITT Overall Population ... 70
Table 16.1.4.1 Parkinson's Disease History at Screening from Study 2553 - ITT Primary Population ... 71
Table 16.1.4.2 Parkinson's Disease History at Screening from Study 2553 - ITT Overall Population ... 73
Table 16.1.5.1 Concomitant Parkinson's Disease Medications by WHO Drug Dictionary ATC Class and Preferred Name - ITT Primary Population ... 74
Table 16.1.5.2 Other Concomitant Medications by WHO Drug Dictionary ATC Class and Preferred Name - ITT Primary Population ... 75
16.2 EFFICACY DATA TABLES ... 76
16.2.1 UPDRS PRIMARY EFFICACY TABLES ... 76
Table 16.2.1.1 OFF State UPDRS Motor Score (Part III): Change and Percentage Change from Baseline to Week 80/e40 – MMRM, ITT Primary Population ... 77
Table 16.2.1.2 OFF State UPDRS Motor Score (Part III): Change and Percentage Change from Baseline to Week 80/e40 – MMRM, ITT Overall Population ... 78
Table 16.2.2.1 ON State UPDRS Motor Score (Part III): Change and Percentage Change from Baseline to Week 80/e40 – MMRM, ITT Overall Population ... 79
Table 16.2.2.2.1 OFF State UPDRS ADL Score (Part II): Change and Percentage Change from Baseline to Week 80/e40 – MMRM, ITT Primary Population ... 79
Table 16.2.2.2.2 OFF State UPDRS ADL Score (Part II): Change and Percentage Change from Baseline to Week 80/e40 – MMRM, ITT Overall Population ... 79
Table 16.2.2.3 ON State UPDRS ADL Score (Part II): Change and Percentage Change from Baseline to Week 80/e40 – MMRM, ITT Overall Population ... 79
Table 16.2.2.4.1 OFF State UPDRS Total Score: Change and Percentage Change from Baseline to Week 80/e40 – MMRM, ITT Primary Population ... 80
Table 16.2.2.4.2 OFF State UPDRS Total Score: Change and Percentage Change from Baseline to Week 80/e40 – MMRM, ITT Overall Population ... 80
Table 16.2.2.5 ON State UPDRS Total Score: Change and Percentage Change from Baseline to Week 80/e40 – MMRM, ITT Overall Population ... 80
Table 16.2.2.6.1 OFF State UPDRS Motor Score (Part III): Change from Baseline to Week 40/e0 for GDNF/GDNF Group Compared to Change from Baseline to Week 80/e40 for Placebo/GDNF Group – MMRM, ITT Overall Population ... 81
Table 16.2.2.6.2 OFF State UPDRS ADL Score (Part II): Change from Baseline to Week 40/e0 for GDNF/GDNF Group Compared to Change from Baseline to Week 80/e40 for Placebo/GDNF Group – MMRM, ITT Overall Population ... 82
Table 16.2.2.6.3 OFF State UPDRS Total Score: Change from Baseline to Week 40/e0 for GDNF/GDNF Group Compared to Change from Baseline to Week 80/e40 for Placebo/GDNF Group – MMRM, ITT Overall Population ... 82

Table 16.2.2.7.1 OFF State UPDRS Motor Score (Part III): Change from Baseline to Week 80/e40 for GDNF/GDNF Group Compared to Change from Baseline to Week 40/e0 for Placebo/GDNF Group – MMRM, ITT Overall Population 82
Table 16.2.2.7.2 OFF State UPDRS ADL Score (Part II): Change from Baseline to Week 80/e40 for GDNF/GDNF Group Compared to Change from Baseline to Week 40/e0 for Placebo/GDNF Group – MMRM, ITT Overall Population 82
Table 16.2.2.7.3 OFF State UPDRS Total Score: Change from Baseline to Week 80/e40 for GDNF/GDNF Group Compared to Change from Baseline to Week 40/e0 for Placebo/GDNF Group – MMRM, ITT Overall Population 82
16.2.3 PD DIARY SECONDARY EFFICACY TABLES 83
Table 16.2.3.1 Motor Fluctuation Diary Ratings by Visit: Change from Baseline – MMRM, ITT Primary Population 83
Table 16.2.3.2 Motor Fluctuation Diary Ratings by Visit: Change from Baseline – MMRM, ITT Overall Population 85
Table 16.2.3.3.1 Treatment Response at Week 40/e0 and Week 80/e40 - ITT Overall Population 86
Table 16.2.3.3.2 UPDRS Responder at Week 80/e40 Compared to Week 40/e0 - ITT Overall Population 87
16.2.4 SUPPLEMENTARY EFFICACY TABLES 88
Table 16.2.4.1 OFF and ON State Timed Walking Test by Visit: Change from Baseline – MMRM, ITT Overall Population 88
Table 16.2.4.2 OFF and ON State Timed Tapping Test by Visit: Change from Baseline – MMRM, ITT Overall Population 90
Table 16.2.4.3 NMSS Score by Visit: Change from Baseline – MMRM, ITT Overall Population 91
Table 16.2.4.4 PDQ-39 Score: Change from Baseline to Week 80/e40 – ANCOVA, ITT Overall Population 92
Table 16.2.4.5.1 EQ-5D Questionnaire: Baseline, Week 40/e0, and Week 80/e40 – Observed Data, ITT Overall Population 94
Table 16.2.4.5.2 EQ-5D Visual Analog Scale: Change from Baseline to Week 80/e40 – ANCOVA, ITT Overall Population 95
Table 16.2.4.6 SNAQ Score: Change from Baseline to Week 80/e40 – ANCOVA, ITT Overall Population 95
Table 16.2.4.7 Total Daily Levodopa Dose (mg): Change From Baseline to Week 80/e40 – ANCOVA, ITT Overall Population 96
Table 16.2.4.8 Total Daily Levodopa Equivalent Dose (mg): Change From Baseline to Week 80/e40 – ANCOVA, ITT Overall Population 97
16.3 IMAGING TABLES 98
Table 16.3.1 Volume of Distribution of Infusate as Determined by Contrast-Enhanced T1-Weighted MRI: Change from Baseline to Week 80/e40 – ANCOVA, ITT Primary Population 98
Table 16.3.2.1 Volume of Interest Coverage as Determined by Contrast-Enhanced T1-Weighted MRI: Change from Baseline to Week 80/e40 – ANCOVA, ITT Primary Population 98
Table 16.3.2.2 Total Putamenal Coverage as Determined by Contrast-Enhanced T1-Weighted MRI: Change from Baseline to Week 80/e40 – ANCOVA, ITT Primary Population 98
Table 16.3.3.1 Correlation Analyses of Percentage Change from Baseline to Week 80/e40 in OFF State UPDRS Motor Score (Part III) to Volume of Interest Coverage and Total Putamenal Coverage at Baseline as Determined by Contrast-Enhanced T1-Weighted MRI – ITT Primary Population 99

Table 16.3.3.2 Correlation Analysis of Percentage Change from Baseline to Week 80/e40 in OFF State UPDRS Motor Score (Part III) to Change from Baseline to Week 40/e0 in ^{18}F-DOPA Uptake as Determined by PET Scan – ITT Primary Population 100
Table 16.3.3.3 Correlation Analysis of Percentage Change from Baseline to Week 80/e40 in OFF State UPDRS Motor Score (Part III) to Change from Baseline to Week 40/e0 in ^{18}F-DOPA Uptake as Determined by PET Scan – ITT Overall Population 100
16.4 Safety Data Tables 101
16.4.1 Exposure Data Tables 101
Table 16.4.1.1 Exposure to Study Medication - Safety Overall Population 101
Table 16.4.1.2 Study Medication Infusion Details by Visit - Safety Overall Population 103
16.4.2 Adverse Event Tables 104
Table 16.4.2.1.1 Overall Summary of Adverse Events - Safety Overall Population 104
Table 16.4.2.1.2 Overall Summary of Adverse Events During the Initial Extension - Safety Overall Population 105
Table 16.4.2.1.3 Overall Summary of Adverse Events During the Pilot and Supplemental Extensions - Safety Overall Population 105
Table 16.4.2.2 Treatment-Emergent Adverse Events by System Organ Class and Preferred Term - Safety Overall Population 106
Table 16.4.2.3.1 Treatment-Emergent Adverse Events Experienced by at Least 3 Subjects Overall by Preferred Term - Safety Overall Population 107
Table 16.4.2.3.2 Treatment-Emergent Adverse Events Experienced by at Least 3 Subjects Overall During the Initial Extension by Preferred Term - Safety Overall Population 108
Table 16.4.2.3.3 Treatment-Emergent Adverse Events Experienced by at Least 3 Subjects Overall During the Pilot and Supplemental Extensions by Preferred Term - Safety Overall Population . 108
Table 16.4.2.4 Treatment-Emergent Adverse Events by System Organ Class, Preferred Term, and Maximum Severity - Safety Overall Population 109
Table 16.4.2.5.1 Serious Treatment-Emergent Adverse Events by System Organ Class and Preferred Term - Safety Overall Population 111
Table 16.4.2.5.2 Serious Treatment-Emergent Adverse Events by System Organ Class and Preferred Term During the Initial Extension - Safety Overall Population 111
Table 16.4.2.5.3 Serious Treatment-Emergent Adverse Events by System Organ Class and Preferred Term During the Pilot and Supplemental Extensions - Safety Overall Population 111
Table 16.4.2.6 Study Medication-Related Treatment-Emergent Adverse Events by System Organ Class and Preferred Term - Safety Overall Population 112
Table 16.4.2.7 Serious Study Medication-Related Treatment-Emergent Adverse Events by System Organ Class and Preferred Term - Safety Overall Population 112
Table 16.4.2.8 Device-Related Treatment-Emergent Adverse Events by System Organ Class and Preferred Term - Safety Overall Population 112
Table 16.4.2.9 Serious Device-Related Treatment-Emergent Adverse Events by System Organ Class and Preferred Term - Safety Overall Population 112
Table 16.4.2.10 Treatment-Emergent Adverse Events of Special Interest by Preferred Term - Safety Overall Population 113
16.4.3 Laboratory Tables 114
Table 16.4.3.1 Clinically Significant Postbaseline Hematology Results - Safety Overall Population 114
Table 16.4.3.2 Clinically Significant Postbaseline Serum Chemistry Results - Safety Overall Population 115

16.4.4 ANTI-GDNF SERUM ANTIBODY TABLES 116
Table 16.4.4.1 Anti-GDNF Binding Serum Antibodies by Visit – Safety Overall Population 116
Table 16.4.4.2 Anti-GDNF Neutralizing Serum Antibodies by Visit - Safety Overall Population. 118
16.4.5 PLASMA GDNF CONCENTRATION TABLES 119
Table 16.4.5 Plasma GDNF Concentrations by Visit - Safety Overall Population 119
16.4.6 VITAL SIGN TABLE 120
Table 16.4.6 Clinically Relevant Postbaseline Abnormalities in Vital Sign Results in the Initial Extension - Safety Overall Population 120
16.4.7 ELECTROCARDIOGRAM TABLES 121
Table 16.4.7.1 Electrocardiogram Results by Visit – Safety Overall Population 121
Table 16.4.7.2 Summary of Electrocardiogram Results at Week 80/e40 - Safety Overall Population 122
16.4.8 GLASGOW COMA SCALE TABLE 123
Table 16.4.8 Glasgow Coma Scale Score = 15 or < 15 During or After Infusion by Visit and Time Point in the Initial Extension– Safety Overall Population 123
16.4.9 OTHER SAFETY TABLES 124
Table 16.4.9.1 QUIP by Visit - Safety Overall Population 124
Table 16.4.9.2 MoCA by Visit - Safety Overall Population 126
Table 16.4.9.3 MDRS by Visit - Safety Overall Population 126
Table 16.4.9.4 Stroop Test by Visit, Safety Overall Population 126
Table 16.4.9.5 FrSBe by Visit - Safety Overall Population 126
Table 16.4.9.6 Deary-Liewald Four-Choice Reaction Time by Visit - Safety Overall Population.. 126
Table 16.4.9.7 Verbal Fluency Assessment by Visit - Safety Overall Population 126
Table 16.4.9.8 BDI by Visit - Safety Overall Population 127
Table 16.4.9.9 UPSIT by Visit - Safety Overall Population 127
16.5 FIGURES 128
Figure 16.5.1.1.1 OFF State UPDRS Motor Score (Part III): Percentage Change Over Time - ITT Primary Population 128
Figure 16.5.1.1.2 OFF State UPDRS Motor Score (Part III): Percentage Change Over Time - ITT Overall Population 130
Figure 16.5.1.2.1 OFF State UPDRS Motor Score (Part III): Change Over Time - ITT Primary Population 130
Figure 16.5.1.2.2 OFF State UPDRS Motor Score (Part III): Change Over Time - ITT Overall Population 130
Figure 16.5.1.3 ON State UPDRS Motor Score (Part III): Change Over Time - ITT Overall Population 130
Figure 16.5.1.4.1 OFF State UPDRS ADL Score (Part II): Change Over Time - ITT Primary Population 130
Figure 16.5.1.4.2 OFF State UPDRS ADL Score (Part II): Change Over Time - ITT Overall Population 130
Figure 16.5.1.5 ON State UPDRS ADL Score (Part II): Change Over Time - ITT Overall Population 130
Figure 16.5.1.6.1 OFF State UPDRS Total Score: Change Over Time - ITT Primary Population.... 130
Figure 16.5.1.6.2 OFF State UPDRS Total Score: Change Over Time - ITT Overall Population...... 130
Figure 16.5.1.7 ON State UPDRS Total Score: Change Over Time - ITT Overall Population........ 130
Figure 16.5.2.1 Motor Fluctuation Diary Total OFF Time Per Day (Hours): Change Over Time - ITT Primary Population 131

Figure 16.5.2.2 Motor Fluctuation Diary Total OFF Time Per Day (Hours): Change Over Time - ITT Overall Population 131
Figure 16.5.2.3 Motor Fluctuation Diary Total Good-Quality ON Time Per Day (Hours): Change Over Time - ITT Primary Population 131
Figure 16.5.2.4 Motor Fluctuation Diary Total Good-Quality ON Time Per Day (Hours): Change Over Time - ITT Overall Population 131
Figure 16.5.3 OFF State UPDRS Motor Score (Part III): Subject Scores Over Time - ITT Primary Population 132
Figure 16.5.4.1 OFF State UPDRS Motor Score (Part III): Frequency Distribution of Change at Week 40/e0 - ITT Overall Population 133
Figure 16.5.4.2 OFF State UPDRS Motor Score (Part III): Frequency Distribution of Change at Week 80/e40 - ITT Overall Population 133
Figure 16.5.5.1 Correlation Analysis of Percentage Change from Baseline to Week 80/e40 in OFF State UPDRS Motor Score (Part III) to Volume of Interest Coverage at Baseline as Determined by Contrast-Enhanced T1-Weighted MRI - ITT Primary Population 134
Figure 16.5.5.2 Correlation Analysis of Percentage Change from Baseline to Week 80/e40 in OFF State UPDRS Motor Score (Part III) to Total Putamenal Coverage at Baseline as Determined by Contrast-Enhanced T1-Weighted MRI - ITT Primary Population 135
Figure 16.5.5.3 Correlation Analysis of Percentage Change from Baseline to Week 80/e40 in OFF State UPDRS Motor Score (Part III) to Change From Baseline to Week 40/e0 in ^{18}F-DOPA Uptake as Determined by PET Scan - ITT Primary Population 136
Figure 16.5.5.4 Correlation Analysis of Baseline (Week 0) OFF State UPDRS Motor Score (Part III) to Baseline (Week 0) ^{18}F-DOPA Uptake as Determined by PET Scan for All Subjects - ITT Overall Population 136
Figure 16.5.5.5 Correlation Analysis of Baseline (Week 0) OFF State UPDRS Motor Score (Part III) to Baseline (Week 0) ^{18}F-DOPA Uptake as Determined by PET Scan for GDNF/GDNF Subjects - ITT Overall Population 136
Figure 16.5.5.6 Correlation Analysis of Baseline (Week 0) OFF State UPDRS ADLScore (Part II) to Baseline (Week 0) ^{18}F-DOPA Uptake as Determined by PET Scan for All Subjects - ITT Overall Population 136
Figure 16.5.5.7 Correlation Analysis of Baseline (Week 0) OFF State UPDRS ADL Score (Part II) to Baseline (Week 0) ^{18}F-DOPA Uptake as Determined by PET Scan for GDNF/GDNF Subjects - ITT Overall Population 136
Figure 16.5.5.8 Correlation Analysis of Week 40/e0 OFF State UPDRS Motor Score (Part III) to Week 40/e0 ^{18}F-DOPA Uptake as Determined by PET Scan for All Subjects - ITT Overall Population 136
Figure 16.5.5.9 Correlation Analysis of Week 40/e0 OFF State UPDRS Motor Score (Part III) to Week 40/e0 ^{18}F-DOPA Uptake as Determined by PET Scan for GDNF/GDNF Subjects - ITT Overall Population 136
Figure 16.5.5.10 Correlation Analysis of Week 40/e0 OFF State UPDRS ADLScore (Part II) to Week 40/e0 ^{18}F-DOPA Uptake as Determined by PET Scan for All Subjects - ITT Overall Population 137
Figure 16.5.5.11 Correlation Analysis of Week 40/e0 OFF State UPDRS ADL Score (Part II) to Week 40/e0 ^{18}F-DOPA Uptake as Determined by PET Scan for GDNF/GDNF Subjects - ITT Overall Population 137

Figure 16.5.5.12 Correlation Analysis of Week 80/e40 OFF State UPDRS Motor Score (Part III) to Week 40/e0 ^{18}F-DOPA Uptake as Determined by PET Scan for All Subjects - ITT Overall Population ... 137
Figure 16.5.5.13 Correlation Analysis of Week 80/e40 OFF State UPDRS Motor Score (Part III) to Week 40/e0 ^{18}F-DOPA Uptake as Determined by PET Scan for GDNF/GDNF Subjects - ITT Overall Population ... 137
Figure 16.5.5.14 Correlation Analysis of Week 80/e40 OFF State UPDRS ADLScore (Part II) to Week 40/e0 ^{18}F-DOPA Uptake as Determined by PET Scan for All Subjects - ITT Overall Population ... 137
Figure 16.5.5.15 Correlation Analysis of Week 80/e40 OFF State UPDRS ADL Score (Part II) to Week 40/e0 ^{18}F-DOPA Uptake as Determined by PET Scan for GDNF/GDNF Subjects - ITT Overall Population ... 137
17 DATA LISTINGS ... 138
17.2.1 BASELINE LISTINGS ... 138
Listing 17.2.1.1 Study Completion Status - ITT Overall Population ... 138
Listing 17.2.1.2.1 Major Protocol Deviations - ITT Overall Population ... 139
Listing 17.2.1.2.2 Minor Protocol Deviations - ITT Overall Population ... 140
Listing 17.2.1.3 Subject Populations - ITT Overall Population ... 141
Listing 17.2.1.4.1 Demographic Characteristics in Study 2553 - ITT Overall Population ... 142
Listing 17.2.1.4.2 Parkinson's Disease History at Screening in Study 2553 - ITT Overall Population ... 143
Listing 17.2.1.5 Concomitant Medications – ITT Overall Population ... 144
Listing 17.2.1.6 Catheter Trajectory - ITT Overall Population ... 145
Listing 17.2.1.7 Catheter Positioning Accuracy by Surgery - ITT Overall Population ... 146
Listing 17.2.1.8 Test Infusion Data by Visit - ITT Overall Population ... 147
Listing 17.2.1.9 Test Infusion Catheter Interruptions/Early Terminations by Visit - ITT Overall Population ... 148
17.2.2 EFFICACY LISTINGS ... 149
Listing 17.2.2.1 OFF and ON state UPDRS Scores by Visit - ITT Overall Population ... 149
Listing 17.2.2.2 PD Motor Fluctuation Diary Ratings by Visit - ITT Overall Population ... 151
Listing 17.2.2.3 Treatment Response at Week 40/e0 and Week 80/e40 - ITT Overall Population ... 152
Listing 17.2.2.4.1 OFF and ON State Timed Walking Test by Visit - ITT Overall Population ... 154
Listing 17.2.2.4.2 OFF and ON State Timed Tapping Test by Visit - ITT Overall Population ... 155
Listing 17.2.2.5 NMSS Scores by Visit - ITT Overall Population ... 156
Listing 17.2.2.6 PDQ-39 Scores by Visit - ITT Overall Population ... 156
Listing 17.2.2.7 EQ-5D Scores by Visit - ITT Overall Population ... 156
Listing 17.2.2.8 SNAQ Scores by Visit - ITT Overall Population ... 156
Listing 17.2.2.9.1 Levodopa and Levodopa Equivalent Medication Actual Total Daily Doses - ITT Overall Population ... 157
Listing 17.2.2.9.2 Effective Levodopa and Levodopa Equivalent Medication Total Daily Doses - ITT Overall Population ... 159
17.2.3 IMAGING LISTINGS ... 160
Listing 17.2.3.1 Volume of Distribution, Volume of Interest Coverage, and Total Putamenal Coverage as Determined by Contrast-Enhanced T1-Weighted MRI by Visit - ITT Overall Population ... 160
17.2.4 SAFETY LISTINGS ... 161

Listing 17.2.4.1.1 Exposure to Study Medication - Safety Overall Population 161
Listing 17.2.4.1.2 Study Medication Infusion Data by Visit - Safety Overall Population 162
Listing 17.2.4.1.3 Study Medication Infusion Catheter Interruptions/Early Terminations by Visit - Safety Overall Population 163
Listing 17.2.4.2 Adverse Events - Safety Overall Population 164
Listing 17.2.4.3.1 Adverse Events of Special Interest: Dyskinesias- Safety Overall Population 165
Listing 17.2.4.3.2 Adverse Events of Special Interest: Falls- Safety Overall Population 165
Listing 17.2.4.3.3 Adverse Events of Special Interest: Adverse Changes in Mood- Safety Overall Population 165
Listing 17.2.4.3.4 Adverse Events of Special Interest: Impulsivity- Safety Overall Population 165
Listing 17.2.4.4 Port Symptoms by Visit - Safety Overall Population 166
Listing 17.2.4.5 Hematology Results by Visit - Safety Overall Population 167
Listing 17.2.4.6 Serum Chemistry Results by Visit - Safety Overall Population 168
Listing 17.2.4.7 Urinalysis Results by Visit - Safety Overall Population 168
Listing 17.2.4.8 Anti-GDNF Serum Antibodies - Safety Overall Population 169
Listing 17.2.4.9 Plasma GDNF Concentration Results by Visit - Safety Overall Population 170
Listing 17.2.4.10 Physical Examination - Safety Overall Population 171
Listing 17.2.4.11 Vital Signs from Infusions with a Clinically Revelant Postbaseline Abnormal Result by Visit and Time Point - Safety Overall Population 173
Listing 17.2.4.12 Weight (kg) by Visit - Safety Overall Population 174
Listing 17.2.4.13 Electrocardiogram Results by Visit – Continuous Parameters, Safety Overall Population 174
Listing 17.2.4.14 Electrocardiogram Results by Visit – Overall Impression, Safety Overall Population 175
Listing 17.2.4.15 Glasgow Coma Scale Results < 15 by Visit - Safety Overall Population 176
Listing 17.2.4.16 QUIP Items Answered with Yes by Visit - Safety Overall Population 177
Listing 17.2.4.17 MoCA Total Score by Visit - Safety Overall Population 179
Listing 17.2.4.18 MDRS AEMSS Total Score by Visit - Safety Overall Population 180
Listing 17.2.4.19 Stroop Test by Visit - Safety Overall Population 181
Listing 17.2.4.20 FrSBe by Visit - Safety Overall Population 182
Listing 17.2.4.21 Deary-Liewald Four-Choice Reaction Time by Visit - Safety Overall Population 183
Listing 17.2.4.22 Verbal Fluency Assessment by Visit - Safety Overall Population 184
Listing 17.2.4.23 BDI by Visit - Safety Overall Population 185
Listing 17.2.4.24 UPSIT by Visit - Safety Overall Population 186

Appendix 6 Shells for Post-Text Tables, Figures and Listings

16.1 Demographic Data Tables

Table 16.1.1.1 Subject Populations - All Enrolled Subjects

Population	GDNF/GDNF	Placebo/GDNF	Total
INITIAL EXTENSION			
Pilot Stage	xx	xx	xx
Subjects enrolled [n]	xx	xx	xx
Subjects treated [n]			
Primary Stage			
Subjects enrolled (ITT Primary Population) [n]	xx	xx	xx
Subjects treated [n]	xx	xx	xx
Overall (Pilot + Primary Stage)			
Subjects enrolled (ITT Overall Population) [n]	xx	xx	xx
Subjects treated (Safety Overall Population) [n]	xx	xx	xx
PILOT EXTENSION (Pilot Stage subjects only)			
Subjects enrolled [n]	xx	xx	xx
Subjects treated [n]	xx	xx	xx
SUPPLEMENTAL EXTENSION			
Pilot Stage	xx	xx	xx
Subjects enrolled [n]	xx	xx	xx
Subjects treated [n]			
Primary Stage			
Subjects enrolled [n]	xx	xx	xx
Subjects treated [n]	xx	xx	xx
Overall (Pilot + Primary Stage)			
Subjects enrolled [n]	xx	xx	xx
Subjects treated [n]	xx	xx	xx

Source: Listing xx.x.xx, Dataset: [NAME], Program: xxxxxx.sas, Output: T_16_1_1_1_XXXXX.rtf, Generated on: DDMONYYYY HH:MM
Page 1 of 1

Table 16.1.1.2.1 Subject Disposition - ITT Primary Population

Variable	GDNF/GDNF (N = XXX) n (%)	Placebo/GDNF (N = XXX) n (%)	Total (N = XXX) n (%)
Completed Initial Extension	xx (xx.x)	xx (xx.x)	xx (xx.x)
Discontinued study during Initial Extension (primary reason for early termination)	xx (xx.x)	xx (xx.x)	xx (xx.x)
Death	xx (xx.x)	xx (xx.x)	xx (xx.x)
Adverse event(s)	xx (xx.x)	xx (xx.x)	xx (xx.x)
Withdrawal by subject	xx (xx.x)	xx (xx.x)	xx (xx.x)
Protocol violation(s)	xx (xx.x)	xx (xx.x)	xx (xx.x)
Lost to follow-up	xx (xx.x)	xx (xx.x)	xx (xx.x)
Physician decision	xx (xx.x)	xx (xx.x)	xx (xx.x)
Other	xx (xx.x)	xx (xx.x)	xx (xx.x)
Missing	xx (xx.x)	xx (xx.x)	xx (xx.x)

Note: Reasons for discontinuation are based on the End of Study CRF page.
Source: Listing xx.x.xx, Dataset: [NAME], Program: xxxxxx.sas, Output: T_16_1_1_2_1_XXXXX.rtf, Generated on: DDMONYYYY HH:MM
Page 1 of 1

Programming Note: Sort in order as on CRF. Include all discontinuation reasons on CRF (even if no subjects discontinued for that reason).

Table 16.1.1.2.2 Subject Disposition - ITT Overall Population

Programming Note: Repeat table for different population.

Table 16.1.2 Protocol Deviations - ITT Overall Population

**Category**<br>**Deviation (brief description)**	**GDNF/GDNF (N = XXX) n (%)**	**Placebo/GDNF (N = XXX) n (%)**	**Total (N = XXX) n (%)**
Subjects with at least one major protocol deviation	xx (xx.x)	xx (xx.x)	xx (xx.x)
[Category 1]	xx (xx.x)	xx (xx.x)	xx (xx.x)
[Deviation 1]	xx (xx.x)	xx (xx.x)	xx (xx.x)
[Deviation 2]	xx (xx.x)	xx (xx.x)	xx (xx.x)
[Category 2]	xx (xx.x)	xx (xx.x)	xx (xx.x)
[Deviation 1]	xx (xx.x)	xx (xx.x)	xx (xx.x)
[Deviation 2]	xx (xx.x)	xx (xx.x)	xx (xx.x)
…			
Subjects with at least one minor protocol deviation	xx (xx.x)	xx (xx.x)	xx (xx.x)
[Category 1]	xx (xx.x)	xx (xx.x)	xx (xx.x)
[Category 2]	xx (xx.x)	xx (xx.x)	xx (xx.x)
…			

Note: For each category and deviation, subjects are included only once, even if they experienced multiple events in that category or deviation. If a subject missed more than one study drug infusion during the Initial Extension, the second missed infusion is classified as major.

Source: Listing xx.x.xx, Dataset: [NAME], Program: xxxxxx.sas, Output: T_16_1_2_XXXXX.rtf, Generated on: DDMONYYYY HH:MM
Page 1 of 1

Programming Note: Categories should be sorted alphabetically.Within category, sort should be by decreasing frequency of deviations in Total group.

Table 16.1.3.1 Demographic Characteristics from Study 2553 - ITT Primary Population

Variable	GDNF/GDNF (N = XXX)	Placebo/GDNF (N = XXX)	Total (N = XXX)
Age (years)			
N	xx	xx	xx
Mean (SD)	xx.x (xx.xx)	xx.x (xx.xx)	xx.x (xx.xx)
Median	xx.x	xx.x	xx.x
Min, Max	xx, xx	xx, xx	xx, xx
Age group [n (%)]			
< 65 years	xx (xx.x)	xx (xx.x)	xx (xx.x)
≥ 65 years	xx (xx.x)	xx (xx.x)	xx (xx.x)
Sex [n (%)]			
Female	xx (xx.x)	xx (xx.x)	xx (xx.x)
Male	xx (xx.x)	xx (xx.x)	xx (xx.x)
Race [n (%)]			
White	xx (xx.x)	xx (xx.x)	xx (xx.x)
Black	xx (xx.x)	xx (xx.x)	xx (xx.x)
Asian	xx (xx.x)	xx (xx.x)	xx (xx.x)
Native Hawaiian or other Pacific Islander	xx (xx.x)	xx (xx.x)	xx (xx.x)
American Indian or Alaska Native	xx (xx.x)	xx (xx.x)	xx (xx.x)
Other	xx (xx.x)	xx (xx.x)	xx (xx.x)
Missing	xx (xx.x)	xx (xx.x)	xx (xx.x)
Ethnicity [n (%)]			
Hispanic or Latino	xx (xx.x)	xx (xx.x)	xx (xx.x)
Not Hispanic or Latino	xx (xx.x)	xx (xx.x)	xx (xx.x)

[a]NART error score is the number of words pronounced incorrectly out of 50 total words.
Source: Listing xx.x.xx, Dataset: [NAME], Program: xxxxxx.sas, Output: T_16_1_3_1_XXXXX.rtf, Generated on: DDMONYYYY HH:MM
Page 1 of 2

Table 16.1.3.1 Demographic Characteristics from Study 2553 - ITT Primary Population (cont.)

Variable	GDNF/GDNF (N = XXX)	Placebo/GDNF (N = XXX)	Total (N = XXX)
Weight at baseline (kg)			
N	xx	xx	xx
Mean (SD)	xx.xx (xx.xxx)	xx.xx (xx.xxx)	xx.xx (xx.xxx)
Median	xx.x	xx.x	xx.x
Min, Max	xx.x, xx.x	xx.x, xx.x	xx.x, xx.x
Height at baseline (m)			
N	xx	xx	xx
Mean (SD)	xx.x (xx.xx)	xx.x (xx.xx)	xx.x (xx.xx)
Median	xx	xx	xx
Min, Max	xx, xx	xx, xx	xx, xx
BMI at baseline (kg/m^2)			
N	xx	xx	xx
Mean (SD)	xx.x (xx.xx)	xx.x (xx.xx)	xx.x (xx.xx)
Median	xx	xx	xx
Min, Max	xx, xx	xx, xx	xx, xx
NART error score (points)[a]			
n	xx	xx	xx
Mean (SD)	xx.x (xx.xx)	xx.x (xx.xx)	xx.x (xx.xx)
Median	xx	xx	xx
Min, Max	xx, xx	xx, xx	xx, xx

[a] NART error score is the number of words pronounced incorrectly out of 50 total words.
Source: Listing xx.x.xx, Dataset: [NAME], Program: xxxxxx.sas, Output: T_16_1_3_1_XXXXX.rtf, Generated on: DDMONYYYY HH:MM
Page 2 of 2

Programming Note: Add “Missing” if necessary to categorical variables.

Table 16.1.3.2 Demographic Characteristics from Study 2553 - ITT Overall Population

Programming Note: Repeat table for different population.

Table 16.1.4.1 Parkinson's Disease History at Screening from Study 2553 - ITT Primary Population

Variable	GDNF/GDNF (N = XXX)	Placebo/GDNF (N = XXX)	Total (N = XXX)
Duration since first PD symptom (years)			
N	xx	xx	xx
Mean (SD)	xx.xx (xx.xxx)	xx.xx (xx.xxx)	xx.xx (xx.xxx)
Median	xx.x	xx.x	xx.x
Min, Max	xx.x, xx.x	xx.x, xx.x	xx.x, xx.x
Duration since PD diagnosis (years)			
N	xx	xx	xx
Mean (SD)	xx.xx (xx.xxx)	xx.xx (xx.xxx)	xx.xx (xx.xxx)
Median	xx.x	xx.x	xx.x
Min, Max	xx.x, xx.x	xx.x, xx.x	xx.x, xx.x
Hoehn and Yahr stage in OFF state [n (%)]			
Stage 0: No signs of disease	xx (xx.x)	xx (xx.x)	xx (xx.x)
Stage 1: Unilateral symptoms only	xx (xx.x)	xx (xx.x)	xx (xx.x)
Stage 1.5: Unilateral and axial involvement	xx (xx.x)	xx (xx.x)	xx (xx.x)
Stage 2: Bilateral symptoms; no impairment of balance	xx (xx.x)	xx (xx.x)	xx (xx.x)
Stage 2.5: Mild bilateral disease with recovery on pull test	xx (xx.x)	xx (xx.x)	xx (xx.x)
Stage 3: Balance impairment; mild to moderate disease; physically independent	xx (xx.x)	xx (xx.x)	xx (xx.x)
Missing	xx (xx.x)	xx (xx.x)	xx (xx.x)
OFF state UPDRS motor score (part III)			
N	xx	xx	xx
Mean (SD)	xx.xx (xx.xxx)	xx.xx (xx.xxx)	xx.xx (xx.xxx)
Median	xx.x	xx.x	xx.x
Min, Max	xx.x, xx.x	xx.x, xx.x	xx.x, xx.x
ON state UPDRS motor score (part III)			
N	xx	xx	xx
Mean (SD)	xx.xx (xx.xxx)	xx.xx (xx.xxx)	xx.xx (xx.xxx)
Median	xx.x	xx.x	xx.x
Min, Max	xx.x, xx.x	xx.x, xx.x	xx.x, xx.x

[a] Percentage change in UPDRS motor score (part III) following a levodopa challenge.
Source: Listing xx.x.xx, Dataset: [NAME], Program: xxxxxx.sas, Output: T_16_1_4_1_XXXXX.rtf, Generated on: DDMONYYYY HH:MM
Page 1 of 2

Table 16.1.4.1 Parkinson's Disease History at Screening from Study 2553 - ITT Primary Population (cont.)

Variable	GDNF/GDNF (N = XXX)	Placebo/GDNF (N = XXX)	Total (N = XXX)
Total daily levodopa dose (mg)			
N	xx	xx	xx
Mean (SD)	xx.xx (xx.xxx)	xx.xx (xx.xxx)	xx.xx (xx.xxx)
Median	xx.x	xx.x	xx.x
Min, Max	xx.x, xx.x	xx.x, xx.x	xx.x, xx.x
Total daily levodopa equivalent dose (mg)			
N	xx	xx	xx
Mean (SD)	xx.xx (xx.xxx)	xx.xx (xx.xxx)	xx.xx (xx.xxx)
…	…	…	…
PD medications [n (%)]			
Levodopa preparations	xx (xx.x)	xx (xx.x)	xx (xx.x)
Dopamine agonists	xx (xx.x)	xx (xx.x)	xx (xx.x)
COMT inhibitors	xx (xx.x)	xx (xx.x)	xx (xx.x)
MAO-B inhibitors	xx (xx.x)	xx (xx.x)	xx (xx.x)
Other	xx (xx.x)	xx (xx.x)	xx (xx.x)
Responsiveness to levodopa[a] (%)			
N	xx	xx	xx
Mean (SD)	xx.xx (xx.xxx)	xx.xx (xx.xxx)	xx.xx (xx.xxx)
…	…	…	…
OFF time per day (hours)			
N	xx	xx	xx
Mean (SD)	xx.xx (xx.xxx)	xx.xx (xx.xxx)	xx.xx (xx.xxx)
…	…	…	…

[a] Percentage change in UPDRS motor score (part III) following a levodopa challenge.
Source: Listing xx.x.xx, Dataset: [NAME], Program: xxxxxx.sas, Output: T_16_1_4_1_XXXXX.rtf, Generated on: DDMONYYYY HH:MM
Page 2 of 2

Programming Note: Add "Missing" if necessary to categorical variables. See Section 5.0 definition of screening levodopa and levodopa equivalent dose.

Table 16.1.4.2 Parkinson's Disease History at Screening from Study 2553 - ITT Overall Population

Programming Note: Repeat table for different population.

Table 16.1.5.1 Concomitant Parkinson's Disease Medications by WHO Drug Dictionary ATC Class and Preferred Name - ITT Primary Population

**ATC Class**<br>**Preferred Name**	**GDNF/GDNF**<br>**(N = XXX)**<br>**n (%)**	**Placebo/GDNF**<br>**(N = XXX)**<br>**n (%)**	**Total**<br>**(N = XXX)**<br>**n (%)**
Subjects with at least one concomitant PD medication	xx (xx.x)	xx (xx.x)	xx (xx.x)
[ATC Class 1]	xx (xx.x)	xx (xx.x)	xx (xx.x)
[Preferred Name 1]	xx (xx.x)	xx (xx.x)	xx (xx.x)
[Preferred Name 2]	xx (xx.x)	xx (xx.x)	xx (xx.x)
[ATC Class 2]	xx (xx.x)	xx (xx.x)	xx (xx.x)
[Preferred Name 1]	xx (xx.x)	xx (xx.x)	xx (xx.x)
[Preferred Name 2]	xx (xx.x)	xx (xx.x)	xx (xx.x)
…			

Note: Concomitant medications are defined as any medications ongoing at the start of open-label study medication dosing or with a start date on or after the first open-label study medication dose date. Medications are coded using WHO Drug Dictionary Enhanced version 2016Mar01. For each ATC class and preferred name, subjects are included only once.
Source: Listing xx.x.xx, Dataset: [NAME], Program: xxxxxx.sas, Output: T_16_1_3_XXXXX.rtf, Generated on: DDMONYYYY HH:MM
Page x of y

Programming Note: Sort alphabetically for both ATC class and preferred name.

Table 16.1.5.2 Other Concomitant Medications by WHO Drug Dictionary ATC Class and Preferred Name - ITT Primary Population

**ATC Class**<br>**Preferred Name**	**GDNF/GDNF**<br>**(N = XXX)**<br>**n (%)**	**Placebo/GDNF**<br>**(N = XXX)**<br>**n (%)**	**Total**<br>**(N = XXX)**<br>**n (%)**
Subjects with at least one other concomitant medication	xx (xx.x)	xx (xx.x)	xx (xx.x)
[ATC Class 1]	xx (xx.x)	xx (xx.x)	xx (xx.x)
[Preferred Name 1]	xx (xx.x)	xx (xx.x)	xx (xx.x)
[Preferred Name 2]	xx (xx.x)	xx (xx.x)	xx (xx.x)
[ATC Class 2]	xx (xx.x)	xx (xx.x)	xx (xx.x)
[Preferred Name 1]	xx (xx.x)	xx (xx.x)	xx (xx.x)
[Preferred Name 2]	xx (xx.x)	xx (xx.x)	xx (xx.x)
…			

Note: Concomitant medications are defined as any medications ongoing at the start of open-label study medication dosing or with a start date on or after the first open-label study medication dose date. Medications are coded using WHO Drug Dictionary Enhanced version 2016Mar01. For each ATC class and preferred name, subjects are included only once.
Source: Listing xx.x.xx, Dataset: [NAME], Program: xxxxxx.sas, Output: T_16_1_3_XXXXX.rtf, Generated on: DDMONYYYY HH:MM
Page x of y

Programming Note: Sort alphabetically for both ATC class and preferred name.

16.2 Efficacy Data Tables

16.2.1 UPDRS Primary Efficacy Tables

Table 16.2.1.1 OFF State UPDRS Motor Score (Part III): Change and Percentage Change from Baseline to Week 80/e40 – MMRM, ITT Primary Population

	GDNF/GDNF (N = XXX)			Placebo/GDNF (N = XXX)		
Visit<br>Statistic	Value	Change From Baseline	Percentage Change From Baseline	Value	Change From Baseline	Percentage Change From Baseline
Week 0 (Baseline)						
N	xx			xx		
Mean (SD)	xx.xx (xx.xxx)			xx.xx (xx.xxx)		
Median	xx.x			xx.x		
Min, Max	xx.x, xx.x			xx.x, xx.x		
Week 40/e0						
N	xx	xx	xx	xx	xx	xx
Mean (SD)	xx.xx (xx.xxx)	xx.xx (xx.xxx)	xx.xx (xx.xxx)	xx.xx (xx.xxx)	xx.xx (xx.xxx)	xx.xx (xx.xxx)
Median	xx.x	xx.x	xx.x	xx.x	xx.x	xx.x
Min, Max	xx.x, xx.x	xx.x, xx.x	xx.x, xx.x	xx.x, xx.x	xx.x, xx.x	xx.x, xx.x
Week 80/e40						
N	xx	xx	xx	xx	xx	xx
Mean (SD)	xx.xx (xx.xxx)	xx.xx (xx.xxx)	xx.xx (xx.xxx)	xx.xx (xx.xxx)	xx.xx (xx.xxx)	xx.xx (xx.xxx)
Median	xx.x	xx.x	xx.x	xx.x	xx.x	xx.x
Min, Max	xx.x, xx.x	xx.x, xx.x	xx.x, xx.x	xx.x, xx.x	xx.x, xx.x	xx.x, xx.x
Least squares mean[a] (95% CI)		xx.x (xx.x, xx.x)	xx.x (xx.x, xx.x)		xx.x (xx.x, xx.x)	xx.x (xx.x, xx.x)
Least squares mean difference versus placebo[a] (95% CI)		xx.x (xx.x, xx.x)	xx.x (xx.x, xx.x)			
p-value[a]		0.xxxx	0.xxxx			

[a] Estimates are from a mixed-effect model with repeated measures (MMRM) with baseline UPDRS score as a covariate, treatment group and visit and treatment group*visit as fixed effects, and subject within treatment group as a random effect.
Note: Lower scores represent better functioning. End of study visit data are included in the appropriate visit week. For subject 45, items 22, 27, 28, 29 and 30 are excluded from the score.
Source: Listing xx.x.xx, Dataset: [NAME], Program: xxxxxx.sas, Output: xxxx.rtf, Generated on: DDMONYYYY HH:MM
Page x of y

Table 16.2.1.2 OFF State UPDRS Motor Score (Part III): Change and Percentage Change from Baseline to Week 80/e40 – MMRM, ITT Overall Population

Programming Note: Repeat Table 16.2.1.1 for different population.

16.2.2 UPDRS Secondary Efficacy Tables

Table 16.2.2.1 ON State UPDRS Motor Score (Part III): Change and Percentage Change from Baseline to Week 80/e40 – MMRM, ITT Overall Population

Programming Note: Repeat of Table 16.2.1.1, but for ON state parameters and ITT Overall Population.

Table 16.2.2.2.1 OFF State UPDRS ADL Score (Part II): Change and Percentage Change from Baseline to Week 80/e40 – MMRM, ITT Primary Population

Programming Note: Repeat of Table 16.2.1.1, but for OFF state ADL (Part II) score. Change footnote regarding subject 45 to: "Data for subject 45 are excluded from analysis."

Table 16.2.2.2.2 OFF State UPDRS ADL Score (Part II): Change and Percentage Change from Baseline to Week 80/e40 – MMRM, ITT Overall Population

Programming Note: Repeat of Table 16.2.2.2.1, but with different population.

Table 16.2.2.3 ON State UPDRS ADL Score (Part II): Change and Percentage Change from Baseline to Week 80/e40 – MMRM, ITT Overall Population

Programming Note: Repeat of Table 16.2.2.2.1, but for ON state ADL (Part II) score and with different population.

Table 16.2.2.4.1 OFF State UPDRS Total Score: Change and Percentage Change from Baseline to Week 80/e40 – MMRM, ITT Primary Population

Programming Note: Repeat of Table 16.2.1.1, but for OFF state total score. Change footnote regarding subject 45 to: "Data for subject 45 are excluded from analysis."

Table 16.2.2.4.2 OFF State UPDRS Total Score: Change and Percentage Change from Baseline to Week 80/e40 – MMRM, ITT Overall Population

Programming Note: Repeat of Table 16.2.2.4.1, but with different population,

Table 16.2.2.5 ON State UPDRS Total Score: Change and Percentage Change from Baseline to Week 80/e40 – MMRM, ITT Overall Population

Programming Note: Repeat of Table 16.2.2.4.1, but for ON state total score and different population."

Table 16.2.2.6.1 OFF State UPDRS Motor Score (Part III): Change from Baseline to Week 40/e0 for GDNF/GDNF Group Compared to Change from Baseline to Week 80/e40 for Placebo/GDNF Group – MMRM, ITT Overall Population

	GDNF/GDNF (N = XXX)		Placebo/GDNF (N = XXX)	
**Visit**<br>**Statistic**	**Value**	**Change From Baseline**	**Value**	**Change From Baseline**
Week 0 (Baseline)				
N	xx		xx	
Mean (SD)	xx.xx (xx.xxx)		xx.xx (xx.xxx)	
Median	xx.x		xx.x	
Min, Max	xx.x, xx.x		xx.x, xx.x	
Week 40/e0				
N	xx	xx		
Mean (SD)	xx.xx (xx.xxx)	xx.xx (xx.xxx)		
Median	xx.x	xx.x		
Min, Max	xx.x, xx.x	xx.x, xx.x		
Week 80/e40				
N			xx	xx
Mean (SD)			xx.xx (xx.xxx)	xx.xx (xx.xxx)
Median			xx.x	xx.x
Min, Max			xx.x, xx.x	xx.x, xx.x
Least squares mean[a] (95% CI)		xx.x (xx.x, xx.x)		xx.x (xx.x, xx.x)
Least squares mean difference versus placebo[a] (95% CI)		xx.x (xx.x, xx.x)		
p-value[a]		0.xxxx		

[a] Estimates are from a mixed-effect model with repeated measures (MMRM) with baseline UPDRS score as a covariate, treatment group and visit and treatment group*visit as fixed effects, and subject within treatment group as a random effect.
Note: Lower scores represent better functioning. End of study visit data are included in the appropriate visit week. For subject 45, items 22, 27, 28, 29 and 30 are excluded from the score.
Source: Listing xx.x.xx, Dataset: [NAME], Program: xxxxxx.sas, Output: xxxx.rtf, Generated on: DDMONYYYY HH:MM
Page x of y

Table 16.2.2.6.2 OFF State UPDRS ADL Score (Part II): Change from Baseline to Week 40/e0 for GDNF/GDNF Group Compared to Change from Baseline to Week 80/e40 for Placebo/GDNF Group – MMRM, ITT Overall Population

Programming Note: Repeat of Table 16.2.2.6.1. Change footnote regarding subject 45 to: "Data for subject 45 are excluded from analysis."

Table 16.2.2.6.3 OFF State UPDRS Total Score: Change from Baseline to Week 40/e0 for GDNF/GDNF Group Compared to Change from Baseline to Week 80/e40 for Placebo/GDNF Group – MMRM, ITT Overall Population

Programming Note: Repeat of Table 16.2.2.6.1. Change footnote regarding subject 45 to: "Data for subject 45 are excluded from analysis."

Table 16.2.2.7.1 OFF State UPDRS Motor Score (Part III): Change from Baseline to Week 80/e40 for GDNF/GDNF Group Compared to Change from Baseline to Week 40/e0 for Placebo/GDNF Group – MMRM, ITT Overall Population

Programming Note: Repeat Table 16.2.2.6.1 for different endpoint and population. Apart from baseline, present only Week 80/e40 for GDNF/GDNF and Week 40/e0 for Placebo/GDNF.

Table 16.2.2.7.2 OFF State UPDRS ADL Score (Part II): Change from Baseline to Week 80/e40 for GDNF/GDNF Group Compared to Change from Baseline to Week 40/e0 for Placebo/GDNF Group – MMRM, ITT Overall Population

Programming Note: Repeat of Table 16.2.2.6.1. Apart from baseline, present only Week 80/e40 for GDNF/GDNF and Week 40/e0 for Placebo/GDNF. Change footnote regarding subject 45 to: "Data for subject 45 are excluded from analysis."

Table 16.2.2.7.3 OFF State UPDRS Total Score: Change from Baseline to Week 80/e40 for GDNF/GDNF Group Compared to Change from Baseline to Week 40/e0 for Placebo/GDNF Group – MMRM, ITT Overall Population

Programming Note: Repeat of Table 16.2.2.6.1. Apart from baseline, present only Week 80/e40 for GDNF/GDNF and Week 40/e0 for Placebo/GDNF. Change footnote regarding subject 45 to: "Data for subject 45 are excluded from analysis."

16.2.3 PD DIARY SECONDARY EFFICACY TABLES

Table 16.2.3.1 Motor Fluctuation Diary Ratings by Visit: Change from Baseline – MMRM, ITT Primary Population

Parameter<br>Visit<br>Statistic	GDNF/GDNF (N = XXX)<br>Value	 Change From Baseline	Placebo/GDNF (N = XXX)<br>Value	 Change From Baseline
OFF time per day (hours)				
Week 0 (Baseline)				
n	xx		xx	
Mean (SD)	xx.xx (xx.xxx)		xx.xx (xx.xxx)	
Median	xx.x		xx.x	
Min, Max	xx.x, xx.x		xx.x, xx.x	
Week 40/e0				
n	xx	xx	xx	xx
Mean (SD)	xx.xx (xx.xxx)	xx.xx (xx.xxx)	xx.xx (xx.xxx)	xx.xx (xx.xxx)
Median	xx.x	xx.x	xx.x	xx.x
Min, Max	xx.x, xx.x	xx.x, xx.x	xx.x, xx.x	xx.x, xx.x
Week 80/e40				
n	xx	xx	xx	xx
Mean (SD)	xx.xx (xx.xxx)	xx.xx (xx.xxx)	xx.xx (xx.xxx)	xx.xx (xx.xxx)
Median	xx.x	xx.x	xx.x	xx.x
Min, Max	xx.x, xx.x	xx.x, xx.x	xx.x, xx.x	xx.x, xx.x
Least squares mean[a] (95% CI)		xx.x (xx.x, xx.x)		xx.x (xx.x, xx.x)
Least squares mean difference versus placebo[a] (95% CI)		xx.x (xx.x, xx.x)		
p-value[a]		0.xxxx		

[a] Estimates are from a mixed-effect model with repeated measures (MMRM) with baseline variable as a covariate, treatment group and visit and treatment group*visit as fixed effects, and subject within treatment group as a random effect.
Note: End of study visit data are included in the appropriate visit week.
Note: Total good-quality ON time per day is defined as the sum of ON time per day without dyskinesias + ON time per day with non-troublesome dyskinesias.

Source: Listing xx.x.xx, Dataset: [NAME], Program: xxxxxx.sas, Output: xxxx.rtf, Generated on: DDMONYYYY HH:MM
Page x of y

Programming Note: PD diary parameters are OFF time per day, total good-quality ON time per day (sum of ON time per day without dyskinesias + ON time per day with non-troublesome dyskinesias), ON time per day without dyskinesias, ON time per day with non-troublesome dyskinesias, and ON time per day with troublesome dyskinesias. Visits are baseline, Week 40/e0, and Week 80/e40. Statistics are for Week 80/e40 only.

Table 16.2.3.2 Motor Fluctuation Diary Ratings by Visit: Change from Baseline – MMRM, ITT Overall Population

Programming Note: Repeat above table for different population. Statistics are for Week 80/e40 only.

Table 16.2.3.3.1 Treatment Response at Week 40/e0 and Week 80/e40 - ITT Overall Population

Treatment Response Criteria	Visit	GDNF/GDNF (N = XXX) n (%)	Placebo/GDNF (N = XXX) n (%)	Total (N = XXX) n (%)
Decrease from baseline by >=10 points in OFF state UPDRS motor score (part III)	Week 40/e0	xx (xx.x)	xx (xx.x)	xx (xx.x)
p-value		0.xxxx		
	Week 80/e40	xx (xx.x)	xx (xx.x)	xx (xx.x)
p-value		0.xxxx		
Increase from baseline by >=1 hour in total good-quality ON time per day (ON without dyskinesias or ON with non-trouble-some dyskinesias)	Week 40/e0	xx (xx.x)	xx (xx.x)	xx (xx.x)
p-value		0.xxxx		
	Week 80/e40	xx (xx.x)	xx (xx.x)	xx (xx.x)
p-value		0.xxxx		
Both of the above criteria	Week 40/e0	xx (xx.x)	xx (xx.x)	xx (xx.x)
p-value		0.xxxx		
	Week 80/e40	xx (xx.x)	xx (xx.x)	xx (xx.x)
p-value		0.xxxx		

Note: For subject 45, items 22, 27, 28, 29 and 30 are excluded from the UPDRS score. P-values are from a Fisher's exact test.

Source: Listing xx.x.xx, Dataset: [NAME], Program: xxxxxx.sas, Output: T_16_X_XXXXX.rtf, Generated on: DDMONYYYY HH:MM
Page x of y

Table 16.2.3.3.2 UPDRS Responder at Week 80/e40 Compared to Week 40/e0 - ITT Overall Population

	GDNF/GDNF (N = XXX)		Placebo/GDNF (N = XXX)	
	UPDRS Responder at Week 80/e40		UPDRS Responder at Week 80/e40	
**UPDRS Responder at Week 40/e0**	**Yes n (%)**	**No n (%)**	**Yes n (%)**	**No n (%)**
Yes	xxx (xx.x)	xxx (xx.x)	xxx (xx.x)	xxx (xx.x)
No	xxx (xx.x)	xxx (xx.x)	xxx (xx.x)	xxx (xx.x)

Note: UPDRS responder is defined as a decrease from baseline by >=10 points in OFF state UPDRS motor score (part III). For subject 45, items 22, 27, 28, 29 and 30 are excluded from the UPDRS score.

Source: Listing xx.x.xx, Dataset: [NAME], Program: xxxxxx.sas, Output: xxxx.rtf, Generated on: DDMONYYYY HH:MM
Page x of y

16.2.4 Supplementary Efficacy Tables

Table 16.2.4.1 OFF and ON State Timed Walking Test by Visit: Change from Baseline – MMRM, ITT Overall Population

Parameter<br>Visit<br>Statistic	GDNF/GDNF (N = XXX)<br>Value	 Change From Baseline	Placebo/GDNF (N = XXX)<br>Value	 Change From Baseline
OFF state timed walking test (seconds)				
Week 0 (Baseline)				
n	xx		xx	
Mean (SD)	xx.xx (xx.xxx)		xx.xx (xx.xxx)	
Median	xx.x		xx.x	
Min, Max	xx.x, xx.x		xx.x, xx.x	
Week 40/e0				
n	xx	xx	xx	xx
Mean (SD)	xx.xx (xx.xxx)	xx.xx (xx.xxx)	xx.xx (xx.xxx)	xx.xx (xx.xxx)
Median	xx.x	xx.x	xx.x	xx.x
Min, Max	xx.x, xx.x	xx.x, xx.x	xx.x, xx.x	xx.x, xx.x
Week 80/e40				
n	xx	xx	xx	xx
Mean (SD)	xx.xx (xx.xxx)	xx.xx (xx.xxx)	xx.xx (xx.xxx)	xx.xx (xx.xxx)
Median	xx.x	xx.x	xx.x	xx.x
Min, Max	xx.x, xx.x	xx.x, xx.x	xx.x, xx.x	xx.x, xx.x
Least squares mean[a] (95% CI)		xx.x (xx.x, xx.x)		xx.x (xx.x, xx.x)
Least squares mean difference versus placebo[a] (95% CI)		xx.x (xx.x, xx.x)		
p-value[a]		0.xxxx		

[a] Estimates are from a mixed-effect model with repeated measures (MMRM) with baseline time as a covariate, treatment group and visit and treatment group*visit as fixed effects, and subject within treatment group as a random effect.
Note: Shorter times represent better function. Two trials per state per visit are averaged for analysis. If only the first trial is completed for a particular state, then the second trial result will be imputed with the worst non-missing second trial result from all subjects for that particular state during the study. If both trials are missing, then the endpoint is not reported for that visit. End of study visit data are included in the appropriate visit week. Data for subject 45 are excluded from analysis.
Source: Listing xx.x.xx, Dataset: [NAME], Program: xxxxxx.sas, Output: xxxx.rtf, Generated on: DDMONYYYY HH:MM
Page x of y

Programming Note: Timed walking test parameters are OFF state timed walking test and ON state timed walking test (seconds). Visits are baseline, Week 40/e0, and Week 80/e40. Statistics are for Week 80/e40 only.

Table 16.2.4.2 OFF and ON State Timed Tapping Test by Visit: Change from Baseline – MMRM, ITT Overall Population

Programming Note: Repeat Table 16.2.4.1, but for time tapping test by visit. Timed tapping test parameters are OFF state timed tapping test, and ON state timed tapping test . Visits are baseline, Week 40/e0, and Week 80/e40*. Statistics are for Week 80/e40 only. Replace footnote text with "…(MMRM) with baseline number of taps as a covariate…". Replace note with: "More taps represent better function. Four trials per state for each visit are averaged for analysis. If any trial is not completed for a particular state, then the trial result will be imputed with the worst non-missing applicable trial result (for example, first left hand trial) from all subjects for that particular state during the study. If all four trials are missing, then the endpoint is not reported for that visit. End of study visit data are included in the appropriate visit week."*

Table 16.2.4.3 NMSS Score by Visit: Change from Baseline – MMRM, ITT Overall Population

Parameter	GDNF/GDNF (N = XXX)		Placebo/GDNF (N = XXX)	
Visit<br>Statistic	Value	Change From Baseline	Value	Change From Baseline
NMSS Total Score				
Screening (Baseline)				
n	xx		xx	
Mean (SD)	xx.xx (xx.xxx)		xx.xx (xx.xxx)	
Median	xx.x		xx.x	
Min, Max	xx.x, xx.x		xx.x, xx.x	
Week 40/e0				
…				
Week 80/e40				
n	xx	xx	xx	xx
Mean (SD)	xx.xx (xx.xxx)	xx.xx (xx.xxx)	xx.xx (xx.xxx)	xx.xx (xx.xxx)
Median	xx.x	xx.x	xx.x	xx.x
Min, Max	xx.x, xx.x	xx.x, xx.x	xx.x, xx.x	xx.x, xx.x
Least squares mean[a] (95% CI)		xx.x (xx.x, xx.x)		xx.x (xx.x, xx.x)
Least squares mean difference versus placebo[a] (95% CI)		xx.x (xx.x, xx.x)		
p-value[a]		0.xxxx		

[a] Estimates are from a mixed-effect model with repeated measures (MMRM) with baseline score as a covariate, treatment group and visit and treatment group*visit as fixed effects, and subject within treatment group as a random effect.
Note: The NMSS is a 30-item interview-based scale that rates non-motor symptoms that occurred in the preceding month in 9 domains. Each item is rated from 0 (none) to 3 (severe) for severity and from 1 (rarely) to 4 (very frequent) for frequency. The maximum score for an individual item is 12. The higher the score, the worse the subject's condition. The maximum NMSS total score is 360. Missing individual item scores are imputed using LOCF. End of study visit data are included in the appropriate visit week. Data for subject 45 are excluded from analysis.
Source: Listing xx.x.xx, Dataset: [NAME], Program: xxxxxx.sas, Output: xxxx.rtf, Generated on: DDMONYYYY HH:MM
Page x of y

Programming Note: NMSS parameters are Cardiovascular including falls domain, Sleep/fatigue domain, Mood/cognition domain, Perceptual problems/hallucinations domain; Attention/memory domain, Gastrointestinal tract domain, Urinary domain, Sexual function domain, Miscellaneous domain, and NMSS total score. Visits are baseline, Week 40/e0, and Week 80/e40. Statistics are for Week 80/e40 only.

Table 16.2.4.4 PDQ-39 Score: Change from Baseline to Week 80/e40 – ANCOVA, ITT Overall Population

Parameter<br>Visit<br>Statistic	GDNF/GDNF (N = XXX)		Placebo/GDNF (N = XXX)	
	Value	Change From Baseline	Value	Change From Baseline
Single Index (Total) PDQ-39 Score				
Screening (Baseline)				
n	xx		xx	
Mean (SD)	xx.xx (xx.xxx)		xx.xx (xx.xxx)	
Median	xx.x		xx.x	
Min, Max	xx.x, xx.x		xx.x, xx.x	
Week 40/e0				
n	xx	xx	xx	xx
Mean (SD)	xx.xx (xx.xxx)	xx.xx (xx.xxx)	xx.xx (xx.xxx)	xx.xx (xx.xxx)
Median	xx.x	xx.x	xx.x	xx.x
Min, Max	xx.x, xx.x	xx.x, xx.x	xx.x, xx.x	xx.x, xx.x
Week 80/e40				
n	xx	xx	xx	xx
Mean (SD)	xx.xx (xx.xxx)	xx.xx (xx.xxx)	xx.xx (xx.xxx)	xx.xx (xx.xxx)
Median	xx.x	xx.x	xx.x	xx.x
Min, Max	xx.x, xx.x	xx.x, xx.x	xx.x, xx.x	xx.x, xx.x
Mean difference versus placebo[a] (95% CI)		xx.x (xx.x, xx.x)		
p-value[a]		0.xxxx		

[a] Estimates are from an analysis of covariance (ANCOVA) model with baseline score as a covariate and treatment group as a factor.
Note: The PDQ-39 is a self-administered 39-item PD-specific scale that rates symptoms that occurred in the preceding month in 8 dimensions. Each item is rated from 0 (never) to 4 (always) for frequency. The score for each dimension is the average of responses in the dimension, weighted by the number of questions and multiplied by 100. PDQ-39 dimension and total scores range from 0 to 100 (0 = no problem, 100 = problem as worse as possible). The higher the score, the worse the subject's condition. Only subjects with a Week 40 value are included in the observed data analysis. If an individual question response is missing, then that dimension score and the single index (total) score are also missing. Data for subject 45 are excluded from analysis.
Source: Listing xx.x.xx, Dataset: [NAME], Program: xxxxxx.sas, Output: xxxx.rtf, Generated on: DDMONYYYY HH:MM
Page x of y

Programming Note: PDQ-39 parameters are Mobility dimension, ADL dimension, Emotional well-being dimension, Stigma dimension, Social support dimension, Cognitions dimension, Communication dimension, Bodily discomfort dimension, and Single index (total) PDQ-39 score. Visits are baseline, Week 40/e0, and Week 80/e40.

Table 16.2.4.5.1 EQ-5D Questionnaire: Baseline, Week 40/e0, and Week 80/e40 – Observed Data, ITT Overall Population

**EQ-5D Dimension**<br>**Visit**<br>**Response Level**	**GDNF/GDNF**<br>**(N = XXX)**<br>**n (%)**	**Placebo/GDNF**<br>**(N = XXX)**<br>**n (%)**
Mobility		
Screening (Baseline)		
No problem	xx (xx.x)	xx (xx.x)
Moderate problem	xx (xx.x)	xx (xx.x)
Severe problem	xx (xx.x)	xx (xx.x)
Week 40/e0		
No problem	xx (xx.x)	xx (xx.x)
Moderate problem	xx (xx.x)	xx (xx.x)
Severe problem	xx (xx.x)	xx (xx.x)
Week 80/e40		
No problem	xx (xx.x)	xx (xx.x)
Moderate problem	xx (xx.x)	xx (xx.x)
Severe problem	xx (xx.x)	xx (xx.x)
Self-care		
Screening (Baseline)		
No problem	xx (xx.x)	xx (xx.x)
Moderate problem	xx (xx.x)	xx (xx.x)
Severe problem	xx (xx.x)	xx (xx.x)
…		

Note: The EQ-5D is a self-administered 5-item scale with three possible answers for each item (1=no problem, 2=moderate problem, 3=severe problem), and a visual analog scale ranging from 0 to 100, where 0 indicates worst health and 100 indicates best health. Only subjects with a Week 40 value are included in the observed data analysis. Data for subject 45 are excluded from analysis.
Source: Listing xx.x.xx, Dataset: [NAME], Program: xxxxxx.sas, Output: T_16_2_4_5_1_XXXXX.rtf, Generated on: DDMONYYYY HH:MM
Page 1 of 1

Programming Note: Sort in order as on CRF. Continue for Self-care, Usual Activities, Pain / Discomfort, and Anxiety / Depression. Visits are baseline, Week 40/e0, and Week 80/e40. EQ-5D parameters are the frequency counts and percentages of subjects with the different answers to the 5 questions (categorical) and the visual analog scale score (continuous). Visual analog scale data is in the next table.

Table 16.2.4.5.2 EQ-5D Visual Analog Scale: Change from Baseline to Week 80/e40 – ANCOVA, ITT Overall Population

Programming Note: Repeat ANCOVA table for EQ-5D. Report observed data for baseline, Week 40/e0, Week 80/e40, and change from baseline summary statistics for visual analog scale (with mean difference, 95% CI, and p-value). Visits are baseline, Week 40/e0 and Week 80/e40. Add note: "The EQ-5D is a self-administered 5-item scale with three possible answers for each item (1=no problem, 2=moderate problem, 3=severe problem), and a visual analog scale ranging from 0 to 100, where 0 indicates worst health and 100 indicates best health. Only subjects with a Week 80/e40 value are included in the analysis. Data for subject 45 are excluded from analysis" EQ-5D parameters are the frequency counts and percentages of subjects with the different answers to the 5 questions (categorical) and the visual analog scale score (continuous).

Table 16.2.4.6 SNAQ Score: Change from Baseline to Week 80/e40 – ANCOVA, ITT Overall Population

Programming Note: Repeat ANCOVA table for SNAQ. SNAQ has one total SNAQ score. Visits are baseline, Week 40/e0 and Week 80/e40. Add note: "The SNAQ is a self-administered 4-question instrument with total scores ranging from 4 to 20 (4=poor appetite, 20=good appetite). Only subjects with a Week 80/e40 value are included in the analysis. If an individual question is not answered, then the total score is considered missing."

Table 16.2.4.7 Total Daily Levodopa Dose (mg): Change From Baseline to Week 80/e40 – ANCOVA, ITT Overall Population

	GDNF/GDNF (N = XXX)		Placebo/GDNF (N = XXX)	
**Visit Statistic**	**Value**	**Change From Baseline**	**Value**	**Change From Baseline**
Week 0 (Baseline)				
n	xx		xx	
Mean (SD)	xx.xx (xx.xxx)		xx.xx (xx.xxx)	
Median	xx.x		xx.x	
Min, Max	xx.x, xx.x		xx.x, xx.x	
Week 40/e0				
n	xx	xx	xx	xx
Mean (SD)	xx.xx (xx.xxx)	xx.xx (xx.xxx)	xx.xx (xx.xxx)	xx.xx (xx.xxx)
Median	xx.x	xx.x	xx.x	xx.x
Min, Max	xx.x, xx.x	xx.x, xx.x	xx.x, xx.x	xx.x, xx.x
Week 80/e40				
n	xx	xx	xx	xx
Mean (SD)	xx.xx (xx.xxx)	xx.xx (xx.xxx)	xx.xx (xx.xxx)	xx.xx (xx.xxx)
Median	xx.x	xx.x	xx.x	xx.x
Min, Max	xx.x, xx.x	xx.x, xx.x	xx.x, xx.x	xx.x, xx.x
Mean difference versus placebo[a] (95% CI)		xx.x (xx.x, xx.x)		
p-value[a]		0.xxxx		

[a] Estimates are from an analysis of covariance (ANCOVA) model with baseline levodopa dose as a covariate and treatment group as a factor.
Note: Only subjects with a Week 80/e40 levodopa value are included in the analysis.
Source: Listing xx.x.xx, Dataset: [NAME], Program: xxxxxx.sas, Output: xxxx.rtf, Generated on: DDMONYYYY HH:MM
Page x of y

Programming Note: See Section 5.0 for definition of levodopa dose.

Table 16.2.4.8 Total Daily Levodopa Equivalent Dose (mg): Change From Baseline to Week 80/e40 – ANCOVA, ITT Overall Population

	GDNF/GDNF (N = XXX)		Placebo/GDNF (N = XXX)	
**Visit Statistic**	**Value**	**Change From Baseline**	**Value**	**Change From Baseline**
Week 0 (Baseline)				
n	xx		xx	
Mean (SD)	xx.xx (xx.xxx)		xx.xx (xx.xxx)	
Median	xx.x		xx.x	
Min, Max	xx.x, xx.x		xx.x, xx.x	
Week 40/e0				
n	xx	xx	xx	xx
Mean (SD)	xx.xx (xx.xxx)	xx.xx (xx.xxx)	xx.xx (xx.xxx)	xx.xx (xx.xxx)
Median	xx.x	xx.x	xx.x	xx.x
Min, Max	xx.x, xx.x	xx.x, xx.x	xx.x, xx.x	xx.x, xx.x
Week 80/e40				
n	xx	xx	xx	Xx
Mean (SD)	xx.xx (xx.xxx)	xx.xx (xx.xxx)	xx.xx (xx.xxx)	xx.xx (xx.xxx)
Median	xx.x	xx.x	xx.x	xx.x
Min, Max	xx.x, xx.x	xx.x, xx.x	xx.x, xx.x	xx.x, xx.x
Mean difference versus placebo[a] (95% CI)		xx.x (xx.x, xx.x)		
p-value[a]		0.xxxx		

[a] Estimates are from an analysis of covariance (ANCOVA) model with baseline levodopa equivalent dose as a covariate and treatment group as a factor.
Note: Only subjects with a Week 80/e40 levodopa equivalent value are included in the analysis.
Source: Listing xx.x.xx, Dataset: [NAME], Program: xxxxxx.sas, Output: xxxx.rtf, Generated on: DDMONYYYY HH:MM
Page x of y

Programming Note: See Section 5.0 for definition of levodopa equivalent dose.

16.3 Imaging Tables

Table 16.3.1 Volume of Distribution of Infusate as Determined by Contrast-Enhanced T1-Weighted MRI: Change from Baseline to Week 80/e40 – ANCOVA, ITT Primary Population

Programming Note: Repeat ANCOVA Table 16.2.4.4. Visits are Healing phase (Baseline) (last test infusion at the end of the healing phase), Week 40/e0, and Week 80/e40. The post-infusion time point at each visit is used. Parameter is volume of distribution (in mL), separately for left and right hemispheres, as determined by contrast-enhanced T1-weighted MRI. Table will have 2 pages, one for left and right hemisphere; please subtitle each page clearly.

Table 16.3.2.1 Volume of Interest Coverage as Determined by Contrast-Enhanced T1-Weighted MRI: Change from Baseline to Week 80/e40 – ANCOVA, ITT Primary Population

Programming Note: Repeat observed data ANCOVA Table 16.2.4.4. Visits are Healing phase (Baseline) (last test infusion at the end of the healing phase), Week 40/e0, and Week 80/e40. The post-infusion time point at each visit is used. Parameter is VOI coverage as a percentage of total VOI, separately for left putamen and right putamen and for both putamina combined. Table will have 3 pages, one for each of left and right putamen and one for both putamina combined; please subtitle each page clearly.

Table 16.3.2.2 Total Putamenal Coverage as Determined by Contrast-Enhanced T1-Weighted MRI: Change from Baseline to Week 80/e40 – ANCOVA, ITT Primary Population

Programming Note: Repeat observed data ANCOVA Table 16.2.4.4. Visits are Randomization (Baseline), Week 40/e0, and Week 80/e40. The post-infusion time point at each visit is used. Parameter is total putamenal coverage as a percentage of total putamenal volume, separately for left putamen and right putamen and for both putamina combined. Table will have 3 pages, one for each of left and right putamen and one for both putamina combined; please subtitle each page clearly.

Table 16.3.3.1 Correlation Analyses of Percentage Change from Baseline to Week 80/e40 in OFF State UPDRS Motor Score (Part III) to Volume of Interest Coverage and Total Putamenal Coverage at Baseline as Determined by Contrast-Enhanced T1-Weighted MRI – ITT Primary Population

Parameters	GDNF/GDNF (N = XXX) n Spearman Rank Correlation (95% CI) p-value	Placebo/GDNF (N = XXX) n Spearman Rank Correlation (95% CI) p-value
Percentage change from baseline to Week 80/e40 in OFF state UPDRS motor score (part III) versus VOI coverage at baseline, both putamina combined	xx<br>0.xxx (0.xxx, 0.xxx)<br>0.xxxx	xx<br>0.xxx (0.xxx, 0.xxx)<br>0.xxxx
Percentage change from baseline to Week 80/e40 in OFF state UPDRS motor score (part III) versus Total putamenal coverage at baseline, both putamina combined	xx<br>0.xxx (0.xxx, 0.xxx)<br>0.xxxx	xx<br>0.xxx (0.xxx, 0.xxx)<br>0.xxxx

Note: For subject 45, items 22, 27, 28, 29 and 30 are excluded from the UPDRS score.
Source: Listing xx.x.xx, Dataset: [NAME], Program: xxxxxx.sas, Output: xxxx.rtf, Generated on: DDMONYYYY HH:MM
Page x of y

Table 16.3.3.2 Correlation Analysis of Percentage Change from Baseline to Week 80/e40 in OFF State UPDRS Motor Score (Part III) to Change from Baseline to Week 40/e0 in ^{18}F-DOPA Uptake as Determined by PET Scan – ITT Primary Population

Programming Note: Repeat above Table 16.3.3.1 for parameters "Percentage change from baseline to Week 80/e40 in OFF state UPDRS Motor score (Part III)" and "Change from Baseline to Week 40/e0 in ^{18}F-DOPA Uptake." Each region will be on a separate page.

Table 16.3.3.3 Correlation Analysis of Percentage Change from Baseline to Week 80/e40 in OFF State UPDRS Motor Score (Part III) to Change from Baseline to Week 40/e0 in ^{18}F-DOPA Uptake as Determined by PET Scan – ITT Overall Population

Programming Note: Repeat Table 16.3.3.2 for different population.

16.4 Safety Data Tables

16.4.1 Exposure Data Tables

Table 16.4.1.1 Exposure to Study Medication - Safety Overall Population

Extension Part<br>Variable	GDNF/GDNF<br>(N = XXX)	Placebo/GDNF<br>(N = XXX)	Total<br>(N = XXX)
Initial Extension			
Number of infusions of study medication			
n	xx	Xx	Xx
Mean (SD)	xx.x (xx.xx)	xx.x (xx.xx)	xx.x (xx.xx)
Median	xx.x	xx.x	xx.x
Min, Max	xx, xx	xx, xx	xx, xx
Total GDNF exposure[a] (mg)			
n	xx	Xx	Xx
Mean (SD)	xx.x (xx.xx)	xx.x (xx.xx)	xx.x (xx.xx)
Median	xx.x	xx.x	xx.x
Min, Max	xx, xx	xx, xx	xx, xx
Pilot Extension			
Number of infusions of study medication			
n	xx	Xx	Xx
Mean (SD)	xx.x (xx.xx)	xx.x (xx.xx)	xx.x (xx.xx)
Median	xx.x	xx.x	xx.x
Min, Max	xx, xx	xx, xx	xx, xx
Total GDNF exposure[a] (mg)			
n	xx	Xx	Xx
Mean (SD)	xx.x (xx.xx)	xx.x (xx.xx)	xx.x (xx.xx)
Median	xx.x	xx.x	xx.x
Min, Max	xx, xx	xx, xx	xx, xx

[a] Total exposure in mg assumes the entire infusion was completed at each administration.
Source: Listing xx.x.xx, Dataset: [NAME], Program: xxxxxx.sas, Output: T_16_4_1_1_XXXXX.rtf, Generated on: DDMONYYYY HH:MM
Page x of y

Table 16.4.1.1 Exposure to Study Medication - Safety Overall Population (cont.)

Extension Part<br>Variable	GDNF/GDNF<br>(N = XXX)	Placebo/GDNF<br>(N = XXX)	Total<br>(N = XXX)
Supplemental Extension			
Number of infusions of study medication			
n	xx	Xx	Xx
Mean (SD)	xx.x (xx.xx)	xx.x (xx.xx)	xx.x (xx.xx)
Median	xx.x	xx.x	xx.x
Min, Max	xx, xx	xx, xx	xx, xx
Total GDNF exposure[a] (mg)			
n	xx	Xx	Xx
Mean (SD)	xx.x (xx.xx)	xx.x (xx.xx)	xx.x (xx.xx)
Median	xx.x	xx.x	xx.x
Min, Max	xx, xx	xx, xx	xx, xx
Overall			
Number of infusions of study medication			
n	xx	Xx	Xx
Mean (SD)	xx.x (xx.xx)	xx.x (xx.xx)	xx.x (xx.xx)
Median	xx.x	xx.x	xx.x
Min, Max	xx, xx	xx, xx	xx, xx
Total GDNF exposure[a] (mg)			
n	xx	Xx	Xx
Mean (SD)	xx.x (xx.xx)	xx.x (xx.xx)	xx.x (xx.xx)
Median	xx.x	xx.x	xx.x
Min, Max	xx, xx	xx, xx	xx, xx

[a] Total exposure in mg assumes the entire infusion was completed at each administration.
Source: Listing xx.x.xx, Dataset: [NAME], Program: xxxxxx.sas, Output: T_16_4_1_1_XXXXX.rtf, Generated on: DDMONYYYY HH:MM
Page x of y

Table 16.4.1.2 Study Medication Infusion Details by Visit - Safety Overall Population

Visit<br>Variable	GDNF/GDNF<br>(N = XXX)	Placebo/GDNF<br>(N = XXX)	Total<br>(N = XXX)
Week e0			
Duration of infusion[a] (minutes)			
n	xx	Xx	Xx
Mean (SD)	xx.x (xx.xx)	xx.x (xx.xx)	xx.x (xx.xx)
Median	xx.x	xx.x	xx.x
Min, Max	xx, xx	xx, xx	xx, xx
Any infusion interruption/early termination [n (%)]			
No	xx (xx.x)	xx (xx.x)	xx (xx.x)
Yes	xx (xx.x)	xx (xx.x)	xx (xx.x)
Week 44/ e4			
…			

[a] Duration of infusion of study medication in minutes is calculated as (infusion end time – infusion start time + 1). Interruptions are not subtracted.
Source: Listing xx.x.xx, Dataset: [NAME], Program: xxxxxx.sas, Output: T_16_4_1_2_XXXXX.rtf, Generated on: DDMONYYYY HH:MM
Page x of y

Programming Note: Continue for all infusion visits, including the Pilot Extension and Supplemental Extension.

16.4.2 ADVERSE EVENT TABLES

Table 16.4.2.1.1 Overall Summary of Adverse Events - Safety Overall Population

Adverse Event Category	GDNF/GDNF (N = XXX) n (%)	Placebo/GDNF (N = XXX) n (%)	Total (N = XXX) n (%)
Any TEAE	xxx (xx.x)	xxx (xx.x)	xxx (xx.x)
Any severe TEAE	xxx (xx.x)	xxx (xx.x)	xxx (xx.x)
Any serious TEAE	xxx (xx.x)	xxx (xx.x)	xxx (xx.x)
Any TEAE leading to permanent discontinuation of study medication	xxx (xx.x)	xxx (xx.x)	xxx (xx.x)
Any study medication-related TEAE	xxx (xx.x)	xxx (xx.x)	xxx (xx.x)
Any serious study medication-related TEAE	xxx (xx.x)	xxx (xx.x)	xxx (xx.x)
Any device-related TEAE	xxx (xx.x)	xxx (xx.x)	xxx (xx.x)
Any serious device-related TEAE	xxx (xx.x)	xxx (xx.x)	xxx (xx.x)

Note: For each category, subjects are included only once, even if they experienced multiple events in that category. All AEs reported during the study period are considered TEAEs. TEAEs that are present or ongoing at the beginning of Study 2797 (date of consent or date of last visit in Study 2553, if consent was obtained earlier) are considered pre-existing, if the event term, severity, and date and time of onset in Study 2797 are identical to the corresponding information given for the TEAE in Study 2553. If any of these conditions is not met, the TEAE is considered new or worsening. Only new or worsening TEAEs are summarized.
Source: Listing xx.x.xx, Dataset: [NAME], Program: xxxxxx.sas, Output: xxxx.rtf, Generated on: DDMONYYYY HH:MM
Page 1 of 1

Table 16.4.2.1.2 Overall Summary of Adverse Events During the Initial Extension - Safety Overall Population
Repeat Table 16.4.2.1.1.

Table 16.4.2.1.3 Overall Summary of Adverse Events During the Pilot and Supplemental Extensions - Safety Overall Population
Repeat Table 16.4.2.1.1.

Table 16.4.2.2 Treatment-Emergent Adverse Events by System Organ Class and Preferred Term - Safety Overall Population

**System Organ Class**<br>**Preferred Term**	**GDNF/GDNF (N = XXX) n (%)**	**Placebo/GDNF (N = XXX) n (%)**	**Total (N = XXX) n (%)**
Subjects with at least one TEAE	xx (xx.x)	xx (xx.x)	xx (xx.x)
[System Organ Class 1]	xx (xx.x)	xx (xx.x)	xx (xx.x)
[Preferred Term 1]	xx (xx.x)	xx (xx.x)	xx (xx.x)
[Preferred Term 2]	xx (xx.x)	xx (xx.x)	xx (xx.x)
[System Organ Class 2]	xx (xx.x)	xx (xx.x)	xx (xx.x)
[Preferred Term 1]	xx (xx.x)	xx (xx.x)	xx (xx.x)
[Preferred Term 2]	xx (xx.x)	xx (xx.x)	xx (xx.x)
…			

Note: Adverse events are coded using MedDRA version 19.0. All AEs reported during the study period are considered TEAEs. TEAEs that are present or ongoing at the beginning of Study 2797 (date of consent or date of last visit in Study 2553, if consent was obtained earlier) are considered pre-existing, if the event term, severity, and date and time of onset in Study 2797 are identical to the corresponding information given for the TEAE in Study 2553. If any of these conditions is not met, the TEAE is considered new or worsening. Only new or worsening TEAEs are summarized. For each system organ class and preferred term, subjects are included only once, even if they experienced multiple events in that system organ class or preferred term.
Source: Listing xx.x.xx, Dataset: [NAME], Program: xxxxxx.sas, Output: T_16_4_2_2_XXXXX.rtf, Generated on: DDMONYYYY HH:MM
Page x of y

Programming Note: Sort alphabetically by SOC and then alphabetically for PT.

Table 16.4.2.3.1 Treatment-Emergent Adverse Events Experienced by at Least 3 Subjects Overall by Preferred Term - Safety Overall Population

	GDNF/GDNF (N = XXX)		Placebo/GDNF (N = XXX)		Total (N=XXX)	
**Preferred Term**	**n Subjects (%)**	**n Events**	**n Subjects (%)**	**n Events**	**n Subjects (%)**	**n Events**
[Preferred Term 1]	xx (xx.x)	xxx	xx (xx.x)	Xxx	xx (xx.x)	Xxx
[Preferred Term 2]	xx (xx.x)	xxx	xx (xx.x)	Xxx	xx (xx.x)	Xxx
[Preferred Term 3]	xx (xx.x)	xxx	xx (xx.x)	Xxx	xx (xx.x)	Xxx
[Preferred Term 4]	xx (xx.x)	xxx	xx (xx.x)	Xxx	xx (xx.x)	Xxx
[Preferred Term 5]	xx (xx.x)	xxx	xx (xx.x)	Xxx	xx (xx.x)	Xxx
[Preferred Term 6]	xx (xx.x)	xxx	xx (xx.x)	Xxx	xx (xx.x)	Xxx
[Preferred Term 7]	xx (xx.x)	xxx	xx (xx.x)	Xxx	xx (xx.x)	Xxx
[Preferred Term 8]	xx (xx.x)	xxx	xx (xx.x)	Xxx	xx (xx.x)	Xxx
…						

Note: Adverse events are coded using MedDRA version 19.0. All AEs reported during the study period are considered TEAEs. TEAEs that are present or ongoing at the beginning of Study 2797 (date of consent or date of last visit in Study 2553, if consent was obtained earlier) are considered pre-existing, if the event term, severity, and date and time of onset in Study 2797 are identical to the corresponding information given for the TEAE in Study 2553. If any of these conditions is not met, the TEAE is considered new or worsening. Only new or worsening TEAEs are summarized. For each preferred term, subjects are included only once, even if they experienced multiple events in that preferred term.
Source: Listing xx.x.xx, Dataset: [NAME], Program: xxxxxx.sas, Output: T_16_4_2_3_XXXXX.rtf, Generated on: DDMONYYYY HH:MM
Page 1 of 1

Programming Note: Sort by descending frequency of number of subjects in the total column.

Table 16.4.2.3.2 Treatment-Emergent Adverse Events Experienced by at Least 3 Subjects Overall During the Initial Extension by Preferred Term - Safety Overall Population

Repeat Table 16.4.2.3.1.

Table 16.4.2.3.3 Treatment-Emergent Adverse Events Experienced by at Least 3 Subjects Overall During the Pilot and Supplemental Extensions by Preferred Term - Safety Overall Population

Repeat Table 16.4.2.3.1.

Table 16.4.2.4 Treatment-Emergent Adverse Events by System Organ Class, Preferred Term, and Maximum Severity - Safety Overall Population

	GDNF/GDNF (N = XXX)				Placebo/GDNF (N = XXX)			
**System Organ Class<br>Preferred Term**	**Mild<br>n (%)**	**Moderate<br>n (%)**	**Severe<br>n (%)**	**Total<br>n (%)**	**Mild<br>n (%)**	**Moderate<br>n (%)**	**Severe<br>n (%)**	**Total<br>n (%)**
Subjects with at least one TEAE	xxx (xx.x)	xxx (xx.x)	xxx (xx.x)	xxx (xx.x)	xxx (xx.x)	xxx (xx.x)	xxx (xx.x)	xxx (xx.x)
[System Organ Class 1]	xxx (xx.x)	xxx (xx.x)	xxx (xx.x)	xxx (xx.x)	xxx (xx.x)	xxx (xx.x)	xxx (xx.x)	xxx (xx.x)
[Preferred Term 1]]	xxx (xx.x)	xxx (xx.x)	xxx (xx.x)	xxx (xx.x)	xxx (xx.x)	xxx (xx.x)	xxx (xx.x)	xxx (xx.x)
[Preferred Term 2]	xxx (xx.x)	xxx (xx.x)	xxx (xx.x)	xxx (xx.x)	xxx (xx.x)	xxx (xx.x)	xxx (xx.x)	xxx (xx.x)
[Preferred Term 3]	xxx (xx.x)	xxx (xx.x)	xxx (xx.x)	xxx (xx.x)	xxx (xx.x)	xxx (xx.x)	xxx (xx.x)	xxx (xx.x)
[Preferred Term 4]	xxx (xx.x)	xxx (xx.x)	xxx (xx.x)	xxx (xx.x)	xxx (xx.x)	xxx (xx.x)	xxx (xx.x)	xxx (xx.x)
…								
[System Organ Class 2]	xxx (xx.x)	xxx (xx.x)	xxx (xx.x)	xxx (xx.x)	xxx (xx.x)	xxx (xx.x)	xxx (xx.x)	xxx (xx.x)
[Preferred Term 1]	xxx (xx.x)	xxx (xx.x)	xxx (xx.x)	xxx (xx.x)	xxx (xx.x)	xxx (xx.x)	xxx (xx.x)	xxx (xx.x)
[Preferred Term 2]	xxx (xx.x)	xxx (xx.x)	xxx (xx.x)	xxx (xx.x)	xxx (xx.x)	xxx (xx.x)	xxx (xx.x)	xxx (xx.x)
…								
[System Organ Class 3]	xxx (xx.x)	xxx (xx.x)	xxx (xx.x)	xxx (xx.x)	xxx (xx.x)	xxx (xx.x)	xxx (xx.x)	xxx (xx.x)
…								

Note: Adverse events are coded using MedDRA version 19.0. All AEs reported during the study period are considered TEAEs. TEAEs that are present or ongoing at the beginning of Study 2797 (date of consent or date of last visit in Study 2553, if consent was obtained earlier) are considered pre-existing, if the event term, severity, and date and time of onset in Study 2797 are identical to the corresponding information given for the TEAE in Study 2553. If any of these conditions is not met, the TEAE is considered new or worsening. Only new or worsening TEAEs are summarized. For each system organ class and preferred term, subjects are included only once, in the maximum severity, for that TEAE. TEAEs without severity information are counted in the respective Total column for severity only.

Source: Listing xx.x.xx, Dataset: [NAME], Program: xxxxxx.sas, Output: xxxx.rtf, Generated on: DDMONYYYY HH:MM

Page x of y

Table 16.4.2.4 Treatment-Emergent Adverse Events by System Organ Class, Preferred Term, and Maximum Severity - Safety Overall Population (cont.)

	Total (N = XXX)			
**System Organ Class<br>Preferred Term**	**Mild<br>n (%)**	**Moderate<br>n (%)**	**Severe<br>n (%)**	**Total<br>n (%)**
Subjects with at least one TEAE	xxx (xx.x)	xxx (xx.x)	xxx (xx.x)	xxx (xx.x)
[System Organ Class 1]	xxx (xx.x)	xxx (xx.x)	xxx (xx.x)	xxx (xx.x)
[Preferred Term 1]]	xxx (xx.x)	xxx (xx.x)	xxx (xx.x)	xxx (xx.x)
[Preferred Term 2]	xxx (xx.x)	xxx (xx.x)	xxx (xx.x)	xxx (xx.x)
[Preferred Term 3]	xxx (xx.x)	xxx (xx.x)	xxx (xx.x)	xxx (xx.x)
[Preferred Term 4]	xxx (xx.x)	xxx (xx.x)	xxx (xx.x)	xxx (xx.x)
…				
[System Organ Class 2]	xxx (xx.x)	xxx (xx.x)	xxx (xx.x)	xxx (xx.x)
[Preferred Term 1]	xxx (xx.x)	xxx (xx.x)	xxx (xx.x)	xxx (xx.x)
[Preferred Term 2]	xxx (xx.x)	xxx (xx.x)	xxx (xx.x)	xxx (xx.x)
…				
[System Organ Class 3]	xxx (xx.x)	xxx (xx.x)	xxx (xx.x)	xxx (xx.x)
…				

Note: Adverse events are coded using MedDRA version 19.0. All AEs reported during the study period are considered TEAEs. TEAEs that are present or ongoing at the beginning of Study 2797 (date of consent or date of last visit in Study 2553, if consent was obtained earlier) are considered pre-existing, if the event term, severity, and date and time of onset in Study 2797 are identical to the corresponding information given for the TEAE in Study 2553. If any of these conditions is not met, the TEAE is considered new or worsening. Only new or worsening TEAEs are summarized. For each system organ class and preferred term, subjects are included only once, in the maximum severity, for that TEAE. TEAEs without severity information are counted in the respective Total column for severity only.
Source: Listing xx.x.xx, Dataset: [NAME], Program: xxxxxx.sas, Output: xxxx.rtf, Generated on: DDMONYYYY HH:MM
Page x of y

Programming Note: Sort alphabetically by SOC and then alphabetically for PT.

Table 16.4.2.5.1 Serious Treatment-Emergent Adverse Events by System Organ Class and Preferred Term - Safety Overall Population

Repeat SOC & PT Table 16.4.2.2. First row label is "Subjects with at least one serious TEAE."

Table 16.4.2.5.2 Serious Treatment-Emergent Adverse Events by System Organ Class and Preferred Term During the Initial Extension - Safety Overall Population

Repeat SOC & PT Table 16.4.2.2. First row label is "Subjects with at least one serious TEAE during the Initial Extension."

Table 16.4.2.5.3 Serious Treatment-Emergent Adverse Events by System Organ Class and Preferred Term During the Pilot and Supplemental Extensions - Safety Overall Population

Repeat SOC & PT Table 16.4.2.2. First row label is "Subjects with at least one serious TEAE during the Pilot and Supplemental Extensions."

Table 16.4.2.6 Study Medication-Related Treatment-Emergent Adverse Events by System Organ Class and Preferred Term - Safety Overall Population

Programming Note: Repeat SOC & PT Table 16.4.2.2. First row label is "Subjects with at least one study medication-related TEAE."

Table 16.4.2.7 Serious Study Medication-Related Treatment-Emergent Adverse Events by System Organ Class and Preferred Term - Safety Overall Population

Programming Note: Repeat Table SOC & PT 16.4.2.2. First row label is "Subjects with at least one serious study medication-related TEAE."

Table 16.4.2.8 Device-Related Treatment-Emergent Adverse Events by System Organ Class and Preferred Term - Safety Overall Population

Programming Note: Repeat Table SOC & PT 16.4.2.2. First row label is "Subjects with at least one device-related TEAE."

Table 16.4.2.9 Serious Device-Related Treatment-Emergent Adverse Events by System Organ Class and Preferred Term - Safety Overall Population

Programming Note: Repeat Table SOC & PT 16.4.2.2. First row label is "Subjects with at least one serious device-related TEAE."

Table 16.4.2.10 Treatment-Emergent Adverse Events of Special Interest by Preferred Term - Safety Overall Population

AESI Category<br>Preferred Term	GDNF/GDNF<br>(N = XXX)<br>n (%)	Placebo/GDNF<br>(N = XXX)<br>n (%)	Total<br>(N = XXX)<br>n ((%)
Subjects with at least one treatment-emergent AESI	xx (xx.x)	xx (xx.x)	xx (xx.x)
Dyskinesias	xx (xx.x)	xx (xx.x)	xx (xx.x)
[Preferred Term 1]	xx (xx.x)	xx (xx.x)	xx (xx.x)
[Preferred Term 2]	xx (xx.x)	xx (xx.x)	xx (xx.x)
Falls	xx (xx.x)	xx (xx.x)	xx (xx.x)
[Preferred Term 1]	xx (xx.x)	xx (xx.x)	xx (xx.x)
[Preferred Term 2]	xx (xx.x)	xx (xx.x)	xx (xx.x)
Adverse changes in mood	xx (xx.x)	xx (xx.x)	xx (xx.x)
[Preferred Term 1]	xx (xx.x)	xx (xx.x)	xx (xx.x)
[Preferred Term 2]	xx (xx.x)	xx (xx.x)	xx (xx.x)
Impulsivity	xx (xx.x)	xx (xx.x)	xx (xx.x)
[Preferred Term 1]	xx (xx.x)	xx (xx.x)	xx (xx.x)
[Preferred Term 2]	xx (xx.x)	xx (xx.x)	xx (xx.x)
…			

Note: Adverse events are coded using MedDRA version 19.0. All AEs reported during the study period are considered TEAEs. TEAEs that are present or ongoing at the beginning of Study 2797 (date of consent or date of last visit in Study 2553, if consent was obtained earlier) are considered pre-existing, if the event term, severity, and date and time of onset in Study 2797 are identical to the corresponding information given for the TEAE in Study 2553. If any of these conditions is not met, the TEAE is considered new or worsening. Only new or worsening TEAEs are summarized. For each category and preferred term, subjects are included only once, even if they experienced multiple events in that category or preferred term.
Source: Listing xx.x.xx, Dataset: [NAME], Program: xxxxxx.sas, Output: T_16_4_2_10_XXXXX.rtf, Generated on: DDMONYYYY HH:MM
Page 1 of 1

16.4.3 LABORATORY TABLES

Table 16.4.3.1 Clinically Significant Postbaseline Hematology Results - Safety Overall Population

Parameter	GDNF/GDNF (N = XXX) n (%)	Placebo/GDNF (N = XXX) n (%)
[Lab parameter 1 – high]	xxx (xx.x)	xxx (xx.x)
[Lab parameter 2]	xxx (xx.x)	xxx (xx.x)
[Lab parameter 3]	xxx (xx.x)	xxx (xx.x)
[Lab parameter 4]	xxx (xx.x)	xxx (xx.x)
[Lab parameter 5]	xxx (xx.x)	xxx (xx.x)
[Lab parameter 6]	xxx (xx.x)	xxx (xx.x)
[Lab parameter 7]	xxx (xx.x)	xxx (xx.x)
[Lab parameter 8]	xxx (xx.x)	xxx (xx.x)
…		

Note: Results were rated by the investigator as clinically significant on the CRF based on medical judgment, not using any pre-specified numerical criteria. For each parameter, subjects are included only once, even if they experienced more than one clinically significant result.
Source: Listing xx.x.xx, Dataset: [NAME], Program: xxxxxx.sas, Output: xxxx.rtf, Generated on: DDMONYYYY HH:MM
Page x of y

Programming Note: See text for parameters. Sort by order of parameters shown in SAP Table 5.

Table 16.4.3.2 Clinically Significant Postbaseline Serum Chemistry Results - Safety Overall Population

Programming Note: Repeat clinically significant hematology Table 16.4.3.1 for serum chemistry parameters.

16.4.4 ANTI-GDNF SERUM ANTIBODY TABLES

Table 16.4.4.1 Anti-GDNF Binding Serum Antibodies by Visit – Safety Overall Population

Visit<br>Variable	GDNF/GDNF<br>(N = XXX)<br>n (%)	Placebo/GDNF<br>(N = XXX)<br>n (%)
All visits[a]		
All Negative	xxx (xx.x)	xxx (xx.x)
1 Positive	xxx (xx.x)	xxx (xx.x)
> 1 Positive	xxx (xx.x)	xxx (xx.x)
1 Missing or Not Done	xxx (xx.x)	xxx (xx.x)
2 Missing or Not Done	xxx (xx.x)	xxx (xx.x)
>2 Missing or Not Done	xxx (xx.x)	xxx (xx.x)
Screening (Baseline)		
Positive	xxx (xx.x)	xxx (xx.x)
Negative	xxx (xx.x)	xxx (xx.x)
Not done	xxx (xx.x)	xxx (xx.x)
Missing	xxx (xx.x)	xxx (xx.x)
Week 40/e0		
Positive	xxx (xx.x)	xxx (xx.x)
Negative	xxx (xx.x)	xxx (xx.x)
Not done	xxx (xx.x)	xxx (xx.x)
Missing	xxx (xx.x)	xxx (xx.x)
Week 44/e4		
Positive	xxx (xx.x)	xxx (xx.x)
Negative	xxx (xx.x)	xxx (xx.x)
Not done	xxx (xx.x)	xxx (xx.x)
Missing	xxx (xx.x)	xxx (xx.x)
…		

[a] "All visits" includes all postbaseline visits from Study 2553 and Study 2797. "All negative" includes all subjects without a positive result even if some results are missing or not done.

Source: Listing xx.x.xx, Dataset: [NAME], Program: xxxxxx.sas, Output: xxxx.rtf, Generated on: DDMONYYYY HH:MM
Page x of y

Programming Note: Visits are screening and Weeks 40/e0, 44/e4, 56/e16, 68/e28, 80/e40, e2-24, e2-48, e2-72, and Last Study Visit in Supplemental Extension.

Table 16.4.4.2 Anti-GDNF Neutralizing Serum Antibodies by Visit - Safety Overall Population

Programming Note: Repeat table for neutralizing antibodies.

16.4.5 Plasma GDNF Concentration Tables

Table 16.4.5 Plasma GDNF Concentrations by Visit - Safety Overall Population

Plasma GDNF Concentration (unit)	GDNF/GDNF (N = XXX)		Placebo/GDNF (N = XXX)	
Visit<br>Statistic	Value	Change From Baseline	Value	Change From Baseline
Screening (Baseline)				
n	xx		xx	
Mean (SD)	xx.xx (xx.xxx)		xx.xx (xx.xxx)	
Median	xx.x		xx.x	
Min, Max	xx.x, xx.x		xx.x, xx.x	
Not Done	xx		xx	
Missing	xx		xx	
Week 40/e0				
n	xx	xx	xx	xx
Mean (SD)	xx.xx (xx.xxx)	xx.xx (xx.xxx)	xx.xx (xx.xxx)	xx.xx (xx.xxx)
Median	xx.x	xx.x	xx.x	xx.x
Min, Max	xx.x, xx.x	xx.x, xx.x	xx.x, xx.x	xx.x, xx.x
Not Done	xx	xx	xx	xx
Missing	xx	xx	xx	xx
Week 44/e4				
n	xx	xx	xx	Xx
Mean (SD)	xx.xx (xx.xxx)	xx.xx (xx.xxx)	xx.xx (xx.xxx)	xx.xx (xx.xxx)
Median	xx.x	xx.x	xx.x	xx.x
Min, Max	xx.x, xx.x	xx.x, xx.x	xx.x, xx.x	xx.x, xx.x
Not Done	xx	xx	xx	xx
Missing	xx	xx	xx	xx
…				

Note: Results of <LOD and <LLOQ are not included in the summary statistics

Source: Listing xx.x.xx, Dataset: [NAME], Program: xxxxxx.sas, Output: xxxx.rtf, Generated on: DDMONYYYY HH:MM
Page x of y

Programming Note: Visits are screening and Weeks 40/e0, 44/e4, 56/e16, 68/e28, 80/e40, e2-24, e2-48, e2-72 and Last Study Visit in Supplemental Extension.

16.4.6 Vital Sign Table

Table 16.4.6 Clinically Relevant Postbaseline Abnormalities in Vital Sign Results in the Initial Extension - Safety Overall Population

Parameter and Criterion<br>..Visit<br>Time Point	GDNF/GDNF<br>(N = XXX)<br>n (%)	Placebo/GDNF<br>(N = XXX)<br>n (%)
[VS parameter and criterion 1] at any visit in Initial Extension	xxx (xx.x)	xxx (xx.x)
[Visit 1]	xxx (xx.x)	xxx (xx.x)
[Time point 1]	xxx (xx.x)	xxx (xx.x)
[Time point 2]	xxx (xx.x)	xxx (xx.x)
…		

Note: bpm = beats per minute. For each parameter and criterion, visit, and time point, subjects are included only once, even if they experienced more than one clinically relevant abnormality. For the comparisons of values during or after infusion with pre-infusion values, the baseline is the pre-infusion value.
Source: Listing xx.x.xx, Dataset: [NAME], Program: xxxxxx.sas, Output: xxxx.rtf, Generated on: DDMONYYYY HH:MM
Page x of y

Programming Note: Only include those VS parameters and criteria that have at least one clinically relevant abnormality present in the data. This table should include test infusion visits (interim visits after catheter repositioning and Week 80/e40 visits) as well as study medication infusion visits in the Initial Extension.

16.4.7 Electrocardiogram Tables

Table 16.4.7.1 Electrocardiogram Results by Visit – Safety Overall Population

Parameter (unit)<br>Visit<br>Statistic	GDNF/GDNF<br>(N = XXX)		Placebo/GDNF<br>(N = XXX)	
	Value	Change From Baseline	Value	Change From Baseline
Heart rate (beats/minute)				
Screening (Baseline)				
n	xx		xx	
Mean (SD)	xx.xx (xx.xxx)		xx.xx (xx.xxx)	
Median	xx.x		xx.x	
Min, Max	xx.x, xx.x		xx.x, xx.x	
Week 40/e0				
n	xx	xx	xx	xx
Mean (SD)	xx.xx (xx.xxx)	xx.xx (xx.xxx)	xx.xx (xx.xxx)	xx.xx (xx.xxx)
Median	xx.x	xx.x	xx.x	xx.x
Min, Max	xx.x, xx.x	xx.x, xx.x	xx.x, xx.x	xx.x, xx.x
Week 80/e40				
n	xx	xx	xx	Xx
Mean (SD)	xx.xx (xx.xxx)	xx.xx (xx.xxx)	xx.xx (xx.xxx)	xx.xx (xx.xxx)
Median	xx.x	xx.x	xx.x	xx.x
Min, Max	xx.x, xx.x	xx.x, xx.x	xx.x, xx.x	xx.x, xx.x

Note: Hodges QT correction formula was used.
Source: Listing xx.x.xx, Dataset: [NAME], Program: xxxxxx.sas, Output: xxxx.rtf, Generated on: DDMONYYYY HH:MM
Page x of y

Programming Note: Visits are screening, Week 40/e0 and Week 80/e40. Parameters are heart rate (beats/min), PR interval (ms), QRS interval (ms), QT interval (ms), and QTc interval (ms).

Table 16.4.7.2 Summary of Electrocardiogram Results at Week 80/e40 - Safety Overall Population

ECG Evaluation	GDNF/GDNF (N = XXX) n (%)	Placebo/GDNF (N = XXX) n (%)
Overall impression		
Normal Week 80/e40 result	xxx (xx.x)	xxx (xx.x)
Any abnormal Week 80/e40 result	xxx (xx.x)	xxx (xx.x)
Any clinically significant abnormal Week 80/e40 result	xxx (xx.x)	xxx (xx.x)
Any clinically relevant abnormal Week 80/e40 QTc interval result based on criteria below	xxx (xx.x)	xxx (xx.x)
[Criterion 1]	xxx (xx.x)	xxx (xx.x)
[Criterion 2]	xxx (xx.x)	xxx (xx.x)
…		

Note: Overall ECG impression was rated by the investigator as abnormal and clinically significant on the CRF based on medical judgment. Hodges QT correction formula was used.

Source: Listing xx.x.xx, Dataset: [NAME], Program: xxxxxx.sas, Output: xxxx.rtf, Generated on: DDMONYYYY HH:MM
Page x of y

Programming Note: Present all QTc parameters even if no subjects had an abnormal result.

16.4.8 Glasgow Coma Scale Table

Table 16.4.8 Glasgow Coma Scale Score = 15 or < 15 During or After Infusion by Visit and Time Point in the Initial Extension– Safety Overall Population

Visit and Time Point<br>Glasgow Coma Scale Score	GDNF/GDNF<br>(N = XXX)<br>n (%)	Placebo/GDNF<br>(N = XXX)<br>n (%)
Week e0 at any time during or after infusion		
All scores = 15	xx (xx.x)	xx (xx.x)
Any score < 15	xx (xx.x)	xx (xx.x)
No score < 15 but at least one score is missing	xx (xx.x)	xx (xx.x)
Week 44/e4 at any time during or after infusion		
....		
Week 80/e40 at any time during or after infusion		
…		

Note: Glasgow Coma Scale items include visual response, verbal ability, and motor skills. Each item has 4-6 possible responses. The best possible total score is 15.
Source: Listing xx.x.xx, Dataset: [NAME], Program: xxxxxx.sas, Output: xxxx.rtf, Generated on: DDMONYYYY HH:MM
Page x of y

Programming Note: Present for all test infusion visits and study medication infusion visits in the Initial Extension. Add information in Table 8 as an endnote.

16.4.9 Other Safety Tables

Table 16.4.9.1 QUIP by Visit - Safety Overall Population

	GDNF/GDNF (N = XXX)			Placebo/GDNF (N = XXX)		
	Postbaseline Value			Postbaseline Value		
**Assessor**<br>**Parameter**<br>**Visit**<br>**Baseline Value**	**Positive n (%)**	**Negative n (%)**	**Missing n (%)**	**Positive n (%)**	**Negative n (%)**	**Missing n (%)**
Subject assessment						
Issue with too much gambling						
Week 48/e8						
Positive	xxx (xx.x)	xxx (xx.x)	xxx (xx.x)	xxx (xx.x)	xxx (xx.x)	xxx (xx.x)
Negative	xxx (xx.x)	xxx (xx.x)	xxx (xx.x)	xxx (xx.x)	xxx (xx.x)	xxx (xx.x)
Missing	xxx (xx.x)	xxx (xx.x)	xxx (xx.x)	xxx (xx.x)	xxx (xx.x)	xxx (xx.x)
Week 56/e16						
Positive	xxx (xx.x)	xxx (xx.x)	xxx (xx.x)	xxx (xx.x)	xxx (xx.x)	xxx (xx.x)
Negative	xxx (xx.x)	xxx (xx.x)	xxx (xx.x)	xxx (xx.x)	xxx (xx.x)	xxx (xx.x)
Missing	xxx (xx.x)	xxx (xx.x)	xxx (xx.x)	xxx (xx.x)	xxx (xx.x)	xxx (xx.x)
…						

Note: The QUIP is a self-administered or informant-completed scale that includes 13 questions covering symptoms related to the 4 commonest impulse control disorders in PD, other behaviors, and problematic use of medication. The number of “Yes” responses is compared to the number required for a positive result. Missing individual responses are imputed using LOCF. Each impulse control disorder, other behavior, and problematic use of medication is rated as a positive result (ie, present) or a negative result (ie, not present). Percentages are based on the total number of observations for that visit.
Source: Listing xx.x.xx, Dataset: [NAME], Program: xxxxxx.sas, Output: xxxx.rtf, Generated on: DDMONYYYY HH:MM
Page x of y

Programming Note: Visits are Week 40/e0 (baseline), Weeks 48/e8, 56/e16, 64/e24, 72/e32, and 80/e40; e2 16, e2-32, e2-48, e2-64, and e2-80; e3-16, e3-32, and the last study visit in the Supplemental Extension. Assessor includes subject assessment and informant assessment. Parameters (30) include: Issue with too much gambling, issue with too much sex, issue with too much buying, issue with too much eating,
Think too much about gambling, think too much about sex, think too much about buying, think too much about eating,
Excessive or distressing urge for gambling, excessive or distressing urge for sex, excessive or distressing urge for buying, excessive or distressing urge for eating,
Difficulty controlling gambling, difficulty controlling sex, difficulty controlling buying, difficulty controlling eating,
Engage in activities specifically to continue gambling, engage in activities specifically to continue sex, engage in activities specifically to continue buying, engage in activities specifically to continue eating, Spend too much time on specific tasks hobbies or other organized activities, spend too much time repeating certain simple motor activities, spend too much time walking or driving with no intended goal or specific purpose, difficulty controlling the amount of time spent on these activities, activities interfere with daily functioning or cause relationship or work difficulties,

Consistently take too much PD medications, increased PD medications without medical advice for psychological reasons, increased PD medications without medical advice because only feel fully "on" when dyskinetic, difficulty controlling use of PD medications, hoard or hide PD medications to increase overall dosage.

Table 16.4.9.2 MoCA by Visit - Safety Overall Population

Programming Note: Repeat Table 16.4.5 (Electrocardiogram results by visit specified) for MoCA total score. Visits are screening, pre-test infusion (baseline), Weeks 40/e0, 56/e16, and 80/e40; e2-80; and the last study visit in the Supplemental Extension. Add footnote: "Note: The MoCA is a rater-administered cognitive screening tool with 8 components. The total score ranges from 0 to 30, with lower scores representing poorer cognitive function. A total score of 26 or above is considered normal. Missing individual scores are imputed using LOCF if necessary."

Table 16.4.9.3 MDRS by Visit - Safety Overall Population

Programming Note: Repeat Table 16.4.5 (Electocardiogram results by visit specified) for MDRS total score. Visits are screening (baseline), Weeks 40/e0, 56/e16 and 80/e40; e2-80; and the last study visit in the Supplemental Extension. Add footnote: "Note: The MDRS is a rater-administered global scale of cognition including 5 subscales. The total score ranges from 0 to 144, with higher scores representing better cognitive function. A total score lower than 123 is associated with some degree of dementia in PD. The AEMSS total score ranges from 0 to 20, with higher scores representing better cognitive function. Individual missing items are imputed using the average of non-missing scores in each subscale if necessary."

Table 16.4.9.4 Stroop Test by Visit, Safety Overall Population

Programming Note: Repeat Table 16.4.5 (Electocardiogram results by visit specified) for Stroop test parameters. The parameters are the 4 conditions: color naming, word reading, inhibition, and inhibition/switching. Visits are screening, Week 40/e0, and Week 80/e40. Add footnote: "Note: The Stroop test is a global scale of reaction time including 4 conditions. Total time to complete the test in each condition can range from 0 to 999 seconds, with lower time representing better reaction time."

Table 16.4.9.5 FrSBe by Visit - Safety Overall Population

Programming Note: Repeat Table 16.4.5 (Electocardiogram results by visit specified) for FrSBe parameters. The parameters are the 3 subscales: apathy, disinhibition, and executive dysfunction. Total scores are not calculated. Visits are screening "before", screening "after" (baseline), Week 40/e0 "after", and Week 80/e40 "after." Add footnote: "Note: The FrSBe is a scale that assesses behavior related to frontal systems damage including 3 subscales. Higher subscale scores indicate greater pathology. Individual missing items are imputed using the average of non-missing scores in each subscale."

Table 16.4.9.6 Deary-Liewald Four-Choice Reaction Time by Visit - Safety Overall Population

Programming Note: Repeat Table 16.4.5 (Electocardiogram results by visit specified) for RT parameters. Visits are screening (baseline), Week 40/e0, and Weeks 56/e16 and 80/e40. Add footnote: "Note: The Deary-Liewald RT is a computerized measure of simple and four-choice reaction time. The parameter is the mean reaction time, variance and SD for correct responses for four-choice reaction time. A shorter reaction time is better."

Table 16.4.9.7 Verbal Fluency Assessment by Visit - Safety Overall Population

Programming Note: Repeat Table 16.4.5 (Electocardiogram results by visit specified) for *for verbal fluency parameters. Parameters are phonemic and semantic verbal fluency. Visits are screening (baseline), Week 40/e0, and Week 80/e40. Add footnote: "Note: The verbal fluency assessment measures verbal functioning in 2 categories. Scores represent number of correct words in one minute and range from 0 to 200. Higher scores represent better verbal functioning."*

Table 16.4.9.8 BDI by Visit - Safety Overall Population

Programming Note: Repeat Table 16.4.5 (Electocardiogram results by visit specified) for *for BDI parameters. The parameter is the total score. Visits are screening (baseline), Week 40/e0, and Week 80/e40; and the last study visit in both the Pilot Extension and the Supplemental Extension. Add footnote: "Note: The BDI is a self-administered test which consists of 21 questions that measure the severity of depression. Scores range from 0 to 63, with higher scores representing worse depression. Individual missing items are imputed using the average of non-missing scores in each subscale."*

Table 16.4.9.9 UPSIT by Visit - Safety Overall Population

Programming Note: Repeat Table 16.4.5 (Electocardiogram results by visit specified) for UPSIT parameters. The parameter is the number of correct responses out of 40 total items. *Visits are screening (baseline), Week 40/e0, and Week 80/e40. Add footnote: "Note: The UPSIT is a self-administered test which can be used to identify and quantitate olfactory dysfunction in PD. The number of correct responses out of 40 total items constitutes a subject's score. Lower scores represent greater olfactory dysfunction. Individual missing responses are imputed as zeros (ie, incorrect responses)." Add information in SAP Table 9 as an endnote.*

16.5 FIGURES

Figure 16.5.1.1.1 OFF State UPDRS Motor Score (Part III): Percentage Change Over Time - ITT Primary Population

Figure 14.X.X Title

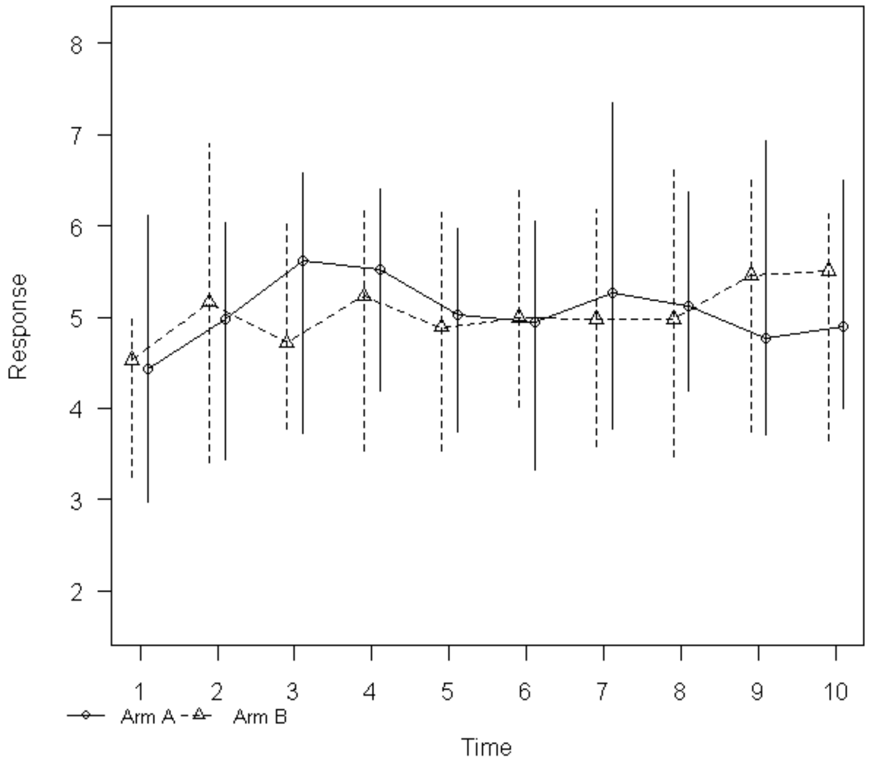

Note: Data points represent means, and error bars represent standard errors. For subject 45, items 22, 27, 28, 29 and 30 are excluded from the score. P-value from a mixed-effect model with repeated measures (MMRM) for the percentage change from baseline to Week 80/e40 between treatment groups is x.xxx.

Programming Note: Replace with appropriate titles, labels (eg, for y-axis “Change in OFF state UPDRS Motor Score from baseline [unit]”), and legend. Visits are Weeks 0, 8, 16, 24, 32, 40/e0, 48/e8, 56/e16, 64/e24, 72/e32 and 80/e40. Add a line for the modelled control using the values 3.3% at Week 40 and 6.7% at Week 80.

Figure 16.5.1.1.2 OFF State UPDRS Motor Score (Part III): Percentage Change Over Time - ITT Overall Population
Programming Note: Repeat figure above for OFF state UPDRS motor score (part III) percentage change over time in the ITT Overall Population. Visits are Weeks 0, 8, 16, 24, 32, 40/e0, 48/e8, 56/e16, 64/e24, 72/e32 and 80/e40. Add a line for the modelled control using the values 3.3% at Week 40 and 6.7% at Week 80.

Figure 16.5.1.2.1 OFF State UPDRS Motor Score (Part III): Change Over Time - ITT Primary Population
Programming Note: Repeat figure above for OFF state UPDRS motor score (part III) absolute change over time in the ITT Primary Population. Visits are Weeks 0, 8, 16, 24, 32, 40/e0, 48/e8, 56/e16, 64/e24, 72/e32 and 80/e40. Add a line for the modelled control using the values 1.17 at Week 40 and 2.34 at Week 80.

Figure 16.5.1.2.2 OFF State UPDRS Motor Score (Part III): Change Over Time - ITT Overall Population
Programming Note: Repeat figure above for OFF state UPDRS motor score (part III) absolute change over time in the ITT Overall Population. Visits are Weeks 0, 8, 16, 24, 32, 40/e0, 48/e8, 56/e16, 64/e24, 72/e32 and 80/e40. Add a line for the modelled control using the values 1.17 at Week 40 and 2.34 at Week 80.

Figure 16.5.1.3 ON State UPDRS Motor Score (Part III): Change Over Time - ITT Overall Population
Programming Note: Repeat figure above for ON state UPDRS motor score (part III) absolute change in the ITT Overall Population. Visits are Weeks 0, 8, 16, 24, 32, 40/e0, 48/e8, 56/e16, 64/e24, 72/e32 and 80/e40.

Figure 16.5.1.4.1 OFF State UPDRS ADL Score (Part II): Change Over Time - ITT Primary Population
Programming Note: Repeat figure above for OFF state UPDRS ADL score (part II) absolute change in the ITT Primary Population. Visits are Weeks 0, 8, 16, 24, 32, 40/e0, 48/e8, 56/e16, 64/e24, 72/e32 and 80/e40. Change footnote regarding subject 45 to: "Data for Subject 45 are excluded from analysis."

Figure 16.5.1.4.2 OFF State UPDRS ADL Score (Part II): Change Over Time - ITT Overall Population
Programming Note: Repeat figure above for OFF state UPDRS ADL score (part II) absolute change in the ITT Overall Population. Visits are Weeks 0, 8, 16, 24, 32, 40/e0, 48/e8, 56/e16, 64/e24, 72/e32 and 80/e40. Change footnote regarding subject 45 to: "Data for Subject 45 are excluded from analysis."

Figure 16.5.1.5 ON State UPDRS ADL Score (Part II): Change Over Time - ITT Overall Population
Programming Note: Repeat figure above for ON state UPDRS ADL score (part II) absolute change in the ITT Overall Population. Visits are Weeks 0, 8, 16, 24, 32, 40/e0, 48/e8, 56/e16, 64/e24, 72/e32 and 80/e40. Change footnote regarding subject 45 to: "Data for Subject 45 are excluded from analysis."

Figure 16.5.1.6.1 OFF State UPDRS Total Score: Change Over Time - ITT Primary Population
Programming Note: Repeat figure above for OFF state UPDRS total score (parts II+III) absolute change in the ITT Primary Population. Visits are Weeks 0, 8, 16, 24, 32, 40/e0, 48/e8, 56/e16, 64/e24, 72/e32 and 80/e40. Change footnote regarding subject 45 to: "Data for Subject 45 are excluded from analysis."

Figure 16.5.1.6.2 OFF State UPDRS Total Score: Change Over Time - ITT Overall Population
Programming Note: Repeat figure above for OFF state UPDRS total score (parts II+III) absolute change in the ITT Overall Population. Visits are Weeks 0, 8, 16, 24, 32, 40/e0, 48/e8, 56/e16, 64/e24, 72/e32 and 80/e40. Change footnote regarding subject 45 to: "Data for Subject 45 are excluded from analysis."

Figure 16.5.1.7 ON State UPDRS Total Score: Change Over Time - ITT Overall Population
Programming Note: Repeat figure above for ON state UPDRS total score (parts II+III) absolute change in the ITT Overall Population. Visits are Weeks 0, 8, 16, 24, 32, 40/e0, 48/e8, 56/e16, 64/e24, 72/e32 and 80/e40. Change footnote regarding subject 45 to: "Data for Subject 45 are excluded from analysis."

Figure 16.5.2.1 Motor Fluctuation Diary Total OFF Time Per Day (Hours): Change Over Time - ITT Primary Population

Programming Note: Repeat figure above for OFF time per day (hours). Visits are Weeks 0, 8, 16, 24, 32, 40/e0, 48/e8, 56/e16, 64/e24, 72/e32 and 80/e40. Delete footnote regarding subject 45.

Figure 16.5.2.2 Motor Fluctuation Diary Total OFF Time Per Day (Hours): Change Over Time - ITT Overall Population

Programming Note: Repeat figure above for ITT Overall Population.

Figure 16.5.2.3 Motor Fluctuation Diary Total Good-Quality ON Time Per Day (Hours): Change Over Time - ITT Primary Population

Programming Note: Repeat figure above for good-quality ON time per day (hours). Visits are Weeks 0, 8, 16, 24, 32, 40/e0, 48/e8, 56/e16, 64/e24, 72/e32 and 80/e40. Delete footnote regarding subject 45. Add footnote "Note: Total good-quality ON time per day is defined as the sum of ON time per day without dyskinesias + ON time per day with non-troublesome dyskinesias."

Figure 16.5.2.4 Motor Fluctuation Diary Total Good-Quality ON Time Per Day (Hours): Change Over Time - ITT Overall Population

Programming Note: Repeat figure above for ITT Overall Population.

Figure 16.5.3 OFF State UPDRS Motor Score (Part III): Subject Scores Over Time - ITT Primary Population

Figure	16.5.3
Title 1	OFF State UPDRS Motor Score (Part III): Subject Scores Over Time
Title 2	ITT Primary Population
Type of graph	Lineplot
y-axis	Individual subject OFF State UPDRS Motor Score
y-axis (label)	OFF State UPDRS Motor Score
x-axis	Visit
x-axis (label)	Visit
Legend (if applicable)	Not applicable
Footnote 1	Note: For subject 45, items 22, 27, 28, 29 and 30 are excluded from the score.
Footnote 2	
Additional information	GDNF/GDNF and placebo/GDNF groups should be on separate pages, keeping the y-axis scale the same for both

Figure 16.5.4.1 OFF State UPDRS Motor Score (Part III): Frequency Distribution of Change at Week 40/e0 - ITT Overall Population

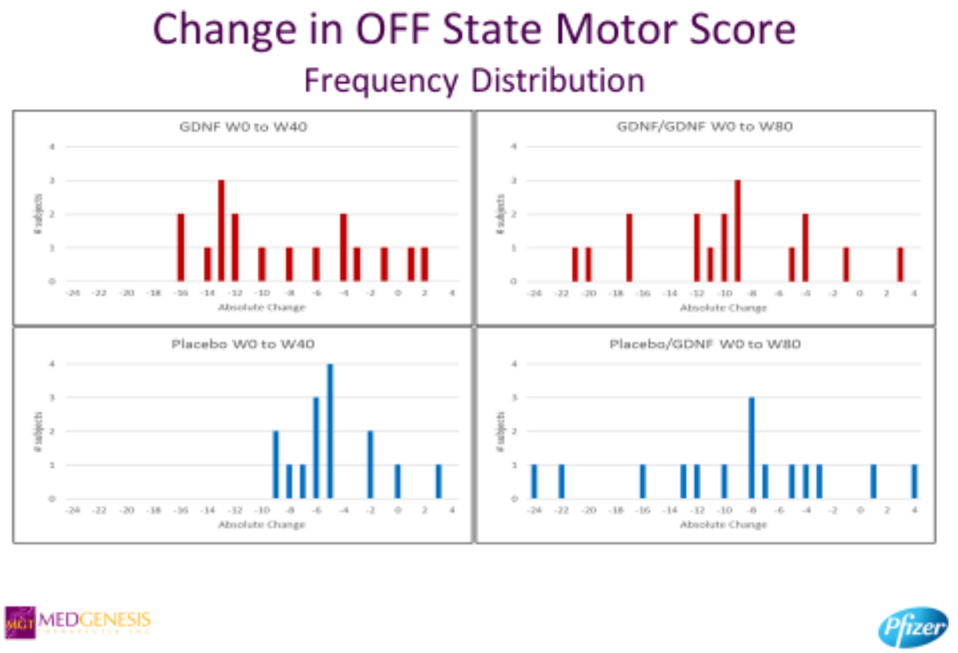

Note: For subject 45, items 22, 27, 28, 29 and 30 are excluded from the score.

Programming Note: Replace with appropriate titles, labels (eg, for x-axis "Change in OFF state UPDRS Motor Score at Week 40/e0 from baseline [unit]"), and legend. Only create one panel for change at Week 40 but include both treatment groups on the same graph with an appropriate legend.

Figure 16.5.4.2 OFF State UPDRS Motor Score (Part III): Frequency Distribution of Change at Week 80/e40 - ITT Overall Population
Programming Note: Repeat figure above for Week 80/e40.

Figure 16.5.5.1 Correlation Analysis of Percentage Change from Baseline to Week 80/e40 in OFF State UPDRS Motor Score (Part III) to Volume of Interest Coverage at Baseline as Determined by Contrast-Enhanced T1-Weighted MRI - ITT Primary Population

Figure	16.5.5.1
Title 1	Correlation Analysis of Percentage Change from Baseline to Week 80/e40 in OFF State UPDRS Motor Score (Part III) to Volume of Interest Coverage at Baseline as Determined by Contrast-Enhanced T1-Weighted MRI
Title 2	ITT Primary Population
Type of graph	Scatterplot
y-axis	Change From Baseline to Week 80/e40 in OFF State UPDRS Motor Score (Part III) (%)
y-axis (label)	Change From Baseline to Week 80/e40 in OFF State UPDRS Motor Score (Part III) (%)
x-axis	Volume of Interest Coverage at Baseline, Average of Both Putamina (%)
x-axis (label)	Volume of Interest Coverage at Baseline, Average of Both Putamina (%)
Legend (if applicable)	Treatment group symbol (please include N's)
Footnote 1	Note: Analysis uses non-parametric Spearman rank correlation. For subject 45, items 22, 27, 28, 29 and 30 are excluded from the score.
Footnote 2	
Additional information	Include estimated correlation coefficient value, p-value and line of best fit for each treatment group.

Figure 16.5.5.2 Correlation Analysis of Percentage Change from Baseline to Week 80/e40 in OFF State UPDRS Motor Score (Part III) to Total Putamenal Coverage at Baseline as Determined by Contrast-Enhanced T1-Weighted MRI - ITT Primary Population

Figure	16.5.5.2
Title 1	Correlation Analysis of Percentage Change from Baseline to Week 80/e40 in OFF State UPDRS Motor Score (Part III) to Total Putamenal Coverage at Baseline as Determined by Contrast-Enhanced T1-Weighted MRI
Title 2	ITT Primary Population
Type of graph	Scatterplot
y-axis	Change From Baseline to Week 80/e40 in OFF State UPDRS Motor Score (Part III) (%)
y-axis (label)	Change From Baseline to Week 80/e40 in OFF State UPDRS Motor Score (Part III) (%)
x-axis	Total Putamenal Coverage at Baseline, Average of Both Putamina (%)
x-axis (label)	Total Putamenal Coverage at Baseline, Average of Both Putamina (%)
Legend (if applicable)	Treatment group symbol (please include N's)
Footnote 1	Note: Analysis uses non-parametric Spearman rank correlation. For subject 45, items 22, 27, 28, 29 and 30 are excluded from the score.
Footnote 2	
Additional information	Include estimated correlation coefficient value, p-value and line of best fit for each treatment group.

Figure 16.5.5.3 Correlation Analysis of Percentage Change from Baseline to Week 80/e40 in OFF State UPDRS Motor Score (Part III) to Change From Baseline to Week 40/e0 in ^{18}F-DOPA Uptake as Determined by PET Scan - ITT Primary Population

Programming Note: Repeat above figures. Parameters are "Percentage change from baseline to Week 80/e40 in OFF state UPDRS motor score (part III)" and "Change from baseline to Week 40/e0 in ^{18}F-DOPA uptake rate constant, average of both hemispheres". Perform separate analyses for each of the 5 regions (dorsal caudate nucleus, dorsal anterior putamen, dorsal central/posterior putamen, ventral striatum, and substantia nigra). ***Figure will have 5 pages, one for each of 5 regions; please subtitle each page clearly.***

Figure 16.5.5.4 Correlation Analysis of Baseline (Week 0) OFF State UPDRS Motor Score (Part III) to Baseline (Week 0) ^{18}F-DOPA Uptake as Determined by PET Scan for All Subjects - ITT Overall Population

Programming Note: Repeat above figure. Parameters are "Baseline (Week 0) OFF state UPDRS motor score (part III)" and "Baseline (Week 0) ^{18}F-DOPA uptake rate constant for dorsal central/posterior putamen, average of both hemispheres".

Figure 16.5.5.5 Correlation Analysis of Baseline (Week 0) OFF State UPDRS Motor Score (Part III) to Baseline (Week 0) ^{18}F-DOPA Uptake as Determined by PET Scan for GDNF/GDNF Subjects - ITT Overall Population

Programming Note: Repeat above figure. Parameters are "Baseline (Week 0) OFF state UPDRS motor score (part III)" and "Baseline (Week 0) ^{18}F-DOPA uptake rate constant for dorsal central/posterior putamen, average of both hemispheres".

Figure 16.5.5.6 Correlation Analysis of Baseline (Week 0) OFF State UPDRS ADLScore (Part II) to Baseline (Week 0) ^{18}F-DOPA Uptake as Determined by PET Scan for All Subjects - ITT Overall Population

Programming Note: Repeat above figure. Parameters are "Baseline (Week 0) OFF state UPDRS ADL score (part II)" and "Baseline (Week 0) ^{18}F-DOPA uptake rate constant for dorsal central/posterior putamen, average of both hemispheres".

Figure 16.5.5.7 Correlation Analysis of Baseline (Week 0) OFF State UPDRS ADL Score (Part II) to Baseline (Week 0) ^{18}F-DOPA Uptake as Determined by PET Scan for GDNF/GDNF Subjects - ITT Overall Population

Programming Note: Repeat above figure. Parameters are "Baseline (Week 0) OFF state UPDRS ADL score (part II)" and "Baseline (Week 0) ^{18}F-DOPA uptake rate constant for dorsal central/posterior putamen, average of both hemispheres".

Figure 16.5.5.8 Correlation Analysis of Week 40/e0 OFF State UPDRS Motor Score (Part III) to Week 40/e0 ^{18}F-DOPA Uptake as Determined by PET Scan for All Subjects - ITT Overall Population

Programming Note: Repeat above figure. Parameters are "Week 40/e0 OFF state UPDRS motor score (part III)" and "Week 40/e0 ^{18}F-DOPA uptake rate constant for dorsal central/posterior putamen, average of both hemispheres".

Figure 16.5.5.9 Correlation Analysis of Week 40/e0 OFF State UPDRS Motor Score (Part III) to Week 40/e0 ^{18}F-DOPA Uptake as Determined by PET Scan for GDNF/GDNF Subjects - ITT Overall Population

Programming Note: Repeat above figure. Parameters are "Week 40/e0 OFF state UPDRS motor score (part III)" and "Week 40/e0 ^{18}F-DOPA uptake rate constant for dorsal central/posterior putamen, average of both hemispheres".

Figure 16.5.5.10 Correlation Analysis of Week 40/e0 OFF State UPDRS ADLScore (Part II) to Week 40/e0 ^{18}F-DOPA Uptake as Determined by PET Scan for All Subjects - ITT Overall Population

Programming Note: Repeat above figure. Parameters are "Week 40/e0 OFF state UPDRS ADL score (part II)" and "Week 40/e0 ^{18}F-DOPA uptake rate constant for dorsal central/posterior putamen, average of both hemispheres".

Figure 16.5.5.11 Correlation Analysis of Week 40/e0 OFF State UPDRS ADL Score (Part II) to Week 40/e0 ^{18}F-DOPA Uptake as Determined by PET Scan for GDNF/GDNF Subjects - ITT Overall Population

Programming Note: Repeat above figure. Parameters are "Week 40/e0 OFF state UPDRS ADL score (part II)" and "Week 40/e0 ^{18}F-DOPA uptake rate constant for dorsal central/posterior putamen, average of both hemispheres".

Figure 16.5.5.12 Correlation Analysis of Week 80/e40 OFF State UPDRS Motor Score (Part III) to Week 40/e0 ^{18}F-DOPA Uptake as Determined by PET Scan for All Subjects - ITT Overall Population

Programming Note: Repeat above figure. Parameters are "Week 80/e40 OFF state UPDRS motor score (part III)" and "Week 40/e0 ^{18}F-DOPA uptake rate constant for dorsal central/posterior putamen, average of both hemispheres".

Figure 16.5.5.13 Correlation Analysis of Week 80/e40 OFF State UPDRS Motor Score (Part III) to Week 40/e0 ^{18}F-DOPA Uptake as Determined by PET Scan for GDNF/GDNF Subjects - ITT Overall Population

Programming Note: Repeat above figure. Parameters are "Week 80/e40 OFF state UPDRS motor score (part III)" and "Week 40/e0 ^{18}F-DOPA uptake rate constant for dorsal central/posterior putamen, average of both hemispheres".

Figure 16.5.5.14 Correlation Analysis of Week 80/e40 OFF State UPDRS ADLScore (Part II) to Week 40/e0 ^{18}F-DOPA Uptake as Determined by PET Scan for All Subjects - ITT Overall Population

Programming Note: Repeat above figure. Parameters are "Week 80/e40 OFF state UPDRS ADL score (part II)" and "Week 40/e0 ^{18}F-DOPA uptake rate constant for dorsal central/posterior putamen, average of both hemispheres".

Figure 16.5.5.15 Correlation Analysis of Week 80/e40 OFF State UPDRS ADL Score (Part II) to Week 40/e0 ^{18}F-DOPA Uptake as Determined by PET Scan for GDNF/GDNF Subjects - ITT Overall Population

Programming Note: Repeat above figure. Parameters are "Week 80/e40 OFF state UPDRS ADL score (part II)" and "Week 40/e0 ^{18}F-DOPA uptake rate constant for dorsal central/posterior putamen, average of both hemispheres".

17 DATA LISTINGS

17.2.1 BASELINE LISTINGS

Listing 17.2.1.1 Study Completion Status - ITT Overall Population

ITT Pilot Stage (N=XXX)

GDNF/GDNF (N=XXX)

Subject ID Age/Race/Ethnicity/ Sex	Completed Initial Extension?	Last visit	Date of Last Dose of Study Medication	Date of Discontinuation	Primary Reason for Discontinuation
xxxxxxxxxx/xx/x/x/x	Yes/No	Week XX, DDMMMYYYY	DDMMMYYYY	DDMMMYYYY	Xxxxxxxxxxxxxxxxxxxxxxxxxxxxxxxxxxxxxxxxxxx

Note: W = white; B = black; P = native Hawaiian or other Pacific islander; A = Asian; I = American Indian or Alaska native; H = Hispanic or Latino; N = not Hispanic or Latino; M = male; F = female.
Source: Dataset: [NAME], Program: xxxxxx.sas, Output: xxxx.rtf, Generated on: DDMONYYYY HH:MM
Page x of y

Programming Note: Repeat listing for ITT Pilot Stage (N=XXX) Placebo/GDNF (N=XXX), then ITT Primary Stage (N=XXX) GDNF/GDNF (N=XXX) and Placebo/GDNF (N=XXX). Sort by stage, treatment group, and subject ID. Include all ITT Overall subjects. If primary reason for discontinuation is Other, then concatenate specify text.

Listing 17.2.1.2.1 Major Protocol Deviations - ITT Overall Population

ITT Pilot Stage (N=XXX)			
GDNF/GDNF (N=XXX)			
**Subject ID Age/Race/Ethnicity/ Sex**	**Date of Deviation**	**Category**	**Deviation (brief description)**
xxxxxxxxxx/xx/x/x/x	DDMMMYYYY	Xxxxxxxxxxxxxxxxxxxx	Xxxxxxxxxxxxxxxxxxxxxxxxxxxxxxxxxxxxxxxxxxxxxxxxxxxxxxxxxxxxxx

Note: W = white; B = black; P = native Hawaiian or other Pacific islander; A = Asian; I = American Indian or Alaska native; H = Hispanic or Latino; N = not Hispanic or Latino; M = male; F = female.
Source: Dataset: [NAME], Program: xxxxxx.sas, Output: xxxx.rtf, Generated on: DDMONYYYY HH:MM
Page x of y

Programming Note: Repeat listing for ITT Pilot Stage (N=XXX) Placebo/GDNF (N=XXX), then ITT Primary Stage (N=XXX) GDNF/GDNF (N=XXX) and Placebo/GDNF (N=XXX). Sort by stage, treatment group, and subject ID. Include all ITT Overall subjects with major protocol deviations.

Listing 17.2.1.2.2 Minor Protocol Deviations - ITT Overall Population

Programming Note: Repeat listing above for minor protocol deviations.

Listing 17.2.1.3 Subject Populations - ITT Overall Population

ITT Pilot Stage (N=XXX)			
GDNF/GDNF (N=XXX)			
**Subject ID Age/Race/Ethnicity/Sex**	**Treatment Received?**	**Safety Population**	**ITT Population**
xxxxxxxxxx/xx/x/x/x	Yes	Yes	Yes

Note: W = white; B = black; P = native Hawaiian or other Pacific islander; A = Asian; I = American Indian or Alaska native; H = Hispanic or Latino; N = not Hispanic or Latino; M = male; F = female.

<Subjects who are not treated are excluded from the safety population; subjects with no postbaseline assessments are excluded from the ITT population.>

Source: Dataset: [NAME], Program: xxxxxx.sas, Output: xxxx.rtf, Generated on: DDMONYYYY HH:MM

Page x of y

Programming Note: Repeat listing for ITT Pilot Stage (N=XXX) Placebo/GDNF (N=XXX), then ITT Primary Stage (N=XXX) GDNF/GDNF (N=XXX) and Placebo/GDNF (N=XXX). Sort by stage, treatment group, and subject ID. Include all ITT Overall subjects.

Listing 17.2.1.4.1 Demographic Characteristics in Study 2553 - ITT Overall Population

			ITT Pilot Stage (N=XXX)		
			GDNF/GDNF (N=XXX)		
**Subject ID Age/Race/Ethnicity/ Sex**	**Date of Birth**	**Race (specify if Other)**	**Baseline Weight (kg) Height (m)**	**Baseline BMI (kg/m^2)**	**NART Error Score (points)**
xxxxxxxxxx/xx/x/x/x	DDMMMYYYY	Xxxxxxxxxxx	xxx.x<br>xxx.x	xx.x	xx

Note: W = white; B = black; P = native Hawaiian or other Pacific islander; A = Asian; I = American Indian or Alaska native; H = Hispanic or Latino; N = not Hispanic or Latino; M = male; F = female.
Source: Dataset: [NAME], Program: xxxxxx.sas, Output: xxxx.rtf, Generated on: DDMONYYYY HH:MM
Page x of y

Programming Note: Repeat listing for ITT Pilot Stage (N=XXX) Placebo/GDNF (N=XXX), then ITT Primary Stage (N=XXX) GDNF/GDNF (N=XXX) and Placebo/GDNF (N=XXX). Sort by stage, treatment group, and subject ID. Include all ITT Overall subjects.

Listing 17.2.1.4.2 Parkinson's Disease History at Screening in Study 2553 - ITT Overall Population

ITT Pilot Stage (N=XXX)							
GDNF/GDNF (N=XXX)							
Subject ID Age/Race/Ethnicity/ Sex	Date of First PD Symptom	Date of PD Diagnosis	Hoehn and Yahr Stage in OFF State	UPDRS Motor Score (Part III) OFF State	UPDRS Motor Score (Part III) ON State	OFF Time per Day (hours)	Response to Levodopa[a] (%)
xxxxxxxxxx/xx/x/x/x	DDMMMYYYY	DDMMMYYYY	x	xx	xx	xx	xx.x

[a] Percentage improvement in screening UPDRS motor score (part III) following a levodopa challenge.

Note: W = white; B = black; P = native Hawaiian or other Pacific islander; A = Asian; I = American Indian or Alaska native; H = Hispanic or Latino; N = not Hispanic or Latino; M = male; F = female.

Source: Dataset: [NAME], Program: xxxxxx.sas, Output: xxxx.rtf, Generated on: DDMONYYYY HH:MM

Page x of y

Programming Note: Repeat listing for ITT Pilot Stage (N=XXX) Placebo/GDNF (N=XXX), then ITT Primary Stage (N=XXX) GDNF/GDNF (N=XXX) and Placebo/GDNF (N=XXX). Sort by stage, treatment group, and subject ID. Include all ITT Overall subjects.

Listing 17.2.1.5 Concomitant Medications – ITT Overall Population

ITT Pilot Stage (N=XXX)

GDNF/GDNF (N=XXX)

Subject ID<br>Age/Race/Ethnicity/<br>Sex	ATC Class<br>Coded Medication Name<br>Verbatim Medication Name	Start Date (Study Day)/<br>Stop Date (Study Day)<br>or Ongoing	Dose per<br>Frequenc<br>y	Unit	Frequency	Indication
xxxxxxxxxx/xx/x/x/x	xxxxxxxxxxxxxxxxxxx<br>xxxxxxxxxxxxxxxxxxx<br>xxxxxxxxxxxxxx	DDMONYYYY (xxx)/<br>DDMONYYYY (xxx)	xxx	xxx	xxx	xxxxxxxxxxxx

Note: Medications are coded using WHODRUG DDE version MONYYYY. W = white; B = black; P = native Hawaiian or other Pacific islander; A = Asian; I = American Indian or Alaska native; H = Hispanic or Latino; N = not Hispanic or Latino; M = male; F = female.
Source: Dataset: [NAME], Program: xxxxxx.sas, Output: xxxx.rtf, Generated on: DDMONYYYY HH:MM
Page x of y

Programming Note: Repeat listing for ITT Pilot Stage (N=XXX) Placebo/GDNF (N=XXX), then ITT Primary Stage (N=XXX) GDNF/GDNF (N=XXX) and Placebo/GDNF (N=XXX). Sort by stage, treatment group, subject ID and start date. Include all ITT Overall subjects with data.

Listing 17.2.1.6 Catheter Trajectory - ITT Overall Population

ITT Pilot Stage (N=XXX)			
GDNF/GDNF (N=XXX)			
**Subject ID Age/Race/Ethnicity/ Sex**	**Surgery**	**Date of Surgery (Study Day)**	**Catheter Placement**
xxxxxxxxxx/xx/x/x/x	Respositioning Surgery x	DDMONYYYY (xxx)	Vertical/ Horizontal: Anterior-Posterior/ Horizontal: Posterior-Anterior

Note: W = white; B = black; P = native Hawaiian or other Pacific islander; A = Asian; I = American Indian or Alaska native; H = Hispanic or Latino; N = not Hispanic or Latino; M = male; F = female.
Source: Dataset: [NAME], Program: xxxxxx.sas, Output: xxxx.rtf, Generated on: DDMONYYYY HH:MM
Page x of y

Programming Note: Repeat listing for ITT Pilot Stage (N=XXX) Placebo/GDNF (N=XXX), then ITT Primary Stage (N=XXX) GDNF/GDNF (N=XXX) and Placebo/GDNF (N=XXX). Sort by stage, treatment group, subject ID and date of surgery. Include all ITT Overall subjects with data.

Listing 17.2.1.7 Catheter Positioning Accuracy by Surgery - ITT Overall Population

ITT Pilot Stage (N=XXX)

GDNF/GDNF (N=XXX)

Subject Age/Race/ Ethnicity/ Sex	Surgery	Date and Time	Distance Between Planned Target and Actual Target (mm) Catheter 1	Catheter 2	Catheter 3	Catheter 4	Mean Across All Catheters	Catheter Positioning Satisfactory	Hemorrhage
xxxxxxxxxx/xx /x/x/x	Repositioning Surgery #1	DDMONYYYY HHMM	xx.x	xx.x	xx.x	xx.x	xx.x	Satisfactory<br>Required Repositioning<br>Other: xxxxxxx	No Hemorrhage Detected<br>Minor Hemorrhage without Clinical Signs<br>Minor Hemorrhage with Clinical Signs<br>Major Hemorrhage
		DDMONYYYY HHMM	xx.x	xx.x	xx.x	xx.x	xx.x		

Note: W = white; B = black heritage; P = native Hawaiian or other Pacific islander; A = Asian; I = American Indian or Alaska native; H = Hispanic or Latino; N = not Hispanic or Latino; M = male; F = female.
Source: Dataset: [NAME], Program: xxxxxx.sas, Output: xxxx.rtf, Generated on: DDMONYYYY HH:MM
Page x of y

Programming Note: Repeat listing for ITT Pilot Stage (N=XXX) Placebo/GDNF (N=XXX), then ITT Primary Stage (N=XXX) GDNF/GDNF (N=XXX) and Placebo/GDNF (N=XXX). Sort by stage, treatment group, and subject ID then surgery. Include all ITT Overall subjects with data. Surgeries are Initial Surgery, Repositioning surgery #1, and so on. Abbreviate as necessary.

Listing 17.2.1.8 Test Infusion Data by Visit - ITT Overall Population

ITT Pilot Stage (N=XXX)							
GDNF/GDNF (N=XXX)							
**Subject ID Age/Race/Ethnicity / Sex**	**Visit**	**Date**	**Time First Start Time Last Stop**	**Catheter 1**	**Catheter 2**	**Catheter 3**	**Catheter 4**
xxxxxxxxxx/xx/x/x/x	Week 40/e0	DDMONYYYY	HH:MM HH:MM	Standard	Standard	Standard	Not Used
		DDMONYYYY	HH:MM HH:MM	Non-standard	Standard	Standard	Not Used

Note: W = white; B = black; P = native Hawaiian or other Pacific islander; A = Asian; I = American Indian or Alaska native; H = Hispanic or Latino; N = not Hispanic or Latino; M = male; F = female.
Note: Standard regime is a linear ramp up infusion rate of 3-5 µL/min with 400 µL total volume.
Source: Dataset: [NAME], Program: xxxxxx.sas, Output: xxxx.rtf, Generated on: DDMONYYYY HH:MM
Page x of y

Programming Note: Repeat listing for ITT Pilot Stage (N=XXX) Placebo/GDNF (N=XXX), then ITT Primary Stage (N=XXX) GDNF/GDNF (N=XXX) and Placebo/GDNF (N=XXX). Sort by stage, treatment group, subject ID, visit, and date of infusion. Include all ITT Overall subjects with data. Include all test infusions , including repeat or unscheduled test infusions, from all extension parts of Study 2797. For all listings by visit, use actual visit labels and not "screening" or "baseline."

Listing 17.2.1.9 Test Infusion Catheter Interruptions/Early Terminations by Visit - ITT Overall Population

ITT Pilot Stage (N=XXX)

GDNF/GDNF (N=XXX)

Subject ID Age/Race/Ethnicity / Sex	Visit	Date	Time First Start Time Last Stop	Catheter No. Interrupted/ Terminated Early	Time Infusion Stopped	Time Infusion Restarted	Reason for Stop
xxxxxxxxxx/xx/x/x/x	Week 40/e0	DDMONYYYY	HH:MM HH:MM	1	HH:MM	HH:MM	xxxxxx
				2	HH:MM	HH:MM	xxxxxx
				3	HH:MM	HH:MM	xxxxxx
				4	HH:MM	HH:MM	xxxxxx
		DDMONYYYY	HH:MM HH:MM	1	HH:MM	HH:MM	xxxxxx
				2	HH:MM	HH:MM	xxxxxx
				3	HH:MM	HH:MM	xxxxxx
				4	HH:MM	HH:MM	xxxxxx

Note: W = white; B = black; P = native Hawaiian or other Pacific islander; A = Asian; I = American Indian or Alaska native; H = Hispanic or Latino; N = not Hispanic or Latino; M = male; F = female.
Source: Dataset: [NAME], Program: xxxxxx.sas, Output: xxxx.rtf, Generated on: DDMONYYYY HH:MM
Page x of y

Programming Note: Repeat listing for ITT Pilot Stage (N=XXX) Placebo/GDNF (N=XXX), then ITT Primary Stage (N=XXX) GDNF/GDNF (N=XXX) and Placebo/GDNF (N=XXX). Sort by stage, treatment group, subject ID, visit, and date of infusion. Include all ITT Overall subjects with data. Include all test infusions , including repeat or unscheduled test infusions, from all extension parts of Study 2797. For all listings by visit, use actual visit labels and not "screening" or "baseline."

17.2.2 Efficacy Listings

Listing 17.2.2.1 OFF and ON state UPDRS Scores by Visit - ITT Overall Population

ITT Pilot Stage (N=XXX)

GDNF/GDNF (N=XXX)

Subject ID Age/Race/ Ethnicity/ Sex	Parameter (points)	Visit	Date and Time	Result	Change From Baseline
xxxxxxxxxx/xx /x/x/x	OFF state UPDRS motor score (part III)	Week 0	DDMONYYYY HHMM	xxx	
		Week 40/e0	DDMONYYYY HHMM	xxx	xxx.x
		Week 48/e8	DDMONYYYY HHMM	xxx	xxx.x
		…	…	…	…
	ON state UPDRS motor score (part III)	Week 0	DDMONYYYY HHMM	xxx	
		Week 40/e0	DDMONYYYY HHMM	xxx	xxx.x
		Week 48/e8	DDMONYYYY HHMM	xxx	xxx.x
		…	…	…	…
	OFF state UPDRS ADL score (part II)	Week 0	DDMONYYYY HHMM	xxx	
		Week 40/e0	DDMONYYYY HHMM	xxx	xxx.x
		Week 48/e8	DDMONYYYY HHMM	xxx	xxx.x
		…	…	…	…
	ON state UPDRS ADL score (part II)	Week 0	DDMONYYYY HHMM	xxx	
		Week 40/e0	DDMONYYYY HHMM	xxx	xxx.x
		Week 48/e8	DDMONYYYY HHMM	xxx	xxx.x
	etc.	…	…	…	…

Note: Lower scores represent better functioning. OFF and ON state UPDRS total score are sums of motor score (part III) and ADL score (part II). For subject 45, items 22, 27, 28, 29 and 30 are excluded from the motor score (part III). Subject 45 had observed OFF state UPDRS motor score (part III) values of XX, XX, … at visits X, X, … respectively. Subject 45 had observed ON state UPDRS motor score (part III) values of XX, XX, … at visits X, X, … respectively. The observed OFF state and ON state UPDRS motor scores at Screening of subject 45 are used in the calculation of summary statistics in Tables 16.1.4.1 and 16.1.4.2. (Parkinson's Disease History at Screening from Study 2553). W = white; B = black; P = native Hawaiian or other Pacific islander; A = Asian; I = American Indian or Alaska native; H = Hispanic or Latino; N = not Hispanic or Latino; M = male; F = female.

Source: Dataset: [NAME], Program: xxxxxx.sas, Output: xxxx.rtf, Generated on: DDMONYYYY HH:MM

Page x of y

Programming Note: Repeat listing for ITT Pilot Stage (N=XXX) Placebo/GDNF (N=XXX), then ITT Primary Stage (N=XXX) GDNF/GDNF (N=XXX) and Placebo/GDNF (N=XXX). Sort by stage, treatment group, and subject ID then parameter, OFF/ON state, and visit. Include all ITT Overall subjects with data. Parameters are OFF and ON state UPDRS Motor score (part III), OFF and ON state UPDRS Activities of daily living score (part II), OFF and ON state UPDRS

total score (sum of motor + ADL scores), Mentation, behavior, and mood score (part I), and Complications of therapy score (part IV). Visits are Screening Visit 2, Weeks 0, 40/e0, 48/e8, 56/e16, 64/e24, 72/e32 and 80/e40 and all Pilot and Supplemental Extension visits. Baseline is Week 0 for all parameters. Abbreviate as necessary. Do not include imputed values. For all listings by visit, use actual visit labels and not "screening" or "baseline."

Listing 17.2.2.2 PD Motor Fluctuation Diary Ratings by Visit - ITT Overall Population

ITT Pilot Stage (N=XXX)

GDNF/GDNF (N=XXX)

Subject ID Age/Race/ Ethnicity/ Sex	Parameter (hours)	Visit	Date	Result	Change From Baseline
xxxxxxxxxx/xx /x/x/x	OFF time per day	Week 0	DDMONYYYY	xx	
		Week 40/e0	DDMONYYYY	xx	xx.x
		Week 48/e8	DDMONYYYY	xx	xx.x
		…	…	…	…
	Total good-quality ON time per day	Week 0	DDMONYYYY	xx	
		Week 40/e0	DDMONYYYY	xx	xx.x
		Week 48/e8	DDMONYYYY	xx	xx.x
		…	…	…	…
	ON time per day without dyskinesias	Week 0	DDMONYYYY	xx	
		Week 40/e0	DDMONYYYY	xx	xx.x
		Week 48/e8	DDMONYYYY	xx	xx.x
		…	…	…	…
	ON time per day with non-troublesome dyskinesias	Week 0	DDMONYYYY	xx	
		Week 40/e0	DDMONYYYY	xx	xx.x
		Week 48/e8	DDMONYYYY	xx	xx.x
		…	…	…	…
	ON time per day with troublesome dyskinesias	Week 0	DDMONYYYY	xx	
		Week 40/e0	DDMONYYYY	xx	xx.x
		Week 48/e8	DDMONYYYY	xx	xx.x
	etc.	…	…	…	…

Note: W = white; B = black; P = native Hawaiian or other Pacific islander; A = Asian; I = American Indian or Alaska native; H = Hispanic or Latino; N = not Hispanic or Latino; M = male; F = female.
Note: Total good-quality ON time per day is defined as the sum of ON time per day without dyskinesias + ON time per day with non-troublesome dyskinesias.
Source: Dataset: [NAME], Program: xxxxxx.sas, Output: xxxx.rtf, Generated on: DDMONYYYY HH:MM
Page x of y

Programming Note: Repeat listing for ITT Pilot Stage (N=XXX) Placebo/GDNF (N=XXX), then ITT Primary Stage (N=XXX) GDNF/GDNF (N=XXX) and Placebo/GDNF (N=XXX). Sort by stage, treatment group, and subject ID then parameter, and visit. Include all ITT Overall subjects with data. PD diary parameters are OFF time per day, total good-quality ON time per day (sum of ON time per day without dyskinesias + ON time per day with non-troublesome dyskinesias), ON time per day without dyskinesias, ON time per day with non-troublesome dyskinesias, and ON time per day with troublesome dyskinesias. Visits are Screening Visit 2, Weeks 0, 40/e0, 48/e8, 56/e16, 64/e24, 72/e32 and 80/e40 and all Pilot and Supplemental Extension visits. Baseline is Week 0. Abbreviate as necessary. For all listings by visit, use actual visit labels and not "screening" or "baseline."

Listing 17.2.2.3 Treatment Response at Week 40/e0 and Week 80/e40 - ITT Overall Population

ITT Pilot Stage (N=XXX)

GDNF/GDNF (N=XXX)

Subject ID/Age/Race/ Ethnicity/Sex	Criteria	Treatment Responder
xxxxxxxxxx/xx/x/x/x	Decrease from baseline to Week 40/e0 by ≥10 points in OFF state UPDRS motor score (part III)	Yes/No
	Increase from baseline to Week 40/e0 by ≥1 hour in total good quality ON time per day (ON without dyskinesias or ON with non troublesome dyskinesias)	Yes/No
	Decrease from baseline to Week 40/e0 by ≥10 points in OFF state UPDRS motor score (part III) and increase from baseline to Week 40/e0 by ≥1 hour in total good quality ON time per day (ON without dyskinesias or ON with non troublesome dyskinesias)	Yes/No
	Decrease from baseline to Week 80/e40 by ≥10 points in OFF state UPDRS motor score (part III)	Yes/No
	Increase from baseline to Week 80/e40 by ≥1 hour in total good quality ON time per day (ON without dyskinesias or ON with non troublesome dyskinesias)	Yes/No
	Decrease from baseline to Week 80/e40 by ≥10 points in OFF state UPDRS motor score (part III) and increase from baseline to Week 80/e40 by ≥1 hour in total good quality ON time per day (ON without dyskinesias or ON with non troublesome dyskinesias)	Yes/No
xxxxxxxxxx/xx/x/x/x	Decrease from baseline to Week 40/e0 by ≥10 points in OFF state UPDRS motor score (part III)	Yes/No
	Increase from baseline to Week 40/e0 by ≥1 hour in total good quality ON time per day (ON without dyskinesias or ON with non troublesome dyskinesias)	Yes/No
	Decrease from baseline to Week 40/e0 by ≥10 points in OFF state UPDRS motor score (part III) and increase from baseline to Week 40/e0 by ≥1 hour in total good quality ON time per day (ON without dyskinesias or ON with non troublesome dyskinesias)	
	Decrease from baseline to Week 80/e40 by ≥10 points in OFF state UPDRS motor score (part III)	
	Increase from baseline to Week 80/e40 by ≥1 hour in total good quality ON time per day (ON without dyskinesias or ON with non troublesome dyskinesias)	
	Decrease from baseline to Week 80/e40 by ≥10 points in OFF state UPDRS motor score (part III) and increase from baseline to Week 80/e40 by ≥1 hour in total good quality ON time per day (ON without dyskinesias or ON with non troublesome dyskinesias)	

Note: W = white; B = black; P = native Hawaiian or other Pacific islander; A = Asian; I = American Indian or Alaska native; H = Hispanic or Latino; N = not Hispanic or Latino; M = male; F = female.
Source: Dataset: [NAME], Program: xxxxxx.sas, Output: xxxx.rtf, Generated on: DDMONYYYY HH:MM
Page x of y

Programming Note: Repeat listing for ITT Pilot Stage (N=XXX) Placebo/GDNF (N=XXX), then ITT Primary Stage (N=XXX) GDNF/GDNF (N=XXX) and Placebo/GDNF (N=XXX). Sort by stage, treatment group, and subject ID then visit. Include all ITT Overall subjects with data. For all listings by visit, use actual visit labels and not "screening" or "baseline."

Listing 17.2.2.4.1 OFF and ON State Timed Walking Test by Visit - ITT Overall Population

ITT Pilot Stage (N=XXX)

GDNF/GDNF (N=XXX)

Subject Age/Race/ Ethnicity/ Sex	Parameter (seconds)	Visit	Date and Time	Result (mean of replicates)	Change From Baseline
xxxxxxxxxx/xx /x/x/x	OFF state timed walking test	Screening Visit 2	DDMONYYYY HHMM	xx	
		Week 0	DDMONYYYY HHMM	xx	
		Week 40/e0	DDMONYYYY HHMM	xx	xx.x
		…	…	…	…
	ON state timed walking test	Screening Visit 2	DDMONYYYY HHMM	xx	
		Week 0	DDMONYYYY HHMM	xx	
		Week 40/e0	DDMONYYYY HHMM	xx	xx.x
		…	…	…	…

Note: Shorter times represent better function. Two trials per state per visit are averaged for analysis. If only the first trial is completed for a particular state, then the second trial result will be imputed with the worst non-missing second trial result from all subjects for that particular state during the study. If both trials are missing, then the endpoint is not reported for that visit. W = white; B = black; P = native Hawaiian or other Pacific islander; A = Asian; I = American Indian or Alaska native; H = Hispanic or Latino; N = not Hispanic or Latino; M = male; F = female.
Source: Dataset: [NAME], Program: xxxxxx.sas, Output: xxxx.rtf, Generated on: DDMONYYYY HH:MM
Page x of y

Programming Note: Same format as Listing 17.2.2.1. Repeat listing for ITT Pilot Stage (N=XXX) Placebo/GDNF (N=XXX), then ITT Primary Stage (N=XXX) GDNF/GDNF (N=XXX) and Placebo/GDNF (N=XXX). Sort by stage, treatment group, and subject ID then parameter, OFF/ON state, and visit. Include all ITT Overall subjects with data. Parameters are OFF state timed walking test and ON state timed walking test. Visits are Screening Visit 2, Weeks 0, 40/e0, 48/e8, 56/e16, 64/e24, 72/e32 and 80/e40 and all Pilot and Supplemental Extension visits. Baseline is Week 0. Abbreviate as necessary. For all listings by visit, use actual visit labels and not "screening" or "baseline."

Listing 17.2.2.4.2 OFF and ON State Timed Tapping Test by Visit - ITT Overall Population

ITT Pilot Stage (N=XXX)

GDNF/GDNF (N=XXX)

Subject Age/Race/ Ethnicity/ Sex	Parameter (taps)	Visit	Date and Time	Result (mean of replicates)	Change From Baseline
xxxxxxxxxx/xx /x/x/x	OFF state timed tapping test (left hand)	Screening Visit 2	DDMONYYYY HHMM	xx	
		Week 0	DDMONYYYY HHMM	xx	
		Week 40/e0	DDMONYYYY HHMM	xx	xx.x
		…	…	…	…
	OFF state timed tapping test (right hand)	Screening Visit 2	DDMONYYYY HHMM	xx	
		Week 0	DDMONYYYY HHMM	xx	
		Week 40/e0	DDMONYYYY HHMM	xx	xx.x
		…	…	…	…
	OFF state timed tapping test (both hands)	Screening Visit 2	DDMONYYYY HHMM	xx	
		Week 0	DDMONYYYY HHMM	xx	
		Week 40/e0	DDMONYYYY HHMM	xx	xx.x
		…	…	…	…

Note: More taps represent better function. The four separate trials per state for each visit are averaged for analysis. If any trial is not completed for a particular state, then the trial result will be imputed with the worst non-missing applicable trial result (for example, first left hand trial) from all subjects for that particular state during the study. If all four trials are missing, then the endpoint is not reported for that visit. W = white; B = black; P = native Hawaiian or other Pacific islander; A = Asian; I = American Indian or Alaska native; H = Hispanic or Latino; N = not Hispanic or Latino; M = male; F = female.
Source: Dataset: [NAME], Program: xxxxxx.sas, Output: xxxx.rtf, Generated on: DDMONYYYY HH:MM
Page x of y

Programming Note: Same format as Listing 17.2.2.1. Repeat listing for ITT Pilot Stage (N=XXX) Placebo/GDNF (N=XXX), then ITT Primary Stage (N=XXX) GDNF/GDNF (N=XXX) and Placebo/GDNF (N=XXX). Sort by stage, treatment group, and subject ID then parameter, OFF/ON state, and visit. Include all ITT Overall subjects with data. Parameters are OFF state timed tapping test (left hand), OFF state timed tapping test (right hand), OFF state timed tapping test (both hands), ON state timed tapping test (left hand), ON state timed tapping test (right hand), and ON state timed tapping test (both hands). Visits are Screening Visit 2, Weeks 0, 40/e0, 48/e8, 56/e16, 64/e24, 72/e32 and 80/e40 and all Pilot and Supplemental Extension visits. Baseline is Week 0. Abbreviate as necessary. For all listings by visit, use actual visit labels and not "screening" or "baseline."

Listing 17.2.2.5 NMSS Scores by Visit - ITT Overall Population

Programming Note: Repeat listing as above (17.2.2.x). Repeat listing for ITT Pilot Stage (N=XXX) Placebo/GDNF (N=XXX), then ITT Primary Stage (N=XXX) GDNF/GDNF (N=XXX) and Placebo/GDNF (N=XXX). Sort by stage, treatment group, and subject ID then parameter, and visit. Include all ITT Overall subjects with data. NMSS parameters are Cardiovascular including falls domain, Sleep/fatigue domain, Mood/cognition domain, Perceptual problems/hallucinations domain; Attention/memory domain, Gastrointestinal tract domain, Urinary domain, Sexual function domain, Miscellaneous domain, and NMSS total score. Visits are Screening Visit 2, Weeks 40/e0, 52/e12, 64/e24 and 80/e40 and all Pilot Extension visits. *Abbreviate as necessary. Add footnote "Note: The NMSS is a 30-item interview-based scale that rates non-motor symptoms that occurred in the preceding month in 9 domains. Each item is rated from 0 (none) to 3 (severe) for severity and from 1 (rarely) to 4 (very frequent) for frequency. The maximum score for an individual item is 12. The higher the score, the worse the subject's condition. The maximum NMSS total score is 360." Do not list imputed values. For all listings by visit, use actual visit labels and not "screening" or "baseline."*

Listing 17.2.2.6 PDQ-39 Scores by Visit - ITT Overall Population

Programming Note: Repeat listing as above (17.2.2.x). *Repeat listing for ITT Pilot Stage (N=XXX) Placebo/GDNF (N=XXX), then ITT Primary Stage (N=XXX) GDNF/GDNF (N=XXX) and Placebo/GDNF (N=XXX). Sort by stage, treatment group, and subject ID then parameter, and visit. Include all ITT Overall subjects with data. PDQ-39 parameters are Mobility dimension, ADL dimension, Emotional well-being dimension, Stigma dimension, Social support dimension, Cognitions dimension, Communication dimension, Bodily discomfort dimension, and the Single index (total) PDQ-39 score. Visits are Screening Visit 2, Week 40/e0, and Week 80/e40. Abbreviate as necessary. Add note: The PDQ-39 is a self-administered 39-item PD-specific scale that rates symptoms that occurred in the preceding month in 8 dimensions. Each item is rated from 0 (never) to 4 (always) for frequency. The score for each dimension is the average of responses in the dimension, weighted by the number of questions and multiplied by 100. PDQ-39 dimension and total scores range from 0 to 100 (0 = no problem, 100 = problem as worse as possible). The higher the score, the worse the subject's condition. For all listings by visit, use actual visit labels and not "screening" or "baseline."*

Listing 17.2.2.7 EQ-5D Scores by Visit - ITT Overall Population

Programming Note: Repeat listing as above (17.2.2.x). *Repeat listing for ITT Pilot Stage (N=XXX) Placebo/GDNF (N=XXX), then ITT Primary Stage (N=XXX) GDNF/GDNF (N=XXX) and Placebo/GDNF (N=XXX). Sort by stage, treatment group, and subject ID then parameter, and visit. Include all ITT Overall subjects with data. Parameters are the individual item scores and the visual analog scale score. Visits are Screening Visit 2, Week 40/e0, and Week 80/e40. Abbreviate as necessary. Add note: The EQ-5D is a self-administered 5-item scale with three possible answers for each item (1=no problem, 2=moderate problem, 3=severe problem), and a visual analog scale ranging from 0 to 100, where 0 indicates worst health and 100 indicates best health. Change from baseline only applies to the visual analog scale. For all listings by visit, use actual visit labels and not "screening" or "baseline."*

Listing 17.2.2.8 SNAQ Scores by Visit - ITT Overall Population

Programming Note: Repeat listing as above (17.2.2.x). *Repeat listing for ITT Pilot Stage (N=XXX) Placebo/GDNF (N=XXX), then ITT Primary Stage (N=XXX) GDNF/GDNF (N=XXX) and Placebo/GDNF (N=XXX). Sort by stage, treatment group, and subject ID then parameter, and visit. Include all ITT Overall subjects with data. Parameters are total SNAQ score. Visits are Screening Visit 2, Week 40/e0, and Week 80/e40. Abbreviate as necessary. Add note: The SNAQ is a self-administered 4-question instrument with total scores ranging from 4 to 20 (4=poor appetite, 20=good appetite). For all listings by visit, use actual visit labels and not "screening" or "baseline."*

Listing 17.2.2.9.1 Levodopa and Levodopa Equivalent Medication Actual Total Daily Doses - ITT Overall Population

ITT Pilot Stage (N=XXX)

GDNF/GDNF (N=XXX)

			**Actual Total Daily Dose (mg)**		
**Subject ID Age/Race/Ethnicity/ Sex**	**Category**	**Medication**	**Baseline (Week 0)**	**Week 40/e0**	**Week 80/e40**
xxxxxxxxxx/xx /x/x/x	Levodopa preparations	Immediate release preparations without COMT inhibition		xx	xx
		Immediate release preparations with entacapone			
		Immediate release preparations with tolcapone			
		Controlled release preparations			
		Levodopa/carbidopa (Duodopa)			
	Dopamine agonists	Ropinirole immediate release			
		Ropinirole long acting			
		Pramipexole immediate release (base)			
		Pramipexole immediate release (salt)			
		Pramipexole long acting (base)			
		Pramipexole long acting (salt)			
		Cabergoline			
		Rotigotine			
		Piribedil			
		Apomorphine			
		Bromocriptine			
		Pergolide			
		Lisuride			
		Dihydroergocryptine (DHEC)			
	COMT inhibitors	Entacapone			
		Tolcapone			
	MAO-B inhibitors	Selegiline oral			
		Selegiline sublingual			
		Rasagiline			
	Other	Amantadine			
		Other: xxxxxxxx			

Note: W = white; B = black; P = native Hawaiian or other Pacific islander; A = Asian; I = American Indian or Alaska native; H = Hispanic or Latino; N = not Hispanic or Latino; M = male; F = female.
Source: Dataset: [NAME], Program: xxxxxx.sas, Output: xxxx.rtf, Generated on: DDMONYYYY HH:MM
Page x of y

Programming Note: Repeat listing for ITT Pilot Stage (N=XXX) Placebo/GDNF (N=XXX), then ITT Primary Stage (N=XXX) GDNF/GDNF (N=XXX) and Placebo/GDNF (N=XXX). Sort by stage, treatment group, subject ID and category. Include all ITT Overall subjects with data. ***Actual dose means as documented on the CRF (ie, unconverted). Include only those categories and medications with data.***

Listing 17.2.2.9.2 Effective Levodopa and Levodopa Equivalent Medication Total Daily Doses - ITT Overall Population

ITT Pilot Stage (N=XXX)

GDNF/GDNF (N=XXX)

Subject ID Age/Race/Ethnicity/ Sex	Medication	Total Daily Dose (mg) Baseline (Week 0)	Week 40/e0	Week 80/e40
xxxxxxxxxx/xx /x/x/x	Effective Levodopa		xx	xx
	Levodopa Equivalent		xx	xx
xxxxxxxxxx/xx /x/x/x	Effective Levodopa		xx	xx
	Levodopa Equivalent		xx	xx

Note: W = white; B = black; P = native Hawaiian or other Pacific islander; A = Asian; I = American Indian or Alaska native; H = Hispanic or Latino; N = not Hispanic or Latino; M = male; F = female.

Note: Specific conversion factors are used in order to characterize the subject’s effective levodopa dose. Immediate release preparations taken without concomitant catechol-O-methyl transferase (COMT) inhibitors do not require conversion. The daily doses of immediate release preparations taken with COMT inhibitors and of controlled release preparations are multiplied by the corresponding conversion factors. The total daily levodopa dose is then calculated by adding together the converted daily doses of all individual levodopa-containing preparations. Levodopa equivalent dose is calculated by multiplying each PD medication dose by a specific conversion factor indicating the drug’s relative potency with respect to immediate release levodopa unaccompanied by COMT inhibitors. The total daily levodopa equivalent dose is calculated by adding together the daily levodopa equivalent doses of all individual PD medications. COMT inhibitor doses are not included in either of these calculations.

Source: Dataset: [NAME], Program: xxxxxx.sas, Output: xxxx.rtf, Generated on: DDMONYYYY HH:MM
Page x of y

Programming Note: Repeat listing for ITT Pilot Stage (N=XXX) Placebo/GDNF (N=XXX), then ITT Primary Stage (N=XXX) GDNF/GDNF (N=XXX) and Placebo/GDNF (N=XXX). Sort by stage, treatment group, subject ID and category. Include all ITT Overall subjects with data. ***Total daily doses are the converted doses.***

17.2.3 Imaging Listings

Listing 17.2.3.1 Volume of Distribution, Volume of Interest Coverage, and Total Putamenal Coverage as Determined by Contrast-Enhanced T1-Weighted MRI by Visit - ITT Overall Population

Programming Note: Repeat listing as above (17.2.2.x)*. Sort by stage, treatment group and subject ID then parameter and visit. Include all ITT Overall subjects with data. Parameters are volume of distribution (mL, left and right), VOI (mL, left and right), VOI coverage (mL, left and right; %, left and right), putamenal volume of distribution (mL, left and right), total volume of putamen (mL, left and right), total putamenal coverage (%, left and right). Visits are last test infusion at the end of the healing phase, Week 40/e0, and Week 80/e40. Abbreviate as necessary. For all listings by visit, use actual visit labels and not "screening" or "baseline."*

17.2.4 Safety Listings

Listing 17.2.4.1.1 Exposure to Study Medication - Safety Overall Population

Safety Pilot Stage (N=XXX)		
GDNF/GDNF (N=XXX)		
**Subject ID Age/Race/Ethnicity/ Sex**	**No. of Infusions of Study Medication**	**Total Exposure (mg)**
xxxxxxxxxx/xx/x/x/x	xx	xxxx.x

Note: W = white; B = black; P = native Hawaiian or other Pacific islander; A = Asian; I = American Indian or Alaska native; H = Hispanic or Latino; N = not Hispanic or Latino; M = male; F = female.
Source: Dataset: [NAME], Program: xxxxxx.sas, Output: xxxx.rtf, Generated on: DDMONYYYY HH:MM
Page x of y

Programming Note: Repeat listing for Safety Pilot Stage (N=XXX) Placebo/GDNF (N=XXX), then Safety Primary Stage (N=XXX) GDNF/GDNF (N=XXX) and Placebo/GDNF (N=XXX). Sort by stage, treatment group, and subject ID. Include all Safety Overall subjects. Include only infusions from Study 2797.

Listing 17.2.4.1.2 Study Medication Infusion Data by Visit - Safety Overall Population

Safety Pilot Stage (N=XXX)

GDNF/GDNF (N=XXX)

Subject ID Age/Race/Ethnicity/ Sex	Visit	Date	Time First Start Time Last Stop	Catheter 1	Catheter 2	Catheter 3	Catheter 4
xxxxxxxxxx/xx/x/x/x	Week x	DDMONYYYY	HH:MM HH:MM	Standard	Standard	Standard	Not Used
	Week x	DDMONYYYY	HH:MM HH:MM	Non-standard	Standard	Standard	Not Used

Note: W = white; B = black; P = native Hawaiian or other Pacific islander; A = Asian; I = American Indian or Alaska native; H = Hispanic or Latino; N = not Hispanic or Latino; M = male; F = female.
Note: Standard regime is a linear ramp up infusion rate of 3-5 μL/min with 400 μL total volume.

Source: Dataset: [NAME], Program: xxxxxx.sas, Output: xxxx.rtf, Generated on: DDMONYYYY HH:MM
Page x of y

Programming Note: Repeat listing for Safety Pilot Stage (N=XXX) Placebo/GDNF (N=XXX), then Safety Primary Stage (N=XXX) GDNF/GDNF (N=XXX) and Placebo/GDNF (N=XXX). Sort by stage, treatment group, and subject ID then visit for all visits in Study 2797. Include all Safety Overall subjects. For all listings by visit, use actual visit labels and not "screening" or "baseline."

Listing 17.2.4.1.3 Study Medication Infusion Catheter Interruptions/Early Terminations by Visit - Safety Overall Population

Safety Pilot Stage (N=XXX)

GDNF/GDNF (N=XXX)

Subject ID Age/Race/Ethnicity/ Sex	Visit	Date	Time First Start Time Last Stop	Catheter No. Interrupted/ Terminated Early	Time Infusion Stopped	Time Infusion Restarted	Reason for Stop
xxxxxxxxxx/xx/x/x/x	Week x	DDMONYYYY	HH:MM	1	HH:MM	HH:MM	
			HH:MM	2	HH:MM	HH:MM	
				3	HH:MM	HH:MM	
				4	HH:MM	HH:MM	xxxxxx
	Week x	DDMONYYYY	HH:MM	1	HH:MM	HH:MM	
			HH:MM	2	HH:MM	HH:MM	
				3	HH:MM	HH:MM	
				4	HH:MM	HH:MM	xxxxxx

Note: W = white; B = black; P = native Hawaiian or other Pacific islander; A = Asian; I = American Indian or Alaska native; H = Hispanic or Latino; N = not Hispanic or Latino; M = male; F = female.
Source: Dataset: [NAME], Program: xxxxxx.sas, Output: xxxx.rtf, Generated on: DDMONYYYY HH:MM
Page x of y

Programming Note: Repeat listing for Safety Pilot Stage (N=XXX) Placebo/GDNF (N=XXX), then Safety Primary Stage (N=XXX) GDNF/GDNF (N=XXX) and Placebo/GDNF (N=XXX). Sort by stage, treatment group, and subject ID then visit for all visits in Study 2797. Include all Safety Overall subjects. For all listings by visit, use actual visit labels and not “screening” or “baseline.”

Listing 17.2.4.2 Adverse Events - Safety Overall Population

Safety Pilot Stage (N=XXX)

GDNF/GDNF (N=XXX)

Subject ID Age/Race/Ethnicity Sex	MedDRA Preferred Term VERBATIM TERM	Start Date and Time (Day)/ Stop Date and Time (Day) or Ongoing	Pre-existing?	SAE	Severity	Related to Study Drug?	Related to Device?	Out-come[a]	Ac-tion[b]
xxxxxxxxxx/xx/x/x/x	xxxxxxxxxxx xxxxxxxxxxx	DDMONYYYY HHMM (xxx)/ DDMONYYYY HHMM (xxx)	No	Yes	Mild	No	Yes	xx	99: xxxxxx

Note: Adverse events are coded using MedDRA version 19.0. W = white; B = black; P = native Hawaiian or other Pacific islander; A = Asian; I = American Indian or Alaska native; H = Hispanic or Latino; N = not Hispanic or Latino; M = male; F = female.

a Outcome: 1 = Recovered/resolved; 2 = Not recovered/not resolved; 3 = Recovered/resolved with sequelae; 4 = Fatal.

b Action: 1 = Current infusion interrupted and restarted; 2 = Current infusion terminated; 3 = Infusion protocol modified; 4 = Infusion schedule suspended and resumed; 5 = Treatment discontinued permanently; 6 = Surgical revision/replacement of extracerebral device parts; 7 = Surgical repositioning/replacement of intracerebral device parts; 99 = Other.

Source: Dataset: [NAME], Program: xxxxxx.sas, Output: xxxx.rtf, Generated on: DDMONYYYY HH:MM

Page x of y

Programming Note: Repeat listing for Safety Pilot Stage (N=XXX) Placebo/GDNF (N=XXX), then Safety Primary Stage (N=XXX) GDNF/GDNF (N=XXX) and Placebo/GDNF (N=XXX). Sort by stage, treatment group, subject ID, start date and time. Include all Safety Overall subjects with data.

Listing 17.2.4.3.1 Adverse Events of Special Interest: Dyskinesias- Safety Overall Population

Programming Note: Repeat AE listing above. Repeat listing for Safety Pilot Stage (N=XXX) Placebo/GDNF (N=XXX), then Safety Primary Stage (N=XXX) GDNF/GDNF (N=XXX) and Placebo/GDNF (N=XXX). Sort by stage, treatment group, subject ID, start date and time. Include all Safety Overall subjects with data.

Listing 17.2.4.3.2 Adverse Events of Special Interest: Falls- Safety Overall Population

Repeat Listing 17.2.4.3.1

Listing 17.2.4.3.3 Adverse Events of Special Interest: Adverse Changes in Mood- Safety Overall Population

Repeat Listing 17.2.4.3.1

Listing 17.2.4.3.4 Adverse Events of Special Interest: Impulsivity- Safety Overall Population

Repeat Listing 17.2.4.3.1

Listing 17.2.4.4 Port Symptoms by Visit - Safety Overall Population

Safety Pilot Stage (N=XXX)

GDNF/GDNF (N=XXX)

Subject ID<br>Age/Race/Ethnicity<br>Sex	Visit	Date Performed	Result
xxxxxxxxxx/xx/x/x/x	Week 40/e0<br>…	DDMONYYYY<br>….	No skin reaction<br>Redness with slight swelling<br>Redness, moistness and moderate swelling with tissue granulation<br>Overt infection

Note: W = white; B = black; P = native Hawaiian or other Pacific islander; A = Asian; I = American Indian or Alaska native; H = Hispanic or Latino; N = not Hispanic or Latino; M = male; F = female.
Source: Dataset: [NAME], Program: xxxxxx.sas, Output: xxxx.rtf, Generated on: DDMONYYYY HH:MM
Page x of y

Programming Note: Repeat listing for Safety Pilot Stage (N=XXX) Placebo/GDNF (N=XXX), then Safety Primary Stage (N=XXX) GDNF/GDNF (N=XXX) and Placebo/GDNF (N=XXX). Sort by stage, treatment group, and subject ID then visit for all visits in Study 2797. Include all Safety Overall subjects with data.

Listing 17.2.4.5 Hematology Results by Visit - Safety Overall Population

Safety Pilot Stage (N=XXX)

GDNF/GDNF (N=XXX)

Subject ID Age/Race/Ethnicity/ Sex	Parameter (Unit)	Visit	Sample Date	Result	Change from Baseline	Normal Range	Flag	Clin Sig?
xxxxxxxxxx/xx/x/x/x	xxxxxxxxxxxxx	Week 40/e0	DDMONYYYY	xxx.xx		xxx.xx – xxx.xx	H	No
		Week X	DDMONYYYY	xxx.xx	xx.xx	xxx.xx – xxx.xx	H	No
		…	…	…	…	…	…	…

Note: W = white; B = black; P = native Hawaiian or other Pacific islander; A = Asian; I = American Indian or Alaska native; H = Hispanic or Latino; N = not Hispanic or Latino; M = male; F = female; H = high; L = low.
Source: Dataset: [NAME], Program: xxxxxx.sas, Output: xxxx.rtf, Generated on: DDMONYYYY HH:MM
Page x of y

*Programming Note: Repeat listing for Safety Pilot Stage (N=XXX) Placebo/*GDNF *(N=XXX), then Safety Primary Stage (N=XXX) GDNF/*GDNF *(N=XXX) and Placebo/*GDNF *(N=XXX). Sort by stage, treatment group, and subject ID then parameter (order as shown in SAP Table 5), and visit for Week 40/e0 and all further visits in Study 2797. Include all Safety Overall subjects with data. For all listings by visit, use actual visit labels and not "screening" or "baseline."*

Listing 17.2.4.6 Serum Chemistry Results by Visit - Safety Overall Population

*Programming Note: Repeat listing for serum chemistry parameters. Repeat listing for Safety Pilot Stage (N=XXX) Placebo/*GDNF *(N=XXX), then Safety Primary Stage (N=XXX) GDNF/*GDNF *(N=XXX) and Placebo/*GDNF *(N=XXX). Sort by stage, treatment group, and subject ID then parameter (order as shown in SAP Table 5), and visit for Week 40/e0 and all further visits in Study 2797. Include all Safety Overall subjects with data. For all listings by visit, use actual visit labels and not "screening" or "baseline."*

Listing 17.2.4.7 Urinalysis Results by Visit - Safety Overall Population

*Programming Note: Repeat listing for urinalysis parameters. Repeat listing for Safety Pilot Stage (N=XXX) Placebo/*GDNF *(N=XXX), then Safety Primary Stage (N=XXX) GDNF/*GDNF *(N=XXX) and Placebo/*GDNF *(N=XXX). Sort by stage, treatment group, and subject ID then parameter (order as shown in SAP Table 5), and visit for Week 40/e0 and all further visits in Study 2797. Include all Safety Overall subjects with data. For all listings by visit, use actual visit labels and not "screening" or "baseline."*

Listing 17.2.4.8 Anti-GDNF Serum Antibodies - Safety Overall Population

Safety Pilot Stage (N=XXX)				
GDNF/GDNF (N=XXX)				
**Subject ID Age/Race/Ethnicity/ Sex**	**Parameter**	**Visit**	**Sample Date**	**Result**
xxxxxxxxxx/xx/x/x/x	Anti-GDNF Binding Serum Antibody	Screening Visit 1<br>…	DDMONYYYY	Negative
	Anti-GDNF Neutralizing Serum Antibody	…		Positive

Note: W = white; B = black; P = native Hawaiian or other Pacific islander; A = Asian; I = American Indian or Alaska native; H = Hispanic or Latino; N = not Hispanic or Latino; M = male; F = female.
Source: Dataset: [NAME], Program: xxxxxx.sas, Output: xxxx.rtf, Generated on: DDMONYYYY HH:MM
Page x of y

Programming Note: Repeat listing for Safety Pilot Stage (N=XXX) Placebo/GDNF (N=XXX), then Safety Primary Stage (N=XXX) GDNF/GDNF (N=XXX) and Placebo/GDNF (N=XXX). Sort by stage, treatment group, and subject ID then parameter and visit for screening from Study 2553and all postbaseline visits from Study 2553 and Study 2797. Include all Safety Overall subjects with data. Abbreviate as necessary. For all listings by visit, use actual visit labels and not "screening" or "baseline."

Listing 17.2.4.9 Plasma GDNF Concentration Results by Visit - Safety Overall Population

Safety Pilot Stage (N=XXX)

GDNF/GDNF (N=XXX)

Subject ID Age/Race/Ethnicity/ Sex	Visit	Sample Date	Plasma GDNF Concentration (Unit)	Change from Baseline
xxxxxxxxxx/xx/x/x/x	Screening Visit 1	DDMONYYYY	xxx.xx	
	Week 40/e0	DDMONYYYY	xxx.xx	xx.xx
	....			

Note: W = white; B = black; P = native Hawaiian or other Pacific islander; A = Asian; I = American Indian or Alaska native; H = Hispanic or Latino; N = not Hispanic or Latino; M = male; F = female.
Source: Dataset: [NAME], Program: xxxxxx.sas, Output: xxxx.rtf, Generated on: DDMONYYYY HH:MM
Page x of y

Programming Note: Repeat listing for Safety Pilot Stage (N=XXX) Placebo/GDNF (N=XXX), then Safety Primary Stage (N=XXX) GDNF/GDNF (N=XXX) and Placebo/GDNF (N=XXX). Sort by stage, treatment group, and subject ID then parameter, and visit for screening from Study 2553, Week 40/e0, and all further visits in Study 2797. Include all Safety Overall subjects with data. For all listings by visit, use actual visit labels and not "screening" or "baseline."

Listing 17.2.4.10 Physical Examination - Safety Overall Population

Safety Pilot Stage (N=XXX)

GDNF/GDNF (N=XXX)

Subject ID Age/Race/Ethnicity/ Sex	Body System	Visit	Date	Normal/ Abnormal/ Not Done	Abnormality
xxxxxxxxxx/xx/x/x/x	Skin	Week 80/e40	DDMONYYYY	xxxxxxxx	xxxxxxxxxxxxxxxxxxxx
		Week e2-80	DDMONYYYY	xxxxxxxx	xxxxxxxxxxxxxxxxxxxx
	Head, Ears, Eyes, Nose, and Throat	Week 80/e40	DDMONYYYY	xxxxxxxx	xxxxxxxxxxxxxxxxxxxx
		Week e2-80	DDMONYYYY	xxxxxxxx	xxxxxxxxxxxxxxxxxxxx
	Respiratory	Week 80/e40	DDMONYYYY	xxxxxxxx	xxxxxxxxxxxxxxxxxxxx
		Week e2-80	DDMONYYYY	xxxxxxxx	xxxxxxxxxxxxxxxxxxxx
	Cardiovascular	Week 80/e40	DDMONYYYY	xxxxxxxx	xxxxxxxxxxxxxxxxxxxx
		Week e2-80	DDMONYYYY	xxxxxxxx	xxxxxxxxxxxxxxxxxxxx
	Abdomen (incl. liver and kidneys)	Week 80/e40	DDMONYYYY	xxxxxxxx	xxxxxxxxxxxxxxxxxxxx
		Week e2-80	DDMONYYYY	xxxxxxxx	xxxxxxxxxxxxxxxxxxxx
	Musculoskeletal	Week 80/e40	DDMONYYYY	xxxxxxxx	xxxxxxxxxxxxxxxxxxxx
		Week e2-80	DDMONYYYY	xxxxxxxx	xxxxxxxxxxxxxxxxxxxx
	Neurological	Week 80/e40	DDMONYYYY	xxxxxxxx	xxxxxxxxxxxxxxxxxxxx
		Week e2-80	DDMONYYYY	xxxxxxxx	xxxxxxxxxxxxxxxxxxxx
	Gastrointestinal	Week 80/e40	DDMONYYYY	xxxxxxxx	xxxxxxxxxxxxxxxxxxxx
		Week e2-80	DDMONYYYY	xxxxxxxx	xxxxxxxxxxxxxxxxxxxx
	Genitourinary	Week 80/e40	DDMONYYYY	xxxxxxxx	xxxxxxxxxxxxxxxxxxxx
		Week e2-80	DDMONYYYY	xxxxxxxx	xxxxxxxxxxxxxxxxxxxx
	Endocrine	Week 80/e40	DDMONYYYY	xxxxxxxx	xxxxxxxxxxxxxxxxxxxx
		Week e2-80	DDMONYYYY	xxxxxxxx	xxxxxxxxxxxxxxxxxxxx
	Lymph nodes	Week 80/e40	DDMONYYYY	xxxxxxxx	xxxxxxxxxxxxxxxxxxxx
		Week e2-80	DDMONYYYY	xxxxxxxx	xxxxxxxxxxxxxxxxxxxx
	Other	Week 80/e40	DDMONYYYY	xxxxxxxx	xxxxxxxxxxxxxxxxxxxx
		Week e2-80	DDMONYYYY	xxxxxxxx	xxxxxxxxxxxxxxxxxxxx

Note: W = white; B = black; P = native Hawaiian or other Pacific islander; A = Asian; I = American Indian or Alaska native; H = Hispanic or Latino; N = not Hispanic or Latino; M = male; F = female.
Source: Dataset: [NAME], Program: xxxxxx.sas, Output: xxxx.rtf, Generated on: DDMONYYYY HH:MM
Page x of y

*Programming Note: Repeat listing for Safety Pilot Stage (N=XXX) Placebo/*GDNF *(N=XXX), then Safety Primary Stage (N=XXX) GDNF/*GDNF *(N=XXX) and Placebo/*GDNF *(N=XXX). Sort by stage, treatment group, and subject ID then body system and visit. Include all Safety Overall subjects with data. Visits are Week 80/e40 and e2-80. Abbreviate as necessary. For all listings by visit, use actual visit labels and not "screening" or "baseline."*

Listing 17.2.4.11 Vital Signs from Infusions with a Clinically Revelant Postbaseline Abnormal Result by Visit and Time Point - Safety Overall Population

Subject ID Age/Race/Ethnicity/ Sex	Parameter (Unit)	Visit	Date	Time Point	Result	Change from Baseline[a]	Clinically Relevant? If Y, then criteria
			Safety Pilot Stage (N=XXX)				
			GDNF/GDNF (N=XXX)				
xxxxxxxxxx/xx/x/x/x	xxxxxxxxxxxxx	Week e0	DDMONYYYY		xxx.xx		
		…					
		Week x	DDMONYYYY	Pre-infusion	xxx.xx		
				15 min after infusion start	xxx.xx	xx.xx	N
				…	…	…	Y/ < 50 bpm

[a] For test infusion and study medication infusion visits, change from baseline refers to change from pre-infusion value.

Note: W = white; B = black; P = native Hawaiian or other Pacific islander; A = Asian; I = American Indian or Alaska native; H = Hispanic or Latino; N = not Hispanic or Latino; M = male; F = female.

Note: All values for a given parameter with a clinically relevant abnormal result at a particular visit are listed.

Source: Dataset: [NAME], Program: xxxxxx.sas, Output: xxxx.rtf, Generated on: DDMONYYYY HH:MM

Page x of y

Programming Note: Repeat listing for Safety Pilot Stage (N=XXX) Placebo/GDNF (N=XXX), then Safety Primary Stage (N=XXX) GDNF/GDNF (N=XXX) and Placebo/GDNF (N=XXX). Sort by stage, treatment group, and subject ID then parameter, visit, and time point. Include all Safety Overall subjects with data. Parameters are pulse (sitting and standing; beats/min), respiration (breaths/min), systolic and diastolic blood pressure (BP; sitting and standing; mmHg), and temperature (℃). Data are presented for all test infusion visits (interim visits after catheter repositioning and Week 80/e40 visits) and study medication infusion visits in the Initial Extension, including pre-infusion value, 15 min after infusion start, 30 min after infusion start, 45 min after infusion start, 60 min after infusion start, 75 min after infusion start, 90 min after infusion start, 105 min after infusion start, 120 min after infusion start, and post-dose, where applicable. If clinically relevant, then concatenate the criteria listed in SAP Table 6. For all listings by visit, use actual visit labels and not "screening" or "baseline."

Listing 17.2.4.12 Weight (kg) by Visit - Safety Overall Population

*Programming Note: Repeat UPDRS Listing 17.2.2.1 omitting the parameter column. Repeat listing for Safety Pilot Stage (N=XXX) Placebo/*GDNF *(N=XXX), then Safety Primary Stage (N=XXX) GDNF/*GDNF *(N=XXX) and Placebo/*GDNF *(N=XXX). Sort by stage, treatment group, and subject ID then parameter and visit for Week e0 and all further visits in Study 2797. Include all Safety Overall subjects with data. Abbreviate as necessary. For all listings by visit, use actual visit labels and not "screening" or "baseline."*

Listing 17.2.4.13 Electrocardiogram Results by Visit – Continuous Parameters, Safety Overall Population

*Programming Note: Repeat VS Listing 17.2.4.12 (without column for time points). Repeat listing for Safety Pilot Stage (N=XXX) Placebo/*GDNF *(N=XXX), then Safety Primary Stage (N=XXX) GDNF/*GDNF *(N=XXX) and Placebo/*GDNF *(N=XXX). Sort by stage, treatment group, and subject ID then parameter and visit for screening, Week 40/e0, and all further visits in Study 2797. Include all Safety Overall subjects with data. Parameters are heart rate (beats/min), PR interval (ms), QRS interval (ms), QT interval (ms), and QTc interval (ms). Abbreviate as necessary. If clinically relevant, then concatenate the criteria listed in SAP Table 7. For all listings by visit, use actual visit labels and not "screening" or "baseline." Add the following footnote:*
Note: Hodges QT correction formula was used.

Listing 17.2.4.14 Electrocardiogram Results by Visit – Overall Impression, Safety Overall Population

Safety Pilot Stage (N=XXX)

GDNF/GDNF (N=XXX)

Subject ID Age/Race/Ethnicity/Sex	Visit	Date	Overall Impression (Normal or Abnormal?)	Clinically Significant?	Comment
xxxxxxxxxx/xx/x/x/x	Screening Visit 1	DDMONYYYY	Abnormal	Yes	xxxxxxxxxxxxxxxxxxxxxxx
	Week 40/e0	DDMONYYYY	Normal		
	...				

Note: W = white; B = black; P = native Hawaiian or other Pacific islander; A = Asian; I = American Indian or Alaska native; H = Hispanic or Latino; N = not Hispanic or Latino; M = male; F = female.
Source: Dataset: [NAME], Program: xxxxxx.sas, Output: xxxx.rtf, Generated on: DDMONYYYY HH:MM
Page x of y

*Programming Note: Repeat listing for Safety Pilot Stage (N=XXX) Placebo/*GDNF *(N=XXX), then Safety Primary Stage (N=XXX) GDNF/*GDNF *(N=XXX) and Placebo/*GDNF *(N=XXX). Sort by stage, treatment group, and subject ID then visit for screening, Week 40/e0, and all further visits in Study 2797. Include all Safety Overall subjects with data. Abbreviate as necessary. For all listings by visit, use actual visit labels and not "screening" or "baseline."*

Listing 17.2.4.15 Glasgow Coma Scale Results < 15 by Visit - Safety Overall Population

Safety Pilot Stage (N=XXX)

GDNF/GDNF (N=XXX)

Subject ID Age/Race/ Ethnicity/Sex	Parameter	Visit	Date and Time	Time Point	Result
xxxxxxxxxx/xx/x/x/x	Visual Response	Week e0	DDMONYYYY HHMM	Pre-infusion	x
			DDMONYYYY HHMM	30 min after infusion start	x
			DDMONYYYY HHMM	Post-infusion	x
	Verbal Ability		DDMONYYYY HHMM	Pre-infusion	x
			DDMONYYYY HHMM	30 min after infusion start	x
		…	…	…	…

Note: Glasgow Coma Scale items include visual response, verbal ability, and motor skills. Each item has 4-6 possible responses. The best possible total score is 15. W = white; B = black; P = native Hawaiian or other Pacific islander; A = Asian; I = American Indian or Alaska native; H = Hispanic or Latino; N = not Hispanic or Latino; M = male; F = female.

Source: Dataset: [NAME], Program: xxxxxx.sas, Output: xxxx.rtf, Generated on: DDMONYYYY HH:MM

Page x of y

*Programming Note: Repeat listing for Safety Pilot Stage (N=XXX) Placebo/*GDNF *(N=XXX), then Safety Primary Stage (N=XXX) GDNF/*GDNF *(N=XXX) and Placebo/*GDNF *(N=XXX). Sort by stage, treatment group, and subject ID, then parameter, visit, date, and time point for all visits in Study 2797. Include all Safety Overall subjects with a result <15 for a particular visit or missing in all extension parts of Study 2797 (Initial Extension, Pilot Extension, Supplemental Extension). Abbreviate as necessary. Include the scoring information in SAP Table 8 in an endnote.*

Listing 17.2.4.16 QUIP Items Answered with Yes by Visit - Safety Overall Population

Safety Pilot Stage (N=XXX)

GDNF/GDNF (N=XXX)

Subject ID Age/Race/ Ethnicity/Sex	Reported By	Question	Behavior	Visit	Date
xxxxxxxxxx/xx/x/x/x	Subject	Do you or others think you have an issue with too much gambling, sex, buying, or eating behaviors?	Gambling	Week 40/e0	DDMONYYYY
		…		Week 48/e8	DDMONYYYY
			…		
			Sex	…	DDMONYYYY
			Buying	…	DDMONYYYY
			Eating	…	DDMONYYYY
			…	…	
	Informant	Do you or others think you have an issue with too much gambling, sex, buying, or eating behaviors?	Gambling	Week 40/e0	DDMONYYYY
		…	…	…	…

Note: The QUIP is a self-administered or informant-completed scale that includes 13 questions covering symptoms related to the 4 commonest impulse control disorders in PD, other behaviors, and problematic use of medication. W = white; B = black; P = native Hawaiian or other Pacific islander; A = Asian; I = American Indian or Alaska native; H = Hispanic or Latino; N = not Hispanic or Latino; M = male; F = female.
Source: Dataset: [NAME], Program: xxxxxx.sas, Output: xxxx.rtf, Generated on: DDMONYYYY HH:MM
Page x of y

*Programming Note: Repeat listing for Safety Pilot Stage (N=XXX) Placebo/*GDNF *(N=XXX), then Safety Primary Stage (N=XXX) GDNF/*GDNF *(N=XXX) and Placebo/*GDNF *(N=XXX). Sort by stage, treatment group, and subject ID, then reported by, question, behavior, visit, and date for Week 40/e0 and all further visits in Study 2797. Include all Safety Overall subjects with data. Do not list imputed values. Note that behavior column is not applicable for "Other Behaviors"*

(subquestions can go in “Behavior” column) and “Medication Use” (leave “Behavior” column blank) sections. For all listings by visit, use actual visit labels and not “screening” or “baseline.”

Listing 17.2.4.17 MoCA Total Score by Visit - Safety Overall Population

Safety Pilot Stage (N=XXX)

GDNF/GDNF (N=XXX)

Subject ID Age/Race/ Ethnicity/Sex	Visit	Date	Result
xxxxxxxxxx/xx/x/x/x	Screening Visit 1	DDMONYYYY	x
	Pre-test infusion	DDMONYYYY	x
	Week 40/e0	DDMONYYYY	x
		DDMONYYYY	x
		DDMONYYYY	x
	…	…	…

Note: The MoCA is a rater-administered cognitive screening tool with 8 components. The total score ranges from 0 to 30, with lower scores representing poorer cognitive function. A total score of 26 or above is considered normal. W = white; B = black; P = native Hawaiian or other Pacific islander; A = Asian; I = American Indian or Alaska native; H = Hispanic or Latino; N = not Hispanic or Latino; M = male; F = female.
Source: Dataset: [NAME], Program: xxxxxx.sas, Output: xxxx.rtf, Generated on: DDMONYYYY HH:MM
Page x of y

*Programming Note: Repeat listing for Safety Pilot Stage (N=XXX) Placebo/*GDNF *(N=XXX), then Safety Primary Stage (N=XXX) GDNF/*GDNF *(N=XXX) and Placebo/*GDNF *(N=XXX). Sort by stage, treatment group, and subject ID, then visit and date for screening, pre-infusion, Week 40/e0, and all further visits in Study 2797. Include all Safety Overall subjects with data. Do not list imputed values. For all listings by visit, use actual visit labels and not "screening" or "baseline."*

Listing 17.2.4.18 MDRS AEMSS Total Score by Visit - Safety Overall Population

Safety Pilot Stage (N=XXX)

GDNF/GDNF (N=XXX)

Subject ID Age/Race/ Ethnicity/Sex	Visit	Date	AEMSS Total Score
xxxxxxxxxx/xx/x/x/x	Screening Visit 1	DDMONYYYY	x
	Week 40/e0	DDMONYYYY	x
		DDMONYYYY	x
		DDMONYYYY	x
		DDMONYYYY	x
	…	…	x
			xxx

Note: The MDRS is a rater-administered global scale of cognition including 5 subscales. The total score ranges from 0 to 144, with higher scores representing better cognitive function. A total score lower than 123 is associated with some degree of dementia in PD. W = white; B = black; P = native Hawaiian or other Pacific islander; A = Asian; I = American Indian or Alaska native; H = Hispanic or Latino; N = not Hispanic or Latino; M = male; F = female.
Source: Dataset: [NAME], Program: xxxxxx.sas, Output: xxxx.rtf, Generated on: DDMONYYYY HH:MM
Page x of y

Programming Note: Repeat listing for Safety Pilot Stage (N=XXX) Placebo/GDNF *(N=XXX), then Safety Primary Stage (N=XXX) GDNF*/GDNF *(N=XXX) and Placebo*/GDNF *(N=XXX). Sort by stage, treatment group, and subject ID, then visit and date for screening, Week 40/e0, and all further visits in Study 2797. Include all Safety Overall subjects with data. Do not list imputed values. For all listings by visit, use actual visit labels and not "screening" or "baseline."*

Listing 17.2.4.19 Stroop Test by Visit - Safety Overall Population

Safety Pilot Stage (N=XXX)

GDNF/GDNF (N=XXX)

Subject ID Age/Race/ Ethnicity/Sex	Condition	Visit	Date and Time	Total Uncorrected Errors	Total Self-Corrected Errors	Total Time to Complete (secs)
xxxxxxxxxx/xx/x/x/x	Color Naming	Screening Visit 2	DDMONYYYY HHMM	xx	xx	xx
		Week 40/e0	DDMONYYYY HHMM	xx	xx	xx
		Week 80/e40				
	Word Reading		DDMONYYYY HHMM			
	Inhibition		DDMONYYYY HHMM			
	Inhibition/Switching	…	…			

Note: The Stroop test is a global scale of reaction time including 4 conditions. Total time to complete the test in each condition can range from 0 to 999 seconds, with lower time representing better reaction time. W = white; B = black; P = native Hawaiian or other Pacific islander; A = Asian; I = American Indian or Alaska native; H = Hispanic or Latino; N = not Hispanic or Latino; M = male; F = female.

Source: Dataset: [NAME], Program: xxxxxx.sas, Output: xxxx.rtf, Generated on: DDMONYYYY HH:MM

Page x of y

*Programming Note: Repeat listing for Safety Pilot Stage (N=XXX) Placebo/*GDNF *(N=XXX), then Safety Primary Stage (N=XXX) GDNF/*GDNF *(N=XXX) and Placebo/*GDNF *(N=XXX). Sort by stage, treatment group, and subject ID, then condition (CRF order), visit, and date for screening, Week 40/e0, and Week 80/e40. Include all Safety Overall subjects with data. For all listings by visit, use actual visit labels and not "screening" or "baseline."*

Listing 17.2.4.20 FrSBe by Visit - Safety Overall Population

Safety Pilot Stage (N=XXX)

GDNF/GDNF (N=XXX)

Subject ID Age/Race/ Ethnicity/Sex	Scale	Visit	Date	Time Point	Result
xxxxxxxxxx/xx/x/x/x	Apathy Score	Screening Visit 2	DDMONYYYY	Before	x
		Screening Visit 2	DDMONYYYY	After	x
		Week 40/e0	DDMONYYYY	After	x
		Week 80/e40	DDMONYYYY	After	
	Disinhibition	…	…	Before	…
	Disinhibition			After	
	Executive Dysfunction			Before	
	Executive Dysfunction			After	

Note: The FrSBe is a scale that assesses behavior related to frontal systems damage including 3 subscales. Higher subscale scores indicate greater pathology. W = white; B = black; P = native Hawaiian or other Pacific islander; A = Asian; I = American Indian or Alaska native; H = Hispanic or Latino; N = not Hispanic or Latino; M = male; F = female.
Source: Dataset: [NAME], Program: xxxxxx.sas, Output: xxxx.rtf, Generated on: DDMONYYYY HH:MM
Page x of y

*Programming Note: Repeat listing for Safety Pilot Stage (N=XXX) Placebo/*GDNF *(N=XXX), then Safety Primary Stage (N=XXX) GDNF/*GDNF *(N=XXX) and Placebo/*GDNF *(N=XXX). Sort by stage, treatment group, and subject ID, then scale (CRF order), visit, and date for screening, Week 40/e0, and Week 80/e40. Include all Safety Overall subjects with data. Baseline value is "after" at Screening Visit 2; Week 80/e40 data has "after" only. Do not list imputed values. For all listings by visit, use actual visit labels and not "screening" or "baseline."*

Listing 17.2.4.21 Deary-Liewald Four-Choice Reaction Time by Visit - Safety Overall Population

Safety Pilot Stage (N=XXX)

GDNF/GDNF (N=XXX)

Subject ID<br>Age/Race/<br>Ethnicity/Sex	For correct responses for the four-choice RT:<br>Parameter	Visit	Date	Result<br>(ms)
xxxxxxxxxx/xx/x/x/x	Mean reaction time	Screening Visit 2	DDMONYYYY	x
	Variance	Week 40/e0	DDMONYYYY	x
	SD	Week 56/e16	DDMONYYYY	x
		Week 80/e40	DDMONYYYY	x

Note: The Deary-Liewald RT is a computerized measure of simple and four-choice reaction time. The parameter reported is the mean reaction time, variance, and SD for correct responses for four-choice reaction time. A shorter reaction time is better. W = white; B = black; P = native Hawaiian or other Pacific islander; A = Asian; I = American Indian or Alaska native; H = Hispanic or Latino; N = not Hispanic or Latino; M = male; F = female.

Source: Dataset: [NAME], Program: xxxxxx.sas, Output: xxxx.rtf, Generated on: DDMONYYYY HH:MM

Page x of y

Programming Note: Repeat listing for Safety Pilot Stage (N=XXX) Placebo/GDNF *(N=XXX), then Safety Primary Stage (N=XXX) GDNF*/GDNF *(N=XXX) and Placebo*/GDNF *(N=XXX). Sort by stage, treatment group, and subject ID, then parameter, visit, and date for screening, Week 40/e0, Week 56/e16, and Week 80/e40. Include all Safety Overall subjects with data. For all listings by visit, use actual visit labels and not "screening" or "baseline."*

Listing 17.2.4.22 Verbal Fluency Assessment by Visit - Safety Overall Population

Safety Pilot Stage (N=XXX)

GDNF/GDNF (N=XXX)

Subject ID Age/Race/ Ethnicity/Sex	Test	Visit	Date	Number of Correct Responses in 1 Minute
xxxxxxxxxx/xx/x/x/x	Phonemic Verbal Fluency	Screening Visit 2	DDMONYYYY	xx
		Week 40/e0	DDMONYYYY	xx
		Week 80/e40		
	Semantic Verbal Fluency	Screening Visit 2	DDMONYYYY	xx
		Week 40/e0	DDMONYYYY	xx
		Week 80/e40		

Note: The verbal fluency assessment measures verbal functioning in 2 categories. Scores represent number of correct words in one minute and range from 0 to 200. Higher scores represent better verbal functioning. W = white; B = black; P = native Hawaiian or other Pacific islander; A = Asian; I = American Indian or Alaska native; H = Hispanic or Latino; N = not Hispanic or Latino; M = male; F = female.

Source: Dataset: [NAME], Program: xxxxxx.sas, Output: xxxx.rtf, Generated on: DDMONYYYY HH:MM

Page x of y

*Programming Note: Repeat listing for Safety Pilot Stage (N=XXX) Placebo/*GDNF *(N=XXX), then Safety Primary Stage (N=XXX) GDNF/*GDNF *(N=XXX) and Placebo/*GDNF *(N=XXX). Sort by stage, treatment group, and subject ID, then test, visit, and date for screening, Week 40/e0, and Week 80/e40. Include all Safety Overall subjects with data. For all listings by visit, use actual visit labels and not "screening" or "baseline."*

Listing 17.2.4.23 BDI by Visit - Safety Overall Population

Safety Pilot Stage (N=XXX)

GDNF/GDNF (N=XXX)

Subject ID Age/Race/ Ethnicity/Sex	Visit	Date	Total Score
xxxxxxxxxx/xx/x/x/x	Screening Visit 2	DDMONYYYY	xx
	Week 40/e0	DDMONYYYY	
	Week 80/e40	DDMONYYYY	
	Last study visit, Pilot Extension	DDMONYYYY	
	Last study visit, Supplemental Extension	DDMONYYYY	

Note: The BDI is a self-administered test which consists of 21 questions that measure the severity of depression. Scores range from 0 to 63, with higher scores representing worse depression. W = white; B = black; P = native Hawaiian or other Pacific islander; A = Asian; I = American Indian or Alaska native; H = Hispanic or Latino; N = not Hispanic or Latino; M = male; F = female.
Source: Dataset: [NAME], Program: xxxxxx.sas, Output: xxxx.rtf, Generated on: DDMONYYYY HH:MM
Page x of y

Programming Note: Repeat listing for Safety Pilot Stage (N=XXX) Placebo/GDNF *(N=XXX), then Safety Primary Stage (N=XXX) GDNF*/GDNF *(N=XXX) and Placebo*/GDNF *(N=XXX). Sort by stage, treatment group, and subject ID, then visit and date for screening, Week 40/e0, Week 80/e40 and the last study visit in both the Pilot Extension and the Supplemental Extension. Include all Safety Overall subjects with data. For all listings by visit, use actual visit labels and not "screening" or "baseline."*

Listing 17.2.4.24 UPSIT by Visit - Safety Overall Population

Safety Pilot Stage (N=XXX)

GDNF/GDNF (N=XXX)

Subject ID Age/Race/ Ethnicity/Sex	Visit	Date	Total Number of Correct Responses	Percentile Value	Descriptive Term
xxxxxxxxxx/xx/x/x/x					Normosmia/ Mild microsmia/ Moderate microsmia/ Severe microsmia/ Anosmia/ Probable malingering
	Screening Visit 2	DDMONYYYY	xx	xx	
	Week 40/e0	DDMONYYYY	xx	xx	
	Week 80/e40	DDMONYYYY	xx	xx	
	..				

Note: The UPSIT is a self-administered test which can be used to identify and quantitate olfactory dysfunction in PD. The number of correct responses out of 40 total items constitutes a subject's score. Lower scores represent greater olfactory dysfunction. W = white; B = black; P = native Hawaiian or other Pacific islander; A = Asian; I = American Indian or Alaska native; H = Hispanic or Latino; N = not Hispanic or Latino; M = male; F = female.
Source: Dataset: [NAME], Program: xxxxxx.sas, Output: xxxx.rtf, Generated on: DDMONYYYY HH:MM
Page x of y

Programming Note: Repeat listing for Safety Pilot Stage (N=XXX) Placebo/GDNF *(N=XXX), then Safety Primary Stage (N=XXX) GDNF*/GDNF *(N=XXX) and Placebo*/GDNF *(N=XXX). Sort by stage, treatment group, and subject ID, then visit, and date for screening, Week 40/e0, and Week 80/e40. Include all Safety Overall subjects with data. For all listings by visit, use actual visit labels and not "screening" or "baseline."*

Document History

Version Date	Modified/Reviewed By	Brief Summary of changes to current version
29-NOV-2016	Emma Lewis/ Kerri Barber/ Liam Collard/ Lara Longpre/ Matthias Luz	Draft version 0.1. Created from PRS 005 T 17 E.
16-DEC-2016	Emma Lewis/ Lara Longpre/ Matthias Luz	Draft version 0.2.
10-JAN-2017	Emma Lewis/ Lara Longpre/ Matthias Luz	Draft version 0.3.
07-FEB-2017	Emma Lewis/ Lara Longpre/ Matthias Luz	Final version 1.0.
10-APR-2017	Emma Lewis/ Lara Longpre/ Matthias Luz	Final version 2.0.

Statistical Analysis Plan Change Log

Section / Table / Listing	Page	Comment
Figures 16.5.1.1.1, 16.5.1.1.2	126, 127	Add footnote “P-value from a mixed-effect model with repeated measures (MMRM) for the percentage change from baseline to Week 80/e40 between treatment groups is x.xxx.“
Tables 16.3.3.2, 16.3.3.3	101	Add a programming note to say that each region will be on a separate page.
Table 4	27	Change Week 80/e40 study day range for study 2797 to 260-309
Tables 16.4.4.1, 16.4.4.2	115, 116	Add rows for "1 Missing or Not Done", "2 Missing or Not Done" and ">2 Missing or Not Done" to the all visits section. Add a "Missing" row for each individual visit section. Also add the following to the footnote<br>"All negative" includes all subjects without a positive result even if some results are missing or not done.
Table 16.4.5	117	Add "Not Done" and "Missing" rows for each visit. Also add the footnote "Note: Results of <LOD and <LLOQ are not included in the summary statistics."
Table 16.2.3.3.1	87	Add the footnote "Note: For subject 45, items 22, 27, 28, 29 and 30 are excluded from the UPDRS score."
Table 16.2.3.3.2	88	Add the footnote "For subject 45, items 22, 27, 28, 29 and 30 are excluded from the UPDRS score."
Section 9.1.3	30	LOCF will be used for missing postbaseline PET scan data.
Section 9.9.2.1	44	Add an overall summary and serious TEAEs summary for the initial extension only. Similarly, Add an overall summary and serious TEAEs summary for the pilot and supplemental extensions.
Appendix 2	53	Clarify major/minor deviations for missed infusions. First is minor, second is major if within first 40 weeks, all subsequent are minor.
Table 16.1.2	68	Add footnote "A deviation is counted as major if the subject missed two infusions during the first 40 weeks of the Extension Study."
Table 16.2.3.3.2	88	Remove missing row and columns
Table 16.4.1.1	103	Add total column
Table 16.4.1.2	104	Remove missing row

Figures 16.5.1.1.2, 16.5.1.2.1, 16.5.1.2.2	130	Add modelled control line
Figures 16.5.5.1 - 16.5.5.15	134, 135, 136, 137	Add line of best fit for each treatment group
Listing 17.2.4.12	174	Add unit to title
Section 9.7.3	37	Change all references to the ITT Primary Population to the ITT Overall Population
Figures 16.5.2.1, 16.5.2.2	132	Repeat figures for the ITT Overall Population
Table 16.4.1.2	111	Add total column
Section 9.7.6, Table 16.2.3.3.1	36, 94	Add Fisher's exact test for Week 40 and Week 80.
Section 9.9.2.1	44	Add a TEAEs experienced by at least 3 subjects overall by PT summary for the initial extension only and for the pilot and supplemental extensions combined.

Note to File

**Date:**	17 July 2017
**Sponsor:**	North Bristol NHS Trust (NBT)
**Protocol:**	GDNF 2797 / MDGGNDNFD-GDNFDM
**Study Title:**	An Extension Study to Assess the Safety and Efficacy of Intermittent Bilateral Intraputamenal Glial Cell Line-Derived Neurotrophic Factor (GDNF) Infusions Administered via Convection Enhanced Delivery (CED) in Subjects with Parkinson's Disease

In Sections 9.8.2.3 to 9.8.2.8, SAP v1.0 consistently stated: "Parameters include [baseline / Week 40/e0 / Week 80/e40] OFF state UPDRS [motor / ADL] score and [baseline / Week 40/e0] ^{18}F-DOPA uptake rate constant determined by PET for dorsal central/posterior putamen and for dorsal anterior putamen, using the averages from both hemispheres. Correlations are calculated separately for each treatment group and putamen region combination. The estimated correlation coefficients and p-values are provided on scatterplots, one for each of the 4 correlations analyzed."

The following issues were observed after finalization of SAP v2.0 and the initial production of the TFLs:

1. The following intended change to Sections 9.8.2.3 and 9.8.2.4 of SAP v1.0 was not implemented in SAP v2.0: Correlations are also calculated for all subjects combined, and the estimated correlation coefficients and p-values are provided on scatterplots.
2. Unintentional changes were made to the SAP between V1.0 and 2.0 which suggested that:
   - Correlations in Section 9.8.2.3 would "be repeated just for the GDNF/GDNF treatment group" instead of "be calculated by treatment group".
   - Correlations in Sections 9.8.2.4 to 9.8.2.8 would be "calculated separately for each treatment group" and "be repeated just for the GDNF/GDNF treatment group" instead of "calculated by treatment group".
   - Figures in Sections 9.8.2.3 to 9.8.2.8 would be produced for all subjects and for the GDNF/GDNF group instead of for all subjects and by treatment group (Sections 9.8.2.3 and 9.8.2.4) and by treatment group (Sections 9.8.2.5 to 9.8.2.8), respectively; however, figures were consistently produced by treatment group. For completeness, the figures for motor score (Section 9.8.2.3) and ADL score (Section 9.8.2.4) for all subjects were produced once the discrepancy was detected, but the figures for all subjects for the remaining sections (9.8.2.5 to 9.8.2.8) were not produced as they would not be relevant or meaningful.
   - Parameters in Sections 9.8.2.3 to 9.8.2.8 would include ^{18}F-DOPA uptake rate constant determined by PET 'for dorsal central/posterior putamen' instead of 'for dorsal central/posterior putamen and for dorsal anterior putamen'; however, consistent with the original plan, the ^{18}F-DOPA uptake rate constant determined by PET for dorsal anterior putamen was also used as a parameter.
   - In Sections 9.8.2.7 and 9.8.2.8, figures would assess correlation between Week 80/e40 OFF State UPDRS Motor Score (Part III)/ ADL Score (Part II) and Week 80/e40 ^{18}F-DOPA uptake. However, consistent with the original plan and the figure titles in the shells, correlation was assessed between Week 80/e40 OFF State UPDRS Motor Score (Part III)/ADL Score (Part II) and Week 40/e0 ^{18}F-DOPA uptake.

Details of the changes in the SAP, impact and the analysis method used are documented in the spreadsheet entitled "Changes incorporated into V1.0 10Feb17 not included in V2.0 SAP" dated 17 July 2017.

1.0 Approvals

Name	Signature	Date (dd-Mmm-yyyy)
Jess Read<br>PRA Principal Biostatistician	[signature]	17-JUL-2017
Matthias Luz, MD<br>Chief Medical Officer<br>MedGenesis Therapeutix Inc.	[signature]	17-Jul-2017
