# Supplementary material for: Extended Treatment with Glial Cell Line-Derived Neurotrophic Factor in Parkinson’s Disease
Source: J Parkinsons Dis. 2019 May 23;9(2):301–13. doi: 10.3233/JPD-191576 (PMC6597995; doi:10.3233/JPD-191576)
Supplement: Supplementary Material — Appendix D – Efficacy Outcomes for Primary Stage cohort [file jpd-9-jpd191576-s004.pdf]

Appendix D – Table T1: Efficacy Outcomes for Primary Study Cohort (ITT Primary Population)

Table 2. Efficacy Outcomes

Outcome category / Variable	GDNF (N=17)			Placebo (N=18)			Least squares mean difference vs. placebo (95% CI); p
	Baseline	Week 80	Change	Baseline	Week 80	Change	
**UPDRS (part) scores**							
Motor (III) OFF	35.3±9.4	27.1±7.5	-8.2±6.5<br>-21.7±17.1%	32.2±8.7	23.3±9.3	-8.8±8.1<br>-27.0±24.6%	1.4 (-3.3, 6.0); 0.56*<br>6.0 (-8.6, 20.7); 0.41*
ADL (II) OFF	18.4±6.3	11.4±5.0	-6.9±5.9<br>-34.6±24.4%	16.9±6.1	11.9±6.8	-5.0±4.8<br>-30.5±26.3%	-1.4 (-4.8, 1.9); 0.39*<br>-2.3 (-20.4, 15.8); 0.80*
Total (II+III) OFF	54.3±13.8	38.4±11.0	-15.8±9.1<br>-28.4±13.6%	49.1±11.6	35.3±13.8	-13.8±10.5<br>-28.8±20.8%	-0.9 (-7.4, 5.6); 0.77*<br>1.2 (-11.5, 13.8); 0.85*
**Motor fluctuation diary ratings – hrs**							
Total OFF-time	6.1±1.8	4.5±1.9	-1.5±1.1	4.8±2.3	3.9±2.2	-0.9±2.8	-0.1 (-1.4, 1.3); 0.92*
Good quality ON-time	10.3±2.1	12.0±2.3	1.7±1.6	12.5±2.7	13.2±3.2	0.7±3.1	0.5 (-1.2, 2.2); 0.58*
ON-time with troublesome dyskinesias	0.5±1.1	0.3±1.1	-0.2±0.8	0.5±1.0	0.4±0.8	-0.1±1.2	-0.0 (-0.6, 0.6); 0.91*

* MMRM with baseline variable as a covariate, treatment group and visit and treatment group*visit as fixed effects, and patient within treatment group as a random effect

† ANCOVA model with baseline variable as a covariate and treatment group as a factor

Notes:

(1) One GDNF patient had a conus injury due to a car accident and was included in the UPDRS motor scores without items 22, 27, 28, 29, and 30. The same patient was excluded from the UPDRS ADL and total scores.

(2) Timed tapping numbers are averages of left and right.
